# miR-BAG: Bagging Based Identification of MicroRNA Precursors

**DOI:** 10.1371/journal.pone.0045782

**Published:** 2012-09-25

**Authors:** Ashwani Jha, Rohit Chauhan, Mrigaya Mehra, Heikham Russiachand Singh, Ravi Shankar

**Affiliations:** Studio of Computational Biology & Bioinformatics, Biotechnology Division, CSIR-Institute of Himalayan Bioresource Technology (CSIR-IHBT), Palampur, Himachal Pradesh, India; University of Memphis, United States of America

## Abstract

Non-coding elements such as miRNAs play key regulatory roles in living systems. These ultra-short, ∼21 bp long, RNA molecules are derived from their hairpin precursors and usually participate in negative gene regulation by binding the target mRNAs. Discovering miRNA candidate regions across the genome has been a challenging problem. Most of the existing tools work reliably only for limited datasets. Here, we have presented a novel reliable approach, miR-BAG, developed to identify miRNA candidate regions in genomes by scanning sequences as well as by using next generation sequencing (NGS) data. miR-BAG utilizes a bootstrap aggregation based machine learning approach, successfully creating an ensemble of complementary learners to attain high accuracy while balancing sensitivity and specificity. miR-BAG was developed for wide range of species and tested extensively for performance over a wide range of experimentally validated data. Consideration of position-specific variation of triplet structural profiles and mature miRNA anchored structural profiles had a positive impact on performance. miR-BAG’s performance was found consistent and the accuracy level was observed to be >90% for most of the species considered in the present study. In a detailed comparative analysis, miR-BAG performed better than six existing tools. Using miR-BAG NGS module, we identified a total of 22 novel miRNA candidate regions in cow genome in addition to a total of 42 cow specific miRNA regions. In practice, discovery of miRNA regions in a genome demands high-throughput data analysis, requiring large amount of processing. Considering this, miR-BAG has been developed in multi-threaded parallel architecture as a web server as well as a user friendly GUI standalone version.

## Introduction

miRNAs are now expected to regulate approximately 80% of genes [1]. They are mainly involved in post transcriptional regulation through transcript disruption and translational blockade. miRNAs can be found in the intergenic, intronic as well as exonic regions [2], [3]. In animal systems, once transcribed from the genome either by RNA Polymerase II [4] or by RNA Polymerase III [5], the transcripts (primary miRNAs) are recognized by the Drosha-DGCR8 microprocessor complex. This complex cleaves pri-miRNAs into pre-miRNAs. Thereafter, Exportin transports these pre-miRNAs to the cytoplasmic space, utilizing Ran-GTP transport pathway [6]. In cytoplasm, another RNAse III, Dicer, cleaves the pre-miRNA into mature miRNA duplex [7].

The initial approaches for miRNA precursor discovery relied mainly on detection of the hairpin-shaped structure, which is common to all pre-miRNAs. However, groups like Bentwich *et al* suggested that there are approximately 11 million hairpins in human genome, making it a daunting task to correctly identify miRNA precursor candidates [8]. Some novel features and rules thus became imperative for better identification of miRNAs. Initially, one approach was designed for *C. elegans* [9], on the basis of the degree of conservation of miRNAs across various species. Similar approach was adopted by miRSeeker [10] designed for *Drosophila*. While searching for homology, miRSeeker also considered sequence conservation along with criteria like base pairing and presence of miRNAs in at least one of the arms of the hairpin sequences. Although these tools were important milestones, issues with their accuracy and consistency persisted widely, leading to development of better approaches. Later, Bentwich *et al* developed PalGrade [8], which assigned a stability score to every hairpin, depending upon its secondary structure. It also implemented a scoring scheme based on various features like hairpin length, loop length, sequence repetitiveness, bulge length and type of inverted repeat. Berezikov’s group analyzed genomic regions with conserved profiles, employing Phylogenetic Shadowing, and selected the sequences having ability to form hairpins [11]. Sætrom *et al* identified some miRNA specific properties like structural conservation in miRNA primary transcripts, which might ead to development of better performing precursor identification tools [12].

Most of the initial approaches for miRNA candidate identification relied upon the filter based protocols. These included various combinations of rules derived for stem size, loop size, number and size of bulges, GC content, etc. However, such approaches may not be appropriate, particularly when the instances exhibit deviation from the conservation rule. It has been observed that the multi-variate statistical approaches deliver better than the rule based methods. One such pioneering approach had been Triplet-SVM [13]. There, the authors opined for the need to consider the fact that besides miRNAs, the hairpin structure also exists with several other genomic elements. Therefore, the authors considered psuedo-hairpins for a better model while preparing the negative dataset. The same group also identified a property named triplet element, which captured structural as well as sequence information through support vector models. It resulted into a remarkable increase in accuracy and performance consistency. Subsequently, there was surge in use of different machine learning approaches including Random Forests [14], Bayesian methods [15] and many other SVM based tools, where inclusion of triplet or its variants gained importance. Agarwal *et al* [16] developed a method to discover miRNA precursors while applying context sensitive HMM to model RNA secondary structures. The authors used memory supported probabilist models to construct paired regions as well as symmetrical bulges in miRNAs. Ritchie *et al* developed MirEval [17], which combined windowed structural scanning using Triplet-SVM’s methodology [13] and a protocol to evaluate structural properties. It also implemented phyolgenetic conservation through GERP method [18] and a sequence homology search method introduced by Tanzen and Standler [19]. Using Drosha processing site information along with regular sequence and structural features implemented through SVM, successful identification of miRNA precursors was demonstrated by Helvik *et al* [20]. A recently developed tool MiRPara [21] took a more realistic approach while considering datasets. The authors proposed that the structures and sequences reported in miRBase [22] might have incomplete information for miRNAs, as in actual the precursors could have longer sequences. Therefore, actual precursors might have different structural and compositional features. The authors identified a few region specific sequence and structural features for partial pri-miRNA sequences, which performed well for large number of species.

**Figure 1:**
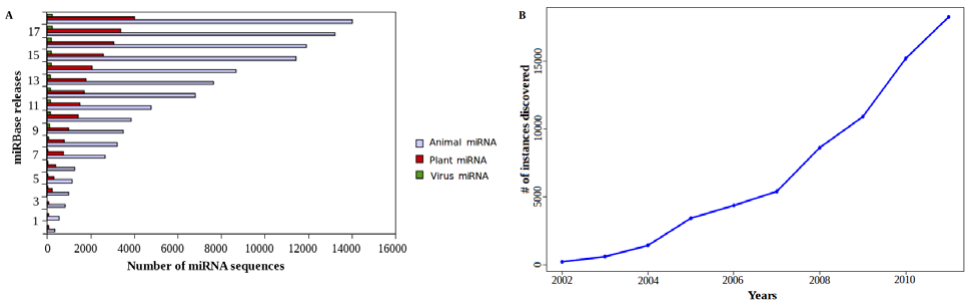
Trend of miRNA discovery with time. (a) Bar Graph depicting the number of miRNAs present in various versions of miRBase in animals, plants and viruses. (b) The line diagram depicts the year-wise growth in the number of reported miRNAs.

As mentioned above, the initial approaches for miRNA discovery had largely relied upon conservation of sequences across various species, homology, hairpin detection and free energy calculation. This resulted into localization of miRNA model building and detection of similar kind of miRNA candidates. Therefore, even with newer approaches, influence of homology would suppress the identification of novel candidates and other unseen properties of miRNAs. Thus, expansion of datasets also became limited. Recent advances with Next Generation Sequencing (NGS) driven technologies helped in guiding the process of miRNA discovery by providing a confidence measure through read mapping to the reference sequences. This also encouraged the genome wide scanning for miRNA candidates with better speed and confidence, reporting novel miRNA candidate regions which were otherwise missed by earlier techniques and tools [23]. Due to such developments, an approximate exponential increase in number of novel miRNA families is notable in the recent releases of miRBase (**figure 1a–b** **)**. Leveraging from breakthroughs made by NGS, recently, some groups have developed tools for detection of miRNA candidates using NGS read data. miRDeep [24] has been one such tools for analyzing data from Illumina Genome Analyzer sequencing platform and for identifying miRNA candidates while considering reads distribution across a reference. The mapped regions are considered to measure the RNA secondary structure based information. Following miRDeep, a few more such tools like miRNAkey [25], miRanalyser [26] and MIReNA [27] have come up.

The present work reports a novel approach to identify miRNA candidates with high accuracy and stable performance over wide range of species. Biologically relevant novel features like miRNA specific mature miRNA guided structural profile matrices and structural triplet density variation profiles with respect to position have been introduced to derive a superior and stable performance. An ensemble machine learning methodology, **B**ootstrap **Agg**regati**ng** (BAGging), has been implemented. It employs complementary classifiers like Support Vector Machine (SVM), Naive Bayes (NB) and Best First Decision Trees (BFTree) to build the final classifier models for large number of species, enhancing the performance strongly. An NGS module has been built to find miRNA precursor candidates, using Illumina read data. The process of miRNA candidate detection requires large volume of sequence data scanning, which makes it dependent upon extensive computing. Considering this, the entire approach has been implemented as a web-server as well as user friendly standalone GUI version, both in parallel architecture.

## Methods

### Datasets

Six different species viz., Human, Mouse, Rat, Dog, Nematode and Fruit fly were used in this study. For the above six species, positive and negative datasets were created. The positive sets included known miRNA containing sequences from the species while the negative sets included pseudohairpin sequences generated from mRNAs, rRNAs, snoRNAs, snRNAs, tRNAs, and SINE elements.

Genomic sequences were downloaded from UCSC [28] and Ensembl [29]. The precursors, mature miRNAs and their co-ordinates were downloaded from miRBase version 18 [22]. The sequences for mRNA, rRNA, snoRNA, snRNA, SINEs and tRNA, along with their co-ordinates were downloaded from UCSC [28] and Ensembl [29]. SINE sequences were collected after running RepeatMasker/Repbase [30].

### Dataset Generation

#### Positive dataset generation

The central base of the terminal loop was treated as the reference point. Mature miRNAs were mapped into the pre-miRNAs and the genomic location of various structural components were recorded. Considering the central base of the terminal loop as the midpoint, genomic sequences up to 200 bp were extracted from the flanking regions. This placed the reference point appropriately at constant position, providing uniformity across all possible dataset instances, irrespective of variation in length, loop size and relative positioning of mature miRNA regions.

#### Negative dataset creation

In the negative dataset sequences, the terminal loops were identified. The middle base of the terminal loop was considered as the reference central position for every negative instance, as in case of the precursors for reference position identification. The negative dataset consisted of different types of RNA sequences including ribosomal RNA, small nucleolar RNA, small nuclear RNA, transfer RNA, SINE sequences and mRNA sequences, all taking pseudohairpin shapes. For all instances, the genomic co-ordinate of central reference position was considered for taking 200 bp sequence with equal flanks. For the dataset of human pseudohairpins, 8,494 pseudohairpins (used by Triplet-SVM) were downloaded and searched across the genomic sequences [13]. RNAfold [31] was run against the mRNA transcriptome data from all target species considered here, to derive the pseudo-hairpin datasets. The sequences used to create the training and testing datasets for all target species are mentioned in Supporting Material **S1**.

### Features Generation and Extraction

**Figure 2:**
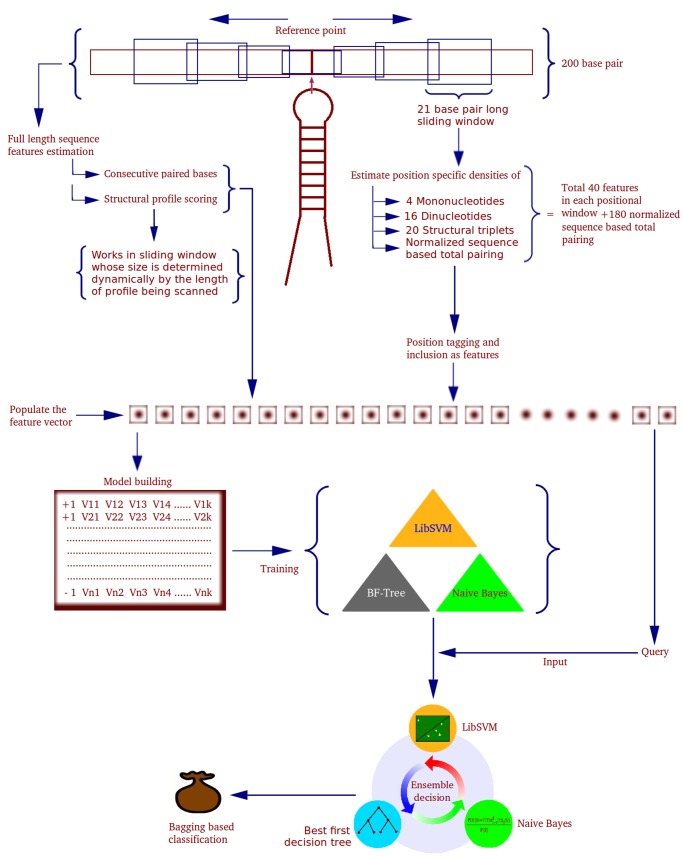
miR-BAG workflow. The complete work flow of miR-BAG, depicting the various classes of features calculated and the classification schema.

The feature data vectors were generated for 200 nucleotides long primary windows, having three different sub-window types, of which two were sliding windows. The features were classified into two broad categories, based on the manner they were estimated. Class I properties were estimated with overlapping sliding windows run over full length of the sequences. Class II properties were estimated for complete length sequences, without the sliding window. A comparative study was performed by changing the window size from 17 nucleotides to 45 nucleotides for deciding the most appropriate window size regarding Class I properties. The best discrimination was observed for window size of 21 nucleotides. Thus, window size of 21 nucleotides with one nucleotide overlap for each sliding window was employed. The major properties considered using the sliding windows were: total normalized hydrogen bondings per window, mononucleotide densities, dinucloetide densities and structural triplet element densities for all 20 combinations of the three structural characters: “ (“, ”)” and “.”. Every such window provided a relative position in a sequence and associated values for the features mentioned above, giving positional feature values with respect to the reference position. The overall work-flow is mentioned in **Figure 2**.

**Figure 3:**
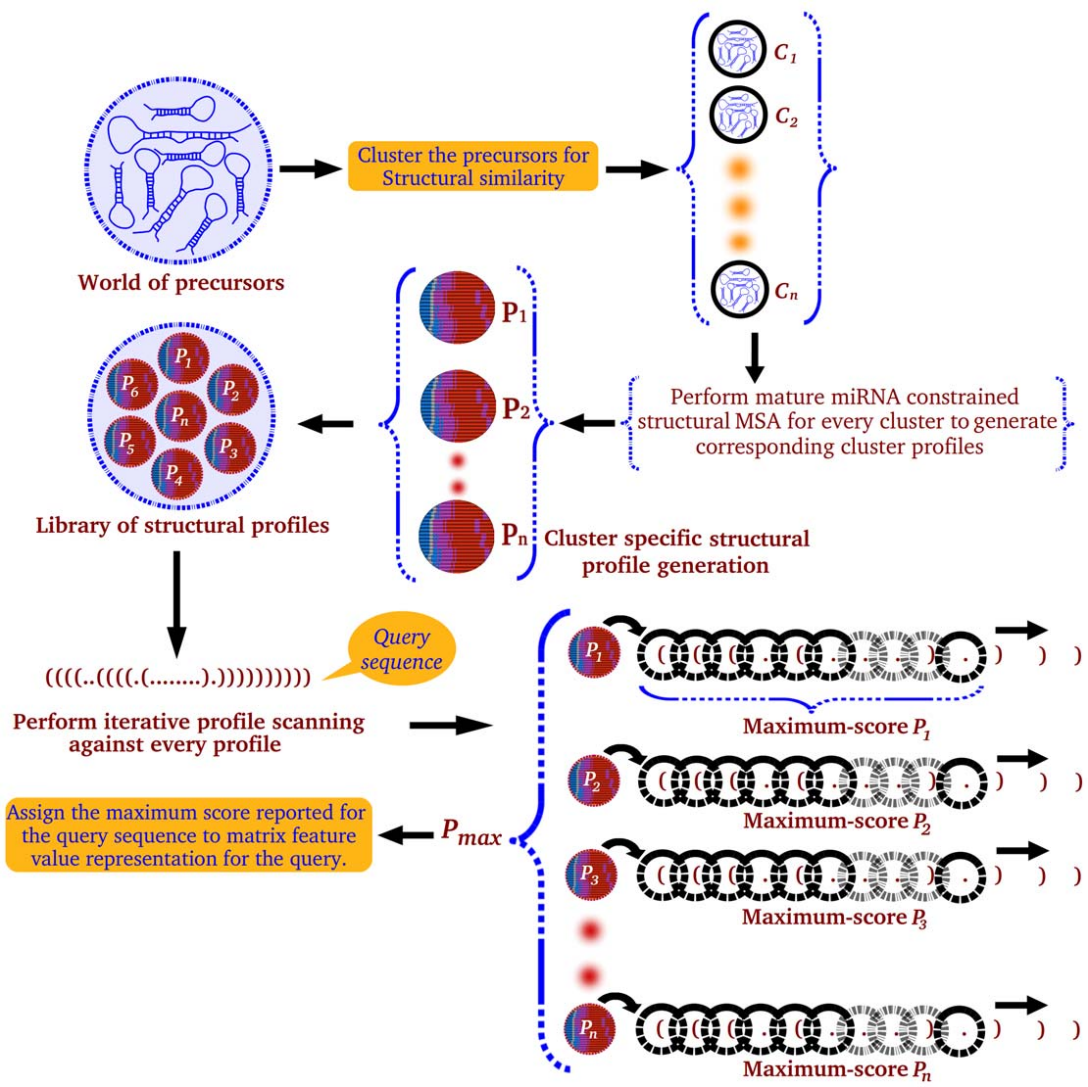
Structural profile matrix derivation and application. The flow diagram represents the steps in structural profile matrix generation and its application.

Class II features were calculated for full length of the sequences instead of the sliding windows. This class includes structural profile based scoring feature and maximum number of consecutive paired bases (CPB) observed in a given structure-sequence. CPB represented the longest pairing stretch observed in a given structural sequence. miRNA specific structural profile matrix based scoring emerged as one of the most critical features observed in the present study. It derives motivation from the fact that miRNA precursors are structurally highly conserved for mature miRNA regions, which are supposed to host a few binding sites for various processing factors in miRNA genesis and localization. For every target species, a library of position specific structural matrices was created utilizing the pre-miRNA sequences mentioned in miRBase version 18. The secondary structures of pre-miRNAs were derived using RNAfold [31]. This was followed by mature miRNA region constrained alignment to evaluate the structural similarity across various miRNA families and develop structural similarity based clusters of miRNAs (**figure 3**).

For developing the structural profiles, information about coordinates of mature miRNA regions was necessary. This information was derived by mapping mature miRNAs into their precursors, which in turn were also mapped into the corresponding secondary structures. RNAfold [31] draws secondary structure of RNA and gives output in dot-bracket form (“(“,”.“,”)”). To distinguish mature miRNA regions from rest of the regions, RNAfold [31] notations were transformed using the following: (“(“−>”M”, “.”−>”O”, “N” −>” )”. For the flanking regions around the mature miRNA region, the transformation was done as: “(“−>”L”, “.”−>”D” and “)”−>R.

The in-house developed scripts aligned the modified structural notations/encodings by aligning first for the mature miRNA regions (containing M,O,N), followed by bidirectional alignment for rest of the structural regions (L,D,R). By doing so, the mature miRNA region constraints and guides a suitable alignment, restoring structural information with respect to the mature miRNA region. Such structural information could otherwise be lost while aligning various precursor sequences end to end (**Figure 3**). In this manner, structural proximities between various precursor molecules were measured and clusters of proximal precursors anchored at mature miRNA regions were formed. For every miRNA, structural alignment was done against the rest of miRNAs with 80% identity cut-off. All closely similar miRNA structures formed miRNA specific clusters, which were multiple-aligned using an in-house developed constrained progressive multiple structural alignment tool. This generated a library of precursor miRNA structural profiles, where every profile contained information about probability of occurrence of a secondary structure state for every position in a more accurate manner. The length of such profile is the distance between the coordinates of the smallest node at the left side and that at the right side of the mature miRNA region. This ensures consideration of most informative and fully aligning regions only. Every putative miRNA candidate is scanned through this library of structural profiles to identify the highest scoring regions. The highest score observed for sequence becomes the representative score for the corresponding full sequence. The same score is also taken as a feature component for machine learning step (**figure 3**). For every profile, the structural sequence is first scanned through a series of overlapping sliding windows. The size of such windows is fixed dynamically by the length of the profile, as described above. Using the above mentioned procedure, a total of 1,411 miRNA specific structural profiles were generated for human miRNAs. Similarly, a total of 727, 320, 393, 222 and 207 structural profiles were generated for mouse, dog, rat, *C. elegans* and *Drosophila* miRNAs, respectively.

Finally, for every input sequence considered with 200 bp length, there were 80 left sliding windows, 80 right sliding windows and one fixed window. Each window had 40 features, 180 position specific structure weighted bond information features and two window independent features (CPB and Profile score), leading to a total of 6,622 features, representing every 200 bp sequence.

Feature selection was performed to identify the most important features contributing to the discrimination process. Feature selection is a process for identification of features, which could ably discriminate between the positive and negative instances. The feature score was calculated using following equation.
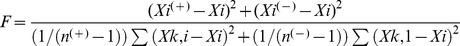
Where,

F  =  Feature score

Xi^(+)^  =  Mean value for *i*-th feature in experimentally validated positive instances

Xi^(−)^  =  Mean value of the *i*-th feature in negative instances

Xi  =  Overall mean value for the *i*-th feature

Xk,i^(+)^  =  Feature value for *k*-th positive instance for i-th feature

Xk,i^(−)^  =  Feature value for *k*-th negative instance for *i*-th feature

The features considered in this study are independent and unaffected from the arm location of the mature miRNA region in the precursor sequence

### Classification

miR-BAG uses an ensemble approach of machine learning, bagging (**B**ootstrap **AG**gregating), to classify miRNA. It employs JavaML [32] and Weka Classifier [33]. Ensemble methods like Bagging have been reported to increase classification accuracy by aggregating the classifications made by multiple complementary classifiers. It overcomes over-fitting, bias-variance and thus, delivers stable performance. Bagging does not focus upon some specific instances or classifier, which makes bagging less prone to over-fitting. Bagging repeatedly samples the data from the dataset with replacement. A combination of different complementary base classifiers forms a committee, which learns from different training subsets, and the component classifiers are tested together for performance. Models generated by the data are combined to form a composite model C*. If a classifier is unstable, bagging tackles it by reducing the errors of the classifier. It has been found that bagging ensures better performance than any single classifier while the possible error is always less than the error by a single classifier. It also minimizes the impact of noise in a given data. miR-BAG bagging classifier has three different classifiers: Naïve Bayes, Support Vector Machine (SVM) and Best First decision tree (BFTree).

Naïve Bayes removes the noisy features of data while estimating conditional probability. A Naïve Bayes classifier estimates the class probability by assuming conditional independence between the features, using the following equation:
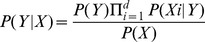
Where:

P(X)  =  Prior probability of observing the feature vector X.

P(Y|X)  =  Posterior probability of the given hypothesis Y (identified class).

P(Xi|Y)  =  Posterior conditional probability of observing *i* ^th^ feature’s value Xi, given prior of hypothesis.

Best First decision tree (BF Tree) expands the best nodes first while creating the tree. The best node is the node whose split reduces impurity. The above mentioned two methods reduce over-fitting and do not need any huge learning set to classify. Also, such classification utilizes each attribute to find a local optimal solution.

The third classifier implemented in the present study is Support Vector Machine (SVM). SVM forms a decision boundary with maximum error margin hyperplanes to classify instances, providing global solutions. The SVM kernel implemented in the present work is a Gaussian kernel, having the following equation:
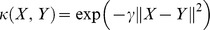
Where, κ  =  Kernel of functions X and Y.

Inconsistency with variable datasets has been an issue with kernels of SVM. A kernel may classify some datasets accurately while on some other datasets its accuracy may decrease. With the bagging approach implemented in the present study, each classifier makes up for others’ demerit in a complementary fashion, bringing stability and consistency along with high accuracy.

The basic bagging algorithm:

Input:

D, a set of training samples.

Let K be the number of bootstrap samples.

C, a vector of learning schemes (Naive Bayes, BFTree and SVM).

Pseudo code:

- 1 for I  = 1 to k do
- 2 Create a bootstrap sample of size N, Di
- 3 for j  = 1 to C do
- 4 Train a base classifier Cj on the bootstrap sample Di
- 5 end for
- 6 end for
- 7 C*(x)  =  argmax Σ_i_ δ(Ci(x)  =  y).

### Performance Measurement

Different performance-measure tests were conducted: 1) Tests with datasets for six different species pre-miRNAs (miRBase version 18) [22]. Above mentioned sources were used to develop the resources for positive and negative instances. 2) Performance comparison with recently published tools: microPred [34], Triplet-SVM [13], CSHMM [16], miRNA SVM [20], MiRPara [21] and MirEval [17] 3) Ten fold cross validation based Receiver Operating Characteristic Curve (ROC) tests for performance over large datasets. The values for accuracy(Ac), sensitivity(Sn), specificity(Sp) and MCC were calculated using the following equations:

Sn = TP/(TP+FN)

Sp = TN/(TN+FP)

Acc = (TN+TP)/(TN+FP+TP+FN)

MCC = {(TP*TN)−(FP*FN)}/√(TP+FN)*(TP+FP)*(TN+FP)*(TN+FN)

Where:

TP  =  True Positives

TN  =  True Negatives

FP  =  False Positives

FN  =  False Negatives

Sn  =  Sensitivity

Sp  =  Specificity

Acc  =  Accuracy

MCC  =  Matthew’s Correlation Coefficient

The Receiver Operating Characteristic Curves (ROCs) with corresponding Area Under the Curve (AUC) were plotted for all models using 10 folds cross validation. Open source “R”-statistical package and ROCR were used to build the ROC plots.

For all training and testing datasets considered in the present study, thorough care was taken to avoid any bias created due to redundancy and common occurrences of instances. All identical entries common to the test and training sets were removed. Therefore, the testing datasets are mostly with instances which were never used during the training step. Also, they were never seen before instances for the classifiers. All these precautions minimized any possibility of bias.

### Implementation of Parallelism

In miR-BAG, divide and conquer approach has been implemented to achieve parallelism. Parallelism has been implemented by applying Java Concurrent Library (JCL). Parallelism has been implemented at three major levels: 1) Sequence 2) Windows and 3) Matrices. Details about implementation of concurrency is given in Supporting Material **S2**. In general, the program has inbuilt capacity to decide the total number of threads to be utilized in a processor, which could be useful in case of processor overload variation.

### Next Generation Sequencing Data Analysis

The FASTQ files for *Bos taurus* transcriptome was downloaded form NCBI database GEO [35]. In this experiment, the authors had reported Illumina reads for 95 different tissues and conditions [GSE21544] [36]. The length of the reads in the FASTQ files was 16 bases. Sequences of miRNA hairpins were downloaded from miRBase version 18 [22], non coding RNA sequences were downloaded from Rfam [37] version 10.0, and the mRNA sequences were downloaded from UCSC [28]. Genomic index for Bos taurus was downloaded from Bowtie database [38]. The downloaded FASTA sequences were converted to Bowtie indexes, using Bowtie-Build. In total, 290,240,521 reads were generated in the experiment. These reads were extracted from the FASTQ files and count of each unique read was recorded. After considering the unique reads only, the number of total reads decreased to 7,434,705. This step was performed to reduce the computation time, memory requirement and processor load. Thereafter, these reads were mapped across the miRNA precursor sequences, non-coding RNA sequences and mRNA sequences to filter out the already annotated reads using Bowtie with 10 hits threshold. The remaining reads which did not map to any of the annotated regions were considered for novel miRNAs identification.

#### Detection of known miRNA

The known pre-miRNA sequences specific to *Bos taurus* were downloaded from miRBase [22] and were mapped back to the genome to find their coordinates. The coordinates were used to extract the sequences with a total of 250 bases, which included the flanking regions. miR-BAG was run with a human specific classifier model.

#### Identification of novel miRNA candidates

The final reads which did not map to any of the already annotated RNA categories were mapped to *Bos taurus* genome. The mapped coordinates were extracted from the Bowtie result file. Using these coordinates, sequences of 250 bases were extracted in FASTA format from the genome file, which was downloaded from UCSC while considering the read mapping region as the center. All these sequences were searched for possible novel miRNA candidate regions, using miR-BAG. MiRNA abundance was measured by applying Read per million read count normalization (RPKM).

### Server and Standalone Implementation

The entire server was implemented in Apache-Linux platform using PHP. Majority of the codes were developed in C++, JAVA and PERL. Statistical processing and calculations were implemented through methods developed in “R” open source statistical package. The standalone GUI version was developed in QT C++. The entire work was carried out in Open Source OS environment of Ubuntu and Fedora Linux platforms.

## Results and Discussion

miR-BAG has been developed to identify miRNA precursors in genomic sequences with high efficiency, accuracy and performance stability. miR-BAG algorithm has applied some novel sequence-structure based features and an efficient machine learning protocol. As mentioned earlier, many of the previously developed tools worked with conventional properties like minimum free energy (MFE), phylogenetic conservation and hair-pin loop detection. In miPred [39], the authors introduced an approach independent of conservation, which was dependent upon the Minimum Free Indexes proposed by Zhang *et al* [40], using single sequence *ab initio* folding. As per the observations made by Gardener and Giegerich [41], MFE might not be discriminating enough, as the reliability of such methods decreases with increase in the length of sequences. In case of miRNAs, this becomes a matter of concern, as finding the TSS and exact precursors is still a major challenge. Considering the limitations of single sequence based *ab initio* folding, such uncertainty may have impact over the accuracy of secondary structure determination process. To analyze the contribution of minimum free energy in distinguishing miRNAs from non-miRNA sequences, energy distribution plots were drawn using RNAfold [31] (Supporting Material **S3**). As could be observed from the plots, there has been a large overlap between the MFE values for miRNA candidates and non miRNA sequences, suggesting insufficiency of MFE as an efficient discriminating property. Thus, single sequence structure derived conventional properties may have some limitations as a good discriminator.

### Consistent Accuracy while Identifying miRNAs for Wide Range of Species

Large volumes of different datasets were created for each species considered in the present study. The process of dataset generation is also dependent upon the availability of sequences for a given species. The positive and negative sets were made by randomly selection of sequences from the parent sets. For positive sets, this consisted of known miRNA containing sequences, while the negative sets consisted of various classes of RNA sequences. Related information is summarized in **Table 1** and elaborated in the implementation section. While creating training and testing datasets, a balance was maintained between the positive and negative sets to prevent any issue arising due to class imbalance. In negative sets, proportionate representation of negative instances from various categories was maintained to ensure a balanced representation. All possible redundancy in the datasets was curtailed.

**Table 1:** Number of sequences taken in testing and training sets.

	Training Set							Testing Set						
	Positive	Negative						Positive	Negative					
		r	sno	sn	t	SINE	ps		r	sno	sn	t	SINE	ps
*Homo sapiens*	584	98	98	98	86	5	250	500	97	97	97	86	5	225
*Canis familiaris*	158	34	34	34	0	0	55	159	33	35	35	0	0	53
*Rattus norvegicus*	195	43	45	44	0	0	60	196	30	30	30	0	0	84
*Drosophila melanogaster*	112	12	24	11	25	0	40	113	13	24	11	25	0	40
*Caenorhabditis elegans*	108	5	25	25	25	0	25	109	5	25	25	25	0	25
*Mus musculus*	348	75	75	75	0	0	123	348	75	75	75	0	0	123

* ps- pseudohairpin sequences from mRNAs, r– rRNA sequences, sno- snoRNA sequences, sn- snRNA sequences, t- tRNA sequences.

All possible sources of redundancy were eliminated while preparing the datasets. The test sets contained never seen before instances.

The developed species models included *Homo sapiens*, *Rattus norvegicus*, *Mus musculus*, *Canis familiaris*, *Caenorhabditis elegans* and *Drosophila melanogaster.* When tested over the experimentally validated instances in the test set, the developed model for human yielded an accuracy of ∼91%. The classifier was able to identify a total of 556 true negatives out of a total of 607 negative instances, approaching a specificity value of 91.59%. The observed sensitivity was at 89.8%, with a total of 449 true positives correctly identified while 51 instances were identified wrongly as false negative instances. Almost similar level of performance was noted for other species (associated details are available in **Table 1** and **Table 2**), exhibiting high sensitivity and specificity values with much smaller gaps.

**Table 2:** Performance of miR-BAG for different species.

Species	TP	FN	TN	FP	Sensitivity (%)	Specificity (%)	Accuracy (%)	MCC
*Homo sapiens*	449	51	556	51	89.80	91.59	90.78	0.81
*Canis familiaris*	143	16	150	6	89.93	96.15	93.01	0.86
*Mus musculus*	302	46	319	29	86.78	91.66	89.22	0.78
*Rattus norvegicus*	184	12	161	13	93.87	92.52	93.24	0.86
*Drosophila melanogaster*	102	11	108	5	90.26	95.57	92.92	0.85
*Caenorhabditis elegans*	97	12	98	7	88.99	93.33	91.12	0.82

* TP  =  True positive, FN  =  False negative, TN  =  true negative, FP  =  False positive, MCC  =  Matthew Correlation Coefficient.

Performance is described in terms of sensitivity, specificity, accuracy and MCC values obtained for each species.

**Figure 4:**
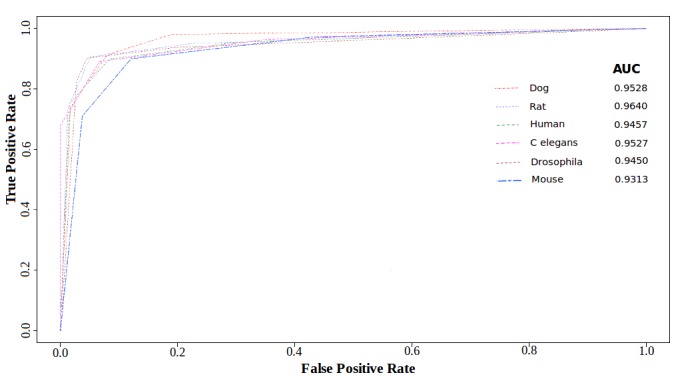
ROC plots for performance with 10 fold CV. The Receiver’s Operating Characteristic Curves obtained for animal model classifiers.

On an average, about 92% overall accuracy was observed for all species considered in the current study, suggesting high accuracy of the classifiers with an average sensitivity of ∼90% and an average specificity of ∼93%. Complete performance break up in species specific manner has been summarized in **Table 2**. Besides this, 10 folds cross validation based performance tests yielded good quality ROC curves for all target species with high AUC values (average AUC value  = 0.94) (**Figure 4**). Similarly, the MCC values for the classifiers also exhibited higher scores for most of the species. The highest MCC was observed for dog and rat with a value of 0.86 (**Table 2**). MCC and AUC are considered as an appropriate indicator for performance, robustness and reliability for a given classification system. These values emerged considerably good for the species models developed in the present study.

The redundancy was curtailed from every dataset considered in the present study and cases common to training and testing datasets for the target species were not removed. Though the subfamilies of a common miRNA family could be present in the training and testing datasets, such condition did not create bias in the classification system. At mature miRNA level, a substantial similarity exists between the mature miRNA regions of instances having a common miRNA family. However, in the present work, information has been extracted from 200 bp precursor sequences. In such sequences, despite of some instances sharing a common miRNA family, the overall identity level is much lower than the identity at mature miRNA level. Still, to assess the possibility of bias introduced due to presence of similar sequences, a test was performed. All relationship instances between training and testing sets which exhibited >80% identity were removed. The performance was analyzed for the new sets without such instances. The total number of such sequences exhibiting >80% identity was found to be much small in number than the total sequences considered for testing. Extremely slight changes in performance was noticed, where the performance drop never crossed even 0.9%, clearly suggesting that the classification system was free from any such bias.

One may still argue that despite of having poor overall similarity, existence of common family led mature miRNA region similarity alone may cause some bias. Though the presented algorithm considers the features contributed by a large region of 200 bp in position specific manner where significant existence of such bias is least expected, a test was carried out to measure any such bias. Considering the human miRNA families, the precursors were distributed in such a manner that no common family existed for any pair of training and testing instances. The training set contained precursors belonging to entirely different miRNA families than those in the testing set (Supporting Material **S1**). A total of 573 positive instances in the training set and 392 positive instances in the testing set were taken. Since the bias was expected to be introduced through the positive instances only, same negative instances were retained in the test and training sets as were taken in the above mentioned tests for human. The new classifier displayed almost a similar level of performance (Sn = 87.7%, Sp = 92.4%, Ac = 90.5%) as was observed earlier, concurring with the above mentioned observation that the classification system does not suggest any significant bias.

### miRNA Specific Structural Profile Matrix as the Top Scoring Feature

Several properties have been used previously to form classification systems which could be able to fish out the miRNA candidates from sequence data. Several tools considered properties as GC content or AU content of the sequence, number of bulges, size of bulges, length of the hairpin and many others to distinguish miRNAs and non miRNAs [8], [34]. A comparative study was performed to calculate the ranges of these criteria. For most of the above mentioned features, there was a large overlap between the various RNA categories considered. For instance, in case of human, the GC content for miRNAs (avg: 0.53), rRNA (avg: 0.49), snoRNA (avg: 0.43), snRNA (avg: 0.40), tRNA (avg: 0.56) and pseudohairpins (avg: 0.56) exhibited large amount of overlaps. The complete information for every species is provided in **Table 3** (Supporting Material **S4** for details). Thus, such features may not be sharp enough to efficiently differentiate between miRNAs and background sequences. Also, we have already discussed above about the implications in considering MFE, a consistent feature in most of the identification tools. Continued application of such features as the central feature might be a reason for the observed performance lag, which some tools exhibited here when tested for different datasets in the present study.

**Table 3:** Average values for conventional features considered for miRNA precursors.

Species	MFE (kcal/mol)	GC content	AU content	Loop length	Stem length	Number of bulges	Hairpin length
Animals	−37.44	0.49	0.51	17	34	3	85
*Homo sapiens*	−40.09	0.50	0.50	16	34	3	84
*Canis familiaris*	−29.23	0.48	0.52	13	28	3	69
*Mus musculus*	−36.80	0.49	0.51	17	36	3	85
*Rattus norvegicus*	−39.49	0.50	0.50	18	36	4	91
*Caenorhabditis elegans*	−34.37	0.42	0.58	19	35	3	89
*Drosophila melanogaster*	−33.00	0.43	0.57	20	37	4	95

The average values for the various conventional features: Minimum Free Energy, GC content, AU content, loop length, stem length, number of bulges and length of the hairpin.

As mentioned before, the structural triplet element features were calculated in sliding windows. Xue *et al* [15] had captured both structural and sequence features, considering full length of the precursor at one go. However, in the present study the triplet structural elements’ density profile variation alone with respect to position could provide more information and discrimination than considering triplet elements with central nucleotide information without positional references. A possible reasoning for such observation could be the fact that more than sequence level similarity, structural level similarity is expected for elements like miRNA precursors. Besides this, the positional information works like a relative structural motif. Our analysis with and without sliding windows confirmed this observation (**Table 4**). Also, it appeared among the top scoring features during feature selection.

**Table 4:** Effect of Structural triplet element on classification.

	Structural Triplet element with windows			Structural Triplet element without windows			Triplet element with central base,in windows			Triplet element with central base, without windows		
	Sn	Sp	Acc	Sn	Sp	Acc	Sn	Sp	Acc	Sn	Sp	Acc
Percentage	89.80	91.59	90.78	75.00	79.57	77.50	90.00	88.46	89.15	76.60	77.42	77.05

* Sn  =  Sensitivity, Sp  =  Specificity, Acc  =  Accuracy.

Inclusion of triplet elements with respect to position appeared more informative and discriminating than considering triplet elements without positional references.

One of the most important parts of a miRNA candidate sequences is the region containing the mature miRNA itself, which controls the target gene’s expressions in spatio-temporal manner [42], [43]. It has been observed that structure of a functionally critical entity is comparatively more conserved than its sequence. Therefore, due to its functional importance, the structure of miRNA containing region might be more conserved than the rest of the precursor regions. Also, some recent studies have suggested that this region harbors binding sites for factors critical for miRNA biogenesis [44], [45]. Though it is just a single feature among the array of various features considered in this study, structural profile matrices introduced in this study emerged as one of the most discriminating features. It relied upon the property of structural conservation in miRNAs. It exhibited high discrimination capacity between the positive and negative instances (p-value <0.0001, Mann-Whitney test at 95% confidence interval). A test for its contribution in the classifiers as a single feature suggested a drop in the classifier performance if the profiles were not considered (**Table 5**). This is a remarkable contribution, considering the fact that structural profiles make just a single feature. This impact was evident across most species taken in this study, suggesting the universal validity of the structural profiles. The structural profile matrices scoring plots were generated for the positive and negative instances, considering all species involved in this study (Supporting Material **S3**). The plots clearly displayed the demarcating ability of matrix scores.

**Table 5:** Effect of structural profile matrix on classification.

	Classification With Matrix			Classification Without Matrix		
Species	Sensitivity(%)	Specificity(%)	Accuracy(%)	Sensitivity(%)	Specificity(%)	Accuracy (%)
*Homo sapiens*	89.80	91.59	90.78	91.00	90.44	90.69
*Canis familiaris*	89.93	96.15	93.01	91.19	95.51	93.33
*Mus musculus*	86.78	91.66	89.22	89.94	87.93	88.93
*Rattus norvegicus*	93.87	92.52	93.24	92.34	91.37	91.89
*Drosophila melanogaster*	90.26	95.57	92.92	85.84	94.69	90.26
*Caenorhabditis elegans*	88.99	93.33	91.12	88.07	93.33	90.65

The structural profile matrix proves itself as an important feature which increases the performance of the classification system by providing a novel biological feature.

Furthermore, feature selection was performed to identify the most discriminating features in every species studied here. Structural profile matrices emerged as the highest scoring top ranked feature in majority of the species, followed by positional triplet structural elements. **Table 6** shows the feature scores and ranks for structural profile matrix feature for various species. The top 15 features and their corresponding F-scores are mentioned in Supporting Material **S5**.

**Table 6:** Feature rankings and F-scores for structural profile matrix for different species.

Species	Matrix based scoring	
	Rank	F-score
*Homo sapiens*	17	0.532
*Canis familiaris*	2	1.162
*Mus musculus*	1	0.813
*Rattus norvegicus*	1	1.029
*Drosophila melanogaster*	49	0.335
*Caenorhabditis elegans*	1	0.722

Rank and feature scores obtained after performing feature selection show the structural profile matrices among the top scoring discriminating features.

Structural profiles were generated using all species specific precursors. Since part of the same set was used as positive instances for testing the classifier, one may doubt for presence of some bias arising due to this. However, possibility of such bias in profile based approaches is very less. The profiles dilute individual identity of instances and promote domain specific group identity, which was verified through some tests. The profiles developed from human specific precursors were applied against cow, rat, and *C. elegans* specific test datasets, which contained precursor sequences specific to these species only (42, 61 and 99 species specific precursors from cow, rat and *C. elegans,* respectively). The datasets contained almost equal amount of pseudo-hairpin instances. The human specific profiles were totally naive to these instances. If a profile memorizes the individual instances used to develop it and imbibes some bias, a poor performance is expected if such profiles are used against never seen before instances. Also, there should not be any major change in the performance if such profiles are not used. Therefore, if there is some bias, the classification system containing human specific profiles should display no change in its performance if the classification system works without these profiles. However, when tested over the above mentioned unseen datasets (containing rat, cow and *c. elegans* specific miRNAs), the same did not hold, and a sharp decline in performance was observed after removal of human specific profiles (**Table 7**).

**Table 7:** Structural profiles provide precursor specific signatures universally.

	TP	FN	TN	FP	Sn (%)	Sp (%)	Acc(%)
**Cow Data**							
Classification with matrix	42	0	36	6	100	85.71	92.85
Classification withoutmatrix	37	5	34	8	88.09	80.95	84.52
**Dog data**							
Classification with matrix	54	7	56	5	88.52	91.80	90.16
Classification withoutmatrix	50	11	57	4	81.96	93.44	87.04
***C. elegans*** ** Data**							
Classification with matrix	94	5	93	9	94.94	93.00	93.96
Classification withoutmatrix	91	8	89	9	91.91	89.00	90.45

For never seen before datasets from cow, rat and *C. elegans*, the classification systems with the profiles developed from human specific precursors performed significantly better than the classification systems without human specific profiles. This suggests that the profiles have domain specific memory instead of instance based individual memory, making them useful for universal characterization of the precursors.

In another test, using human miRNA precursors, the test and training sets were created with entirely different sets of miRNA families (Supporting Material **S1**). No instances from training and test sets belonged to any common miRNA family. The negative instances in both datasets were same as those taken in the above mentioned benchmarking studies for human. The structural profiles were built using the training set precursors only. The resultant classifier’s performance with the new matrices was measured for the test set having totally different set of miRNA families. As observed earlier, the performance remained almost similar (Sn: 88.3%, Sp: 92%, Ac: 90.83%), suggesting no significant bias. About 1% drop in the accuracy was observed when the classifier was run without the profiles. As already mentioned above, such drop is significant one because the profile matrices contribute only as a single feature.

Above results suggest that the profiles provide some structural signatures specific to microRNA precursors, which could be useful for miRNA identification. The applied approach also seems to be free from any significant bias.

### Ensemble Installs a Balance

While developing miR-BAG, three different base classifiers were implemented in an ensemble where each component classifier displayed its own set of merits and demerits. The suitable combination of classifiers was determined through hit and trial selection of classifiers. The combination which maintained the highest balance between sensitivity, specificity, and accuracy was considered as the best one. It was observed that the combination of BFtree, SVM and Naive Bayes not only classified the data with high accuracy but also maintained a good balance between sensitivity and specificity, a highly desirable property for a reliable classification system. To investigate the importance of individual classifier, performance of the classification system was measured separately in absence of each of the component classifiers. Human datasets were considered for this part of the study. When BFtree component was removed from the combination and classification was carried out using only SVM and Naive Bayes, the sensitivity increased to 95%. However, the specificity dropped by ∼17%, causing fall in overall accuracy by ∼7%. Almost similar pattern was observed when the performance was measured without Naive Bayes and SVM components, separately. Sharp drops in specificity and overall accuracy values were clearly visible. Also, huge gaps between specificity and sensitivity values were observed (**Table 8**). Therefore, it is evident that bagging installs a balance between sensitivity and specificity with high overall accuracy, providing a better classification system. The best result was obtained when all three of these classifiers were implemented in a combined form. This is a remarkable property, as in genome wide scanning higher false positive values cause impeding effect. Detailed information is given in **Table 8**.

**Table 8:** The effect of applying ensemble approach with bagging.

Combinations	Sn (%)	Sp (%)	Acc (%)
**All three classifiers**	89.80	91.59	90.78
**LIBSVM and Naive** **bayes**	95.00	74.46	83.73
**BFTree and Naive** **bayes**	93.20	76.27	83.92
**BFtree and LIBSVM**	92.40	80.56	85.90

* Sn  =  Sensitivity, Sp  =  Specificity, Acc  =  Accuracy.

Tested over *Homo sapiens* datasets, it was found that every component/base classifier was contributing in increasing the performance of the ensemble classifier, installing balance in its performance.

### Faster Analysis Over Larger Data through Concurrency

Unlike other miRNA identification tools, miR-BAG runs on single processor as well as on multi-processor machines in concurrent mode. The parallel version of miR-BAG uses Java Concurrent Library (JCL). miR-BAG JCL creates threads, which are equally distributed across the number of processors provided by the user for faster execution. These threads can be executed simultaneously and independently on multiple processors, performing similar operations for different sequences or concurrently solving different parts of a simultaneously existing problem. The execution time of miR-BAG algorithm reduced by manifolds with implementation of concurrency, which also depended upon the number of processors utilized by concurrent processes.

**Figure 5:**
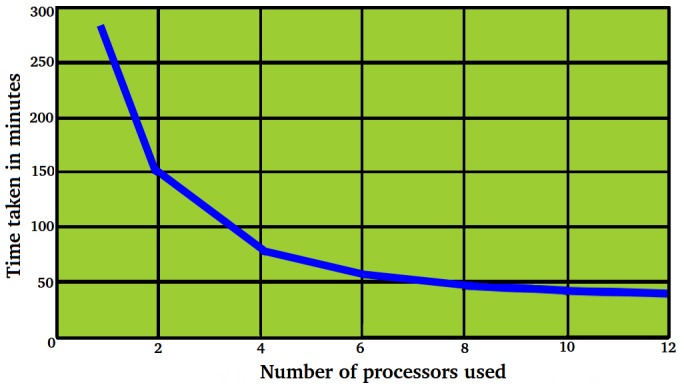
Impact of parallelism. Parallelism causes increase in execution speed, which is highly desirable for large scale data processing. Realistic miRNA discovery requires large scale data processing.

A study was performed to analyze the effect of parallelism upon the classifier’s execution rate where the number of processors varied (**Figure 5**). The result clearly suggested a reasonable increase in the processing speed due to parallelism. For the presented data, the input contained 1,500 sequences, each of length 200 bp. Without parallelism, miR-BAG took 632.66 minutes to process the same job while with parallelism its performance improved to 282.72 minutes for a single processor. The same job took only around 36.37 minutes (0.6 hrs) when executed with 12 processors in parallel. The execution performance for 12 processors increased by 7.77 times when compared to the single processor run (**Figure 5**). In the present analysis, AMD Opteron Magny-cours (12 cores) processors (2.2 Ghz) were used. These observations look important especially after considering the fact that miRNA discovery largely depends upon analysis of high throughput data. At present, hardly any miRNA discovery tool provides any such scope for performing large scale analysis in concurrent mode, on any high end server as well as on any simple desktop machine. Here, parallelism has been introduced at processor as well as at threads level. The system has been designed to automatically decide the number of threads to be utilized from each processor, depending upon the processor load.

### miR-BAG Emerges as a Reliable Performer

miR-BAG was compared for performance with six existing tools for miRNA precursor identification: Triplet-SVM [13], microPred [34], CSHMM [16], MirEval [17], miRNA SVM [20] and MiRPara [21]. The above mentioned human datasets were used. The analyses suggested better performance by miR-BAG as shown in **Table 9**.

**Table 9:** Performance of miR-BAG against six different software on unseen data.

Software	Sensitivity (%)	Specificity (%)	Accuracy (%)
MiRPara	83.80	78.91	81.12
miRNA SVM	43.20	94.72	71.45
MirEval	78.75	80.65	79.73
Triplet SVM	70.20	93.57	83.02
microPred	16.60	67.54	44.53
CSHMM	98.40	24.05	57.63
miR-BAG	89.80	91.59	90.78

miR-BAG performed better than the compared tools.

Triplet-SVM [13] works with triplet structural elements, considering position independent structural as well as sequence based information. When tested, Triplet-SVM exhibited a specificity value of 93.57% and a lower sensitivity value of 70.2%. Thus, the overall accuracy of Triplet-SVM was 83.02% (**Table 9**). Almost the similar issue was apparent for miRNA SVM [20], where the overall accuracy was ∼71%. However, a huge imbalance between its sensitivity (43.20%) and specificity (94.72%) values was conspicuous. This suggested that miRNA SVM has an inclination towards over-prediction of false negatives and overall high prediction of negative instances. Almost the opposite was apparent with CSHMM [16], where the tool was found more inclined towards over-prediction for miRNA candidates. Fort the same test set, CSHMM exhibited a sensitivity of 98.4% while specificity took a dip at 24.05%, resulting into overall accuracy of 57.63%. CSHMM [16] applies memory backed context sensitive HMM to model long distanced pairing between the bases in a RNA secondary structure. However, as discussed above, it appears that accurate identification of miRNA precursors relies more upon measuring multiple model features instead of a few features.

Another tool, microPred [34], performed well (Sn: 90.44%, Sp: 77.72%) when tested over the same dataset which was used to report its bench-marking results (**Table 10**). However, it exhibited declined accuracy for other datasets (**Table 9**). For our benchmarking dataset, microPred displayed 16.6% sensitivity, 67.54% specificity and 44.53% accuracy. microPred [34] considers different types of RNA families in its negative set, including rRNAs, tRNAs and Y RNAs. It considers minimum free energy of folded RNA for a fixed query length. It was found that microPred was extremely sensitive towards the query length and a drastic performance drop was noted if the input length was varied. Variation in input length changed the structural and MFE parameters, the properties upon which microPred relies immensely. As already discussed above, free energy distribution does not emerge as a strong discriminating feature, as a large amount of negative and positive instances overlap with each other (Supporting Material **S3**). With this work, we suggest that the above mentioned limitations of microPred could be fixed if the same process is done with sliding windows of some fixed length instead of considering complete sequence at one go.

**Table 10:** microPred’s performance is sensitive to the query length.

	Total numbersequences	TP/TN	FP/FN	Accuracy (%)
Positive dataset (original)	691	625	66	90.44
Negative dataset (original)	754	586	168	77.72
Positive testing dataset (200 bp sequence length)	263	39	224	14.83
Negative testing dataset (200 bp sequence length)	82	52	30	63.41

* TP  =  True positive, FN  =  False negative, TN  =  true negative, FP  =  False positive.

microPred appears to be sensitive towards the exact length of input sequences. Its performance dropped drastically when it was tested over the dataset with query length of 200 bp. The instances were from the same dataset (original) which was used by the authors of microPred for its performance benchmarking. However, the instances taken in the present study were with longer length (200 bp) than their corresponding entries in the original dataset.

MiRPara [21] considers minimum fold energy of the query sequence along with other parameters. This tool takes a realistic approach while considering the dataset. It reasons that since the submitted precursors might have incomplete sequence, structure of precursor given in miRbase might differ from the actual one. Such consideration looked genuine when MiRPara was tested over the mentioned test set. Though the overall accuracy of MiRPara was found lesser than Triplet-SVM by ∼2%, the balance between its specificity and sensitivity was found better than most of the tools studied here, including Triplet-SVM. Also, MiRPara is available as a stand-alone tool, thus it is useful for genome wide and large scale analysis for miRNA identification (**Table 9**). Another tool, MirEval [17], performed almost equally good and displayed almost similar level of balance between its sensitivity (78.7%) and specificity (80.6%). Its overall observed accuracy was 79.7% (**Table 9**). MirEval [17] applies SVM, considering some structural theories in combination with Triplet-SVM protocol. It delivered sensitivity of 78.7%, specificity of 80.6% and overall accuracy of 79.7%. However, MirEval is available only as a webserver tool, which limits its application for large scale analysis.

Compared to the mentioned tools, miR-BAG performed better with overall accuracy of 90.78% and least difference was observed between the values for sensitivity (89.8%) and specificity (91.59%). An associated comparative ROC plotting has been made available in Supporting Material **S6**. The sequences used for benchmarking these tools are available in Supporting Material **S1**.

### NGS Reads Guided Novel miRNA Discovery in the Cow Genome

**Figure 6:**
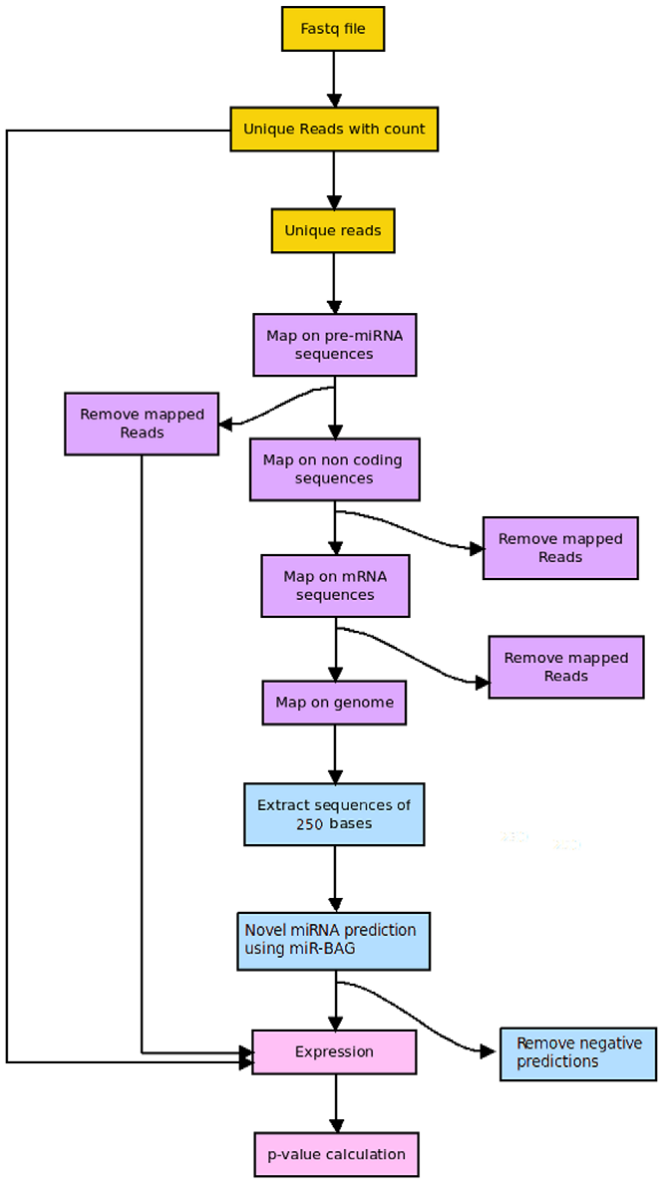
Workflow of miR-BAG NGS module. The flow diagram describes about the various steps involved during NGS reads data led miRNA discovery.

The Next Generation Sequencing data can detect even poorly expressed RNAs, inluding coding as well as non-coding ones. Recently, NGS read data has been used successfully in identifying novel miRNAs [46]. Therefore, it becomes imperative for miRNA discovery tools to provide an approach to detect miRNAs using short read data. Considering this, miR-BAG provides an NGS interface to identify miRNA candidates in genomes. This module utilizes short read data directed reference mapping for guiding the process of precursor identification. The working principle assumes that within a 75 bp region in a given genomic sequence, a miRNA candidate region would display two peaks for the mapped reads. The NGS module of miR-BAG was used to discover novel miRNA regions in *Bos taurus* genome. *Bos taurus* has a genome sequence released with lesser number of known miRNAs compared to extensively studied vertebrates like human and mouse. It is an economically important species, which is also a driving force for large scale industries like dairy. Apart from this, its products are also used as a bioreactor for production of various vaccines and other items [47], [48]. *Bos taurus* has an average genome size of 2,670,422,299 bp and a total of 31 chromosomes. The draft assembly for *Bos taurus* was released in year 2009 [49]. Only 662 pre-miRNAs are reported for *Bos taurus* in miRBase version 18 [22]. Out of this, only 42 pre-miRNAs are specific to Bos taurus. Using human model, miR-BAG-NGS module and *Bos taurus* specific miR-seq reads data, miR-BAG correctly identified all 42 *Bos taurus* specific miRNA precursors, suggesting highly reliable performance. The overall basic work-flow for miR-BAG NGS module is explained in **Figure 6**.

Encouraged with this, *Bos taurus* genome was scanned for potential novel miRNA candidate regions using available Illumina miRNA-Seq read data. miR-BAG NGS module was applied for the same purpose. Associated details are described in the implementation section. Out of 7,434,705 unique reads, 7,332,970 reads were mapped across the mRNA sequences of *Bos taurus*. Remaining 101,735 reads did not map to any mRNA. Such reads were mapped across a number of noncoding RNA sequences downloaded from Rfam. Only 34,253 reads mapped to Rfam sequences. Remaining 67,482 reads were searched against *Bos taurus* pre-miRNA sequences. Total 1,952 reads were mapped on already known pre-miRNA sequences from *Bos taurus*. Remaining 62,447 reads which did not map to these non coding RNA sequences were mapped across the genome for novel miRNA precursor discovery. Finally, miR-BAG identified total 22 novel miRNA candidate regions in *Bos taurus* genome, well supported by read mapping based abundance assessment for expression for those regions. All these candidate sequences scored a value of one, which suggests highest confidence. These candidates may be considered positively for further experimental work and to unravel miRNA regulatory networks in cattle. Information regarding the identified potential novel miRNA candidate regions has been made available in Supporting Material **S7**.

### Availability

The webserver and standalone versions for miR-BAG are freely available at: http://scbb.ihbt.res.in/presents/mirbag. The standalone version (Linux based) can be downloaded from the download tab of the server page. The package comes with an easy to do auto-installer, which automatically installs the package. Details about the server and stand-alone implementations are given in Supporting Material **S8**.

### Conclusion

It has been evident in recent years that large number of miRNAs display substantial migration from the traditional rules for miRNA identification. Moreover, novel miRNAs discovered with the help of Next Generation Sequencing (NGS) data and other technological advances suggest that there may be several miRNAs candidates which are yet to be identified. This lag could be due to a continued dissemination of certain traditional classification rules, causing a plateau phase in the area of miRNA discovery. The current work presented a view on this matter and demonstrated that the approach presented here worked well for a wide range of datasets from different species. Some recent machine learning methodologies with novel biological observations have reported good performance. However, in the present work it was found that several of such methods could not display the required performance consistency and balance between sensitivity and specificity. Wide gaps between sensitivity and specificity evident in some erstwhile approaches made imperative to develop an approach which could reduce the consistency issue and the gaps between sensitivity and specificity values, with best possible accuracy. Through the mentioned novel approach, this study suggests that such goals are achievable. Further, inclusion of novel biologically significant properties may improve the reliability of miRNA discovery tools. The same was observed in this study when structural profile matrices and consideration of positional variation were introduced as the novel features. All associated theories and implementations were tested comprehensively for a wide range of datasets and species, confirming universality of the presented approach for a wide range of species, with high reliability. Through extensive and variable benchmarking steps, we could conclude that miR-BAG’ performed better than the compared tools. The area of miRNA discovery requires scanning of large volume of sequences and reads data, making it essential to develop new tools with high processing speed. Considering this, two required but not so common features have been implemented in the presented tool: A) Implementation of concurrency at all levels B) Availability of miR-BAG as a web server as well as a stand-alone user friendly GUI version. Besides this, miR-BAG has an inbuilt next generation sequencing associated pipeline to identify novel miRNAs across a genome. This all makes it an important miRNA discovery tool and a desirable resource in the field of miRNA biology and regulatory studies.

## Supporting Information

Supporting Material S1 **Total sequences used in training and testing datasets of miR-BAG for different species and benchmarking of various tools.**
(DOC)

Supporting Material S2 **Implementation details for parallelism in miR-BAG.**
(DOC)

Supporting Material S3 **Energy and Structural profile matrix score plots drawn between the positive and negative instances for all target species, depicting the distribution patterns for positive and negative instances.**
(DOC)

Supporting Material S4 **The statistics of all conventional features used by previously existing tools.**
(XLS)

Supporting Material S5 **The top 15 feature scores along with the feature names and type of the features obtained after feature selection for every species.**
(DOC)

Supporting Material S6 **Comparative ROC plots for different precursor identification tools.**
(TIFF)

Supporting Material S7 **Sequence data for novel miRNAs identified in** ***Bos taurus*** **genome, using the NGS module of miR-BAG.**
(DOC)

Supporting Material S8 **Web-server and standalone implementation details for miR-BAG.**
(PDF)
