# Supplementary material for: miR-BAG: Bagging Based Identification of MicroRNA Precursors
Source: PLoS One. 2012 Sep 25;7(9):e45782. doi: 10.1371/journal.pone.0045782 (PMC3458082; doi:10.1371/journal.pone.0045782)
Supplement: Supporting Material S1 — Total sequences used in training and testing datasets of miR-BAG for different species and benchmarking of various tools. (DOC) [file pone.0045782.s001.doc]

**Supporting Information S1 : Sequences used in the testing and training files as well as in benchmarking of differetn tools**

*Homo sapiens testing sequences*

*>hsa-mir-3942*

*UACCCGAAUAGCUCACUGUUCACCUCGUUGUUUUGGUAUUUGAUUGGUCUUCAGUAUGACACCUCAAAGAAGCAAUACUGUUACCUGAAAUAGGCUGCGAAGAUAACAGUAUUUCAGAUAACAGUAUUACAUCUUUGAAGUGUCAUAUUCACUGACACUGAAUAAAUAUGGUGCAUUUUCCUUCCCUUUCCUUCCCUUUU*

*>hsa-mir-626*

*UCAUCCUGAUAGAAUCUCUAGUACAAGUAAGUAGACGUGGCAAGAACUGAUAUAUUUGUCUUAUUUGAGAGCUGAGGAGUAUUUUUAUGCAAUCUGAAUGAUCUCAGCUGUCUGAAAAUGUCUUCAAUUUUAAAGGCUUCUUUUGGUAGGUAUAGAACAUUAGUUUUUCUUUCAACACUUCAUGAUUGGCGGUAAAGUAU*

*>hsa-mir-4264*

*AACCUUUUUCAAACUCUAAUGGAAUCACAAGUGGCAUAAUAAUGAUGAUGAACACGGAAGUUCUGUAGGAAAGCUGGAUACUCAGUCAUGGUCAUUGUAACAUGAUAGUGACAGGUACUGGGUAAGACUGCAUAGAGAAUUUCCAUUAUAACAUGGGCAUGCUUGUAGACAUACGCUGAUAGAUCAAGGAAGGCACAUAA*

*>hsa-mir-3938*

*AAGAAUCCAUGUACCUUUACCCUCAUUAAUUCUGCUGCAAGAUUUUUUUAAAGGAAUUUUUAACCCGAUCACUAGAUUAUCUACAAGGGAAUUUUUUUUUAAUUUAAAAAAUUCCCUUGUAGAUAACCCGGUGGUCAGGUUGGAUGGCUCCAUGGAGGUCAUGAGGUGUAGAGCUGUAACUAGGAGAACUUGCUGUUGUG*

*>hsa-mir-559*

*UAGUAUCCCUAAAACAUUUGAGUUUUCUUAUCCUGGAGAACCUGUCCCUGUGGAUGAGCUCCAGUAACAUCUUAAAGUAAAUAUGCACCAAAAUUACUUUUGGUAAAUACAGUUUUGGUGCAUAUUUACUUUAGGAUGUUACUGGAGCUCCCAUCUUCUCUGCUUUAAGGAACUAGUCCUUAACUAGUUAGCCCUUACUU*

*>hsa-mir-4330*

*UAUGAACUAUCUGUUUCAGAGAGGAUAGAAGCACCAGAACUACUAGCUAAUUGUCAGCAGGCAAUUAUCUGAGGAUGCAGGAGAGGAAGGGGGCUUCUUUUUGACGCCUACUUCAUCAGCUGCUCCUCAGAUCAGAGCCUUGCAGGUCAGGCCAGAGGGGAGACUCAGGAAGUGUUUUUGGUGUUUGUUCCAAAUGGGGG*

*>hsa-mir-422a*

*UCAUUGGUGAGCAUAUAAUACUGUAUGUUGCUGGGGGAAGCCCACAAUGUGAGGACACGAGAGAAGCACUGGACUUAGGGUCAGAAGGCCUGAGUCUCUCUGCUGCAGAUGGGCUCUCUGUCCCUGAGCCAAGCUUUGUCCUCCCUGGGCCUCAGUUUCCUCCUUUGUUCAUUUAGGCAUGGCCUCCAGGGCCCCUCCUG*

*>hsa-mir-3676*

*CUGACCUGGCCAAGGAAACAAGAUCCUAAGCGUCUUUCCGGCGGCGCCGUGGCUUAGUUGGUUAAAGCGCCUGUCUAGUAAACAGGAGAUCCUGGGUUCGAAUCCCAGCGGUGCCUCCGUGUUUCCCCCACGCUUUUGCCAACAUUAAACAUUGUGAGGACAGUUGCAGAAACUCAUAACUUCCAUCCUACAUGGUUUAC*

*>hsa-mir-3673*

*AUGUGGAAUAUAUAUAAGGAAUAUAUAUAUAUGUGGAAUAUAUAUACGGAAUAUAUAUAUAUGGAAUGUAUAUACGGAAUAUAUAUAUAUAUGGAAUGUAUAUACGGAAUAUAUAUAUAUAUGGAAUGUAUAUACGGAAUAUAUAUAUAUAUGGAAUGUAUAUACGGAAUAUAUAUAUAUGGAAUGUAUAUACGGAAUAU*

*>hsa-mir-589*

*CCAGCCUGAGAGACCGACCCUGCUCAUAAUGCCAUCUCUGUGGACAAACUCCAGCCUGUGCCCAGCAGCCCCUGAGAACCACGUCUGCUCUGAGCUGGGUACUGCCUGUUCAGAACAAAUGCCGGUUCCCAGACGCUGCCAGCUGGCCCCGUUCCUCUGGAUUCCUGCCUUCUGCGGCAAGGGAGCCCGGACGCUGCAGU*

*>hsa-mir-301b*

*CACCCAUCCAUGGUUGAGCUUCCCGCCCUGCGUGUAUUGGGGGUGGUGGGGUCCCCCCUGCUGGCCGCAGGUGCUCUGACGAGGUUGCACUACUGUGCUCUGAGAAGCAGUGCAAUGAUAUUGUCAAAGCAUCUGGGACCAGCCUUGGGGAUCUCCCUCCCUAUAACCCUCACCUCCCACUCCCCAGGCAGGGCCCCUUG*

*>hsa-mir-223*

*AUCUCCCAGGAAGAUCUCACUUCCCCACAGAAGCUCUUGGCCUGGCCUCCUGCAGUGCCACGCUCCGUGUAUUUGACAAGCUGAGUUGGACACUCCAUGUGGUAGAGUGUCAGUUUGUCAAAUACCCCAAGUGCGGCACAUGCUUACCAGCUCUAGGCCAGGGCAGAUGGGAUAUGACGAAUGGACUGCCAGCUGGAUAC*

*>hsa-mir-95*

*CCACCUGCACCCCGGGACGUCCAUCUGUAGCGCGCCCAAGGAAGGUAGGAUUGUGACACCCAACACAGUGGGCACUCAAUAAAUGUCUGUUGAAUUGAAAUGCGUUACAUUCAACGGGUAUUUAUUGAGCACCCACUCUGUGCCAGACGCUGAGCGGGGCGCCGAGGGGGACAGAGAAGACAAGAGCAGCCCCUGACCUG*

*>hsa-mir-4310*

*AGGGCUCAUUUACCAGGUGCUGCCCGCCCCACCCCUGAGGCCAGCCUCCUGGGAAUGGCUGGGGGCUAGGCCUGUGGCGUCUGGGGCCUGAGGCUGCAGAACAUUGCAGCAUUCAUGUCCCACCCCCACCAGGCUGAGGACUGGAUCCAGGCGUGGGCCCAGCAGCUGAAGGAGCCGGUCCCUCCUGGGGACCUGAGAGA*

*>hsa-mir-3660*

*AUCAAAGUUUCCGUAGCCAGCUGGAGAAGGAGAAAUGCAGAGUGAAAAGGAGAAAGAAGAACUGGACAAAAUUAAAAUGCUCUUCUGUCAUUGUAAUAGUUCAUAUGGGCACUGACAGGAGAGCAUUUUGACUUUGUCAAGUGUGUCUGCUACUCAUUUAUUCCAUCUCCUGCUUGUUACCGAAGUUUCAGAUUAAAUGU*

*>hsa-mir-149*

*CCCGAGGCUCCCAGGCCUUCGCCCGCCUUGCGUCCAGCCUGCCGGGGGCUCCCAGGCCGGCGCCCGAGCUCUGGCUCCGUGUCUUCACUCCCGUGCUUGUCCGAGGAGGGAGGGAGGGACGGGGGCUGUGCUGGGGCAGCUGGAACAACGCAGGUCGCCGGGCCGGCUGGGCGAGUUGGCCGGGCGGGGCUGAGGGGUCG*

*>hsa-mir-4313*

*CAUGGGAGGCCCUCCACUUUAGCACCUUGACAAGAAGGGUGGGGACACUGGGAUCAGGCCCAGCCCCCUGGCCCCAAACCCUGCAGCCCCAGCUGGAGGAUGAGGAGAUGCUGGGCUUGGGUGGGGGAAUCAGGGGUGUAAAGGGGCCUGCUCACUCCUCAGCUUGGCUCAAGGCUCAAGGCAUCAAAGGCUCAGCCUGC*

*>hsa-mir-3186*

*ACUGAGGCCUCGUGCACGGAAGCUGCUGAAGUUCAACCGAGUUGAAUGCUGUGGCUCAAGCCUGCGGUUCCAACAGGCGUCUGUCUACGUGGCUUCAACCAAGUUCAAAGUCACGCGGAGAGAUGGCUUUGGAACCAGGGGCUUCAGCACCUGGGCAUCACUCCAUCCAGGUGGUGGAGGCUGGCGUCCCCCAGCAGCUA*

*>hsa-mir-297*

*AUGGCCACCUGCCUGAACGAUGGCUUAUUUCCAACUCAAGAUCUCUUUCUACAUACUUUAUGAACAUAUGUAUGUAUGUGUGCAUGUGCAUGUAUGUGUAUAUACAUAUAUAUGUAUUAUGUACUCAUAUAUCAAGGAGAUUUCAUUUCUAUUUUUAAUCUAUGGUAUAACUUCUGCAUUGAUCAUAUAGUCAUGAACCA*

*>hsa-mir-3686*

*CUCUCCCUGUGCUGUGGCCUGGAAACUGUCUGUGCGGUCAGCUGGGGCAGUCAGAGGGCUCACCUCAUUCAUUUACCUUCUCUUACAGAUCACUUUUCUGCACUGGACAGUGAUCUGUAAGAGAAAGUAAAUGAAAGAGGUGAGACUUAGGUUCAAUGUCUCAAAAAUGUUUCAUGUGUUUUAAAUGCUGGCUUUUUAGU*

*>hsa-mir-1538*

*AGGCGCGGAGGGGGGUGCCCGGGAGCGCGGAAGAGCCGUGGCGGCCCCCGGAGGGCGGGGAGGCGCGCCAGGGAACAGCAGCAACAUGGGCCUCGCUUCCUGCCGGCGCGGCCCGGGCUGCUGCUGUUCCUGAGCCGGGAAAGGGCCCCGCUGCCAGGGUGGGAGGGGGGGAGGCUGGGAGGUGGCCGCGCGCUGGGGCC*

*>hsa-mir-4261*

*CAGUGCCCAAGUCCACAGUGGGACUGACCCCAGACAGGCCCCCUAAUACUUUCAGUCUUCCCCUGCUGGGUGGUGGAAGUGGGUUCCUCCCAGUUCCUGAGACAGGAAACAGGGACCCAGGAGACCAGCCCCAGCGAGGGGGUGCCCAGCCGCCUCUCCCGGCCCCUUUUCCACUUAGGCUUGGGCUACCUGAGACUGCU*

*>hsa-mir-3937*

*AGCCCACGUAGAGAGGCCAAUUAGAGAGGCCCUGAGACCGUGUGAAGAGAGAAGAAUGCCCAACCAGCCCUCAGUUGCUACAGUUCCCUGUUGUUUCAGCUCGACAACAACAGGCGGCUGUAGCAAUGGGGGGCUGGAUGGGCAUCUCAAUGUGCUUCUCCCACAUUGCUUCAGCCACAGUCUAAUUGUAACCACAUGAG*

*>hsa-mir-4266*

*AGUCUCUCUCCACUCAUCUCCCAGAAUGUUUUCUUCCCAUCCCCCUGGGAUGUUGAGCCUGAGAGGCAGUGAGCCCCACUGCUGGCCGGGGCCCCUACUCAAGGCUAGGAGGCCUUGGCCAAGGACAGUCCCUAUCUGGGCCUCAAUGUCUUCCUCAGUGAUGCUUAAGACCAUUGCGAUGACCAACUGGUACAGCGCUC*

*>hsa-mir-621*

*CCUUUACCAGCUUCCCUUCUCUCCCAAAGAACUUCCCUUCUUGGGCUUUAGAUUGAGGAAGGGGCUGAGUGGUAGGCGGUGCUGCUGUGCUCUGAUGAAGACCCAUGUGGCUAGCAACAGCGCUUACCUUUUGUCUCUGGGUCCUGGCCUGGGGCCAUCAAUCCACUUUGGGCCACUCACUGUCUGCUCUGCCUCCACCA*

*>hsa-mir-412*

*AGGCCGUCUUGGAGGCUGGGGCACCUCGGGGAAGGACGCCGGCAUCAGCACCAUUCUGGGGUACGGGGAUGGAUGGUCGACCAGUUGGAAAGUAAUUGUUUCUAAUGUACUUCACCUGGUCCACUAGCCGUCCGUAUCCGCUGCAGCCUGUGGGGCCUGCGGGCCGGGGAGCCGAUCGCGCUUCAGCUCAGCGCCUUUCC*

*>hsa-mir-208b*

*CAGGCGAUGCUUAGCUAGUGUUUGACAACACAGGAGGACUCUGCCCCGGCCCCACCUCCUUCUCCUCUCAGGGAAGCUUUUUGCUCGAAUUAUGUUUCUGAUCCGAAUAUAAGACGAACAAAAGGUUUGUCUGAGGGCAGAGUGCUUCCGUCUGGGGCAGGGCACUGUGGCAGGGAAAGGCAGUGGGGAGGGCUGCAGAA*

*>hsa-mir-532*

*UCCAAGGCAUCUGGGAUACGCAUGCACACAGUAUACAGGAGAGCAGACCUCCCGACUUGCUUUCUCUCCUCCAUGCCUUGAGUGUAGGACCGUUGGCAUCUUAAUUACCCUCCCACACCCAAGGCUUGCAAAAAAGCGAGCCUUCUCUUCCCUCUCCAGUGCAUAGGCAUGAGCACAUGGACAGGUACACCAAAGGGAGG*

*>hsa-mir-2277*

*UUGCAUUAUUUCUAGAUGAGUGCAACUGUCAAAGCAAUAUGGGUUCACUGGUCGUGCUUCCUGCGGGCUGAGCGCGGGCUGAGCGCUGCCAGUCAGCGCUCACAUUAAGGCUGACAGCGCCCUGCCUGGCUCGGCCGGCGAAGCUCUAAUUGCCCUGAAGGAGACCGCGCGGGCGCUGCGGGUCCGGCGGCGUCGGCGCG*

*>hsa-mir-203*

*CAAGGGCGUCGGGGGCUCCUCUCUCCGCAGCUCGGCGAACCGACGGUGUUGGGGACUCGCGCGCUGGGUCCAGUGGUUCUUAACAGUUCAACAGUUCUGUAGCGCAAUUGUGAAAUGUUUAGGACCACUAGACCCGGCGGGCGCGGCGACAGCGACGGAGCGUCCCACGCGCGGCCUGGAGUCAGAGUCACAGUCAGGGG*

*>hsa-mir-17*

*UUUCAAAUUUAGCAGGAAAAAAGAGAACAUCACCUUGUAAAACUGAAGAUUGUGACCAGUCAGAAUAAUGUCAAAGUGCUUACAGUGCAGGUAGUGAUAUGUGCAUCUACUGCAGUGAAGGCACUUGUAGCAUUAUGGUGACAGCUGCCUCGGGAAGCCAAGUUGGGCUUUAAAGUGCAGGGCCUGCUGAUGUUGAGUGC*

*>hsa-mir-3691*

*CUUGAGGCUCAUAAAGGUCUUUGCUUCUCCUUCCUGCUUUUACGUUUCAGUGAAUAUUGAGGCACUGGGUAGUGGAUGAUGGAGACUCGGUACCCACUGCUGAGGGUGGGGACCAAGUCUGCGUCAUCCUCUCCUCAGUGCCUCAAACGGUCUGGGAGCCAAUGUUCAACAAACGAGCAUGCGGGUGAGUAAAACACAAC*

*>hsa-mir-497*

*UGGUGCUGGGGUCUUCCCAGCACUGCUAUGUGCUCUCUUCCUUUCAACCCACCCCGGUCCUGCUCCCGCCCCAGCAGCACACUGUGGUUUGUACGGCACUGUGGCCACGUCCAAACCACACUGUGGUGUUAGAGCGAGGGUGGGGGAGGCACCGCCGAGGCUUGGCCCUGGGAGGCCAUCCUGGAGAAGUGACACAAAAA*

*>hsa-mir-1255a*

*UGUCUUGGGGUGGCUGUUGCUUUAUCAGCUAAAUGUAUGUAGUAUUGGAAAUCCUUUGAGUUGCUUCUCAAGGAUGAGCAAAGAAAGUAGAUUUUUUAGAUUCUAAAGAAACUAUCUUCUUUGCUCAUCCUUGAGAAGCAACUCCUUAUCCAUUAAAAUUUUAUCAUGGGAUUGCACCAAGUCAGUCCUAUCUUCAGGAU*

*>hsa-mir-548v*

*UCAUGCCCAGAUGCUGUAGCCGUACAUAAAGAGAUGGAGGAGAAGCAAGAGGGAUUUAUUGAAUACUAGGUUUGAGCAAAAGUAAUUGCGGUUUUGCCAUCAUGCCAAAAGCUACAGUUACUUUUGCACCAGCCUAAUAUUUACUAAGUGCCAGAUAUUCUUACUUACCUUAUUUAAAUCCUCACCAAAGACACGAGUUG*

*>hsa-mir-1256*

*CAAAUUAAAGUCACCAGCUACAUUAGCCCCUAACAGUAGAGUCAGCCUGUUGAAGCUUUGAAGCUUUGAUGCCAGGCAUUGACUUCUCACUAGCUGUGAAAGUCCUAGCUAAAGAGAAGUCAAUGCAUGACAUCUUGUUUCAAUAGAUGGCUGUUUCAUCUACACUGAAAAUCAUGUGUUUAGUGUAGGCACUUUUAUCA*

*>hsa-mir-3649*

*GGUGAAGGUUAGGUGGCACGUUGGCGGGACAUGUGACUCACAUGCGCAGGUGUGUUCCUUGGGAGGAAGGCUUGGAACAGGCACCUGUGUGUGCCCAAGUGUUUCUAGCAAACACAGGGACCUGAGUGUCUAAGCACAGGCCCACUUUACAGGGGUCUCCUGAGAACUCCUCCCCCUCCUGAGAACUGUGCCUUCCCAGC*

*>hsa-mir-1263*

*GAAGGGCAGCAAAAGCUUCACGGGAACGUACUGCUUCUAUGACUCUCUAAUUCCUUCAGCUACCCCAAAAUAUGGUACCCUGGCAUACUGAGUAUUUUAAUACUGGCAUACUCAGUAUGCCAUGUUGCCAUAUUUUGGGGUAGCAUGCUGUAAGUCCCAUCAGUACCAAGUCCAUUCUUAUUUAUAGUCCCACUAUGAGU*

*>hsa-mir-466*

*UAAGUAUACAAAACUUUAAAUGUACAACUUUAUGAACUUUUAUAUGUGUAUAUUUGUAUGUGUGUGUAUAUGUGUGUUGCAUGUGUGUAUAUGUGUGUAUAUAUGUACACAUACACAUACACGCAACACACAUAUAUACAUGCACACACCCACGUAAUGGCACCUAGAUCAAGAUACAGAACAUUUACAACACCACAGCC*

*>hsa-mir-374b*

*CUGGUGCCUGGGAGGACUUCAUGGAUCCAGCAGCAACCUGGAGUGGUGCUCCUCUGAAGAAAUCCUACUCGGAUGGAUAUAAUACAACCUGCUAAGUGUCCUAGCACUUAGCAGGUUGUAUUAUCAUUGUCCGUGUCUAUGGCUCUCGUCUACCAGACUUUAAAUUCCUUAAGGGCAAGGACAGUGCCUUACUCAUCUUU*

*>hsa-mir-28*

*CCUUUUGCAUGGUAGACAACAUGCUUGAUGCUGAAGAUACAAGAAAAAAUUUAAAAUGGUCCUUGCCCUCAAGGAGCUCACAGUCUAUUGAGUUACCUUUCUGACUUUCCCACUAGAUUGUGAGCUCCUGGAGGGCAGGCACUUUCGUUCAUCUGAAAAAGAGCUUAAAUUUCAGUGUUAAUCCUAGAUUACAAUCCCGC*

*>hsa-mir-369*

*UGCAGCCUGUGGGGCCUGCGGGCCGGGGAGCCGAUCGCGCUUCAGCUCAGCGCCUUUCCUGGUACUUGAAGGGAGAUCGACCGUGUUAUAUUCGCUUUAUUGACUUCGAAUAAUACAUGGUUGAUCUUUUCUCAGUAUCAAAUCUCACCUUGGAGGACCCGUUGGAGAUGAAGCCCUUUUGAGGGUAGGAGCAGGACGGG*

*>hsa-mir-3679*

*UUUGUUUGGUAUACUUCCUUCGUCCCCUUUCCUGUAAUUUUUUAGAGCACCUAGUUAGAGGCCCCACGUGGUGAGGAUAUGGCAGGGAAGGGGAGUUUCCCUCUAUUCCCUUCCCCCCAGUAAUCUUCAUCAUGCGGUGUCCCCAGUCCUGUGUAAGACCUUUGCAAACACAGCUUCAAGCCCCUCUGUUCAUCUUGAUU*

*>hsa-mir-1297*

*ACCUGUAAAAUGAUAUUUUGAGGAGAGCAGACAUUUCAGUGAGUCUAAUUUUAAAGUUAGGUGUUUAUCUCUAGGGUUGAUCUAUUAGAAUUACUUAUCUGAGCCAAAGUAAUUCAAGUAAUUCAGGUGUAGUGAAACACUUCUAAUUUUAAAUUCAGAAGCACUGAAGAGUGAGUUGGAUUAUUGGCUGUAGCACUUAC*

*>hsa-mir-2114*

*CUCACCCAGUCAGCAAUGGAUCAAACGUCCCAGCUUCUGGGGCCAAAGACAGGCCUCUGCCUCCAUGCUCCUAGUCCCUUCCUUGAAGCGGUCGGAUAAUCACAUGACGAGCCUCAAGCAAGGGACUUCAAGCUGGUGGCUUCCAGCAGCUAGCUUCCUGCUUCCGAACCAUCAUUGCCUCCUAUUGAAGUCCUCCCAUC*

*>hsa-mir-652*

*UUUUGGCUUUGUUCUCAAACACUAGGAAUGUUUCCAAGCAGGGCCGCCACGAAUGGCUAUGCACUGCACAACCCUAGGAGAGGGUGCCAUUCACAUAGACUAUAAUUGAAUGGCGCCACUAGGGUUGUGCAGUGCACAACCUACACAACUGUACUCGGCGGCCCUGGCAUAAUGUAAUGUCAGUACUUUCUUCAUCAAGG*

*>hsa-mir-361*

*AGCACAACAUAAGAGAUACAACAGUAGCAGUGGCACGCUUGACAGUGAUUUUUUUCCUGGGAUUUGGGAGCUUAUCAGAAUCUCCAGGGGUACUUUAUAAUUUCAAAAAGUCCCCCAGGUGUGAUUCUGAUUUGCUUCCUUUUCCUCCUCCUGCUCUGAGAAUCACCGUUUCUUUAGAAGCAUCAGCAUAAAUAGGCACA*

*>hsa-mir-762*

*AGCGCUCACCAUCUCCGGACCUUCUCGAUGGUCGCCAUCACCUUCUUGAUGUCAUCCUUGGCCCGGCUCCGGGUCUCGGCCCGUACAGUCCGGCCGGCCAUGCUGGCGGGGCUGGGGCCGGGGCCGAGCCCGCGGCGGGGCCGCCUCCCGUCCGGCGGGCUCAGGCUCCGCGCCAGGCCCGGGCGCCGCGCUCUCGGCCU*

*>hsa-mir-632*

*CACCCACUCAGGGCGGAGGUCUGCGUCAUGUGACCCUCCCCUUCUUGGCUCCGCCUCCUACCGCAGUGCUUGACGGGAGGCGGAGCGGGGAACGAGGCCGUCGGCCAUUUUGUGUCUGCUUCCUGUGGGACGUGGUGGUAGCCGUUGGGUUGGGAAAGUGAGGGAUUUUUGGCCUCGUUUCUCCUGCUUCUUUUCUCCUC*

*>hsa-mir-520d*

*UUUGACCAGGUCAAGGAAAAUUUCAACAAGAAACCCAGAGUGCCGGAGCAAGAAGAUCUCAAGCUGUGAGUCUACAAAGGGAAGCCCUUUCUGUUGUCUAAAAGAAAAGAAAGUGCUUCUCUUUGGUGGGUUACGGUUUGAGAAAAGCAACGUUGAAGUUGAUGCUGAUUUCGGUAAUACAUUUGCAGAGCAUGCUUAUC*

*>hsa-mir-182*

*CUGUCUCUUCCUCAGCACAGACCGAGGCCUCCCCAGCUCCUGGGGGGAGCUGCUUGCCUCCCCCCGUUUUUGGCAAUGGUAGAACUCACACUGGUGAGGUAACAGGAUCCGGUGGUUCUAGACUUGCCAACUAUGGGGCGAGGACUCAGCCGGCACCCUGUGCACAGCCAGCGAGGGAAGGGCCGGCCAUGCUGGACCUG*

*>hsa-mir-522*

*UUUAACCUGGUCAAGGAAAAUUCCAACAAAAAAUCCACGAUGCUGGAGCAAGAAGAUCUCAGGCUGUGUCCCUCUAGAGGGAAGCGCUUUCUGUUGUCUGAAAGAAAAGAAAAUGGUUCCCUUUAGAGUGUUACGCUUUGAGAAAAGCAUCGCUGAUCUUGGUAACACAUUUGCAGAGAAUGCUUAUAAUCAGACGUGGA*

*>hsa-mir-1203*

*CUUUUGGACUCAGAUAUUUCUGAACAGCUUUUUCUGGUGAGGGACUAAACUUCCCUUCCUCCCCGGAGCCAGGAUGCAGCUCAAGCCACAGCAGGGUGUUUAGCGCUCUUCAGUGGCUCCAGAUUGUGGCGCUGGUGCAGGUCUCCUCAUACGUAACCAAUGGCUCCUGAGAGCCUGCGACUCGUAAAUUACUUCCCACG*

*>hsa-mir-3144*

*UCCAGCUUCCAAAAAUGGUUAUUCUUCAGAAAGAAAAUCUACAGGAAAACAUCAGACUGAAACUACACUUUAAGGGGACCAAAGAGAUAUAUAGAUAUCAGCUACCUAUAUACCUGUUCGGUCUCUUUAAAGUGUAGUUUAACUGAUUUUUUAAUGGAUAUAUACAUAUCAAUGGAGACAUAUAUAACUUAUGGAUAUAG*

*>hsa-mir-1247*

*GCUGUUGCCUGAACGCCGGAGCGCUCCCCUCUCGCCGCUUGCCUCGCCCAGCGCAGCCCCGGCCGCUGGGCGCACCCGUCCCGUUCGUCCCCGGACGUUGCUCUCUACCCCGGGAACGUCGAGACUGGAGCGCCCGAACUGAGCCACCUUCGCGGACCCCGAGAGCGGCGGCGCGACUCACCGCGGAGGCGCCCGGACGC*

*>hsa-mir-519b*

*UUUAACCUGGUCAAGGAAAAUUUCUACAAAAAACCCAGGGUGCUGGAGCAAGAAGAUCCCAUGCUGUGACCCUCUAGAGGGAAGCGCUUUCUGUUGUCUGAAAGAAAAGAAAGUGCAUCCUUUUAGAGGUUUACUGUUUGAGGAAAGCAACAGUGAAGUUGAUGCUGAUCUUGGUAAUACAUUUGCAGAGCAUGCUUAUC*

*>hsa-mir-711*

*AUGUUCUUGCCAUGCAGGCCUUAGGCUCACAGGGACUAGAGCCCCUGACCCCAUGACCAUGAUGAACUGACUUUGAGUCUCUCCUCAGGGUGCUGCAGGCAAAGCUGGGGACCCAGGGAGAGACGUAAGUGAGGGGAGAUGCUGGGACAGAGGGGGCUCGGGGCUGCGUAAGCUCCAACCAGAUCAUCAUAGUCACAGCA*

*>hsa-mir-760*

*GCGUCCUUGGAGGCAGGUGCUGGCGGCAAGACGAGCUCUCGGAGGAUGCUGCGGCGCGGGGCGCGUCGCCCCCCUCAGUCCACCAGAGCCCGGAUACCUCAGAAAUUCGGCUCUGGGUCUGUGGGGAGCGAAAUGCAACCCAAACCCCGUUUUUCGAACCCCGCGCAAAUAAACACGCACAAGCACGCACACAGACGUCG*

*>hsa-mir-575*

*GGACACUAUCACUGUCAGAGUCCACCCGCGUGACUCUGGGGCGCACUGCUUUGUGCCUUUAAAUUCAGCCCUGCCACUGGCUUAUGUCAUGACCUUGGGCUACUCAGGCUGUCUGCACAAUGAGCCAGUUGGACAGGAGCAGUGCCACUCAACUCUGGCCAUACACAGAACCCCCCACCCCCACCCCAGGGAGCUUUUAA*

*>hsa-mir-603*

*UCAAGUAUGUUUUUUUUCUCAUUAAGCCUUUGCUUAAGAUGCAGGCAAGAUUGAUGCUGUUGGUUUGGUGCAAAAGUAAUUGCAGUGCUUCCCAUUUAAAAGUAAUGGCACACACUGCAAUUACUUUUGCUCCAACUUAAUACUUGGCUUUAUUGACUUUUAGCAUCAGUGCAGUUUGGUUAAUGAUAAAAUUUUCCAAA*

*>hsa-mir-18b*

*AGAACUGUAGUUUGUGCAUAAUUAAGUAGUUGAUGCUUUUGAGCUGCUUCUUAUAAUGUGUCUCUUGUGUUAAGGUGCAUCUAGUGCAGUUAGUGAAGCAGCUUAGAAUCUACUGCCCUAAAUGCCCCUUCUGGCACAGGCUGCCUAAUAUACAGCAUUUUAAAAGUAUGCCUUGAGUAGUAAUUUGAAUAGGACACAUU*

*>hsa-mir-452*

*AAUGCUUUGCAGUCAUCCCGUGCAAACAUUGUAAGACAUGGCUCCUGUCUUAUAAUUGCUAAGCACUUACAACUGUUUGCAGAGGAAACUGAGACUUUGUAACUAUGUCUCAGUCUCAUCUGCAAAGAAGUAAGUGCUUUGCCAAGCUCCUUGAGAGGUUAGGUAAGUAGAUAAAGUUCUGCUGCUGUCGGAAUGUGCAG*

*>hsa-mir-98*

*GUCUGUCACCAUGUAAAAUGGCCUUUUGCCUGCUGCCCUUAUUAGAGGAUUCUGCUCAUGCCAGGGUGAGGUAGUAAGUUGUAUUGUUGUGGGGUAGGGAUAUUAGGCCCCAAUUAGAAGAUAACUAUACAACUUACUACUUUCCCUGGUGUGUGGCAUAUUCACACUUAGUCUUAGCAGUGUUGCCUCCAUCAGACAAA*

*>hsa-mir-3151*

*ACUGAAUACAAAUGUCUUAGUAUGGAAUUCAAGGCUUUCUCUCCAGCCUCUCCAUACAACCUUUGGGGUGAUGGGUGGGGCAAUGGGAUCAGGUGCCUCAAAGGGCAUCCCACCUGAUCCCACAGCCCACCUGUCACCCCAAAAGCCUGCUGCUCCUGCCCUUCUGCUUCAGUUAUGUGUUCCCUGAUAGCACACACCAU*

*>hsa-mir-363*

*UUAUUUUAACUUUUAAAAGCCGUAAGUUCUGAUAUUUAGUCAUUGUAAAAUGAUCUGUUUUGCUGUUGUCGGGUGGAUCACGAUGCAAUUUUGAUGAGUAUCAUAGGAGAAAAAUUGCACGGUAUCCAUCUGUAAACCGCAGGACCUUUGUUGGCGACAUUCCUGAUCAGCGCUACAGUAAGCUAGAUGGUAAAAAAUCC*

*>hsa-mir-770*

*GUGGAUGGCAACAGCAUUUGGAAGGCGGAGGACAUGGAAUUCAUGUGUCAGGAGCCACCUUCCGAGCCUCCAGUACCACGUGUCAGGGCCACAUGAGCUGGGCCUCGUGGGCCUGAUGUGGUGCUGGGGCCUCAGGGGUCUGCUCUUCUUCUCUUUCAGAAUCUGGGGCUCCAGGCUAUGCCUUGGCUGGACUGAGGUCU*

*>hsa-mir-3654*

*GAGCACUUUGAUAAGGACGGCUGGUCCCUGUGGUACUCAGAGUAUCGCUUCCCUGAAGAACUCACUCAGACCUUCAUGAGCUGCAAUCUCAUCACUGGAAUGUUCCAGCGACUGGACAAGCUGAGGAAGAAUGCCUUCGCCAGUGUCAUCCUUUUUGGAACCAACAAUAGCAGCUCCAUUUCUGGAGUCUGGGUCUUCCG*

*>hsa-mir-519d*

*UUAACCUGGUCAAGGAAGAUUUCAACAAAAAACCCAGAGUGCUGGAGCAAGAAGAUCCCAUGCUGUGACCCUCCAAAGGGAAGCGCUUUCUGUUUGUUUUCUCUUAAACAAAGUGCCUCCCUUUAGAGUGUUACCGUUUGGGAAAAGCCACGUUGAAGAUGAUGCUGAUCUUGGUAAUACAUUUGCAGACCAUGCUUGUA*

*>hsa-mir-19a*

*UAUGUAUUCAUCCAAUAAUUCAAGCCAAGCAAGUAUAUAGGUGUUUUAAUAGUUUUUGUUUGCAGUCCUCUGUUAGUUUUGCAUAGUUGCACUACAAGAAGAAUGUAGUUGUGCAAAUCUAUGCAAAACUGAUGGUGGCCUGCUAUUUCCUUCAAAUGAAUGAUUUUUACUAAUUUUGUGUACUUUUAUUGUGUCGAUGU*

*>hsa-mir-1260b*

*CGCAAUCAGGGCUGAGUUCCUCUGGUCCCCUGGGGUUGAAGCGGGCGGCUGCUGCUCUCCGUUUAUCCCACCACUGCCACCAUUAUUGCUACUGUUCAGCAGGUGCUGCUGGUGGUGAUGGUGAUAGUCUGGUGGGGGCGGUGGGGCUGCUGUUGCUGCUGCUUGGGAGGCCGCAGGAGGGGCAGCAGGGGGCGGUGGAG*

*>hsa-mir-23b*

*GCGUCCUUCCGCGGAAGCCCAGUGUGUGCAGACAGCACGGGGUGGCGCUGCUCUCAGGUGCUCUGGCUGCUUGGGUUCCUGGCAUGCUGAUUUGUGACUUAAGAUUAAAAUCACAUUGCCAGGGAUUACCACGCAACCACGACCUUGGCUGCUCCUCCAGAAACCGUGGUCGCGCUCACUGCAGAUUGGAGAACAGGUGC*

*>hsa-mir-409*

*UCUUCUGCAAGCACAGCCGCCUGCAAGCAUUCACCUUAGUCCGAGCAUCUGAGCCUCUCCAUGGUACUCGGGGAGAGGUUACCCGAGCAACUUUGCAUCUGGACGACGAAUGUUGCUCGGUGAACCCCUUUUCGGUAUCAAAUUCCACCAGGGAGGCCGUCUUGGAGGCUGGGGCACCUCGGGGAAGGACGCCGGCAUCA*

*>hsa-mir-3190*

*CAAUUUCUCAGCUUCUCCUCCACAGGCACCAGAAAGCAAGAAGUACCUGGGGACGCCGCUCUGGGGUCACCUGUCUGGCCAGCUACGUCCCCACGGCCCUUGUCAGUGUGGAAGGUAGACGGCCAGAGAGGUGACCCCGGGGGUGGUCAGGCUUAACGACCUCUAUUUUCCAAAGGGGCCACAUUUUGCCUUUUCCAUGU*

*>hsa-mir-514b*

*AAAAGAAAGGUGCAUGAGUCAGUCAUUAGGCUGGGCUGCCAUCCUUAAGUGUUCUAUUCUUCAUGUGGUACUCUUCUCAAGAGGGAGGCAAUCAUGUGUAAUUAGAUAUGAUUGACACCUCUGUGAGUGGAGUAACACAUGAUAUAUACAUCAUAUGACAUGGCAUGGUGGAUACACGGGGCGUGUUAAGUUGCAAGCAG*

*>hsa-mir-887*

*AUGACAAGCAGGCCAGUGGCGUUGGAGGAAUGAGGUAAAAGAUGCCGUCAGAGAGCCCCGGGGUGCAGAUCCUUGGGAGCCCUGUUAGACUCUGGAUUUUACACUUGGAGUGAACGGGCGCCAUCCCGAGGCUUUGCACAGGGGCAAGCUUCGAGUUCCGUUUUCCAAGACACCUCGGGCAGAUAGGGGUGUGGGGAAUC*

*>hsa-mir-3165*

*UACUUGAGUGCCUUCCUGUGACUGGCCAGAGACACUUGUUUUGCAGAUUGUCAAAUUUUUGGUACCCGGUGGCAAGGUGGAUGCAAUGUGACCUCAACUCUUGGUCCUCUGAGGUCACAUUGUAUCCACCUUACCACUGGGUACUAACGUGUGGGCAGCAGCUGCAUUCUGCCUAGCGAGUGGGCAUUUGUUCAGUGCCU*

*>hsa-mir-1288*

*UCUGCUAUUGCCCCCAGACAAUGGAAGGGGGGCGACAGCUGUCCAUGGGGGCACUGCUAAGAGGGUGUUGAUCAGCAGAUCAGGACUGUAACUCACCAUAGUGGUGGACUGCCCUGAUCUGGAGACCACUGCCUUUCGAGAAUAAUUCACCCUACAGAAGCUAUCUGAGCAUCACCAGACAGGGUCUUCCUGGGAAUUAG*

*>hsa-mir-1294*

*GUAAUAAAUGUCACCAUUUAUUAACACCUAAUGUGUGCCAAGAUCUGUUCAUUUAUGAUCUCACCGAGUCCUGUGAGGUUGGCAUUGUUGUCUGGCAUUGUCUGAUAUACAACAGUGCCAACCUCACAGGACUCAGUGAGGUGAAACUGAGGAUUAGGAAGGUGUAAACUGGACAAGCGCAUGGCAGAGUCAGAAUCCAA*

*>hsa-mir-30a*

*UGCUGGGAUUACUUCUUCAGGUUAACCCAACAGAAGGCUAAAGAAGGUAUAUUGCUGUUGACAGUGAGCGACUGUAAACAUCCUCGACUGGAAGCUGUGAAGCCACAGAUGGGCUUUCAGUCGGAUGUUUGCAGCUGCCUACUGCCUCGGACUUCAAGGGGCUACUUUAGGAGCAAUUAUCUUGUUUACUAAAACUGAAU*

*>hsa-mir-569*

*CAUUCUAGGACACUCCAAAUUUAGAGUGUGUGAUUUGUGAACAAGGAGUUAGUAGGUAUUGUUAGAUUAAUUUUGUGGGACAUUAACAACAGCAUCAGAAGCAACAUCAGCUUUAGUUAAUGAAUCCUGGAAAGUUAAGUGACUUUAUUUCCUUGUCUGUGGAGACGUUUUCCAUUAGAAAAAUAUAUGGCUUUGUCAGU*

*>hsa-mir-1911*

*GCAUUCAUCAUCUCCCACUGGGCAUCCAUCAUCUGCCACUGGGUAUCCAUCAUCAUUUGCUCGGCAUCUGCUGAGUACCGCCAUGUCUGUUGGGCAUCCACAGUCUCCCACCAGGCAUUGUGGUCUCCGCUGACGCUUUGCCAUUUCGCCACCGGUGCCUUCCAUGAGAAUGCUGGUUACUUCUCCAGAGUGCUGUCACC*

*>hsa-mir-1291*

*GUUCUCAUUGUAGAAGGACCAGUAAAGUAAUGUGAUUUGAGUUCUGUCCGUGAGCCUUGGGUAGAAUUCCAGUGGCCCUGACUGAAGACCAGCAGUUGUACUGUGGCUGUUGGUUUCAAGCAGAGGCCUAAAGGACUGUCUUCCUGUGGUCUGUUGGCUGUUCUGGGACCUCAGUAGGGAAUGGCUAUUUCAUUUGGAAG*

*>hsa-mir-599*

*UUCGUGACUUCUAUUCAGAACUUUAAUGACUGGGAAUACGUUUAAUGCCAAAGACAUGCUGUCCACAGUGUGUUUGAUAAGCUGACAUGGGACAGGGAUUCUUUUCACUGUUGUGUCAGUUUAUCAAACCCAUACUUGGAUGACAGUCAAUUCAGUUAAAGGAUAAUGGGGUCCCAGGAUACAAAACAUUAUUUCCUUUA*

*>hsa-mir-3163*

*CGGACAGGAUGGAGUUCAGGCUUAGCCCUGAUCAGCAAGUUCCUUAGCUUCUUCAAGCCUUGGUUUCCUCAUCUAUAAAAUGAGGGCAGUAAGACCUUCCUUCCUUGUCUUACUACCCCCAUUUUAUAGAUGAGGAAACCAACCAGUGGGGGCUGUUGGGUAGAGAUCAAAUGCAAUAACUUUUGUACAAAUGAAAUAAC*

*>hsa-mir-3180-5*

*CAGUGCGACGGGCGGAGCUUCCAGACGCUCCGCCCCACGUCGCAUGCGCCCCGGGAACGCGUGGGGCGGAGCUUCCGGAGGCCCCGCCCUGCUGCCGACCCUGUGGAGCGGAGGGUGAAGCCUCCGGAUGCCAGUCCCUCAUCGCUGGCCCGGUCGCGCUGUGGCGAAGGGGGCGGAGCCUGCACCCGCCCCGCCCCCCC*

*>hsa-mir-548s*

*UCCUCAGCCAAAAAAGGAGGAGCUAGACCAGGUGGUCUCGGAGGAAUCUUUCUAUUAGCUUGCUGCAAAAAUAAUUGCAGUUUUUGCCAUUAUUUUUAAUAAUUAUAAUAAUGGCCAAAACUGCAGUUAUUUUUGCACCAACGUACUGUUUUGGGUUCCAUCUUUCCUGGUCAGGGACCUGCCCUCCCGAACCCAGAACU*

*>hsa-mir-299*

*CGCCCUCAGGCAACUUCGUGGACAUGAGCCUUCUUCCAGCCACCACCACCACCAUCGUGCGGUACUUGAAGAAAUGGUUUACCGUCCCACAUACAUUUUGAAUAUGUAUGUGGGAUGGUAAACCGCUUCUUGGUAUCCAGCCCAGCCAUCCAAACCUCCCUGGAGAAAGCCUGGAGUUAGAGGAGAGCCACGGGUCUCGG*

*>hsa-mir-523*

*UUUAACCUGGUCAAGGAAAAUUGCUACAAAAAACCCAGGGUGCUGGAGCAAGAAGAUCUCAUGCUGUGACCCUCUAGAGGGAAGCGCUUUCUGUUGUCUGAAAGAAAAGAACGCGCUUCCCUAUAGAGGGUUACCCUUUGAGAAAAGCAGCAUUGAAGUUGAUGCUGAUCUUGCUAAUACAUUUGCAGAGCAUGCUUAUC*

*>hsa-mir-3133*

*AUUUGUAGGCCUUUUUUGGAGAAAUGUCUAUUCAAAUCCUUUGCCCACCUUUUAUUUUAUUCAGAAAUUGUAAAGAACUCUUAAAACCCAAUAGUAAAAAGACAACCUGUUGAGUUUUAAGAGUUCUUUAUAUAUUCUGAAUGCUAGACCUUUAUCAGACUAUGAUUUGCAAACAUUUUCUCCCAUUGUGUGGGUUGUCU*

*>hsa-mir-518c*

*CUUAACCUGGUCAAGGAAAAUUUCAACAAAAAUCCCAGAUGGCUGGAGCGAGAAGAUCUCAUGCUGUGACUCUCUGGAGGGAAGCACUUUCUGUUGUCUGAAAGAAAACAAAGCGCUUCUCUUUAGAGUGUUACGGUUUGAGAAAAGCAACGUUGAAGUUGAUGCUGAUCUUGGUAAUACAUUCGCAGAGCAUGCUUAUC*

*>hsa-mir-1267*

*UCACUCAUCACCAAGAGGAUGGCCCAAGCUAUUCAUGAGGGAUCUGCCUCCGUGAUCCAAAUACCUCCCAAAUCUCCUGUUGAAGUGUAAUCCCCACCUCCAGCAUUGGGGAUUACAUUUCAACAUGAGAUUUGGAUGAGGACAAAUAUCCGGACUCCAUCAGUGGUCAAGAACUUUUUAUUUCUGGAGAGCUAUGUUAA*

*>hsa-mir-548k*

*AAUGUUGGAAGUGUCCAGGUUUAGGUAUAAAGAAUCAGAAGCUUUUCUCAAGUAUUGCUGUUAGGUUGGUGCAAAAGUACUUGCGGAUUUUGCUUUACUUUUAAUGGCAAAAACCGCAAUUAUUUUUGCUUCAACCUAAUAUGAUGCAAAAUUGGCUACAACUGUAAUUUGUUGAGUGCUGCAAACAAGGUACUUGCUAA*

*>hsa-mir-3188*

*UGCCGGCCUCCGGGGACUGGAGGGGGAUGGGGUCCCGCAUGGCUAAAUAACUCCUUGGCGCCUCCUGCUCUGCUGUGCCGCCAGGGCCUCCCCUAGCGCGCCUUCUGGAGAGGCUUUGUGCGGAUACGGGGCUGGAGGCCUGGUGGGGCUGUGUCCGGGGGCUCGCCGGGGGUGUGGGGAGGUCUGCGUUCCUCACCCUC*

*>hsa-mir-432*

*UAUCAGAUGCUCCACUGAGUGGAUCCCCCCUUGCCUGGUGUGAACCGUUGCAUGACUCCUCCAGGUCUUGGAGUAGGUCAUUGGGUGGAUCCUCUAUUUCCUUACGUGGGCCACUGGAUGGCUCCUCCAUGUCUUGGAGUAGAUCAGUGGGCAGCUCUUCCAUUUCCUGGAGUGGGCCACUGGGGGGCUCCUUCUUUUCC*

*>hsa-mir-3192*

*GAAUGUUGCUGAUUUCUAGUUAUUUCACUGUGGUGAAGGUCUUCAGCUCUAGGAAAGACAGAGGAAGGGAUUCUGGGAGGUUGUAGCAGUGGAAAAAGUUCUUUUCUUCCUCUGAUCGCCCUCUCAGCUCUUUCCUUCUGACUGAAAAUCAUCUUCAGCACCUCUCUUUUACCACCUCUGCCACCACCCACCCACUGCGA*

*>hsa-mir-340*

*CUCCUUUUCCAGCUUGAGUCUUCAAGAGGGAGCCUCAACUGAUUUCUAAAACCGUUUGUACCUGGUGUGAUUAUAAAGCAAUGAGACUGAUUGUCAUAUGUCGUUUGUGGGAUCCGUCUCAGUUACUUUAUAGCCAUACCUGGUAUCUUAUACCACAGAUCAUGGCCCGUUGAACAACAUUUUAAGAGAUUUCUAAAGGG*

*>hsa-mir-4291*

*UUGAUGGGCUCUGUGAUGGAUGGGUCUCCACUGUGGAGGUGCCACAGCUCAGGACGCCCUGAGGGUGACGCCGGGGGCUUCAGCAGGAACAGCUGGGUGGAGGCAGAGCUGUUCUGCUGUGGCUGCAGCCCUGAACUGUGGGAUUCAGAGCUGAGAGUGAGGUAAUGAAGCCAAGUGCUUCCAGGGUCUACAGAUGGAGG*

*>hsa-mir-3925*

*CUGCAAGUUUUCGCUGAGUCUUAUUUCCUGAACAUAAACUCAGGAUCCUGGACCAUGGUCAUGUGGGAAUAGCAAGAGAACUGAAAGUGGAGCCUGUCACAUCUCCAGACUCCAGUUUUAGUUCUCUUGCUAUUUCCACCACAUCUUUCAUCACUUCCUCCACUAAAGUCUUGAACUCCUCAAAGCCAUCCACGUGGGCU*

*>hsa-mir-99a*

*AAUUGCAUCAGAUAUUUACAACAAAUUCUAUAUUAAUAGGGGGCCCAUGCAAGAUGUUGCCCAUUGGCAUAAACCCGUAGAUCCGAUCUUGUGGUGAAGUGGACCGCACAAGCUCGCUUCUAUGGGUCUGUGUCAGUGUGGUAAUCUGACAAAAUGCUAUACACAGUGCCGUUCAACAAUAGUUCAGUAAAAUCCUGUUA*

*>hsa-mir-548g*

*AUCAUUGCCAGCAAUCUACACAACUUCAUUACCCCUUUAGAGAAACACUUUCUCUGAGUUAUUAGAUUAGUGCAAAAGUAAUUGCAGUUUUUGCAUUACGUUCUAUGGCAAAACUGUAAUUACUUUUGUACCAACAUAAUACUUCAUAUAUGUAUAUCUGUAUGUAUGGUUAUGUACAUGUGUGUAUAUAGAUUACAUGC*

*>hsa-mir-604*

*UCAAGCUCUCUUGGCUCAGUGGUCUGUUUCCAUGCACUGAGAGAGGUGUCUGCAGGAGAGCAUCGUGCUUGACCUUCCACGCUCUCGUGUCCACUAGCAGGCAGGUUUUCUGACACAGGCUGCGGAAUUCAGGACAGUGCAUCAUGGAGAAACAUACAUCUUGGGGGCCAUUCUAAAGAACAGACAUCUGGGUCCUCCUC*

*>hsa-mir-602*

*AAAACUCAACGCACGAGACCUGGAAGAGGAACGUGAAACACUCCAGAAAUCCCCCUUCUCACCCCCGCCUGACACGGGCGACAGCUGCGGCCCGCUGUGUUCACUCGGGCCGAGUGCGUCUCCUGUCAGGCAAGGGAGAGCAGAGCCCCCCUGGCUUUGGCAGGAGCGUCGGACGCAACGCGCGUCUGGACCAGGUGAAA*

*>hsa-mir-942*

*ACAUUUGGAGCAGGCCAUAAAGUGGGUAAAGCUCUUUCACUUUCUUCUUCAGCAAACAUUAGGAGAGUAUCUUCUCUGUUUUGGCCAUGUGUGUACUCACAGCCCCUCACACAUGGCCGAAACAGAGAAGUUACUUUCCUAAUAUUUGCCUCCUUGGAGUGUCUCAAGUCCUGGAAGCAAGAGAUAAUAAGCAAUUAAUA*

*>hsa-mir-23c*

*GCCAUAGAACAAUAGUAUAGCUUUUCCCAUUUUUUAAAUGGGCAUCAAGAACAUUGAGUGACUUUCCAGGUGUCACACAGUGAGUGGCAUAAUCAGAGUACAAUUUGAGUCAUGCCCAUACAUCACAUUGCCAGUGAUUACCCAAGGAAAGUGACGUACAUGUGAGGAGGAGGAACAUUAGAAAGGUUAAAACAAGCUCU*

*>hsa-mir-3658*

*UCUCCAAGAGACAGAGGAGGGGUCAGGAGGCACUGCUCAUCUGUACAUACUGUUUCCUAUGACAUUACUGUAUUUAAGAAAACACCAUGGAGAUGAAAUGCCUUUGAUUUUUUUUUUCUUUUUGUACUUUGGAACGACAAAAUGAAACAGAACUUGACCCUGAGCUUAAAUAACAAAACUGUGCCAACUACUACUGGUGA*

*>hsa-mir-92b*

*CCCCGGGUUCCCGGGUCGGGCCGGCUUCAGGCGGUGGGGAGCGGGAUCCCGGGCCCCGGGCGGGCGGGAGGGACGGGACGCGGUGCAGUGUUGUUUUUUCCCCCGCCAAUAUUGCACUCGUCCCGGCCUCCGGCCCCCCCGGCCCCCCGGCCUCCCCGCUACCCCUAGCGGGGCAGCCCCCGCCCUCCUGGCUCUCCAUC*

*>hsa-mir-628*

*ACUAUUAUUUUCAUAAAGGAGCAGCACCAGAAUAGUCCUUAAUAUCGGAGAUAGCUGUUGUGUCACUUCCUCAUGCUGACAUAUUUACUAGAGGGUAAAAUUAAUAACCUUCUAGUAAGAGUGGCAGUCGAAGGGAAGGGCUCAUCUGACUUCUGGAAACCUAUGUUAAAAUACAGUGCUUUGAACCUUGAUCAUGGGCU*

*>hsa-mir-1280*

*GAAGCACUGCACCUGGGAGGCUGCGCUCCCAAAGCUCAUCUUGGCAAGCCGUCUGUCCCACCGCUGCCACCCUCCCCUCUGCCUCAGUGUGCCAGGCAUCAGCACUCACUCACAGAGGCAGGCUGGAUGGCGGGUGGGACAACAGCUUCCGCAGCCCCUCCUGUUCCCACUUUGCAGGGAGAAAAGAACACCUUGAUGAC*

*>hsa-mir-3683*

*CCCUAUGACUUUGGAAGUAGUACCAUCUCACCCCUUGGAUAUUAUGAACAAUAACACAGAGGGUGUACACCCCCUGCGACAUUGGAAGUAGUAUCAUCUCUCCCUUGGAUGCUACGAACAAUAUCACAGAAGGUGUACACCCCCUGCGACACUGGGAGUAGUAUUAUCUCCCAUCUUGGGUAUCACACACAAUAUCGCAG*

*>hsa-mir-766*

*CAAAAGUCCCCAGAGUCUUCACACAAGCCGUGGUGUAUGAAGCUGCAUCCUCAGGACCUGGGCUUGGGUGGUAGGAGGAAUUGGUGCUGGUCUUUCAUUUUGGAUUUGACUCCAGCCCCACAGCCUCAGCCACCCCAGCCAAUUGUCAUAGGAGCUGGAUUGGAAGAAGCGGAAGAAAUCUGUUGCCUGAGUGGAGAAGA*

*>hsa-mir-1180*

*GGGGGGACCUUGGGAGCUGUGGUACAGAGGAGGCGCGGCCGCGAGUCCCGGCGGGGCGAAGCCCGCGGCUGCUGGACCCACCCGGCCGGGAAUAGUGCUCCUGGUUGUUUCCGGCUCGCGUGGGUGUGUCGGCGGCGGGGCCUGACACCCCCGGCCUCCUGGCCUCCCGCCUGGCCCAACUGGGGUCUGGGGCUGGGGAG*

*>hsa-mir-196b*

*GGCAGCGGCGGAGGCGGCGGCGGCGGCGGGCGAGGCAGCCCUUCGCGGGCAGCACCAGAACUGGUCGGUGAUUUAGGUAGUUUCCUGUUGUUGGGAUCCACCUUUCUCUCGACAGCACGACACUGCCUUCAUUACUUCAGUUGAAAUCGUCUCCAGGUACCUCUGCGCGCGGGGGUCGGGCCGGGCGGGGCAUCACGGCC*

*>hsa-mir-3929*

*GGGACAGAUGGCCUGAGAUUUCAUCAUGCUACUCAGAACAGCACACAAUUUAAAACGUAUGAGUUGGCCAGGUGCAGUGGCUCACACCAGUAAUCCCAGCACUUUGGGAGGCUGAUGUGAGUAGACCACUUGAGCCCGGGAGUUCAAGACCAGCCUGGGCAACAUGGCGAACUCCUAUCUUUACAAAAAAUACAAAAAUU*

*>hsa-mir-595*

*GUCAGAGUGCAGCACGGGCCACAUUCUCUCUGCCAUGUGCAGAGGGCUCCUGCACGGAAGCCUGCACGCAUUUAACACCAGCACGCUCAAUGUAGUCUUGUAAGGAACAGGUUGAAGUGUGCCGUGGUGUGUCUGGAGGAAGCGCCUGUUGCUGGUUUUUGUUGUUUUGUUAUGUAUUUUUUGCCAUGUUCCAUGCUGUU*

*>hsa-mir-802*

*GCCCAUUGCGUGAUUUGAAGCGUGUAUUCCCAGCCUGACUCUUGGUCACCACUCGUUCUGUUAUUUGCAGUCAGUAACAAAGAUUCAUCCUUGUGUCCAUCAUGCAACAAGGAGAAUCUUUGUCACUUAGUGUAAUUAAUAGCUGGACCUUGCAAAGGAAGGUGGGAGAAGGAAGCCGUUCAUGCAUAGACCUGGAUAAA*

*>hsa-mir-3146*

*UGGUGAAUAAUACCAAAAUUGCCUCUUAUUCAGUCAUGUCCACAGAGAUACACAUUUUCUUUGCUAAGUCCCUUCUUUCUAUCCUAGUAUAACUUGAAGAAUUCAAAUAGUCAUGCUAGGAUAGAAAGAAUGGGACUUGGCCAGGGAAGAAGAGUUGAUUUACUGCUAAUGCAUUAAUUGGGCAUUUUGUUUCUGUGUAU*

*>hsa-mir-9-2*

*ACUGCCAGAAAGGAUCAGGACCUGGAGUCUGGCAAGAGGAAGACAGAGGCCUGUGUGGGAAGCGAGUUGUUAUCUUUGGUUAUCUAGCUGUAUGAGUGUAUUGGUCUUCAUAAAGCUAGAUAACCGAAAGUAAAAACUCCUUCAAGAUCGCCGGGGAGCGUGUGAGAAUGAAAGACUACAGCCGAGAGACAGUAAAAACC*

*>hsa-mir-492*

*GUGACUAGUACCAGAAGCAGGGGCACAGGCCCUCCCACAACUACAGCCACUACUACAGGACCAUCGAGGACCUGCGGGACAAGAUUCUUGGUGCCACCAUUGAGAACGCCAGGAUUGUCCUGCAGAUCAACAAUGCUCAACUGGCUGCAGAUGACUUCUGAACCAAGUUUGGUUUGAGUGUUUGGGUGCUGGAUGAGGUG*

*>hsa-mir-4295*

*UUCUCCUCAGUCUUUAUUCCAUAAGAAAUAUGCUUCAAUGGCUUAUGCAUUUAGAAUUGACUUUGUGGAACAGUGCAAUGUUUUCCUUGCCUGUGGCAAGACCACUUCGGUUCAAGGCUAAGAAACUAGACUGUUCCUACAGAGACCCUCUGGGUGACUCAUCUCUUUUGAGAUCCUGAUUGUCCAUUUUUUGGUUGGUG*

*>hsa-mir-658*

*CUGGAAGGGUAGUCAUUGGUUCGCCGCUCUGUCUCGGCCUGAGCCCGCCCCCGCUCGGUUGCCGUGGUUGCGGGCCCUGCCCGCCCGCCAGCUCGCUGACAGCACGACUCAGGGCGGAGGGAAGUAGGUCCGUUGGUCGGUCGGGAACGAGGCUCAGGCGGCCAGGCCCGCGCGGAGCCGUUGCCAUGGCAGCCGCCGCC*

*>hsa-mir-490*

*CCAACGGUGGUGAAGAGGAUCUCCCUUCACUUUAUGGAGGCCUUGCUGGUUUGGAAAGUUCAUUGUUCGACACCAUGGAUCUCCAGGUGGGUCAAGUUUAGAGAUGCACCAACCUGGAGGACUCCAUGCUGUUGAGCUGUUCACAAGCAGCGGACACUUCCAACUCCAUCUUCCCAAGAACUAAAUGGGUGUCUUCUAUA*

*>hsa-mir-566*

*AGUUCGAGACCAGCCUGACCAACAUGGAGAAACCCCAUCUCUACUAAAAAUACAAAAAUUAGCUAGGCGUGGUGGCGGGCGCCUGUGAUCCCAACUACUCAGGAGGCUGGGGCAGCAGAAUCGCUUGAACCCGGGAGGCGAAGGUUGCAGUGAGCCAAGAUCGCGCCAUUGCACUCCAGCCUGGGUGACAGAGUGAGACU*

*>hsa-mir-3936*

*AGGCCCAAGCUUAGUCCAAUAUUAUAGCUUCAGCAUCAGAGUAGAGAAUGAUUCAGAGCAUCUGUCCAGUGUCUGCUGUAGAUCCCUCAAAUCCGUGUUUGGACGCUUCUGGUAAGGGGUGUAUGGCAGAUGCACCCGACAGAUGCACUUGGCAGCAAUAACUUAUGCAUACCUGAAGAAUGACCCUAUGGUCUAAGAAG*

*>hsa-mir-562*

*UGUGUACAGGUUUUGGUGUGGACAUUCAGUUAUUUUGGGUAUACACCUAGGAGUGAAAUUGCUAGGUCAUAUGGUCAGUCUACUUUUAGAGUAAUUGUGAAACUGUUUUUCAAAGUAGCUGUACCAUUUGCACUCCCUGUGGCAAUGUAUGAGGGUCUCAGUUGCUCCAUAUCCUAACACUUGUUAUCUGUAUUUGUGAU*

*>hsa-mir-1257*

*GGGGACGGGGGGAUGCUGGGCACCAGGGGGACCUGUGGGCCAGCUGGCCCUGGGCUUGUGCUUGGGGAGUGAAUGAUGGGUUCUGACCCCCAUGCACCCCUGUGGGCCCCUGGCAUCACUGGCCCCAUCCUUCACCCCUGCCAACCACGCUUGCCCUGUGCCUCUGAAGCUCCCCCACUAAGGGGGUGGAGUCUAAUUCC*

*>hsa-mir-3171*

*UACAUAUACUAAUUCUUGUUUUAUAGAUAGAUAAAUGGAUAUAUGUGUGUAAAUAUUUAUGCAUAUAUAUAGAGAUGUAUGGAAUCUGUAUAUAUCUAUAUAUAUGUGUAUAUAUAGAUUCCAUAAAUCUAUAUAUGUAUGUGUGUAUAUAUAUGUAAAUACAUAUAAAAUAUGUAUGGAAUAUCUCUCUCUAUAUAUAU*

*>hsa-mir-558*

*GUAGGGGAAUUAUUCUCUUUGAUUUCAUGUCGUGUGUGUGUGUGUGUGUGUGUGUGUGUGUGUGUGUGGUUAUUUUGGUAUAGUAGCUCUAGACUCUAUUAUAGUUUCCUGAGCUGCUGUACCAAAAUACCACAAACGGGCUGGCUUAAAACAACUGAAAUUUAUUCUCAAUAGUUGAGGAGGCUGUAAGUCUGAAAUCA*

*>hsa-mir-3677*

*GGAGCUGGGGUUUCUGGAGCCUCAGGGGCCCUGGUUUGCUGGAAGGGCAGGCAGGGCUUCAUGGAGGGCAUUAGGCAGUGGCCAGAGCCCUGCAGUGCUGGGCAUGGGCUUCUCGUGGGCUCUGGCCACGGCCCUGAGCUCCUCCCCUUACCUUCCCAGAGCGGAGGGUCACCUGCUGAGCUGGGCACGGGCUUCUCGUG*

*>hsa-mir-634*

*AGAAAUACUUUCCCCACACUUCUAAGAGAGAUGGCAGAAACACCACAAACCCACACCACUGCAUUUUGGCCAUCGAGGGUUGGGGCUUGGUGUCAUGCCCCAAGAUAACCAGCACCCCAACUUUGGACAGCAUGGAUUAGUCUUGGCCUGUUUUGAACUCUGCACAAACCGAACCUUGUAGUGUUGCUCCUGGCCUGGCU*

*>hsa-mir-520a*

*UUAAACCUGGUCAAGGAAAAUUCCAACAAUAAACCCAGAGUGCUGGAGCAAGAAGAUCUCAGGCUGUGACCCUCCAGAGGGAAGUACUUUCUGUUGUCUGAGAGAAAAGAAAGUGCUUCCCUUUGGACUGUUUCGGUUUGAGUAAAGCAGCGUUGAAGUUGAUGCUGAUCUUGGUAAUACAUUUGCAGAGCACGCUCAUC*

*>hsa-mir-573*

*UUUAAUAACCUUAGAGAAACAUGUAGGAUUUUCUACAAAAAGUGUCGGCUGCUUUAGCGGUUUCUCCCUGAAGUGAUGUGUAACUGAUCAGGAUCUACUCAUGUCGUCUUUGGUAAAGUUAUGUCGCUUGUCAGGGUGAGGAGAGUUUUUGAGAUGCAAAUUUUCCCGUUUUUGUUUUAUUACCAUGGAGAAAUGUAAUA*

*>hsa-mir-3141*

*GCCUUGGGAACUUUUAAAGUAUGUCAGGACCCACAAGAGGAGGAUCAACUUUAAACAGGGAUGUAGAGGGACUCACCCGGUGAGGGCGGGUGGAGGAGGAGGGUCCCCACCAUCAGCCUUCACUGGGACGGGAGAUGCUGAGUGGGCUCAGGCGGCACUUAUCUGCCCCUCCCUGGGGGCCUGAGUCAGUCGGGACUUCG*

*>hsa-mir-132*

*CCCCCGCGGCCCUGACGUCAGCCCGCCCCGCGCCGGCGCCGUCCGCGCGCCCCGCCCCCGCGUCUCCAGGGCAACCGUGGCUUUCGAUUGUUACUGUGGGAACUGGAGGUAACAGUCUACAGCCAUGGUCGCCCCGCAGCACGCCCACGCGCCGCGCCACGCCGCGCCCCGAGCCGCGGGCUGGGGACGCGGAGCAGAAG*

*>hsa-mir-606*

*GCUUUGUUCUGUGUUUUUUUUUUUUCCCCCCCCUCUGGCUGCUUUCAGAAUUUUCACUGUAUCCUUGGUUUUUAGUAGUUUUACUAUGAUGAGGUGUGCCAUCCACCCCAUCAUAGUAAACUACUGAAAAUCAAAGAUACAAGUGCCUGACCACUUCUACCCCUCAUCCCCUACUUGGGAUUUUCUGAGGUUCUUUGAAC*

*>hsa-mir-513c*

*UUGAAUAAAAACGGCAUGAAUAGGGAACAUUUGGUCUGGGAUGCCACAUUCAGCCAUUCAGCGUACAGUGCCUUUCUCAAGGAGGUGUCGUUUAUGUGAACUAAAAUAUAAAUUUCACCUUUCUGAGAAGAGUAAUGUACAGCAUGCACUGCAUAUGUGGUGUCCCUAGGGAUGGGGCUGUAGCUGCGAGCAUGGUGAUG*

*>hsa-mir-324*

*UACUGCUGGCAGGCACCUGAGCAGAACAUCAUUGCUGUCUCUCUUCGCAGAAGCUGAGCUGACUAUGCCUCCCCGCAUCCCCUAGGGCAUUGGUGUAAAGCUGGAGACCCACUGCCCCAGGUGCUGCUGGGGGUUGUAGUCUGACCCGACUGGGAAGAAAGCCCCAGGGCUCCAGGGAGAGGGGCUUGGGAGGCCCUCAC*

*>hsa-mir-1276*

*CUUGGGCCUCUGCGUUGGUCUCGAGGGUGAGUCACUGGCGGCCCCACCUCCAAGCCAUCACUUGCCCCAGCUAGGUAAAGAGCCCUGUGGAGACACCUGGAUUCAGAGAACAUGUCUCCACUGAGCACUUGGGCCUUGAUGGCGGCUCUUGUUCCAAGGCAAGGGGGAUGUCUAGCCAGUACCGCUUGGAGCCCGGCCCA*

*>hsa-mir-548e*

*UUUCCUUUUAAAAGUUUUUUCUAAUUUACCCUUUUGUUUCUUCUGUGAUCUAUGGCUUUAUUAGGUUGGUACAAAAGCAAUCGCGGUUUUUGCUAUUACUUUUAAAGGCAAAAACUGAGACUACUUUUGCACCAACCUGAUAGAAGAAUGUUAUUUUGUUCCCAAAAGCUUGACAAUUUUCCAUGAUAUAUUAUUGACUU*

*>hsa-mir-590*

*UUUGGCUGAACAGGUGACGACUACCUGUUGAAUAAUAUAGUUCUUAAAAGUCCUGUAGCCAGUCAGAAAUGAGCUUAUUCAUAAAAGUGCAGUAUGGUGAAGUCAAUCUGUAAUUUUAUGUAUAAGCUAGUCUCUGAUUGAAACAUGCAGCAGCUGUCUUCUUAGAGCCAACCAGCAGCUCAGGGAACGGCCUUUCCCCU*

*>hsa-mir-1272*

*GUCCUGGUUCUCAGUGGGGUCUCUUUCCCUUGGGGAAGCCAGAUCAGAUCUGGGUGCGAUGAUGAUGGCAGCAAAUUCUGAAAACGUGCUCAGUGUCUUUAUAACAGGAAAGCCGUAAACUUAGAAAUGUAGGCUGCAGCUCGUGUGCUCUGUGGUCUGGGCUGGUAGGGAUGGCUGGGCCUGCUCCAGCACCCAUCCCC*

*>hsa-mir-3692*

*GGAGACACACUCAGACUUCCAGUUUUACUCAGCACCUACCACCUGUACUCUUCUGAGGUUCUUUGCCAUUCCUGCUGGUCAGGAGUGGAUACUGGAGCAAUAGAUACAGUUCCACACUGACACUGCAGAAGUGGAGAUCUCUGGAGUCUCUGCAACCCCACCCUCUCUUCACAGGGAGCAAGUUACUUCUUGAGUAUAAC*

*>hsa-mir-1323*

*ACAGCGAACCCUAGUGUGAGUUAACCACGUAGGAAGAGUUUGAAGUCAGACAUGACAUUCAGACUGAGGUCCUCAAAACUGAGGGGCAUUUUCUGUGGUUUGAAAGGAAAGUGCACCCAGUUUUGGGGAUGUCAAUUGUGAAUCCUCAUCAUAACCCAUCCCCAGUCCCCCAUCGCAUUGCACCACAUGGAUGCACCCAG*

*>hsa-mir-585*

*GAGAAGCCAGCACGAGAUGGAGCAGCUGGGGGAGGCUGCAGGGGGAGUGUGGGGUGUCUGUGCUAUGGCAGCCCUAGCACACAGAUACGCCCAGAGAAAGCCUGAACGUUGGGCGUAUCUGUAUGCUAGGGCUGCUGUAACAAUGUACCACAAACUGGGUGCCUUACAACAGCAAUCAUCCUUUCAGACUUCUGGAGCCU*

*>hsa-mir-504*

*UCCAUUUCCCACAUAGAUUCACAACAGAAGACAGAAAAUAUGGAGUGGAAUGUUGAAUCAGCUGCUGUUGGGAGACCCUGGUCUGCACUCUAUCUGUAUUCUUACUGAAGGGAGUGCAGGGCAGGGUUUCCCAUACAGAGGGCACAGACUACCACCGUAUGGCAAAUGUUGACUGGAACUCAACUUCAAAGCCAUUUCAC*

*>hsa-mir-1914*

*GUUCCCCCGGGGAGAGGCUGGGGACAAGCUACCCAUUCUGGGGUAUGACACCUAUCAGCACGUGUGAGCCCGCCCUGUGCCCGGCCCACUUCUGCUUCCUCUUAGCGCAGGAGGGGUCCCGCACUGGGAGGGGCCCUCACAGCCUACCCCGCUAGCUUUCUGCCUGAUGGUGAGCUUGCCCCGUUGUUCGCCGCCAUCUU*

*>hsa-mir-3127*

*ACAAGAUUCUGUCUCAAAAAUAACAAAAAAGAAAAUAACCACUGAGGUGGGCCCCUAGGCUGGCCAGGCCCAUCAGGGCUUGUGGAAUGGGAAGGAGAAGGGACGCUUCCCCUUCUGCAGGCCUGCUGGGUGUGGCUGGACCCCUCGUGUGCUCACCCACUCUGAGGAGCUUGCAUCCAGCUCUGCUCUUACUCUCCAGC*

*>hsa-mir-302b*

*UUUGUUUUCUUUCUCCUCAGCUCUAAAUACUCUGAAGUCCAAAGAAGUUGUAUGUUGGGUGGGCUCCCUUCAACUUUAACAUGGAAGUGCUUUCUGUGACUUUAAAAGUAAGUGCUUCCAUGUUUUAGUAGGAGUGAAUCCAAUUUACUUCUCCAAAAUAGAACACGCUAACCUCAUUUGAAGGGAUCCCCUUUGCUUUA*

*>hsa-mir-331*

*AUGGGUGAAAAGCCCAGAAGUCUACCUGAGCUGAAAGCACUCCCAAGGAGUUUGGUUUUGUUUGGGUUUGUUCUAGGUAUGGUCCCAGGGAUCCCAGAUCAAACCAGGCCCCUGGGCCUAUCCUAGAACCAACCUAAGCUCGCGCAUCAUUCCUGGAACAUCAAGAGUGUGAAGACUGAAGAUAACCUGAGGCCUACCAG*

*>hsa-mir-210*

*CCAAGUUGGAGGGGACGGGGGUGGGGUCAAUCCCUCCGCCCGCCCACCCGGCAGUGCCUCCAGGCGCAGGGCAGCCCCUGCCCACCGCACACUGCGCUGCCCCAGACCCACUGUGCGUGUGACAGCGGCUGAUCUGUGCCUGGGCAGCGCGACCCUCCUGGCCCCGUGUCCGGGUCUGGCGGGCAGGCGAGCGGCCACAC*

*>hsa-mir-1468*

*GCCUAAGCAAAGGUACCAGCCUGUGUUGUGUUCCCCAGAGUUGGGGGAAUCAUAAAGAGGUGGGUGGUUUCUCCGUUUGCCUGUUUCGCUGAUGUGCAUUCAACUCAUUCUCAGCAAAAUAAGCAAAUGGAAAAUUCGUCCAUCAACAAGCUACCUUUCAUGUUCCUAAGGGCAUGAAGGAGAAGCCUGGAGUGGUGUGU*

*>hsa-mir-3181*

*CGGCCGGCGGGCGGCCGGAUCCCCUCGGCGCAGCCGCCUGGCCUCAGGGCGUCCGGAGCCGCCGCGGCGACCAUCGGGCCCUCGGCGCCGGCCCGUUAGUUGCCCGGGCCCGAGCCGGCCGGGCCCGCGGGUUGCCGAGCCCGCUGACGUCAGCCCGGGUUUCCCCCCCCCACCGGGGCUUCCCCAUCCCCCGAGGCUUC*

*>hsa-mir-874*

*GAACCUGCUUGGACAAGUCUUCUGGCUCGACCUCGACAUGCUCCAUCGGAUGAAUUGUUGGUGUUAGCCCUGCGGCCCCACGCACCAGGGUAAGAGAGACUCUCGCUUCCUGCCCUGGCCCGAGGGACCGACUGGCUGGGCCUGCCUUCUGCCCAGCUCACCGGUCACAGAAGAGAGUUUGGACAUGCAUUCUACUCUUG*

*>hsa-mir-142*

*UGGAGCAGGAGUCAGGAGGCCUGGGCAGCCUGAAGAGUACACGCCGACGGACAGACAGACAGUGCAGUCACCCAUAAAGUAGAAAGCACUACUAACAGCACUGGAGGGUGUAGUGUUUCCUACUUUAUGGAUGAGUGUACUGUGGGCUUCGGAGAUCACGCCACUGCUGCCGCCCGCUGCCCGCCACCAUCUUCCUCGGC*

*>hsa-mir-1299*

*GAAACAUUCAGACCACAGCAGGAAUGUUCUGGAGUCCUAAGUGAGGAGCAAACAUUAAGACCCUCAUGGCAGUGUUCUGGAAUCCUACGUGAGGGACAAUCAUUCAGACCCACGUAGCAGUGUUCUGGAAUUCUGUGUGAGGGACAAACAUUCAGACCCUCGUAGCAGUGUCCUGGAAUCCUGUUUGACAGACAAUCAUU*

*>hsa-mir-449c*

*UCGUUUGCAUCUGAAUCGUACUUUCUAGUUUCUGCCUCCACAGCAAAGGAUAAAGCUGGGAUGUGUCAGGUAGGCAGUGUAUUGCUAGCGGCUGUUAAUGAUUUUAACAGUUGCUAGUUGCACUCCUCUCUGUUGCAUUCAGAAGCACGCCCCCCAAAGAAAUGGAAAUGGUGCCAUCUUGUGGACAAUGUGGAGAAGUG*

*>hsa-mir-1271*

*GGGAGGGAGCUCCUUGGUGCAGGGCCCCUGCUUGCCUUGCUCACCUCUGUAUCUGCAGCACCCAGAUCAGUGCUUGGCACCUAGCAAGCACUCAGUAAAUAUUUGUUGAGUGCCUGCUAUGUGCCAGGCAUUGUGCUGAGGGCUUUGUGGGGAUUCAGAGGUAAGUGCCUCUGACUCUCACAGCUUAGAAUCCUCAAGUG*

*>hsa-mir-3685*

*UGAGAAUAUUCUUUCAGAGGUAUUUUCAUUUUUACUUUUUGAUUAAUUGUGUUUUAUAUAUUAGGGUAGUACAUUUCCUACCCUACCUGAAGACUUGAGAUUAUAGUCUUUGGGGGGAUGGGCAAAGUACUCUCUUUUUUUGCAUCUUUUAAAUUGUGAUUUUUAAAAACACAUAUAAAUUUUACUAUCUUAACCAUUUU*

*>hsa-mir-126*

*CCCCGUCCCGGGGUCCUGUCUGCAUCCAGCGCAGCAUUCUGGAAGACGCCACGCCUCCGCUGGCGACGGGACAUUAUUACUUUUGGUACGCGCUGUGACACUUCAAACUCGUACCGUGAGUAAUAAUGCGCCGUCCACGGCACCGCAUCGAAAACGCCGCUGAGACCUCAGCCUUGACCUCCCUCAGCGUGGCCGGGACC*

*>hsa-mir-3687*

*AGGCGUCCGUCCCGGGCGUCGGCGUCGGGGAGAGCCCGUCCUCCCCGCGUGGCGUCGCCCCGUUCGGCGCGCGCGUGCGCCCGAGCGCGGCCCGGUGGUCCCUCCCGGACAGGCGUUCGUGCGACGUGUGGCGUGGGUCGACCUCCGCCUUGCCGGUCGCUCGCCCUUUCCCCGGGUCGGGGGGUGGGGCCCGGGCCGGG*

*>hsa-mir-572*

*GGCUCCGAGAAGAAAGAGUGUGGCCCGGGGCGGGCGCAUUAGGAGGUGUCGAGGCCGUGGCCCGGAAGUGGUCGGGGCCGCUGCGGGCGGAAGGGCGCCUGUGCUUCGUCCGCUCGGCGGUGGCCCAGCCAGGCCCGCGGGACUCAGACCAGCGGGGAGCGCGACCUCCGCCCUUGGGGCUCUCCCGCUGGGCCGGAGAC*

*>hsa-mir-1298*

*UCCCCUUUAUAUAGGAUGUCUUGAAGCCGAGAAGGAAGAGCUGUUGGAGAGACGAGGAGUUAAGAGUUCAUUCGGCUGUCCAGAUGUAUCCAAGUACCCUGUGUUAUUUGGCAAUAAAUACAUCUGGGCAACUGACUGAACUUUUCACUUUUCAUGACUCAGCUGGAACCCUCCACAACCAGAAGAGCCUGUUAUAAAGU*

*>hsa-mir-1237*

*AGCGCCAGAGCGGCCAGGAGUUCGCAGUCAAGAUCCUCAGUCGCAGGUGGGAGGGCCCAGGCGCGGGCAGGGGUGGGGGUGGCAGAGCGCUGUCCCGGGGGCGGGGCCGAAGCGCGGCGACCGUAACUCCUUCUGCUCCGUCCCCCAGGCUGGAGGCGAACACGCAGCGCGAAGUGGCUGCCCUGCGCCUGUGCCAGUCA*

*>hsa-mir-3134*

*UAGAUAAGUUCUUUAUUGAUGAUUUCUGAGAUUUUGGUGCACUGGUCCCCUGAGCAAUGUACACUGUAUCCAAUGUGUAGUCUUUUAUCCCUCACAUGGAGUAAAAUAUGAUGGAUAAAAGACUACAUAUUGGGUACAAUUUGGGAACUUCCCCCUGAGUCCCCAAAGUCCAUUCUAUCAUUUUUGUGCCUUCGCGUCUC*

*>hsa-mir-4277*

*UCCUGCCAGUGCUUAGCAGGGAGGAGAUGGGGCUGUGACUGCCCGGUCACUUGUCUGGCUCUGGGUCGAGGCAGUUCUGAGCACAGUACACUGGGCUGCCCCCACUGCCCAGUGCCCUGCUCAGCUCAAGUCCUUGUGCCCCUCCAUGUCUAGACAAAGCUGCCCCUGGGCGGGCUCCAAGUGGGGGCAGGGGCCGCCUG*

*>hsa-mir-660*

*UUCUGUCUUUCUUUCUCUCUCCUCCCCCACCCCCCUCCCAUGUGCAGAAGAAUGAACUGCUCCUUCUCCCAUACCCAUUGCAUAUCGGAGUUGUGAAUUCUCAAAACACCUCCUGUGUGCAUGGAUUACAGGAGGGUGAGCCUUGUCAUCGUGAAUCUUCUGCUGAACAAUCCAAGUAGCCCGUGUUUAUUGCGCACUUU*

*>hsa-mir-204*

*GGGUGGAGGCAAGCAGAGGACUUCCUGAUCGCGUACCCAUGGCUACAGUCUUUCUUCAUGUGACUCGUGGACUUCCCUUUGUCAUCCUAUGCCUGAGAAUAUAUGAAGGAGGCUGGGAAGGCAAAGGGACGUUCAAUUGUCAUCACUGGCAUCUUUUUUGAUCAUUGCACCAUCAUCAAAUGCAUUGGGAUAACCAUGAC*

*>hsa-mir-759*

*UAUUUUUCAGUGCAAAAGAAUGACUUUCAAAGUAUAUAUUAUGGAAAGGAUUAUAAUAAAUUAAAUGCCUAAACUGGCAGAGUGCAAACAAUUUUGACUCAGAUCUAAAUGUUUGCACUGGCUGUUUAAACAUUUAAUUUGUUAGAAUGGAAGUAGCGGCACAGAAUAAGCAUGUUAAUUAAACUUGGGCUUUAGCAAUG*

*>hsa-mir-300*

*UCCUGGAGCUGGUGGCAACUUAGUCACAGAGGAAAUGGCCUUCCUGUCACUGCCAUCAUUUGCUACUUGAAGAGAGGUAAUCCUUCACGCAUUUGCUUUACUUGCAAUGAUUAUACAAGGGCAGACUCUCUCUGGGGAGCAAAUCCUGCCUGGGAAGCCUUCUUGGGAACAAGGCUGACUCCAGCAACCUGUUAGAUGGC*

*>hsa-mir-1231*

*ACUCUGCCCACCAACUGGAUCAGCUUCGGGAGACCAUGCACAACAUGCAGGUCAGUGUCUGGGCGGACAGCUGCAGGAAAGGGAAGACCAAGGCUUGCUGUCUGUCCAGUCUGCCACCCUACCCUGUCUGUUCUUGCCACAGUUGGAGGUGGACCUGCUGAAAGCAGAGAAUGACCGACUGAAGGUAGCCCCAGGCCCCU*

*>hsa-mir-548z*

*AAUCAGAAUAAAAGAGAUUAAGCAACAAUUUUCUACUUAGGCUUCAACAAGCCUGAAAGUAUUAAGUUGGUGCAAAAGUAAUUGAGAUUUUUGCUACUGAAAGUAAUGGCAAAAACCGCAAUUACUUUUGCACCAACCUAAUAGAUGCCAAUGCCGAUUCCUUUCUCAUCGCAAUUUUUCAACAAAAGACAGUAAGCCUA*

*>hsa-mir-27b*

*CUGAGGAAGAUGCUCACCGGUCACCGUCCCUUUAUUUAUGCCCAGCGAUGACCUCUCUAACAAGGUGCAGAGCUUAGCUGAUUGGUGAACAGUGAUUGGUUUCCGCUUUGUUCACAGUGGCUAAGUUCUGCACCUGAAGAGAAGGUGAGAUGGGGACAGUUAAGUUGGAGCCGCUGGGGCAGAGGCCGUUGCUGACGGGC*

*>hsa-mir-888*

*AUGGAAGCUACAGGUGGAGAACACGCAAGAUGCGACAGCACCUCCACAAUUAGCCAUGUUGUGGGCAGUGCUCUACUCAAAAAGCUGUCAGUCACUUAGAUUACAUGUGACUGACACCUCUUUGGGUGAAGGAAGGCUCACCAAGUACACUUUUGUGGUGGUCCUCAGACUGGUGAGGGGGUAGACCUGCAAGCAACUUG*

*>hsa-mir-877*

*UUCAGGGACAGCUGGAACAAGGGGAUGACACAGCUGCUGAGAGGCUAGAGAAGGUAGAGGAGAUGGCGCAGGGGACACGGGCAAAGACUUGGGGGUUCCUGGGACCCUCAGACGUGUGUCCUCUUCUCCCUCCUCCCAGGUGUAUGAGGAAUUGCGGGCCACUGGGGCGGCAGCUGCAGAGGCCAAAGCACGGCGGAUCC*

*>hsa-mir-3167*

*GCUGCUGCUCCAUUCAGGAGCCACCUCCAGGCCUAUGCCCACCUGUGCCCUCGACACGGCUGUGGAGGCACCAGUAUUUCUGAAAUUCUUUUUUCUGAAAUUCUUCAGGAAGGAUUUCAGAAAUACUGGUGUCCCGACAGCCGUGUGACCAUCAGCAAGCCACAGAAACCCCUGCCCUGGCCGGUGGGUCCCCUCCUUCA*

*>hsa-mir-3908*

*UCCAGUGGGAGGAUCCCUUGAGUUCAAGAGUUCUAGUCUAGCCUGAGCAAUGUAGGUAGACUGUUUCUAAAAAAAUAAAAAGUUAAAAAAAUUUAUGUUAACGUGUAAUGUGUUUACUAAUUUUUUUUUUUUUUUUUGGAGACAGAGUCUCCCUCUGUCGCCAGGCUAGAGUGCAGUGGCUCGAUCUCAGCUCGCUGCAG*

*>hsa-mir-4285*

*CCCCGCCCCCAGGGCCACCGCCCACUGUGCCGCGCAUCGAUUGGUCGCGGGCCCAUUAGCUGGGGCGGCGAGUCCGACUCAUCAAUAUUUUAAGGAAUGACCCGGCCUUGGGGUGCGGAAUUGCUGCGCGGGCGGGGGCUGAAGACCGGCUUUUUUUUUUUUUUUUUUUUUUGGUCCUCCAAUUGUCAGCGCUAUUUUUA*

*>hsa-mir-4308*

*CAGAGAGCUGGAGUUGUUGGUGCUGAUCACCAGAUACUCCAGACGGCACUAAUUCCUAUACUAUGGGUUCAGAGGGAACUCCAUUGGACAGAAAUUUCCUUUUGAGGAAAUCUUUCCCUGGAGUUUCUUCUUACCUUUUUCCCUCACUUUCUCCACAGCAGAAACAGGUCUGUUUGUCCUUAAAUAUUUACCUAUAUAUU*

*>hsa-mir-758*

*CUGCUCUGGCUUGGCAGCAGGGUUAACUUGCGGACAAGGAGCGUGGUGUCAGCACGUGCCUGGAUACAUGAGAUGGUUGACCAGAGAGCACACGCUUUAUUUGUGCCGUUUGUGACCUGGUCCACUAACCCUCAGUAUCUAAUGCCCCCUUGGAGAACUUCCUGGAAGAGGUGUCAACUUUUUGGGAAAGAGACAGUAAA*

*>hsa-mir-508*

*AAAUUCUUCAUCGUAUCUAAACUUCACGUAUAUAUUUGGGUAGAGCCACCACCUUCAGCUGAGUGUAGUGCCCUACUCCAGAGGGCGUCACUCAUGUAAACUAAAACAUGAUUGUAGCCUUUUGGAGUAGAGUAAUACACAUCACGUAACGCAUAUUUGGUGGCCAUGGGUGUGAGUGUAUACAGCCGCAUGCACAGAGC*

*>hsa-mir-567*

*UAGGUGGAAGGAGGAGGUUAGGAUCACCUCAACUUUUUGUGAUGAAUCUAUGGAAGAGGAUUCUUAUAGGACAGUAUGUUCUUCCAGGACAGAACAUUCUUUGCUAUUUUGUACUGGAAGAACAUGCAAAACUAAAAAAAAAAAAAGUUAUUGCUUAAAUUCAUUUCUUGAGUUGCAGUAUGAUGCUGUUUAUGUUUUAA*

*>hsa-mir-548q*

*UUUUGAGUUUAAUGGAAAGAAGCUACAAAUACUUUCACACGUAAGAAUAUAUUAGGCUGGUGCAAAAGUAAUGGCGGUUUUUGCCAUUACUUUUCAUUUUUACCAUUAAAAGUAAUGGCAAAAAGCAUGAUUACUUUUUCACCAACCUAAGAUUCAUGCAUUCCAAGGGUGCCGAUUUCCAUGGCUGACAAAGAGCCCCU*

*>hsa-mir-376c*

*GUCUUUAAUUUUGAUAGAUUGUGCUUAGGUUCAUGCUUUCCAGGACUCAAUCCUUCUUUGGUAUUUAAAAGGUGGAUAUUCCUUCUAUGUUUAUGUUAUUUAUGGUUAAACAUAGAGGAAAUUCCACGUUUUCAGUAUCAAAUGCUGCUUGGAAACAUUCCUGGACUGAAACCACCAUCAGGGAAGAGACAAUAAAGUCC*

*>hsa-mir-1178*

*CUACCUGCAAGCCAGGGCCAGACUCUCUGGGCCCGGCUGGGGUAGAGAAGCCCAGGCGUUGGCUGGCAGAGGAAGGGAAGGGUCCAGGGUCAGCUGAGCAUGCCCUCAGGUUGCUCACUGUUCUUCCCUAGAAUGUCAGGUGAUGUGUCACCCCAAGUGCUCCACGUGCUUGCCAGCCACCUGCGGCUUGCCUGCUGAAU*

*>hsa-mir-2117*

*AUCUCAUCAACCACAUGUUUAUAGAGAGAGCAGUGGCCCUUAAGGAACUGGAGAGAAUGCUCUGAUUUACUUCUGUCCGGCAUGGUGAACAGCAGGAUUGGCUGUAGCUGUUCUCUUUGCCAAGGACAGAUCUGAUCUGAUUGCCUUGAGGUGAAAGUGAGGUAAGGUGGGGCGGGGAUUAGCUUUUGGCUCCCAAGAAU*

*>hsa-mir-3664*

*CAUGCGUGUGGGGUUUUUUUCCCCUCCUUUCCACUGUGCACCCCGUCUCCUCUGUAAACUUGAAGGUAGGGAACUCUGUCUUCACUCAUGAGUACCUUCCAACACGAGCUCUCAGGAGUAAAGACAGAGUUCCCUACCUUCAAUGUGGAUCUGCAAAAAGAUCUGAAAAGCGAAUGCCUUCUCCGUAGCUCGUUGGCAGU*

*>hsa-mir-605*

*AUUCUUUAAAGUAGGAACUUUGGUAGAACUUUCACAGCCUGUAACAUAUGUCUCUAGCCCUAGCUUGGUUCUAAAUCCCAUGGUGCCUUCUCCUUGGGAAAAACAGAGAAGGCACUAUGAGAUUUAGAAUCAAGUUAGGACUGCAGAUACAGGUUACCUAUGUUACAGGCUGACAGCCACAGGUAUAUUGCUCUCUGCAC*

*>hsa-mir-561*

*CAGCUUUUUCUUAAUAUUAGGUGUUACAGUUGAUCCAUUUCUUGAAACUUCAUCCACCAGUCCUCCAGGAACAUCAAGGAUCUUAAACUUUGCCAGAGCUACAAAGGCAAAGUUUAAGAUCCUUGAAGUUCCUGGGGGAACCAUGGAUGGCUAUCCUUAUUUCUAUAAGAGAGGAGACUGAAUACUGUGGGCUCAGUCUU*

*>hsa-mir-4316*

*UAAUUUGUUCCAGCAGCCCCAGGACACUCACAACGAGACCCCUGGGAAGAUGAAACCCCUGGCUGCACAGUGGCCCAGGGUGAGGCUAGCUGGUGUGGUCACCCACUCUCCAGCCCAGCCCCAAUCCCACCACAACCACAUUCGACAAAUGGCCCACUGUGGGCCAACGGACACGUGGCCAUUCCAGAGCUGCCACAUCA*

*>hsa-let-7d*

*GUUAAUUUGAAGUGCAUCUGCCAAGUAGAAGACCAGCAAGAAAAAAAAAAUGGGUUCCUAGGAAGAGGUAGUAGGUUGCAUAGUUUUAGGGCAGGGAUUUUGCCCACAAGGAGGUAACUAUACGACCUGCUGCCUUUCUUAGGGCCUUAUUAUUCACCGAUAACCUGUUUCCUUGCUACUUUGCUUUGGUGUAAGCAGAG*

*>hsa-mir-668*

*CUUGUCUGGAAGGACGAACUGCAUCCUUGCUGCUGGGGAGAAGGCAGUGCCCUCAGCACUCCCUUAAGGUAAGUGCGCCUCGGGUGAGCAUGCACUUAAUGUGGGUGUAUGUCACUCGGCUCGGCCCACUACCCAAUACUAUCCCACCCAUUCCUAACAGGACUCCCGAGAAGGGCGGCCACCUCUGCCAGAGAGGCAAU*

*>hsa-mir-3612*

*AAAUGGUGACCUAUCUUGGGUUUUGCAGAGCAGUAGAAACUUCAGUCAUUCACUUUGGGGACUGGGGAUGAGGAGGCAUCUUGAGAAAUGGAAGGAAUGGGAUCUACUUCCAGUUCACUAGAGGCGUCCUGACACCCCUAGCUCAGCAUCUUCCUCUGUGAACCGCCCAUGGGAAGGUGUCCAAAGGUGUCCUAGUAGAA*

*>hsa-mir-193b*

*CGGUGCUGUCAGCCGUGAAUGGGGACUCACUUCUUGGGAAAAGAGGCUUUUGGAGGCUGUGGUCUCAGAAUCGGGGUUUUGAGGGCGAGAUGAGUUUAUGUUUUAUCCAACUGGCCCUCAAAGUCCCGCUUUUGGGGUCAUUCUGGACAGCGAGGGACUCAGGCCCCGGCAGUCUCGGGAGGGUGAGGGCAGAGAGCUAC*

*>hsa-mir-20b*

*GUUGGGAACAGAUGGUGGGGACUGUGCAGUGUACAGUUGUGUACAGAGGAUAAGAUUGGGUCCUAGUAGUACCAAAGUGCUCAUAGUGCAGGUAGUUUUGGCAUGACUCUACUGUAGUAUGGGCACUUCCAGUACUCUUGGAUAACAAAUCUCUUGUUGAUGGAGAGAAUAUUCAAAGACAUUGCUACUUACAAUUAGUU*

*>hsa-mir-450b*

*GGGAAUAAGCAAAAAAUCUAACAGCGGCUAUCUGUGGUUUACUUCUGUGAUAAAUGUAAGAUGCAGAAUUAUUUUUGCAAUAUGUUCCUGAAUAUGUAAUAUAAGUGUAUUGGGAUCAUUUUGCAUCCAUAGUUUUGUAUCAACAUAUGGAGAAAAGUCUAGGAAGAAAUAUACCAAAACUUGUAACAGUAUUAUAGUAU*

*>hsa-mir-3662*

*ACAAGUCACUUUAUGUUUAGGGUGGUAUUCUAGAAGAGUCAGUUCUAGAGGAAGUGUGUUUUCCUCAACGCUCACAGUUACACUUCUUACUCUCAAUCCAUUCAUAUUGAAAAUGAUGAGUAGUGACUGAUGAAGCACAAAUCAGCCAAACUCUGUUGGUUGUUAUGGUUUGAUAUCGAGGUGUUUCACUGUAUGCUUUG*

*>hsa-mir-4299*

*GGAAACAAAACACCACCUACAAAACUGAUCAAGCCUAUUAGUAAAAUAUGGUCCAUUGAUUGUUUGGGUUCUGACCAAUCAUGUUACAGUGUUUUCUCCUUUAGAGAGAGCUGGUGACAUGAGAGGCAGAAAAAGGACCAUGGUGGGGAGGCCCUGAUGUGAGAAGGUUGGUGGCAGGCAGGAGAACUCUAGCCACUAGU*

*>hsa-mir-583*

*UUAAAGUUAAAAAAAAACCUAUUUAAAUGAUAGGAAGGCUGUUCUUUACAUCUUACAACUCACACAUUAACCAAAGAGGAAGGUCCCAUUACUGCAGGGAUCUUAGCAGUACUGGGACCUACCUCUUUGGUACUUUCCCCUGGGCCCCCUUCAUUUCACUCCCUACCCACACUCUUUGUCCUGAGCUCCCACUUACCAAA*

*>hsa-mir-484*

*CGUCCUGUCCGCGCGGGGCAUGCUGGGAACCCCGGGGGGGGCGGGGCCUCGCGGCCCUGCAGCCUCGUCAGGCUCAGUCCCCUCCCGAUAAACCCCUAAAUAGGGACUUUCCCGGGGGGUGACCCUGGCUUUUUUGGCGAAAACCCCCAGUUUAAGGAGGCCUUCCCCCUGGCUUCACGUUGUGGAGUCGAAAGGAACCU*

*>hsa-mir-135b*

*CUUCUCGGGUGCCCCAGCUUCUCGCUUCCCUAUGAGAUUCCUGCCGCUGGACCCCUCCACUCUGCUGUGGCCUAUGGCUUUUCAUUCCUAUGUGAUUGCUGUCCCAAACUCAUGUAGGGCUAAAAGCCAUGGGCUACAGUGAGGGGCGAGCUCCUUCUCCUGCGCAGCUGCACCUCCCAUGGGACCAGGUUCGGAGCCAG*

*>hsa-mir-1226*

*ACAGCGUCACAUAUAGGACCAAAUCAGGCAACAUCCUGCUGCACAAGUCGACCAUUAACAGGUGAGGGCAUGCAGGCCUGGAUGGGGCAGCUGGGAUGGUCCAAAAGGGUGGCCUCACCAGCCCUGUGUUCCCUAGGGAGGCCACACGGUUACGGAGCCGAUGGCUGACGUAUUUCAUGGCAGUCAAGUCCAAUGGCAGC*

*>hsa-mir-423*

*CCCCGUACAUUUUCCCGGAUGGAAGCCCGAAGUUUGAGGGAGAAACUUGUGAGGAAAUAAAGGAAGUUAGGCUGAGGGGCAGAGAGCGAGACUUUUCUAUUUUCCAAAAGCUCGGUCUGAGGCCCCUCAGUCUUGCUUCCUAACCCGCGCUUGAGUUUCUCCCCGCUUGGAUGCUCUCAGGGGCAGUGUGAAGAGAGACA*

*>hsa-mir-199b*

*GGGCUGAAGCGCCCACCGGAUGGACAGACACUGCUGCCUGGAUGGACCAGAGGACACCUCCACUCCGUCUACCCAGUGUUUAGACUAUCUGUUCAGGACUCCCAAAUUGUACAGUAGUCUGCACAUUGGUUAGGCUGGGCUGGGUUAGACCCUCGGCACCGCCGCCACUGGAGAGCUGGACCGACCCCUGCCUGCCAUCC*

*>hsa-mir-134*

*CUUUGAAGAGAGGCUCCCUGGGCCCCAGGCCGACUUCCAGAAGAGAUGUUGGUGUCAGCACCGUCCAGGGUGUGUGACUGGUUGACCAGAGGGGCAUGCACUGUGUUCACCCUGUGGGCCACCUAGUCACCAACCCUCAGCAUCACUCCCACUCCAGGAAGACUUUCCAGAGCUCCCACCAACUCUGGGGAAGCGGCCAU*

*>hsa-mir-3164*

*AAGAUGCAUGAUCGUGUGUGCACACAUCCACAGUAAGGCCUCAGUGCUAUCCACAGGUUCUUGGAAACUGUGACUUUAAGGGAAAUGGCGCACAGCAGACCCUGCAAUCAUGCCGUUUUGCUUGAAGUCGCAGUUUCCCAGGACCUCUCACGUGAGUAUAUACAUGCUCACGAUGACACGCAACGUGUGCACACGUACAU*

*>hsa-mir-3143*

*GAGCCAUUCUACACUGUCUUAGAGAAAGCUGGACACAGUCAUGUUUGUUUCACUUCAUGUUUGCUUCGCUAGAUAACAUUGUAAAGCGCUUCUUUCGCGGUUGGGCUGGAGCAACUCUUUACAAUGUUUCUAAAUUAACCCACUGGAAAGCAAUGAAGACCCAAAAGUUGUUGUAGGAUACUCCGGAGAAGCUUAUUCAG*

*>hsa-mir-1287*

*ACCAGGCAGUGAGAGGGCCCUCCUCCCAUCCAGGCUCCUAAGCCCUUCUCUCUGCUGUUGUGCUGUCCAGGUGCUGGAUCAGUGGUUCGAGUCUGAGCCUUUAAAAGCCACUCUAGCCACAGAUGCAGUGAUUGGAGCCAUGACAAGUCCCCACACUCCGGGGAGUGGGUGAGUGAGGGGGGUGAGGUGGGACACAGGAG*

*>hsa-mir-543*

*GGAAGCCCGCAGGAGUGAUACCAGCCGCGGAGAAGCAGUGUUGUCAACGGUGCUGACGUGCGGUACUUAAUGAGAAGUUGCCCGUGUUUUUUUCGCUUUAUUUGUGACGAAACAUUCGCGGUGCACUUCUUUUUCAGUAUCCUAUUCUGCCUUGAAGACGUCUUGGUUUGGGUGCAACUUCAGGGAAGGCACAUGGGCCC*

*>hsa-mir-4301*

*UUCUUUUGAAAUAUUGUUUCCAUUCUCAAAUGGCAACAAUAGACUAAGGAUCCCAUUCUCUCCAGUUUACCAGCCACCUCCCACUACUUCACUUGUGAACAUUGCAUUCGUGGAGGGUGGCAGGUGCAGCUCUGGAUGCUGGGAAUAAGUGCAUACAAAAUGGAAGAGAUAUGAACUGCACAGCGAGACAAGUGGAACAG*

*>hsa-mir-154*

*CUUCCUGGAGGAGGCCAACUUUGGAGAACAGGCAUUGACAUUGGGUCUGCGCUAGCGUGUGGUACUUGAAGAUAGGUUAUCCGUGUUGCCUUCGCUUUAUUUGUGACGAAUCAUACACGGUUGACCUAUUUUUCAGUACCAAAUCCCGCCUUGGAGGGCUUCCUGGAGCGGAGUCUUACUUCAGGGUGUCAGCUCCUCUC*

*>hsa-mir-4262*

*GGCCGUUCACCACAAGCUCCACCUCCCGCCAUGAGGCCAGGCAGCCUCUCUGCAGAUACCAAACCACCCCAGAGAGGAAAGCUGCAGGUGCUGAUGUUGGGGGGACAUUCAGACUACCUGCAGCAGAGCCCAUGGCUGACAGGACCUGCACACCCCACAAAGAGACACCCACACCCCCCAUGUUCACAACACAGACACCC*

*>hsa-mir-570*

*UGUAGUUGUGGGCCAAGUGUCCUGGGUUUUGGGUGGAGAAAGAGUCCCCUAGAUAAGUUAUUAGGUGGGUGCAAAGGUAAUUGCAGUUUUUCCCAUUAUUUUAAUUGCGAAAACAGCAAUUACCUUUGCACCAACCUGAUGGAGUCCCCCUUGCCAUGCUUCUCUCUGUGAAAAACCCCAAGCCGAAGUCAGCAUAACAU*

*>hsa-mir-3185*

*CAGAUGUAUUGGUCCAGCCCCCGAGAGACCCAGGAAGAAGGCAAGGAGGUUCGGUACGAGCCAUCUCGAAUGGAAGAAGAAGGCGGUCGGUCUGCGGGAGCCAGGCCGCAGAGCCAUCCGCCUUCUGUCCAUGUCUUCUUUAUCUUCCUCGCCAUAAUAAUUAGUGGUGUGGACCCCCCCACACACACUUUUUUUCGGAU*

*>hsa-mir-411*

*CAAGGCCUUGGAGGGCUUUCUGGAUGUGUCCUGAGCCUUUGUCCUCGGCAUCUCUGUGUGGUACUUGGAGAGAUAGUAGACCGUAUAGCGUACGCUUUAUCUGUGACGUAUGUAACACGGUCCACUAACCCUCAGUAUCAAAUCCAUCCCCGAGGCUCCUGGAAACAACGCUGUCUUCGGGGAGGAACGGUUGGACCCAC*

*>hsa-mir-3675*

*UUCCCGGGGCUGAUCCUGAAUCAGGGAAUCAUAUUUCCUAAUGCAGGACAGAAGAGGAAAGGGUGGAUGAUAAGUUAUGGGGCUUCUGUAGAGAUUUCUAUGAGAACAUCUCUAAGGAACUCCCCCAAACUGAAUUCUGGCACGUAAGCCAUAGGAGGUAUUUAAGAGUAAAUUCUACCCUGAUAAAUUAUUGCACUAAA*

*>hsa-mir-598*

*GCUCCUGCAUGGCCAGUGGGGGCCCAAGCUGCCACAACGCAGGAAGAUGGCUUGAUGAUGCUGCUGAUGCUGGCGGUGAUCCCGAUGGUGUGAGCUGGAAAUGGGGUGCUACGUCAUCGUUGUCAUCGUCAUCAUCAUCAUCCGAGCAGCCACCAGAUAGCUUGCACCCACGCUGGGCUGGGCACGAGACUGGCUGUUUG*

*>hsa-mir-4254*

*GAGGGUGGGUGGUUGGGGGGAGAGGCACAGCCUGUCUGGCUUCCGCCGAAUAACUCCAUGUGGGUCUUGGGAGGAGGGUGGGGUGGCUCCUCUGCAGUGAGUAGGUCUGCCUGGAGCUACUCCACCAUCUCCCCCAGCCCCUGUAUGGCUGGGAGGGGAAGAUGCUUGCUUGGGCCUGGCUUGGAGGUGCAGCAUGAAGG*

*>hsa-mir-1264*

*UCUAAUAGUUUUAAAGAAAAUAUCAUAACAUAUUCAACACAUUGUGUUUCUCCAUGCCUGGCAUAUAGCAGGUCCUCAAUAAGUAUUUGUUGAAAGAAUAAAUAAACCAACAAGUCUUAUUUGAGCACCUGUUAUGUGGAGGAUACAAUCCUACGACCCAGACAGACCUUAUGAUCUAGUAUGGGAGAGAAGAAAUAUAA*

*>hsa-mir-192*

*GCUCCCAGGCCAGAUGGUGGCGGGUAGUGGAGGGCUGUCGGUGGGCUGCCGAGACCGAGUGCACAGGGCUCUGACCUAUGAAUUGACAGCCAGUGCUCUCGUCUCCCCUCUGGCUGCCAAUUCCAUAGGUCACAGGUAUGUUCGCCUCAAUGCCAGCCACCAGGACCUGCAGGGAUAGGGGAGGGCCGGGGGUGUCCAGC*

*>hsa-mir-1252*

*UUUCUACUCACAUUUCCCAUCACACAGUUGUUGAGAAAUUGGUAAGUUGAGAAAAAAUGUGAGGUGUAGAAAGAAGGAAAUUGAAUUCAUUUAGAAAAGAGAAUUCCAAAUGAGCUUAAUUUCCUUUUUUCUAAAUCUUAAUAAUCUACCUUGCUUUUCUUCUCCUUCCACUGCACCCUUUCUCCAUGAAUUCAUCAGUC*

*>hsa-mir-3619*

*CUAAGUAAGUUCCACUUCAGGACUCAUCCGAGAGGUUGUGAAGCCACACGUCAUCAAAAACGGCAUCUUUGCACUCAGCAGGCAGGCUGGUGCAGCCCGUGGUGGGGGACCAUCCUGCCUGCUGUGGGGUAAGGACGGCUGUGUGCGCCAGGCUACAGGGAAGCCAAGGCUACUGCUUGGGGGUUUCACUGGGGAAUAUU*

*>hsa-mir-493*

*AUUCCAGCCUCCAUCACCCGCCUCACCUCCAUCAUCCUCGCUUCGGGGCUCAUUCUGGCCUCCAGGGCUUUGUACAUGGUAGGCUUUCAUUCAUUCGUUUGCACAUUCGGUGAAGGUCUACUGUGUGCCAGGCCCUGUGCCAGGCAUCGUGUAGAUGGACGUUGCCCUUGUGAGUGCACGCCUCGGGGAGACGCACGCCA*

*>hsa-mir-623*

*AAGAAAUGUCAUUAAAGUAAACAAGACUUGGGACACAUGUCUGUCAGACUGUACACAGUAGAAGCAUCCCUUGCAGGGGCUGUUGGGUUGCAUCCUAAGCUGUGCUGGAGCUUCCCGAUGUACUCUGUAGAUGUCUUUGCACCUUCUGUCCUCAUUGCCAUCCCCAAUCAGUCCCUGGAACACCAGCUGUGGCCCUGACC*

*>hsa-mir-137*

*AGCAGCGGCAGCGGUAGCAGCGGCAGCGGUAGCAGCGGCAGCGGCAGCUUGGUCCUCUGACUCUCUUCGGUGACGGGUAUUCUUGGGUGGAUAAUACGGAUUACGUUGUUAUUGCUUAAGAAUACGCGUAGUCGAGGAGAGUACCAGCGGCAGGGGGGCAGCGGCCGCCCUCCCCAGCCCACCAGCUGGCCACUAAACGC*

*>hsa-mir-892b*

*AAACCUGCAGGGGACAAUGGUGGGGCACAGGGCAGCGCUUCCGCAAUCAGCCAUGCUGUGUGCAAUGCCCUACUCAGAAAGGUGCCAUUUAUGUAGAUUUUAUGUCACUGGCUCCUUUCUGGGUAGAGCAAGGCUCACCAUGUAUAUAUUUGUGGUGGCCUUUGUACCCGUGUUGGAGUAGAGCUGCAAAAGCCUCAAGC*

*>hsa-mir-325*

*UGUGAGAAAAAGUUGCUUAAAUAUAGGUUUUGAGAUGGAUUCAAGUCCACAGAACCAAUACAGUGCUUGGUUCCUAGUAGGUGUCCAGUAAGUGUUUGUGACAUAAUUUGUUUAUUGAGGACCUCCUAUCAAUCAAGCACUGUGCUAGGCUCUGGGACUACAUAGAUAAGUUAUUCAACAUCCUACAUUUUGAGGUACUU*

*>hsa-mir-3916*

*AUAUUCUCAAAUGCAGGUGUGAUUUGUCCAGAUAAUCUCACCUGAGAAGGAAUCCCAGAGAAGAAGGAAGAAGAGGAAGAAAUGGCUGGUUCUCAGGUGAAUGUGUCUGGGUUCAGGGGAUGUGUCUCCUCUUUUCUUCUGGGAUGUUAUGUGUUUAGAACUUGCAAACCUUUAUUUCUGUACUUCUGAUGUACCUGCCU*

*>hsa-mir-518f*

*UUUAACCGGGUGAAGGAAAAUUCCAACAAAAAACCCAGAGUGUUGGAGCAAGAAGAUCUCAUGCUGUGACCCUCUAGAGGGAAGCACUUUCUCUUGUCUAAAAGAAAAGAAAGCGCUUCUCUUUAGAGGAUUACUCUUUGAGAAAAGCAACGUUGAAGUUGAUGCUUAUCUUGGUAAUAAAUUUGCAGAGAAUGCUUAUA*

*>hsa-mir-222*

*UUCUUCCACAGAGCCCCUCCCCAGAAGGCAAAGGAUCACCCAGCUGCUGGAAGGUGUAGGUACCCUCAAUGGCUCAGUAGCCAGUGUAGAUCCUGUCUUUCGUAAUCAGCAGCUACAUCUGGCUACUGGGUCUCUGAUGGCAUCUUCUAGCUUCUGCUUCAGUGUCCUGAGAGAGAUUAUUGGGCAGUCUUUAAUAAUGA*

*>hsa-mir-3129*

*CAUUCCAGCCUGCCGACAGAGCAAGACUCGGUCUCAAAAAAAAAAAAAAAAAAAAAAAAAGUCGUACUUGGGCAGUAGUGUAGAGAUUGGUUUGCCUGUUAAUGAAUUCAAACUAAUCUCUACACUGCUGCCCAAGAGCAUUCUAUAUUCAACUUCUUUAUCUCUGAGAAAUUGCUUAUAUCUCCCCAACUGUUAUUUCC*

*>hsa-mir-4321*

*GGGAGGAAGGGGACCGGUAGAGCGGGGCUGGGUAAGCCUCCAUCCAGCCGGGCUGAGCCCUGGUCUCCGCAGAGCCUCUGCCCCUCCCGAGACACCCGCUACCUGGUGUUAGCGGUGGACCGCCCUGCGGGGGCCUGGCGCGGCUCCGGGCUGGCCUUGACCCUGCAGCCCCGCGGAGAGGGUAGGUCCGCGUGGAGAGG*

*>hsa-mir-4319*

*UUUCCCAUUUCCUUUCGGUGAUAACUCACUGUACUAAGUCAGAGCCCAUCAUCACCUUUUUGGCUUGAGUCCCUGAGCAAAGCCACUGGGAAUGCUCCCUGAGGACGUUAUAUGAGUGCUCAGCUCAUGGGGCUAUGAUGGUCAAAGGAGAAAAUGCACAUGAAUGUAUUUUGAAAAAUAUGUCACUAUCACGGAGCAGC*

*>hsa-mir-3174*

*AUUAACCUUGAUGUGAACCUUGCCACUCCAUAUGUGGUCCAUGGACCAGCAUCAGCGUUACCUGGUAGUGAGUUAGAGAUGCAGAGCCCUGGGCUCCUCAGCAAACCUACUGGAUCUGCAUUUUAAUUCACAUGCAUGGUAAUGUCUGUAAAGCACUGAUGGACUCAAAAGUACUGAAAUGACAGGUUCUCAGCUUUGCU*

*>hsa-mir-648*

*AGUAAAUACUGCCCUCCUGCAGUCCACAGUGUCUGGAGGGGACCAAUAGACAGAUGAUCACAGACACCUCCAAGUGUGCAGGGCACUGGUGGGGGCCGGGGCAGGCCCAGCGAAAGUGCAGGACCUGGCACUUAGUCGGAAGUGAGGGUGCAGUUGAGCAGGAACUGAGGGUGACGGGCCAGCACAGCCGCAUCUGGGCG*

*>hsa-mir-1206*

*GCCUGAAAAACACAGCCAGGUCCAUCUAGGAACUAGAAGCUAUGGUCAGGCUUACAUGCUUUUUUCAUAUCAGUGUUCAUGUAGAUGUUUAAGCUCUUGCAGUAGGUUUUUGCAAGCUAGUGAACGCUGCCAAAUUAUGCUCGCCACACAAUUGCAGCCACCUAUUCCCAGGCAGUAUAAAUAUAUGGCUGAACUAACAU*

*>hsa-mir-586*

*ACUUUGUAAAUUCAAAGAAAAGCAAUUAUCUAACAAAGUCAAAUUCUUAUUAAAAGACAUGGGGUAAAACCAUUAUGCAUUGUAUUUUUAGGUCCCAAUACAUGUGGGCCCUAAAAAUACAAUGCAUAAUGGUUUUUCACUCUUUAUCUUCUUAUUUUAUGUAAAUGAAAAAAUAGUAUUGUCAAUUUUUUUCUGCAAAU*

*>hsa-mir-584*

*UUGAGGCAAUGGAGAGUUGUUGCUCUAAGAAAUUGUCAACUCAUGGGAGAGUGACUUAGGGUGACCAGCCAUUAUGGUUUGCCUGGGACUGAGGAAUUUGCUGGGAUAUGUCAGUUCCAGGCCAACCAGGCUGGUUGGUCUCCCUGAAGCAACCUUCUUGCUUUUUUUCAGAGGAGAAUCAAGGUAGGGCCCUGUGUUCU*

*>hsa-mir-10b*

*AUUGGGGCCGGCAGCGACCUAGGUACCUCACUCUGGGUGGGACCCAGAGGUUGUAACGUUGUCUAUAUAUACCCUGUAGAACCGAAUUUGUGUGGUAUCCGUAUAGUCACAGAUUCGAUUCUAGGGGAAUAUAUGGUCGAUGCAAAAACUUCACGUUUCUUCGGAAUAGCCAGAGACCAAAGUGCGACAUGGAGACUAGA*

*>hsa-mir-1179*

*UGCUUAUUUUCUUUAUGGACAAAGGGUCUACUUUCAAGAAGAUAACUGAGCUCUUAUUGGCUGGAAAGGAAGAAGCAUUCUUUCAUUGGUUGGUGUGUAUUGCCUUGUCAACCAAUAAGAGGAUGCCAUUUAUCCUUUUCUGACUAGCUGAGUGAGCAGGGUACACAGAAGGAUUGAAUCUGCCUUAGUUCCAUCCAGUA*

*>hsa-mir-1181*

*UCACACAGGGCGGGGUUUCCCGCGCUCGGCGGAAGCGGAAGGGGCGGGGCUCCGCGCUCCUAGUCUCCACUGCUGCCGCCGUCGCCGCCACCCGAGCCGGAGCGGGCUGGGCCGCCAAGGCAAGAUGGUGGACUACAGCGUGUGGGACCACAUUGAGGUGUCUGAUGAUGAAGACGAGACGCACCCCAACAUCGACACGG*

*>hsa-mir-144*

*CCUGUUCUGCCCCCAGCCCCUCACAGUGCUUUUCAAGCCAUGCUUCCUGUGCCCCCAGUGGGGCCCUGGCUGGGAUAUCAUCAUAUACUGUAAGUUUGCGAUGAGACACUACAGUAUAGAUGAUGUACUAGUCCGGGCACCCCCAGCUCUGGAGCCUGACAAGGAGGACAGGAGAGAUGCUGCAAGCCCAAGAAGCUCUC*

*>hsa-mir-503*

*CCUGGCUAGGCUGGGGCGCAUAGGCGGUGCGAGUCGAGGAGAGACGCGGUGCCCGCGCUCAGCCGUGCCCUAGCAGCGGGAACAGUUCUGCAGUGAGCGAUCGGUGCUCUGGGGUAUUGUUUCCGCUGCCAGGGUAAGUCUGGGACUGCCGUUCGGGCCUACCCAGCAGGUAAGUCUUAGGCCAUCGCCUGCGGUGUCGG*

*>hsa-mir-1225*

*GUGGAGUUGUUCCUGCGCAGGCUGCGCCUCUGGAUGGGCCUCAGCAAGGUCAAGGAGGUGGGUACGGCCCAGUGGGGGGGAGAGGGACACGCCCUGGGCUCUGCCCAGGGUGCAGCCGGACUGACUGAGCCCCUGUGCCGCCCCCAGUUCCGCCACAAAGUCCGCUUUGAAGGGAUGGAGCCGCUGCCCUCUCGCUCCUC*

*>hsa-mir-1229*

*GCCCUGCAGGAGGGCCGCACCGCCACCCUCCGGUACCCUCGGAGCCCCGACGGCUACCUCCAGAUCGGUGGGUAGGGUUUGGGGGAGAGCGUGGGCUGGGGUUCAGGGACACCCUCUCACCACUGCCCUCCCACAGGCUCCUUCUACAAGGGAGUGGCAGAGGGAGAGGUGGACCCAGCCUUCGGCCCUCUGGAAGCACU*

*>hsa-mir-125a*

*UGCCUAUCUCCAUCUCUGACCCCCACCCCAGGGUCUACCGGGCCACCGCACACCAUGUUGCCAGUCUCUAGGUCCCUGAGACCCUUUAACCUGUGAGGACAUCCAGGGUCACAGGUGAGGUUCUUGGGAGCCUGGCGUCUGGCCCAACCACACACCUGGGGAAUUGCUGGCCUGACUUCUGACCCCUGACUCCUCAUACC*

*>hsa-mir-1265*

*GGAUGGGGGAGGUAGGAGAGCAGACGUCUAUUGAUUUGGUGUUUUAGCUAAUCGACCUAUGGUUUGGGACUCAGGAUGUGGUCAAGUGUUGUUAAGGCAUGUUCAGGAACAAUACUUGACCACAUUUUGAAUUCCAAACCAUAUUCUACAACUUUUUCCACAUAUUUGGUGUAAUCCUCCAUUUUCUAUUUUUGAGUUCG*

*>hsa-mir-1286*

*GACCUGGCUCAGAGUGCGUCCCCAGGGUGGGAGCAGGCCGGCCACCGGUCACUUGCUCAUUAGUGUCCUCUGGGGACUCAGCUUGCUCUGGCUGCUGGAUUGAAUUAGCUGCAGGACCAAGAUGAGCCCUUGGUGGAGACAGGAUUCUCCAGAUAAUUAGGCAGCAGGUGUUUGUUGGCUCCUGGUGGAGCAUGGAGGAG*

*>hsa-mir-1293*

*AGAUCGUGCCACUGCACUCCAGCCUGGGCAACAGAGCAAGACUCUGUCUCAAAAAAAAAAAAAAAGGUUGUUCUGGGUGGUCUGGAGAUUUGUGCAGCUUGUACCUGCACAAAUCUCCGGACCACUUAGUCUUUAAAUGGAGAAAACCUGUUCCUCCUUGAAAAGAACUUUAAUUUUUUUCUCAGAGGUCUUCCAAUCCA*

*>hsa-mir-1304*

*UAGCUGGACACAGUGGUGCCUGUGGUCCCCAGCUACUCUGGAGGCUAAGGCAGGAGAAACACUUGAGCCCAGCGGUUUGAGGCUACAGUGAGAUGUGAUCCUGCCACAUCUCACUGUAGCCUCGAACCCCUGGGCUCAAGUGAUUCAGCAAGAAUCACACGAUUUUUAAAAGACAUGGCAGAUCCUGUGUCUUUAAAAAA*

*>hsa-mir-1322*

*UGGCGAUCUUGCAUUGGAAAGAAGCAAUUUAACCUAAUAGAACCAACUCCCCAGUUUGUCUGUAGAACAGUAUCAUGAAUUAGAAACCUACUUAUUACAUAGUUUACAUAAGAAGCGUGAUGAUGCUGCUGAUGCUGUAAUAUCUAGUCUCUGUUGAUGGUUCUUUCCUGGGAGGUUGGAUGUGUUUCUACCUUGAUAUG*

*>hsa-mir-136*

*UGGCUGGCCCUGCCUCUCCCCGCACUCCACUGCCCGACGUCGCCUCGGUGGUGUUGGAUGAGCCCUCGGAGGACUCCAUUUGUUUUGAUGAUGGAUUCUUAUGCUCCAUCAUCGUCUCAAAUGAGUCUUCAGAGGGUUCUAUCAUUUCGUCGGAUGGAAAGGAGUGUAUUCUGAAGAUUGGUAAGGUUGUGAUGGCGUCC*

*>hsa-mir-141*

*CUGUGGCCAGGGUCCCCUGUAGCAACUGGUGAGCGCGCACCGUAGUUCUCUGUCGGCCGGCCCUGGGUCCAUCUUCCAGUACAGUGUUGGAUGGUCUAAUUGUGAAGCUCCUAACACUGUCUGGUAAAGAUGGCUCCCGGGUGGGUUCUCUCGGCAGUAACCUUCAGGGAGCCCUGAAGACCAUGGAGGACUACUGACCA*

*>hsa-mir-143*

*UCUCCUGGCCAGGUUGGAGUCCCGCCACAGGCCACCAGAGCGGAGCAGCGCAGCGCCCUGUCUCCCAGCCUGAGGUGCAGUGCUGCAUCUCUGGUCAGUUGGGAGUCUGAGAUGAAGCACUGUAGCUCAGGAAGAGAGAAGUUGUUCUGCAGCCAUCAGCCUGGAAGUGGUAAGUGCUGGGGGGUUGUGGGGGGCCAUAA*

*>hsa-mir-146a*

*GCUGGGACAGGCCUGGACUGCAAGGAGGGGUCUUUGCACCAUCUCUGAAAAGCCGAUGUGUAUCCUCAGCUUUGAGAACUGAAUUCCAUGGGUUGUGUCAGUGUCAGACCUCUGAAAUUCAGUUCUUCAGCUGGGAUAUCUCUGUCAUCGUGGGCUUGAGGACCUGGAGAGAGUAGAUCCUGAAGAACUUUUUCAGUCUG*

*>hsa-mir-1539*

*CAAAGGCGCGCUUGCGUCAACCCGGGGUCGCACAGCACGCAGCAGUGUUGCCCAAGGGGCGCGGCGAGGCUUCCGGCUCUGCGGCCUGCAGGUAGCGCGAAAGUCCUGCGCGUCCCAGAUGCCCAACUAAAAGCUUCGCUGCGGGAAGAAAGCCUGACCUGACUACACCCAGACCCAGAACGCAGGAUCUCGGUAAAGGA*

*>hsa-mir-181d*

*GGGGCCUGGGGAGCCCCCAAUCCAGCCUGGGCACGUCCCCUCCCCUAGGCCACAGCCGAGGUCACAAUCAACAUUCAUUGUUGUCGGUGGGUUGUGAGGACUGAGGCCAGACCCACCGGGGGAUGAAUGUCACUGUGGCUGGGCCAGACACGGCUUAAGGGGAAUGGGGACUGGGGACAGGACCCCCCACCGCCACAGUC*

*>hsa-mir-183*

*GGGAACGGGCAUCGUGGGCCGCUGGUCUCUCCGCAGGGUCGGCAGGCCGCAGAGUGUGACUCCUGUUCUGUGUAUGGCACUGGUAGAAUUCACUGUGAACAGUCUCAGUCAGUGAAUUACCGAAGGGCCAUAAACAGAGCAGAGACAGAUCCACGAGGGCCUCCGGAGCACCUUACCCACUUCUGCCUUGAGUGCUCCUA*

*>hsa-mir-187*

*GGCAGCCCACCUGGAAGAGGAGGCCAGGCAGGUGCUGCAGGUCGGGCUCACCAUGACACAGUGUGAGACCUCGGGCUACAACACAGGACCCGGGCGCUGCUCUGACCCCUCGUGUCUUGUGUUGCAGCCGGAGGGACGCAGGUCCGCAGCAGAGCCUGCUCCGCUUGUCCUGAGGGACUCGACACAGGGGACUGCACAGA*

*>hsa-mir-1908*

*CCGGCGCAUGCGCAGACGAAACAGGCACCAACGCUGGAGCUUCCCGCAGUGUGAUUUGGGGCCGGGAAUGCCGCGGCGGGGACGGCGAUUGGUCCGUAUGUGUGGUGCCACCGGCCGCCGGCUCCGCCCCGGCCCCCGCCCCACACGCCGCAUCACUUACAGGGCCCGGGGCUGCCGGACCUGCCAACGUGAAUCUUAUC*

*>hsa-mir-1909*

*CCUGGGCCUCCCCUACAAGCGGUCCCUGCGGAACCUCAUGGCCGACUACCUCAGACAGAUCAUCCAGGACAAUGGUGAGUGCCGGUGCCUGCCCUGGGGCCGUCCCUGCGCAGGGGCCGGGUGCUCACCGCAUCUGCCCCGCAGUGGAUGGGCACAGCUCCAGCGAGGACGCCGGCGCCUGCAUGCACCUGGUGAUCUGG*

*>hsa-mir-190b*

*GUCUGCUCUAUUCUUUCUUUGCAACUGGAAGGAAGGCAGAUGACCCCCAAAGCUCUCCUGCCUGCUUCUGUGUGAUAUGUUUGAUAUUGGGUUGUUUAAUUAGGAACCAACUAAAUGUCAAACAUAUUCUUACAGCAGCAGGUGAUUCAGCACCACCCUCUUUCAUACUUCAAUCUCUGGGGCUCCUGUCUCUUUUACUG*

*>hsa-mir-1915*

*CUCCCCAGGGCGCGGACCACGGUGUCCCCUUCUCUCCAGCUGGGGGUCUCGGGUCCUGGCGCUGAGAGGCCGCACCUUGCCUUGCUGCCCGGGCCGUGCACCCGUGGGCCCCAGGGCGACGCGGCGGGGGCGGCCCUAGCGACCUGCGGCGGCGCCGGGAAAGCCCUGCCUCUGCAGCGGGUCCCAGGGGUCGGGGCCUG*

*>hsa-mir-205*

*ACAGGCUGAGGUUGACAUGCAUCCCCACCCUCUGAGAAAAAGAUCCUCAGACAAUCCAUGUGCUUCUCUUGUCCUUCAUUCCACCGGAGUCUGUCUCAUACCCAACCAGAUUUCAGUGGAGUGAAGUUCAGGAGGCAUGGAGCUGACAACCAUGAGGCCUCGGCAGCCACCGCCACCACCGCCGCCGCCACCACCGUAGC*

*>hsa-mir-208a*

*AGUGCUUGGAAGAAAGCCUGAACUCUUUGCUCUGUGAACUCUGGCUGGCCCUGACCCACUUCCUGUGACGGGCGAGCUUUUGGCCCGGGUUAUACCUGAUGCUCACGUAUAAGACGAGCAAAAAGCUUGUUGGUCAGAGGAGCUACCGUCGAUCAGCCUGUGUGGGGGGUGAGGGCAGGGGGCACUGACACCCAGAUGCC*

*>hsa-mir-211*

*UCACAUCUCUAAUUUUUCUGGACUUGUAAGUUUUUACAACACCCCAUUUCACCUGGCCAUGUGACUUGUGGGCUUCCCUUUGUCAUCCUUCGCCUAGGGCUCUGAGCAGGGCAGGGACAGCAAAGGGGUGCUCAGUUGUCACUUCCCACAGCACGGAGCUUCGAGGACUCCAGCCUAAGCCAAGAAGAACCUGUUCUACU*

*>hsa-mir-216a*

*UGAGGCAGCCACGUGCCUCUGCUCAUACACUUUCUCUUGCAGGUCUAACACGGAUGGCUGUGAGUUGGCUUAAUCUCAGCUGGCAACUGUGAGAUGUUCAUACAAUCCCUCACAGUGGUCUCUGGGAUUAUGCUAAACAGAGCAAUUUCCUAGCCCUCACGAAUCAAAAGACUUGCAUGGCAACCAAAAUUAAACACCAA*

*>hsa-mir-21*

*AUCCUGCCUGACUGUCUGCUUGUUUUGCCUACCAUCGUGACAUCUCCAUGGCUGUACCACCUUGUCGGGUAGCUUAUCAGACUGAUGUUGACUGUUGAAUCUCAUGGCAACACCAGUCGAUGGGCUGUCUGACAUUUUGGUAUCUUUCAUCUGACCAUCCAUAUCCAAUGUUCUCAUUUAAACAUUACCCAGCAUCAUUG*

*>hsa-mir-221*

*AGUAGGCAGUUGUGUUGAAAUAGUAUGUGAGAAUUACUUGCAAGCUGAACAUCCAGGUCUGGGGCAUGAACCUGGCAUACAAUGUAGAUUUCUGUGUUCGUUAGGCAACAGCUACAUUGUCUGCUGGGUUUCAGGCUACCUGGAAACAUGUUCUCCAUUGGCUGUCUCACCAAUGCUACCCCUAUAAUGUUUCUGAAUAA*

*>hsa-mir-26b*

*GCCUGCAGAGUGGGCUCCUCCUCUAGGCUCCCCCGUGCUGUGCUCCCUCGCCCCACCCUGCCCGGGACCCAGUUCAAGUAAUUCAGGAUAGGUUGUGUGCUGUCCAGCCUGUUCUCCAUUACUUGGCUCGGGGACCGGUGCCCUGCAGCCUUGGGGUGAGGGGGCUGCCCCUGGAUUCCUGCACUAGGCUGAGGUUGAGG*

*>hsa-mir-2909*

*UUCAUCUUUGUGACAGGAUAAUUCUAGGGGUGGUAGGCAAUUGUUGUCCAUGGCUUUUGCUUCUCGGUGGUGUUAGGGCCAACAUCUCUUGGUCUUUCCCCUGUGGUCCCAAGAUGGCUGUUGCAACUUAACGCCAUCAAACAUGCAUUCAAGGCAGGAAGAAGAGGAAAGAACUGUGCCAGCCAUACCUGUUUGUUUUU*

*>hsa-mir-298*

*CAGCCACAUGGACCUGAUGCUAGAGGCCUGUCAGCACUGGGGGACCCGGGAGAAGCAGCUCAGGUCUUCAGCAGAAGCAGGGAGGUUCUCCCAGUGGUUUUCCUUGACUGUGAGGAACUAGCCUGCUGCUUUGCUCAGGAGUGAGCUGCCGGCUCCCCUUUGAUGUGCUGCGUCUGUUCAGAACUAGGCAGGGGUGCUUC*

*>hsa-mir-301a*

*CGUUUCUCACUGAAGCUUCAACCGAUGCAAGAUGCUUUUGUGUUCCUUUCCUACUUAUUACUGCUAACGAAUGCUCUGACUUUAUUGCACUACUGUACUUUACAGCUAGCAGUGCAAUAGUAUUGUCAAAGCAUCUGAAAGCAGGAUGCACGCCAGAAUUUUGAUUCUUACCUUACAUCUUUCUUCGGUAUCAGUAAAUA*

*>hsa-mir-302c*

*UUUUAGUAGGAGUGAAUCCAAUUUACUUCUCCAAAAUAGAACACGCUAACCUCAUUUGAAGGGAUCCCCUUUGCUUUAACAUGGGGGUACCUGCUGUGUGAAACAAAAGUAAGUGCUUCCAUGUUUCAGUGGAGGUGUCUCCAAGCCAGCACACCUUUUGUUACAAAAUUUUUUUGUUAUUGUGUUUUAAGGUUACUAAG*

*>hsa-mir-302e*

*CAGUUAAUAGAGUCUCUAGGCAGACUGCCUGGGUUUGAAACCUGGCUCUCCUAAUUAUUGGCUGUUUCACCUUGGGUAAGUGCUUCCAUGCUUCAGUUUCCUUACUGGUAAGAUGGAUGUAGUAAUAGCACCUACCUUAUAGAAUUAUUUUGAGGAAUCAAUGAGUUAUUAUUUGUAAAGUGCUUAGAACAGCAACAGCU*

*>hsa-mir-3065*

*GGGGUGGGUUGGGGUUGGGGGCAGGGCAGCUGGGGCCCGGCAGGCAGGUCGGGAGGCUGGCAGCUGCCCUCUUCAACAAAAUCACUGAUGCUGGAGUCGCCUGAGUCAUCACUCAGCACCAGGAUAUUGUUGGAGAGGACAGCCGUGCGGCGUCUGGGGCUGACGGGGCCACGUGUCCUUCGCCACUUCUUCCAGCAUGU*

*>hsa-mir-30b*

*GAGUCUUACGUAAAGAACCGUACAAACUUAGUAAAGAGUUUAAGUCCUGCUUUAAACCAAGUUUCAGUUCAUGUAAACAUCCUACACUCAGCUGUAAUACAUGGAUUGGCUGGGAGGUGGAUGUUUACUUCAGCUGACUUGGAAUGUCAACCAAUUAACAUUGAUAAAAGAUUUGGCAAGAAUAGUAUACAGAGGCUUGA*

*>hsa-mir-3115*

*AACUUCCUGACCAGAAUUUGGAAGACUAUGAUGUAAUUCCCUGGGCCUUGAUUCCAGAAGAAGGAGCUCUCUGAAUAUGGGUUUACUAGUUGGUGGUGAAUUCAUGAGUCGCCAACUAUUAGGCCUUUAUGUCCAGAUAUUAUCCUAAUUUAUGGAGUAAGGUCCCUGAGUUCUCCUCUUUCCUUCCACAGGGCAAGCAG*

*>hsa-mir-3123*

*CUGUGCAGGGAGGCCCAAUUUUUUUCCAGGUGGCCAUGCAUCAGAAUUUUGUUAGAAUGUAACAUGGAUUUGAUUGAAUGAUUCUCCCAUUUCCACAUGGAGAGUGGAGCCCAGAGAAUUGUUUAAUCAUGUAUCCAUGAUUCAAUAUUCAUUUAUGGAGCACCUAUGCUGGGCCAGAGGUACUAUGAGUGGGGAUGACA*

*>hsa-mir-3126*

*CAGUCAAGUUCCUAAUUUAAAGAUGUACGUGAUUCAUGUGGUUGACCAGGAGAGAUGGGGAAGAUGAUUAUAUGAGGGACAGAUGCCAGAAGCACUGGUUAUGAUUUGCAUCUGGCAUCCGUCACACAGAUAAUUAUUUAUUCCACAUCUGCAGUGAUAACUGUCAUAAUAUACUGCAUUUUCUGUAAUGCUAUGUACCU*

*>hsa-mir-3128*

*UUGAAGAACCAUGAAUAUUUCGCAAAAAGAAAAUAAUUUUUUUAACCAUUAAAAUUCCUGGUAGUGACUUCCUCUGGCAAGUAAAAAACUCUCAUUUUCCUUAAAAAAUGAGAGUUUUUUACUUGCAAUAGGAAAACUUGCCAAUUUUUCAAGUUAUUUUUAUUCUUGAUGAUUCUCAAUGAGACAUAAUUAAAAUACAC*

*>hsa-mir-3137*

*GAUUAAUCUUGUCAGCUGCUGGGAACACAAAUGUGUGUGCUGAUUCAUUACAGUAGCAUGGUAUACAGGUCUGUAGCCUGGGAGCAAUGGGGUGUAUGGUAUAGGGGUAGCCUCGUGCUCCUGGGCUACAAACCUGUACAACAUGUUUCUGUACUGAACACUGUAGGCAGCUAUAACACAGGGGUAAAUAUUUGUAUAUC*

*>hsa-mir-3138*

*GGACCUGCUCUGGGGCCCAGCCACGCCAUCUCCUGCCACCCAGGGCUGGAGCCUUCCCCUCCCCUCCUCGGCACUUCCCCCACCUCACUGCCCGGGUGCCCACAAGACUGUGGACAGUGAGGUAGAGGGAGUGCCGAGGAGGGCACAGCUGUGCCUCAGAGCCAGCCCCAUGAGUGGCGGCAGGAGGGGAGCGCAAGAGG*

*>hsa-mir-3152*

*AUUCCUUGUGCAUAUAUUUGGCCAGCAUGAGUUCGUGAACUUCUCACAACAACCUUGAGAGCUGUGCAGAGUUAUUGCCUCUGUUCUAACACAAGACUAGGCUUCCCUGUGUUAGAAUAGGGGCAAUAACUCUGCACUGCAUUGCAGCCACCCACCCAUCUAAGGAGGAAGUCCAUGCUCUUUCCAUUCUGCCAUGCUGC*

*>hsa-mir-3168*

*CUAAUGUGUAAAUGUUUUUGUUCCUCCUCAUUGAUGACAAAAUGAUGGAAUAAUGCUUAAAGAUCAUGAGUUCUACAGUCAGACAGCCUGAGUUGGAGGCUCAUCUUCACUUCUUGCUGUGUGACCCUGGGCCAGUGACUUAACUUCUCUAUGCCAUUUUCUUAUCUGCAAAAGGAGGGUAAUACUAGUAUCUGUCUCAU*

*>hsa-mir-3173*

*AGAAACAUUGGAGGUCUAGGGCUUAUUUUCCAGAUAGAAUUGAGUCUUUGUUGGUCUUGGGCCAGCUUCCCUGCCCUGCCUGUUUUCUCCUUUGUGAUUUUAUGAGAACAAAGGAGGAAAUAGGCAGGCCAGGGAAACGAUCUCUCUCCCUCUCUUGUGCCGAGGAAGAACUGUCCAGAUUUUUCCUCAAUUUGUUUCCG*

*>hsa-mir-3177*

*UUCUCGGGCCACUGCUGCAGAGUGUGUGUGUGUCUGUGUGGCCACAUGUGUGCCGUCACCACGUGCCAUGUGUACACACGUGCCAGGCGCUGUCUUGAGACAUUCGCGCAGUGCACGGCACUGGGGACACGUGGCACUGGCUGAACUGCCACACAGCCCCGUUCCAGGGCCUCUUGUCAAAGCCAAGAGGGCUCGUGUGU*

*>hsa-mir-326*

*AGGGUUAAGUAGCAGCGGGACUCCCAUCAAGAAGAAGGAAUGUCUUCCGGAGCCUCAUCUGUCUGUUGGGCUGGAGGCAGGGCCUUUGUGAAGGCGGGUGGUGCUCAGAUCGCCUCUGGGCCCUUCCUCCAGCCCCGAGGCGGAUUCACCAUGAGGCUGAUGCAGCUUCAGCUUCCAGCCCUUCACGCUCCAGGGCCCUU*

*>hsa-mir-32*

*AUGUUGUCUCCUGAAUGUAUACAUCACACCCUUCUUCCCUGAUGCUCCCAUUCUGCUUGCUCUGGUGGAGAUAUUGCACAUUACUAAGUUGCAUGUUGUCACGGCCUCAAUGCAAUUUAGUGUGUGUGAUAUUUUCACAUGAGUGCAUGCACACGGGUAUGGCUCUUGCUUGCUGGGAUUACAGCUUUUCAGUGCCAGUG*

*>hsa-mir-338*

*GGGGACAUGCUGGAAGAAGUGGCGAAGGACACGUGGCCCCGUCAGCCCCAGACGCCGCACGGCUGUCCUCUCCAACAAUAUCCUGGUGCUGAGUGAUGACUCAGGCGACUCCAGCAUCAGUGAUUUUGUUGAAGAGGGCAGCUGCCAGCCUCCCGACCUGCCUGCCGGGCCCCAGCUGCCCUGCCCCCAACCCCAACCCA*

*>hsa-mir-342*

*GCAGACUAAGAUGGAGUUCCUGAACCAAGACCGCUUGCUGGCCAACCUGUGAAACUGGGCUCAAGGUGAGGGGUGCUAUCUGUGAUUGAGGGACAUGGUUAAUGGAAUUGUCUCACACAGAAAUCGCACCCGUCACCUUGGCCUACUUAUCACCACCCCAAACAGAGGAACACGCCUUCUCCAGCCACAGCCUAUGGAAG*

*>hsa-mir-345*

*UCCCCAGUGCUCAUGUUAGUUUCCUUUUAGAGUCUAAGUAGAAUGUUAAGCAGAGACCCAAACCCUAGGUCUGCUGACUCCUAGUCCAGGGCUCGUGAUGGCUGGUGGGCCCUGAACGAGGGGUCUGGAGGCCUGGGUUUGAAUAUCGACAGCCUCUCUGACCCACUUGGUUGCCUCAGGGAGGCAGGUGUGCGGAUGGG*

*>hsa-mir-34a*

*GAGGCAGGACAGGCCUGUCCCCCGAGUCCCCUCCGGAUGCCGUGGACCGGCCAGCUGUGAGUGUUUCUUUGGCAGUGUCUUAGCUGGUUGUUGUGAGCAAUAGUAAGGAAGCAAUCAGCAAGUAUACUGCCCUAGAAGUGCUGCACGUUGUGGGGCCCAAGAGGGAAGAUGAAGCGAGAGAUGCCCAGACCAGUGGGAGA*

*>hsa-mir-3609*

*AGAAAUGCAUAAAUAUGACACAUUUAUCUCAGCUCAGAUGUUGUGUACUUUGAGAUUAAUCGUAACAGUAACUUUUAUUCUCAUUUUCCUUUUCUCUACCUUGUAGAGAAGCAAAGUGAUGAGUAAUACUGGCUGGAGCCCCAAAGAGGCACGUGUGUGUGUUUGUGUGUGUGUGUAUAUGCUUGUCAGUGCAUGCACGU*

*>hsa-mir-3611*

*AAAGUUCUCAAUGAGGAAAGAAAAAAAUAAUAGACUGAGAUUGCUAAGACCUACUGUAAGCAGGUCUAAUAAGAAUUUCUUUUUCUUCACAAUUAUGAAAGAAAAGAAAUUGUGAAGAAAGAAAUUCUUACUAGUUUUGCUGUCAAAUCUCAAUCUGUAAAAGUGAUAGUUAUAUCGUGUAAUAAGUGCUUAGUUAAUUU*

*>hsa-mir-3614*

*CUGGGAAGUGAAGUCAUGAGUGCCCGAUUCCUCUUAGAGAAAAUCCAUAGCUACUGUAGGUUCUGUCUUGGGCCACUUGGAUCUGAAGGCUGCCCCUUUGCUCUCUGGGGUAGCCUUCAGAUCUUGGUGUUUUGAAUUCUUACUAUAGAUGUUUUUAAAGUUCCAAAGUCAUUGAGUUUCAAUGUUACAUAAACUCCAUU*

*>hsa-mir-3616*

*GUACCGCAGGGGCUGGCACCCCCGCACAUUUUUUGCCCCAUCAACAUAUUAUCAUGUCACUCCGCCAGCAUCAUGAAGUGCACUCAUGAUAUGUUUGCCCCAUCAGCGUGUCACGAGGGCAUUUCAUGAUGCAGGCGGGGUUGGCAGGUGUGCAUCGUGUAGGCUCCACAUGGGACAUGGCAUCCCUUAUGACUCUUGGC*

*>hsa-mir-3620*

*AGGGAAGACCACGAUCCUCUACAAGCUUAAGCUGGGUGAGAUCGUGACCACCAUUCCCACCAUAGGUGAGGUGGGGGCCAGCAGGGAGUGGGCUGGGCUGGGCUGGGCCAAGGUACAAGGCCUCACCCUGCAUCCCGCACCCAGGCUUCAACGUGGAAACCGUGGAGUACAAGAACAUCAGCUUCACUGUGUGGGACGUG*

*>hsa-mir-198*

*GUUUCCUCAGUGAGCUGGUAAGAAUGCACUAACCUUUUGAUUUGAUAAGUUAUAAAUUCUGUGGUUCUGAUCAUUGGUCCAGAGGGGAGAUAGGUUCCUGUGAUUUUUCCUUCUUCUCUAUAGAAUAAAUGAAAUCUUGUUACUAGAACAAGAAAUGUCAGAUGGCCAAAAACAAGAUGACCAGAUUUGAUCUCAGCCUG*

*>hsa-mir-3674*

*UAUAGAUGUAAACGACGACCAGCCAUUACUCUGGAGGUCACAAGAUUUGCAGCUUCUGCAGAUAAACAUCACUAUUGUAGAACCUAAGAUUGGCCGUUUGAGAUGUCCUUUCAAGUUUUUGCAUUUCUGAUGUCCGACUGAUCCUGUGGCCCCACUCAGAAGCGGACUCCCUGGCCUGCUAAACUACCCUUGAAAUACAG*

*>hsa-mir-3663*

*CUCCUCCCUGAUCGCUGAGCUAAGCCACAGGAGAAGGGGAGGAGGGCUUCCUUCCCGGGACCUUGGUCCAGGCGCUGGUCUGCGUGGUGCUCGGGUGGAUAAGUCUGAUCUGAGCACCACACAGGCCGGGCGCCGGGACCAAGGGGGCUCGACAAGAGGAUCUCAGAACAAGCUGAGUCUCUGGAACUAGCUGGGGUGCG*

*>hsa-mir-3672*

*UGUAAUGGUAUGCAUUGAUUUCUUUUUUUAUCUUUUGUGUAUUUACUACACAUUUUUUUCUUUGUGAUUACCAUGAGACUCAUGUAAAACAUCUUAGACUAUUACAAGAUGUUUUAUGAGUCUCAUGAUAAUCACAAAGAUUACUAUUAAAGCUGAUAACUUUGAACAUGUUAAAACAACCUUACACUUCAACUUUUUCU*

*>hsa-mir-3690*

*GACAGGAGGAGACCCUGCCCCACCUCCACCUGGACCCAGUGUAGACAGGAGAAGACUCUGCCCCAUCUCCACCUGGACCCAGCGUAGACAAAGAGGUGUUUCUACUCCAUAUCUACCUGGACCCAGUGUAGAUGGGAGGAGACCCUGCCCCACCUCCACCUGGACCCAGUGUAGACAGGAGAAGACUGCACCACCUCCAC*

*>hsa-mir-373*

*UGGAGGCCAACCUCUUGUAGGCAAGAAAGUCACAGUGAUGGCAGAUCCUCGCGAGGAGCUCAUACUGGGAUACUCAAAAUGGGGGCGCUUUCCUUUUUGUCUGUACUGGGAAGUGCUUCGAUUUUGGGGUGUCCCUGUUUGAGUAGGGCAUCACGAACCAUCCUGCUUCAAGGGAGCCUGCGGGUCUGACUGCAGCUUCA*

*>hsa-mir-374a*

*UUCCUCACCUCUCUUGAUGUUACAGCCAGCAGCAGCCUGGAGCAGCACCCCCUGGGAAGAAAUUUUACAUCGGCCAUUAUAAUACAACCUGAUAAGUGUUAUAGCACUUAUCAGAUUGUAUUGUAAUUGUCUGUGUAUAUGCCUGUCUCUCUUCCUGUUAUCAGUUAGACUGUGAGCUCCUUGAGGGCAAGGAAGGUGUC*

*>hsa-mir-374c*

*CAAAGAUGAGUAAGGCACUGUCCUUGCCCUUAAGGAAUUUAAAGUCUGGUAGACGAGAGCCAUAGACACGGACAAUGAUAAUACAACCUGCUAAGUGCUAGGACACUUAGCAGGUUGUAUUAUAUCCAUCCGAGUAGGAUUUCUUCAGAGGAGCACCACUCCAGGUUGCUGCUGGAUCCAUGAAGUCCUCCCAGGCACCA*

*>hsa-mir-375*

*GAGCUCGCGGAAGACCAGGACCAGGAGAUCACCGAGGGCGACCGCCAGGCCCCGGGCCCUCCGCUCCCGCCCCGCGACGAGCCCCUCGCACAAACCGGACCUGAGCGUUUUGUUCGUUCGGCUCGCGUGAGGCAGGGGCGGCCUCUCAGCACCAGCCCGGGGGCCGGCCUGAUCGCCACGCAGGCACCUGCCGCCGCCAC*

*>hsa-mir-379*

*UGGAAGUGACGCCAACUUCAGGGGCAAGGCCCUGGUUCUGGGGUCAGCACCAUUCCGUGGUUCCUGAAGAGAUGGUAGACUAUGGAACGUAGGCGUUAUGAUUUCUGACCUAUGUAACAUGGUCCACUAACUCUCAGUAUCCAAUCCAUCCUCGGAGGGCUUCCUGGAGGUGUUGCCAACUUCAGGGAAGAGAACACGUG*

*>hsa-mir-3920*

*CAAGCAAGCUUGGUACAUAGAGGAUGAGCAAGCAGAUCAGUGUGGCUGGAGCAGAGAGACUGAGUGAGGGAGUCAGAGAGUUAAGAGAAUUAGUACAGGUGAGAUUGUACUGAUUAUCUUAACUCUCUGACCCCCUCACUCAGUAAAGAUCAGAUUGUGCCAGGCCUUUCAGGUCAUCGAAUCUAGAUCUAGUAUCUAGA*

*>hsa-mir-3924*

*CACACAGAGAGAUACAUGUAUGCGCACAUAUGUGUACAUAAAUAUGUAUUUAAAUUAAAAUAAAUGAAAAAGUAGUAGUCAAAUAUGCAGAUCUAUGUCAUAUAUACAGAUAUGUAUAUGUGACUGCUACUUUUUUGUUUAAUUCUCUUACCCCUAUCUUUCUCUUUUCUAUGUAUGCAGGUAUGUAACAGAUAUGCAAU*

*>hsa-mir-3944*

*UCUCACCCCUGGAACGCCAUUGUCCUUUGUCUCCUUCCCACCUUGCUGUCCACCCAGCAGGCGCAGGUCCUGUGCAGCAGGCCAACCGAGAAGCGCCUGCGUCUCCCAUUUUCGGGCUGGCCUGCUGCUCCGGACCUGUGCCUGAUCUUAAUGCUGCAGGAACACCCAGGAUUUUCACAUCCCCCACCCUGCGUCCAGUU*

*>hsa-mir-620*

*CCUUUCUUUUAUGCUCCUCUUUCCCAAAAUAAGAAAAUCAAGAUAAACUACUUAUAUCUAUAUAUAUCUAUAUCUAGCUCCGUAUAUAUAUAUAUAUAUAUAUAGAUAUCUCCAUAUAUAUGGAGAUAGAUAUAGAAAUAAAACAAGCAAAGAAGUUUUUAGUAUGGAUUGUCAACUUGACAUCACUUUUUCAGUGAUUU*

*>hsa-mir-22*

*UCUUUCCCAUUUUCCCUCCCUUUCCCUUAGGAGCCUGUUCCUCUCACGCCCUCACCUGGCUGAGCCGCAGUAGUUCUUCAGUGGCAAGCUUUAUGUCCUGACCCAGCUAAAGCUGCCAGUUGAAGAACUGUUGCCCUCUGCCCCUGGCUUCGAGGAGGAGGAGGAGCUGCUUUCCCCAUCAUCUGGAAGGUGACAGAAAU*

*>hsa-mir-4269*

*UGGCUCGCUGUGGGUCCGCGGCUCCAUUACGGGGGAGUCUGGGUCAUCUGUGAGCCCAACAGCGCCCUGCAGGCACAGACAGCCCUGGCUUCUGCCUCUUUCUUUGUGGAAGCCACUCUGUCAGGCCUGGGAUGGAGGGGCAAGAGGCUUUCAAAUUUUUCCUUAUUGUUGUAUAUUUUUUAAAAAACUAUUUAUUUUAA*

*>hsa-mir-4270*

*AACACGUCCACCUGAUGGAAAUAUUCUGAACAGGAAUGAGCUGUAAAUAAACUAGGCUCCUAAAAACAAAUAGCUUCAGGGAGUCAGGGGAGGGCAGAAAUAGAUGGCCUUCCCCUGCUGGGAAGAAAGUGGGUCAAGGGGGAAAGGGUGAGGGGGAUGGGGUGGGGGAGCCGAACAAUGCAGAAGCCUCAGCCAGAAAU*

*>hsa-mir-4280*

*AGAACAGACCUGAAAAUGACACAGGAAGUCAAAAUAGAAAGAUCAUUAGGAACUUAAGGUAAGGAAUCAGGGUGGAGUGUAGUUCUGAGCAGAGCCUUAAAGGAUGAGGUAUGUUCAAGACUGAAUGACACCUUUGUGAUGCUAUGGAGUGAAAGCAGUGGAACAUGCUGGUGACCGACGGAAACAGUGCUGGGUGAGGA*

*>hsa-mir-4281*

*GCGGCGGAGAAGACCAAGGAGGGCGUCCUCUACGUCGGUGGGUAAGGGGCGGGGUUUCUGGGGCUGCAGGGCUGGGGGUCCCCCGACAGUGUGGAGCUGGGGCCGGGUCCCGGGGAGGGGGGUUCUGGGCAGGAGAAUAUGAGUCAGCAGAUGGGGCGAGGUCAGCAGGGGUCAUAGGGGACAUAGCCAGCCCAUAGAAG*

*>hsa-mir-4286*

*AUUAAUUUUUCUUUACCUACCUUCUUCAUUCAGUCCCUGUCAAGUAUUAGCUUCUUUUACUUAUGGCACCCCACUCCUGGUACCAUAGUCAUAAGUUAGGAGAUGUUAGAGCUGUGAGUACCAUGACUUAAGUGUGGUGGCUUAAACAUGAAAGGGUUUGUUUUUGUCAUCUGAUGAGAAGUCCAGGCAUAGUAACUGCU*

*>hsa-mir-346*

*UCUGCAGGGCCAACCAGGAAGGGGCGGCGGUGACACAGACGAGCGUGUGGGAGGUCUCUGUGUUGGGCGUCUGUCUGCCCGCAUGCCUGCCUCUCUGUUGCUCUGAAGGAGGCAGGGGCUGGGCCUGCAGCUGCCUGGGCAGAGCGGCUCCUGCAUGGGACAUGGCUCUUCUCUCUGGUUACAGACUCACCACUUCGCUC*

*>hsa-mir-1228*

*GCUGGGUGUGUGAUGGCGCCAAUGACUGUGGGGACUACAGUGAUGAGCGCGACUGCCCAGGUGGGCGGGGGCAGGUGUGUGGUGGGUGGUGGCCUGCGGUGAGCAGGGCCCUCACACCUGCCUCGCCCCCCAGGUGUGAAACGCCCCAGAUGCCCUCUGAAUUACUUCGCCUGCCCUAGUGGGCGCUGCAUCCCCAUGAG*

*>hsa-mir-429*

*CCUUCCCUUCACAGGCCCCGCAGACACCAGCCCAGGACCCGGAGGCCACCCACACCACCGCCGGCCGAUGGGCGUCUUACCAGACAUGGUUAGACCUGGCCCUCUGUCUAAUACUGUCUGGUAAAACCGUCCAUCCGCUGCCUGAUCACCGUUAGAGGAGAGAGCUGCCUGCCCUGCAGCUCAUCAGUGCAAAGCCGCCC*

*>hsa-mir-4312*

*GUGCUAGGGUAAUCUGCAGUCCCUUCCGUUGGUAGCCUGGCCUGGCCCUCUCAGGAUGUUUGAGAAAGGUUGGGGGCACAGAGAGCAAGGAGCCUUCCCCAGAGGAGUCAGGCCUUGUUCCUGUCCCCAUUCCUCAGAGUUUAUCCUCUGAGCACACACCUCUGGUGUGACUCAGUUGGUUCUUCCUCCAAAGUGAAGGA*

*>hsa-mir-4323*

*GUCCAGGCGUCUGUGCAUGCACCUGUGGUUUAGGGGGCCCCCCAGCCACGGGGCGCAGGAUGUCCUGCGGGGCCCAGGCGGGCAUGUGGGGUGUCUGGAGACGCCAGGCAGCCCCACAGCCUCAGACCUCGGGCACCAAGAGGAAGAGGGCAGCGGACUCGGGCGCGCCUUUGUCUGAGCUGCGGUGCGCCGGGAGUCCU*

*>hsa-mir-485*

*CCGAGAAGGGCGGCCACCUCUGCCAGAGAGGCAAUGGAUUUCUCACCAGUGUUCAUGUGUGGUACUUGGAGAGAGGCUGGCCGUGAUGAAUUCGAUUCAUCAAAGCGAGUCAUACACGGCUCUCCUCUCUUUUAGUGUCAAAUUCUGCCUCGGAGGGCUUCCUGAGCCAGUGCUGCUUCCAAGGUAGGUUUCUGGAUGGC*

*>hsa-mir-487b*

*CGCCUUGAAGACGUACCAAGUCCACCCAAGGCAGUGGCUUUCUUUUCCGUGCUAACCUUUGGUACUUGGAGAGUGGUUAUCCCUGUCCUGUUCGUUUUGCUCAUGUCGAAUCGUACAGGGUCAUCCACUUUUUCAGUAUCAAGAGCGCAGACUCUGGAGCUCGAUUUCCUGUGUUUGGAUCCCACCUCUGACAUUUACUA*

*>hsa-mir-488*

*GACCACAACUGGUGUUUCCUCCUAAGGAAGAAAGAAGAGCCUAGAGUGGAAAAUUUCAGAGAAUCAUCUCUCCCAGAUAAUGGCACUCUCAAACAAGUUUCCAAAUUGUUUGAAAGGCUAUUUCUUGGUCAGAUGACUCUCAAUUUCUUCUGGAAGACUCAGGAGUCAUAUUUUGCUGUUCAGACAAACAAAGUUAAUAA*

*>hsa-mir-489*

*CAAAGUAUUUGUCGACUGAAAAAAACUGAUGAUGUUUGGGCUGCUGACUACUGCUGUUGGUGGCAGCUUGGUGGUCGUAUGUGUGACGCCAUUUACUUGAACCUUUAGGAGUGACAUCACAUAUACGGCAGCUAAACUGCUACAUGGGACAACAAUUAAGAGUUUUGUUUGUUUGUAUGUUGAAAAAUCAUGUAAGAUGG*

*>hsa-mir-491*

*ACAUACAGCACCUUGGAUACUUUCAACAAUUAACUUCGGAGAUGUCUGCUGUGGGAAUUGACUUAGCUGGGUAGUGGGGAACCCUUCCAUGAGGAGUAGAACACUCCUUAUGCAAGAUUCCCUUCUACCUGGCUGGGUUGGAGUCAUAUCCUGCAGGUCCUUACCAUGAAGAAGAAGAUGAAGGCUUGGAGGAUACUAUU*

*>hsa-mir-500a*

*ACCUUGUGUGAAGAAGAAUGAGCCCUCUCACUGAACAUUCACAGGGCUUUGUGUUCUGCUCCCCCUCUCUAAUCCUUGCUACCUGGGUGAGAGUGCUGUCUGAAUGCAAUGCACCUGGGCAAGGAUUCUGAGAGCGAGAGCUUCAUCUUCGUGUAGGACAGAGCGUCUUUCUGUCCUCCUAUCAUCCAUUCCACAAGUAU*

*>hsa-mir-500b*

*AAGCCUUCUUGUUAGAUGCACAUGUGAGGUGUACAGGUACUGGAAUCUCCUGUCCUGGUUCCCCCUCUCUAAUCCUUGCUACCUGGGUGAGAGUGCUUUCUGAAUGCAGUGCACCCAGGCAAGGAUUCUGCAAGGGGGAGUGACAUCUUCGCAUAGGACAGAGCACUUCUACCUCCUCCUUCCAUCCUUUCUAUGACUAU*

*>hsa-mir-513b*

*UUGAAUAAAAAUGGCAUGAAUAGGGAGCAUUUGGUCUGGGAUGCCACAUUCAGCCAUUCAGUGUACAGUGCCUUUCACAAGGAGGUGUCAUUUAUGUGAACUAAAAUAUAAAUGUCACCUUUUUGAGAGGAGUAAUGUACAGCAUGCACUGCAUAUGUGGUGUCCCUAGGGAUGGGGCUGUAGCUGCGAGCAUGGUGAUG*

*>hsa-mir-517a*

*CACCACCGCAUCCAGCCUGGGCGGCAGAGUGAGACUCCGUGUCAAAAAAAAGAAGAUCUCAGGCAGUGACCCUCUAGAUGGAAGCACUGUCUGUUGUAUAAAAGAAAAGAUCGUGCAUCCCUUUAGAGUGUUACUGUUUGAGACAAGCAACGUUGAAGAUGCUGCUGAUCUUGGUAAUACAUUUGCAGAGCGUGCUUAUC*

*>hsa-mir-520f*

*UUAAACCUGGUCAAGGAAGAUUCCCACAAAAAAUCCACGGUGCUGGAGCAAGAGGAUCUCAGGCUGUGACCCUCUAAAGGGAAGCGCUUUCUGUGGUCAGAAAGAAAAGCAAGUGCUUCCUUUUAGAGGGUUACCGUUUGGGAAAAGCAAUGUUGAAGUUGAUGCUGAUCUUGGUAAAAUAUUUGCAGAGCGUGCUUAUC*

*>hsa-mir-527*

*GUUUUGACCUGGUCAAGGAAAAUUUCAACAAGAAACCCAGAGUGCCGGAGCAAGAAGAUCUCAAGCUGUGACUGCAAAGGGAAGCCCUUUCUGUUGUCUAAAAGAAAAGAAAGUGCUUCCCUUUGGUGAAUUACGGUUUGAGAAAAGCAACGUUGAAGUUGAUGCUGAUCUCGGUAAUACAUUUGCAGAGCAUGCUUAUC*

*>hsa-mir-545*

*UUAGACUGUGAGCUCCUUGAGGGCAAGGAAGGUGUCUUCAUCUCCAGAGCCCAGCCUGGCACAUUAGUAGGCCUCAGUAAAUGUUUAUUAGAUGAAUAAAUGAAUGACUCAUCAGCAAACAUUUAUUGUGUGCCUGCUAAAGUGAGCUCCACAGGCUCUGGAUUGUCAGCUGUUUCUUCAAAAAGCAUGUGGUCAGAAAU*

*>hsa-mir-548c*

*AUAGGCUUACUGUCUUUUGUUGAAAAAUUGCGAUGAGAAAGGAAUCGGCAUUGGCAUCUAUUAGGUUGGUGCAAAAGUAAUUGCGGUUUUUGCCAUUACUUUCAGUAGCAAAAAUCUCAAUUACUUUUGCACCAACUUAAUACUUUCAGGCUUGUUGAAGCCUAAGUAGAAAAUUGUUGCUUAAUCUCUUUUAUUCUGAU*

*>hsa-mir-548n*

*AGGAAAGGAGGCUGGUUGGAAGCCUUUAAUCUUGAGUCUUAUCCCACAGCAUGACUGAUGAUAUAGGUUGGUGCAAAAGUAAUUGUGGAUUUUGUCGUUAAAAAUAGCAAAACCCGCAAUUACUUUUGCACCAACCUAAUAGAAAGUGUCACCCAGGGCAGGUUUUCCAAGUGUGGGGUUCCUGCUGGUCACACAGGACC*

*>hsa-mir-548x*

*AGAUACUAUCAUAACAACAUUAAGCCUUCCAAUCUGUAAUCACAUUAUACAUUGCAAUAUAUUAGGUUAGUGCAAAAGUAAUUGCAGUUUUUGCGUUACUUUCAAUCGUAAAAACUGCAAUUACUUUCACACCAAUCUUAAUAUUUCGAUCUUGUUUUAUUUUUCUCAGCAAAGUUUUGAAGAUUUCAGUAAACAGGUAU*

*>hsa-mir-557*

*AGUGGUGAUCAGAAAGGCAGGGCCAGAUCAAGGGUGUGCAGACAGAAUGGGCAAAUGAACAGUAAAUUUGGAGGCCUGGGGCCCUCCCUGCUGCUGGAGAAGUGUUUGCACGGGUGGGCCUUGUCUUUGAAAGGAGGUGGAGAAAGGAAGCUCCUUCUAUGAGGCACUUAGAUUCAUUUCUAUCUUCCUAGCCUGAGCAC*

*>hsa-mir-564*

*CAGGAGCGUCGCGUCACGUGAGGCUGCCAUCCAAUCGCGGCGCGCGCUCUGCCCUGCGGGCAGCGGGUGCCAGGCACGGUGUCAGCAGGCAACAUGGCCGAGAGGCCGGGGCCUCCGGGCGGCGCCGUGUCCGCGACCGCGUACCCUGACACCCCCGCGGAAUUCCCUCCGCACCUCCAGGCGGGUGCGAUGCGGCGCCG*

*>hsa-mir-581*

*CGAAAAUAUAUAGGGAAGGAGUAUAUAGGGAAGGAGUAAGUAAGGUUUUCCAAAAAGUUAUGUGAAGGUAUUCUUGUGUUCUCUAGAUCAGUGCUUUUAGAAAAUUUGUGUGAUCUAAAGAACACAAAGAAUACCUACACAGAACCACCUGCAGACUGGUGCCAGUCCACAAAAUAUCUGUUUGAGUAUGCAAGAAGAUA*

*>hsa-mir-593*

*AUUGUUUGUUCCUACAUACCCCUGGCUAUGCUGGGCAGGAUGAUUUGUAGCCCCCAGAAUCUGUCAGGCACCAGCCAGGCAUUGCUCAGCCCGUUUCCCUCUGGGGGAGCAAGGAGUGGUGCUGGGUUUGUCUCUGCUGGGGUUUCUCCUUCUCUAGAGGUUAACAUAGCCCUGAGUGAUAGCCAGUUUCCAUGGAAACA*

*>hsa-mir-596*

*UUCAGUGAGUUCUGAGGAACGCAUAGCAGGUGGAUCCGUCGCCACAGUCGGGAAAGCGAGCACGGCCUCUCCGAAGCCUGCCCGGCUCCUCGGGAACCUGCCUCCCGCAUGGCAGCUGCUGCCCUUCGGAGGCCGACCCGACCCAUCCCUGGGUGCUGUCUAGGUCACUGUCCUUGUGUGUUUUUGUUUUACUUUAGAAC*

*>hsa-mir-608*

*GAGACUCUAUCUCUAAUAAAAAAUAAUUAAAAAAAAAUUCCCAAGAUCCACUGGGCCAAGGUGGGCCAGGGGUGGUGUUGGGACAGCUCCGUUUAAAAAGGCAUCUCCAAGAGCUUCCAUCAAAGGCUGCCUCUUGGUGCAGCACAGGUAGAAAAUGGGGCUGGGGAGGCAGAUGGAACCAGGUCAUGGAAGGCCACAGA*

*>hsa-mir-610*

*AUUAUUCUCUGUGAAUAAGGUCUUACAUAUUAGCCCUUCACUCCCAACUAUUUGUCUAUUUGUCUUAGGUGAGCUAAAUGUGUGCUGGGACACAUUUGAGCCAAAUGUCCCAGCACACAUUUAGCUCACAUAAGAAAAAUGGACUCUAGUUGGGAGUGAGGGGCUAAUAAACACCAGAUCCCAAGAAAAUUCCCCAUAAU*

*>hsa-mir-616*

*AUGGAGAAUGUUCAGGAUGGAGGUCAGAGUGUUACAUCAGGUGGUCAGGAAUUACCUUAGGUAAUUCCUCCACUCAAAACCCUUCAGUGACUUCCAUGACAUGAAAUAGGAAGUCAUUGGAGGGUUUGAGCAGAGGAAUGACCUGUUUUAAAAGGCUCACUCAGGCUGCUGUAUGGUGAAUAGAGUUGCGGAGGGGUGGC*

*>hsa-mir-618*

*CGGUUGAUGAGAGGAGGUGCUAAUCGGACUAAGAUUUAUUUUUAAAGUUUGCUCUUGUUCACAGCCAAACUCUACUUGUCCUUCUGAGUGUAAUUACGUACAUGCAGUAGCUCAGGAGACAAGCAGGUUUACCCUGUGGAUGAGUCUGAAAAAAUUCAUCAGCAGCUCAUGGAAAAUCUAUAACCAAGAGAUGAUGGGUU*

*>hsa-mir-627*

*UUGCCCUACCUGCAUUUGGGAGGUGAGGAUGAAUUGCUGGUUUACUUAUUACUGAAUUACUUAUUACUGGUAGUGAGUCUCUAAGAAAAGAGGAGGUGGUUGUUUUCCUCCUCUUUUCUUUGAGACUCACUACCAAUAAUAAGAAAUACUACUAAUGGGCAUAAGCUUCCUGUAUAUAUGGUGUGGUGGUAGGAAAAAAA*

*>hsa-mir-639*

*GGUUCGGACCGGCCCCAAGGAGCAGGGGCGAACGUGGGCGCCUCGUGCCCUGAUUGGCCGACGGGGCGCGCGCGGCCUGGAGGGGCGGGGCGGACGCAGAGCCGCGUUUAGUCUAUCGCUGCGGUUGCGAGCGCUGUAGGGAGCCUGUGCUGUGCCGCGCAGUUAGGCAGCAGCAGCCGCGGAGCAGUAGCCGCCGUGGG*

*>hsa-mir-718*

*GCUCGCGGAACCCGCCGGGACCCGGGCCCUCCCCGGCGCGGGGCGCCCCCGUGUGACCCAGCGCGCGGCCGCGGCGCGCAAGAUGGCGGCGGGCCCGGGCACCGCCCCUUCCGCCCCGCCGGGCGUCGCACGAGGCCGGCUCGAAGGGGAAGUGAGUCAGUGUCCGCGGACCCGGCCGGCCCAGGCCCGCGCCCGCCGCG*

*>hsa-mir-1193*

*UGGCCUUCUGGACACGCUAGCAACGUCAUUGAAGGGACGAUGGUGCCAUCUGCUCUCCAGGGUAGCUGAGGGGAUGGUAGACCGGUGACGUGCACUUCAUUUACGAUGUAGGUCACCCGUUUGACUAUCCACCAGCGCCCGGUCCAUCUGUGGGAUGACAUCUUGGUGCACAUGGCAACUUCGGAGAAGAUGUGGGAGGA*

*>hsa-mir-761*

*CUUUUUUUCUGUCUGUUUCUGCUUUUUUUCCUUAGGUCUGCACCUGUUCAGCAUUUGGCAGGAUGGCAAGCGGAGGAGCAGCAGGGUGAAACUGACACAGUUCUGGUGAGUUUCACUUUGCUGCUCCUCCUGAUUCUCAGGGAAAGAAUUUUGUUACUCCUAUUGGAAAACAUCCAUCUCCAAAUGAAUCCUUAAGAUAA*

*>hsa-mir-876*

*UUUGGUGGCCAAAGGGUACUGUUUUUGUUGGUUCAUAGGUACCAGGCUUUACACAAACUGUGAAGUGCUGUGGAUUUCUUUGUGAAUCACCAUAUCUAAGCUAAUGUGGUGGUGGUUUACAAAGUAAUUCAUAGUGCUUCACAGGUGCUUCUGCAGUUGAUCUACACCAUGAAGCCUAAGAUUCCUUUACUGAGCCAUUG*

*>hsa-mir-889*

*UCUGGAAGAGACACCAACUUUGGAGAAGAUGCCCAGCUCUUCUUGAUGGCACAGUUCUUUGGUGCUUAAAGAAUGGCUGUCCGUAGUAUGGUCUCUAUAUUUAUGAUGAUUAAUAUCGGACAACCAUUGUUUUAGUAUCCAAAUUUGCUUCAGAAGUUUUUGAAAGCACAGUGCUGACUUCAGUGAGGGGACAUUGGUGC*

*>hsa-mir-920*

*AUCUGAUCACACUGAAGGACAAAGACAUUUAUCUCUCUGUAUAAUGAAAACUGAAAAAGCCACUGGUAGUUGUUCUACAGAAGACCUGGAUGUGUAGGAGCUAAGACACACUCCAGGGGAGCUGUGGAAGCAGUAACACGGGAGGGGUUACAAGGAAGGAAGGAUUACAAGGAAGGAGGGGACCACCAAGUCUUCACUCA*

*>hsa-mir-938*

*GUCCCACAGCAGAGAAGGGAGGGUGAGACAAGGCAGGUUAAGCAGGUACGGCAUGUUGGAAGGUGUACCAUGUGCCCUUAAAGGUGAACCCAGUGCACCUUCAUGAACCGUGGUACACCUUUAAGAACUUGGUAUGCCUUCAUGAACACUUAAAGUACCAGGGGGCACUUCAUGAAACAUCAUUAAGUUUCUCCUAAGUC*

*>hsa-mir-944*

*ACCUUAAUUUUUUUCAACCAAGAUAAACAAUUUUUCUGCAGUAAAUUUGUAUUUAUGUUCCAGACACAUCUCAUCUGAUAUACAAUAUUUUCUUAAAUUGUAUAAAGAGAAAUUAUUGUACAUCGGAUGAGCUGUGUCUGGGAUUAAGUAUUAGCUCCACUGUAACCUUCAUUUUCUUGAACACUGGAAAGAAAGGCCAA*

*>hsa-mir-34c*

*UGAAUUGCCUGCCUGUCACAACGUGUUGGGGUACCAACUUGAGACUGCAAUUUUUUCUAUGAGUCUAGUUACUAGGCAGUGUAGUUAGCUGAUUGCUAAUAGUACCAAUCACUAACCACACGGCCAGGUAAAAAGAUUUGGGAAUUCGUCCAAAUGAGCUGCCUGUGCAUCAUCAAUGUGCGUGGGGAAGAGGGGUGUUG*

*>hsa-mir-147b*

*AUGUCCAAAGGGUGACCAAAUGACGAGCCCUCGCCUCUUUCUUCUGAAGAGUACUCUAUAAAUCUAGUGGAAACAUUUCUGCACAAACUAGAUUCUGGACACCAGUGUGCGGAAAUGCUUCUGCUACAUUUUUAGGGUUUGUCUACAUUUUUUGGGCUCUGGAUAAGGAAUUAAAGGAGUGCAGCAAUAACUGCACUGUC*

*>hsa-mir-193a*

*GCCGGCGACCGGACCCAGCCCCGGGAAGCCCGUCGGGGACGCACCCCGAACUCCGAGGAUGGGAGCUGAGGGCUGGGUCUUUGCGGGCGAGAUGAGGGUGUCGGAUCAACUGGCCUACAAAGUCCCAGUUCUCGGCCCCCGGGACCAGCGUCUUCUCCCCGGUCCUCGCCCCAGGCCGGCUUCCUCCCGGGCUGGCGUGC*

*>hsa-mir-323b*

*CCCAAGCUGCCCAGGCUGUGCAGAAGAUGCAGGAAUGCUGCGAGCAGUGCCACCUCAUGGUACUCGGAGGGAGGUUGUCCGUGGUGAGUUCGCAUUAUUUAAUGAUGCCCAAUACACGGUCGACCUCUUUUCGGUAUCAGAUCUCACCAGGUGUCUUCUUGGACCUGAUGCCGAUUUAGGGGACACCAUACUCAUGUUGG*

*>hsa-mir-380*

*CCUGGAGGUGAUGCUAGUGAGAGGAAAGAGACACCGGCUCUGACCUCAGCCCUCUCCAAGGUACCUGAAAAGAUGGUUGACCAUAGAACAUGCGCUAUCUCUGUGUCGUAUGUAAUAUGGUCCACAUCUUCUCAAUAUCAAAUUCAGUCAUAGAGGGCUUCCCAGAGAGAGGCAAAGUCACCUUCGUGGGAGACACACUG*

*>hsa-mir-410*

*GGGGGCAGCGCUGGCACCACGGGACGCGGCAGCCACGUUCUUGAGCCGAUGGCACUCUGGGUACCUGAGAAGAGGUUGUCUGUGAUGAGUUCGCUUUUAUUAAUGACGAAUAUAACACAGAUGGCCUGUUUUCAGUACCGCUACCGCCCGGUGGUGUGCGGGCGCCACGCCUGAGGCGGGACUUUCCAGGGUACGUGAAU*

*>hsa-mir-455*

*GCAGGUCCUGGAGCCCUGGCGUGGGGCGGGCCUCCUGCCGGCGAGCGCCUGCGCCCUUCCCUGGCGUGAGGGUAUGUGCCUUUGGACUACAUCGUGGAAGCCAGCACCAUGCAGUCCAUGGGCAUAUACACUUGCCUCAAGGCCUAUGUCAUCGAGGAGCCACCGGAGCUGCCACUGCCACCAGGGAGGAAGAGGAGGAG*

*>hsa-mir-517b*

*AAAAAGAAACAAAAACAAAAACAAAAAAAAGAACCCUACAGUACUGGAGCAAGAAGACCUCAUGCUGUGACCCUCUAGAUGGAAGCACUGUCUGUUGUCUAAGAAAAGAUCGUGCAUCCCUUUAGAGUGUUACUGUUUGAGAAAAGCAACGUUGAAGAUGCUGCUGAUCUUGGUAAUACAUUUGCAGAGCGUGCUUAUCA*

*>hsa-mir-539*

*AAGCAGUCACCAUCUAACCUUGAGCCAAAGACCUGGAGGUGGUGGUCCGUGCUGCUGUUUCAUACUUGAGGAGAAAUUAUCCUUGGUGUGUUCGCUUUAUUUAUGAUGAAUCAUACAAGGACAAUUUCUUUUUGAGUAUCAAAUCUUGCCUCGGCAGACUUCCUGGACGCCAUGACAAGUUCUGUAAAGGGAUGCACGCA*

*>hsa-mir-563*

*UCUGUAACUGUAACAAUGUUGUUUAACACAACUUUGAAUACAUUUUUACAGGAAGCAAAGAAGUGUGUUGCCCUCUAGGAAAUGUGUGUUGCUCUGAUGUAAUUAGGUUGACAUACGUUUCCCUGGUAGCCAAUAUAAGGAAUGGGUCCACUAACCCUUGAUAUCAGAAAAUUCUGGGGUUGGAUUAGAUGUCAAGGUUG*

*>hsa-mir-597*

*AGUUAAUUUAUAAACUAAGUAUUUUACCUUUUGAUCCCCAUCAGUUAAGAUGUUACUUACUCUACGUGUGUGUCACUCGAUGACCACUGUGAAGACAGUAAAAUGUACAGUGGUUCUCUUGUGGCUCAAGCGUAAUGUAGAGUACUGGUCGACCUUGUCUAACUUCACGUUCCCUUGAGGCUGACUUGCUGUCACCUGAA*

*>hsa-mir-656*

*GGGUGGCAGGGCCCUUCCCUGUGGAAGAAGCCUCCUCCCUGGAGUCAGUGCUGCUCAGUGGUACCUGAAAUAGGUUGCCUGUGAGGUGUUCACUUUCUAUAUGAUGAAUAUUAUACAGUCAACCUCUUUCCGAUAUCGAAUCCCACCUUGGAUGCCUUCCUCUUUGCUGUCAACUCCAGGCAGCAGCUGGCUCUGCGCUG*

*>hsa-mir-769*

*CAGGAGGACCCCCAGGACCCACUGUUGGUGACAUCCCGGCCGCCUUGGUGCUGAUUCCUGGGCUCUGACCUGAGACCUCUGGGUUCUGAGCUGUGAUGUUGCUCUCGAGCUGGGAUCUCCGGGGUCUUGGUUCAGGGCCGGGGCCUCUGGGUUCCAAGCACCAGUGAGGAGAGUGCUGGGAGGGGAAGGGGUGGGAGGGC*

*>hsa-mir-1912*

*AACAUUUAAAAUUUGAUCAAAUGAUACCACCCUUUGAGCCUUCUAUCACAACUGGACUUGGCUCUAGGAUGUGCUCAUUGCAUGGGCUGUGUAUAGUAUUAUUCAAUACCCAGAGCAUGCAGUGUGAACAUAAUAGAGAUUGAAAGACAGCCAGAAGGGUAAAACAUGAAUUCUUAAAGAGUAAUUUUGUACUUCAUGUA*

*>hsa-mir-3159*

*UGUAAGCAUGACUUACUGCAGCCUCAAUCUCCUGGGCUCAAGCAAUCCUCCUGCUUCAGGCUCCCAAAGUCCUAGGAUUACAAGUGUCGGCCACGGGCUGGGCACAGUGGCUCACGCCUGUAAUCCCAGCAUUUUGGGAGACAGAUCUGGGUGGAUCACGAGGUCAGAAGAUCGAGACCAUCCUGGCCAACAUGGUGAAA*

*>hsa-mir-3197*

*CAGCGGACGGGAAACGCCUCGAAAGCAGCCAGACCCGGCGACUGAAAUGAGGCGGAGGAGCUUGGCGAGGGGAGGCGCAGGCUCGGAAAGGCGCGCGAGGCUCCAGGCUCCUUCCCGAUCCACCGCUCUCCUCGCUGACCUCCGAGUCACCCCCGGAAGCUCCCGCCACUGCCGGGCGAAUAGACCCCCGCGGACCCCCA*

*>hsa-mir-4273*

*UAACCUGAGGUUUGACUUAUUGAUAAAGGGUUUUCCACACUCAUUACAUUCAUACGGUUUUUCCCCUGUGUGUGUUCUCUGAUGGACAGUAAGCCUUGACUUAUGGCUAAAUGCUUCUUCACAAUGGUCACAUGCAUAGGGCUUUUCCCCUGUGUGAGUUCUAUGAUGUAUUGUGAGGUAUGACUUCUGGCUAAAGGUUU*

*>hsa-mir-139*

*GCCAACAGUGUGGGGUCAGGCUCCUGUGGAUGGGGACACCCUUGGGAGGCACUGGGACUGGCUCAGGUGUAUUCUACAGUGCACGUGUCUCCAGUGUGGCUCGGAGGCUGGAGACGCGGCCCUGUUGGAGUAACAACUGAAGCCGGAGUCUGCGAAGGGUGGGCAGGAGGGUGGAGGGAUGGGGGCAUGGAGCGGGAGGG*

*>hsa-mir-148a*

*GCUGGGGGGUGGGGGAACACACCUGCAGGAAGAAACUUCUGGAGGAAGACAGCACGUUUGGUCUUUUGAGGCAAAGUUCUGAGACACUCCGACUCUGAGUAUGAUAGAAGUCAGUGCACUACAGAACUUUGUCUCUAGAGGCUGUGGUCGCCGAUGCCGCCAGUGCUCCAGACGCCAGGCCGAGUGGCCCUGGUGGCCAC*

*>hsa-mir-224*

*CAGCUUUUCCCGGAUAGGUGGGGACCCAUCAUCAAAAGUGACAGAGAAGAUAAGGCCCAGGGGCUUUCAAGUCACUAGUGGUUCCGUUUAGUAGAUGAUUGUGCAUUGUUUCAAAAUGGUGCCCUAGUGACUACAAAGCCCCAGAGCCAGCAUCAUCAUCAAAGCAAUGACAGUAGGUAAGCACCAGACCUCCUUGGGAG*

*>hsa-mir-424*

*UUCAGUCAUCCAGUCUUUAUUCACCCGCAGGUACCCCCAGAUCGAUCCCCCUUCAUUGACUCCGAGGGGAUACAGCAGCAAUUCAUGUUUUGAAGUGUUCUAAAUGGUUCAAAACGUGAGGCGCUGCUAUACCCCCUCGUGGGGAAGGUAGAAGGUGGGGUCUGCCGGACGCGUGUUCCUGCCACCAGGUGCCCGCUCCC*

*>hsa-mir-505*

*UUGAAAGCACUUACUGUUUGUCACUUGAAGCAGAAGAGCAGCCUGCCGGUGGUAAAUUGAUGCACCCAGUGGGGGAGCCAGGAAGUAUUGAUGUUUCUGCCAGUUUAGCGUCAACACUUGCUGGUUUCCUCUCUGGAGCAUCGCCAAGUUCGUGGAUUUUCAUUUUUCCUGACUUUUGGACUUUUGUUACUGCUCCCAUU*

*>hsa-mir-549*

*GUGUGUUUCUAAGUUGACUGUUGAGAACCUGGCAUCACAUCUUCUGAUAGACAUGCAACUCAAGAAUAUAUUGAGAGCUCAUCCAUAGUUGUCACUGUCUCAAAUCAGUGACAACUAUGGAUGAGCUCUUAAUAUAUCCCAGGCACUGUGCUAGGCAUUUUCCAUGCAUCUUUGUAUGUAAUCCCCGCCAGGAAUUAGUA*

*>hsa-mir-873*

*CUUGAAAUGGAGGAAAUAUGAACAGCUGCUUAUCUGCAAAUGGUUCCUGCUUAGAAUAAGUUUGUGUGCAUUUGCAGGAACUUGUGAGUCUCCUAUUGAAAAUGAACAGGAGACUGAUGAGUUCCCGGGAACACCCACAAAUCUUCCUACUCAUUUCUACUGCUUUGCAAUGGACAACAUUUUAUGGCUGGUACCUUCCA*

*>hsa-mir-875*

*ACUGCAAUUCUGUAACUCUAUGCAUGUUACUUUUUUUCCCUAGUUUUUGGAAAAUUCUUAAAGUUUAGUGGUACUAUACCUCAGUUUUAUCAGGUGUUCUUAAAAUCACCUGGAAACACUGAGGUUGUGUCUCACUGAACAUAAGCUUCGUGACUUCUAUUCAGAACUUUAAUGACUGGGAAUACGUUUAAUGCCAAAGA*

*>hsa-mir-1234*

*CCCCCGUAUGGCGUGGCUCUCAACAGCCUCACCACAGGAACCACGGCUUUCCCGCUUCGUGAGUGUGGGGUGGCUGGGGGGGGGGGGGGGGGGCCGGGGACGGCUUGGGCCUGCCUAGUCGGCCUGACCACCCACCCCACAGGCACCCAGGAGGGUGUGCGGAUCACCCUGGACUGCGCCCAGGCCACCUUCAUCUCCUA*

*>hsa-mir-1279*

*CUAAAAAAAGAACAUUGGUUAAGACAAACCUAAAUCAAUCUGAACAGUGUUGCCUGAAUAUUUAGAAUAUUCACAAAAAUUCAUAUUGCUUCUUUCUAAUGCCAAGAAAGAAGAGUAUAAGAACUUCCUCCCAGAAGCCUAGACAUUUUAAUACAUUUUCUAUUUCAACUUACAAUGAAACUUUAAUCCUGAAUCUUGAU*

*>hsa-mir-1537*

*UCUUGAGAAGUUUGUGGGGGGGCAAGAAGUUUGUGGUGCUUGGAGGCGUGCCUGGGAGUUGGCACUAAGUACAGCUGUAAUUAGUCAGUUUUCUGUCCUGUCCACACAGAAAACCGUCUAGUUACAGUUGUAAGUUGUGCCAGACCUAAUCGCUAAAAUGCUCUGAAAGGUAGAAUUCUGAAAGAUAUAGUUUGGUUGUG*

*>hsa-mir-3157*

*UACAACUUCUCAAUGAGUCUGCCCUCACUGUCCAACAAUUGAGCUGAGAAUAUAAGAAGGGAAGGGCUUCAGCCAGGCUAGUGCAGUCUGCUUUGUGCCAACACUGGGGUGAUGACUGCCCUAGUCUAGCUGAAGCUUUUCCCUUCUUUCUACACCCAGCUCAAGUCCCAGGUCCAUAAAACCUUUAGAAACUCUUCAGA*

*>hsa-mir-3191*

*ACAUGGAAAAGGCAAAAUGUGGCCCCUUUGGAAAAUAGAGGUCGUUAAGCCUGACCACCCCCGGGGUCACCUCUCUGGCCGUCUACCUUCCACACUGACAAGGGCCGUGGGGACGUAGCUGGCCAGACAGGUGACCCCAGAGCGGCGUCCCCAGGUACUUCUUGCUUUCUGGUGCCUGUGGAGGAGAAGCUGAGAAAUUG*

*>hsa-mir-3617*

*ACAAGAGUACUUAUUUUACUCAUGAGAAAAAGAAAGUAAAAAAGAGGGAAUUGUCUUACUCAAGGUCAUAGAAAGACAUAGUUGCAAGAUGGGAUUAGAAACCAUAUGUCUCAUCAGCACCCUAUGUCCUUUCUCUGCCCUGGGAGCACACUCCAUUUAGCAGGCUUGCGGCUAGUGUUAAUGUCUGGACAAUUAAUCAA*

*>hsa-mir-3917*

*CCGCCGCUCUCGGCCAAUGCGGAGCCCCGCGCGGAGGUCACGUGCCUCUGUUUGGCGCUUUUGUGCGCGCCCGGGUCUGUUGGUGCUCAGAGUGUGGUCAGGCGGCUCGGACUGAGCAGGUGGGUGCGGGGCUCGGAGGAGGCGGCGGCUGGCUGAGGCCAGCAAGAGGGACGCGGUCGGCGGGAGGGGCUGGGCCGUGG*

*>hsa-mir-4675:10:20840837:20841038*

*GAAGAUUAAAAAGUCAGCUUUAUAACUGAUAAAAUCUAGGCUGAAUUGGACAGACUCACUCCAUGAGAAAUCCUGCUGGUCAACCAUAGCCCUGGUCAGACUCUCCGGGGCUGUGAUUGACCAGCAGGACUUCUCAUGCACUGAGUCUUAGAGCCCUCUCCAAGCAAAAGGAGAGUAGAAGCGACUUACUCAACCAUGGC*

*>hsa-mir-4530*

*CAGCACAUUCCGUCCUCCCCACCCGGCCCCUCCCGCCUCCCGCGGCCCCGUGGGGACGGAAACAUCCCGUCCCCGACCGCACCCGCCCGAAGCUGGGUCAAGGAGCCCAGCAGGACGGGAGCGCGGCGCCCCCGACGCCCGGGAACUGGCUCCCCCGGUUCCCAUCUGGUGACCUCACCUGGUCCCACCCUUUCAGGUGA*

*>hsa-mir-548ax*

*UUCAGAAUUUCUUCAUUUGUGUUUUAUCCUUCCAUUUUUCUUUCUAAGCAUAUAUAAUUUAUUUAGAUUGGUGCAGAAGUAAUUGCGGUUUUGCCAUGGAAAGUAAUGGCAAAAACCGUAAUUACUUUUGUACCAACCUAAUAAUUUCAGCCAACUAUUCAUAUGCAGCACUGUUUACAAUGGGGAUGAAUAAGUAACAG*

*>hsa-mir-4643:6:92231316:92231517*

*CUCAUCCUUUCAGAUUCAGCACAAGCAUGUUCAUUACGUUGUAUUGUGCUCAUUAAAGUAUGUGUGCCCUAGCAUUUAUAAUCAUGUGUUCAUUCACAUGAUCAUAAGUGGACACAUGACCAUAAAUGCUAAAGCACACUUACUUCUACAACAUACUGAGCUCACCUCACAGGUAUAAACAAAAACAUGCACACAAAUGU*

*>hsa-mir-4753*

*CCCUGCUAGGAAUCUUGAAUCUUUGUUUUUCGUAUCCAGAAAUUUAACUCUGAAAUAUAUCUACACAAGGCCAAAGGAAGAGAACAGAUAUAUCCACAGUACACUUGGCUGUUCUCUUUCUUUAGCCUUGUGUAGAUAUAUUUUCAUCCCCUGUGAUGCCUACUUGCUAGACUCUUUCAUUCUAGCAACUUCUGUCUUUC*

*>hsa-mir-4499*

*UGGGCCACUGUGGCAGACUGGGGGUCACCAUCACUCAGUGAAAGUGUGAGCAGGAAAAAUCGCAGAGAAGACUGAGAGGAGGGAACUGGUGAGUUGUACAUAGAAAUGCUUUCUAACUCCUUGUCUCAGUCUGUUUUUGCUACCAGUGAGGCUUACAAUGUUUGAAAGACCCCAAUCCCAGCAUGCCUGCUUUAUAUAAA*

*>hsa-mir-4695*

*GGGGCAGCAGCCCAUCAGCGUGCCCCCGAGCACCAACAGCACGGUGUACCGGGGUCUGGAGGUACCUGCAGGAGGCAGUGGGCGAGCAGGCGGGGCAGCCCAAUGCCAUGGGCCUGAUCUCACCGCUGCCUCCUUCCCCAGGGCUUCGUGGCGGCCAUCUCGCCCUUUAACUUCACUGCAAUCGGCGGCAACCUGGCGGG*

*>hsa-mir-2964a:9:131154837:131155038*

*CGCCCCGAGAGGUCUCGCCCGCGCUGAGCCCUGUCCCUGGGGCCCCCGCCCCUGUUGCGCCCGGAGCUCAGCCACAGAUGUCCAGCCACAAUUCUCGGUUGGCCGCAGACUCGUACAAGAAUUGCGUUUGGACAAUCAGUGGCGAAGCCCCUGAGUUCAGGGCCCUGGUCUCCUGCGGGGUUCCCCUUCGCUCCUAAAAA*

*>hsa-mir-548as:13:93142343:93142544*

*UUUCAGCGCAUUGUUUCCCGAAAACUCCAAGCUGAAUUGUUCUCGGUUCUUUGUGAAUUAUUAGGUCAGUGCAAAAGUAAUUGCGGGUUUUGCCGUUGCUUUUAAUGGUAAAACCCACAAUUAUGUUUGUACCAGCCUGAUCCUAAGCAAUCUCAUUAUCGUCUAGAUUCUGCCACUUUUCAUUGUAUUAUUAUAUCUAU*

*>hsa-mir-548ab*

*UAUUUAUUUAUUUAUUUAUUUACAAUAACAUGUCUAUAAUCUGUUUUUUCCAUCCAUUAUGUUGGUGCAAAAGUAAUUGUGGAUUUUGCUAUUACUUGUAUUUAUUUGUAAUGCAAAACCCGCAAUUAGUUUUGCACCAACCUAAUAGAAAGCAUGUUCCAUUGGGGCUAGGUCAGCAGUGUAUUGGCUCACCACCUCUU*

*>hsa-mir-4495*

*AUCUUCAAAACUAUUCUAUGUGAUGGGUGAUAUUUUUUUCUCAUUUUACAGAUGAGAAAACUGAGGCUAAGAAAUGUAAACAGGCUUUUUGCUCAGUGGAGUUAUUUUGAGCAAAAAGCUUAUUUACAUUUCUGAGUCUCAGUUUCCCCAACAAGUAGCUGUUAUUUUGAUAACCCAGGAAUACCAUGGUGUGUAUAAGG*

*>hsa-mir-4489:11:65416595:65416796*

*AGACAUUUUUCACUGUUGUCAGGAUGCCGGGGAGCUCUCCUGCUCCUGGAAGUGAGCCUGGCGGGCUGGGGGUGGGGCUAGUGAUGCAGGACGCUGGGGACUGGAGAAGUCCUGCCUGACCCUGUCCCACCCCACAGGAGUUUUUCGGAGCUGUACACGCUGUCGCUGCAGGAGCCUAGCCGGCGGGGGGCCCCAGAUCC*

*>hsa-mir-4708*

*CUGUUGUUAUGUUACCUACCCGCUCUUGGCAGGGCCCUGAGAAGCUGAGGUAUGAGACACAACAGCAUAUUUAGGAGAGAGAUGCCGCCUUGCUCCUUGAACAGGAGGAGCAAGGCGGCAUCUCUCUGAUACUAAAAGGAGAAUACGGGAGCACCUUUCUUACCUACAUUUCCCCUGGAACUAGUCUUUCCACUUGCAAG*

*>hsa-mir-4467:7:102111847:102112048*

*GCCCAGGAGGAGCUUCGACACAGCAGCUGCUGUGUUGAAAGGAGCAGCAGGGGCCUUGGCGGCAGAGGUGGUGGCGGCGGUAGUUAUGGGCUUCUCUUUCUCACCAGCAGCCCCUGGGCCGCCGCCUCCCUCCCUGCCCGUAUGUCUGGUCCCUGUGUCUAAGAUGCUUCUUCUCUUACCCUCUGCCCAACUGGCUCAGG*

*>hsa-mir-4662a:8:125834160:125834361*

*AUUUAUAUACAAGGAAGUUUACUGCAACCAUGGCUGCAACAGAAAAAGCCAUUGUGUACCACAGUGUCUAUUUAGCCAAUUGUCCAUCUUUAGCUAUUCUGAAUGCCUAAAGAUAGACAAUUGGCUAAAUAGAAAUUGUGGUACAUCCAUACAAUGGAAUACCAUGAAGCCAUCGACAAAACAUCAAGAUUCUAUUGACC*

*>hsa-mir-4767:X:7065836:7066037*

*CGCCGACCGGAAGCGGGUGCUGGGGCCGCGUAGACGCCGCGGCCACGCGCGCCCGCCAGCCCGGACAUGGGCCCGCGGGCGCUCCUGGCCGCCGCCCGACUUCGGGGCCAGCCGGGGGCAGAGCGCGCGGGAGCCCGAGCGUCCCUGCAUGAACACCGCCCCGCCGCGGCCCCCAGGCCGUGACGUACCCCGCGCCGACC*

*>hsa-mir-548am*

*AAGGCAAUUCUACCAAGAAAAGAGGUAAUGAAGCCCAGCAGGUAGUGGUUUUACUUUUUUAUUAAGUUGGUGCAAAAGUAAUUGCGGUUUUUGCCGUCGAAAAUAAUGGCAAAAACUGCAGUUACUUUUGUACCAAUGUAUUAGAUGCUAUGGGUGAUUGCCUUUAGCCCCCUGGAAAUAGGAGAUAUGGCACCGGGGGA*

*>hsa-mir-4750:19:50391359:50391560*

*GCGCACUUCUGAGCCCCCAGAGGGCCAGCCCGGUGUGUCUUCAUUGUUUCCCAGCACGAAGCACAGAGUGGGCGCUCGGGCGGAGGUGGUUGAGUGCCGACUGGCGCCUGACCCACCCCCUCCCGCAGCACAUCAACGAGCUGACUAUGAAGCUGAGCGUGGAGGACGUGCUGACCCGCGCCGAGGCCCUGCACCGCCAG*

*>hsa-mir-5689:6:10439888:10440089*

*AUGCCUGGACUCUGUCACUUAUGUCAAUAAAUUUACAAAUGCAAAAUACACAUUUAAUUCCAGCGUGGUAGCAUACACCUGUAGUCCUAGAUACUCAGGAGGGUGAGUAUCUAGGACUACAGGUGUGUGCUACCACGCUUGAACUCAGGAGUUCAAGGCCAGCCUGGACAACACAGGGAGACCCCCUCUCUAAAUGUAUA*

*>hsa-mir-4800*

*CAGGUGCCGUGUGAGGGGCUUAAUGACCACCACAGGGAGCUCAGACCCCAACCCUGGAUCCCAGGAGAAAGGAGUGGACCGAGGAAGGAAGGAAGGCAAGGCUGUCUGUCCAUCCGUCCGUCUGUCCACCUACCUGUCAGUCCACAUAGGCUCCUGGCGUGGACAAGGGGUCUGUGAAGGGCGGGAACUGGGUGAGCACC*

*>hsa-mir-4520a*

*GCGCACAGGAGCAUGCCCUCAAUUCCAAGAAGAUCAGGUAGCCGGAAAAUUUAUUAAGCGCUUACUGUGUGCCACCUGCGUGUUUUCUGUCCAAAUCAGAAAAGGAUUUGGACAGAAAACACGCAGGAAGAAGGAAUCAACCCCUGGUACAGCAGGCAGGCGAGGUAGGGGUAGGAGUUAUGCACGUGUAUGCAAGCCUG*

*>hsa-mir-5700:12:94955501:94955702*

*AUUGUGCCACUGCACUCCAGCCUGGGCAACAGAGCGAGACUCUGUCUCACAUAAAUAAACUAAUUAAUUAAUGCAUUAAAUUAUUGAAGGCCCUUGGGCACCCCAGGCCUUCAAUAAUUUAAUGCAUUUAUUGAGCAUCUACGAUCUCAUCUAACCCCUAUAAUCCUAGGCAGUUAACAGUUUAGGAAGGGGAGAUUCUC*

*>hsa-mir-4497:12:110271094:110271295*

*CCCCAGGGGCGCGAGGGGACCUCGGCUGAGCCGCCCCUCCCGGGCCCGGGCCCCACUUACCUCCGGGACGGCUGGGCGCCGGCGGCCGGGAGAUCCGCGCUUCCUGAAUCCCGGCCGGCCCGCCCGGCGCCCGUCCGCCCGCGGGUCCCUCCUCCGCCGGCUCAGGUUCCAGCCGCGCGCCGCCCCCGGCCAGCUCCCCG*

*>hsa-mir-4479:9:139781119:139781320*

*GGCGGGAGAGGCCGCGGGGCGGGGGCGGGGCCAGCCCGAGGGGAUUUCUCUGAGCCGCUGGGCCGGAAACCAAGUCCGAGCGUGGCUGGCGCGGGAAAGUUCGGGAACGCGCGCGGCCGUGCUCGGAGCAGCGCCAGGGCACGGUCCGGGGUUUUGCGCGGGGUCGCGGCUCCCAGUACCGCCUGAGGCCCGUGGGCGGC*

*>hsa-mir-4503*

*AUAAGAAUCUUUAAGAACAAGAAAUAAUAAAUGACUUCCUCCCCACAAAUUGAAAUUGACAAUGUAGAUAUUUAAGCAGGAAAUAGAAUUUACAUAUAAAUUUCUAUUUGUUUCUAUUUCCUGCUUAAAUAUCUACAUUGCCAAAAACCAAAGGAAAGAAGACAAUCAUUUAUUUAUUCUACUCUGAUUCUUUCUUUUUU*

*>hsa-mir-4690:11:65403710:65403911*

*GGGCGGUGGCCUGACCUCCCUCAGCAAUAACCCCCCCGUGGCACACCCCACACCUGAGAACACGGCAGGUGAGCAGGCGAGGCUGGGCUGAACCCGUGGGUGAGGAGUGCAGCCCAGCUGAGGCCUCUGCUGUCUUAUCUGUCUCCUACAGGCAAUGGUGACCAACCCCUCCCACCAGGCCCUGGCUGGGGGCCGCGGUC*

*>hsa-mir-5582*

*UUACACUUAGAAACCAUAAUGUCCGUAAGUCAACUUCCUAGGCAACCUAACAAUCAUAAUCAACUUCCUAGGCACACUUAAAGUUAUAGCUACAUCAGUUAUAACUAUAUCAGUUAAAACUUUAAGUGUGCCUAGGAAGUUGAUUUAUUACUCUUCUUCAUCAUGGACCAAAGGUAUUUUAGCCUGAGACCAUAGCUAAA*

*>hsa-mir-4641:6:41566392:41566593*

*AGGUCUGAUGGGAUCCUGGGGAGGGGGCUGUGGGCACCCCACUGUGCCCCAGAGUUCUGGGUGAGUUUGGGGGGCAGGGGGCAGAGGGCAUCAGAGGACAGCCGCCUGGUGCCCAUGCCAUACUUUUGCCUCAGCCACCAGGUGCAGGUGAAGGAGGAGCCAGCAGAGGCAGAGGAAGACAGGCAGCCCGGGCCUCCCCU*

*>hsa-mir-4636*

*UGCUAUUACCCUUGAAGUGACUUUAAAACAUAGCAUCUAUAAUAAAAUAGUUCUCACUGAGCUAUAGAUUCAGAACUCGUGUUCAAAGCCUUUAGCCCAGCAAUGGGAGAGUGCUAAAGGCUUCAAGCACGAGUUCUGAAUCUAUGGCAUGUUACAACACCCAAUCUCCCUAAUUAGGAAACAUCCCAAUUUAUUCUUGA*

*>hsa-mir-5706:5:118490271:118490472*

*AGGUUGGUCCAUGAGGUUUAAGGAAAGGAGCUUUCUCCGUAAACUACAAGUACAAGGUGAAGCUAGGUCUUCUGGAUAACAUGCUGAAGCUUCUACGUCAUUCAGCACUUGCUUCAGCAUGUUUUCCAGAGGAUCUAGCUAAGAUAAUGGAUGAAGGUGGCUACACAAAAGGACAGAUUUUUAAAGUACAUGGAACAGCC*

*>hsa-mir-5004:6:33406061:33406262*

*GGCCAAGGUGAGUGUUGUGCCCUCAGGGAAAGGUGACUUGGGAAUGGGCACUUGCUUGGGGGUUAGUGAGGACAGGGCAAAUUCACGAGAUUGGGUUGUGCAGAGGCUGACACUUGGAUUUUCCUGGGCCUCAGGACUUCCUUUCAGACAUGGCCAUGUCUGAGGUAGACCGGUUCAUGGAACGGGAGCACCUCAUAUUC*

*>hsa-mir-4538*

*GCUGAACUGGGCUGGGCUGAGCUGAGCUGAACUGGGCUGGGCUGAACUGGGCUGGGCUGAGCUGAGCUUGGAUGAGCUGGGCUGAACUGGGCUGGGUUGAGCUGGGCUGGGCUGAGUUGAGCCAGGCUGAUCUGGGCUGAGCCGAGCUGGGUUAAGCCGAGCUGGGUUGGGCUGGGCUGGGUUGGGCUGGGCUGAGCCGG*

*>hsa-mir-4756*

*CAAUGUGUGUGAAGUGCCUGGCACCAGGGUCAGGGCAAGGGUGAAGCCAGUGAGCACCUGGGGGAUAAAAUGCAGGGAGGCGCUCACUCUCUGCUGCCGAUUCUGCACCAGAGAUGGUUGCCUUCCUAUAUUUUGUGUCCUGCUAUGCUCACCCUGGUGCUGCCUGGCACAUAGUAAGCCUCCAAUUAAAAUUACCUGCG*

*>hsa-mir-548ap:15:86368813:86369014*

*UUUCAGGUGUUUCUCUCUACUAGGAGACUGCCUUUCCCUGGCACCAGCUGUGACCAAUUCCUAGGUUGGUGCAAAAGUAAUUGCGGUCUUUGUCAUUAAAACCAAUAACAAAAACCACAAUUACUUUUUACUGACCUAAAGAUUAAUUUAGGGAGACAGUGUAACAAAUGCUUUACCAUCACCUGAUGGUUGCCUGACAU*

*>hsa-mir-4779*

*CCUCCCAAAAUGCUGGGAAUAUAGGCACGAGCCACCGUGCCCAGCCAACCCCAUCUUCUUAAAUGUCUUACUGCUUUUACUGUUCCCUCCUAGAGUCCAUUCUUUACUCUAGGAGGGAAUAGUAAAAGCAGUAAGACAUUUAGUAAAAGGCUUUUUAAAAGGCAAACAUGAGGUCAUGCUCUUUGGAGACAUCGCAUCAC*

*>hsa-mir-4720:16:81418560:81418761*

*CAUUCUACUUAAAUGGAGGAGUUCAGUGAUGCCGUGGCCAAAAGAGUUAUGCCCAUUUCUAAAAGCCUGGCAUAUUUGGUAUAACUUAAGCACCAGGUAAAAUCUGGUGCUUAAGUUGUACCAAGUAUAGCCAAGUUUAACUGUCGUUGUGUAUUAAUAAGAUUUAAUUUUUAUGCUUGUGCUUUUCAGAGUAAAGGGCU*

*>hsa-mir-4766*

*UUUGCAUUCUUAAUUGAUUCCAGUUCUUUGUAAGUAUUCUAUACUGUUGACCCUUCUACCUUUCUGAAGCUCCUUCUGAAAGAGCAGUUGGUGUUUAUUUUUUACUAAAUAGCAAUUGCUCUUUUGGAAGGAACUUGAGAAGACAUUACUCCUUUUAGGGCCCUAAAAUUAACAGUGGUUUCUACUACUAACAGAUACUA*

*>hsa-mir-1273f:1:53394295:53394496*

*UACGAAAAUUAGUCGGGCAUGGUAGCACACGCCAGCUACUCGGGAGGCUGAGGUGGGAGGAUUGCUUGAGCCUGGGAGAUGGAGGUUGCAGUGAGCUGAGAUCACGCAACUGCACCCCCAGCCUGGGCCAUAGAGUCAGUCCUUGUCUCAACAAAAACAAAAAAUAUCCAUGGAAAUGGAAAAGUGGACCAAUACCUUAU*

*>hsa-mir-1245a:2:189842753:189842954*

*UAGGGAAAGAAAAAUGCUCAGGUAAUAACAGAGCCUUGAAAAAUUUGGAUUUUCAAAACUACCUAUUUAUGUAUAGGCCUUUAGAUCAUCUGAUGUUGAAUACUCUUUAAGUGAUCUAAAGGCCUACAUAUAAAAAGGUAUUUUUAUUAAAUUCUGGAAUUAAACAUUUCAGCAUUACAAGAAAAGAAAGCAAAUCCACU*

*>hsa-mir-4715*

*AUAGAACUUAAAUUGUAAAACAUCCCUGGAGCUACUGCCCUCCCUUAGGUCCCAUGAGAAAGGGGAAUGAAAGUUGGCUGCAGUUAAGGUGGCUAAUCAGCUGAUGGUGCCACCUUAACUGCAGCCAAUUCUAAUUCCCCUUUCUCUUGGAAAAAGAUGGGGAGAUUAUUCUGGAGUGAAUUGUGGGCCCAGUGUAAUCA*

*>hsa-mir-5704*

*GUAUUUCUUCCAUCCAUCUUGGUUGAAAACCCUUAGAAUUAUUUCCAAUAGUCACUAUUGUCUGAUCUUGUUUAGGCCAUCAUCCCAUUAUGCUAAGUCCAUGGGCAAACAUAACAGGAUGAUGGCCUAAACAAGACCAGACAAUUAUCCCAUCAUCAGUGUUGUGCUCAGUACAUGGACUAGGUAGCAGAAAUCAGUGA*

*>hsa-mir-663a*

*GGGGCCCCGGUGGCGGGACCAGGGGCUCGUGAGGCAGGUCUUGGUGGGUGCCGAGGGCCUUCCGGCGUCCCAGGCGGGGCGCCGCGGGACCGCCCUCGUGUCUGUGGCGGUGGGAUCCCGCGGCCGUGUUUUCCUGGUGGCCCGGCCAUGCCUGAGGUUUCUCCCUCAGCCGCCCCUCUGCGGGCUCCCAGGUGCCCUUG*

*>hsa-mir-5681b*

*GUAAAACACAUUUUAAAGUGCCAUUGACUGCUGAGGCAACUCACUUAAUAUCAACAAGUCUCAGUUUUUGGAAGAGGUAUUGCCACCCUUUCUAGUCUAAUAGGGACUAGAAAGGGUGGCAAUACUCUUCAAAAACUUCACAAGGAAGUUGUAAGGAAUAUAUGAGAUCAUCUAUAUAAAGCCCUUUGUGAACAAUAAAU*

*>hsa-mir-3689c*

*GUGAUCCCGUGCUCCCCAGGAGGUCUGAUCCCGUGCUUCCUGGGAGGUGUGAUAUCGUGGUUCCUGGGAGGUGUGAUAUCGUGGUUCCUGGGAGGUGUGAUAUCGUGGUUCCUGGGAGGUGUGAUAUUGUGGUUCCUGGGAGGUGUGAUCCCGUGCUUCCUGGGAGGUGUGAUCCUGUGCUUCCUGGGAGGUGUGAUCCU*

*>hsa-mir-4517:16:28969843:28970044*

*UAUUUCUUUGUGUUACUCGCCUGUACCCCUUGCUGACACGAUUUUAUUCUAUACCUUGCCAGGUAAAUAUGAUGAAACUCACAGCUGAGGAGCUUAGCAAGUAGCUAAGGCCAGAGCUUGUGUUUGGGUGGUGUGGCUGGGGGCAUGGAUUUCAAGGCAGGAAGACCUCUUUUGCUUGUUGUCCCCAUCCCUAGUCGGAG*

*>hsa-mir-4742*

*ACCAUAAAUUUGUCAGAAUGUUGGUAUUUUGCAUAAUAGAAAAUGAAGAUAUUUCAUCAGGCAAAGGGAUAUUUACAGAUACUUUUUAAAAUUUGUUUGAGUUGAGGCAGAUUAAAUAUCUGUAUUCUCCUUUGCCUGCAGGGAGUUCAUUUAUGGGACUUGCAAGACAGAGUUUUAGUAAGAAAGUAUCAAGGUGUUAC*

*>hsa-mir-5095:1:53400545:53400746*

*CCAGGAUGGUCUCGAUCUCCUGACCUCGUGAUCCGCCCGUCUCGGCCUCCCAAGUGCUGGGAUUACAGGCGUGAACCACCGCGCCCGGCCUAACUUUUAAGAAACGUCGGCCCGGGAGCGGUGGCUCACGCCUGUAAUCCCAGCACUUUGGGAGGCCGAGGUGGGCGGAUCACCUGAGGUUAGGAGUUUGAGACCAGCCU*

*>hsa-mir-5195*

*UUCAUCCGGGGAGGAAACACUGGCUGUUUGUGUCCUCAGGAGCAAAAACCAGAGAACAACAUGGGAGCGUUCCUAACCCCUAAGGCAACUGGAUGGGAGACCUGACCCAUCCAGUUCUCUGAGGGGGCUCUUGUGUGUUCUACAAGGUUGUUCAUGGUGUAUAUUACAUGGUUAACAUCAAAAGGCUGCCUAAUAGGCAC*

*>hsa-mir-548u:6:57254869:57255070*

*UGGUCUCCCUUGCUGCUGAUCUGUGGGAAACAAAACGUUGCAGUUGAAAGCCAUGAGACUAUUAGGAUGGUGCAAAAGUAAUGUGGUUUUUUUCUUUACUUUUAAUGGCAAAGACUGCAAUUACUUUUGCGCCAACCUAAUAACAGAAAAAAAAUCAUUUUUCGGGGGAAAAUAAGCAUAUGCUCUUUCUAGUUUGACAG*

*>hsa-mir-4672*

*ACACAGCUGAACAGAGGCAGGGGUGAGCCUGGGUCCAGGUCAGUCUGACGCCGGAGGUCAGGCUGCUUCUCGCCUCUGUCCAGCUGUGUGGCCUUGGACAAGCCUCUUGGUUACACAGCUGGACAGAGGCACGAAACAGCCUGACCUGAGAGGCAGUGCCCCAAGGCCUUGACAGGGGUCCAGGGCCAAAGCUGCGCAGA*

*>hsa-mir-4681:10:121137417:121137618*

*AAAAACUGAUUGGGAAAGGAUGCAAAGAUUUGUUUUCCUCCCCUUGGGGGAUUGUGUUGAGUGGGAGGCAACGGGAAUGCAGGCUGUAUCUGCAGGGCAUUGUGCUAACAGGUGCAGGCUGCAGACCUGUCACAGGCCGCUGAGACCAGGUUCCCUACUGUGGCUGAUGAUCACACUCAUCUAGCUUGACAUUUUAUUAC*

*>hsa-mir-4532:20:56470375:56470576*

*GCUACCACCAUUCACAACCAGGGGCAGGCCCACCCCUUGCCUAUAAUCUUCUUGGAUAAGGGACAUCACACUGUACAGACCCCGGGGAGCCCGGCGGUGAAGCUCCUGGUAUCCUGGGUGUCUGAAGACAAAGGUGGUUGAACAGAGCAACCCUGAACUGUUUCUGGGACAUCCACAAGCUAAAGCAGCCUGAUGGUGAG*

*>hsa-mir-5684:19:12897874:12898075*

*UGGAGGCUGAGGCAGGAGGAUCGCUUUUACCCAGGAGUUCAGGGCUGCAGCGAGCUAUGAUUGUGUAGCUGAACUCUAGCCUGAGCAACAGAGUGAGAUGGUCUUGUUUUGUUGCCCAGGCUGGAGUCCAGUGUCAAGAUCAUGGCUCAUGUGAUCCUCCUGCCUCAGCCUUCGGAGUAGCUGGAACUACAGGCACACCC*

*>hsa-mir-3976:18:5840664:5840865*

*AGGGGUGGGCGUGAGGCGGGAGGAUGGAAUGAGGGAUAUAGAGAGCAGGAAGAUUAAUGUCAUAUUGGAGUUGGACUGCAGGGCUUCCUUUACACAAUAAAUAUUGUAUGAAGUGCUGAUGUAACCUUUACUGCAGCAUGACAUGGGAUUUGGCUGUUUUUAUGGCUCAUGAUUAAAGUAAGAAACAAGUCUCUGAUUAG*

*>hsa-mir-5690*

*AUAAAAGGGUCCUGAAGUGAGAGAAAUCAAAGAUUGGAGAAGGAAAGAGGGAGAAAUUAUGCCACUUUUAAUUUCAGCUACUACCUCUAUUAGGAUUUGGGAGUUAUACUAAUAGAGGUAAUAGUUGAAAUUAAGAGUGGAUGAGUUCUGGUAUGAAGUGGUUGAUGGGGACAACUGUUGAUUGACAACUGCAUUUUAAG*

*>hsa-mir-4788:3:134156609:134156810*

*AAAGAGAAGUCUAAAUUUGAUGAAAUGGCAAAGGUGGAUAAAGUAUGCUAUGAUUGGGAAAUGAAGGAUUACGGACCAGCUAAGGGAGGCAUUAGGAUCCUUAUUCUUGCCUCCCUUAGUUGGUCCCUAAUCCUUCGUUUGGAUCCCACUGUCUGGAUUCUUCCUGCUCUGUUCCAAAUUCUGCCCUAAUAUCAAAUCCA*

*>hsa-mir-5686:10:98901380:98901581*

*UAGCCACCUUAAUGCACUGUAUUGCAUUGUAUUGUAUUGCAUUGCAUUGUAUCGUAUUGUAUCGUAUCGUAUCGUAUCGUAUUGUAUUGUACUGUAUUGUAUUGUACUGUAUUGUAUCGUAUCGUAUCGUAUCGUAUCGUAUUGUAUCGUACUGUAUUGUAUUGUACUGUAUUGUAUUGUAUUGUACUAGACAGGGUCUC*

*>hsa-mir-4528:18:50763414:50763615*

*GUAACUAUUUAUAUAUGUUUAAAACCGUUACUCUCCACAAAUAUAUAUAUAUUAUAUAUUCUACUGAGAGUACAGAUCUUUAUAUAUAUGAUCAUUAUAUGUAUGAUGAGAUCAUUAUAUGUAUGAUCUGGACACCCAGUAGAAUCUAGCUCAUUAGAUUGUCCUUCACAGUUAAACAAUGCAAAGCCUAUAGAAGAACA*

*>hsa-mir-2682*

*GGAGAAACAGGAUGGGAACCGGAUUGGUUGCAGUGUGGCACAGACCUGGAACCUUCCUGAAAGAGGUUGGGGCAGGCAGUGACUGUUCAGACGUCCAAUCUCUUUGGGACGCCUCUUCAGCGCUGUCUUCCCUGCCUCUGCCUUUAGGACGAGUCUCAAACACCAACAAACGCAAGGCACGUCCCCCCUCUCAGGUCAGC*

*>hsa-mir-1268a*

*UGACACCAGCAUGGGCAACAUGGUGUAAGAACCUGUAUCUACAAAAAAAAAAAAAAAAAAAGAAGAAAGAAAAGUUAGCCGGGCGUGGUGGUGGGGGCCUGUGGUCCCAGCUACUUUGGAGGCUGAGGUGGAAGGAUCACCUGAGCCCAGGAGGUGGAGGCUGCAGUAAGCUUAGAUUGCAGCACUGCGGUCCAGCCUGG*

*>hsa-mir-4698:12:47581534:47581735*

*GAACUAUAGCUUCAUCAUAAGGCAGCCAAGUCUUGUGAUAUAUGGGUUAAAGGAAAGUUAUGCUUCUCCUGGGGUCUUCCUCUACAUUUCCACCUAGACGGGCCUGGGUCAAAAUGUAGAGGAAGACCCCAGAAGGAGCAAAACUUUCACCUGCGCCUCUUCCACCUGGGUCUGUCCUUUAUCCUACCAGCUCCAAGAAC*

*>hsa-mir-4639:6:16141723:16141924*

*CUUUAAUGUUAAGCAUUUUCAGUGUGACUUUUAAAAUUGCUUUCCUUAAAAGUGACUGUGAAAUUGCUAAGUAGGCUGAGAUUGAUGUCAGGUUAUCCCCAAGCAUAACCUCACUCUCACCUUGCUUUGCAGGCAUAUCUUUUUCUUGCACAUCAAGGAGGCCCUCUUGGCAGGCCACCUCUUGUGUUCCCCAGAGCAGG*

*>hsa-mir-4710*

*GCAGGGCGCAGAGCAGCGCUGCAGGCACAGGGGCGGGGGCUCCCGGCAGGCCAGUGCCCUGCCUGUCCCAGACCGAGUGGGGUGAGGGCAGGUGGUUCUUCCCGAAGCAGCUCUCGCCUCUUCGUCAGGGAAGGGAGUGUUGAGUGUUCCGCCCAGCAGAACCAGGCCACACCAGGGGCUCCCUCCUCCCUGCCCCACCU*

*>hsa-mir-1255b-2*

*UACUCUAUAAUAGAUCCUUUUGUUGUCAUUUCAGCAGUGUUCACAGCAUCUUCACUAGGAGUUGCUUCUUACGGAUGAGCAAAGAAAGUGGUUUGCGCCUCAAGAAACCACUUUCUUUGCUCAUCCAUAAGGAGCAACUCCUUAUCUGUUAGUUUUAUCAUGAGAUUGCAACAAUUCUCACACCUUCAGUCUGUACUUCU*

*>hsa-mir-502*

*GUGUAACAGAGAACCUUCUCUGCUUCGAGCACGGCAGGUGCCCAAGCCUUCUGCCCUGCUCCCCCUCUCUAAUCCUUGCUAUCUGGGUGCUAGUGCUGGCUCAAUGCAAUGCACCUGGGCAAGGAUUCAGAGAGGGGGAGCUCAACUGUACCAAGAAGGACAGAGAGCCUAGACAUCACCCCUCCCACCCUCCCCUGUCA*

*>hsa-mir-548ah:4:77496641:77496842*

*AACAAAUUCAGUCAAGUCACAGGAUACAAAAUCAACAUACCAAAAUCAGCAGCAUUUCUAUUAGGUUGGUGCAAAAGUGAUUGCAGUGUUUGCCAAUAAAAGUAAUGACAAAAACUGCAGUUACUUUUGCACCAGCCCAAUAUAUGCCAACAGUGAACAAUCUGAACAAGAAAUUUAAAAAGUAAUCCCAUUUACAAUAG*

*>hsa-mir-4667:9:35608023:35608224*

*UUUGCAAAGAAAGAAUUCCAGACUAGCUGGCUCAUAACCAAGGAGGAUUCCCUAGAGGUUGAGGUUCUGACUGGGGAGCAGAAGGAGAACCCAAGAAAAGCUGACUUGGAGGUCCCUCCUUCUGUCCCCACAGGACUUUGGCCUGGAUGUGCCUGCUUUCCGAACUCUGGUGGGGGAUGACUGCCCACUGCCUUUCUUGC*

*>hsa-mir-4423:1:85599416:85599617*

*GCAGAAAGCUUUCCCCUGAGAUGUGGAACAAGACACAGCUCACACCAUCAGGUGCCUGACAUCAUGUACUGCAGUUGCCUUUUUGUUCCCAUGCUGUUUAAGCCUAGCAUAGGCACCAAAAAGCAACAACAGUAUGUGAAACUGAGACACAAAAGAGCUUGGAGCCAGAUUGUGAAGGCUCCUGUUUACCAAGCUAAGGA*

*>hsa-mir-365b:17:29902384:29902585*

*ACCUCGGCUCAUCUGGGCGUGGCAGACGACUGCUUCCUCACCUGGAGAGUGUUCAAGGACAGCAAGAAAAAUGAGGGACUUUCAGGGGCAGCUGUGUUUUCUGACUCAGUCAUAAUGCCCCUAAAAAUCCUUAUUGUUCUUGCAGUGUGCAUCGGGCAGCGGCCAGCAACCUGCCUCUCACCCACCUGGCCCUUCUGGGA*

*>hsa-mir-4633:5:128433321:128433522*

*UUUGAGGAAGUGCAGAAGAACAUAGGGAGGUGGAGGUGAGUAACUUGCUGCAGGUUACAUGGCAAGUCUCCGCAUAUGCCUGGCUAGCUCCUCCACAAAUGCGUGUGGAGGAGCUAGCCAGGCAUAUGCAGAGCGUCACUUACAGCCGAACACAGCACUACUGCUUGCAUGUUUAAUGAGAAUUGAGCAGUCUGGAAAAC*

*>hsa-mir-4427:1:233759830:233760031*

*CUCUGUGACCUGAGUUAAGCUAUUUACUCAAGUCGAUGCCCCAGUUCCCUAUUCACUCUGCUUCACUGAAGCCUCUUGGGGCUUAUUUAGACAAUGGUUUCAUCAUUUCGUCUGAAUAGAGUCUGAAGAGUCUUUGGUGGAAGUAUGCACUGAGGAGGGAGGCGGCAGAAGUGCCCUAUCCCAGGAAAGUGUGCUCCCCA*

*>hsa-mir-4654:1:162126835:162127036*

*CAAACAGAGGUACAGGAAAACAUCACCUGGAAGCAAGGGAAGGCCGAGGCAUGGUAUGGGGCUGGCUGGUUGUGGGAUCUGGAGGCAUCUGGGGUUGGAAUGUGACCCCAGUCUCCUUUUCCCUCAUCAUCUGCCAGCCCUUAUGGCCCAUGUGCCUCUUCCUGUGGCUCCCUUCCUAUGUGCUGCUUCCAGUGCUUAAU*

*>hsa-mir-4677:1:243509418:243509619*

*UGAACAGACAGUUUCAGAAAAGAAGUCGUGGUGUCUCAUUCCAUGGAUGAUUUGGAGUAGCAAAGCAGCAAUUGUUCUUUGGUCUUUCAGCCAUGACCUGACCUUCUGUCUGUGAGACCAAAGAACUACUUUGCUUGGCCACCAUCUGCACUCAAGAGAGAAAUUUACAUUUAACUUUUAAAAGCGGGGCAUGUUGAGCA*

*>hsa-mir-4656*

*CUCCCCUGGGUUUGGGGAGACCCCUGGCGUCAGCACACCCGGGACAGUAAAGCCAUCGGGACAAAGGCUGGCGUGGGCUGAGGGCAGGAGGCCUGUGGCCGGUCCCAGGCCUCCUGCUUCCUGGGCUCAGGCUCGGUUUGUCCAUCUGCAAAAUGGAGCACCCACAGGGCCAACCUCCAAGCUUAUGAGGGUUGAUGUGG*

*>hsa-mir-551a*

*CCUCGCGGCCGACGCCUGAGGCUGUUCCUGGCUGCUCCGGUGGCUGCCAGAGGGGACUGCCGGGUGACCCUGGAAAUCCAGAGUGGGUGGGGCCAGUCUGACCGUUUCUAGGCGACCCACUCUUGGUUUCCAGGGUUGCCCUGGAAACCACAGAUGGGGAGGGGUUGAUGGCACCCAGCCUCCCCCAAGCCUGGGAAGGG*

*>hsa-mir-212*

*GCGGAGCAGCAGAGCCCCCAGCCGGGGCAGCCGGGGCGACCGGGGCACCCCGCCCGGACAGCGCGCCGGCACCUUGGCUCUAGACUGCUUACUGCCCGGGCCGCCCUCAGUAACAGUCUCCAGUCACGGCCACCGACGCCUGGCCCCGCCCCAGGACCGCGGCCCCGGCCGCCGCCGCCGCGCCCGGGGACCCCCAGUCC*

*>hsa-mir-4772:2:103048687:103048888*

*UGCGUUCCAGUGAGACCACUUUCUGAACUCUAUGGUCAGGUUGAGUCGCUGGCUGAGCUCUGUGAUUGCCUCUGAUCAGGCAAAAUUGCAGACUGUCUUCCCAAAUAGCCUGCAACUUUGCCUGAUCAGAGGCAGUCACAGAGCAAUACCCUUGUGUCUUCCAUUCACCCACAACAUGAAGGAAUUGCUUAAUUAACUAA*

*>hsa-mir-4508*

*CCUUCUCAGCACUCUCCUAUCCUUCAGAUCUAACCUUUAGGCACUGCCGGCUGCCGGCGGGGUCCAGGACCCAGCGGGGCUGGGCGCGCGGAGCAGCGCUGGGUGCAGCGCCUGCGCCGGCAGCUGCAAGGGCCGCAGAAAUUAAACGCGAGUGUCCAGAAUCGGGCAUUGUGGGUUUAAAUCCUGAAUCCACUGGUCAC*

*>hsa-mir-5708:8:81153566:81153767*

*GGUCUCUAAUUCUCUGCCUCAAGCAGUCCUACCACCUCACCUUCCCAAAGUGCUGGUAUUACAGACAUGAGCGACUGUGCCUGACCAAAAGUCAACAUUAAACAACAAAUCUUGGCCAGGCACAGUGGCUCAUGCCUGUAAUCCCAGCACUUUGGGAGGCCGAAGUGGGCAGAUCACUUGAGGUCAGGAGUUGGAGACCA*

*>hsa-mir-4432*

*UGGUGGAUUAUGGCCAGAACAGUCUAUAACUGAGAAUCCCCUCAGACUCUGGGCAGAAAGGCAUCUUGCAGAGCCGUUCCAAUGCGACACCUCUAGAGUGUCAUCCCCUAGAAUGUCACCUUGGAAAGACUCUGCAAGAUGCCUUGUUAACCAUCAUGGACCCUGUUAACCAUUGUGGGCUAGCAUGGUUCUACAAAGAC*

*>hsa-mir-4515:15:83736026:83736227*

*CGCAGGCCCCUCCCUGACCGCGCGCGCCCCCGCACGCCCGGGUUGCCGCUGUGCGCGGCCGCGGGAGGUGUAACAGGACUGGACUCCCGGCAGCCCCAGGGCAGGGGCGUGGGGAGCUGGUCCUAGCUCAGCGCUCCCGGAGGUGGAGCUUCUGCAGGCUGGGUGCUGCCGGGCCUACGGGCACCCACCCCCUGGCUCAG*

*>hsa-mir-4474*

*AGCUAUUACCUGUAGAAUUCAGCUCCUUACAGUGCUAUGUAAGGAACAUCACAGGCUGGCAUUUGCCUACCUUGUUAGUCUCAUGAUCAGACACAAAUAUGGCUCUUUGUGGCUGGUCAUGAGGCUAACAAGGUAGGCACCUGGAACCACACUUCAAGUCAGGUUCAUUUGUGGUGCCCUGUCAAGCCAGGCAUUCUGUA*

*>hsa-mir-5047*

*CAUGUCGAGACCUGCCAGAGAGAGACACAUUCUCAAGUGAAUCCUGGCUUCUUGGAAGCGCUUGCCUAGACGAGACACAGUGCAUAAAAACAACUUUUGGGGGACAGGUAUGUUUUCUUGCAGCUGCGGUUGUAAGGUCUUGGCAAGACAAGCAGUGUGGCCAGAAUUUUGAACUUCUGAUGAAUGUGUAAUGCAAAGGA*

*>hsa-mir-4700:12:121160934:121161135*

*CAGGAUACUCUUUUCCCCUCUGUAAAGUCACUUUCUUCUGAUGGCCAGUGUCACUAUGAUGUCAGUGAGGUCUGGGGAUGAGGACAGUGUGUCCUGAAAUUCACAGGACUGACUCCUCACCCCAGUGCACGAGGAUUCCUGUGGCAUCAGGUGCUGCUGUACCUGGUGUAGGAGCCUAAUCAUUGAACCAUUGUGUUACU*

*>hsa-mir-4748:19:10890867:10891068*

*UGUCAGGGCUAAAUUCAAAACCCUGUUUAGCUGUGAAGAUCCGGGGCUGAGAAGCAAAUCUGUGGCUGGCUGAGGUUUGGGGAGGAUUUGCUGGUGCUAGAGAGGAAAGCAGACCCUACCCAACCCCACGCCCUACUACAGCCACCCCGACCCGGCUGCCCUUGGACACUGUUAGGCCUCCAUCUUGGAUCCUAAGGGCC*

*>hsa-mir-4425:1:25349935:25350136*

*CAGAUGCUCAGUAGGUGAGAUUCGUGGUAAGAUUUAUUGAGCAUUUAUUGUGUGCUAAGUGCUUUACAUGAAUGGUCCCAUUGAAUCCCAACAGCUUUGCGAAGUGUUGUUGGGAUUCAGCAGGACCAUUCGUGUAAAGUAAUAUUCCCAUUUUACAGAUGAAGAAACUGAAGGAUGGGGAGGGGGUCAAGUAAUUUAUA*

*>hsa-mir-4733*

*UAGACUGCUGAGAAACAAAAAAGGAAGGCCAAAGAGAGAUUCCGUGAUUUUUUUUUUUUAGAGGUCGCUUAAAUCCCAAUGCUAGACCCGGUGGCAAUCAAGGUCUAGCCACCAGGUCUAGCAUUGGGAUUUAAGCCCAGCUUUCCUCUGCAAAACUCAUAGCCAUCCAAGUUAAUAUAACUAUUGCACAGUUGAUGAGA*

*>hsa-mir-1273a*

*UUAGCCGGGCGUGGUGGCGCUCGCCUAUAAUCCCAGCUACUCAGCGGGCUGAGGCAGGAGAAUUGCUUGAACCCGGGUGGUGGAGGUUGCAGUGAGCCAAGAUUGCGCCACUGCACUCCAGCCUGGGCGACAAAGCAAGACUCUUUCUUGGAAAAAAACAAAAAACAACAAAAAAAGGGAGCUCUUAAUAAAUUGUAUUG*

*>hsa-mir-4746:19:4445911:4446112*

*GAUUCAAACCCAGGGACCUGCCCAGGCCCCCUGCUCUGAGAACAAGGAGGCUCCCUCCUGGGGGUGUCUGUGCCGGUCCCAGGAGAACCUGCAGAGGCAUCGGGUCAGCGGUGCUCCUGCGGGCCGACACUCACCAGCCCGCACUCGUUCAAGGCCAGGUUCUCGUCCUCGGACAGCUUCUGCCCUCCCGAGGCCAGCAG*

*>hsa-mir-4658*

*CCCCUUUGUCCCUCCUGCAGGGCCUGGAAGUUCCCCUGAUUGCUGUGGUUCAGUGGUCUACCCCAAAGCUGCCCUUCACUCAGAGCAUCUACACCCACUACCGGUGAGUGUGGAUCCUGGAGGAAUCGUGGCGUUCGCCUUUGUGGCCUGACACACACAGCCAGCAUCCCUCUCUCACCACUCCUAGCCUGCCCAGUGUC*

*>hsa-mir-5572:15:80873414:80873615*

*CACACCGGGCUGAUCCCAGGCCUGUGCCUAGCCAGACAAGAGGGUCAUGGGGAGUCACUGUCAACCCAGAGCAGGCACUGCCCCUGCGACCAGCCUGGGGCAUCGGUUGGGGUGCAGGGGUCUGCUGGUGAUGCUUUCCAUCUCUUUGCUUUGUCCUGAUUGUAGCAAAUCCCAUCUCAGUCCAGCAAGACUCAGUCAUC*

*>hsa-mir-4802*

*AUAUGUCAGAAACAGUUACAAAAUGCUCAAGCUUGGCCUCUGUAAAUUCAUUCUAUUGAGCUGACUGGCUUGUAUGGAGGUUCUAGACCAUGUUAGUGUUCAAGUCUACAUGGAUGGAAACCUUCAAGCAGGCCAAGCAGGAGACAGGUGGAAGAAGAUGUGUGCAUUUGAGGGCCUCGCUGUGGGGCUUGUGGUGGUGU*

*>hsa-mir-4769:X:47446766:47446967*

*GGUGGCCCUGGCUCCCCAGCCAUGGCUGCAGGACAUUCCUUGGAAGGGACUGUUUGGGGCAGAGGAGAGGUGGGAUGGAGAGAAGGUAUGAGCUAAAAAUCCCCAAGCUCUGCCAUCCUCCCUCCCCUACUUCUCCCCUCACUCCUCUGACCAACUUGGAAGGAAAUACUGACAGCAAUGAAAUGCAGGUAGGGUUCAAA*

*>hsa-mir-1295b:1:171070809:171071010*

*UUCUAACCCUGAUCUAGAGCCACGAUGAUGUAUUUCCCUCACCUUCUUUGGUCACAGAAGGAGGACAUUUCACCCAGAUCUGCGGCCUAAUCACAGGCCACAUUUCUGAAUAGGCCACGGAUCUGGGCAAAAUGUCCUCUUUUCUCAGGAGCUCCCCAAACAAUAAAUCAGUAAAUCAUAUGAAUUUUUGCAUGAAGUUU*

*>hsa-mir-2467*

*AUCGACUUCUGUCUUGCGUCCACAUGAGAGAGAAGACGGUGGCCACUGGACAGACGCUUGGACAGGCACCUGAGGCUCUGUUAGCCUUGGCUCUGGGUCCUGCUCCUUAGAGCAGAGGCAGAGAGGCUCAGGGUCUGUCUGGGUCACUCUCUUUGAGAUUGCCGGAACGCUUACACACAUCUUUCCCUGGGUGGAAUCGU*

*>hsa-mir-5696:2:101925856:101926057*

*AUAGUAUUUUAUGCAUAAUCUAUAAUUUUGAAAUAGGCAUAAGCAUACAACAUAAGUGCUCAUUUAAGUAGUCUGAUGCCUACUACUGAUGACAUACAAUGUAAGUGCUCAUUUAGGCGUCAGACUACCUAAAUGAGCACUUACGUUUCAUGUCAAUAAAAAAGGGAAAAGGAUUUAUUGUUUGGACAUCAAUACCCAGU*

*>hsa-mir-4804:5:72174354:72174555*

*AUGUUCCUAUUUUCAUAUGGUCAACUGAGUUUUUUCUUUCCAUCAAGAAAGCAACGAUUCCAGUCAGUGUAUUUGGACGGUAAGGUUAAGCAAGGUGCGUCGUAUCUUGCUUAACCUUGCCCUCGAAAUACACUGACUGGAUUUUUUUCUCUUUAGGAAAUCUAUUAAUUGUUCUUCCUUUUCUCUUUACUUUGUAAUGC*

*>hsa-mir-4791*

*CACCAGAUAUAAAGCAAUACCACAACAACAGUCUUAUUAACUCAUAGCUAAUACAAUUUAAGAACUGGAUAUGAUGACUGAAAUAAGCUCCAUAUCAAUGAGAAUUUCAAUGGGAUUAUGUGCAGUCAAUGUCCAGUAAUUAGAAAAGCUAUCCAGCUAUUCAGUCCAAUGCUAUUUUCAAUAUGGAAUGAUCUGGGAUU*

*>hsa-mir-548aq*

*GAAAUAUUCCUAGAAGUAUAAUUGGAUUAAAGAGUAAUAUAAGGAUUAAGACACUUGCUAUUUAGGUUGGUGUGAAAGUAAUUGCUGUUUUUGCCAUUACUUUCAGUGGCAAAAACUGCAAUUACUUUUGCAUUAACCUAAAAUAAUCAUUGCCAAUGCUUUCCAAAAUGGCUGCACUAAAAUAUAUCCCCUGAACAGUA*

*>hsa-mir-4470:8:62627283:62627484*

*UGCCGCGCUCCCCGCCCCUGCUCACUUAGGGCUGUUCGGGCGCUACGCAGCUGCCUGUGAGCGCGAGCCUCUUUCGGCUUUCCAGUUUGUCUCGGUCCUUUGGAACGUGGCAAACGUGGAAGCCGAGAGGGCUCUCGCGAGCGUUGGCAGACCCCGCUCCAUAGGGAUGGGACAGGGCGCGGUGCGGAAUAGGUGGACAC*

*>hsa-mir-4674*

*AUCCUUCCGCAGCCCCGGCUCAAACUUUUGGCCUCUGAAAACUUUCAAACGAGAAGUAGUCCCAGGCGCCCGCUCCCGACCCACGCCGCGCCGCCGGGUCCCUCCUCCCCGGAGAGGCUGGGCUCGGGACGCGCGGCUCAGCUCGGGGAGGCGCAAAGGCGGACGGGGCGUGCGGGAGGAGGUGGCCGCGGAGGGGGCGG*

*>hsa-mir-4712:15:50652467:50652668*

*CCGAGGCUAAAAUAGGUUGAAUAACUUACCUAAAUUCACCAUCCUCAAAGGUGUUAAAGACAGGAUUCCAGUACAGGUCUCUCAUUUCCUUCAUGAUUAGGAAUACUACUUUGAAAUGAGAGACCUGUACUGUAUCUGUUAAUUCCAAAUUCCCUGGCAUAAAGACUCAGUACAUUGUUUAAAGAGAAUGCAUGAAGUUA*

*>hsa-mir-1268b:17:78072550:78072751*

*AACAUGGCGAAACCCCAUCUCAACUAAAAAUACAAAUAUUAGCCAGGCAUGGUGGCGGGCGCCUGUAAUCUCAGCUACCCGGGCGUGGUGGUGGGGGUGGGUGCCUGUAAUUCCAGCUAGUUGGGAGGCUGAGGCAGGUGAAUCGCUUGAAGCCGGGAGCCAGAGGUUGCAGUGUGCCAAGAUUGUGCCAUUGCACUCCA*

*>hsa-mir-4761:22:19951216:19951417*

*GAGAUCAACCCCGACUGUGCCGCCAUCACCCAGCGGAUGGUGGAUUUCGCUGGCGUGAAGGACAAGGUGUGCAUGCCUGACCCGUUGUCAGACCUGGAAAAAGGGCCGGCUGUGGGCAGGGAGGGCAUGCGCACUUUGUCCUCCCCACCAGGUGUUCACACCACGUUCACUGAAAACCCACUAUCACCAGGCCCCUCAGU*

*>hsa-mir-5587:16:585240:585441*

*CCACGGGUGGGCUGUCAAGGCCAGCACCAGAGACCCCCAGCAGACCUCAGUGGCCGCAGAUGGAGCGGGGCGGCAAUGGUCACCUCCGGGACUCAGCCCUGUGCUGAGCCCCGGGCAGUGUGAUCAUCCUGGCCCUUCUCGUGCACGUCCCCUGGCUGGAUGCUCCUUGCUGCCCUCACGGGGUGUGUGUGUGGCAUACA*

*>hsa-mir-4418:1:22592664:22592865*

*GGUGGGAGGAGAGGAGUCAGCAGAGCCUCUGUGUCACCCAGGGGCCUGGGGGGCCUCCUGGGGGCUUUGGUUUUUGCUCUGAGUGACCGUGGUGGUUGUGGGAGUCACUGCAGGACUCAGCAGGAAUUCUGGAUAGUAAGUGGGAGGUGGGGGACCAGUGAGGAGGCUGUCGCCAGAACCCACUGAGACGUGGUGGUGGC*

*>hsa-mir-5197:5:143059379:143059580*

*UCUGUCACUGCAGAUUGACCCUGAUCUACAGCUAGGGAAGAACUGUAUGGGAUUCCACAGACAAUGAGUAUCAAUGGCACAAACUCAUUCUUGAAUUUUUGCCAGUUCAAGAAGAGACUGAGUCAUCGAAUGCUCUAAAUGUCACUUCACCUCAUGUUCUGUCAACACAGAGGGUAACUCAGAGUGGCCAGUUCCACUCA*

*>hsa-mir-5580*

*GGAGCAGAGGAGAGGCCUUUGCUUUUUCUUACUUGCCACCAACAACUAAAUUAAGCCUUGAAUCUUGGUCUGUGCUGGCUCAUUUCAUAUGUGUGCUGAGAAAAUUCACACAUAUGAAGUGAGCCAGCACAGACCAAGAUCCCUUUGGAUCCCUUUGGAGCAGAAACAUUUAGUGUCUUUGUAUGUUCUCUGUGGGGAAG*

*>hsa-mir-4460*

*CAUCUCUUCUGAUUCACCUUGAUAAGAAUUAACAGACAACUCUCACAGAGAGGCACUGUUUUUUGCCCAUAGUGGUUGUGAAUUUACCUUCUCCUCUUUGCAGUGAUAAAGGAGGUAAAUUCACAACCACUGUGGGCAGAAACCAGGUGAAUCUUCAGAACCUUGCUGGAUAGAGGUUUGCUGCGUUGUCUCACCAGUGU*

*>hsa-mir-4781:1:54519689:54519890*

*CGUCACUGAGCCUAAUGUAAUCCUAACCCUUUCACAGCCCAAUCUUAUUUGGAAGAGGGCUUAGGUGCACGCUCUAGCGGGGAUUCCAAUAUUGGGCCAAUUCCCCCAAUGUUGGAAUCCUCGCUAGAGCGUGCACUUCUGGAAGCUAGGAACCUCCUAACUCAGGACGUAGACUGAUGAUUGAUUACAGUUUCAAUUCU*

*>hsa-mir-4464:6:91022407:91022608*

*CAGGAACAGCAAAUAAAAAUUGCUUCAAUCUAUUAUAAGUUUAUUAAAAAUCAGGAACCUUAGUAAGGUUUGGAUAGAUGCAAUAAAGUAUGUCCACAGCUGAAAGGACAUACUUUAUUGCAUGUAUCCAAACCUUACUAAUUCAGAGUUUUGUGCUGCGUGUAUGUGUGUGUAGUAGGAGUAUGUGUGUGUGUGUUUCA*

*>hsa-mir-4684:1:23045950:23046151*

*CACAUUCCCACAUGGCCACAGUUGGCUGGAGUGACUGCCUCUUAAGACAGAAUGUGUGAGCACCAGGGGUACCUCUCUACUGACUUGCAACAUACAUUUGUCUUGGUGUGUUGCAAGUCGGUGGAGACGUACCCUUGGUGCUCAACCCCUGUAAGGGCUUGAGUUUGCCUCCUGGAUCUGCCCCUGUUACCCUGUGUGAC*

*>hsa-mir-4493*

*UAAUUUGCUUAUAUGCAUGCCUAAGACCUGCCAUUCAUAUAAAGACCGGGCAUACAAGAAUCUCAACCAGAGAUGGGAAGGCCUUCCGGUGAUUAUCACAGCCAUGCCUUUACCUCCAGAAGGCCUUUCCAUCUCUGUCUAAUAAGAGGAAGACAAUGCUGGUAAUUUGGGACCCAGCUCUCCUAACCAGGACUUGUCUC*

*>ENSG00000252972:69659868:69660068:15:1*

*GCUGCCCCCUCUGCAACGUGCAGCCUCCAAGGUCAUCCCAGAAGGGGAAGCCAGGGUCUACAGCAAUACCACCCUGAACACACCUAUCUUGCCUGAUCUCAGAAACUAGGCAGGGCUGGGCCAGGUUCAAGCUUGGAUGGGAGAAGAAGAAGAGAAAGUUAGUUUCCCUAGAAACAGACUUUGAGAAAAAGAUUCGGGCA*

*>ENSG00000212237:228783627:228783827:1:-1*

*GCCUUUGGGGCAGACAGGCUGCACUACCGGCGUCUAAGGCCAUACCACCCCGAAGGCGUCCGGUCUCCUCUGAUCUCGUGAAGAUAAGCAGGGUCUGGGCCAGACCGCUACUUAGAUGAAACGCCACUUGAGAAAACCAGGAGACGCAGGCUUGUUUAGACUUCAAGUUCCUCCCUCUUUACCGUUUGGUCGCCGUACUU*

*>ENSG00000202430:24787434:24787634:2:-1*

*UUAAUUUAUCUCUUAUAAUUGACAUGCUUUCCUAAUUUUAAUAAAUAUAUCCCUUUAAAAUCUUGUCUACAGCCAUACCACCCUGAACACACCUGAUCUCAAAAGCAAAGCACAGUCGGGCCUGGUUAGUACUUGGUUGUGAGACCACAUUGGAAUACUGAGUACUGCAGGCUUUUGGAAAAAAAAUCUGGAUCUUUUCU*

*>ENSG00000252368:37730224:37730424:1:-1*

*UUUGUCACUAUUUUGAUUGUUAUUCCCAUUUUGCAGAUAGAAGACUGAGACUAGUCUAUAAUUAUACCACUCUGCACGUACCUGAUCUUGCCUGAUCUUGGAAGCUAAGAGUUAGGGUCAGGACUGGUUAGUACUUGGAUGGAGAAAACCGAGGCUGAAGAGGUUUCUCAAGGUCACACAGCACAAAUGGUGGAGCAGGG*

*>ENSG00000252591:18082770:18082970:19:1*

*GAGUUCCAGACCAGCCUGGGCAAUGUAGCAAGGCCUGAUCUCUACUAGAAAUAAAAAUAAAUCAUCUAUGGCCAUACCACCCUGAACACUCCCGAUCUCAUCUGAUCUCGGAAAAAUAAAUCAUUAGCUGGGGGUGGUGGCGCAUACCUGUAGUCCUUGCUGCUUGGGAGGCUGAGGUGGGAGGAUCGCUUGAGCCCAGG*

*>ENSG00000252060:12107549:12107749:19:1*

*CGGAGCAAGACUCCGUCUAAAAACAAAAACAAAAAACUUUUGUGCUUCAAAAAACACCAUCAAGAAAUUGAAGCCUGUGUGGCCAUGCCACCCUAAACGCAUCUGUUUGGCAUAGCUAAGCGGGAUUGGGCCUGGUUAGUAAAUGGAUGGAGAAUGCCUGGGAAUACUGGGUGCUAUAUGCUUUGAAAAAAAGAAGAAGA*

*>ENSG00000252637:68011453:68011653:8:1*

*UGUCCCUUUGUGACAAAACCCAACAUGUUAAGGUGUGGUAAGUGAACUUAUAUAUUAAAAGCCAUAGAAUAUUUAGAUAGAUUUUCUAUGGCUAUACCACCCUGGAAUGUGCCUGAUCUUGUUUGAUGUUGGAAACUAAGUAGGCUUGGACCUGGUUAGUAAUUUGGUGGGAGAGUAUUUAGAUAGAUUACUUUUGGGUC*

*>ENSG00000201790:105258625:105258825:5:-1*

*ACCUAUAAUUUUAAAAUAUUACUUUUUAAAUUCAUACAUUUAUAUUCUGAAAAAUAUAGACUAGUCUACAGCCAUGCCACCUUGAAUGCACCUGAUCUCAUCAGAUCUCAGAAGCUAGUUAAGCAGUGAUGGGCCUGGUUAGUACUUGGAUGGGAAAAAUAGAGAACAUAUAUUUAAAAAGCUUAUUUAAACUCUCAACA*

*>ENSG00000207129:87570033:87570233:5:-1*

*AUGAGGUAGAAAAAAUGGAAUAAUUAAAAAUAAUCUAAAAGGUCUAUAGCCAUACCACCCUGAAUAUGCCUGUUCUCAUCUGAUCUGGGAAGCUAAGCAGGGCUGGGGCUGAUUGGCACUUGGAUGAGAGUCUGCCUGGGAAUAUCAGGUGCUAUGGCCUAAUUAAGAAAAAAAAAAAUCAAAAAGGCAAAAGAAGACAA*

*>ENSG00000200558:68776316:68776516:16:1*

*GGACUACAGGUGUGUGCCUCCAUGCCUGGCUAAUUUUUGUAUUUUUAGUAGAGACAGGGUUUCGACUACGGCCAUACCACCCUGAACGCGCCCGAUCUCGUCUGAUCUCGGAAGCUAAGCAGGGUCGGGCCUGGUUAGUACUAGUACUUGGAUGGGAGACAGGGUUUCACCAUGUUGGUUAGGCUGGUCUACAAUCUGGA*

*>ENSG00000251746:178003352:178003552:2:1*

*UUUCUUUUCUCAUCAAUCCCCAAAAUGUACAAUGGUACUUUAGUGCUUCCUCUAUGCCAAAUCAUAGAUAUCCAAUAUAUGGCCAUACUGCCUGAUCUCAUCUAACCUCAGAAGCUAAGCAGGAAUGGUCCAGUUAAUAUUGGAUGGGAGACCACCUGAAAAUGCCAUGUAUAGGCUUUUAAAAAACAAAGCCGAGAUAC*

*>ENSG00000212542:31701331:31701531:22:1*

*CAUCACCGCUAUCUAAUUCCAGAACACUUCAUCACCCCAAAAAGAAACGCUGGUACCUGUCUACAGCCAUACCACCCUGAACAUGUGCAAUCUUGUCUGAUCUCAGAAGCUAAGCAGGGUUGAGUCUGGUUAGUACUCAGAUGGGAGAAACCUAGUACCUGUUGGCAGCCACUCUUCAUUUUCCUUUCUCCUCACCCUCU*

*>ENSG00000222459:110681101:110681301:9:-1*

*UCUCUACUCACAUGUUACCACCUCUUCUGAGUGGCACUUCCACGUGCCUGCAGCCAUGCCUCCUUGGGCAGAGAUCCCAUCAGAUUUUACAAGCCACGCAGGGUCAGGCUUGGUGAAAACGUGAAUGGGAAUGCCCAGAACAAUAAAGGCAGCAGGCGGUUUUCCUUGGGAGACGGGGGAGAGCCAAGCCACGUGCUGGC*

*>ENSG00000252745:142310428:142310628:3:-1*

*AUAUUUAUUUCAUUUGGCUGGUUUUUUUUUUCCAGAACAAAGAGAGUAGAGAAAAAAACAUAUGUGAGAAAGACUAAGAUUCAAUAUUCAGUCUGUGGCCAUACCACCCUGAACACUCUCGAUCUCGUGUGAUCUCGGAAGCCAAGCAGGGUCAGACCUGGUUAGUACUUGGAUGGGAGAUUCAGUAUUCAUUUCAACUC*

*>ENSG00000212289:27543113:27543313:11:-1*

*GGUGGGCCUAGGGUAGAAUGCUUGAGAUUGAGAUUUUGAUCUAUGGCUAUACCACCCUGAACAUGCUUGAUCUUGUCUGAGCUCAGAAGCUAAGCAUGGUCGGACCUGGUUAGUUCUUGGAUGGGAGAUGGAGAUUAUGGAAGAUGAGGAGUUAUUGAUAAGGACAAAGUUGAGGGGAGGCUAUGAAUGGCUCAUUCCAU*

*>ENSG00000201931:178378210:178378410:4:-1*

*AGAUACAAGGUUGGUUUUCCAAUCAGUAGCUGUGGGAUAGAAUUAAAUAAAUUAGUCUAUGGCCAUACCACCCUGAACAUGCCUGCUCUCGUCUGAGCUCAGAAGCUAAGCAGGGUCGGGCCUGGUUAGUACUUGGAUGGGAGAAUUACAUAAAUUAGUUUUGUUUUUGUUUUCUUCAGACAGAAUCUCACUCUGUUACC*

*>ENSG00000212365:14156473:14156673:11:1*

*AUGCUGAAUUAGUUAUGACUCUCCAAACACACAAUGUUAUUUAAUGUUUCUGAGUCUGGUCUAUGGCUAUACCACCCUGAAUGCACCCAAUCUUGUCUGAUCUCAGAAGCUAAGCAGGGUCAAGCCUGGUUAGCACUUGGAUGGGAGUCUGCCAGAACAGUCCUUUCUACUCUAUUUUGCCUUACAAAUAUGAUCAGCCU*

*>ENSG00000200719:28905931:28906131:8:-1*

*AUCUUGGGGUUCUAUAGAGCAUAUGAGAACUGUACUGAUGAAUAAAAAGUGUAUUUACAAAGGAAGGAAAUGAAAUGCAUUCUAUGCCCAUACCACGCUCAAUGCGCCCGAUCUCGUCUGAUCUUGGAAGCUAAGCAGGGUCAGGCCUGGUUAGUACUUGGAUGGAAGAAAUGAAAUGCAAUUUUAGAAGCUAAAAACCA*

*>ENSG00000251760:34987117:34987317:16:1*

*GAGUCUCUGGCUCUCCCUUUUGCCCCGCCUGAUCCCCCACCGUGGCCCGGUGCCUCCUUGUGCAGAUCUACCCCGGCAGGCCAAAGUGCCAUAGUCGCCUGAGGGCACCCCAUCCCGGAAGCUAAGCAAGGUCAGUCCUGGCUAUCGCUUGGAUUGGGGACCACCUGGAGAGACCAGGGACUGGAAGCUUUUGGCUUCCU*

*>ENSG00000251705:10037712:10037912:Y:1*

*CCAGGAGGGCCUCCCAUUGUUGGGAGCGCCCUUGCCAAAUUGACCUUGUAUGACUUUUAGCGGUGGAUCACUCGGCUCCUGCGUUGAUGAAGAAUGCAGCUAGCUGUGAGAAUUAAUGUGAAUUGCAGGACACAUUGAUCAUCGACACUUCGAACGCACUUGCGGCCCGGGUUCCUCCCAGGGCUAUGCCUGUCUGAGCU*

*>ENSG00000252267:26786572:26786772:22:-1*

*AAAGGUGAAUGAAUUCUCAUGCGGGAUCAUGGCAUGAAAACAUAUAAUUGUCCAUAAGGUCUUGCCACUGUAAAUGUGCCCGAUCUUAUCUGAUCUUGGAAGCUAAGCAGGAUCGGGCCUACUUAGUGUCUUGGAUGGGAGACUGCCUAGGAGUACAAAAGCCUACAAGUGCUGUAGGCUUUUGAAAAAACAAAACAAAA*

*>ENSG00000251953:20507934:20508134:Y:-1*

*GAGUGGCGGUAAUGCCUGGAGUCUGGCGGGCAUGGUGGGCCAGGCUCUUGUGUCAGCCAGGUAUCACUGCGGGUGUCUAAGGCCUUACCGCCCUGAGACACCUGAUCUCUGGCCUGUGACGCUAAGCAGGGUGGGGCCUGGUUCAGUACUUGGAAGAGAGACCGCCUGGGAAUACAAGGUGCUGUAGGCUUUUGGCUUCC*

*>ENSG00000212276:109605980:109606180:9:-1*

*UGUUGUUUCAGGCACUGUACUAACUCCUCAUAGAAACUAUUUCAAUUCUGUCUGUGGCUACACUACCCUUAACGUACUCGAUCUUAACUGAUCUUGAAAGCCAAGCAGGGUCAGGUCUGGUUAGUACUUGGAUGGGAGAAAUUAUCUGAGUUCAAACAUCACAAAAAAUCUGAAAGCCAGCUACCAUUAUACACAAUUUA*

*>ENSG00000252179:13393489:13393689:8:1*

*UGGAUGUUGUACAGAGGUUCAUCUUAACACCAAGGAGAACAACAAAGAGUCUACGGCCAUACCACCCUGAACGUGCCAGAUCUUGGAAGCUAAGCAGGAUUGAGCCUGGUUAGUAUUUGGAUGGGAGAACAGCAAAGAAUGGUGAACAGCAGCUCCCCCUUGUAUACUGAUGACAACAAGCUAAGGAUAAGCUGUAGAUC*

*>ENSG00000252624:34969936:34970136:16:1*

*GCCCGCCUUCGCUUCCAAAGCGGCCUGGGAUCUCCCUGUUGGGGUUCGUCCCAGCAGCACCCAGGGGCCAUAGCAGCCUGAGGGCACCCCAGUCUGUAAGAAAAGCAGGGCUGGGCCUGGCUGGGUCUUGGAUAGGGGACCCCCUGUGAAGACCAGGGGCUGAAGGCUUUUGGCUUCUCACUCCCUUCCCACUUUCUCCU*

*>ENSG00000252866:128337679:128337879:7:1*

*UCCCCCUUUUACUCCGUCGCAGCCCGGGACCUCCUGGUGGGGGUCCGCCACUGCAGCACCAGGCGCCACUGCCAUACCACCCUGAACGCGCCCGAUCUCGUCUGAUCUCGGAAGCUAAGAUGUGGCCGCAUUUAGGACCCCACCGGCUUGGCUGUGCCUCUCCCAGCCCCCUGGCGGAGCGGACUAACCCUAGCAUUUCA*

*>ENSG00000201532:138347968:138348168:5:1*

*CCUGGGCUCAAGCAAUCCUUUCACUUUGGCCUCCCAAAGUGCUUUGAUUAUCUAUGACCAUGCUACCCUGAAUGUGCCUGAUCUCAUCUGAUCUUGGAAGCUAAGCAAGGUUGGGCCCGGUUAGUACUUGGAUGGGAGACCAAAGUGCUGGCAUUACAGGCACGAGCCACUGUGCCUGGCCCGACAUCCUAUCUUUUACC*

*>ENSG00000252041:24172285:24172485:7:1*

*GUAAGGACAUUACUACAGGAGCAUUAGUAAGGGGAUAUUAUCACAGCUAUGCUGCCCCGAACAUGCCCCAUCUCCUCUAAUCUCAGAAGCUAAGCAGGGCAGGGCCUGGCUAGUACUUGGAUGGGAGAAAGGGAACAUCAGAGGACUUGCAGAGUCAUGGUAGUGAGAUGAUUUUAACCCCUUCUGCUUUGGGAAGGGCA*

*>ENSG00000222208:37383053:37383253:3:1*

*GCUCACUGCGGAGAUGAUUCUUGCUGCAAAGAUUAUUUACUAUAGCUAAGUCACUGAAUAUCUCAGCAUAGAACUAGACUGAAUCAGCCUACAGCCAUACCACCGUGAAUGCUCCCAAUCUCAUCUGAUCUCAGAAGUUAAGCAGGGUCAGGCCUGGUUACUACUUGGAUGGGAGAACUAUACUAAAUCAAUACUCAGCA*

*>ENSG00000199202:98667248:98667448:9:1*

*UUUUAGCUUCAGAAUAAGAAUUAUCACCCUUCCCCCAUGUCUACAGCAAUACCACUCUGAACACACCACAUCUCGUCUGAUCUUGGAAGCUAAGCAGGGUCGGACCUGGUUAGUACUUGGAUUAGAGGAAUAGCUCCCAGAGUUGCUUUGUGUGUAGUGACUUAGCAGUUGGACACUGCUUUCCAUUUUAUUAUUUUGGC*

*>ENSG00000222675:156891669:156891869:3:-1*

*UGAAUGAAUAUAAAUAAAUUAUAGGUAUAAAAACAUGGUCCACAGCCACACCACCCUGAACUUGCCCUGUCUUCUCUGAUCUUGGAAGCUAAGCAGGGUCUGACCUGCUUAGCUAGUCCUUGAAUGCGAGACCACCUUGAAACACUGGGUGCCGCAGGCUUUUGAAAAAUAAAAUAUAUAUAUACGUUUGAUCAUUCCCU*

*>ENSG00000212258:7047265:7047465:5:1*

*ACAUUCAACCUUACAGUAUGUAAAGAAAAAAAAUAAUUUGCAGCUUUCAUACCUUCUUAAAAAUAAAAUUUUUGGUCUACUGCCAUACCACUUUGAACGUGCUUGAUCUUGGAAGCUAAGCAGGGUCAGACCUGGUUAGGACUUGGUAUGGGAGACCACAUGGGAAUACCAGGUGUUGCAGGGUUGAUAUAAAAUCUAAA*

*>ENSG00000222418:182913486:182913686:2:1*

*AUAGUUGUAAUCCAAAGGUCGCUGUAAAGAAAGAGAUUCUACCUUCAAACAUGUUAGAUACAGUCUACUGCCCUACCACCCUGAAAGCGCCCCAUCUCAUUUGAUCUUGGAAGCUAAGCAGUGUUGAGCCUGGUGAGUAGUUGGAUGGGAAACAUGUUGGAUACAAAAAUGGAGAAUCUGCUGAAAUGUGAUAAUUGAGA*

*>ENSG00000223293:70842065:70842265:8:1*

*UCAGAAAAAAAAAAAAAAAAAGUAAAGUUACAGUAAGCUAAGGUUAAUUUAUUAUUGAAGAAAGAAGAGUAUUUGCCUAUGUCCAUACCACCUUGACUGUAGCCAGUCUCUUCUGAUCUCAGAAACUAAGCCAGGUCAGGCCUGGUUAGUACUUGGAUAGGAGAAUCCUAGGACUACUGAGUGCUGGAGUCUUUAAAGGA*

*>ENSG00000202472:140086537:140086737:7:-1*

*CAAAUAUCGGUUGAUAAUUGUGUAUUCAGUAUUCAUUAAUAGAGUCUACAACCAUACCUGCAUACCACCCUGAACAUGGCCUGCCUUGUCUGACUUUGGAAGCUAGGUAUGGUGGGGUCUGGUUAGUGCUUGGGUGGGACACUGCCUGGGAAUACCGGGUGCUGUAGGCUUUAAAAAAUAAAAAUUCACCGAUUGAGAAA*

*>ENSG00000201523:19438449:19438649:6:1*

*AUGGUAUGCAGACCUCUGAAACCACCUAAAAUAGUACAUUGGGGAAUGGAAAGAAAGCAUUAGAGUCUACGGCCAUACCACCCUGAAUGCCCCCGAUCUUCUCUGAUCUCAGAAGCUAAGCAGGGUUGGGCCUGGUUAGUACUUGGUAUGGGAGAAAGCAUUAGACAUCAAUCUUGUAAGAAUUUAAAGUUCAAUAGAUU*

*>ENSG00000201812:15443139:15443339:21:1*

*AGGCGGGCGUGGCCGGGCCCGGCUCUUGUGUCAGCCAGGUAUCACUGCCGCUGUCUAAGGCCUUACCGCCCUGAAGACGCCUGCUCUCCUCUGGUCUGUGAAGCUACGCAGGGUGGGGCUUGUUUCAGUUUGAGGGAGACCGCGUGGGACUACUGGACGCUGUGGGCUUUUGGCUUCCCGCUCUCUCCCUCUUUCCCCCU*

*>ENSG00000199395:56462265:56462465:2:-1*

*UUAUUUUUCAAAGUACAUCUAACGGUUGCUAUUUAAUCACGGCUCUGGUUGGUUCUACAGGAAGUGUAUAUAAAACUACCUAUGCUGUCUGCGGUCACACCACCCUGAAUGCACCAGAUCUCCUCUGAUCUUGGAAGCUAAGCAGGGUCAGGCCUGCUUAGUACUUGGAUGGGAGAAAACCACCUAUGCCUUUGUUAACA*

*>ENSG00000212242:136951323:136951523:6:1*

*UGGUAUUUUACUUAUUUGUGUUUAUUGUCCGUCUCUCAUUAGAAAGUAACCUGAGUCUACAGCUAUACCACCCUGAAUGCGUCCAAUCUCUUCUGAUCUCGGAAGCUAAGCAGAGUUGGGCCCGAUUAAGACUUAGAUGAGAGCAUGUAAGCUCUCAGUGAUAGCAGGAAUUUCUCUCCAUUUUGUUCUCUGCCAUCUCU*

*>ENSG00000201846:149479034:149479234:4:1*

*UUGCUCUUGGGGGGAAAAAGGUGAAUGUAGUUAGAUGAUAGUGAAUAUGAGAGUUGAAGCUUCUGUCUACAGCCAUACCACCCUGAACAUGCCCGAUCUCAUCUGAUCUUGGAAGCUAAGCAGGGUUGGGCCUGGUUAGUGCUUGGAUGGGAGACAGUUGAAGCUUCUGAGUUAAGAAGGUAAGAGAAGAGAAAUAAGUU*

*>ENSG00000222608:52694220:52694420:X:1*

*ACUGCACUCCAGCCUGGCAACAGACAGAGUGAGACUAUGUCUCAAAAAAAAAAAAAAACAGUCUAUGGCCAUACCACCUGAAUAUGCCAAAUCUUGUCUGAUCUCAGAAGUUAAACAGAGUCAGGCUUGGUUAGUACUUGGACUGAAGUCCAAUAUAAAAAUGAAAAAUUAUAGGACUAAGACUGGGGGUAAAAACACGA*

*>ENSG00000212265:55556107:55556307:5:-1*

*UUUCCAUGGAGUAAAUCUUGACCAUUGGGAAACAGGAAAUGAAAGAGAAUCAGAGUCUGGCCAUACCACCCUGAAUGAGCCCAAUCUCGCCUGCUCUCGGAAGCUAAGCAGGGUGGGGCCUGGUUAGUACUCAGGUGGAAAAAAGAGAAUCAGGCAGACAUAUGUCCUUCCUUUCAUCCAUUCAUAGUAUGGCUUCUCUU*

*>ENSG00000251829:15685586:15685786:17:1*

*AUAUAUAGGCUGGUCUGUCUUUUUCUUAUUGAUUUGUAGGAGUUCCUUAUGUAUUCUGGAUAUGAGCCCUUUAUGGCCAUACCACCCUGAAUGUGUCUGAUUUCAGAAGCUAAGCAGGGUCUAGCCUGGUUAGUACUUGGAUGCAUAUGAGCCCUUCAUCAGUUACGCUUGUUGCUCUAUCUUCUUUCCCUCUGUGGGUU*

*>ENSG00000199839:181540595:181540795:3:-1*

*UAUUGUAUAUUUCAAAAUGGCUAGAAGAGAAAAUUUGCAAUGUUCCCAACACAUAGAAAAAAUAGUCUACGGCCAUACCACCCUGAACGCGCCCGAUCUCGUCUGAUCUCGGAAGCUAAGCAGUGUCGGGCCUGGUUAGCACUUGCAUGGGAGACCGCCUGGGAAUACCGGGUGCUGUAGGGUUGGCCGGGCGCGGUGGC*

*>ENSG00000238379:129202589:129202789:2:-1*

*CGGUAUCAGCUUUCCUGCCCUGAGACAGUCAACAACCUCACUAUAGUGACUUAAUCAGAGAUACAGUCUGUGCCAUACCAUCCUGAACAUGCCUGAUCUCGUGUGAUCUCAGAAGCUAAACAGGGUUGUGCCUGAUUAGUAUUUGGAUGGGAGACCACCCGGGAAUACUGGGUGCUGUAGGCUUUUGUAAAAAAUAAAAA*

*>ENSG00000202147:6038162:6038362:11:1*

*GAACUUGAAGACAGGCUUUUGAAAAUAAUUCAGUCAGACCAAAAAAAAUAAUAAUAUUUUUUAGUCUAUGGCCAUACCACCCUGAAUGCAUCCAAUUUCAUUGGAUCUUGGAAGCUAAGCAGGGUCAGGCCUGGUUAGUACUUGGAUGAGAGACCUCCUGGGAAUAUUGGGUGCUGUAGGAAAAAAAAAAAUUCUGAAAG*

*>ENSG00000251781:30459311:30459511:12:-1*

*AUUGUGUGGGUCAGUAGAAGAGUGCAUGUAAUACAAGGAUUCAUGAAAUUGGUCCACUGCCCAUAUCACCCUGAACGUGCCGGGUCUCAUCUGAUCUUGGAAGCUAAGCAGAGUCAGGCCUGGUUAGUCCUUGGAUGGGAGAGUAAUGGGAUUCAUGAAAUUGGAACACCUGCGAGAGUACUCCAGUGUACGAUGCUGCA*

*>ENSG00000200473:69472802:69473002:X:1*

*UGCUUCUCACACUCGAAGGUUUAUUCUUUGUCAUGAUCUUAACUAUCACAUCUAAGCAGCAGAUUCAAAUAUUGAGUUCUGGUUCCAUGAUCUAUGGCCAUACCACCAUGAACGUGCCCGAUCUCAUCUGAUUUCCGAAGCUAAGCAGGGUCGGGCCUGAUUAGUACUUGAAUGGGAGCUCUUAUUCCACGUCCAGCUAU*

*>ENSG00000252169:172719432:172719632:5:-1*

*AAACUGAAAACUAAAAAGAUUAGGGUUUCAGGCACAGCUACAUCAGUGUGCUCAAAUGGUGCCAUUAGAAAUCUGCUUUCUCUGUCUACAGCCACACCGCCCUGGACAUGCCUGGUCUUGCCUGAUCUCAGGAGCUAAACAGGGUCAGGCCUGGUUAGUAUGAGAAAGGGAGAAAUCUGCUUUGUCUGUCUCUUGGUUCU*

*>ENSG00000251890:33031037:33031237:22:-1*

*GGAUAGACAGACAGACAGAUAGAUAGACAGACAGAUAGAUAGAAAGAUUAGCCUACAGACAUACCACCCUUAACACACCGCUCUUGUCUGAUCUUGGAAACUAAGCAGGGUCGGGCCUGGUUAGUAUUUGGAUGGGAGAUGUAGACCGAUAUACAACAAGCACAGAGUUGAGAACAUCAUGAGGGUUCUCCCAGUAGACC*

*>ENSG00000222682:106807658:106807858:13:-1*

*GAUUUCCCAUGCUCCCUGUCUUAUUUUUCUGUCUUCUGAUUGAAAUAGAAACCCCGACAUGUGUCUACCACCAAACCACCCUGAACGUGCCCAAUCUCAUCUGAUCUCACAAGCUAAGCAGGGUCGGGCCUCGUUAGUACUUGGAUGGGAAAAAUUCCAGCAAGUACUCGUUUUGUUGGUGCAUAUGUUAUGAUACGUGU*

*>ENSG00000222054:9760961:9761161:5:1*

*GUAGUGCAAUAUGACAAGCAAGGGUUAUACAGAUUGGAAAGAAAGAAAACCCUCUUCGUCCACCGCCAUACCAGCCUGAACGCACCCGAGCUCGUCGAUCUCCGAAGCUAAGCAGGGUUGGGCCUGGUUAGUACUUGGAUGGGAGAAAACUGUCUUCAUUCACAAAUGAUAUGAUUAUCUAUGCAGAAAACCCCCUAAGA*

*>ENSG00000212308:78840780:78840980:1:-1*

*AGAUUCAUUACAAUGACCUAGUAGAUAAGGUACAAGAAUCAUAAGCACAAUAAGAAAUGUUAGUUAAGUUCUAUGGCCAUACCACCCUGAAUGUGCCUGAUCUUGGAAGCUAAGCAGGGUAGGGCCUGGUUAGUACUUGGGUGGAAGAAAUAUUAGUUAAAUUGCCAUGGGAAUUGUGAGGAAGAGGAGAUUGAUUCCUG*

*>ENSG00000201962:25393690:25393890:3:-1*

*CAAAUGGUUUUAAACUUGCUUCUGGUAGACUCCCAUUAUCAGAGGCCUCAAGGCUGUUGCCAAACCCAAGAGAAUCUGAGAUUGGUGUAUGGCCAUACCACCAUGAAUGUGCCCCAUCUCAUCUGAUCUUAGAAGCUAAGCAGGGUUGGCCCUGGUUAGUACUUGGACAGGAGAGAAUCUGAGAUUGUUUUCCUCUCCUA*

*>ENSG00000212559:94677644:94677844:13:-1*

*UGUUGACUUUUCUUUUAAGGAUAUAUUAAUGCCAUUAUGUAAUCUCCAGACAGGCUAUUUUGUAGAUAGAGCCAAAUUUGUCCAAGGCCAUACCAUGCUGAAUGCACCCAAUCUCACCUGAUCUUGGAAGCUAAGGAGGGUUGGGCCAGGUUAGUACUUGGAUGGGAGAUAGUGCCAAGUUUUACCCGUAACUGAGCAGC*

*>ENSG00000252246:33557885:33558085:2:1*

*GCAAAGCCUCUGAGACAGAGAACUUGGUGCACUCAAGGGAGUGGAAAAGGCCAUGCCUGGUUCUGAGAGAGGAGGUGUAGUCUACAGCAAUCCCACCGUAAAUACACCUCUCAUCUGAUCUUGAAAGCUGAGCAGGGUCGGGCCUCAUUAGUACUUGGAUGGGAGAGAGAGGCUCAGCAAGGGUAGAGCUAGGGAGGCAG*

*>ENSG00000200036:166975157:166975357:1:1*

*AUACUUCAAUAUGUGUUUCCUAAGAAUAAGAAUGUUUAGUCUACGGCCAUAGCACCCUGAACACACCUGAUCUUGUCUGAUCUCACAAGCUAAGCAGGGAUUGGCCUGGUUAGUACUUAGAUGAGAGACUGCCUGGGAAUACUGGGUGCUGUAGGCUUUUACUGUGUGAGUGCGUGCGUGUGUGUGUGUGUAUAGAACAU*

*>ENSG00000252587:34989247:34989447:16:1*

*AUCCCCGUUGAGACCUGGGGAUUCCACCUAGGGGUCUGUUCCAGCAGCCAUCAGUGCCAUAGCAGACUGAGGGCACCCCAUACCGGAAGCUAAGCAGGAUGGGACCUGUCUGGUUCUUGGAUGGGGCACAACCUUAGGAGACCAAUGGCUGAAGGUGUUUGGAUUCCUACUCCCUUCCUGCUUUUCCUCACAUGUUGGCC*

*>ENSG00000201671:31451113:31451313:2:1*

*ACAGUUUCCAUCUUCUCCCUUUACUUCUGGCCAGACUCUGGUGUUGUGGAUGAGAAAGGGAAGUGUCUGUAUCCACACCACCCUGAAUGCACCCGAUCUCAUCUGAUCUUGGAAGCUAAGGAGGGUUCGGCCUGAGUUAGUAUCUGAGUGGGAGAAACAGAAGAGAUGGAAAAGUCUUCUGUUUCAUAGUGUCACCCCUA*

*>ENSG00000201518:128570452:128570652:X:-1*

*UCAAACUCGAACUUGGAUUGGAAUCACUUGGAAGACUUGUUAAAACUCAAACUGGUCUAUGGCCAUACCACCCUGAACGUGCCCAAUCUUGCCUGAUCUUGGAAGCUAAGCAGGGUUGGGCCUGGUUAGUACUUGGAUGGAAGACCCCCUGGGAAUACGGGUGCUGUAGGCAUUAUUUAAAUUUAAUUUAAAAAAAAUCC*

*>ENSG00000252553:189635188:189635388:1:-1*

*AUUUAUAGUAUUAGCUCAUGGAAACCAAGUUGUGUGUGGUGGGAAGUAAAGCCCGAGAAGUCUAAAGGCAAACAUAGAGAAGGAUGUUUGUUCUACUGGCAUACCACUCUGAACGUGUCUGAUCUUGGAAGCUAAGCAGUGUCGGGCAUGGUUAGUAAUUGGAAGGGAGAGGGAUGUUUGAUCUGGCAAUUGCUGAAGCA*

*>ENSG00000201527:23141425:23141625:20:-1*

*AGUCUGCAGAGCUGGGUCUGGAAGCUAGGGCAGCAACAUGAUUUAGCAACACAGAAAAUAAUAUUUCUGCCAAGAGCCAUACCACCCUGAACAUGCCCCAUCUCAGCAGAUCUCAGAAGCUCAGCAGGGUCUGGCCUGGUUAGUACCUUGAUGGGAGACUGAGACUAUCAGGUGCUAUAGGCUUAAAAAAAAAUUCCAGU*

*>ENSG00000252231:173890140:173890340:1:1*

*CAGGAGCACACAUUGCAUUUAGUUGUCAUGUCUCUUUAGUCUUCUGCAGCUUGGAACAGUUCCUCUCGUCUAUGGUCAUACUACCCUGAAAGUGCUUGAUCUUAGAAGCUAAGCAGGGUCGGCCCCGGUUAGUACUUGGAUGGGAACAGUUCCUCUCUUUUUCUUUCAUGAUUUUGGCUUGUUUGAAGCAUAUAGGCCAG*

*>ENSG00000202225:120621415:120621615:7:-1*

*CUACCAUUGGAUCCAGCAAUCCUAUACAGGGUAUCUUCACAAAGGAAAAGGCAUCAUUAUAUCAGUCUACAGCCAUACGACCCUGAACACACUUGAUCUCAUCUGAUCUCAGAAGUUAAGCAGGGUCAGGCCAUCAGGCCUGAUUAUUCCUUAGACAGAAGACACAAUUAGUCAAAAAGAUACCUGUACUCCUAUGUUUA*

*>ENSG00000212625:61028904:61029104:15:1*

*ACUCUCUACCUUGCUCAGUCUAAAAAUUUAUACUGGGGAAACAUUCAAUAAACAGUUAUAGGCAUCUACGGCCAUACCUCCCUGAAGGUGCCCGAUCUUGUCUGAUCUUGGAAGCUAAGCAGAGCCGAGCCUGGUUAGUACUUCGAUAGGAGAAUAAAUAGAUACACCUUGGCUAAAGGAACAUAGCGGUGAGAAUUCAA*

*>ENSG00000252816:21405291:21405491:11:-1*

*AGAUUAUAGUUUGCCAUCAAAUAUAAUGAAAGAAGACUUAGAAGAAGCAAUAUGAGUCUAUGGCCAUACCACCCUUAACAUGCCCGAUCUUGUCUGAUCUUGAGAAGCCAAAGGGUCGGGCCUGGUUGGUACUGAUUAGUUAGUAAUAUUGGGUAUUGUAAAAAAAGCAUUUUGAGGGGGGAAAAUCAGGACUAUUCUGG*

*>ENSG00000202063:230955929:230956129:1:1*

*GCCCUGGAAUGAGGCAAGGCAUUUGAUUACAGAGGCACAAAGACAGCUGUUUAAAAGUGGAGCACAGUCUACGGCCAUACCACCUUGAAUGCACCUGAUCUUGUCAGCUACGCAGGGUCUGGCCUGGUUAGUACUAGGGUGGGAGACCACAUGGGAAUACCGGAUGCUGCAGGCUUUAAAAAAAUGCAUAAUGAGCUCCU*

*>ENSG00000200058:134578104:134578304:6:1*

*GCAUUUAUAAUGUGCUAGUUUUAAAGUUGAACAAAACAACAACAACAACAAAGCAACCCACAGCCCGUGUCUACGGCCAUACCACCCUGAACACGCCUGAUCUCAGAAGCUAAGCAGGAUCGGACCUGGCUGGUACUUGGAUGGGAGACUGUUUGGGAAUACCAGGUGCUGUAGGCUUCGCUGGGCGUGGUGGCUCAUGC*

*>ENSG00000252346:228627694:228627894:2:-1*

*GGAUAUGCAUAUUUUUGUGUGAAUAUCAUUUAUUUACAUAACAUGUGCUAUAUCUUAUAUAAGACACUAUAUAGUCUAUGGCCAUAGUACUCUGAACAUGCCCGAUUUCAUCUGUUUGAGGAAGCUAAGCAAGGUCGGGCCUGGUUAGUACUCAGACAGAAGGUACUGUAUACCCACAUGUACGUGCCAGGAGAAUGAUC*

*>ENSG00000222546:3557945:3558145:8:1*

*UUAUAAUAACUUAAAAAACACAAAAACAGAGCAAGUAACAGAAGAAGAUAGAAGGUGUGGAUGCUCAAGUCAACAGCCAUACCACCCUGAAUGCACCUGAUCUCGGAACCUAAGCAGGGUCGGGGCUGGUUAGUACUUGGAUGGGAGAAGUUGUGGAUGCUCAAGAUACAUGAUAUUUAGUUAGGGUGAUCAGUGACAGC*

*>ENSG00000201876:74195186:74195386:2:-1*

*CCUAGUUUUCUAGCUGGGUGGCUGGAAGGAUGAUGGGGUCACUGCUGAUAUAGAGAAUACAGGAUCUACUGUCAUGCCACCCUGAAUGCACCUGAUCUCAUCUGAUCUUGGAAGUUAAGCAGGGUCGGGCCUGGUUAGUAUUGGAUAAGAGAAUAAUACAGGGUCUUCUCCCAGAAACCUUUCUUGCCUUGGGGAAUACU*

*>ENSG00000200411:96207697:96207897:11:-1*

*GAGGUCACUCUCAUGGCCAUCUUGAUUUUGAUGGGUUUUUCUAUGGCUGUACCACCCUGAAUACACCUGAUCCCAUCUGAUCCUGGAAGCUAAGCAGGGUCAGGCCUGAUUAGUACUUGGAUGAGAGAUUUUGGUGGGUGUUAGCUGGCUUCUUCUAACCUGUUUUAUCAGCAACUUGUUUUAUGACCUGUAUUUUGUGC*

*>ENSG00000252086:219935073:219935273:1:1*

*CUGUUCUCUGGCCUCAAAAAGUGUGCAGCAUAGUUGGGGGAAUAAGAUUCAUGUUCAGUCUACAGCCAUACUACCCUGAAUGCACCCAAUCUCAUCUGAUCUCAGAAACUAACAGGGUCAGGCCUGGUUAGUGUUCUGAUGGCAAGGGUCAUGUUCAUUCAUGGAUACUUAUUCAAAACAACAAUAAUGAUAUCAAAGUA*

*>ENSG00000223113:140070173:140070373:7:1*

*UUCUGAUGAUAAUCUUUUUGUUUGGCAGAAUCCAAUAUCCAAUUAAUUCGAAAUUAUGCCUACCAUCUAGGGCCAUACCACCCUGAAAGCACGCGAUCUCAUCUGAUGUGGAAAGUUAAGUGGGGUCAGGCCUGGUUAGUACUUGGAUGGGAGAAAUUAUGUCUAACUCCAUCUAAGGAAACAUUUUACAAUACUCUUCA*

*>ENSG00000199837:182913441:182913641:1:-1*

*GAAGACCAUCAAGGAAUUCUAUUUUUCUUUCUUUUUUUUUUUUUUGAGACUGAGUAUCAUCUACGGCCAUACCACUCUGAACACGCCCCAUCUUGUCUGAUCUCGGAAGCUAAGCAGGGUCGGGCCUGGUUAGUGCUUGGAUGGGAGAUGGAGUCUCGCUCUGUCACUCAGGCUGGAGUGCAGUAGCAUGAUCUCGGCUC*

*>ENSG00000252833:75387440:75387640:5:-1*

*ACUGCAAAUAAUGAUGCAAUAAACAUAUAAAAUUCUGAUGAGUAUUCUGAUACCUCUGAGCAUGAUUAGAAGGGGAGGGACUAAGAAGUCUCUUGGGAUUUUAUCUCAACUUGGAAGCUGAGCAGGGUCAGGCCUGAUUAGUACUUGAGUGGAAGACCACCUGGGAACAUGAGCUGUAGGCUUAAAAAAAAAAAAAAGUC*

*>ENSG00000200037:72552171:72552371:13:1*

*UAAUUCUGUGUACAGUAUAAAAAAUGGCUUUAAAAUGAUGAUGAAUAAUGUCAGGGUUCUGAUCAUACCACCCUGAACAUGCCUGAUCUAAUCUGAGCUCAGGAGCUAAGCAGGGGCAGGCCUGAGUAGGACGUGGAUGGGAGAUUGCCUUGGAAUCUGGAGUGCUGCAAGCUUUAAAAAAAUAAUAAUAGAAGAAAUGU*

*>ENSG00000222500:15261319:15261519:20:-1*

*CAAUGCAAAACUAUGAGUUAAUUAAACCUCUUUCCUUUAUCAAUUACCCAGUCUUAGGUAUUGCUUUAUAGCAGCAUGAGAACAGACUAAUACAGAGGCCAUGCCACCCUAAAUGUGCCCAGUCUCAUCUAAUCUUGGAAGCUAAGCAAGGUCUGCCCUGAUUAGUACUUGGAUGGGAGAUAUUUAGGAAAAAUCUGAAU*

*>ENSG00000200326:27525406:27525606:15:-1*

*CUAACUCAGUGCUGAGAGGGAACAGUGUAGCACAAAAUGUUCACACUAAAAAACAAGAUUCUAUGGCCAUACCACCCUGAACAUUUCCAAUCUAGUCUGAUCUCAGAAGCUAAGCAGGGCCUGGUCUGGUCAGCACUUGAAUAGGAGACUGCCUGGAAACACCAGGUGCUAUAGGCUUAAAUUUUAAAAUUGUUUUUAAA*

*>ENSG00000201861:327891:328091:10:-1*

*CAGAGGCCGAGUCAAUGUCUCCAUCUUUCUUUGUCUGAUUUAAAUUAGAAAAUACUUAUCUCUGUCUACGGCCAUACCACCCUGAACGCGCCCAAUCUCGUCUGAUCUCGGAAGCUAAGCAGGGUCGGGCCUGGUUAGUACUUGGAUGGGAGAAAAUACUUAACUCUGAUGUUAUUUUAUAUGUCAAUCAUGCCUUUCAA*

*>ENSG00000202164:209785196:209785396:2:-1*

*UUUUGCUCUUUUUAGAAUUGUGCCAAGAGCUAAUUUUUUUUUUUUACUUCUGUACAUCAAAAGCUCACAGUUGAGUCUAUGGUCAUAUCACCCUGAACAUGUCUGAUCUCAUCUAUCUCAAAACCUAAGUAGAGUUGGGCCUAGUUAGUACUUGGAUAAGAGACUGCCUGGGAAUACUAAGUGCUGUAGGCUUUUAAAAA*

*>ENSG00000251945:34980817:34981017:16:1*

*GCACCCUGACUCUCUGGCCCCUCCCUUUCCUGCCCACCUGCACCAACACCACGGCCUGGGACCUCCCAGUGGGGGUUGGUCCUGGCAGCCCCCGGGCGCCAUAGCAGCCUGAGGGUGCUCCAGCCCGGAAGCUAAGCAGGGUUGAUCCUGGCUGGGACUUGGAUGAGGGACCACUUGGGGAGAUCGGGGCUGGAGGCUUU*

*>ENSG00000199564:41151987:41152187:X:1*

*AAAAUAGAAAAGAGAGGAAGCUGCCCAAUUCAUUUUAUGGAACUAUUAGAAUCCUGAUAUUUGGUCUACGGCCAUACCACCCUGAAUGCACCCAAACCCAUCUGAUCUCGGAAGCUAAGCAGGGUCGGGCCUGGUUAGUACUUGGAUGGGAGAAUCCUGAUAUUAAAACAAGAAAGUAGAGUAUCAAGAUAAGAAAGAAA*

*>ENSG00000239152:46351714:46351914:10:1*

*CAGGCACAGAUGCCUGGGGCACCAAUCUGAAUCUGCCAACAUCCAAGGCCACAUGAACACAAAUGCACAGUCUCCUCUGCGAUCGGUAGCUAAGGAUGUUAGGCCAUGAUUGGACUAGGAUGGGAGAUGAACUGAGAACUCUGGAUUCUGUAGGUUGUUGUUGUCUCUUCCCACCCCCAAGCAACAAGUGAUGAACACAC*

*>ENSG00000201713:25066440:25066640:3:1*

*UCUCAAAAAAAAAAAAAAAAAAAAAAGGAAAUUUAAAAAGAAAAAUUGGUUAGCAAAUAUGGCCAUACCACCCUGAACACUCUCAAUCUCAUCUGAUCUCAGAAGUUAAGCAGGAUUGGGCCUGGUUAGUACUUGGAUGGGAGAAAAAUUGGUUAGAGAAAUCCAUGUCUGCCAAGAAAAGUUGUAAGCAUUCUUAUGAA*

*>ENSG00000199450:117034836:117035036:8:-1*

*UUUAUGGGGUACAUAGUGAUGUUUUGAUAUAUAUAAUGUAUUGUGAUCAGAUAAGGGUAAUCAGUCUACGGCCGUACCACCCUGAACGCACCCAAUCUCGUCUGAUCUUGGAAGCUAAGCAGGGUCAGGACUGGUUAAUACUUGGAUGGGAUAUAAGGAUAAUUAGCAUAUUCAUCAUCUCAAACAUUUAUUAUUUAUUU*

*>ENSG00000199354:91523295:91523495:8:1*

*UGGAGGGAGGAAGAUGAGAAGAUGCAGUCACUCUAGGGCAGAAUCUCAGAAGAUAAAGAAGUCUCGAACACCCUGAACAGGCCUGAUCUUGUCUGAAUGUGGAAGCUAAGCAGGGUCAUGCCUGGUUAGUACUUUGAUGGGAGACCACCUGGGAAUACCGGGUGCUAUAGGCUUGGCCAGGCAUGGUGGCUCGUGCCUGU*

*>ENSG00000202474:66458080:66458280:9:-1*

*CGCCUGGAGUCAUGCGGGCUUGGCUGGGCCGGGCUCUUGGGGCAGCCAGGCGCCGCUGCUGGCGUCUAGGCCACACCACCCUGAACGCGCCCGGCUCGCCUCUGAUCUGUUGAAGCUAAGCAGGGUCGGGACUGGUUAGUACUUGGAUGGGAUUCCGCCUGGUAAUAGCGGUACCGUAGGCUUUUGGCUUCCCGCUCCCU*

*>ENSG00000252161:55217884:55218084:10:1*

*AAAUAGCUGGGAGUGAGAGUAAUAGAUUGCAUGGUCAGUAUUUUAAUAAGAAACUCCUCAACAGCCCACAGGCAUGCUAUCCUGAAUGCAUGUAAUCUCACGGAUCUUGGAAGCUAAGCAGGGCAGGGCCUGGUUAGUACUUGAAUGGGAGGAACUAACCAGCCAAUUUCUAAAAUGGUGUUCACAUCCUGCACUAUCAU*

*>ENSG00000199299:75894877:75895077:16:-1*

*UCCUAGAGAAAUACUUAAGCCAGGAUUCCUAAUUCAUAUUAAUCCUUCCCCAAAAAUUCGUUGCUGUUGUCUACAGCCAUACCACCCUGAACGCACCUGAUUUCAGAAGCUAAGCAGGGGUCAAGGCUGAUUAGUCCUGGGAUGGGAGACCACCUUGGAAUACCAGAUGCCCUAGGCUUUUAAAAUAUAUAUAUAUUUGC*

*>ENSG00000200227:150856961:150857161:5:-1*

*UGCCUCACGAGUUAAAAUCAACUGAAAAAGAAUAUAAAAAAAUAAAAAGCGAAUCCAGGUCUAUAGCCAUACCAUUCUGAAUGUGCUCAAUUUCACCUGAUCUUGGAAGCUAAGCAGGGUCAAGCCUGGUUCGUACUUGAACGGGAAAGCCCCUGGAAAUACUGAGUGCUGUUGUCUUAAAAAAUACAAUAAAAUAAAAC*

*>ENSG00000199402:29163232:29163432:10:-1*

*GCAAUGCUUGACAGAGAAAAACUGGGAUUUGGACAAGAUCUAUUCAAUGUCCCUGCACAUUGUGUAGAAGCUAGAAUUGAUGGUGCAAAUAUUACAUCUUGAAGAUAAUGCACCUGAUCUCAUCCAAACUCAGAAGCUAAGCAGGUUUGGGCCUGGUUAGUAUUUGGAUGGGAGACCACCUGGAAAUACUGGGUGCUGUA*

*>ENSG00000212433:10544023:10544223:8:1*

*CCCUGUCGUUCUCAUAAGCACCCACAUGCUUUUCAGUACAAGAAUCUAGACUUGGGUCUACAGCCACACCACCUUGAACAUGCCUAUCUUGUCUAGUCUUAGAAGCUAAGCAGAGUCGGACAUGGUUAGUACUUGGAUGGGAGACCGCCCUGGAAUACCUGGUGGUAUUAAAAAAUUAAUUAAUUCAAUAAAGUAAUAGU*

*>ENSG00000201447:76287092:76287292:X:1*

*AAAAAAAAAAUACAAAAUAAAAAAUAAAGUAACUAUGGUCUAUGGCCGUACUACCCUGGAUGUGCCCAAUCUUGUCUGAUAUCUGAAACCAAGCAGGGUCAGGUCUGUUUAGUACUUGGAUGAGAGACCUCCUGGGAAUACUGGGUGUUGUAGGCUUUUUAAAUAAAUAAAUAAGAAGUGACUAUGAGGGAUGUUAAUAA*

*>ENSG00000239049:88606808:88607008:2:1*

*CCCAGGACUCUGUCUCUUAGUUCAUGUUUUCCCAAUACACCAUGGUGCAUCUUUUAAACAAUGUGUAUAGAUCCUUUUGCAGCUCAUAAGUGUGAUGAUUGGGUUUUCACACUCAUGUGUAAGAUGUGUUUCCCUCAAACCUUGCUAUGAUAUCAGCACAGUACCUAUCUGACAUGAAAAACAUAAAAUAUUUUUAAAUG*

*>ENSG00000199392:75143447:75143647:18:-1*

*UAUUUAAUUAGAAAAUUAAAAGAUCUAAACUCAAUCACAGAGUUUCCACUUUAAGAAACUAGAAAAAAUAAAGGACAUUUCAAUGCGGGCUCAUGGGGCUGUGAAGCCAAGAGCUAUUAACACUAUGACCAAGGAUUGAAAUUCUCUAUAGGAUCCGUAGCACUGAAUAGUGCUAUAUUUUCCGGAGGAAGUAUAAGACU*

*>ENSG00000201384:77667291:77667491:14:-1*

*UACUGGGAGCACAGAGGAGGAGCUCCUGCCUCAGUCCUGGAGAAUCCACAAAGGCUGCCUGGAGGAGGUGCCAGCCAAGGCAACUCUUAGAAGAUGGAUAGCUUCUCUUUUGCUGUGGGUGUGGCCACAAAACGUUUCCCACUUGGUUUUCUAUCUGCUACCUUGCUCUUAGCCAGCCUAUUGGGAACAUUUUAUGAACA*

*>ENSG00000238598:78291728:78291928:9:1*

*UGCACCAAUCAAAAGAGAAUGCCAGAGUGGAUCAAAAAACAAGAGCCAAGUGUAUGUUGCCCACGAGACACACAGAAUAAAAGUAAAAGAUUUAAUCCUUUUGGGGUUCAUAAGCGUGAUGACUGGGUUUUUGACACUCAUGUGUGAGAUGCACUUCUCUCAAACCUUUUUGCAAUGUUGGCAUACUAUCAUACCUGCUU*

*>ENSG00000252669:153970450:153970650:1:-1*

*UUAAUAUAGGCAGCAAACCACCAUAAUAUCAGCAGUAUUGUGUGCUUUUGUCACCAACAGAAUUCAUUGACUAUACUUUCAGGGAUCAUUUCUAUAGUUCAUUACUAGAGAAGUUUCUCAGAAUGUAUAGAGAAAAAAAAGAAUCCACAGAUAUAUUCAUGUUCCUACUGCCAGAUUUAAAAAAUAUUUCUCUUAAAAAU*

*>ENSG00000252774:64634671:64634871:15:1*

*CCUCAACCAGCCCACUUCAUUCAUAUCUUUUAGUAAUCCAAAGUAUUGUGCAGAUCACCAUCAGAUUUACCUUGAAUACAUACAGAUACUUCCUGUUACUCUCUAGCUUAAGAACCUUUAAUGUGGUGAUGUCCAUCUCAUGUCAUCCAGGGAUAAAGCAACCCUUGUUUAUCCCAUCUUGGCUCUUGGUCUGUGCCCAU*

*>ENSG00000221475:51933643:51933843:X:1*

*UCUUCCUCUCCAGUCGUUUCCUCUGGUGGGCCUCUCCAGGGCUCCGCCGGGGUGUGGCCAAGACCCUCGAGGUGGGGUGUGCUCAGAGCAGGGGGCCUGAAGAAUGGCUCCUCUGUUUACAACACACCCAACAGGAAGCUGGGGUCAUCGUGAUGAGGGGCACAAACUUGUGGCCUCCCUACAGACAAAUGCCCUACAUG*

*>ENSG00000212533:31229716:31229916:12:-1*

*AGUAGAUCGAGAGCCCAUCUUUAUAACUUUGGCCUCAAUAAGACUAGUGAUAGUGUCACUCUAUUUACGAGGGGAAAGGUAGUUGCCACGAUGCUCUUCGGUUUAGGGGCAAUUAAAAUGACUACAGAUUGGUGGCAGUUUAUGGAUUUGCACAAGAAGAGAGAACUCACAGAACUAGCAUUAUUUUACCCUCUGUCUUC*

*>ENSG00000201487:76255093:76255293:1:1*

*GAGUUGGAAAUUGAAACUGUAUCAGGAUUUGGUCAAAAAGUUUUGUUACAAGUGAAGCUGUCCUACAAGGUCAAUGAUGUAAUGGCAUGUAUUAGCUGAAUCUAAAGUUGAUGUGAGUUCUAAAAUUACACUGAGACCUUGGAGGGUAAAAUUUUUAUCUUCAGUAUGUGUAAAUACUGUAAAAUAGCUAAAUGGAGCUA*

*>ENSG00000238717:56152936:56153136:5:1*

*UCUUAAUUUCAGAAAUCUUCUAGGAAAGAAAACAUUUAAUUGCAGUAAUUCAUAAGUGUAAUGAUUGGGUUUUCACAUGCAUGCAUAAGAUGUGCGUUUCUCCAACUUUGUUACAGUGUCAGCAUGUUAUCCUCAUUUGACAUGAAAAAAUGUUUAAUAGACCUAUGGGGAUUAAGAUAAAUUAAAAAUGUGUGGGGUUU*

*>ENSG00000252787:52722836:52723036:3:1*

*AUAUUUGCAUUUUUGAAAUCCAUCUUGAUCAGAGACUGCUGAGCAAGCCUAUGUUUUACUUUCCUGUGUGAGAAAUGAUGAGGGUCAACAUUCUUCAUACCAAAGUGAAGACAUGAGAUCCAACUCUGAGCUCACCCUGUUGCUAAAUGGAUAAUGCCAGUACUCUCUUGUGGAAGGUAUUACCAGAACAAGGGAUGUAG*

*>ENSG00000238414:27157008:27157208:10:1*

*CCCCACUGCCUGAAAUAUCUAUAGUAAUUUUUGUUUCCUGACUGAACUCUGAUACAACUAAACACAAAACACAUUUAAAAUUCUGAUCCUUUCAUAGUUCAUAAGCAUGAUUGGGUUUUCACACUAUGUGUAAGAUGUGCCUCCAUCAAACCUUGUUAGGAUGUUGGCAUAUCACCCAUCUGAUGUGGGGGGAAAAUAAA*

*>ENSG00000200733:90489341:90489541:13:1*

*GGACAAAAGCAAUUUGUGAGUGGAUAAACAUCAGAAUUUAAUAUUCAAAAUUCAAGACCAAAUACUGGAAUUAAUAAAUUCAUGUCAGUGCUGAAACUUUGUCAAGUUAUGCUACUGACAUGAAGUAAUGAUACAGUAUAUCUGAGGAGAAAAAAAUAAAUAAAUUCACCUAUUGGGAAAGUGUUGUUGGAGGAGAGAAA*

*>ENSG00000206688:25330462:25330662:15:1*

*UAGGAUGGUAAGUGUCCAUUUCUCAGAAGCUCCAGAUAUUCCUCCUCUGCAGGGACAAAGACUGUGCCUGGAUCGAUGAUGACUUCCUUAUAUACAUUCCUUGGAAAGCUGAACAAAAUGAGUGAAAACUCUAUACCGUCAUCCUCGUCGAACUGAGGUCCAGCACAUUGCUCUUACAGGGGCUAGAGAGAGAGGGACAA*

*>ENSG00000252672:116073218:116073418:7:1*

*UGCCAUCUUACCAUACAUUUCUUGUCUCUGCAAUGAGGCUGAGCGAGACUGUCCCAAAUGUGUUUUUAUCCUGAGUCUGAGGCAGGCUGAACUUUAAUCUGCAUGAUGUGCUGCAGCUUGCCAUCAUGUCGGGACAGCAGAUCCAGUCCCUUGAGUAAGCCAGUUGGUCACAGUUCAGGAGAGGCUGAGAUGAUCUGUGA*

*>ENSG00000200729:173834403:173834603:1:-1*

*CUUGGGUAAGGACAUGAAGACAGUUCCUGUCAUACCUUUUAAAGGUACAUGUUUUAUUGAUGUUAACGUUAAUUGAUUGAGCUACUGUUAGUGAUGAUUUUAAAAUUAAAGCAGAUGGGAAUCUCUCUGAGAAAGAAAAUGGAGAUUAAUCUUAAACUGAAACAGUAGUUGGGAAAUCUUUUAGAAAUCCACCUAUUACU*

*>ENSG00000202048:101447278:101447478:14:1*

*GUGGAUUAAUAAAUCAUUAAUAAGCAAUUAGAAGUGAGUGGAGAGAAAGAGCACCUGUCCUGUGGAUCGAUGAUGACUACUGGUGGCGUAUGAGUCAUCUACAGUGAAUACGUCUCUGGAACUCUGAGGUCUGUCACAAAAGAAAUCUAGCCCAUUUUCUAUGUUUGAUACAAUCCUUUAGAUGGGUAUCAUGAACAUAG*

*>ENSG00000252256:86096096:86096296:9:1*

*AAGGUCCCAGCUUUUAUAUUAGACAGCAGGUUUCUAGAUGUUUAUUACAUUAUUAUAAACAAAUAAGUAAAAGGGAGGCCUUGCAUAUUCCAAUGAUGCAAGUGUGUCGUGAACUAAGGAUUAUGAUUAAUCCAGUUUUGUAGCUAGAGGGAUUUUAGGGAAAAAUAAAUAAAGAAGGAACAUGACUUCUCCAGGUAGAU*

*>ENSG00000238760:97646948:97647148:2:-1*

*UUGAUAAAUUGGGCUACAUUAAAGUUAAGGACUUUUCUUUAUCGAAAUAUACCAUUAAUAGAGUGAAAUGAUCCUUUGUGGUUCAUAAGCAUGAUGAUUGAGUGUUCACGUGCAUGUGUGAGAUGUGCCACCCUGAGACCUCGUUACGAAGUCAACGUAUUACCCAUUUGAGAUUACCAAAAAAGAGUGAGUUGGGCGCG*

*>ENSG00000252531:3628068:3628268:2:1*

*UAGACUCUUUCUGUGGUCUUUUAGUUAGGCUGUAUAUUCUUGGUGAGUUAGGGGUGGGUGGUGAUGGGAUCAGUGUCUUGGGGGGACAUAACCAUGUGGGUGACUGCUGGGGUCCCCUGAUGGCUUCCCCGGUGCAGUGGUGUACAGUUCUGUCCCACAGCAUUGGAGAAGAGCUUGUCCCCGGUCGUGAAGACUGCUGC*

*>ENSG00000238920:40144305:40144505:X:1*

*CUACAAGACAUUGUAAAUUUCUGGGUAGCAGUGAGUGGUGUAGAUCCUUUUAUGGUUCAUAAGUAUGUUGAUUAUGUUUUCAUGCUAUUGUGUGAGAUGUACCUCCCUCAAACCUUGUUAUGAUGUCAGCACAUUAUCCAUCUGAUGUGAAAAAAAAAAAUGGUGAGUGGAGAGUGAAAUGCUGGGAGCUGAGAAGGUAG*

*>ENSG00000212579:35619520:35619720:6:1*

*CCACACCUAAUACUUGUUUUCUAAUGAUUCAAGGUACACAAAUUUUAUUUAAUGCACAAAAUUAUUAAAAAUACUGUAUACUACACUGUUUUUGUUUAACUUGUGGACAAAGACUUAUGGUUAGGUGCAAAAAAUAAAUCCUCUUUUGCAACCCAGAACUCAUUGUUCAGUAUGAGUUUUGAUACAUAUAAGAAGGUGUU*

*>ENSG00000238929:37238177:37238377:3:1*

*GGGUUUACUUGUAUAAUAAACAAUGUUUGAGAGAUUUUUAAAAAAACAGAGAUGAUCCUUUUGUAGUUCAUGAGCAUGAUGAUUGGGUGUUCCUGCCCACGUGUGAGAUGAGCCACCCUCGAACCUUGUUACCAUGUUGACAUAUUACCUGACUGACGUGAGAAAAACAAAAAAAAAGGAGAUUAUGUUAAGUGCCUGGG*

*>ENSG00000252525:70660276:70660476:X:-1*

*UGCAGAGAAACUGGAUCACAUACAUUGCUGGUGGGAAUGUAAAAUGGUACAGACACUCCAAAAGUGUGGCACAAUCUUUUUGUAGUUCAUAAGCUUAUGAUGACGUAAGUGUGACGACAUUGGGUUUUCACGUUCAUGUGUGAGAUGUGCCUCCCUCAAGCCUUAUUACAAUGCCAGUACAUUUUUUUUCCACAUCUGAU*

*>ENSG00000200486:25434475:25434675:15:1*

*GCCCUGGUCUCCUGCACGGAGCUGCGGUGAGCACAUCCGGGUCCCGAUGGAUGCAUGCGUGGGGAUGGAGGGCGUGCCCUGAGUUGGGUCAAUGAUGAGAACCUUAUAUUGUCCUGAAGAGAGGUGAUGACUUAAAAAUCAUGCUCAAUAGGAUUACGCUGAGGCCCAGCUUAGGUGAGAAUUCUGGAAGAGGAUGCUGA*

*>ENSG00000199663:19015667:19015867:17:-1*

*UUUCUCGCCAUCAGUUAAAAGUUUGCGGCAGAUGUAGACCUAGCAGAGGUGUGCGAGGAGGCCGUUAAGGCUAUACUUUCAGGGAUCAUUUCUAUAGUGUGUUACUAGAGAAGUUUCUUUGAACGUGUAGAGCACCGAAAACCCCGAGGAAGAGAGGUAGCGUUUUCUCCUGAGCGUGAAGCCGGCUUUCUGGCGUUGCU*

*>ENSG00000202389:26349303:26349503:17:-1*

*AACAGGCAAAUCAAUGAAAUAGAGUAGAAAUCCCAUAACAAAUAAAAGUUUGUAGAAGCCAAUUAAGCCAACUGAGUUUCUUUCCUCACAGGGACCUGGUGUGCCAUGGCUGCAAACAGCAGCUUCCUUGUCAGGGUACGCAGACUGUUUCUUGGAUGGGUUGCUCUAAGGGACCUUGGAGACAGGCCUUUCAAAUGUAU*

*>ENSG00000238628:88430546:88430746:6:1*

*AUACACACACAUAUAUAUAUUUGCGUGUGUGUGUGUGUGUGUGUGUGUGUGUGUGUGUAAUAUGUAUCUGAAAGAAUACAUAUUAGAUCCUUUUGUAGUUCAUGAGCAAGAUGAUUGGGUGAUCAUGCACAUGUGUGAGAUGGGCCAUCCUCAAACCUUGUUACAACAUUGGCAUAUUACCCAUCUGAUAUGAAAAAACA*

*>ENSG00000200832:101420622:101420822:14:1*

*UUAUAUUUACAUGGGUUAUAAUAUUAAUAUUCAUGAAUUAAUGCAUAAAUAACAUGUAAAUGAGAAUUAAGCUAAACAGUCCUAAGCCUGAGCCAGUGAUGAAAACUGGUGGCAUAGAAGUCAAGGAUGCUGAAUAAUGUGUGUCUAGAACUCUGAGGUUCAAUAGAGGAGGAAAUAUAGUCUAUUGCUUUUGAAGUGAU*

*>ENSG00000201810:180260422:180260622:3:1*

*AAAGAAAAUCAAGACAAAGAGCAGGAAUAAAUGUAACAAAGAUACCAUAACGAUAUCUUGGAGUUAUGGUCAGGUGGAAUAAUCCUUAUCUGUUUCUCCUUUUGAAGGGCAGAUUAGAACAUGAUAAUUGGAGUUCACAUGAUACAUGAUUAAAGUCUCUGUGUAAUCAUGACUUACAAUAUCCUGAUUAUUCCUAUCUU*

*>ENSG00000212295:156699817:156700017:6:1*

*AGAGUUGACAACCCUGACAAGGGCUUCUGUAUCAAGAAGUCACAGAGUCAAUUAAAGAGGAAUCUCUUCAGAUGAUUUGAAUUGAUAAGCUGAGGUUCUGUGAGGAAUGACAGUUAAGAGCAUGUUAAAGUUGUUAUGAAAAAAGAGAAGAAUCUCUGAGUCUUCGUUCGAUGCCAGAUGCAGAACUGAAGACCUUCUGA*

*>ENSG00000199571:40213460:40213660:12:-1*

*AUUUAUACCUGCAAAGAUAAAGAGUUUUUUCUGUGGUCAUUUUAUAGGAUUGAAAUUUGACAUUGUGCUACAUGCUAUUAAAAAUGUAUUAUUGGGCAGUGAACACCCAAGUGUGCUUUAUAGUUCCUUUGGCUUUGACUUUGUGCUAGAGCAAUGUCUGUUAUUUUUCUCUGCAUUGAAAGGAGCAUUUAUCCUUUUAA*

*>ENSG00000252923:197550780:197550980:2:-1*

*AAUGAACUAAAAUAGCUAAAACACUUUAGGAAAAUAACACCGUUGGAGGACUCUACCUAAUUUCACGAUUUAUUAUAAAGCUACAAUAAUCAUAGUCUGUGGAAAGGCUACUGUGUUGUUGAACUUGCCCAGCUGUGGGUGCAGCCAUGUACCAGGCAGCCCAAGCCUGACAUAAAGUAAAUUCCCAGGAGAAGUCUUUC*

*>ENSG00000238810:25227057:25227257:15:1*

*UGCUUUUAGGACAGAAACGUAAACUGCUCAGGUAAAAUAACCUUAUAAAGAGAAAUAAGCAUAUAUUGCAGAGGCCUUGGCUAGGUUCAUGAUGACACAGGACCUUGUCUGAACAUAAUGAUUUCAAAAUUUGAGCUUAAAAAUGACACUCUGAAAUCCAGUCAGUGUGCCUCACUAGACUUUUCGAUUUCAAGAUUUUC*

*>ENSG00000202231:100077536:100077736:X:-1*

*UGUCGCCCAGGCUGGAGCAACUGAAGAAUUUCUAAUGUCAACUUUAUCUGUUUCUUAAAGAUAUACACUUACUAGCAAGCCUCCAGCAUGCUUAGGUCUGCAGUGACCCUAUGCAUCCCUACAGUGCUUGCCAGAACAGUUUUGAAACGGUUUGAGGCCUUGCCCUGCUCCAUCCAGAGCAAGGGUAUAGAAAUUUCAGA*

*>ENSG00000238792:96995709:96995909:9:1*

*AGAAAAUUGGACAGAAAAAUCAUUUGAAAAGUGGUUAAGGAUUUUCCCUGAUCCUUUUGUAUCUCAUAAACAUGAUGAUUGGGUUUUCACACACACAUGUAAGAUGUGUCACCAUCAAUCCUUGUUAUGACAUCAGCACAUUACCCAUCUAACAUGAUUGAAAAAAAAAAAAAAAGAAUUUUCCCCAAAUGGUGAAAGAC*

*>ENSG00000201689:101456363:101456563:14:1*

*UAGUAAAUUAAUGAAUUAAUCAACAGACAAUGUAGAAAAUAACAGAGAAAAGAAGGGCCAAUCCUGGAUCGAUGAUGACUACUGGUAGCAUGAGUCAUAUACAGUGAAUACAUGUCUGGAACUCUGAGGUCUGUCUCAAAAGAUAUCUAGUCUAUUUCCUGUUUCAAAUGAUCCUACAGACAGAUGGCAAAAAAUAGAAU*

*>ENSG00000206602:47017568:47017768:18:-1*

*GGUUCCAAUCUUCGUGUUCACUUUAAGGUAUGCGAUUCAUAGUUGUGAUCCAACAGUUCCUCAUGUUCCACUCAAAAAAGGUAGCUGCAGUGAUGACUUUCUUAGGACACCUUUGGAUUUACCGUGAAAAUUAAUAAAUUCUGAGCAGCCACCUUAUAUUUAGGCAUUGAUGAUCAGGGUGUAAGGAUGUAGCGUUUGUG*

*>ENSG00000212165:16963410:16963610:20:-1*

*CUGUGGGGCUUACUGUGUGGACUGUUUCCUCGGGUUGGGGACCCCUGCAUUAAGAAAUGUAAAAUGAAGAUUAUAUUUUCAGGGAUCAUUUCUAUAGUUUGUCACUAGGGAAGUUCCUCUGAAUGUGUAGAGCACCAGAAACAUGAGGAAGAGGCACAGGGUUCUCUCCUGAGUGUGAAGCUGGCUCUUGGCGCUGCUUU*

*>ENSG00000201465:137872657:137872857:7:-1*

*CCUUAGCUCAGUGUCAGUUUGGGCCUGGAUUGGUUGUUUCAGAAGCUGAAAUCUUGUAAGUUUCAGCCUCAAAGAUUUGUGGUGUUAGCCUCCUGGUGCUCAGCACAGGAUGUGUUCUUACCCUAACCAAAUAGAAAGAGUAGGCUGAAUUAACCUAUGCCAUAUACACCUCAACCCAGGCCCUGCAACUCCUCUGUAUU*

*>ENSG00000238741:160232656:160232856:3:-1*

*CAUAAAACCAGUUAUGAAAAGUGCAAUUUUGGCUCUCUUGGAAAUGAUGAAAUAGAGAUAAUUGGGUGUUGAAUGUUGUAUGACUGGAAAUUUUUAAUAUGUAUAUUAUGUAAUUCUUAUAAGUUUAGUGUUUUAUAGGUACUUAAUAGAGAGUGUGUAUGCAUGUGUGUGUGCUUGUGGUGGCUAUGGAAAGGGGGCAC*

*>ENSG00000251806:2443538:2443738:20:-1*

*GAGGAGCACCCCCUCCAGGUGAGGAGCCCAUAAGGAGACCAGUGCUAAUUGAUAGAAGAUGCCUUAGCUGGAUUAAUGAUGAGAUAUAACCUUGACUGAAGCUGAUGAUGAGUUUGUAUAAUUAAGCAGGAUUACUCUGAGAUCCAGCGUGGCUGACUGAUGAGACUAGGUGGACAAGAGCCAUUGGGAGCCCUGGGUGU*

*>ENSG00000212195:56708928:56709128:17:-1*

*GCUCACGAAAUACUCACCAUCAGCAUAAUAUGUGUGAGUUUCUUUCGCAUGUGGAAGGCACAUAAAUGGACAUUAAGACUAUAUUUUGAGGGAUAAUUUCUAUAGUGUGUUUCUCGAGGAGUAUAUCUGAACGUGUAGAACGCUGGAAACCGCGAGAGGGAGAUAUUUGUGUUCUCUCACGAGUGGGAUAGCAGUGUUUG*

*>ENSG00000207133:25312867:25313067:15:1*

*UUAAGGUCAUCAGCUCUCCCACUGCCCAGCCUGUGGUGUCAAAUGUUCUGCCUCUUCGAACGUGCUUGGAUCGAUGAUGAGUCCCCCAUAAAAACAUUCCUUGGAAAAGCUGAACAAAAUGAGUGAGAACUCAUACCGUCGUUCUCAUCAGAACUGAGGUCCAGCACGUUGCCUCCUCUGGGGACUGUAGGAGCGAGGGA*

*>ENSG00000201750:18967167:18967367:17:-1*

*UUUCUCGCCAUCAGUUAAAAGUUUGCGGCAGAUGUAGACCUAGCAGAGGUGUGCGAGGAGGCCAUUAAGACUAUACUUUCAGGGAUCAUUUCUAUAGUGUGUUACUAGAGAAGUUUCUCUGAACGUGUAGAGCACCGAAAACCCCGAGGAAGAGAGGUAGCGUUUUCUCCUGAGCGUGAAGCCGGCUUUCUGGCGUUGCU*

*>ENSG00000199961:74557133:74557333:17:1*

*AAAAUAGUUUGCGUGUGCUGUGGCUGUUAGUGCUAUGGUCCAGCAGGUUGGUGGGAGCUGAGUCCAUGAUGAUUUCAAGUUAUCCCUGUCUGAAGGCAAAGAAAGGCCUUUCUGUGUGGAAUUUGAAUAUCUGAAACUCAGUUUCCCAAGUUUGCCUUACAGAAAUUUUAAGUUACUUGAGAGUUACUCUGUGUGUAUCU*

*>ENSG00000253014:61073762:61073962:14:1*

*AAAAUAGAAAAUGAAAAUAGAGAAGACUAUACUUUCAGGGAUCAUUUCUAUAGCUCAUUACUUGAAAACAGAAAAAAUGUGUAAAAUAAGGAUAAGAAGAAAGAAAAUAUUUUUGUACAUCUGUAUAAUGUGUUUGUGUUUUAGGCUAAGUGUUAUGAAACAGUCAAAAUGUUUUUAAAACUUAAAAAUUUAUAAUAUUU*

*>ENSG00000212163:2233492:2233692:17:-1*

*UUCUGUUUAUGUGUGACUAAAAUUUUGCUCAUUGGGAUGCAAAAAGUCUGCCUUUAUGAUUAGGAAUUGGAGAAUAAUAGAGAAGUCAAUGAUGGUUUUAUUCAUAUCGUCUGAACCUGUCUGAAGCAUCUCAGUGAUGCAAUCUCUGUGUGGUUCUGAGACUUCUCCAAGUAUGUUUUUCAGACCAGAUUUUGAAUAAG*

*>ENSG00000238981:92378551:92378751:15:1*

*GACUUCUUUACAUUAAUCUUGACUUUUAAAAAACAGUGCCUUAAAAUAUUAAUCUUGCUCCUUUUGCGGUUUAUAAGCAUGAUGAUUGGGUUUUCAUGUGUGAGAUGUGUCUCCCUCAAAUCUUUUUAUGAAGUAGAUGCAUUGUCUGUCUGACAUGAAAAAAGGAAAUAAUUAUUUAUCUUGAUUACUUCAUUUUUGGC*

*>ENSG00000252550:106549829:106550029:13:-1*

*GCUAGAUCAUAUGGUAGUUCUAGCUUUAGUUUUUUGAGGGGUAGGUCAUUUCAAAGAAGGUUCACGUGGCUGUGAGACCAAGAACGCUUAAUGCUGUUACCAAAGAUUGCAAUAGGUUGUGAUAGCCCUCAGUAGUGCUAUGUUCCUCUGGGGAGAUAUUAAUUAAAAAAACACACACACACAGAAAAAAGAUGCCUAAA*

*>ENSG00000238878:22101864:22102064:13:-1*

*AUUGUACUUAACAGCGGUCAUAUUUUAGAAUGGUUCCAUCUUAAUAUUUCUAGAUAAGCAUUGUAACUGACAGAAUUUAUUCAUCUUAAGUGUGAUGACUGGGUUUUCACAUUGAUGAGUGAGAUGCGCCUCCCUCAAACCUUGUUUACAAGGACAACAUAUUGGUUGUCUGACGUGAGGAGAGGAAAAAGUCAGAACUU*

*>ENSG00000212414:101016721:101016921:8:1*

*UUACUUAUAUAUGUGUAGUUUAUAAAAAAUGAAGAAAAGUAUAAAAAUAAAAAUUAAAAAUAAAAACAAAAAUAAAAAGUGAAGAUUAGAUACUAUGAUGGUUGCAUAGUUCAGCAGAUUGAAUUCAUGAAGAGAUUUACAAUGUCUGAUGUAGUUUCAAAAUAUAAAGAUAGGAUUAUGCACCUAUUUAUAGUUUUGUG*

*>ENSG00000239035:57570710:57570910:15:-1*

*GGGAGGGAGGGAGAGGGAGGGAGGGAGGGAGAGAGAGGGAGAGGGAGGAAAGGUUUGAUCCUUUUGUAGUUCAUGAGCAUGAUGAUUGGGUGUUCACGAGUGUGUGUGAGAUGUGCCACCCUCCAACCUUGUUAUGACCUCAGCACAUUACCCAUCUGACCUCAACUUGGAAAAGAAAAAAAAUUUUUUUUUUUACAGUG*

*>ENSG00000238486:52172602:52172802:19:1*

*UAAAACAAUACCACAAAUUGAAUCUUUGUUUACUGCUUGAAAAGCUACUUUCUAGAUCCUUUUGUAGUUCAUAAGUGUGAGGACUGGGUUUUCAGGCUCGUGUAUAAGAUGUGCCUCCCUCACUCCUUGUUACAACAUCGGCAUAUUACUCAUCUGAAUGGAAAAAAAAAAGCUUCUGAAUAGUUUCAGAAGUAGAUGUU*

*>ENSG00000239188:141961660:141961860:X:-1*

*UCGUUUGUUUGCAAUUUAGCUUUUAACAAAAAUGUUUCAAAAGUAAGAUAAUAAAAAGCCUAUAGAAUAAGGAUAUAAAGAAAUAGUAAUUUCGUAGUUCAUAAGCAUGAUGAUUGGGUGUUCACACACAUGCGUGAGAUACGCCACCCUCCCACCUUGUUAAGAUGUUGGCACAUAACCCGUCUUACAUGAAGAAAAAA*

*>ENSG00000252601:29070521:29070721:5:1*

*AAUUUGUCUUAAAGAUCAACAUUUAUUUUACUCUCAAAAGCAACUCUAGUUUUGUGAUACGUUAAGAUUAUACAGUUUGUGGAGGAGGUCCAUUUUAAAAAAGAAUAGAUUGGAAAUGCCUCAUAGAGUAACUCUGUGGUUUUAUUUUACCCACAGGACUAUGGUUAAAUAUGUGGGAAAGAACUACAAGACAGUUGUUA*

*>ENSG00000220986:68456560:68456760:5:1*

*AGCCGAGAUCAUGCCACUUCACUCCUGCCUGGGCGACAGAGUGAAACUCCGUGCAGGAAAAAAAAAAAAAAAAAGCGCUGCCUUUGUACCCCACCGUGCCUCCGCCUGGUGUCUGGGGUCAGGUUGCAGUGCCAGAGCACGUGCUUGCGCAUAAAUGGGGCAGCCUGGCCCCCUGCGGAUGGUCCUUUUAACCGCGAGCA*

*>ENSG00000201500:101443660:101443860:14:1*

*UUAGUAAAUUGCUGAAUUAAUCAAUAAACAAUGCAGAAACAAAUGAAGAAAAGAUGGGCCAUUUCUGGACCAAUGAUGAUGACUGGUGGUGUAUGAGUUAAAGGUGAUGAAUAGUAAGUGUCUUUGUUAGUGGCAAGUUCAGAGUCGUUUGUUUUAAGAGAAUAUGAAAUAAAUAUAUUCCAAAUAAUGAUGAUUUAAGU*

*>ENSG00000251881:80434336:80434536:15:1*

*CCAGCUUUCUUGGGGUUAGUAGAGUCAGGAUGCUUAUAUUAGGAAACUAAAAGAGGCCAUGUAUGAACCAAUGCUGAGAGUGUCUUGAACCAGGGAUUAUGAUUAAUCCAAUAUUCUGUACCGGAAGUCCAAAGUAUCAAAAGCAGAAAGUUAAUUAUAAAGCUGUUUUGAUGAUCACAUUUUAGAACUUGAAGGUUAUG*

*>ENSG00000238676:7937734:7937934:17:-1*

*UCCCCAGAAGAUUAGCCAGGGAAUCUACAUUUUACAGUAGGGUGAUUCUUCUUUAGAACCACUGCCCUUGAUCCUUUUGUACUUGGUAAGCAUGAUGAUUGGGUUUUUAUGCUUAUAUAUGAGACAUGCUUGUCUCAAAUCUUGUUACAGCACAUUACCCUUCCUACUUAAAAGGUUAAAAAAAAAAAGAACCAUCGCCC*

*>ENSG00000238298:133325174:133325374:9:1*

*GGUGUUGAUGUCACGGGCGCAUCCGCACGCCGAAACUUGCUAAAUUGGACACAAAAAAAUAUGCGAUUAUUUUUGUAGUUGAUGAAUGUGCUGAUUGGGUAUUCUCGUGUGUGUGUGAGGUGCCACCCUCAAACUUUGUUAUGAUGUUGGCACAUUACCCAUCUGAUAUUUAAAAAAUGCAAUUUAUGAUAUGUCAAGUA*

*>ENSG00000251704:16380379:16380579:2:1*

*GGGUGUGGAAUGAAUCCACCCGCUCACACCUUCCUCGUGUUCCAGGUGCUGUUCCAUGCACUGAGGAUUCCAAAGAGAAAUAAUCCACAACCAGACUUACAGAUAAGUUCGAGAAAUAAACCCUGUUUUGCAACCCAGAGCACAUUGUUCAGUAUGAGUUUCGAUACAUAUAAGAAUAAAUAUUAUGAUACCAAAAAAAA*

*>ENSG00000253059:36225467:36225667:14:1*

*GAGAUAUUAACAUAUUUUCAAUAGCAAAAAUUUGUCACUUCUUCUUCGAGAAAAUAAGCAACUAUAUGCAUCUACUAUGUGACAGACCUGGAGCAGUUCCUGUCUGCUGCUAAGGUUUCCACUACAGAUGCAAGAAAAACAUGUCCUUGUACUUUCUGUCUUUUUUAUUGUGGCAGCCAAGGAUGAAUAGAGGGAUACAG*

*>ENSG00000252154:97618102:97618302:13:-1*

*AUUAUUUAUCUUUAAUUGGGUUAAUAGAUCACAAGUGUUUAUUGUCUUAAUAAACAAGCAAAGAAAUAAAUUAGGCAAAGGAAGACUUUGCAUAGACCAGUGAUGAGAAUCUGUCAUGAACCAAGGAGUAUUAUUAAUCUAAUUCUGUUUACCUGAGAGUUUUAAAAGAUGAAUAAAUACAUAAAUGAAUGAAUAAAUAA*

*>ENSG00000202449:14692259:14692459:4:1*

*UGCUUCUGCUUGAGAACAUCAACAGCAAAUACCAUUGAGGAAAUACAGCCUUCUCUCAUAAAAAUCUCAUAUAAAGGCAGGUACUGUUAGAAUAUAACUGAAGUGUUUUAUUGUUAUUGUCAUUGCUUAAAGCAUUGGAUACAUAACUAUCUCUAACUCCAUUCCAUACUAAGACAGAGAUGUUGCAUCUCAAACAUCUU*

*>ENSG00000251820:20434012:20434212:22:-1*

*UGUGAAUAAAUUACAGAAGCUCCAUACUCUGGAACAUUUGUCAGCUCUUAAAGAAUGAAUUAUGAUCCUUUUGUAGUUCAUAAGCAUGAUGAUUAUGUGUUCACACACGUUCCCAUGUAAGAUGUGCCACCUUCAAAUAUUUUUUUUUUUUUUUUUAAGAGGGAGUCUUGCUCCGUCACCCAGGCUGGAGUGCAGUGGCG*

*>ENSG00000238840:5004548:5004748:10:-1*

*UUAGAUAUACUUUAUAAUAUUGCUAAACAUUAUUCAUAAAGAAAAACUGGUAACUAAUGACAUAUAAACUGGUGAAUAAUCCUCGCAUGGGAUAAUUUUUACUUUAUUCUCCCUUUGGAGGUCAGGUUAAAACAUGAUUGUAAUUUGCAUGAUCCAUGAUUCACAUCUUGGAGUAGUCACAACUUGCAGUGCCCUGAUUG*

*>ENSG00000252109:50303543:50303743:17:-1*

*AGAGGUUACAGUGAGCCAAGAUCUCGCCACUGCACUACAGCCUGGGCAACAGACGGAGACUCUGUCUCAAAAAAAAAAAAAAAAGAGGGGGGAGUUGAAUCACCUCUCUCUCUCUCUCUGUCACUCACUCGCUCACUUUCAUUGUAGGCCGGUUUCUGUAUUUUACAGAUUUCUCCACAAGUUGGAGAACUUGAACAUGG*

*>ENSG00000212214:3450088:3450288:X:-1*

*CACAGAUCCAAACCCUAUCGCUGACUGUCUUUGAGUGCCUUUCUGAUUUGUCCUAAGAAGCUCAUUUUAUUUCCCUGACCUGGGUAGAGUGGCAUCCAGUUGGUGGUGCCCAUCUCAUAUCAGCCAGGGACAAAGCAACCCCUUGUUCCUCCCAGCUUGGCUUUUCAUCUGUGCCUAUGCCUGGUUCAUGCCUUGGACAC*

*>ENSG00000199311:25477448:25477648:15:1*

*UGGCACCCUGGUCUCCUGCACUGAGCUGUGGUGAGCAUAUCCUGGUCCUGCUGGAUGCAUGCGUGGGGAGGGGUUGUCCUAGGUUGGGUCAAUGAUGAGAACCUUAUAAUGUUCUGAAGAGAGGUGAUGACUUAAAAAUCAUGCUCAAUAGGAUUACGCUGAGGCCCAGCCUAGGUGAUAAUUUUGGAAGAGGACACUGG*

*>ENSG00000212273:134656766:134656966:8:-1*

*AAAUCAUAUUUUAGUAAUUUUAGAGAAAAAAGACAGGAAAUGUACUUUGAUGAGUAAUAUUUAAAUAUUACUUCUGCACUUAUGUAUGUUUUUGUUUAAACUGUGGACAAAGACUUAUGGAUAAGUACAAAAAAUAAAUCCUCUUUUGCAACCCAGAACUCAUUGUUCAGUAUGAGUUUUAAUACAUAUAGCCAGGGAAA*

*>ENSG00000252143:21899111:21899311:22:1*

*AGGGCCUAGGGACCUUGGGCAAAGCAGAGAAGCUGGUGUCAGAUGAGGUGCCGCAGGUACAGUAGGAUAAGGUGUUCUCAGGUCCCCAGUGCAGCCCCAAGAUGAGCCUGCAGUAUUUUCCUUACAUGAUCUGGUCCUACUGUGGGCAGCGCUGCUGCCCAGAGCCUGAGAGGAUUAUGAAAACAUGGCAACGGAAGUGA*

*>ENSG00000212567:40790128:40790328:5:-1*

*CUGUUAACUUGUUAAUUUUAAUGGUGAAUAUCAUAGUUUAGAAAAUCCACCCUCCUUUUCUUGUCAGGGACUGAGUGUGUGGUCCUGCUCCUGUUAUGUAUGGAGGCAAAGGGAAAGGGCUCCGGCCCCCUCAAAAUCAUGUCUUUGGUGCCAGCCGGUUCUGUUCUCAGUCACCUGGAUACUGAACUUUCCUGAGCAGG*

*>ENSG00000200792:33749425:33749625:21:-1*

*GAACAUACUACAGUUUAAUGUUUCUGCUGGGAAGUUUGGCUGUACACAUCAAAACUUUUAAGGUGCUUAUGCAUGGGUUUGGAUUUAUGAUGGGCCCGUCCCCCUGGACCUCUCAUAGUACCCCAUGCCAGAGCAAACUGUAGCCCUGAACCAUUGCCUGGCCUCUGUUCCCGUAGGCUGCUGGCACUGAAGUGGGUUGC*

*>ENSG00000238969:32865957:32866157:X:-1*

*UCACCCAAAGCAAUUUAGUCAGGGUUAACAAAUUUCAUACAUAAUGUUUAAGAUAAUGAAUUUCAGAUCCUUUCGUAAUUCAUAAGCAUGAUUUGGGUGUUUAUACUCAGAUAUGAGAUAAGCCUUUCUCUAAACUUAUGAUGUUGGCACACUACCCAUUUGAUAUAAAGAAAAAACCACUAAUUUCGGCAAAAUUAAAA*

*>ENSG00000202183:81816575:81816775:X:1*

*AAAGACACAGAUAAUUUAAAAAUAAAGAGGUAAAGAAAGGUAUACAGUGUUAACAUUUAUCAAAACAAAGCGUGUUACCAAAAGUUAAUGUUUUCAGAGAAUGCUUUGGUUAAAAAGCCUUUACCAACUUGGGCUGUUUGGGAUAAUAAUGUUGGCCACUUUCCACAGUGGGUUAUAACAUUAAUAUAUAUAUAAGUCAC*

*>ENSG00000239171:30761612:30761812:21:-1*

*AUAACAUGAUAUUGAAAUAGCUAAGAUAAACUAAAUUAAUAGUGGCUUUAAAACAUGUGGUUUUGAUUUCUUAUUUUAAAAAAUCCAGAAUUAGACCAUUUUGUGGUUCGUAAGUGUGAUGAUUGGGUUUGCAUGCUCAUGAGUGAGACGUGCCUCCCUCAAACCUUGUUACAUCAUUAGCACAUUAAAUAUCUGACAUA*

*>ENSG00000238591:17080925:17081125:21:1*

*GAGACUCUGUCUCAAAAAACAAAACAAAACAAAACAAAUAAACCUCACCCCGAUCCUUUUGUAGAUCAUGAGCAUGAUGAUUGGGUGUUGACACAAAUAUGUGAAAUGUGCCAGCCUCAAACCUUCUUAUGACAUCAGAUCAGCACAUGACUCUGCUGGGAAGUCAUUAAUCCAUUAUACAUUAAGUAUCUGGAACUUUU*

*>ENSG00000239059:34650504:34650704:6:1*

*CCUCCCAAAGUGCUGGGAUUACAGACAUGAGCCACCACACCCAGCACAUUGUUAAUUCUUUAAAAUAUCGAUCCUUUUGUAGUCCAUAAGCGUGAUGAUUGAGUGUUCACACUUAUGUGAGACACGUGCCCUCCCUCAAACCUUGUUAUGACAUUGGCACAUUAUUUCUCUGAUGUGAAAAAAAAUAAAGUUAAAAAAAA*

*>ENSG00000206661:4985732:4985932:8:-1*

*UCUUAAAUUUGCAUGAGACCACAAAAGACCCCAAAUAGCCACAAUCUUAAGUAAAAAGAACAAAGCUGCUGCAGCCAGUUAAGCCACCUGAGUUCCUUUCCUCAUAGGGGUCCAAUGUGCAAUGGCUGCAAACAGCCGCUUCCCUGUAGUAUAUGCUGCCCAUUUCUUGUAUGGGUUGCUCUAAGGGACCUUGGAGACAG*

*>ENSG00000251802:34931953:34932153:7:1*

*UCUCUCCUCCACUCAUUCCCAGAGAUAGUGAUAUGUUGGUAUUAACAACACAUCUCUUUAAAAAUUCAAUUACAUUUGCAUCUAUUUGACAGACUAGAAUAAUUACUAUCUGCUGCUAAGGUUUCCCCUGCAGAUGCAAGAAAAAAGUGACCUUACAUUUUCUGUCUGUCUGAUUGUGGCAGCCAAGAUUGAAUAGGGAA*

*>ENSG00000200016:173835679:173835879:1:-1*

*UUGUUUUCUAGUUGUGUCCCCAAGGAAGGAUGAGAAUAGCUACUGAAGUAAGUUGAAAAUUCCCUCUCAAAAAGGUUUAAAGCCAUUGGAUGUGCCACAAUGAUGACAGUUUAUUUGCUACUCUUGAGUGCUAGAAUGAUGAGGAUCUUAACCACCAUUAUCUUAACUGAGGCACCCAAAAUGGUGAGUUGGGGAACAUA*

*>ENSG00000207444:71864970:71865170:14:1*

*UUAGAUUAAUUUUCAAAGGUUAUUAUGUUCCAGGUAUUACUUGAAAGUUCUGAACUAUUUUUGUGCAUAAAAUUAUGUUUUCACUACAAUCAUGGCAAUAUUUUUUGUCAACAGCGGUUCACCUAGUGAAUGUUGAUACUUUGGGUUUGAGUGAAAAUGAAUUGUAGCAUUUUCUUUUGCAUUAAUAAAUACCGUACUCA*

*>ENSG00000206989:137896648:137896848:5:-1*

*AAAACCUUGGUAAUACCCCGCCCCCGGCCACCAUCUUUGUACAUGUGUCUUUGCAGCUUAGUGCAUCUCUUUGACAAAGAUUUGUGCAAUGAUGUAUUUUAUUCAACACAUCAUUCUGAAAGAACGUGUGGAAAACUAAUGACUGAGCACAAACCUGUUGAAGAUGCAGUUCUUCUUGGUAGCCUAAAUAGGAUUAUCUU*

*>ENSG00000252834:83870229:83870429:4:1*

*UUAGAAACCUCCACACUGUUUUCCAUAAUGGCUGCACCAAUCUACAUUCCCACAAUCAAUAUACAAGGGUUCCGUUUUCUGCACAUCCUCACCAACAUUUAUUAUGUUAACUUUUGAUAAUAGCCAUCCUAAUGGGUGUGAAGUGGCAUCUCAUAGUGGCUCUCAUUUUCCAUUUCCCUGAUGAUUAAUGAUGUUGAAUA*

*>ENSG00000238656:113576033:113576233:8:-1*

*GAAAAAUUCUUUUUUAGUUUAAAAGUGUGGUGAUUAGGUUUUCACACUCGUGUGAGCUGUGCCUUCCUCAAUUCUUUUUUAGUUUAAAAGUGUGAUGAUUAGGUUUUCACACUUGUGUGAGAUGUGCCUUCCUCAAACCUUGUUACAACGUCUGCACAUUACCCAUCGGACAUGGAAAGAAAAAAGAAAUGAAAAUGGAA*

*>ENSG00000207165:153628553:153628753:X:1*

*CUUGCCAGCAGGUAGCUGAAGCUGGCAGAGGAGCCCAGUGGCGCCUUUUCAGUGGUUCUUGGGAUGCUCCGCAGCCAAUUAAGCCGACUGAGUUCCUUUCCUCAUGGGGACCCAGUGUGCGAUGGCUGCACACAGCAGCUUCCUUGGUAGUGUACGCAGCCUGUUGGUUGUAUGGGUUGCUCUAAGGGACCUUGGAGACA*

*>ENSG00000251744:140120599:140120799:8:1*

*AUCUUCUACAUUGUGCAUGUUUUAUUGAACGUAAGACAGUGAUAAUGAAACACUCCUAUAACCAUAUGCUCAUAGGUCAUUUUGUAGGGCUUGUGGGCUGUGAAACCAAGAGCUCUUAACGCUAUGACCAAAUAUUGAAGUCUUCCACAGGAUGUGAUGGCCACUAGGAAACAAGACUUAGCCACUCACACUCAUGACAU*

*>ENSG00000238732:43305624:43305824:10:-1*

*UGGAAUCCAACAACACAUAAAAAGAUUUGUAUAAAAAUUUUCACAUGGUGAUCUUUUCGUAGUUCAUAAGUGUGAUGACUGGGUAUUCAUGCAUGUAUGUGGGAUAUGCCACCCUUGACCCUUGUUACAACAUUAGCACAUUAACCAUCUGACAUGAAAGGAAAAAACAAAACAAAAAAACACACACAUUUCCAUAUGUU*

*>ENSG00000199782:25430692:25430892:15:1*

*GUCAUCCUGGUAUCCUGCACUGAGGUGUGGUGAGUCCAUCCAGGUCCCUCUGGAUGUGUGAGUGGGGAUGGGGGUGUCCUGGGUUGGGUCGAUGAUGAGAACCUUAUAUUGUCCUGAAGAGAGGUGAUGACUUAAAAAUCAUGCUCAAUAGGAUUACGCUGAGGCCCAACUUAGGUGAGAAUUUUGAAAGAGGAUGCUGG*

*>ENSG00000238917:7480045:7480245:17:1*

*UUGCUCUUGUGCACGCUUUCCAGUCUUUCAGCGUAAGCCAGAGUCAUUCCCAAGGAUGCUGGUUUCUCUCUGGGGGAAGAGCUGCUCUGUGAUGGAGCCCAUGCGUGUCAUCUGAGCCUCUGGCUUCCCUGCCAGUGCAGCCCUGGCAGUGUCCUACUUCCCAGGGCUGUUGUCUGCCUGGCGGGAAGGUCCUGGGCAAA*

*>ENSG00000252592:82282980:82283180:11:-1*

*UGUUUUGAGUGAUGAGUUCAUGAAUGUGUCUUGCACUGUUAGUAAAUAAGUGAAUAAACUGAUCUAUAUAUGGCGCAAUAACAAGAGUGUAUCAUGAAUGAAAGAUCAUGAUUAAUCCGAUUUUGUUGAUUUGAGACCCAAAAAGAGAGAGAAAAUAAUACCAGGCCAUUUGGCUCCAGAAUACAGGUCUUAACAGAUAU*

*>ENSG00000238811:112244190:112244390:X:-1*

*GUUUCAUUUUUCUUGUUGAGAAUAAUUUACACUGCUCCAUUGCUGGAACUGACUAAAUCCUUUUGUGGUUCAUGAGUGCGAUGAUUGGUUUUUCACACAUAUAUGUGAGAUGUGCCUCCCUCAAACCUUGUUAGGAGUAGAAGGUAGGCACAUUACCUAUCUGACGUGAAAAAAAGAAGAGAAAAAAGUAACAUAUCUAA*

*>ENSG00000206948:153996731:153996931:X:1*

*UUAGAGCUGGUUCUUACCAUCUGCCAGCCUUUGAUGGCACUCCACUUGCUUCAUGUCGUGGGAAAGAUGCUUUCCAAAGUGUUGAGUUCAGUCCAGGGCAGCUUCCCUGUUCUGUUAAUUAAACUUUGGGACAUUAAAAUGGGCUAAGGGAGAUGAUUGGGUAGAAAGUAUUAUUCUAUUCAUUUGCCUCCCAGCCUACA*

*>ENSG00000200398:25458714:25458914:15:1*

*CUGGCCUCCUGCACUGAGCUGUGGUGAGUAUAUCAUGGUCCUGCUGGAUGCAUGUGCAGGGAGGGAAGGUGCCUUGGGUUCAGUCAGUGUCGAGAACCUUAUAUUGUUCUGAAGAGAGGUGGUGACUUAAAAAUCAUGCUCAAUAGGAUUACGCUGAGGCCCAGCCUAGUUGAGAAUUUUGGAAGGGGACAGUAGGAUCC*

*>ENSG00000239044:129984170:129984370:7:1*

*UAAUUAAUUAUAAGUGAAAAGCCAAAAUGAGGUAUUAGGUCAAGCAUAGCUAUGUGUCUCCGCUUCAAAUCCAGCCAUAAGCUGAUAAUUGCAGUUCAUAAGUAUGAUAAUUGGGUGCUCACGCAUGCGUGUGUGAGAUGUGUCACCCUCAAACCUUGUUAUGACGUCAGCACAUUACCCGUCUAACAUGAACUUAGAAG*

*>ENSG00000239170:233543614:233543814:2:1*

*UUGUGAAGAUAAAGUCAAGUCCCUACUUUAUACUCUGCACUAAAAGAAGUGCACUUAGAUCCUUUUGCGGUUCAUAAUGUGAUGACUAGGUUUUCAUGUCCAUGUGUGAAGUGCGCCUUCCUCCAAUCCUGUUAAGAUGUCAGCACAUUUGCCUUCUGACAGGAAAGAAAAAAAAAGUGAAAUAAAUGAGCUUAUUUUUG*

*>ENSG00000252994:104563622:104563822:10:1*

*GGAUUCUAGCCUUGAGCCACCGCGCCCAGCCAGGUUUUCCUUUUUUUUAAAGUUUUCUUCUUUGUGCUUACUUCAGCAGCACCUAUACUAAAAUUGGAAUGAUACUGAGAUUAGUGUGGCCCCGGCCAAGGAUAGCAGGCAAAUUUGUGAAGCACUCCAUAAAAAAAAGUAAAAAAUGUAAAAAUGAACUUUCUUCCUUG*

*>ENSG00000252353:3093488:3093688:18:1*

*AACAGACGUACACACAGAGAAGCAUAGAAAAAAGUCUAAAAAAGUAUAUACCAAUAUGUACAUCAAAAAGCCAUAAGAAGUGUUACAGCUCUUUUAGAAUUUGUCUAGCAGGCUUUCCGGUUUUUGCUGGAAAGACUCCCCACCACCACCACCAAAUUAAGAAAAGCCGUAAGAAUGUGUGAUUAGUCCUUUCUGAGUCU*

*>ENSG00000207134:155328265:155328765:1:-1*

*CUGGACUUAAGCGAUCCUCCCACCUCAGCCUCCCACAGUGCUGGGACUACAGACCUGAGCCACCAUGCCCAGCCAAAUUUUUUAAUUAAGUGCUUGCUUUGACAGCAUAUAUACUAAAAUUGGAAUGAUACAGAGAAGAUUAGCAUGGCCCCUGUGCAAGGAUGACAAACAGAUUACUGAAGCAUUUCAUGUUUUUUUUU*

*>ENSG00000207452:86595263:86595763:5:-1*

*UUUCUGACUCCUGGAACAAUUCACAUCAACAUCACACCUACCACCAGAAAGCAGGUGUCAAACGUUCCCCUUCUUUGUAAGAACCAUAUACAGUACUCACUUCUGCAGCACAUAUACUAAAAAUUGGGAUGAUAAUAGAGAAAAGAUCAAUAUGACCCCUGCUCAAGGAUGACAUGCAAAUUUGUGAAGCAUUCCAUAUU*

*>ENSG00000206652:16840402:16840902:1:-1*

*GCAAGUGACCGUGUGUGUAAAGAGUGAGGCGUAUGAGGCUGUGUCGGGGCAGAGGCACAACGUUUCAUACUUACCUGGCAGGGGAGAUACCAUGAUCACGAAGGUGGUUUUCCCAGGGCGAGGCUUAUCCAUUGCACUCCGGAUGUGCUGACCCCUGCGAUUUCCCCAAAUGUGGGAAACUCGACUGCAUAAUUUGUGGU*

*>ENSG00000252703:132395608:132395808:11:1*

*AUAUUUUAAGUUCUGGGAUACAUGUGCAGAACAUGCAGUUUUGUUACAUAGGUAUACACGUGCCAUGGUGGUUUGCUGCACCCAUCAACCCGUCACCUACAUUAGGUAUAAAAUUGGAACGAUACAGAGAAGAUUAGCAUGGCCCGUGCGCUUUUCAAAGGAAACAAUUUUUUGGAGUUCAAAUGCUUAAAUGUCAUUGA*

*>ENSG00000199789:99910570:99910770:14:1*

*AACUAUUAGAUUACAAGGGCACUUUUAUUGGAUAAUCUGCCCAGCACAAACUUACAGCUAAAAUGAAUUUCCAUGCUCGCUUAGGCAGCACAUAUACUAAAACUGGAAUAAUACUGGGAAGAUGAGCAUGGCACCUGCGCAAGGAUGACACACAAACUAGUGAAGACUUCCAUAUUUUUGUCCUAUGGCACUGUAGGAUG*

*>ENSG00000238415:43122599:43123099:9:-1*

*AGCUAAGCCUCAUCCAUGACUCAUGAAUAUCCAUGUAUCAAACAGGGCUUUGUACUUAUUUCAACAGCACAUAUUUUAAAAUUGGAUCAAUACAGAGCAGAUAAGCAUGGCUACUGCCUAGGGAUGGCACACAAAUUCAGAAAACAUUCCAUAUUUUGCCUAGUCCCAGGUAGGCCAUUUGACUAUUUGUUGAGUAGCUC*

*>ENSG00000222238:103067207:103067407:10:1*

*CAGGGGUCAGCUGUGCAGAGCACCCCCACCCCCACCAUUUCCAUUCACACCCUUUGGGUUCUAUAGAGUUCCACUCCUAUCACCUCUCGGUCUUUUGGCUAAGAUCAAAUGUAGUAUGUUUUUACCAGCUUAACAUCUGAUACAUCCUCUAUCUGAGGACAAUAUAUUAAAUGGGUUUUUUGAGCAGGGGGAUGGAAAAG*

*>ENSG00000207483:28956785:28956985:10:1*

*CAGGAGUUUGAGACCAGGCGGGGCAUAAAAGCGAGACCCUGUCUCUAUUAAAGAAAAAAAAAGUGCUUGCUUUGGCAGCACAUAUGCUAAAAUUGGAAGGAUACACAGAAGAUUAGCAUGGCCCCUGCGCAAGAACGACACGCAAAUUCAUGAAGCGUUCCAUAUUUUUAUCUGCCAGCCAUCACCACCCGAUUUUGAUU*

*>ENSG00000252220:83415575:83415775:12:1*

*UCUAGGGUGUUUUCUAAAAUUUAUAAGUGAGAUCAUAUUGUGCCUCUAUUUCGAAUCCUCCAUGAUGUUCCUUCCUCUUCAAAAGACACUCCAGGGCUCAAUACAGCAGCAUAUACACUAAAAGGGAAAGGACACAGAGAUUUGAUGGCUCUUGUACAAGGACGUCACAUAAAUUCAUGAAGUGUUCCAUAUUUGCACAG*

*>ENSG00000206802:27562934:27563134:21:1*

*GCUAAUUUUUUUAUGAGCUUGAGCCUAGGAGUUCAAUACCAGUCUGGGCAACAUAGUGAAGCCCCAUCUGUACAAAACAAUACAGAAAUUAGUGCUUGCUUCAGCAGCACCUAUACUAAAAUUGGAAUGAUACGGAGAAGUUUAGCACAGCCCCUGUGCAAGAAUGACACGCACAUUCAUAAAACAUUUCAUAUAUUUGU*

*>ENSG00000251787:142519971:142520171:3:1*

*GGAUUUACUGUCUUACAGCCAUCUUUGUAUGCUAGCACAUGUAGAUGUUCUUCAUUCUUUUUAAAAACAAUGUUGAGUAAUGUUACAGCUCUUUUAGAAUUUGUCUAGCAGGCUUUCCGGUUCUUGCGGGAAAGUCCCCCCCAAAAAAUAUUAAAAAAUAAAAAAUAAAAACAAUAUUGAGGAUUUUCUAAAAUUUUUUU*

*>ENSG00000222490:90233720:90233920:3:1*

*UUAUGCAAAAAAAGGGUAUGUAUUCUGUAGAUAUAAGUGUAUAGUGCUACUCAAAGUGAUUUAGUGUUCGCCUUGGCAGUACAUAUACUAAAAUUAGAAUGAUACAGAGAUUAGCGUGGCCCCUGUGCAAGGAUGACACGUUCUAAAAAUUUAUUAAAAUAAAAAAAAAGAUUUAAAGAGCAUGAUGGAAUUUGGUAUAG*

*>ENSG00000207204:98392101:98392601:7:-1*

*UCACUGAGCAUACUGUUCCCAUGUUUUAUUAUGUUGUGCCAUGUGAUAGAAUUUCCAUCUUUUUUAUGGCUGAAUAAUAUUCCAUGGCAUGGUGCUUGCUUUGGCAGCACAUAUACUAAAAUUGGAAAGAUACAGAGAAGAUUAGCAUGGCCCCUGCGUAAGGAUGACAUGCAAAUUCGUGAAGCAUUCCAUAUUUUUUU*

*>ENSG00000201919:22314217:22314417:1:1*

*CUGUGAAUAGCCACUGUACUCCAGCCUGGGCAACACAGCAAGAGCCUCUCUCUUAAAAAAAUGAAAAUAAACUAAAUAAAAUAAAACAGUAGUGCUUGCUUUGGCAGCACAUGUACUAAAAUUGGAAUGAUAUAGAGAUUAGCACGGCCCCUGCGCAAGGAUGACAUGCAAAGUCAUGAAGGCUUCCAUUAAAAAAACAU*

*>ENSG00000239053:10884234:10884434:2:1*

*GAGUUUGAGGCUGCAGCGGGCCAUGUAACGCCAUUGCACUGCAGCCUGGGUGACAGAGUGCGACCCUGUCUCGAAGGAAACAAUACAGCUUUUUUAGAAUUUGUCUAGGAGGUUUUCUGGUUUUCACCAGAACCAACCCUUGCCUGCAAAUGUUUGAUAGAUACUCUAAAAUUACUCUCUAGUGAGGCUGUACCAAUCAA*

*>ENSG00000199849:164320449:164320649:1:1*

*CAAAAAUUGACAGGUGAGACCUAAUUAAACUUAAGUGCUUCUGCAUGGCAUAAGAAACUAUCAUUAUGGUUUCUCUUCAGAAUGAAUAAAUCUUUUGCCUUUUAUUAAAGAUUUCCAUGAAGAGGAACAGAUGGGAGUUAGUAAGAAAUGUUUUGAAGUCCUGCUUGAGCAGGGCCAAGUAAAUUAAAAAAAAAAAAACU*

*>ENSG00000200796:38862296:38862496:1:1*

*AUGUUAACAGGUGACACAAUUACAAAAAGUGCUAGUGCUUACUUUGGCAGCACAUAUACUAAAAUUGAAAUGACACAGGGAAGAUUAGCAUGGCCCCUGCACAAGAAUGACAUGCAAAUUUGUGAAGUAUUUUGUAUUUUAGGCCAGAAAAGAAUGAGAUGAUAUAUUCAAAGUACAGAAAAAAAAAUUGGCAACCAAGA*

*>ENSG00000200393:54156847:54157047:19:1*

*CUCCGGCCUGGGUGACAGAGCGAGACUCCGUCUCAAAAAAUAAAAAUAAAAUAAAAAUAAAAUAAAAUAAAAUAAAUAAAAUAAAAGUGUAGUGCUCGCUUCAGCAGCACAUAUACUAAAAUUGGAAUGCUACAGAGAAGAUUAGCAUGGCCCCUGCACAAGGAUGACAUGCAAAUUCAUGAAGCGUUCCAUAUUUUGUG*

*>ENSG00000199514:38345024:38345524:3:-1*

*AUGAUCCGCCCACCUCAGCCUCCCAAAGUGCUAGGAUUACAGGCGUGAGCCACCGCGCCCGGCCUGAUUGAACUUUUAAAAGAUAAGAAGGGUGCUGGCUUCGGUAGCACAUAUACUAAAAUUGGAACGAUAUAGAGAAGAUUAGCAUGACCUCUGCACAAGGAUGACACACAAAUUUGUAAAGUGUUCCAUAUUUAAAU*

*>ENSG00000222355:53843743:53843943:7:1*

*CCUACCAAGUCUGAGGAGCAAGAGAUUAAAGGAUCUGAGGAGCAAGAGAUUAAAGGAUCUGAGGUGCAAGAGAUUAAAGGAGAGAAUCCCUUCUAGGUCUUUUGGCUAAGAUCAAGUGUAGAAUCUGCUUUUAUCAGUUUAAUAUCUGGUACUUCCUCUAUCCCAGGACAAUAUAUUAAGUGGAUUUUUGGAGCAGGGAG*

*>ENSG00000202300:203287884:203288384:1:-1*

*GACUGAGAGAGCCUGGGAUUCCCAUCUUAGGAAUACAGAAUGGCAAAGAAAGUCUGCAGGAAGGGAAUGGCAUGAUGAAAAUAGAGUUUGCAUGCUCGCUUCGGCAGCACAUAUACUAAAAUUGGAACGAUACGGAGAUUAGCAUGGCUCCUGCGCAAGGAUGACACACAAAUUUGUGAAGCAUUCCGUAUUUUUAUAAA*

*>ENSG00000202423:79109537:79109737:2:1*

*UUAUCAUUCUACGUUUCUUUAAAAAUGCAAUAUGUGUGUUCACUUCAGCUGCACAUAUACUAAAAUUGGAAUGAUACAGAGAAGAUCAGCAUGACCCCUGCACAAGAUGGCAUGUAAAUUCUUGAUGCAUUCCAUUUUCUUAAAAAAAAAGCGAUAUCUUCUUGUGUAGCUGUGAAGAGACUAAGUAGAUUAUUUUUUUA*

*>ENSG00000206843:138999797:139000297:7:-1*

*UAGAUAUCUGGAUAUUAAAUUUAAUACUAGACAGGGAAAAUGUCACAAAGAAAAAUGAGGGACAUCAUAUUGGUAAAAGAACAUUAUAUCAGUGCUUGCUUCAGCGGCACAUAUACUAAAACUGGAAUGAUACAGAGAUUAGCACCACCCCUGCGCAAAGAUGAUACACAAAAUUGUGCAGCGUUCUAUAUUUUUUAAAA*

*>ENSG00000252417:15934340:15934840:1:-1*

*AGUAUCCAUCAACUGAUGAAUGGAUAAAUAAAAUGUGGUAUAUCCAUAUAAAAGAAUAUUAUUUGGCAACCGAAAAAGAAGUAUUAGUGCUACAGUUCUUUUAGAAUUUGUCUAGCAGGGCCAGGCGCGGUGGCUCACGCCUGUAAUCCCAGCACUUUGGGAGGCCGAGGCGGGUUGAUCACGAGGUCAGGAGUUCAAGA*

*>ENSG00000252654:50521430:50521630:20:1*

*AGUGACCCUCAUCUAAUCCUUGGAUCCCCAUCCAGCCUUUCCACAAACUCUAUGGGUUCUGACCUUAAAAUAUAGCCCAGUGUUACAGCUCUUUUAGAAUUUGUCUAGCAGUCUUUCCAGUUUUUGCCGGAAAGCCCCUCUCACGCUUAAAAUAAUAAUAAUAAUAAAAUAAAAUAUAUUUUAAGUCUCACGCCUGCAAU*

*>ENSG00000238923:7052901:7053101:12:1*

*AAAAAAGUCACCUAAGCUCACCCUCAUCAAUUGUGGAGUUCCUUUAUAUCCCAUCUUCUCUCCAAACACAUACGCAGCAGUGUUACAGCUCUUUUAGAAUUUGUCUAGUAGGCUUUCUGGCUUUUUACCGGAAAGCCCCUCUUAUGAUGUUUGUUGCCAAUGAUAGAUUGUUUUCACUGUGCAAAAAUUAUGGGUAGUUU*

*>ENSG00000206926:15474377:15474877:3:-1*

*CAGCACACUCUCCGGGGUAGGUCAAGCUCUCUAACCCUCAGUCUCCGUACCUUUUUUUUUUUUUUUUUUUUUUUAGAUAAUAGCACCUUUCGUACUCACUUCGGCAGCACAUAUACUAAAAUUGGAAUGAUACAGAGAAGAUUAGCAUGGCCCCUGUGCAAGGAUGACACACAAACUCAUGAACAAUUCCAUAUUUUUAA*

*>ENSG00000252141:52139456:52139656:13:1*

*CCAAACUGUGGCCCAUGUAAUGUGACUCAGGUCUGUGAAAACUUGUCAAGUGGUACACCGAAAAGCUUCAUUUUAAGAAUAUCCGUUUCCAGUGCUCGCUUCGGCGGCACAUAUUCUAAAAUUGGGAUGACAGAAAAUUAGCAUGGCCUCUGGGCAGAAAAAUAAAUACAAGUAAAUAUAAAUCUGUUUCCAGAGUUUUA*

*>ENSG00000207343:20770847:20771347:22:-1*

*UUCAGUUUCGGGUGAUGAAAAGAUUCUAGAAAUAGAUGGCAGUGAAUGUACUUAAUGUCACUAAACCGUGCACUUAAAAAUGAUUAAAAUAGUGCUCGCUUCGGCAGCACAUAUACUAAAAUCAGAACGAUAGAAGUUUCUCAUGGCCUCUGCCCAAGGGUGACACGCAAAUGUGUGAAGCGUUCCAUAUUUUUAUUUUU*

*>ENSG00000251748:33868447:33868647:9:1*

*UUUUCCCACAUACUAUUAUUGAACAUUUUAAAGAUUUGUAGUUGGCAGACUUUUGACACUUUGAGCAAGUUAAUAAGAAAGCAUGUUUUAACCACCCUUUUCUUGGGGUUGCGCUGCUGUCCAAGGAUUGCAUAACAAGGGCAGCACUGCUAAUGCCUACACAACACUCCCGCCUCAGCUAGAGCUUUGCUUUACCUUGG*

*>ENSG00000252588:73741993:73742493:13:-1*

*UAGUGAGAUCCUGUCUCUACAGGGGACAAAAAAAAAAAAAAAAAAAAAAAAAAAAAAAAAGUUUUUUUAAAGAAACAAACACAUACUUAUUACAGGUCUUUUAAAAUUUGUCUAGCAGGUUUUCCAAUAUUUACUGGAAACCCUCCCCCACCUGCCACACACACAAAAGAAAGACACAGACAUGUAAACAAAUAAUAAUA*

*>ENSG00000200887:30588258:30588758:10:-1*

*ACACACCCCAAAAGAUGUGACAGGACUUGUUACGUAAUGAGUCUCUGAGAAGAUCAGGGAAGGCUUCCCAGCUGGUUUGAAAAUGGUUUGAGUGCUCGCUUCAGCAGCACAUGUACUAAAAUUGGAGUGAUAGAGAGAAGAUUAGUAUAGACCUGCACAAGAUGACAGGCAAAUUCGUGAAGCAUUCCAUAUUUUAAAAA*

*>ENSG00000251804:142126302:142126802:3:-1*

*GUUUAAAAAAAGACAGUGCUCUCUUCAGCAGUACAUAUACUUAAAAAAAAAACUGGAAUGAUACAAAGAAGAUUAGCAUGGCCCUUGCGCAAGCAUGAUAUACAAAUUCAUGAAGCAUUCCAUGCUAAAAAAUAGUAAUGAUAAAAACAAAAAAGUAGGCCGGGCACAGUGGCUCAUGCCUGUAAUCCCAGCACUUUGGG*

*>ENSG00000202167:149146485:149146985:1:-1*

*AUGGCGCCGGCCAAGCAGAGGGGAAGGUGGUGACCCUGAGCCUGCGGCUACGGGACAGGAGGCUGUACUGUCCCUCCUCUCCUCUUACCUGGCGGGGGACAGACCGUGGUCAGGAAGGGGGUUCUCCCUGGAUGAGGCUAGUCCACCGCACUGCGGCUGCACUGGCCCUGCGAUUUCCCCACAAGCGGGGCCCUUGUCUG*

*>ENSG00000201591:39282581:39282781:11:1*

*CAUAACACUGUAAGCAUUAAAUCUCUGAUAUGUAAAUCCAGAUACAAAUCACUGCUUUAUGGAGUGUUAGAGUUAUUGCUUACUUUAGUAGCACAUAUACUAAAAUUGUGAUAAUACAGAGAAGAUUUGCAUGGCCCCUGUGCAACAACAACACACAGAUUUGUGAAGCAUUCUAUGUUUUACAAAUUGAAAAAAAAGGC*

*>ENSG00000252898:174367514:174368014:4:-1*

*CAUCUAACAACUGUCUUGCUUUGGUGCAUAGCAGAAGUACUUUAUAUGUGCUUCUCAUUUUGUGUCACAGAAUAUUAAAAACAUGUACACUGUGCUUGCUUCAGCAGCACUUAUACUAAAAUUGGAAUGACGCAGACAAGCAGGCCCCCUGCACAAAGAUGAUAUGCAAAUUUGUGCAGAGUUCUAUAUUUUGCUUUCCU*

*>ENSG00000199963:39392450:39392650:1:1*

*GCAGUGAGCCAAGAUCGUGGCACUACACUCCAUCCUGAGUGAUAAAGCGAGACCCUAUCUCAACCAAUCAAUCAAUAAAAGAAUGCUUGUGGUGCUCGCUUCAGCAGCACAUAUACUAAAAACCGGACCAAUACAGAGAAGAUUAACAUGGUCUCUACACAAAGAUGACACGCAAAUUCGUGAAGCAUUCCAUACAAAAC*

*>ENSG00000212456:147994078:147994278:1:1*

*GGCAAGUGACCGUGCGUGUAAAGGGUGAAGCGUGUGAGGCUGUGGCGGGGCGGAGGUGCAAAAGCUCAUACUUACCUGGCAGGGGAGAUACUAUUAUCUAAACGAAGGUGGUUUUUCUCAGGGCGAGGGUUAUCCAUUGUGUUCCGGAUGUGCUGACCUCUGCGAUUUCCCCAAACGUGGGAAACUCGAUUGCAUAAUUU*

*>ENSG00000252825:51215876:51216076:1:1*

*UUCCUUGAAAAAUUGUGAAGACAUGCAGAUGUCAUCAAGAACAAAUCACUGAAUCCAAUAGCAUACUUUUGUAUUUAAAAAAUUAACUUAAGUGCUUGCUUUGGCAGCAUAUAUACUAAAUUUGGAACAAUACAGAGAAGAUUAGCAUGAUAAAUAAGAAUUUAAAACAUUAACUUAAUUGGAUUAUGAUUUAUAUGUAA*

*>ENSG00000252423:68865948:68866448:7:-1*

*GUUUUGUCAAGUGGUUGUUUACUUUGAUAACAUACAAACCAUCCAUCCUCUGUGAUUUCUUUCAACUUAACUUGUAAAGUUACCACCCUUUGUGCUUGCGUUAGCAGCACAUAUACUGAAGUUGCAACAGUACAGUGGAAAUUUGCAUGGCCCUUGCAUCAGGAUGACAUAGAUUUAUAAAGUCCAUAUUUUUUGUAGUA*

*>ENSG00000201119:145784187:145784687:6:-1*

*GUAUAUAUCUUACAUAUUGCAUGGGAGGUGAUCUGGGGAUACUUACUAGGCAGGUGAGAUAUUGUGAUUACAAAGGUGGUUUACCCAGAGUGGGCUUAUCCAUUGCACUCUGGAUGUUCUGACCCCUGUGACUUCCCCUAAUGUGGAAAACCCAAGGCAUAAUUUGUGGUAGUGAGGAAUUUCGUUUGCACUCUCCCUCA*

*>ENSG00000200213:37438751:37438951:21:1*

*UCCAGCUGCAUCCAUGUUGCUGCAAAGGAAAUGAUUCCAUUCUUUUUUGUGGGAACUAUGUUCUUUAGUUGCUUUAAACUAGUGGGUAGUAGUGCUCGUUUGGGCAGCACAUAUACUAAAACCGGAAUGAUACAGACAAGAUUUAGCAUGGUGCCUGGGCAAGGAUGACAUGCAAAUUCAUGAAGCGUUCCAUUAAAAAA*

*>ENSG00000207378:99474931:99475431:8:-1*

*GAAACUGUUUUAGGUUCUAUGUCCUCUGCUUAGAAGGCAGUAUAUUCUGUGGAACACUUUAUAAAGAUUUUCUGUUCAAAACAAUUGAGACGGUGCUCACUUCAGCAGCACAUAUACUAAAAUUGGAAUGAUACAGUGAAGAUUAGCAUGGCCCCUGUGCAAGGCUCACAUGCAAAUUCGUGAAGCAUUCCAUAUAUUUC*

*>ENSG00000239194:67424378:67424878:16:-1*

*CCUAAGCAGCCCCUGCUUCUCAUGUUCCCAGGAGGUGGGGAACUGGGGGGCUGGAACCAGGGAGGACAUCAAGAAGGGGUCAGGGAAACACCAUGAUCACGAAGGUCGUUCUUCCAGGGCAAACCUUAUAUCUAUUGCCCUCCAGAGUUGCUGACCCCUGCGACUUCCCCAAACGUGGGAAAGAAACGCAAUUGCAUAAU*

*>ENSG00000206616:24022344:24022844:15:-1*

*AAUUUUUAAAAAAUCUCUUUGAUAGACUUUUGACAUAGCUGAGGAAAGGAUCAGUGAACUGGAAGAUAAUUAAAUAGAAACUUUACAAUUGGUGCUUGCUUCAGCAGGAUAUAUAUUAAAAUUGGAAGGAUACACAAAAGGGUAGCAUGGCGCCUGCACAAGGAUGUCAAGCAAAUGUGUGAAGCAUUCCAUAUUUUUCA*

*>ENSG00000222426:34282321:34282821:9:-1*

*CUAAGAUCAAAUGUAGUAUCUUUUUUUUUUUUAAGACCAGUGGUUCAGAAUAGAAAGUGUAGUAUCUUUUCUCAGCAGUUUAACAUGUUCUAUUCAAGGACAACCUAUUAAAUGGAUUUUUGGAACAGGGAGAUGGAGUAGGAGCUUGCUCUGUCCGCUCCAUGCAUUGACUUGGUAGUGCAAGACCUCCAGGAAUGGUG*

*>ENSG00000199551:12379514:12379714:12:1*

*CUGAGCUUGGGAAUAUGUCAAUAGAAACUUCCAAAGCUGAAAAGCAGAUUUUUUAAAAAGACUGAAAAAAAGGGAACAGAAUAUCUAAGAACUGCGUUCAGUUCAGCAGCACAUAUACUAACACUGGAACAAUUCACAGAUCAGCAUGGCCCCUGCACAAGGAUGACAUGCAAAUUCGUGAAGCGUUAGUAUUUUUAUAA*

*>ENSG00000223096:179007184:179007384:2:1*

*GACAGGACACAUAGCAAAUAAGUAAUGGAGCUGGGAUUCGCGCUCAAGAAAUAUGACUCCAUGGCCUCACUUAAGUUCACACACUGUGAUACUCUGGUUUCUCUUCAGAUCAUAUAAAUCUUUUGCCUUUUACUGAAGAUUUCCGUGGAGGGAACAGUCUUUACCCAAUUUUUUGAGGCCUCUUGUUUAAGGAUUUUUUU*

*>ENSG00000206583:85220888:85221088:11:1*

*UAUAACUGGAGAAUUUGAUCUAAUGAGGCAGAUCAUGGAAUGCUUCAUUGAAGAAAUGAUUGUGCUGAGAUCUAAAAGACAAAGGUUAGUUCGGCGCUCACUUCAGCAGCACACAUACUAAAAUUGGAAUGAUACAGAGAAGAUUAGCAGGGCCCCUACAAAAGGAUGACACACAAAUUCAUGGAGUGCCCCAUGUUUUU*

*>ENSG00000238444:111903011:111903511:11:-1*

*GCAUUUGUCCCAACCUUACCAGAUAUCUUUCCAAGAUAAAAACGUAAUAUUAAGAACUGUUCUGUGCUCGCUUCAGCAACACAUAUACUAAAAUUGGAACGAUAUAGAGAUUAGCAUGGCCCCUGCGCAAGGACAACAAAUUCGUGAAGCAUUCCAUAUUAAAAAAAAAAACAUUAUUUGGUGACAAUAGUUAAUAACAA*

*>ENSG00000239088:17706371:17706571:3:1*

*GACAGGAAUCAAGGAAGGCAAAUCACAGAAGAAUGUUUGCCUCCACAGCCUUGAAUGAAGACUGGAACUUAUGUUACUGAAUCAAAGGUUACAGGCUCUUUUAGAAUUUGUCUACCAGGCUUUCUGGUUUUUACCGGAAAGCCCCGCUAUUUAAAAAAAAAAAAAAAAAAAAGGAAGAAGAAAGGAAAAAAAAACGGUUA*

*>ENSG00000199791:231451805:231452005:2:1*

*AAUUCUUUAACAUUUAAAAAUCAGGAAAUCAUGCUUGUUUCGGCAACACAUACACUAAAAUCGGAACAAUACAGAGAAGAUUAGCAUGGCCCCUGCACUAGGAUAACACAUAAAUUUGUAAAACACUCCAUUAAAAAAAAAUCAGGAGGCCGGGUGCCGUGGCUCACACCUGUAAUCCCAGCACUUUGGGAGGCUGAGGC*

*>ENSG00000207162:64963370:64963570:15:1*

*UGCACUCCAGCCUGGGGGACAGAGCAAGACUCCGUCUCAAAAAAAAAAAAAAAAAAAAAAAUUAAUUAAAAAAAAAUAAAGUGUUCAGCGGGUGCUCACUUCAGUGGUACAUAUACUAAAAUUGGACCAAUACAGAGAAGAUUAGCAUGGCCCCUGCACAAGGGUGACAUGCAGAUUCGUUAAGUGUUUUAUAUUUUUAA*

*>ENSG00000200369:27603955:27604155:10:1*

*AGACUUCACAAAACCCCAGGUAAGUUGGUUAGAUUUAACAGAGCUAAGCCUCAUCCAUCACUGAUCAGUCUUCAGGUAUAAAAGUAGGGAUUCAUGCUGGCAUCAGCAGUACAUAUAGUAAAAUUGAAACAACUUUGAGAAGAUCAGCAUGGCCCCUGCACGAGGAUGACACACAGAUCUGUGAAGUGUUGUAUAUUUCU*

*>ENSG00000206599:50267541:50268041:19:-1*

*AUUUAAAAUGUCUAUUAGCAGUGAUGUUUGAACUAGUCUUUUUGAGAAGUGUGAAGAGAACAGGAAAAGUCUGUCCCUAAAUAGCAGGGCAGUGCUCGCUUCGACAGCACAUGUAUUAACAUUGGAACGAUACAGAGAAGAUUAGCAUGGGCCCUGCGCAAAGACCACAUGCAAAUUUGUGACAUGUUCCAUAUAUUUUU*

*>ENSG00000251991:14500382:14500582:11:1*

*ACUUAAUUAUAGAAACCAAAGCUCUAAGAGAUUAUGGCUGUCAAAAACAAAUUCUUAAUGUCAUAGCUCUUUUAAAAUUUGUUUAGCAGGCUUUCUGGUUUUUGCCGGAAAGCCCUCUCACAAAAAAAAAAAAAAAAAAAAAAAAAGCAAAUCCUUAAUAUGAUUUUCUUCUCACAAAGGAAGCAAAUCCUGCUCUGCUC*

*>ENSG00000200560:61650363:61650863:17:-1*

*UAUUACAUAAAGAAGGAAGAUUCUAGAUGAAAUGUGGAAUUUUUGGUAUCAGCCUUUGUGGAGGAGAAACGGGAAAAAAACCCAGAUGAGAGUGCUUGCUUCAGCAGCACACACACUCAAAUCAGAACUAUACAAAGGUUAGCAUGGUCCCUGCACAAGGAUGACAUGGAAAUUUGUAAAGUGUUCUGUAUUUUCACAAA*

*>ENSG00000207278:3894468:3894968:18:-1*

*AGACAAUCUGGCCAACAUCUGAAUAGUAUCAUCAAAGAUCAUAUCAGAGGUAGAAAUGGAAGGCCUUGAAAAAGUAAGAGGAGUACUCACUUCAGCAACACAUAUGCUAAACUGGGAAUGACACAGAGAAGAUUAGCAUGGCCCCUGCACAAGGAUGACAUGCAAAUUCAUGAAGCAUUCCAUAUUUUUAAUCCGAAAAU*

*>ENSG00000222170:48765399:48765899:16:-1*

*UGUGCUCAGCUCUCAGUUUCUUAGUCUGCAAAAUGAAACUAAUUUCACCUACCUGUCUCACAAGGACAUCAUUUGGCAGCAUAUACACUAAAAUUGGUAAAAUACUGAGAAAACUAGCCCCUGUACAAGGAUGACACACAAAUUUAUGAAACGUUCCAUAUUUUUUAAAAAAAGAAUUAAAUAAGACAUUCUAGGUAGGA*

*>ENSG00000207114:171221792:171222292:3:-1*

*CAUAAAUUGAUAUGAGGGCUGUGUGAUUCUGAGACAAAGGGAAAACAUAUGUCUUCAUAGCCUAUGAUUUUUCAAUUUAAAAAUAAUAUUGUAGUGCUUGCUUCAGCACCACAUAUACUAAAAUUGGAAUGACACAGAGAAGAUUAGUGUGGUCCCUGUGCAAGGAUGACAUGCAAAUUCUUGAAGCAUUCCAUAUUUUU*

*>ENSG00000238558:135839034:135839234:9:1*

*CGGCCAAAAAAAUAUUUCUUAUGGCAUUAUCUGCAAAAGUUGAAUAUUGGAGACAACCCAAAUGUCAAUUAAUAGGGGACUGAUUAGUGUUACAGUUCUUUUAGAAUUUGUCUAGCAGGCUUUCUGGUUUUUGCCCAAAAGCCUCUCAAAAAAGUUUAAAAAAGAAAAAAAAUAGGGGACUGAUUGAAUAAACUAUGGCG*

*>ENSG00000223330:56469525:56470025:7:-1*

*CUGUUACUAGGUGCCUAUAAAUACCAUAGAUUUUUAAAUUCUCUGUAACUCUUCCUUAUUUCACUUAACCACUCUAUCUUAAAUUACUCAUGCUUGCUCCAGUAGCACACAUACUUAAGUUGGAACAAUAGAGAGAUUGGCAUGGCCUCUCUGAAAGAAUGACAUGCAAAUUUGUGAAGCAUUCCAUAUUUUUUUUAAAA*

*>ENSG00000252599:44381690:44382190:2:-1*

*CCCCAGAACUCCCCUUCUGCCCCCUCCCAAUAGCUAUUCUCUCCUUCUUCCCCAAAGGAAAUCACCUUCUUGAUUUUUAAUAUCAUAGAUUUGCCUGUUUACAAACUUUAUAUAAAUGGAAUCAUGCAUACAGAGAAGAGUAGCAUGGCCCCUGUGCAAGAAUGACAUGCAAAUUUAUGAAGCAUUCAUUAAAAAAUAAA*

*>ENSG00000199460:69409916:69410116:2:1*

*AUCUAAAAUUAUGGACUUGAAAGUACUAGUAAUUACUUUGUCAAUUUCCACAUAAUCUGGGUUGAAAACUAGGCUUUUUAAAAAUCACCGUGGUGCUCACUUCUGCAGCACAUAUAGUAAAACUGGAACAAUAUAGAGAAGAUUAGCAUGACCCCUGGGCAAGGAUGACAUGCAAAUUCGUGAAGUGUUCCAUAUUAAAA*

*>ENSG00000251718:11701234:11701434:2:1*

*CCAUGGCCACCCCUAGGCUCUCCUGACCUGUUCAACAGUGACCAUUCCUUCUGAAAUAUUAUUUCACUUAUAAAAAGCUUCCUACAUUGCUUCUCGGCCUUUUGGCUAAGACCAAGUGUAGUAUCUGUUCUUAUCAGUUUAAAAAAAGCUUCCUACAUCCAUACCAUACCUGACCCUGUUAUAGAAUGGCUAUUUGUAUG*

*>ENSG00000206725:230837939:230838139:2:1*

*AUUUUUCCCUGUCCUCACCAAUACAGGGUUUUAUCACUUAAAAAAAUCACCAAUUUGAGAAAAAAUAAGUGUUAGAAAAUAUCUUAUUAUUGUGUUCGCUUUGGCAGCACAUAUACUAAAACUAGAACUAUACAGGGAAGAUUAGAAUGGCCCCCGCGCAAGGAUGACAUGGAAAUUCAUGGAGUGUUCCAUAUUUUUAA*

*>ENSG00000201452:175485252:175485452:3:1*

*AAGGCAAUCUAGAUAUUCAAAUUAGGAAUAAUUUGAAUAGAAUUCUAAGUUUGUAUGUGCAUUAGAAGUGUUGAAGAUAUAAAAGUGAAGGAAAUGCUCACUUUGGUAUCACAUAUAAUAAAAUUGGAAUGAUACAGAGAUUAGCAUGGCCCUUGCGCAAGGAUGACAUGACAAUUCAUGAAGUGUUCCAUAUUAUUAUA*

*>ENSG00000251908:14402928:14403428:12:-1*

*UGAUCAUCCUGAAACAUCUAUUGCACAAAAAUGAAAGGAAAACAGGAUUACCACAGUAAAAAAAUACCGUUUUCAAAACAGAAGAAACAAUGCUUGCUUCAACAGCACAUAUACUAAAAUUGGAAUUACACAGAGAUGAUUAGCAUUGUCCCUCUGCGGGGAUUACGUGCACGUCAUUUGUGAAGCAUUCCAUAUUAAAA*

*>ENSG00000200917:106328107:106328307:4:1*

*UUGUAUUUCUAAAACAAAGGCAAAAAAAGAUUAUUUUAGAUAAAUAGCACAUACAUAUACUAAAAUUGGAACAAUACAGAGAAGAUUAGCAUAGCCCCUGCGCAAGGAUGACAUGCAAAUUCGUGAAGUGUUCCAUAUUAAAAAAAAAAAAAAGAAAAGAAAAUUCGCCACCAGCAGGAGUGCACUACAAGCAUAGUGAA*

*>ENSG00000222405:70837342:70837842:12:-1*

*AUAUAUUAUGGUACUACCAGGGAAGGAAACGUAAGUGAGAAAGCAAUAAAAUUUCAUGAAUAGUAAGCAUAUCCUUUAAGUACAUUUUCAGCUUCAUGCAGCCAGUGAGGUUUUUCUGAGGUGUGAUUAUUGCUAAUUGAAAACUUCCCAAUACCCGGUGGUGAUGAUUUGCAAUACAGUUGGCAUCGGAAAAUUUUGAC*

*>ENSG00000252826:149214951:149215451:1:-1*

*GCAAGUAACCGUGGGUGUAAAGGGUGAGGCAUAUGGAGCUGUGGCAGGGCGGAGGUAUGUGGACUGCCACUUAUCUGGCAGGGGAAAUCCUAUGGUUACCAAAGUGAUUCGUCCUAAGUUUGAGGCUCAUCCAUUGCACUCCAGAUGUGCUGUUCUCUUGUUGUUUCCCCUAAUGUGUGAAGCUCAACUGCAUAACUGAG*

*>ENSG00000207366:48810187:48810687:14:-1*

*UGUUGACAACUAGUAGAUAAUAUUCACAGGCUAUACCAUGGACACAGGGUUAAUAUCCUUAUUAUUUAAAGAACUCUUAAAAAUUGAAGGACAGUGCUUAUUUUGGCAGGACAUAUACUAAAAUUGGAAAGACACAGAAAAGAUUAUCUUGGCACCUAUGCAAUGAUGACUUGCAAAUUCGUGAAGUAUUCCAUAUUUUU*

*>ENSG00000252661:15255934:15256134:19:1*

*UUUAACACUAUAAGAUGUAUUUUGCAAGCCUCAUGGCAAGCACAAAGCAAAGGCCUAUAAUAGAUACACAACAAUGAAAAGGAAGGAGUCAGUGUUCGCUUUGGCAGCACAUAUACUAGAAAUGGAAUGAUACAGGGAUGAUCGUGGCCCCUGCACAAAGAUGACAUGCGCAUUCUUGAAGCAUCCCAACAUAAAACUUA*

*>ENSG00000207472:182951107:182951607:1:-1*

*CAAGGCAGGCGAAUUACUUGAGGUCAGGAGUUUGAGACCAGCCUGGGGCCAACAUGAUGAAAUCUCGUCUCUACCGAAAAUACAAAAAUUAGUGCUCGCUUCGGCAGCACAUAUACUAAAAUUGGAACGAUACAGAGAAGAUUAGCAUGGCCCCUGUGCAAGGAUGACAUGCAAAUUCGUGAAGCAUUCCAUAUUUUUCA*

*>ENSG00000221439:890237:890437:12:1*

*CAAUGAGCUGUUUUUUGUUUUAUUUCGUUAACAACAACAACAACAACAACAAAAACUGCUAACCAUCUUUUUCUUGGGGUUGCACUACUGUCCAAUGAGCACAUAGUGAGGGCAGUACUGCUAACGCCUACAUAACAUGCCUGCAUCAUCUAGAGCUUUGCUUUACCUUGGUACAAUUUUUGGAAGAAUGAAAAACUGCU*

*>ENSG00000201746:10223236:10223436:1:1*

*CUUUGUAGAUCCAAAGAGCUGGAUCUACAAGCAUGUACCACCACGCUUGGCUAAUUAAAAAAAUUUUUUUUAUAGAGACAAAGUCUCACAUGCUUGCUUCAGCAGCACGUAUACUAACAUUGGAACAAUGCAGAGAAGAUUACCAUGGCCCCUGCUCGAGGAUGACAUGCAAAUUCAUAAAGCAUUCCAUCCUUUAAAAA*

*>ENSG00000252988:73525717:73525917:2:1*

*UAAGAAAUUUGCCCAAGCUUACCAGGUUAGUGAUGAUGCCGGGAUCUGAACUCAUACUAUAGCCAUAGGCUAGAUUACAAAAAGUGAAAUUUCAUGCUCACUUCAGCAGCACAUAUACUAAAAUUGGAAUGAUACAGGGAAGAUUAGUAUGGUCCUUAAAAAAAGUGAAAUUUUUUAGGACAAAUAGAACUGGUUUUAGG*

*>ENSG00000252206:56745938:56746438:12:-1*

*CUGCCCACCUUUGCUUCCCAAAGUGCUGAGAUUACAGGUGUGAGCCACCAUGUCAGCUGGCGAGGCUUUUUAAAAGAUAGUUCCAAGUGUUACAGCUCUUUUAGGAUUUGUCUAGCAGGCUUUCAGGUUUUUGCCAGAAACCACCCCCACCCCCACCCCCACCAAAAAAAAAAAAAAAAAAAAAAGAUAGUUACAAAUGU*

*>ENSG00000212230:243244218:243244718:1:-1*

*AGCUGUUACUAGGUGCCUAUAAAUAGCAUAGAUUUUUAAAUUCUCCAUAAUUCUUCCUUAUUUCACUUAACCACUCUAUCUUAAAUUACUCAUGCUUGCCUCAGUAGCACACAUACUUAAGUUGGAACAAUAGAGAGAUUGGCAUGGCCUCUGUGAAAGAAUGACAUGCAAAUUUGUGAAGCAUUCCAUAUUUUUUUAAA*

*>ENSG00000238697:77156474:77156974:6:-1*

*UAGUCAGCCAUUGCUUUCAGUCAUCUGCGUAUUUCAACAUAAUACUUUCUACUUACGAUGGGUUUAUUGGAAAGUAACUCUAUUGUAAGCCAAGGAGCAUUUGUACUAAAAUUGGAAUGAUAGAAUAUUAACAUGGUCCCUGCACAAAGAUGACAUGAAAAUUUGUGAAUCUUUCCAUAUUUUUCAACCCAAAGAGUUCA*

*>ENSG00000200877:85864177:85864377:11:1*

*GUAACCAGGUUGAAAACCAGUGUCAUAAAUGCUCAAGGAAUAUUUGUUGAAUGAAUGAAUGAACCAAAUGGGAUCAGAAAGAACAAACCCCAUGCUGGCUUCGGCAGCACAUAUACUAAAAAUUGGAAUGAUACGGAGAAGAUUAGCAUGGCCCCUGCACAAGAAUGACACACAAAUUCAUGAAGUGUUCCAUAUUAAAC*

*>ENSG00000206906:19119435:19119635:14:1*

*CUCAAGUAAGUUGGUUAGAUUUAACAGAGCUAAGCCUCAUCCAUCACUGAUCAGUCUUCACGUAUAAAAAUAAGGAUUUGUGCUGGCUUCAGUGGUACAUAUAGUAAAAUUGAAACAACGUUGAGAAGAUCAGCACGGUCCCCACACAAGGAUGACAUAGAAAUCUGUAAAGUGUUGCAUAUUUCUUACAGUCCCCAAAA*

*>ENSG00000201070:99398837:99399037:2:1*

*AAUAAUGGCUGAGAACUUUUAAAAAUUAGUGACAGAGCUUUAUGCAGUGGCAGUAUCAUAGCCAAUGAGGUUUAUCUGAGGUGUGAUUAUUGCUAAUUGAAAACUUUUCCCAAUACUCCACCAUGAUGACUUGAAAUAUAGUCAGCAUUGGCAAUUUUUGACAGUCUGUACAGACACUGAAUUUUAUAGUUAAAAAGAAG*

*>ENSG00000222266:134051108:134051308:5:1*

*UACUAAUUUAUUCAAACAAAUCGAUCUUUUAACAACUGACAUUUUAAAAGUUCUGGGUGCAUUUUUGCUCGCUUCAGCAGCAAAUAUACUAAAAUUGGAAUGAUACAGAGAAGAUUAGCAUGCCCCUUGUACAAGGAUGACACACAAAUUCGUGAAGCAUUUUUAGGAACAGAAAACCGAACACUGCAUGUUCUCACUCA*

*>ENSG00000212609:20717439:20717639:21:1*

*UUUAAGAAACUAAGGAAAAAAAUAUAUACUUAACAUGGCAGAGGAGAUAUCAUAAUCACAAAGGUAGUUUUCCCAGGGCAAGCCUUAUCCACUGCAUUCCAGAUGUGCUCACCUCUGUGGUUUCCCCAAAUGUGGAAAACUGGACUGCAUAAUUUGUGGUAGCGGGGGACUGCAUUAAUACCUUCCUCUGGCUUUUUAUU*

*>ENSG00000212550:45294882:45295382:15:-1*

*UGAUAAAUCUCUACUAUCAAAACUUAUUUGGAUUCUUACCUGGCAGGGGAGAUGCCAUGAUCAUGAAGGUGGUUUUCCCAGGCUGAGGCUCAUCCAUUGCACUCCAUAGGUGUGCUUACCCCUGUGAUUUCCCCAAAUGCGGGAAACUCAACUGCAUAAUUUGUGGUAGUGAGGAACUGCAUUCGUGCUUUCCCCUGGGA*

*>ENSG00000202081:86194513:86195013:15:-1*

*GAGGCAACUCCAAAGUGAUGCUUAUAGUCUGUACCCUGCUGCAGGCUGCGUACAAACGUGAAUGAAGGCUUCUAUCAAAAGUCACUGAUGGGUGCUCACUUCAGUAGCACAUACACUAAAAUCGGAACAAUGCACAGACGAUUAGCAUGGCCUCUGUGCAAGGAUGAUGCACACAUUCGUGAAGCAGUCAUUAAAUUUUU*

*>ENSG00000252807:170976284:170976784:2:-1*

*UCUGACCAUGAGGAAAUAUGAGACAAACACACAAUGAGGAACAUGCAAUUCAAACAAAAAUGGUGCGUGGUGCUUGCUUUGGCAGCAGAUAUGUGAAAAAUUGAAAUGACAUAGAGAAGAUUAGCAUUGCCCUGCACGAAGAUGACACAAAUUUGUGAGGAACAAAAAAGGGUAUUGGUGGUGGGUAAUGGUGGAGUGGG*

*>ENSG00000207307:77453135:77453635:4:-1*

*AUCAUUUUAUUUUCAGUGGAAUAGGGAACCGAAGCAUUCAUACUUUUCUAGCUACUUACUGUGAAAGAAGGAUGCCUUAAAGAGCAACUUGGUGCUCGCUUCAGCAGCACAUAUACUAAAAUUGGAAUGUUACAGAGAAGAUAACAUGGCCCCCACGCAAGGAUGACACGCAAAUUCGUGAAGCGUUCCAUAUUUUUAGG*

*>ENSG00000200869:6923806:6924006:16:1*

*AUUUUCCAGAUAAGGAAACCGAGACUUUGAAACAUUAAGUAACUUGCAUAAAGUCAGACAGCUAGGAAGCAUAGUAUCUGAAAUAGGAAACCAUUGCUCACUUUGGCAGCACAUAUACUAACAUUGGAAUGAUACAGAGAAGAUUAGCGUGACCCCUGCACAACGAUGACAUGGGAAUUUGUGAAGCGUUUCAUAUUUUU*

*>ENSG00000200070:59504608:59505108:15:-1*

*CAGUCUCUGCCUGUGUCUUCACGUAGCCUUUUUCAAUGUCCCUCUGUGUCUAUAUCCAAAUCUCCGUCUCUUUCAUGAAGACACCAUUUUAGCUUUGCGCAAUGGCAAUAUUUUAGCCAGGGCUGUUCAUCUGAGGCAUGAUUAUUGCUAAUUGAAAACUUUUCACAAUACCCUGCCAUGAUGACUGGAAAUACAGUCAG*

*>ENSG00000206752:115984567:115985067:X:-1*

*UGGCACAUGUAUACAUAUGUAACUAACCUGCAUGUUGUGCACAUGUAUCCUAAAACUUAAAGUAUAACAAUAAAAAAAAAAGAAAAUGUUUGUGUUGGCCUGGGCAGCACAUAUACUAAAAUCGGAACGAUACAGAGAAGAUUAGCAUGGCCCCUGCACAAGGAUGACAUGCAAAUUCGUGACACGUUCCAUAUUUUUCA*

*>ENSG00000253032:55421413:55421613:5:1*

*UCUACUUUCAAUGAUUUAUACUUUAAUUUUACUUAAAGUCAUUUAACCAUCUCCAACUUCUGAGACCUAUGUAGAACUCCCCAUUUUUUCCCUGAGUUAAAAGAAUUUCAGGUAUUAAUAGAGAAGAUUAGCGUGGCCCCUGCGCAGGGAUGACACACAAAUUUGUGAAGUAUUCCAUAUUUUAUAAAAUAAUUUUUAAA*

*>ENSG00000207309:97097110:97097610:6:-1*

*UUUGUUAAGCGAUUAAUGGAGGGUGUGUUCCAGCAAAAUACGGGAAUAAAUCAAGUAAGAGGAAAGCAUAAGCUUUGCACAGUGGGAGUAUGGUAGCCAGUGAGGUUUAUUCCAGGUGCAAUUAUUGCUAAUUGAUAACGUUUCCCAAUAUCCCACCAUGACAACUUGAAACAUAGUCGGUAUUGGCAAUUUUUGACAGU*

*>ENSG00000200303:49369234:49369434:12:1*

*UGUAUGUUCCAGGAGAGAGAUAAGUGGAUGAUGCAGGAAAGAAAGAGGGGACAGUUGAUAUCAUGAUUAUUUGAUCUAAAUAGAAAGUUGGGUGCUUGUUUUGGCAGCACAUAUACUAAAAUUGGAAUGAUAUAGAGAUUAGCAUGGCCCCUGCACAAGGAUGACAUGCAAAUUUGUGAAGCAUUUCAUAUUUUGAAAAA*

*>ENSG00000238478:20943916:20944116:15:1*

*CUCCCUUGUCCUCAUCACCAAAGAUAACCAUUAUUCUGAUUCCAUUCCCAUCUGUUAGUUUUGUCUGUUUUUUAAGCUUUACAGAAAUGGAGUGCUCGCUUCGGCAGCAAAUAUACUAAAAUUGGAACAAUACAGAGAACAUUAGCGUGGUCCCUGCACAAGGAUGACAUGCAAAUUGGUGAGGCUUUCCAUCUUUUUAU*

*>ENSG00000212546:80191841:80192041:X:1*

*CAUUCCCUCAAGCAUUUAUUCUUAGUGUUACAAAUAAUCCAUUUACACUCUCUAUUUUAAAAUGUACAAUUAUGUUGUUAUUGACUAUAUUGUGCUUGCUUCGGCAGCACGUAUACUAACAUUGGAAUGCUACAGAGAAAAUUAGUGUGGUCCCUAUGCAAGGAUUACAUGCAACUUCAUGAAGUGUUGAAUAUUAGUAA*

*>ENSG00000207104:54369887:54370087:X:1*

*AAGAUCGCACCACUGCACUCCAGCCUGAGCAACAGAGCAAGACUCCAUCUCAAAAAACAAAAACAAACAAACAAACAAAAAACACAGGCUCGUGCUUGUUUUGGCAGCAUAUAUACUAAAAUUGGAAUGAUACAGAGAAGAUUAGCACGGCCCCUGCACAAGGAUGACAUGCAAAUUCGUGAAGCGUUCCAUAUUUUUAA*

*>tRNA1_GluCUC:18:43299691:43299891:+*

*UGAUUUUUUCUUUUAAUCUUGAAGAAACAAAAGGCGCAAGCCUGGGAGGGAGGAUGAUGCCCCGGGUGGUGUAGUGGAUGGGAUUUGGCGCUCUCACCACCAUGGCCCGGAUUUGAUUCCCGGUCAGGGAAGCCUUUCUUCUUGCCAGGCGUGGUGGCUCAUGCCUGUAAUCCCAGCACUUUGGGAGGCUGAGGCAGAUG*

*>tRNA10_IleTAT:19:39902741:39902941:-*

*AAAUGUUUAAUUUGCAUAUAUAACACAAGGCGGGGCCCUCCGGUGAGGUCACGUGGCUAUGAACCACUGAAGAAGACCCCGGCGCAGGGGUGCGGCCGGAGCUCCAGUGGCGCAAUCGGUUAGCGCGCGGUACUUAUAUGACAGUGCGAGCGGAGCAAUGCCGAGGUUGUGAGUUCGAUCCUCACCUGGAGCACUUUUUU*

*>tRNA19_GlyGCC:2:157257598:157257798:-*

*UGCUUUCUCUGUUUUUGAAUACACAAAUACGUGAUUGUUUAGAGAAUUACCAGGGUGUAAGUGUCUUCUAGUAAGCAAUAGAAAAGCACAUUUGCUAUAAGCAUUGGUGGUUCAGUGGUAGAAUUCUCGCCUGCCACGCGGGAGGCCCGGGUUCGAUUCCCGGCCAAUGCAAGUUUUGACUUCUAGUUUUUUUCUGUGGG*

*>tRNA3_UrpCCA:9:115616914:115617114:+*

*UGAAUGUAAUGUAACAGUGGCAGCAGCAGAAAUCCUUUGCUGCCUUUGGGUCUGGGGCAGCAGCUGCGUUGGUGGGCAGAGGAGGGUGCAGUUGGCAGCCUGUCCAAGUCCAGCACGGUUGGAGCACAGGAUUUAGAAUGGGAUGGUCCUGGGUUCAAACCCCAGCUGCGCCCUAGCUUGCACUGAUCCGCUCCUCAGCC*

*>tRNA7_LeuCAG:6:26521369:26521569:+*

*AUGGGGAACUAAUAGCGUUUAGACAAGAGACAGAAGUGAGUCCUGAGCACAAUGUAGCAUAAGCGCGUCAGGAUGGCCGAGCGGUCUAAGGCGCUGCGUUCAGGUCGCAGUCUCCCCUGGAGGCGUGGGUUCGAAUCCCACUCCUGACAGUUCGUACUUUUAGUAUUUAGACACAAAUCGAGUUUAAGUUUACGCUACGU*

*>tRNA121_GlnCTG:1:144839357:144839557:-*

*GUGUUGCUUGAGGGAUCAGAAGAAAUGCCUUAACAUAGGUGUGUGGGAACUAUGGCUGCUAAGUAUGAACUCAGUUACCUCCAGUUAUAAGCUAGUGUGAGGUUCCAUGGUGUAAUGGUGAGCACUUUGGACUCUGAAUACAGUGAUCAGAGUUCAAGUCUCACUGGGACCUUUCUGUAUAAUUCCAGUGAGGUUCCUCU*

*>tRNA6_LysCTT:19:52425329:52425529:-*

*UUCUCACAGCAUGAUGCUGGCUCUGAGACAGCCAAGAUGCGGCCACAGGGCCUUUUCUGCCUUAGCCUCAGAAGUUAUGUUGUGUCACCUCUGUUGCAUUCUGCAGCUAGCUCAGUCGGUAGAGCAUGAGACUCUUAAUCUCAGGGUCAUGGGUUCGUGCCCCAUGUUGGGUGCCAGAUGAAGGAGGCCUGCCCCUCCAC*

*>tRNA18_GlyGCC:16:70822539:70822739:+*

*AUUGACAGACACAGCCCCUUGCCAAAAGCCCUGCAGAUGCUCUACCAAACAGCUGGAGCAUUGGUGGUUCAGUGGUAGAAUUCUCGCCUGCCAUGCGGGCGGCCGGGCUUCGAUUCCUGGCCAAUGCAAUGAAGUGUUGCUUUUUCUGCCUUUUGCUGGCCAGUUUUCCUCACAGAGAGAUCCUGCUAGAGGAGCCAGUC*

*>tRNA16_HisGUG:1:146544715:146544915:+*

*CUAUUGACGUCAAAACUGCUAGAGCAAGGUCAAGCAUCACCGCUUGCCUUGUGGCCCGCCGUGAUCGUAUAGUGGUUAGUACUCUGCGUUGUGGCCGCAGCAACCUCGGUUCGAAUCCGAGUCACGGCAAAGUUGCUCGUCUGGGUAGUCAGCUGAUCCGUUUUGCUCCCCGCAACCAGGCGCGUCAGGGUAAGGCAGAA*

*>tRNA8_CysGCA:14:73429603:73429803:+*

*GAACUCUAGUGUGGGUGGAACUUGAUUGUAUUGAGUCACCAUAAGUGGACAAGGAAGGAGUUCCUGAUAGGUGAAGGGGGUAUAGCUCAGGGGUAGAGCAUUUGACUGCAGAUCAAGAGGUCCCCGGUUCAAAUCCGGGUGCCCCCUUCCCGGAUGUUUUCUCAUUUAAAAGUAAAUACUGUCAGCCACAGCGUCACCAG*

*>tRNA79_GlnUUG:6:37287939:37288139:+*

*AGCCAGUAAGGGGUUCAGAGAGGAAGCAGAUAUCUUCCUGUCAAGGUUAGGGACUGUGCAGAGUAGUACAGUGGUUAAACCAUGGUCUUUGGAGCCAGACUGCCUGGGGUCGGAUCCCAGCUCUCACACUUUCCUAAACAUGCAGCUGUAAGUAAGUAACUUGUUUCUCUGUGCCUCCGUUUUCUUAUCUAUAAAUGGGG*

*>tRNA38_IleAAU:6:27241695:27241895:+*

*AAUGGGGAGGGGGAGUGGUGUGGGGGAAGAGACCCAAAAUGUUGGCUGGUUAGUUCAGUUGGUUAGAGCGUGGUGCUAAUAACGCCAAGGUCGUGGGUUCGAUCCCCAUAUCGGCCACAUAUUUUCCUCAGUACUUUUUCUCGGUAGUUCUAGAGAAACUCGUCGGUUUUCAGCCACCAAAGAGCUAAGGAUAAUUAACA*

*>tRNA8_PseudoAGG:2:87339512:87339712:+*

*AAAAUCUAAGCUGGCUUUAAAAUAGAAACUGAAAAGCCAUUAAGAAGUUCUUGUGAGUGUGGCUGGUUGGUCUAGGGCUAUGAUUCUCACUUAGGGUGCAAAAGGUCCUGGGUUCAAAUCCCAGAGGAGCCCAACUUUUAGUUCAAAAGUUCUUGUGAAAACAAAAAUAAUCUAGCCAAAAAUAUUCUUGAAAGAGGAAG*

*>tRNA2_LysCUU:11:51359830:51360030:+*

*AGCUGAAGGGAGAGGCUAAUGAGAUAACGAGGGCAGUGUGGGAGUGGAAGGCUGAGGAAAAGGUAGAGAAACCGAAUAGCUUAGUUGAUGAAGCGUGAGACUCUUAAUCUCAGGGUAGUGGGUUCAAGCCCCACAUUGGACACCAGAUAUCAGGGGAAACCAGGCCCUGAUAUUCACGUGGGUCCUUUUCUAUUUUCCCU*

*>tRNA95_AlaAGC:6:58141804:58142004:-*

*GCUCCGCCCUCAAUCUUGUCCUGCCAGGGGUUUGGCGACACAAAGCACCAGCAUGCUCCUAAUAACCAGGAAAAUGGGUGGGGUGCAGCAUUUCUCCCUCGGGGAAUUAGCUCAAGCGGUAGAGCGCUCCCUUAGCAUGCGAGAGGUAGCGGGAUCGACGCCCCCAUUCUCUAGUUUCUUGUCUGGUUUAUGUCUUUUAG*

*>tRNA5_GluTTC:13:41634807:41635007:-*

*CAGCUUCAGUAGCGCAGAGGCGGCGGUGGCGAGAGGUGCGGCGAAGGAGGCAGAGGCACUUAUGCUUGUCAGGUGGGUCACGGCAGUUUCUCAUAGCACUUCCCAUAUGGUCUAGCGGUUAGGAUUCCUGGUUUUCACCCAGGUGGCCCGGGUUCGACUCCCGGUAUGGGAACGCUUCCUUAUUUUUCUUUUUUUUUGCA*

*>tRNA2_GlyGCC:21:18827039:18827239:-*

*UUAUCAUCUUCCUUCUCCAUUAUCUAGUCUCUUGCAACAAAGAACACCUUCCAGUCGCGUUCCAAUCAUGUUACUGCUUUUCCCCAACUCUUCUAUCGGUGCAUGGGUGGUUCAGUGGUAGAAUUCUCGCCUGCCACGCGGGAGGCCCGGGUUCGAUUCCCGGCCCAUGCAGCACGAAAAUGUGUUUUGGACCGUGCGCG*

*>tRNA12_PseudoTTT:14:74055456:74055656:-*

*GGGGGCACAGGGAGUAUAUGGGUAAUCACUACCUUCUGCUCAAUUUUGCUAUGAACCUGAAACUGCUUUAAAAUAUAAAACGUAAAAAAAAAAGUAAACAACCCAGAUAGCUCAGUUGAUAGAGCAUCAGACUUUUAAUCUGAGGGUCCAGGGUUCAUGUCCCUGUUCCUUAAUUUUUUUUUAACUUUUUAAUUAAAUUA*

*>tRNA6_PseudoTTG:13:36639674:36639874:-*

*GGCUAUUUAGAUUUCUGGACAAUUUUAAAACAAUGUGCCAAAUUAGCCAGAUGAGUCUUUCCUUUGCUUAGUCACCUUUAGUCUCUAGCUGACCUUAUUUUAGGAUGUGGUGUGACAGGUAGCAUGGAGAAUUUUGGAUUCUCAGGGUUAGGUUCAAUUCCUAUAGUUCUAGAGAUAAGAGGGUUUAAACCUCUACUAUU*

*>tRNA18_ValCAC:5:180529186:180529386:-*

*AAGCUCAAAGGGACCCACGGAAGAAGCUCAGAACUCUUAUGAGAAAUAAAAUACCGCCGUGUGUCGUGACUAAUUAUGAUGGCAGAAAAGCAUCAAGCCGGUUUCCGUAGUGUAGUGGUUAUCACGUUCGCCUCACACGCGAAAGGUCCCCGGUUCGAAACCGGGCGGAAACAGUUCCUUCUACUUUUCACUUAACUGCU*

*>tRNA1_PseudoGGG:10:22852546:22852746:+*

*AAGGAGAGACAUGAACUCACAAACAUUUACAAGGCAGUGUGGCAUAGUGGUUAGAAAUGUGCGCUCUGGGGCUGCUGAUCCCAGGCUCAAACCCUGGCGCUGUCACUUCCUAACUGUAGGACCCUGGAUGAAGUUACUAAACCUGUCAGUUAAGCAACUUCAUCCACUCAAAGUAAGUAACGACAGCACCAAUCUCAUCU*

*>tRNA7_CysGCA:3:131947885:131948085:-*

*UUUAAAUUCGAAAGGAAUGUUAGACACCAAAGGUAUUUAUGAGGUGUUUUGCAGGUCAUAAAAAUGGAAUAAAUGGUUUUUUGAAGGUUAUUGGCAUCAAGGGGGUGUAGCUCAGUGGUAGAGCAUUUGACUGCAGAUCAAGAGGUCCCUGGUUCAAAUCCAGGUGCCCCCUAUGACAUACUUUUGCUCCUCUUGGCAGC*

*>tRNA19_AlaAGC:6:26682663:26682863:+*

*CCCCUGUGUUCCAGGCCACCCACUAAGGAUCAAUAAAGCUCCCCUGAUGUGGGGGAAUUAGCUCAAGUGGUAGAGCGCUUGCUUAGCAUGCAAGAGGUAGUGGGAUCGAUGCCCACAUUCUCCAAACUUUAUUAUUUAGACCCUGGGUCUCCAAACACUCAGGUCUCCAAACCCAAGUCAGAGAACAGGAUGCUGCUUUG*

*>tRNA16_UyrGUA:6:26577280:26577480:+*

*GGAUUCUAGAGAACGCAGAACUACGUGUCUAAAAAGGACAGCGUUCCGUGUCCUUCGAUAGCUCAGUUGGUAGAGCGGAGGACUGUAGGCUCAUUAAGCAAGGUAUCCUUAGGUCGCUGGUUCGAAUCCGGCUCGGAGGACGGUAGUUUUGACCUACCAAGCUAAUAAUUCCAGACAAUACGGUCUUUACAAAAAAUAAG*

*>tRNA36_ArgACG:6:27182895:27183095:+*

*AUGAUAGAUAAUCGACAGACGCAGACUGAGUGGCCAGUAGUCAAAAGCGCAACCGAGGGCCAGUGGCGCAAUGGAUAACGCGUCUGACUACGGAUCAGAAGAUUCUAGGUUCGACUCCUGGCUGGCUCGGGUGUUAAUCUUGGCUUUUUUUUUUUUUUUUUUUAAGAAAAAUAUCCUGUGUGUUUUCAAACUGAUGACAG*

*>tRNA5_PseudoCTG:10:20036541:20036741:-*

*AGAGAAAUACUAAGUUGAGCUAAGGGAUUAAUUAGUACGGGAAGGGUGUAACCUACAUUUGUGAGGUAUGGGCCCGAUAGCUUAUUUAGCUGACCUUGCUCUAGGACGUGGUGUAAUAGGUAGCACAGAGAAUUCUGGAUUCUCAGGGGUAGGUUCAAUUCCUAUAGAACCUAGGAAUUAUUUCAAAUCCUAGAAAUAAG*

*>tRNA1_PseudoCUA:3:13833819:13834019:+*

*ACCCAGCAGCCCACCUGGCUCAUUGCUAGUGCUCAGUAAAUGGCUGUUACUGACGAUGCCGUGGAGGGCAGGGUAGGGUAGAGGUUAAAACCAUGGAUUCUAGAGCCAGAUGGGUUCAAAUCCCGGCUCUGCCGCAUCCCGGCUGUGUGACCCUGGGCAAGUUGCUCAACCUCUGUGUGCCUCCCCCGUGGGAGUUACCA*

*>tRNA1_PseudoUUG:X:55206599:55206799:+*

*UAUGGGAAGGGUGUAACCAACAUUUUCGGAGUAUGGGCCCGAUAGCUUAUUUAGCUGACCUUACUCUAGGACAUGGUGUGAUAGGUAGCAUGGAGAAUUUUGGAUUCUCAGGGGUAGGUUCAAUUCCUACAGUUCUAGAAAUAAGAGGAUUUUAACCUCUGUUGUUUACUCUAUCAAAGUAGUUCUUUUGUCAGACAUAU*

*>tRNA52_ProCGG:1:167683894:167684094:+*

*CUCGGAACACGAGGUGAGAGGCGGAACAAGGCUGAAGGGAAGCUCUGGGAGUGCCCUUGCCGAGAGCGGCUCGUUGGUCUAGGGGUAUGAUUCUCGCUUCGGGUGCGAGAGGUCCCGGGUUCAAAUCCCGGACGAGCCCAGGCUUUUACCCACCCCACACCCGAAAUUUAAAAACAGAUUUUUUCCCCCUAAUUCCCCAC*

*>tRNA2_ValCAC:5:180524021:180524221:+*

*ACCGCCGUGUGUCGUGACUAAUUAUGAUGGCAGAAAAGCAUCAAGCCGGUUUCCGUAGUGUAGUGGUUAUCACGUUCGCCUCACACGCGAAAGGUCCCCGGUUCGAAACCGGGCGGAAACAGUUCCUUCUACUUUUCACUUAACUGCUUCAAAUUUAUUACACCGAAGACUAGCCUAGAACAUCCCACCAAUAUCUCCAC*

*>tRNA5_CysGCA:7:149007236:149007436:+*

*AUUCCAAAGCUGAAAUAAUAUUAAUAGGAAACAUAAUGGCAGCAGGGGGCAUAGCUCAGUGGUAGAGCAUUUGACUGCAGAUCAAGAGGUCCCUGGUUCAAAUCCAGGUGCCCCCUAGUUUUCUUCACUUCUAGAAUGCAUCUUUUGGGAGACAGUAUUUGAGACAAAAAAGGAGAUGAAGGAGAAACAAUGAAAGAAGC*

*>tRNA1_GlnCUG:6:18836337:18836537:+*

*UCUUAUUAGUCAUCUGAAUCGCUUUCUGCUGCCUGAAUUUCUAGAAUAAAACAGCAGGGAGGAGGGUUCCAUGGUGUAAUGGUUAGCACUCUGGACUCUGAAUCCAGCGAUCCGAGUUCAAAUCUCGGUGGAACCUUUCAUAUUGGCUGGGUGCUGUGGGAGGCCAAGGUGGGCAGAUCACGUGAGGCCAUGAGUUGAAA*

*>tRNA37_ValAAC:6:27203243:27203443:+*

*UCAUUUUAACUAAUUCAAAACUGAAAAACACUCUCACUAAGCGAGUUUCCGUAGUGUAGUGGUUAUCACGUUUGCCUAACACGCGAAAGGUCCCCGGUUCGAAACCGGGCAGAAACAGAGCGUAGUUUCGUUUUUUUGGUUGUUUUUUUUUUUUUUUUUUUUUUUUUUUUUUUCUUCUCUUGAGAAGGAGUCUCACUCUG*

*>tRNA57_IleAAU:6:27636296:27636496:+*

*UUAGAAAAACAAAAAUAUCAACGAAUUUAUAUAUGGUUUCCUUUAAAACAGCAGCGCAUUCGUGUGGCCGGUUAGCUCAGUCGGCUAGAGCGUGGUGCUAAUAACGCCAAGGUCGCGGGUUCGAUCCCCGUACGGGCCACUGGAUGCCAUAGUGGGGCCCUUCACUUACCUGAAGGGAGGCCUUUUAAUUUAAGCCUGUC*

*>tRNA17_SupUUA:17:58863520:58863720:+*

*GUAAUUUCAGGGUAUUAAUAUUGUUUACUUGGACAUAUUGGUAGAAUGUGAGAAUAUAUAUAGUAUGAGGGAGCCCGGAUAGUUCAGUUGGUAGAGCAUCAGACUUAAUCAGAGGGUCCAGGGUUCAAGUCCCUGUUUGGGUGUGUCCUUCGGCUUUUAGCACCUAAUUCUGAUUCCACCUUUGAAAAAGAAAGAAAGAA*

*>tRNA9_ProAGG:16:3239556:3239756:+*

*GGUCCUGCUGGGUCCGCCCCAGCCGCCCCCAGGAUGCUAGGACGCCCACAAAGAUAACCUUAAUGCGUAAAUCGUGUGGCUCGUUGGUCUAGGGGUAUGAUUCUCGCUUAGGGUGCGAGAGGUCCCGGGUUCAAAUCCCGGACGAGCCCGGCUUUUGGUGCAGGGUAAAAGUCGUUUCCUGCUCUUUUUUAGAUUCGGCU*

*>tRNA10_PseudoCTC:3:103879457:103879657:-*

*UCUGAAAUGUGAGACUCUAAGGCCUUUUUUUUUUAUUGUGUGUGCACGUGUGUGUGUAUUUAUCUAGUAACUUUGUUAAUUCUGUUCUUUUUUUUUAUUAUUAUUAUUAUACCUGUGGUUAGGAUUCGGCGCUCUCACCGCCACGACCCGGGUUCAAUUCCCGGUCAGGGAACCAAGAAAUGGUCUGGAUGAGCCAGGGA*

*>tRNA6_GluUUC:2:75123978:75124178:+*

*AAAACAAAGUUAAUAUCCUUCAAAUGCUCACACUGUCUGCUUUGGAUUUUAUAAGCAACGCACUCUAGCCUGUGGUCUAGUGGUUAGAAUUCAGUGUUUUCAGUGCUCUAGUCCAGGUUCAAUUCCUGGUCAGGGAACCAGUUCUUUGGAAAUAAAAGUCCUUUGACUCAGUAGGGGGAAAAAAAAGCAUUUAUGAGAGU*

*>tRNA94_AlaAGC:6:58142514:58142714:-*

*AAGUUUCCGGAGGUGUUCGAAAACCUGGAAACUUAAGUGGGACAUAGGACGCUAUUUGUGUCCCGGUUAUAAAAAUUGGCAGAAAAGACAAGUGUAGUGUGGGGGAUUAGCUCAAGCGGUAGAGCGCCUGCUUAGCAUGCAAGAGGUAGCAGGAUCGAUGCCUGCAUUCUCCAGCUUCUUUUAAUCCCUAGGCUACCAAU*

*>tRNA2_PseudoCTC:13:58458476:58458676:-*

*GUUUUAUACAUGAAGUCCUUGCCCAUACCUAUGUCCUGAAUGGUAUUGCCUAGGUUUUCUUCUAGGGUUUUUAUGGUUUUAGGUCUAACGUUUACGAAGUUCCCUGGUGGUCUAAUGGUUAGGAGUCGGCACUCUCACCGCCGCGGCUGGGGUUUGAUUCCCAGUCAUGUAAACCAAAAUAAUUUUUAAAUAAAUUUAUU*

*>tRNA20_LeuCAG:5:159391975:159392175:-*

*GGGGAGUGACUCACUCAAGGUCACUCAACUGGUGAAUGGCAGAGUGGCCACCACGACCUGUUCAAGUUGCCAUUCAUUUGCUUCAGCAAAUAUUUAUUGAGGCAGUGGAGUUUAGUGGUUAAGGACCUGCUCAGACAUCACAGGUAGGUAGAUCUGGGUUCAAACCCUAGCCCUGGCAUUCUCAGCUGGGCGACCUUGGA*

*>tRNA9_PseudoTTG:3:106620806:106621006:-*

*AGAGAUAAUAAGUUGGGUUAGAGGGUUAAUUAGUACUGAAAGGAUAAAAAGCAACAUUUUCGGGGUAGGGGCCCAAUAGCUUGUUUAGCUGACCUUAGUAUAGGAUGUGGUGUAUUAGGUAGCACAGAGAAUUUUGGAUUCUCAGGGGUAGGUUCGAUUCCUAUAAUUCUACAAAUAAGAGGACUUAAACCUCUAUAAUU*

*>tRNA8_HisGTG:15:45492543:45492743:-*

*UAGAACAAACAAUGACAAAUCAAUGACAUUGCAAGGGGGCGGGGCUUUCAACAGAGAAGAGUUCAGGCGACAAGCCGAGGUCCUGUGUCCCUAGUAGCUCGCCGUGAUCGUAUAGUGGUUAGUACUCUGCGUUGUGGCCGCAGCAACCUCGGUUCGAAUCCGAGUCACGGCAUUGUGAGGACAAUGGCACGGCAAGGGGA*

*>tRNA5_PseudoAAT:20:51218260:51218460:-*

*AAUCUAAGGAUUUUUUUUAUCAGACCGAAACAAACAUGUUAAAACCAAGGAUCCUGUGUUCUGACAUCUUUUCCUUCUCUCCAAAAUUUUUGCCAUGUGUGACCAAUUAGCAAGCACAGUUGGCUAGAACAUGGUGCUAAUAAGGCCACGGUCAGGGGUUCAAUUCCCUUAUGGGCUGAAACUUGAUACUUCCUUCCUAC*

*>tRNA7_LeuAAG:5:180614628:180614828:+*

*UCCCUGUUGGCCCAGAGCCCCUUCGCACGCUUCAUAACUGAAGGAAAAUAUGUAAAUACCCAGCGGCAAAGAGGUAGCGUGGCCGAGCGGUCUAAGGCGCUGGAUUAAGGCUCCAGUCUCUUCGGGGGCGUGGGUUCGAAUCCCACCGCUGCCAGUUUGUGGUAGUUUUGUCACUUUUGUCCCCUAUGGGUUACAAAAGG*

*>tRNA13_LysCTT:14:58706553:58706753:-*

*UACGUUAUAAAUUUUUUUAGAAAAGAAACUACGCGGAGAAUAUAAAACCAUGUAGUUAAACAGUCCUCAAGAAGUAAAAAAGCAGGCUAACGUAACAGGCGCCCGGCUAGCUCAGUCGGUAGAGCAUGGGACUCUUAAUCCCAGGGUCGUGGGUUCGAGCCCCACGUUGGGCGCGGGUUGUAACUUUUUUCCUGAAACUU*

*>tRNA6_AspGTC:9:77517922:77518122:-*

*UUGCAGUGAGCCGAGAUUGCACCACUGCACUCCAGCCUGGGUGACAGAGUGAGAUUCCAUCUCAAAAAAGAAAAAAAAAAAAAAAAAGAAAAAAAGGCGGUCCUCGUUAGUAUGGUGGUGAGUAUCCCUGCCUGUCACGCGGGAGACCGGGGUUCGAUUCCCCAACGGGGAGGCUAAUAAUGUUUGGCCAGGUACGGUGG*

*>tRNA6_PseudoCTT:15:76674702:76674902:-*

*UACAGUUUCUGUUUAAAAGGGUGAAUGCUUGGCAGCCAUGUUUACUAUAACUGCCUGCUCUUGGCCUCUAGAUGCUUCACAUAUACCUUAAUUCAGAAUUGCCUGGCUACCUCAGUUGGUAGAGCAUGGGACUCUUAAUCCCAGAGUCAGUGGGUUCAAGCCUCACAUUGAGUGGUUCUACUUUUUUUUUUUAACACCUG*

*>tRNA2_LeuUAG:14:21093472:21093672:+*

*UGGGGCAAAAUGCUGACAGAAUGAAGAAGGUAAUUGCAAACUCCUAUAACGUGUGUGGUAGUGUGGCCGAGCGGUCUAAGGCGCUGGAUUUAGGCUCCAGUCUCUUCGGGGGCGUGGGUUCGAAUCCCACCACUGCCAGUGGUACGUUUUAGCACAACAAGGAUCCUGAAGUACUAACACUUUCUAACUAGUAAAAUAAU*

*>tRNA27_LeuTAG:16:22206964:22207164:-*

*CAGGAGACAGCGGUUCUAUAUUGAGCAUGCGCAAACUUUGAGGGCGGGGCGCCUCCGUCAUCACACUAAUAUGCCGGCAUAGGAAGCCCUACGAUCGGGAGGUAGCGUGGCCGAGUGGUCUAAGGCGCUGGAUUUAGGCUCCAGUCAUUUCGAUGGCGUGGGUUCGAAUCCCACCGCUGCCACACCUCAGAAGGUCUCAC*

*>tRNA9_UyrGUA:7:149255068:149255268:+*

*UUUUACAAAGCUGAGUGCUCCCACACUGAUGAAAGGUAUUAACUAGGUAACCUCAGUGUGCCAUGGGGGUAUAGCUCAGGGCUAGAGCUUUUUGACUGUAGAGCAAGAGGUCCCUGGUUCAAAUCCAGGUUCUCCCUUCUGUUAAGUUUAAUUUUGGUAGCGAACUUUAUCCACAUACAGCUUAGAUUGGCAUGGGCCAC*

*>tRNA6_UrpCCA:17:8089609:8089809:+*

*UUCAUGACCCUGGCCUCCAGGGCGCACAGUGGUCAUGGGGAGACCUGAGCUGCCGAGUGGCCGGCCGACCUCGUGGCGCAACGGUAGCGCGUCUGACUCCAGAUCAGAAGGUUGCGUGUUCAAAUCACGUCGGGGUCAGCGGCUAUUUUUCUUCGGUUUUUAUUAACCCCCUUUAUUUUAAACUACGGUCGAGCUUCAGC*

*>tRNA103_AsnGTT:1:149230511:149230711:-*

*UGCUCAGUCGUCCUGCCAGGCGGGCGCUGAGAAUAGAAGGGACGGAGGAAGUUUAGUGAGUGUGCCCUUCCUAUAUUGCCUAUUAGAACUGGUAGUGCUUGUCUCUGUGGCGCAAUGGGUUAGCGCGUUCGGCUGUUAACCGAAAGGUUGGUGGUUCGAGCCCAUCCAGGGACGCUGAUUGCAACUUUUAAAGCAUUCAC*

*>tRNA1_GluCUC:18:43299678:43299878:+*

*CCUCACUGGGAAAUGAUUUUUUCUUUUAAUCUUGAAGAAACAAAAGGCGCAAGCCUGGGAGGGAGGAUGAUGCCCCGGGUGGUGUAGUGGAUGGGAUUUGGCGCUCUCACCACCAUGGCCCGGAUUUGAUUCCCGGUCAGGGAAGCCUUUCUUCUUGCCAGGCGUGGUGGCUCAUGCCUGUAAUCCCAGCACUUUGGGAG*

*>tRNA1_SeCUCA:22:44546470:44546670:+*

*AACCUGGGAGGUGGAGGUUGCAGUGAGCUGGGAUCAUGCCAUUGCACUCUAGCCGGGGCACCAAAAGCUCGGAUGAUCCUCAGUGGUCUGGGGUGCAGGCUUCAAACCUGUAGCUGUCUAGUGACAGAGUGGUUCAAUUCCACCUUUGUAGGCCCGGCGCAGUGGCUCACGCCUAUAAUCCCAGCACUUUGGGAGGCCAA*

*>tRNA7_IleGAT:X:3756344:3756544:-*

*GAAGGCAGCCAGAGGCGCGCGGGCUUGGCGAGGAAUCGCUCGUGGACGGGCGCGAACGUGGAGCCCUUUUAUACGCUCCAGGGGCACUGUGCCGUCAGGCGGCCGGUUAGCUCAGUUGGUAAGAGCGUGGUGCUGAUAACACCAAGGUCGCGGGCUCGACUCCCGCACCGGCCACGGCGUUAGCUUUUUUUUUUUUUUUC*

*>tRNA16_TyrGTA:14:21131299:21131499:-*

*CGGGUUCCAAUCAACUCUAAGUGUGUUGACUCCAGCGUUCCAAGGACUUGGCUUCCUCCAUUUGCGGAAAGUCCAGUGAUCCAGCUCUUGCAGCGUGCACCCUUCGAUAGCUCAGCUGGUAGAGCGGAGGACUGUAGAUUGUACAGACAUUUGCGGACAUCCUUAGGUCGCUGGUUCGAUUCCGGCUCGAAGGAAGUGCC*

*>tRNA25_GlyGCC:16:70812040:70812240:-*

*CCACCUUUCUGCUUAUGUGGCAAGAAGUCAAGCCCGCUAGAAAGGAACCACUCCAUCCUGUGGGUCGUGGCCUCAACUAAAAACAUCCCUACCAGCUGGAGCAUUGGUGGUUCAGUGGUAGAAUUCUCGCCUGCCACGCGGGAGGCCCGGGUUUGAUUCCCGGCCAGUGCAGCCUAUCUACCUUUUUAGAUGGUUUUCCU*

*>tRNA6_ProCGG:16:3221988:3222188:+*

*CCGAUUUGUGCCGGAUUGGUGGCAGGUGUCUGAAAUGUCAGCGGAAAUACACGCACGGGAGGCUCGUUGGUCUAGGGGUAUGAUUCUCGCUUCGGGUGCGAGAGGUCCCGGGUUCAAAUCCCGGACGAGCCCUAGAAGUGGUUACUUUUCCCUUGUCAUUUUAGAGAAUAUAGAGCUAGAAAAUCGGGGACCGAGCCUGA*

*>tRNA30_CysGCA:17:36989908:36990108:-*

*UGGGGCUUUAGAACACAAAGCUUGCCAGAACUCGUGUGGGUGGAACUUGAUUGUAUUGAGUCACCGUAAGUGGACACGGAAGAAGCUUAUUACAGGUAGAGGGGGUAGGGCUCAGGGAUAGAGCAUUUGACUGCAGAUCAAGAGGUCCCCGGUUCGAAUCUAGGUGCCCCCUUCUGUGGUGUUUUCUCACUUCCACUCCA*

*>tRNA16_UyrGUA:6:26577258:26577458:+*

*UCCAGGACGCACAUUAUUUUGGGGAUUCUAGAGAACGCAGAACUACGUGUCUAAAAAGGACAGCGUUCCGUGUCCUUCGAUAGCUCAGUUGGUAGAGCGGAGGACUGUAGGCUCAUUAAGCAAGGUAUCCUUAGGUCGCUGGUUCGAAUCCGGCUCGGAGGACGGUAGUUUUGACCUACCAAGCUAAUAAUUCCAGACAA*

*>tRNA5_GluUUC:1:17199012:17199212:+*

*CUCCCAGAGCUCAGGGAAGCUGUCGCUUCUGACAGAAGAAGGGAGACAAAGCUCCCUCCUGUGUGUCCCUGGUGGUCUAGUGGCUAGGAUUCGGCGCUUUCACCGCCGCGGCCCGGGUUCGAUUCCCGGCCAGGGAAUUGUUUUACACUGGCCGCCCUCCCGCAGGAAUCUUCCUUCACUACGCUGUCAGCCGGCCUGCU*

*>tRNA1_PseudoCUA:3:13833817:13834017:+*

*UCACCCAGCAGCCCACCUGGCUCAUUGCUAGUGCUCAGUAAAUGGCUGUUACUGACGAUGCCGUGGAGGGCAGGGUAGGGUAGAGGUUAAAACCAUGGAUUCUAGAGCCAGAUGGGUUCAAAUCCCGGCUCUGCCGCAUCCCGGCUGUGUGACCCUGGGCAAGUUGCUCAACCUCUGUGUGCCUCCCCCGUGGGAGUUAC*

*>tRNA9_PseudoTTT:19:41748085:41748285:-*

*GGAGGUUUCAGUGAGCCGAGACUGGGCCACUGCACUCCAUCCUGGGUGACAGAGCAAGACUCCAUCUCAAAAAAAAAAAAAAAAAAAAAAAAAAGAGAGUGCCAGGAUAGUUCAGGUGGUAGAGCAUCAGACUUUUAACCUGAGGGUUCAGGGUUCAAGUCUCUGUUUGGGCGUUUGCAGUUUUCCUUACUUUAAGCUAC*

*>tRNA18_CysGCA:7:149310078:149310278:-*

*GGGGGUAUAUCGUGACGAAGCAAGUACAUAUCUCAUAAUGGUGACAGUCAGGGUGUGGCCAUACAGGUUUAUAGUUCUUAUUGGAGACAGGCAAUUACAUGGGGGUAUAGCUCAGGGGUAGAGCAUUUGACUGCAAAUCAAGAGGUCCCUGAUUCAAAUCCAGGUGCCCCCUUCCUGUUUUCUUUUGUUGGGGUGAUCAG*

*>tRNA15_PseudoTTG:12:97490631:97490831:-*

*AAUAAAUUGAAUAAUUUCAAAUGUACCAGUAAAGCUUACUGCUUAAAAUAAUACUAAUAUUUUUAUGAAUUAAAUCAAAAACCCCUUAGUUACUGUGAGAGGCAAUGUAGCAUUGUGGCUAAGUGCACAGGCUUUGGAAACUGGCAGGCCUGGGUUCAAAUCCCAGCUUAUUCACUAUUUGACCUUGGUCAAGUCACUUA*

*>tRNA1_LysUUU:12:27843254:27843454:+*

*GAGGGACAACUGAGUAUAUAUCUUUUAAAAGUAUAAUAAUUAAGAGAAUGCACCCAGAUAGCUCAGUCAGUAGAGCAUCAGACUUUUAAUCUGAGGGUCCAAGGUUCAUGUCCCUUUUUGGGUGUUCCCUCUUAGGUGGGCAUGGUGGCUCAUGCCUGUAGUCCCAGUGCAUUGGGAGGCCAAGGCAGGAGGAUCACCUG*

*>tRNA13_PseudoTAA:8:47739975:47740175:-*

*GUAAGAGAAAUAGGGCCAACUUCAUAAAGCACCCUCACUGCAUAGAUGAUGUUAUCUCAGUCUAACAACUCACCACACACCCUGCUCAAAAACAGGGUUUGUUAAGAUGGCAUAGCCCAGCAAUUGCAUAAAACUUAAGACUUUAUAAUUAGAAGUUCAACACCUCUUCUUAACAAUAUGCCUAUAAUUAAACUUCUACU*

*>tRNA22_GlnCUG:1:147800870:147801070:+*

*AUGGGUGGGUGGGAACUAUUGAUACUUAUUAGGAAUCCACACACCUGCAGAAAACUUGCAUUGUGAGGUUCCAUGGUGUAAUGGUAAGCACUCUGGACUCUGAAUCCAGCCAUCUGAGUUCGAGUCUCUGUGGAACCUUUCUGUGUAACUGGGACGAUAAUGCCGUCUUUUACUCCCUAAAUGGAAUGGGGAUUCCGCUG*

*>tRNA14_ProTGG:5:180615793:180615993:-*

*UGAGAAUGGGUUCAAGCUGCCAGACGGAAAUCCGCCUUUUCGGUGAAUCGAGCCAUGAAGAACACAAUCAUGAAAAGAUUCAGCGAGCAACUAGACAGCCGGCUCGUUGGUCUAGGGGUAUGAUUCUCGCUUUGGGUGCGAGAGGUCCCGGGUUCAAAUCCCGGACGAGCCCACUUUUUCAUUUUUUUUUUUCCAUUUCA*

*>tRNA3_GlnCUG:12:74851117:74851317:+*

*CUAAUUAAAAGUCUGAGGAAGGACUUGCACACCUCCAGUGAGUCAGAGCAGCCACCUCUCCGGUGGUUCCAUGGUGUAAUGGUAAGCACCCUGGACUCUGAAUCCAGCAACCAGAGUUCCAGUCUCAGCGUGGACCUUUACCUGAAGUCCAAAUAAAAUCUCUUGCUUUGGAAGCCCAAAAAGGCAAACUUUGGGGUGGA*

*>tRNA20_AsnAUU:1:147718970:147719170:+*

*AUUGGGGAGUUCGGUGAGUGCGCCCUGCCUAUAGCGCCCAGUGGAAUCGCUAGUACCUGUCUCUGUGGCGCAAUCGGUCAGAGCGUUCGGCUAUUAACCGAACGGUGAGUAGUUCAAGACCACCCAGGGACGCCUGUUCUAGCUUUUAAAGCAUUCAUGUAUUAUCAUCACUAGAGAAUCUCCCCCUAUUCUUCCCAUAG*

*>tRNA3_PseudoCAC:1:17186625:17186825:+*

*AACGAUAAUAGAAAUGGCUGACCUAAGAUUUUCAUGAUGAUAUUUUGCCUUUUUUGGUGUAUAUGCAGUUUCUGUGGUGUAGUGGUUAUUAUGUUCGCUUCACAUAUGAAAGGUCUCUGGUUCGAGACUGCGUGGGAACAUCGUGUUUUGUUUUGUUUUUUUGUCCCUAAAUUUAGUGAGUUUAAUCGAGGUUGGGAAAC*

*>tRNA5_LysCUU:19:36066676:36066876:+*

*GGAAUGUGAGUGCCUUAGAGGUCUUGGGGCCGAAACGAUCUCAACCUAUUCUCAAACUUUAAAUGGGCAAGAAGCCCAGCUAGCUCAGUCGGUAGAGCAUAAGACUCUUAAUCUCAGGGUUGUGGAUUCGUGCCCCAUGCUGGGUGCCAGAUGAAGAGGUGAGUUGCCCCUAAAAAGGAUACCCUAUUCCUUCUCUUUCU*

*>tRNA84_GluTTC:1:161391826:161392026:-*

*AAAAGACAUUCCUAUAGGUUUCUAACAUGUGCCUGAGGUGGUUGAGGCGUCUGUCCUAAGAUCUUUUCUCAGAAGGUGCUGCCCACAGUGUCACCAGCCCUCCCUGGUGGUCUAGUGGCUAGGAUUCGGCGCUUUCACCGCCGCGGCCCGGGUUCGAUUCCCGGUCAGGGAAGUUUGCUUUUUUCCUUUCUGAUAUCACU*

*>tRNA13_CysGCA:7:149332717:149332917:+*

*UCGUGUUCUUGGUGUGUCGACACAUGAAAAGGGUGUUAAUUACACCCUCUCCCUGGGCCUGGGGGUAUAGCUCAGGGGUAGAGCAUUUGACUGCAGAUCAAGAGGUCCCUGGUUCAAAUCCAGGUGCCCCCUACCCUGUUUCACUUUUAAUCACCAAAGUGGGUCCUGGAUCACAUGCUCCUCUCGGGCUCUCUCUAUUA*

*>tRNA63_IleUAU:6:28505305:28505505:+*

*UCAUCGUCAAUCUGUAAGAGCAUCUUCUCUUAUACCUUUCCAAAUCCUUAUUAUUUCUGUGGCUCCAGUGGCGCAAUCGGUUAGCGCGCGGUACUUAUAAGACAGUGCACCUGUGAGCAAUGCCGAGGUUGUGAGUUCAAGCCUCACCUGGAGCAGUUUUACUAUUCUCCGACCUGUAGAAAUUCAAAAACAGGGAGAGC*

*>tRNA96_PheGAA:6:28949370:28949570:-*

*ACCUGUCAUUGUGGGUUUUUUUUUUAAUAAAUACCUAAUAGUAAAUAAAAAACAUAAGUAAUAAAUUCAUUAAGCCUUAUUUUUAAGUGGCGUGGGCGGAGCCGAAAUAGCUCAGUUGGGAGAGCGUUAGACUGAAGAUCUAAAGGUCCCUGGUUCGAUCCCGGGUUUCGGCAUGAGAGCGCUCGGUUUUUUGUGCCCAC*

*>tRNA2_LeuTAA:4:156384910:156385110:-*

*AAGGACAAAAUAGGGCCCACUUCAUAAAGCACCCUCGCCUCAUAAAUGAUGCUAUCUCAAUCUAACAAACCAUCACACACCCUACCCAGGAACAGGGUUUGUUAAGAUGGCAGAGCCUGGUAAUUGCAUAAAACUUAAAAUUUUAUAAUCAGAGGUUCAACUCCUCUUCUUAACAAUAUGGCUAUAAUUAACCUUCUCCU*

*>tRNA16_ValTAC:11:59318408:59318608:-*

*UCUCUUUUCGUGAUCGCUGAGCAGUAAAUCCCUUUCAGUCCACGUUGUUAACAUGUCUUUAUGCAGAAUUGCUAAAUCCAGAGACAGCCAUUCCGCACGCGGUUCCAUAGUGUAGCGGUUAUCACGUCUGCUUUACACGCAGAAGGUCCUGGGUUCGAGCCCCAGUGGAACCACGGCGUGAUUCAUACCUUUUUCUUUUC*

*>tRNA2_MetCAU:6:26286689:26286889:+*

*GGUAGUCUGAACGCAUCUCCUGUAAGUAGUUAAGAGUACUGUGAGACCGUGUGCCUGGCAGAACAGCAGAGUGGCGCAGCGGAAGCGUGCUGGGCCCAUAACCCAGAGGUCGAUGGAUCGAAACCAUCCUCUGCUAGGUCCUUUUUUUUUCUCCCCCCCCGUCUAUUUUCCUGAGGAUCCCUUUUUUUUAAGUUACAGUU*

*>tRNA5_LysUUU:11:59323826:59324026:+*

*AAUCGCGAAGGGAAAGAAUGCGGCCACGUGGCCUAUUUUCCUGUGGAUAGACUAAGCAAACGCUUUUCUUCAGGGGCCCGGAUAGCUCAGUCGGUAGAGCAUCAGACUUUUAAUCUGAGGGUCCGGGGUUCAAGUCCCUGUUCGGGCGGAUGCUGUUUUAGUUUCCAAUAAAAUGGAUUUGGGCGAGGCUGAGAGAAAGG*

*>tRNA4_UhrAGU:19:33667896:33668096:+*

*GCGCAUGCGCGCGGCCCAUAUGGUAAAUAGUAAUGAAGAAUAGUUUAGGAAAGUACCUGUACGGGUGGCGCCGUGGCUUAGUUGGUUAAAGCGCCUGUCUAGUAAACAGGAGAUCCUGGGUUCGAAUCCCAGCGGUGCCUCAACCGAGCGUCCAAGCUCUUUCCAUUUUUGCUCCUGCACUUUUGUGCUCUGUACCCGGC*

*>tRNA9_PseudoGAA:8:124270658:124270858:-*

*UUCCUCUUCAUUCUAGUCAAUUUAAUAAACAUUGAGUGAUUACCUCCCUAAAUUCAGGCUUCUUAUAUGCACUUGGGAAUGCAACAUUAAUGAGAUGUCAGCCAAAAUAGCUCAGCUGGGAGAGUAUUAGGUUGAAGAUACAAAGUUCCUUGGCUCAAUCCAGAGUUUGGGGGACUUUGUUUUAUCUCAAAAAAGCUGUG*

*>tRNA2_ArgCCU:16:3202823:3203023:+*

*GACGGGCAGGCUGUGCAGGAACCACCGCGGAGUGAUGGAGAAACUGGUCUAAGACAAGCGACAGCGUUUUGUUAUCCGCCCCGGUGGCCUAAUGGAUAAGGCAUUGGCCUCCUAAGCCAGGGAUUGUGGGUUCGAGUCCCACCCGGGGUAAAGAAAGGCCGAAUUUUAGUGUUCCUUAUCGGGCAGAAGAGUUAGAAUGC*

*>tRNA115_AsnGTT:1:145978958:145979158:-*

*UGCUCAGUCAUCCUGCCGGACAAGCCCUGAGGCUGGAAGGCACAGGGGAAAUUUGUUGAAGGCGCCCUUCCCAUAGCGCACAGUAAAAGCAGUAGUUCUUGUCUCUGUGGCGCAAUCGGUUAGCGCGUUCGGCUGUUAACUGAAAGGUUAGUGGUUCGAGCCCACCCGGGGACGCUUGCUCUAGCUUUUAAAGCGUUCGU*

*>hg19_ct_UserTrack_3545_(null) range=chr5:31317364-31317563 5'pad=0 3'pad=0 strand=+ repeatMasking=none*

*GGCUAUCAUUUUUAUAAUUAAAUUUUUAAAUCAUUUUGUCAAGCAAUUUAUUGCAAAACGGUAAGAAACACUUUUGAGUUAUCAAAAUUUUAUGGCAGCGUUUUGGUUUUUCUCUUAGUUGAUUCAGACCCUGCAUGCUGUUGACAAGGAUGACCCUUAUAGUGGACACCAAUUUUCGUUUUCCUUGGCCCCUGAAGCAG*

*>hg19_ct_UserTrack_3545_(null) range=chr2:143790760-143790959 5'pad=0 3'pad=0 strand=+ repeatMasking=none*

*UUUAAAGUUACCUUCUAUGAUAAUGACAUGAUAUUAAUUUCUCAGAACUGCAGUUAAUCCCUGGGGUCUGUGGAUUCCGAAUUUCAAAUCCUCCCAUUUUGUUGGUCUGUUCCUUGCAUGCUAGUUUAGAGGUAAGUGAUGUGUGUUUCAACCUUCCCACAUCUUUGCGUUUUUUCUUGAUCUGUUUGUGACAAUUCAUA*

*>hg19_ct_UserTrack_3545_(null) range=chr1:97915625-97915824 5'pad=0 3'pad=0 strand=+ repeatMasking=none*

*AAGUCAGCCUUUAGUUCAGUGACACUUUGACACCAAUAUGCAGCCGUUUUCUCACUGAUGAGCUCAAUAUUCAGAAAGGAGCUUUGUCCAGGGCCAUACAUGGGGCCAGAGGUGGUUCCCCGGAUGAUUCUGGGGGAAACAUUUGUCACAAUGUCCUGAUGAAAGAGUAAAGAUAUUGAGUCUCCUUUUGACAAAGAAAA*

*>hg19_ct_UserTrack_3545_(null) range=chr18:909341-909540 5'pad=0 3'pad=0 strand=+ repeatMasking=none*

*GCCCUCCCCGAAGGCUCCCGCGUGGGGUGGGGCCCGCCUGCUCCCCGCGGCGAUUGAACCUGUGUCUCCCGCCCCGCCACCCUCUUCCCGACCCCUUUGCUUGCAGUGGGAGCCUCGGCGGCGGCGCGGGGGACGACGCGGAGCCGCUCUCCAAGCGCCACUCGGACGGGAUCUUCACGGACAGCUACAGCCGCUACCGG*

*>hg19_ct_UserTrack_3545_(null) range=chr4:46930519-46930718 5'pad=0 3'pad=0 strand=+ repeatMasking=none*

*UAUCCAGUUCGGAUAGAAGUAGGAGAAGCAGAUGGAAGUGCUCUUGCUGCAGAUAUGGUUUCAGCUGCAUUUGCACGGCUGAAUGGGUUUGGACUGGAAGCUAAGUAAGACCGAGGUGUGCCUUUAGAAGAUUCUUGAACAACUGUGGAAGAUUUGCUUGAAUGGUUUCCCACCUCAGUUCUGUUGCCAACAUCAGAUUC*

*>hg19_ct_UserTrack_3545_(null) range=chr15:90347471-90347670 5'pad=0 3'pad=0 strand=+ repeatMasking=none*

*GAUGGGGGUCUACCUGGUGGGCCAGCUCAUGAGCAAUCACAGUGACCACCCGCUCCUUGUUGCUGCUGGAGGAGGACAGGGGGUCGAACAGCAGGGAGUUCUCCCGGUAGGUCACCAGUCCCCAGUUCUCCAUGGCGCCGGCGUUGAAGUCUGGCAGGCCAAUCUGGUCUGGGGAGGCGAUGCCAUUGGCAGGAUGAACU*

*>hg19_ct_UserTrack_3545_(null) range=chr3:169990928-169991127 5'pad=0 3'pad=0 strand=+ repeatMasking=none*

*AUAAAACAAAAUUAAAAGUAAAUUCUCACUUAAGUUAUAUUCUUUCAAAAUUAAAUUUUAAUAUGCUGUGUGUUGUUGAAAUAGUGUUAUUUCUGGUGUAAUAGAGGUACUCCACUGAGUUACUGUGUCUUUGGAAAUGUUUGUAGGAACCAGUGAUGCCCAUGGAUCAGUCAUCCAUGCAUUCUGACCAUGCACAGACA*

*>hg19_ct_UserTrack_3545_(null) range=chr5:150924312-150924511 5'pad=0 3'pad=0 strand=+ repeatMasking=none*

*AUAUGUCCCCAAGAUAGGGGUCAAUUCGGAAAUAUGUGUAAUCUUCUGCAAAUUCAUAUGUAACAGCCCCAUUUGUCCCCAAGUCCUCAUCAGUGGCAGAUACCUGAAAGAGGACAUCCCCUGGCUCUGUGCCAUCUUGGAUGAUUGUGUAAUAGGGCAGAUGCUUAAAUUUGGGGGGAUUGUCAUUGACAUCCUCAAUA*

*>hg19_ct_UserTrack_3545_(null) range=chr22:50639701-50639900 5'pad=0 3'pad=0 strand=+ repeatMasking=none*

*GGGCCUGCUUCACCCGCGUGCAGCCCACCCCGCUGCGGCAGCCGCGCCUCGUGGCGCUGUCAGAGCCCGCGCUGGCGUUGCUGGGCCUGGGCGCGCCGCCCGCGCGCGAGGCCGAGGCCGAGGCCGCGCUGUUCUUCAGCGGCAACGCGCUCCUGCCGGGCGCCGAGCCCGCCGCGCACUGCUACUGCGGCCACCAAUUC*

*>hg19_ct_UserTrack_3545_(null) range=chr19:8032416-8032615 5'pad=0 3'pad=0 strand=+ repeatMasking=none*

*UGGCUUUCAGGAGCGCGCACCCUCCCGACCCACCUGAAUCUCUGCGCCUGGUGGUGAACGGGGCCUCCGAACCGUCGCGCUGGCGAGUGGUACAGCUGCGAGAGGAGUGCCACGUUUUUGUUCUGGUUGGGGUUGGCUGCAAACUUCACUGUGAUGGGCUCAGAGGAACCUGGGGGUUUAUGACCAUUGAAACUGGUAAU*

*>hg19_ct_UserTrack_3545_(null) range=chr6:128505466-128505665 5'pad=0 3'pad=0 strand=+ repeatMasking=none*

*AGUGUGACUAUAUCAUAUAUCAAAUAAUGAGAACCUUCAACACUGCAUGUAUUAAUGUGUCUUAUGCUAGAUUCCUUCACAAGGUAGAAAAGGUCUGCUACUCAGUCUUACCUGCACAUUUUGUUCUGGUGAUUAGUGGAGGUCCUGGGAGCCCCGUUCCACCUUCACCAGGUCUUGUAAGUAGAACUCGGAUCUCAUAU*

*>hg19_ct_UserTrack_3545_(null) range=chr15:27193222-27193421 5'pad=0 3'pad=0 strand=+ repeatMasking=none*

*AAGACUUCUGAAAGCAAAAAGACUUACAACAGUAUCAGCAAAAUUGACAAAAUGUCCCGAAUCGUAUUCCCAGUCUUGUUCGGCACUUUCAACUUAGUUUACUGGGCAACGUAUUUGAAUAGGGAGCCGGUGAUAAAAGGAGCCGCCUCUCCAAAAUAACCGGCCACACUCCCAAACUCCAAGACAGCCAUACUUCCAGC*

*>hg19_ct_UserTrack_3545_(null) range=chr4:57856931-57857130 5'pad=0 3'pad=0 strand=+ repeatMasking=none*

*AAGGCUUGGUUAGACAACAGCUGGAUUCUUUUGAUGAGUUUAUUCAGAUGUCUGUUCAAAGAAUUGUGGAAGACGCUCCUCCUAUAGACCUACAGGCUGAAGCUCAGCAUGCUAGUGGAGAAGUUGAAGAACCGGUAAGAUAGUUCUAAUAGUUACACAGGUACAAGAAGCGUAUUGGUUUGAAAUUUUAGCCCUUCUCU*

*>hg19_ct_UserTrack_3545_(null) range=chr1:238048365-238048564 5'pad=0 3'pad=0 strand=+ repeatMasking=none*

*UUAAAGGGCUUCCUUGGCUGCAGAGUAGUAGGACAUGGAAACAGUUGUAAGUUGGGGAGGUUCAGAGCCCUGGUGAGUGUCAUGGAAGGGAACAUCCUACCGGUCCCCUGAGGGCCUGUUUCUCCACUGUAGGGUUCACAAAGCUGAAGGUGAAGAUGCUGAAGCGCUGGUGGUGAGAGGGAAAUGGAAGAUCCAAGGCU*

*>hg19_ct_UserTrack_3545_(null) range=chr6:31636309-31636508 5'pad=0 3'pad=0 strand=+ repeatMasking=none*

*GUUUCAGAUGAAGAACUGGAAGACAACCCCAACCAGAGUGACCUGAUUGAGCAGGCAGCCGAGAUGCUUUAUGGAUUGAUCCACGCCCGCUACAUCCUUACCAACCGUGGCAUCGCCCAGAUGGUGAGGCCUCUCUGCUCCUACCUGCCUCCUUCUGAGCAGUAAGAGACACAGGUUCCUGCAGCAAGAAGUCAUGUUUA*

*>hg19_ct_UserTrack_3545_(null) range=chr17:7402510-7402709 5'pad=0 3'pad=0 strand=+ repeatMasking=none*

*UGGAAUUGGUGGGAGGCGGGCAGGCUGGGUGGCUCCUCAAGGUUUCGCUGCAGACAUCUUCCCAACCCUGACUUUUCUCUUUAACUGUAGUGUGACAACUCCGUACAAUGCAGACUUUGACGGGGAUGAGAUGAACUUGCACCUGCCACAGUCUCUGGAGACGCGAGCAGAGAUCCAGGAGCUGGCCAUGGUUCCUCGCA*

*>hg19_ct_UserTrack_3545_(null) range=chr20:42328586-42328785 5'pad=0 3'pad=0 strand=+ repeatMasking=none*

*GAAACGAGCCUGCCUUACAAGUGGGUGGUGGAGGCAGCUAACCUCCUCAUCCCUGCUGUGGGUUCUAGCCUCUCUGAAGCCCUGGACUUGAUCGAGUCGGUAUGUUGGUCACAACACUUCACAGUGAGCACAGAACACCCCCAGCAGGAAGUGCUUCUCUCUCAGGCCCACAUCCUUUUGCUCAUUCCAUCGCCCUGCUA*

*>hg19_ct_UserTrack_3545_(null) range=chr16:67472623-67472822 5'pad=0 3'pad=0 strand=+ repeatMasking=none*

*UGGCCAUGGCCACAGAGUCAGGUCUAAACCACCAGGUUGGCCUCCCUCUGACAAGCAGACCCUGCAGACUCACCUCGUGCUCAAAGAAUCGGUCCUCCAGCGUCUUGUCUCCAGGGUUGCUACCUGCACCCUCGAAGAGCAGCUUGUACUCCUGGCCAGGGGGGUGGGGGGAAGCACAAGCAUGAGGGUUCUGGGUGGGG*

*>hg19_ct_UserTrack_3545_(null) range=chr3:134084618-134084817 5'pad=0 3'pad=0 strand=+ repeatMasking=none*

*CCUGCACACAGGCCAGGCUUCUUAGUGGGGCCCCUACUCACUCUGAGCAAGCAGCUUGGCCACCAUGUCCUGACUGCCGGCCUGGGCCUCCUGUGUCUUGCUUGCCAGGCGGCGAUUUGCAGAUUCCAAUCUCUCUGUUUCAAGGGAAGGAAAGAUGUUUUAGUGUCAGGUGAAGCUGCCAGCUUGGGAUUUUUCCUGAC*

*>hg19_ct_UserTrack_3545_(null) range=chr20:47858560-47858759 5'pad=0 3'pad=0 strand=+ repeatMasking=none*

*GGUGGGGUGGGUCAGAGUGGCCUGGUGUGUUCCACACUCACACCUCCCCACUCCCCCUAGGCAGAGGAAAGGUCUCAGUUUGAAAUCCUCAAGGCGCAGAUGUUUGCUGAACGGCUAGCGAAGAGGAAUCGCAGAGCCAAGCGGGCCCGAGCAAUGCCCGAGGAGGAGCCAGUGAGAGGUCCUGGUAGGUGAAUGGGGAG*

*>hg19_ct_UserTrack_3545_(null) range=chr11:59361460-59361659 5'pad=0 3'pad=0 strand=+ repeatMasking=none*

*UUAUGGUUCUGAGGUUCCUUUACCUGUUCACAGAGGGAUCGGUACCCAUUCUCCUCUAAUCGGUCCAGCUCAAAGGUCUCCCCAAGCAGUGGGUUGAAUGGCUUACUGGUGCGGAAGACAGUAGUGGAGUAGGAGGACACGGUGAAAGCUGCAACAUAACAGAGCUGUUCUAGAGAAUUCUCACAUUUUGCAGCUCGGUC*

*>hg19_ct_UserTrack_3545_(null) range=chr15:75981273-75981472 5'pad=0 3'pad=0 strand=+ repeatMasking=none*

*CUCCCCAAACUGCAGGGCCCCAGUGACGCGGAACAGCACGCUCACAUCCUGCCCCACGGCAUUGGUCUCCACCGACAGGUUGGCGGGCAAGAUGGGCAUGGCAGAGCCUUGGGCCAGUCGCAACCCUGUGCUGCGGUGGAUCUGUAUGGCCGGCCGGAUGGCCACCACCUUCAGCGUGGCCGGGGGGCUGGCCUGCAGUC*

*>hg19_ct_UserTrack_3545_(null) range=chr14:100384034-100384233 5'pad=0 3'pad=0 strand=+ repeatMasking=none*

*GUGUGUGCACGCACUCACGUGUGUGUGUUUUAUGCUAUAGUGUUCUAUCUGUUCAUCAAUUUACACUUCCUUUAAAAUAUUUUAGGAUCCAGCUCAGUCUUCUGGUUUUCAUCCUUCAGGGUCUGUGGUUGCAGUCGGAACACUCACUGGGAGGUAAGUCCAUGCCAACAGCGCUUGUGCUUGCAAAGCUUAUGGAAAAG*

*>hg19_ct_UserTrack_3545_(null) range=chrX:100081623-100081822 5'pad=0 3'pad=0 strand=+ repeatMasking=none*

*GGUGGAAUGCAUGUCAAUGGCGCACCUCCUCUGAUGCAAGCUUCUAUGCAGGGUGGAGUUCCAGCACCAGGGCAAAUGCCAGCUGCUGUCACAGGACCUGGCCCUGGUUCCUUAGCUCCUGGAGGUAAGUUUCAUUUAGUCUUCACCAUUUCAGUACUUGCUAAAGUUUUGUCAUAGCCACUGUUGCAGUGCAGCCAAAU*

*>hg19_ct_UserTrack_3545_(null) range=chr2:191898156-191898355 5'pad=0 3'pad=0 strand=+ repeatMasking=none*

*UCCCAGUAACCAAAUUUAAGAAAACUAAUUUCACAUGAAUAAACAUUAGUGAGUAUAUACCACUUUCAGAAUGGUCCACCCAGGUGAAAGUUAUUCCUCCGAGAUGGCUUUCACUGAAUCUUAAUAAAAAGGUGCCAGGCAUUUUAUCCUUUAGCAACAGCCGUUCCUUCUCUUUGCUAACAAAGCCCAUGACAUACCUA*

*>hg19_ct_UserTrack_3545_(null) range=chr22:30951840-30952039 5'pad=0 3'pad=0 strand=+ repeatMasking=none*

*GCUGCGGGCGAAGAAGGUCGGGUAGUCGAAGUCAUUGCGGCCGUUAGGGAAGGCGAACUUGAGCCGGUGCUUCUGGCCGAAGCGGAACAGGAUGUUGAGCAGGGUGCUGCUGGCCGUCUUGUGCGUCUUCAAGAACACGAUGUUGCGCCGCGGCUGGCACUCCCCCGCCGAGCCGUUGGCCCGGAUCACUGCCUCUGGCU*

*>hg19_ct_UserTrack_3545_(null) range=chr18:3135497-3135696 5'pad=0 3'pad=0 strand=+ repeatMasking=none*

*UUGCUUUGAAAUUUUCUCUUGAAGUAAAAGAAGUUUACAGUUGCUUACCAAGUUUGUCCCCUACCACAGUCACCUCCGUUGCCUCUGAGGGCUCACCAACUCCUGCAGAAUUAGAACAGCGGACACGGAAACAGUAGGAUUUCCCCUCGGCCAAGUCAAACAGAGCAAAGCGGGGAGACUUCACAGGGAGCUCCGUGUUC*

*>hg19_ct_UserTrack_3545_(null) range=chr17:58524960-58525159 5'pad=0 3'pad=0 strand=+ repeatMasking=none*

*GACAUUCUGAGAAAUCAGGAAGGACUGCACCACUUCUUCAGUGGACUGGGGGCUGGUGCUGACAUCUUCAAGAGCAUCUGUCACUGAAUAUUGCCGAUCUCGCAACCGGUUCCAGUUAGACAGAACAUUGUGAUAUUCAAACACUUUCUCGUAAUUUCCAAUGGAGUUGUAAAGUUUAAUGAGACCUCGAUAAUCAUAUU*

*>hg19_ct_UserTrack_3545_(null) range=chr20:17599204-17599403 5'pad=0 3'pad=0 strand=+ repeatMasking=none*

*GUCACACUCCUGGCAUGGGGACAUGGAGGCCACCUACCAGGGCAAGCUCAUCGCUCUGUUUCUGGGCUUCGCUCUUGGCGGCAUCGAGCUGAGAUUGAGAUUCUAGGAGAAGUUGCCUCAGCUUGGGGAGAAAACAGAGGCGGGUGAUGGGAAAAAAGGAGAGAAAAGGAGAAAACCAGUCCCCGUGAGUUAGCGCCGAA*

*>hg19_ct_UserTrack_3545_(null) range=chrX:43652633-43652832 5'pad=0 3'pad=0 strand=+ repeatMasking=none*

*UUCUAAAGGUUUUUAUAUAAGGAAAGUACUCACCCUUUUUCCUCCAGAAAGGCUCUUUAUAAUAAACUAUACACUUGAUGACUGAACCCAAAGGCACACGAGUGAUCAUCUGGUUUCUCAUCAUUGGCAGAGGGGGAUUGAAGUGAAUCUUCAUGCCCAGAGUAGGAGGAAUAGCACUAAUCACAUAUUUAGCCUGAAAG*

*>hg19_ct_UserTrack_3545_(null) range=chr1:26515611-26515810 5'pad=0 3'pad=0 strand=+ repeatMasking=none*

*CUGGGUUCCGAGUGGGGAUUUGAGUCUCACCUAGGCUCCUCGUGCCACGCUGGCCAGGUGCUGGCUUCCAGGCACCGGACCUCCGGAGUGAAGUCUGGCCUCGGGCUCUGCCCACUUCCCUGGGUGAUCAUGGUCCCUUAGCCCCUCCUCUCCACACAGGCAAAGCUGCAGGAGCUGCAGGUCCUAGAAGAAGUGCUGGG*

*>hg19_ct_UserTrack_3545_(null) range=chr20:62196087-62196286 5'pad=0 3'pad=0 strand=+ repeatMasking=none*

*CCCAGGUCUCGGACACUGAGGGCAUCAUCGAGGUUGCAGGCGCCCUGGGGGUCCACAGUGAAGGUCAAGAAGGCGCGGCAGUCCUCUCGGCGGCCGGCAACCCGGCCAAGCUCCGUGUGGUAUUUCUGCAGCACCUUGGUGAUGGUGGCCUGGUCCGACGGGGGCACCCUCAAGCUGUACUCCAGGCCGAGGAUGCGGAG*

*>hg19_ct_UserTrack_3545_(null) range=chr7:134221245-134221444 5'pad=0 3'pad=0 strand=+ repeatMasking=none*

*GGGCACCCAGGCCCUGGACUAAAAUUUCCUGUGAAUGCUUCGGCUAACCCUGUUACGGUGGAUCCUUUAGCAAUUUCUGCCCCAGGGAGGACUAAGGCAAGCCAGGGUCCCUGUAGUCCCUCUAGAGCUGCCCAUAAGGUAUUCCUUUCUAUGAUAGGCCAUGGAGGAGCUGGUGGAUGAGGGGCUGGUGAAAGCCCUUG*

*>hg19_ct_UserTrack_3545_(null) range=chr17:80274082-80274281 5'pad=0 3'pad=0 strand=+ repeatMasking=none*

*CCAGCACACACGCCACCCCCAGGCCCAGCCCGAGGAGGAAGGAGAUCACCGCCAGGGCCGCAGGGAGGGCAGAGGCUGCUGGCGGGUCAGGGAGGGCAGAGGCUGUCUGCGGGUCAGGGAGGGCGGAGCCUGUCGGUGGGGCAGGGAGGGCAGAGGCCCUUGGUGGGGCGUCCGAGCAUCUGUGCCAUCCUUGGGACUGU*

*>hg19_ct_UserTrack_3545_(null) range=chr10:108427292-108427491 5'pad=0 3'pad=0 strand=+ repeatMasking=none*

*CAAAGUUGUGCUUCAUUGUUAAAAUGAACAAAAGCAAUUACAUUGUGCUGUAAGGAAAUAUGCUUGGUCCUUGAUCAGAAGUAAUUCUGUCAGUAUAACACGUGCUGAAAGGCAGCAAAGGUUGGCCUCUCAAGGUCACUACUCCAGACACUUACCCAGUACUAUUGAGGUAACUCUGUCCCAAGCUGCAAUCCUUUGAC*

*>hg19_ct_UserTrack_3545_(null) range=chr2:189913990-189914189 5'pad=0 3'pad=0 strand=+ repeatMasking=none*

*UUCUAAAUCAAUUGCUAAAUGAAAAAAAUUGUUUCCAUGACACCAGAUAACAAGAGAAGAGUUAUUUUCACUGUAGUACUCACUUCUGGUCCAGGUUCCCCUACAGGACCAUUGGAGCCUGGGGGCCCCACAGGUCCAGGUGGACCUUUAUCUCCUGUUGCACCAGUUGGUCCUACUUUUCCUGGUGUUCCCUGAAAUAG*

*>hg19_ct_UserTrack_3545_(null) range=chr6:7845336-7845535 5'pad=0 3'pad=0 strand=+ repeatMasking=none*

*AACAAUAGUUGUCACUCUCUCUUGUUUAAUCUUAUAGUGGAGUACGACAAGGAGUUCUCCCCUCGUCAGCGACACCACAAAGAGUUCAAGUUCAACUUAUCCCAGAUUCCUGAGGGUGAGGUGGUGACGGCUGCAGAAUUCCGCAUCUACAAGGACUGUGUUAUGGGGAGUUUUAAAAACCAAACUUUUCUUAUCAGCAU*

*>hg19_ct_UserTrack_3545_(null) range=chr16:2342121-2342320 5'pad=0 3'pad=0 strand=+ repeatMasking=none*

*GGAAAUGCGCAUUUACUGACCGAAGGAAGACUUCCUCCAUGGUGGUGAUGGAUGCCCCAAAGCUGGCAAUGCCCAGCUCUUUCUGCUUCUUCUCCAGUUUAGCAAAGAGACCUUCAAACCUGAAAAACAGACCCAGCAUUAUGAGUCACUUCUCAGUGACUUUCUAGAUAAUUUGUUCCUAAAGCAUCACCCCCCCUCGG*

*>hg19_ct_UserTrack_3545_(null) range=chr4:3131554-3131753 5'pad=0 3'pad=0 strand=+ repeatMasking=none*

*UAAAAUGUGCUCUUUCCUCAUUGCACUUCCAUGUUGGAGGGCUUGUCUCUUGGUGAUCACACUUCAAAAUUCUCACAGCCCCCCUUGAACCGUUUAGGUGUUAGACGGUACCGACAACCAGUAUUUGGGCCUGCAGAUUGGACAGCCCCAGGAUGAAGAUGAGGAAGCCACAGGUAUUCUUCCUGAUGAAGCCUCGGAGG*

*>hg19_ct_UserTrack_3545_(null) range=chr12:57501387-57501586 5'pad=0 3'pad=0 strand=+ repeatMasking=none*

*CCUCAAGGGUGCUGAUGUGUUGCAAGAUGGUGCUCCCCUCCCCCUGCUCUCCCACCGAGGCCUGAAGGUGCUGGACAGUGUCUGAAAGUAGGGCACUAGCCAAGUUGCAGCAGAAGGCGUCGGAGCCGACCAGGAACUCCCUGCAGGAAGAUCAGGAUGAGGCUACCUCAGGCCAGGGCUUCCUCAGCUCUCCUGACCCU*

*>hg19_ct_UserTrack_3545_(null) range=chr20:10654017-10654216 5'pad=0 3'pad=0 strand=+ repeatMasking=none*

*GCUCGCGGGGCUCAACCGCCCAGGGCGCCGCGAGGGGAGGGAGAGGACGGCUGGGAGGGAGGCCCGGAGAAGGGCUCCUACCUUGGCUCGCAGGGCACAGAGCAGGGCGAGCAGGAGGCUUAGGGGGCGCCCGGACCGGCCGCGCGUCCGUGGGGAACGCAUCGCUGCGCCGCGCGCCGCGGGCACUCGGGACGCCGCCG*

*>hg19_ct_UserTrack_3545_(null) range=chr14:93397682-93397881 5'pad=0 3'pad=0 strand=+ repeatMasking=none*

*GAGCCAGGCCCCAGGCCCUCCCGGAGCCCAUGCAGGAGUCCAAGGCUGAGGGGAACAAUCAGGCCCCUGGGGAGGAAGAGGAGGAGGAGGAGGAGGCCACCAACACCCACCCUCCAGCCAGCCUCCCCAGCCAGAAAUACCCAGGCCCACAGGCCGAGGGGGACAGUGAGGGCCUCUCUCAGGGUCUGGUGGACAGAGAG*

*>hg19_ct_UserTrack_3545_(null) range=chr11:130286078-130286277 5'pad=0 3'pad=0 strand=+ repeatMasking=none*

*UCUGUGAGAUACAUGGCGCUGCAGGGGGACCAGGGCAGCGUCUGGUUCAGGUGGACGAACAGCGGUGCCAUCACGUGGUGCUUGCCCAUGGGCCCGAAGAGCCGUGUGCAGGGCUUGGAGUCGUCGUGGGGCAUGCUGAGGACGUGCCCUGGGGAGAGAGGCCUGGUCCACUCCGCCCUGUCCUGCCUGAGGGCGCCCCA*

*>hg19_ct_UserTrack_3545_(null) range=chr17:79991511-79991710 5'pad=0 3'pad=0 strand=+ repeatMasking=none*

*GGGAGGCGCAGUAAGGGCCUCCCUGUACCCCACCCUCACUGUCUCCCCUCCUCACUGCCGCUAGGCUCUGUGAAAUACCUGGAGUGCUCAGCCCUGACCCAGCGGGGCCUGAAGACAGUGUUUGACGAGGCGAUCCGCGCGGUGCUCUGCCCGCCCCCAGUGAAGAAGCCGGGGAAGAAGUGCACCGUCUUCUAGAGCCC*

*>hg19_ct_UserTrack_3545_(null) range=chr12:54798378-54798577 5'pad=0 3'pad=0 strand=+ repeatMasking=none*

*GGAAUUAGCCAGAACUGCACCCAGGAACUUAAGGAAAGGUGCAGAAGAGAGAGUAGGGGUCAGUGACCAGGGAAUUUUAAGGUGAAAAGGGGCCUCAGGCACAACUCACCAGGAUAUCCAUUGCCAUCCAGGUCUCGGCCUCCUCGAAGGGCAGAGCCAAAGAAGUCUGGGGUGUGGCUGGCUGCCCACAGGGGCUGCAG*

*>hg19_ct_UserTrack_3545_(null) range=chr22:20229211-20229410 5'pad=0 3'pad=0 strand=+ repeatMasking=none*

*CACGCUCUUGUGUCCGCUGGGGGUCAGCAGGGCCCAAGCACUGUCCACAGCACCAGCGCCAGGCCCAGGGGGGUGAGGCUGCAGGUGAGGCUGGGUAGGGCACCUGAGCCUUCUGAGUCACCAGUCCCGCCACCCCCGCUGCCUGCCUGGCCCAGACGGCAGUGGCUGCGGGUGCGGUUCUUGCGUGAACAGCCUGGCCU*

*>hg19_ct_UserTrack_3545_(null) range=chr19:42509895-42510094 5'pad=0 3'pad=0 strand=+ repeatMasking=none*

*UAGCCCUUGGUGUCGAGGAGUCCCCCGAUCUGGGUGAGGUUGCAGUUGAGGCGCCGGUGGUAUUCGUUCAUGGUGGACUCGAGCAGGAAGGCGUAGCGGGAGUUGAGGACGCGGGCAAUGCCCUCUUCUGUGCUCUUGACGAACACGCUGGGCUGCUUCGACUGCAUGUAGUUCCACAUGCGCUGGUACGUUUGGUACCG*

*>hg19_ct_UserTrack_3545_(null) range=chr10:96580291-96580490 5'pad=0 3'pad=0 strand=+ repeatMasking=none*

*CUUGGUAAUCACUGCAGCUGACUUACUUGGAGCUGGGACAGAGACAACAAGCACAACCCUGAGAUAUGCUCUCCUUCUCCUGCUGAAGCACCCAGAGGUCACAGGUAUGAUCACAGAGGAUGAGUUAAUUGAGUUUUAGGAAAGAUGUUGGGAAGGUGCUGCUAGUGUUCUCCUUUCUGUUUCUCUUAGAGAAGUUCCAU*

*>hg19_ct_UserTrack_3545_(null) range=chr14:23778069-23778268 5'pad=0 3'pad=0 strand=+ repeatMasking=none*

*GCGGCGUCUGCGGGAGGGGAACUGGGCAUCAGUGAGGACAGUGCUGACGGGGGCCGUGGCACUGGGGGCCCUGGUAACUGUAGGGGCCUUUUUUGCUAGCAAGUGAAAGUCCAGGGCCAGGUGGGGCUAGGUGUGGCUGGGGGCCAGGAGAGCAGGAACAGAACAGAGAAAUGCCCUUGGAAGAAGUGGAGUUGGUGGAU*

*>hg19_ct_UserTrack_3545_(null) range=chr5:137527855-137528054 5'pad=0 3'pad=0 strand=+ repeatMasking=none*

*GAGCCAAAACCAUCCACUGUGAUUUGUCAAUAGCUAUCAAAAGAGGAAAAAAAAGUUAUUAGCCCUUCCCAUAUCAGACGCAUUGUCAAGUGACCAGGCUUACCGAAGCUGGUGGGCCCGUCUAUAAUAAUAAAGGCAGUAAAAUGGCAUCUUAAGGAUUUCAUAGGUCUGCCCGAGGCCAUACCAAGCUCUGUAGUCCC*

*>hg19_ct_UserTrack_3545_(null) range=chr3:52257934-52258133 5'pad=0 3'pad=0 strand=+ repeatMasking=none*

*UUGUAGCUCAGGUUUAGCUCUUCCAGGGUGGGCACAGCCAAGAAGGUGCUGGGCUCGAUGGUCAUGUGGCAGGGGAAGUGCAUGGGGCUGAGGCCAACCGGCGGGCAGUUCCACUUGAGGUUGAGAUGCCGCAGGCUGGGCAGGUGGGCAAAGUCAGAAUCAUGGAGGUGGUGGAUGCGGUUGGAGGACAAGGAAAGGCU*

*>hg19_ct_UserTrack_3545_(null) range=chr2:220285174-220285373 5'pad=0 3'pad=0 strand=+ repeatMasking=none*

*UGUGGGCCCUGAGAGGGGACUGAAGCCCAGUCAUGCCCUACAGGAGAUCCGUGAGUUGCAGGCUCAGCUUCAGGAACAGCAGGUCCAGGUGGAGAUGGACAUGUCUAAGCCAGACCUCACUGCCGCCCUCAGGGACAUCCGGGCUCAGUAUGAGACCAUCGCGGCUAAGAACAUUUCUGAAGCUGAGGAGUGGUACAAGU*

*>hg19_ct_UserTrack_3545_(null) range=chr2:37509574-37509773 5'pad=0 3'pad=0 strand=+ repeatMasking=none*

*AUCUUUAAGAAACAAAAUAAAUCAGGAAAACAGAACCUUAUCCAAAGAUAAUACACCAAAGAAUCAUGGCCUUUUCUGAUCUUUCACAAAAAUUAAGAAAAAUAAUUUUACCUCCAUUGAAAGUAACCUCUCCAAGGCAGUCUCUUGGUACUUUUGAUGCACAGCGUUUAUGGCAGUUGAAUUUGCAAUCUAAAAUGAAA*

*>hg19_ct_UserTrack_3545_(null) range=chr1:53678813-53679012 5'pad=0 3'pad=0 strand=+ repeatMasking=none*

*GUAGAGCCUUCCCCCACUCUCAAGGAUGCUGUGAGGGGUAUUCCUACCAUGUGGUGAGUUGGGAGGUUUUCCUGAGGUCCUUUUCCAUCCUGAGACUCUGGUUUUCCAUUUUGUUUCUCACAGGCCAGGGCUUUGACCGACACUUGUUUGCUCUGCGGCAUCUGGCAGCAGCCAAAGGGAUCAUCUUGCCUGAGCUCUAC*

*>hg19_ct_UserTrack_3545_(null) range=chr4:967047-967246 5'pad=0 3'pad=0 strand=+ repeatMasking=none*

*UGGCAGAAGGUGGGCUUGGUGAGCGUCACCUUCCGGAAGCUGUGUCCCGGCGCGGCAGCGGGGCCCGGGGCUCUGACGCCCGCCCGCUCGGGUCCCGGCCCCGGCCCCGGCCCCGGGCGCGCGCGGCCUCCUGAGCCCAGCACGGGGCUGCAGGCCGGGCUGCCGGGGCGCGGGGAGCCGCCGCCCAGCCAGGCGCGGGC*

*>hg19_ct_UserTrack_3545_(null) range=chr22:26074779-26074978 5'pad=0 3'pad=0 strand=+ repeatMasking=none*

*CUAGGUAUGCAAUGAAAUGCUUAGAUAAGAAGAGGAUCAAAAUGAAACAAGGAGAAACAUUAGCCUUAAAUGAAAGAAUCAUGUUGUCUCUUGUCAGCACAGGAGUAAGUAUUCAAUUUCCAGCAUUUCUUUUAAAAAUGUUUUGUUUGUUUCAUAGCAGGAUGAUUUAUUAUUUUUCCUUCUAGCUGAUUGCAAGGCCA*

*>hg19_ct_UserTrack_3545_(null) range=chr2:152541233-152541432 5'pad=0 3'pad=0 strand=+ repeatMasking=none*

*AAGUCCAUUUCAGUUUCCAUCUUUUUAAAAGAGGGGCUACAGAAACCCUGGUUACUCACAUCGCUCUGCAGCGCAUAUGCCUUCUUGGCAAGGUCCACAUUGAUGCUAUCAGGGGGGUAGCUGUAACUGUGUAAGAUGUGCUUAUAAUCAACGUCGCUGGCAAUUGCCUGAGAUUUCUUAGCUUGAGUGACUUGGAGCAU*

*>hg19_ct_UserTrack_3545_(null) range=chr21:47810952-47811151 5'pad=0 3'pad=0 strand=+ repeatMasking=none*

*UCCUGGAAGCUGCUGGCUCUUAGCUCUGCUGCUCUCAGGGGCAGUGGCCCCGUCCCUUCCUCCAAGCAGGCUCUCCUCAGUUACACCCAGGACUGGCUUUGUCAGAGCAUCUGUGUCUCCCACAAAGGUACCCUUGAGGGAUUCAAGGUGGAGACAGCAGAUCUGAAGGAGGUGCUGGCCGGGAAGGAGGAUUCCGAGCA*

*>hg19_ct_UserTrack_3545_(null) range=chr9:101825195-101825394 5'pad=0 3'pad=0 strand=+ repeatMasking=none*

*UAAGAGUCACAGUUACAGACAAUGGCAAAUUAUUUAAAGCAUCUUCUCAAGAAACAUUGCACUUGCACCUCAAUACACUCUGAAGAACAGAUUGAAUGCAUAUUCAGAACUGUUUCUUUUCCUCCUUUUCAUAACAGCCACAUCAGCUUCUGCCUCCACCAAACCCUAUUUCAAGUGCCAAUUAUGAGAAGCCUGCUGUA*

*>hg19_ct_UserTrack_3545_(null) range=chr3:182737838-182738037 5'pad=0 3'pad=0 strand=+ repeatMasking=none*

*AAAAGGACAUAAAUGACAAGUUUAACAAAGCCACGUAUUAAAACUUACUGAACAUCAUUCUACAGAUGUCAUGUGAUUACCUUUUCAAUGGUUCCAGUCAUAGGAGCUAAGGGGCCGCCCUGAGUUUCUUGUGAGCUCACAGAAGAUAAGUAUUUGGGGACUGGAAUGUCAAUCUCAAUACUUCCUUCCUAGAAACAGAA*

*>hg19_ct_UserTrack_3545_(null) range=chr10:81109323-81109522 5'pad=0 3'pad=0 strand=+ repeatMasking=none*

*UUCACUGCUGGGGAUGUAGCAGGGAUUUUGGCUGGAACGGGUAUGACCCUGGAAUUGGGGCCUGGCCCUGUUGACCUGUGUUUCUCUUCGACCCUCAGAGAACUUCAGAGCCCUGUGCACUGGUGAGAAGGGCUUCGGCUACAAAGGCUCCACCUUCCACAGGGUGAUCCCUUCCUUCAUGUGCCAGGUAAUGUAGUUUC*

*>hg19_ct_UserTrack_3545_(null) range=chr22:38221299-38221498 5'pad=0 3'pad=0 strand=+ repeatMasking=none*

*GCCGACGCCGCCACCGUGCCCGCCGCGCCUUGCGUCGCGUCCGCCCCGCGUCCUCGGGCCCACCCGGCUGCCCCGGAGACGCCCGGCCUAGCGGGAGGCUGCUGGCUGGUGGCGGCCAGGGCCCGGAGCCCAGGGAGGGACCCGUCCACGGCGGAGAGGCUGCCCGAGGACCGGAAUAAACCCUGCCGCCUGGACUCCGC*

*>hg19_ct_UserTrack_3545_(null) range=chr1:65321035-65321234 5'pad=0 3'pad=0 strand=+ repeatMasking=none*

*UGAGUUGGGCACAGAAGUGUAAUUAAACUCAAUAAAAAACACCUAGAAACUGUGGGGAGGGUCCACUUUGUUUAAGUCAGUCAGCGUGUUUUGGUCGAUCUGGGUCUCAGCACAUUACUGAUGGGAUACAGCCUGGCUCUGGCACAGGGAGACGAACCUCGGGGCUUGGGCUGGCAGCAGCGUUUUAGCAUGAAGCUGAU*

*>hg19_ct_UserTrack_3545_(null) range=chr1:214820242-214820441 5'pad=0 3'pad=0 strand=+ repeatMasking=none*

*UCAAACACAAUUAAAAGAGCUCAAUGAGAGAGUGGCAGCCCUGCAUAAUGACCAAGAAGCCUGUAAGGCCAAAGAGCAGAAUCUUAGUAGUCAAGUAGAGUGUCUUGAACUUGAGAAGGCUCAGUUGCUACAAGGCCUUGAUGAGGCCAAAAAUAAUUAUAUUGUUUUGCAAUCUUCAGUGAAUGGCCUCAUUCAAGAAG*

*>hg19_ct_UserTrack_3545_(null) range=chr20:56083641-56083840 5'pad=0 3'pad=0 strand=+ repeatMasking=none*

*CUGUGACUUAAGUAAGCUUACCCAGCGGGAAAAGCCUUUGCCACACUUGGAGCAUUUGUAAACAGUCGGGAUGAAAUUUGCAUCGUGGUAUUUCCUGAAGUGAGCGUUUAGAAGUUGCUUCUGUCGGAAACAUUUAUUGCAAGAAAGGCAGGUGAAUGGUUUCUCUCCAGUGUGGGUACGAAUGUGAGCGGUCAUAUGAC*

*>hg19_ct_UserTrack_3545_(null) range=chr5:149497042-149497241 5'pad=0 3'pad=0 strand=+ repeatMasking=none*

*AGGGAUAAGUACUUACCCUACGUAACUUACCUCUGAGGCAAACCUGGCAGCGCGCGCGCGCGCGCGCACACACACACACACACACACACACACACACACACUGUGCACAAUUUCCUUGGCCCCAGGCCAGGGUGGUUACCUGGCUAGGCUGGGGGAACCCUCCAGUGGGCCCUCGUCAGCAACCUCGGGUUUGGGGUCAG*

*>hg19_ct_UserTrack_3545_(null) range=chr7:123332257-123332456 5'pad=0 3'pad=0 strand=+ repeatMasking=none*

*AAAAGUAUUUUUAAAUUUAUAGGGUUAAGAAUGAAUUAGCAAGAGGAAAAAAGAAUACACUUUUCAAAAAUAUCUUUCAAUAAAUUACUUUAAAAUUCCAUUAUGAUAAAGGAUCUAAAAUGAAAAAAAAAAAAGCUUACUUACAGAUUUUAGUUGGAUACCCUGUCGUAUCUGGUCUAACAGUGCAUCUCGUCCAGAGC*

*>hg19_ct_UserTrack_3545_(null) range=chr6:31996973-31997172 5'pad=0 3'pad=0 strand=+ repeatMasking=none*

*UUUGGGGGAGAAAGCAAGUGCUGGGCUCCUGGGUGCCCACGCAGCUGCCAUCACGGCCUAUGCCCUGACACUGACCAAGGCCCCUGCGGACCUGCGGGGUGUUGCCCACAACAACCUCAUGGCAAUGGCCCAGGAGACUGGAGGUGAGGGGUGAGGGGCUCUGGCAGUGAGCCUGAGGCCCAGGGGACCUUAGGAUCCCU*

*>hg19_ct_UserTrack_3545_(null) range=chr2:176957708-176957907 5'pad=0 3'pad=0 strand=+ repeatMasking=none*

*GGUGGCGGCGGCGGCGGCGUCAGGCCAGUGCCGCGGCUUUCUCUCCGCGCCUGUGUUCGCCGGGACGCAUUCGGGGCGGGCGGCGGCGGCGGCAGCGGCGGCUGCGGCGGCGGCGGCGGCAGCCUCCGGCUUUGCGUACCCCGGGACCUCUGAGCGCACGGGCUCUUCCUCGUCGUCGUCCUCUUCUGCCGUUGUAGCGG*

*>hg19_ct_UserTrack_3545_(null) range=chr12:121001516-121001715 5'pad=0 3'pad=0 strand=+ repeatMasking=none*

*GUGGCUUGGGUGGCUUGAGCCCUUGGGCAUGGGGAGGGGUGGGGAGCCUCCUGGGGACUGGCCAAGACUGGUGUGUGAUAGAGCCCCUUGUCCUCAGCGGAAGAUGGACAGCAUAUGUUCCUGCACCCUGUGAAUGUGCGCUGCCUCGUGCGGGAGUACGGCAGCCUGGAGAGGAGCCCCGAGAAGAUCUCAGCAACUGU*

*>hg19_ct_UserTrack_3545_(null) range=chr11:92715008-92715207 5'pad=0 3'pad=0 strand=+ repeatMasking=none*

*AUCCACUUCCUCCUCCCUAUCGCUGUCGUGUCCUUCUGCUACCUGCGCAUCUGGGUGCUGGUGCUUCAGGCCCGCAGGAAAGCCAAGCCAGAGAGCAGGCUGUGCCUGAAGCCCAGCGACUUGCGGAGCUUUCUAACCAUGUUUGUGGUGUUUGUGAUCUUUGCCAUCUGCUGGGCUCCACUUAACUGCAUCGGCCUCGC*

*>hg19_ct_UserTrack_3545_(null) range=chr7:107577428-107577627 5'pad=0 3'pad=0 strand=+ repeatMasking=none*

*UUUUAAAAUUUAUUAGAAAACUUUUUUUUUUCCCCAAAAGUGCUAUAUUAAAACGUUAGACAUAUCGUUAAAUCCAUUGUCUGGGACUAUUUGCACCAAAAAAUGCUCACCCCGAAUAUCUGAGUUUUUGAUAAAUUCCAGUUGUUCAGCAAGUUCUUUCACAGUGUUGUCUAGGCUUUCGGCUUCUGUCUGUAGAGAAU*

*>hg19_ct_UserTrack_3545_(null) range=chr6:169625210-169625409 5'pad=0 3'pad=0 strand=+ repeatMasking=none*

*UCCACGCCCAUGAGCUGAGAGAGCACCCACCGUCCAAGUCCUCCUGGUCUGGGUUGAACACAAGCCGGCAGUUGUCCCUGUCAUCGGGGACGCCAUCGUUGUCAUCAUCAGGGUCACAGGCGUCGCCCUGGCCGUCUCUGUCAUGGUCAGCCUGGUUGGCGUUGGAGAUGUAGGGGCAGUUGUCCUGGUUGUUCUGGUGG*

*>hg19_ct_UserTrack_3545_(null) range=chr1:209965503-209965702 5'pad=0 3'pad=0 strand=+ repeatMasking=none*

*GAGACAGGUAGUUUUUCAAUACAUGGCAAAGAAAUGAAGUUAGAAAGCAGGACAGGAAAGAGUCUAUAAUAGAAGCAGAAGACCGAGCAAGAAAGAUAAAGUCUCACUUACUUGGGAGAGAGCUGAUCCACAGUUCUGGAGAGCUAUAGAAGGGCUGUAUAGGUGCCUGGGGUACUUCCAUCUCCAGGGGUUCAGUUUUG*

*>hg19_ct_UserTrack_3545_(null) range=chr17:72838843-72839042 5'pad=0 3'pad=0 strand=+ repeatMasking=none*

*GCUGCCAGGCGGGGGCCCCUGCCUGCUCGCCCUCCUGGCAGGCCUCCCGGUAGAUCGGCAAGCACAUCGACUGCGCCUGCGCCAAGCGCCUGCAGGCCGAGUGGCCGUCGGGGCGGGCGCAGGCGGGGCCGGUGCACCCAGCGGGCAGCGAGCUGGGCCGAGCGAAGGCCUCGGCCACGGAGCUGGGCAGGGAAGCGUGA*

*>hg19_ct_UserTrack_3545_(null) range=chr2:29551102-29551301 5'pad=0 3'pad=0 strand=+ repeatMasking=none*

*GUGUCAGUGGAUAUCAGGAAGGCUGUCCAUGCUCUCAUGCCUGGGAUAAUGGGGACAACGGGGUUAUGAGCAUGGGCUGGGCUCAGGCAGGGUGGGGCAGCCCCAUCUACUCACGGCACAUCUGGCUCUCAUCUUCUCCCUGGGCACAGUCCUGGUGGAAGUCACAGGCCUGCCCAAGCUGGAGGACUGUCCCAUUCCAA*

*>hg19_ct_UserTrack_3545_(null) range=chr6:144808524-144808723 5'pad=0 3'pad=0 strand=+ repeatMasking=none*

*AUUUUUGUGAAUUAGUAAUUUACUUAACUUUUGGUCCUGUUACUUAGGUAGAUUUUUAGAAAAUAUAAUUACUUGUUUAACAAAUAGACAUUUUGGGGAUCCUUGAUACCUUUUGCCUUAGUAAAUGCAUUAUUAUUUUUUUUUCCCAUCUCCAUUCCAGUCUCUGGAAUCUGUUCUGCGCCACCCGGCAGAUAAUCGCA*

*>hg19_ct_UserTrack_3545_(null) range=chr9:137623210-137623409 5'pad=0 3'pad=0 strand=+ repeatMasking=none*

*AGCACAGGGCCUGAUGGAUCUGGGAUCUCUGCCCAGUUGACCGGAUAGCCACAGUGCCCGCCCAGGGCCUGAUGGAGAGGCAGUGCCUGGUGCGUUUGCGAGGCAACCCUGCGCCUUCCUCUCCCUCUGCAGCCAGCUCCGCCUCCAGGGGAAGGUGCGGAUGACUUGGAGGGGGAGUUCACUGAGGAAACGAUCCGGAA*

*>hg19_ct_UserTrack_3545_(null) range=chr8:74585320-74585519 5'pad=0 3'pad=0 strand=+ repeatMasking=none*

*AAACCAAAAAAGACAUACAAACCUCUGAUUGUACAUGCCCCGAAAGUUGUAAUUAGCUCUAUAAUUUGGGAAUGGCUUUGGAUCUAAUGGCCUGUAGAUGGCAGGCUCUCCCCUUUUCAUAGCAAGCCCAUUCAGUUCCACAGUUGGAGUUAUACUGCCUGUUUAAAAAAAAAACAUUAAAGGCACACAAGUGAAAUAUC*

*>hg19_ct_UserTrack_3545_(null) range=chr6:30459281-30459480 5'pad=0 3'pad=0 strand=+ repeatMasking=none*

*GUCAGGGCCCCUUACGUUCCCCUCUUUUCCCAGAGCCGGCUUCCCAGCCCACCAUCCCCAUCGUGGGCAUCAUUGCUGGCCUGGUUCUCCUUGGAUCUGUGGUCUCUGGAGCUGUGGUUGCUGCUGUGAUAUGGAGGAAGAAGAGCUCAGGUGGGGAAGGGAGAAGGGUGGGGUCUGAGUUUUCUUGUCCCACUGGGUGU*

*>hg19_ct_UserTrack_3545_(null) range=chr10:85973931-85974130 5'pad=0 3'pad=0 strand=+ repeatMasking=none*

*GUCGUGGCCAUCACUGUCCUCAUCUCCACCGCCACCUUCUGGCGCAACAAGAAGUCUAACAAGGUCCUGCCAAUGCGGCGGGUGCUCCGCAAGCGGCCCAGCCCUGCGCCCCGCACCAUCCGCAUUGAGUGGCUCAAGUCCAAGAGCACCAAAGCCGCUACCAAGUUCAUGCUCAAAGAGAAACCUCCCAAUGAGAACUG*

*>hg19_ct_UserTrack_3545_(null) range=chr6:108227849-108228048 5'pad=0 3'pad=0 strand=+ repeatMasking=none*

*UUAACUGCUCAUCGGGGGAUUUGGGAAAUUAGCCUAACCUGUGGUAUUAGAAUAUUAUCCGUUGGUCUGCUUGUGGCAUCUUUAUUAUACUGAGGAUCAAAUUCAGAAGCUCCAGCCAAAACCAUGAUAAGACCUAACAAAACAAAAGAAAUAUGAAGGUAAAUAAAUAUGCUCAUUCAUUAAUUCCCCCUCCUGAGAUA*

*>hg19_ct_UserTrack_3545_(null) range=chr17:13977527-13977726 5'pad=0 3'pad=0 strand=+ repeatMasking=none*

*ACACAGUGUAGCGCUUACCUUAGUGGCUGGCUUUGCUUCUGGGGAGGUGUAGUCAUCAUUUGAUAAGAAGUACGAAUCAUUUAACCUUUCUUCCACUUCUCUCUCUAUUAUAGGUUGCGUAGGAGGCUCUGUCUGGUAUCUUGAAAGAAGAACUAUACAGGACUCCCCUCACAAGUUCUUACAUCUUCUCAGGAAUGUCA*

*>hg19_ct_UserTrack_3545_(null) range=chr9:99003048-99003247 5'pad=0 3'pad=0 strand=+ repeatMasking=none*

*CAUGGGCAAGGCAGCCACAGGUUUCACCUCCAAUUGUGACAUAAUUCAAUGACUCUUUGACAAACUCAUCAGCAGUCUUGGUUAUCACAUUUGUAUUUAGAUACUUUGUCAUUGCAGUCGAGACAGCAUAUGGGGUCAGCACCUACAACGGGAAACAAAGACCAUGGCACAGAAGCAAUCCACCCCAGGAAGAAGACUCA*

*>hg19_ct_UserTrack_3545_(null) range=chr2:176996231-176996430 5'pad=0 3'pad=0 strand=+ repeatMasking=none*

*AAAACAACAAGGACAAAUUUCCCGUUUCCCGGCAGGAGGUGAAGGACGGGGAAACGAAAAAGGAAGCCCAAGAGCUGGAGGAAGACAGAGCCGAAGGCCUGACAAAUUAACUUCUACCUUUAAAAUUUACCACAGACUAUUAAAACUAAUAAUCACCAUAUGCUGUGGACACCACCUAUUUUCUUUGUUGGAAAGGACCU*

*>hg19_ct_UserTrack_3545_(null) range=chr8:1497235-1497434 5'pad=0 3'pad=0 strand=+ repeatMasking=none*

*GGCUUCCACACGCUGCAGUACCAGAGGACGUCCGCGGCCGCCGAGCAGCGCAGCGAGAGCCCCGGGCGGAUCCGCCACCUGGUACACUCCGUGCAGAAGCUCUUCACCAAGUCGCACUCGCUGGAGGGCUCCUCCAAAAGCAACGCCAACGGCACCAAGGCGGACGGCCGGGCGGACGACCACCACCACGCCCACCACGC*

*>hg19_ct_UserTrack_3545_(null) range=chr2:172967000-172967199 5'pad=0 3'pad=0 strand=+ repeatMasking=none*

*GGAACCCAUGUGCGCGUAGGGCGAGCCCCCGCCGCCGCCGCCGCCCGCCGGGUGCUGCUGGUUGGUGUAGUAGCUGCUGUCGGUGGCGGUGGACACCGGAAGGGUGGGCGACUCCUGGGGCUUGUGGAGGCUGCUGCUGCUGCUGCUGUUGCCACCCGGGCCGGCGCCGCCGCCGCUCGGGGGCUGCUGGUGCUGGUGGU*

*>hg19_ct_UserTrack_3545_(null) range=chr21:47423662-47423861 5'pad=0 3'pad=0 strand=+ repeatMasking=none*

*UCUUCUCAGAUGGCAACUCGCAGGGCGCCACGCCCGCUGCCAUCGAGAAGGCCGUGCAGGAAGCCCAGCGGGCAGGCAUCGAGAUCUUCGUGGUGGUCGUGGGCCGCCAGGUGAAUGAGCCCCACAUCCGCGUCCUGGUCACCGGCAAGACGGCCGAGUACGACGUGGCCUACGGCGAGAGCCACCUGUUCCGUGUCCCC*

*>hg19_ct_UserTrack_3545_(null) range=chr5:33963770-33963969 5'pad=0 3'pad=0 strand=+ repeatMasking=none*

*GUAAAGAAAAAAUGUUGCAUCUUUACCUGUUCAGCAUGAUUUUUGUUUUUUGCUCCCUGCAUUGCCAGCUCUGGAUUUACGUAACCAUUUUUAACUUUCUCGAUAGAACCAUACUCGUACAUUCCAUCUGAUGACAAUGGAGGGUCCUGAGGGGUUUGCUGUGGGGGAAUGCCCUUUGCAACCUCUGUAAGUGGGGCUUC*

*>hg19_ct_UserTrack_3545_(null) range=chr5:150922654-150922853 5'pad=0 3'pad=0 strand=+ repeatMasking=none*

*UCGUACUGGCACCAGAGAGUUCCAGUGAGGAGGGCCUCCAUCUUGGGCUUUGAUGAAGAAGUCAAGGGUCUGAUUUUCCAAUCCCACCAGGCUGUCUUUCACCUUGACCACACCAGUGACUGGGUUAAUUUCAAUGACAUCUUUAACUAGGUCCUCUGGGUUCACUGAGUAGGUGACAUCUGCGUUCUGACCUUCAUCUG*

*>hg19_ct_UserTrack_3545_(null) range=chr2:209302489-209302688 5'pad=0 3'pad=0 strand=+ repeatMasking=none*

*UGCUUUCCGACACUGUAACCCCAAUGGAACAUGGGAUUUUAUGCACAGCUUAAAUAAAACAUGGGCCAAUUAUUCAGACUGCCUUCGCUUUCUGCAGCCAGAUAUCAGCAUAGGAAAGGUAAUGGAAUUUCUCUAUUUGUGAAUUCCUAAGGGAAAGCAGAAUAAUAUUUUAAUGCAAUUCUCAAUUGCCAGCCAUUAUG*

*>hg19_ct_UserTrack_3545_(null) range=chr11:5632100-5632299 5'pad=0 3'pad=0 strand=+ repeatMasking=none*

*GACCUUCCUGUCUGGAAAAGCAUUAUGACUGUAGUGUCCUGGGCUCCCAGCACUUCUCCUCUGGUAAGCAUUACUGGGAGGUAGAUGUGGCCAAGAAGACUGCCUGGAUCCUGGGGGUAUGCAGCAAUUCACUGGGACCUACAUUCUCUUUCAACCAUUUUGCUCAAAAUCACAGUGCUUACUCCAGGUAUCAGCCUCAG*

*>hg19_ct_UserTrack_3545_(null) range=chr1:91989803-91990002 5'pad=0 3'pad=0 strand=+ repeatMasking=none*

*AUUUAAAAAGGGGGAUAGUAAUAGCUGUGAGCAUUGUUUUGAUGAGUAUAAUACCAAUUUAGAAGGCUGGAAUGAGGUACCUGAUGAAGCUUAUGACCUGCUUGAUAAACUUCUAGAUCUAAAUCCAGCUUCAAGAAUAACAGCAGAAGAAGCUUUGUUGCAUCCAUUUUUUAAAGAUAUGAGCUUGUGAUAAUGGAUCU*

*>hg19_ct_UserTrack_3545_(null) range=chr13:110435472-110435671 5'pad=0 3'pad=0 strand=+ repeatMasking=none*

*CGGAGAGCGGAGACCGCUGCCGGCUGUCGCUGCUGGUGCCCGGGGUGCCUGAGCCCAGCGACGAGGCCGGGCUGCUGGCGGACAAGAGCGAGGAGGACGAGGCCGCCGACGCCAGCAGGGGAGGCGCGGGCGGCGACAGGCGGGCCCCGGGCUCGCCAAAGUCGAUGUUGAUGUACUCGCCGGGGCUCUUGGGCUCCGGU*

*>hg19_ct_UserTrack_3545_(null) range=chr20:32005523-32005722 5'pad=0 3'pad=0 strand=+ repeatMasking=none*

*ACCUGGGCUCCGGGUCAUUGGGGGUGCACCUCUUCGAGACAUAUGCCAUCUUCAAGGACAUGUGUUUGGCCUCGCUGAAGUUCCGGGGUGUGGGGCCAGGGGAGGAAGGCUGCCGCUGAAGGGGUGAGGCAGGAGGUGAGUCCCAGCCGACCGAGGUCCCACCAGUAGAGUUCUUGAAAUACGGUGAGACGUCCUUCAUA*

*>hg19_ct_UserTrack_3545_(null) range=chr14:105242911-105243110 5'pad=0 3'pad=0 strand=+ repeatMasking=none*

*AGGUGCCAGCACCCCGCCAUCCCCGUGUCCCUCCUAAGCGCUGGGGCUGCCCAAGUGCCUGGCCUGGCCGCCACAGCCCACGUACCGCUCCUCAGGAGUCUCCACAUGGAAGGUGCGUUCGAUGACAGUGGUCCACUGCAGGCAGCGGAUGAUGAAGGUGUUGGGCCGGGGCCGCUCCGUCUUCAUCAGCUGGCACUCUG*

*>hg19_ct_UserTrack_3545_(null) range=chr2:189851712-189851911 5'pad=0 3'pad=0 strand=+ repeatMasking=none*

*CUUCUAAAUGCUUUUUAAAAGAAUUAUGAACUGUCUGUUAAAAUGAUCAUAUCUAUUUGUCUCCUUGCCACAGAACUAUUCUCCCCAGUAUGAUUCAUAUGAUGUCAAGUCUGGAGUAGCAGUAGGAGGACUCGCAGGCUAUCCUGGACCAGCUGUACGUACAAAUGUUUCUCAGCAUUUUGGAGCUUUAUUAUCUUUCU*

*>hg19_ct_UserTrack_3545_(null) range=chr6:142399551-142399750 5'pad=0 3'pad=0 strand=+ repeatMasking=none*

*CACAUAUCCCUUCUAGUUAAGGCCUGUGAAGGCCCAACUCUGUUCUCCUCAGUCAUAGGAUUAAAAGCCCACAAAAUAAAACAAUAAAUAGCAGUUACUUGUUCUGACCACACACCACCAACCCACGCCUACUAAGCUUACCUGUUUUUUGGUAUGUUCAUUGUAUUCUCCAGGAAGAUUGUGUGCGCUUUUAAUUAAGG*

*>hg19_ct_UserTrack_3545_(null) range=chr3:25654002-25654201 5'pad=0 3'pad=0 strand=+ repeatMasking=none*

*AUACCUUCUUCUUCUUCAGCAACUUUUUGCUGGCAUCUGCCUUCAUAGCUGUAAUUUCAGGAAUUAUUCUUCUGCCAUAAGGUGAGGGCAUUGUCUCUUCCAACUGGAGUUUCUUCACCUUAGGUUUGCCAACUUUACCUUUAAUUGCUUUUCCAGACAUUCCAGCCAGAACAUCUUCUCGUUCUUGAGAUUCCACUUUC*

*>hg19_ct_UserTrack_3545_(null) range=chr10:102743169-102743368 5'pad=0 3'pad=0 strand=+ repeatMasking=none*

*AGCAUGGGCCUGAGCGAUGGGCAGGGUGGCUACCGUGUGGGCGUGGACGGGCUGCUGGUUACAGAUGCACAGCCUGAGCACAGUGGCAACUAUGGCUGCUAUGCCGAGGAAAAUGGCCUCCGCACCCUGCUGGCCUCCUAUAGUCUCACAGUCCGGCCAGCCACUCCUGCCCCAGCUCCAAAAGCCCCUGCCACACCUGG*

*>hg19_ct_UserTrack_3545_(null) range=chr20:31996433-31996632 5'pad=0 3'pad=0 strand=+ repeatMasking=none*

*GGGCCCUGGCCAGCCUGGCCUCCGAGAGUGCACACCCCCUCCUCCCCAGGGUGCAGAGGCAGCCCCUCACUCACGAUCUCGCCUUCAGCACCUCCAAAAUCCAGGAAAAGGAGACUGGCACCGUCAUCUGAAGACAUCUGCAGCUUCUCGAAGGGCUGUCGCAGGAGCACAGCUCGGGCUGCACCUGGCUCAGCCGCCCA*

*>hg19_ct_UserTrack_3545_(null) range=chr12:6334493-6334692 5'pad=0 3'pad=0 strand=+ repeatMasking=none*

*GUGGUCACAAAGUCCUUUGCAAGUCUCACCCUUAAGCGCGUGGGGACUGUGUUGUUGCUGGCAGCCAGAAUUAAUGCUGAUGUCCUGUAUGUCUUGCAGCUUGCCGGGAUUGCUGUCCUUGCCAUUGGACUAUGGCUCCGAUUCGACUCUCAGACCAAGAGCAUCUUCGAGCAAGAAACUAAUAAUAAUAAUUCCAGCUU*

*>hg19_ct_UserTrack_3545_(null) range=chr6:44360376-44360575 5'pad=0 3'pad=0 strand=+ repeatMasking=none*

*AAUAUAUAAAAUGAUCUAUGUUUAAUAGGUAUGAAUGGCUGGAUCCAAGCAUUAAGAAGACAGAAUGGUCCAGAGAAGAAGAGGAAAAACUCUUGCACUUGGCCAAGUUGAUGCCAACUCAGUGGAGGACCAUUGCUCCAAUCAUUGGAAGAACAGCGGCCCAGUGCUUAGAACACUAUGAAUUUCUUCUGUAAGUGAGU*

*>hg19_ct_UserTrack_3545_(null) range=chr1:226923986-226924185 5'pad=0 3'pad=0 strand=+ repeatMasking=none*

*UUUUGCCCACUUCAGGCUCCCCAGAGCCCGGCAUGCCACAGGGCAGAUAUCCUUUCCCCAUCUUCCCAGGGGGUUCUCCAUCGCGGGGCCCGCCCCUUUCUGGGGCUGGGCUUGUCUCACUGCCCAGAAACUGCCCCUGCCUCUCCACCAGGGCCUCUGGGGGCUGCAGGUCCUCAAGCUCACGGGCUCUCCCAGACGGC*

*>hg19_ct_UserTrack_3545_(null) range=chr17:41846857-41847056 5'pad=0 3'pad=0 strand=+ repeatMasking=none*

*AGACAAGUGCCUUGUGAUGCUGGGAGCAGGGUACAGUGUGUUUCCUAAACAUGGCAGCUCGGGACACCUUUGCCCACGGCCUCCCCCACGGACCUCUCGAGCAGAGGUGGUGGGGGUGCCCUAGGGUUUCAACUUCCCCUCCUUGGCUAGUCUGUCAUUGAGCUGGCAGAGCUGGGCCAGGAAGCCAUCGUUGGGGCCGA*

*>hg19_ct_UserTrack_3545_(null) range=chr3:197238639-197238838 5'pad=0 3'pad=0 strand=+ repeatMasking=none*

*AACCAGUUAUGGGUCUUCCUGGCAUGGUGGAUAAUCCACACGUGGAUAAUCAAGAGUUGACUAUAUGGGUUCCUCCCUCCCCUCCCCUUCCACCAGGGAUCCCUGACAGAGGCCACAGCGAGACUCUUCAGCGGAUGUAGAUCAUGUCGGAGAUGGCUCCAGGCAAGUGGGUCAUGAUCUGCAUUCGCAGCCACCAGUAG*

*>hg19_ct_UserTrack_3545_(null) range=chr22:50644724-50644923 5'pad=0 3'pad=0 strand=+ repeatMasking=none*

*UGAACUCUGUGUUUGGUUUCAGACAGGCCGACGGUCGCAAGGUCCUACGGUCAAGCAUCCGGGAGUUUCUAUGCAGCGAAGCCAUGUUCCACCUGGGAGUCCCCACCACACGGGCCGGCGCCUGCGUCACGUCCGAGUCCACGGUGGUGCGCGACGUGUUCUAUGAUGGUAAUCCCAAAUAUGAACAAUGCACGGUUGUG*

*>hg19_ct_UserTrack_3545_(null) range=chr1:8923900-8924099 5'pad=0 3'pad=0 strand=+ repeatMasking=none*

*AUUCACAAGCCGUAGCUGCGGGAAAGCUGCUCGCCUGGGAAGACACUUACGCCUGAAGAGACUCGGUCACGGAGCCAAUCUGGUUGACUUUGAGCAGGAGGCAGUUGCAGGACUUCUCGUUCACGGCCUUGGCGAUCCUCUUUGGGUUGGUCACUGUGAGAUCAUCCCCCACUACCUGGAUUCCUGCACUGGCUGUGAAC*

*>hg19_ct_UserTrack_3545_(null) range=chr7:36588175-36588374 5'pad=0 3'pad=0 strand=+ repeatMasking=none*

*CAGAGCCAAAGAAUCUUGAAAAUACUUUUUCUCAAUAACUCACCCACUGCAGACAUCAUUUCCAAUCAUGGCAUAUAUAACGAUGGCGGGAUAGUCCAACACCUUGUUUCUAGACAAGCUGAGAAGGCAUAGAUGGAAUCUGAAUGUCAGAUACUUGGAAACCUAGCUUUGUAGCCAGUGACACAGGCUGGGCCUUUGAA*

*>hg19_ct_UserTrack_3545_(null) range=chr5:140012023-140012222 5'pad=0 3'pad=0 strand=+ repeatMasking=none*

*CGAGUGUGCUUGGGCAAUGCUCAGUACCUUGAGGCCUGGCUUGAGCCACUGCUGCAGCUCGGCGAGCCAAGAACGCCCUGUCGCCCACGACACGUUGCGUAGGCGCAAGCUGGAAAGUGCAAGUCCUGUGGCUUCCAGAGGCAGCGGAGGCAUGGUGCCGGUUAUCUUUAGGUCCUCGAGCGUCAGUUCCUUGAGGCGGG*

*>hg19_ct_UserTrack_3545_(null) range=chr6:57467076-57467275 5'pad=0 3'pad=0 strand=+ repeatMasking=none*

*UCAGUUUGAUAAAGGUUACUCUUAGAACAUCCGUCACAGCUUUGGAAAGGAAGGCAAGAGGACAGACUAUACACCUUUCAGUUGCCUGAAGAUUAUUCUAUCCAAUCCACCAAGCCAAGGGGAUUAUCAUGGUAAGUGCCUCCACUGGAUAUGUUGGCCAAGUACAGAAAUCCAAGAGCUCUGUUGGAAACACUGAAACA*

*>hg19_ct_UserTrack_3545_(null) range=chr7:86468804-86469003 5'pad=0 3'pad=0 strand=+ repeatMasking=none*

*CCUGCUGACCAAGACAAACUGCAUUGCCCGCAUCUUCGAUGGGGUCAAGAAUGGCGCUCAGAGGCCAAAAUUCAUCAGCCCCAGUUCUCAGGUUUUCAUCUGCCUGGGUCUGAUCCUGGUGCAAAUUGUGAUGGUGUCUGUGUGGCUCAUCCUGGAGGCCCCAGGCACCAGGAGGUAUACCCUUGCAGAGAAGCGGGAAA*

*>hg19_ct_UserTrack_3545_(null) range=chr21:47783529-47783728 5'pad=0 3'pad=0 strand=+ repeatMasking=none*

*UGAGGAGAAGUUAACAUUGAUGCUACUUGAACUGAGAGAAAAGGCUGAAUCCGAGAAACAGACCAUCAUAAACAAGUUUGAGCUUCGAGAAGCUGAAAUGAGGCAGCUUCAGGACCAACAGGCAGCCCAGAUCCUGGAUCUGGAGAGGUCCUUGACGGAGCAGCAGGGCCGCCUGCAGCAGCUGGAACAGGACCUCACUU*

*>hg19_ct_UserTrack_3545_(null) range=chr1:161070384-161070583 5'pad=0 3'pad=0 strand=+ repeatMasking=none*

*UUACAAUAGCAGAGAAAAUAAUAAUAAUAACAAUAAAGAGAAAUUAGAAGUGGGAGUCAGGGUAGAAAAAAAUGCAAAGGCCUUGGUCCCUAGGAGACCAACACUCCAGCUGAGCUGGCCUUAGCCCCAGCCCCUUCUGAGUUUUCCUUGGCUGCUGGCUUCUCAUCUUCUCCCAUGAGACGAAUGUUGUGCUUUUCCCG*

*>hg19_ct_UserTrack_3545_(null) range=chrX:48826107-48826306 5'pad=0 3'pad=0 strand=+ repeatMasking=none*

*ACAUAGUAGAAAACGAGGGCUGCGGUGCUCGUGUGUGGAUUCUCGAAGGCCCGCCAGAGCCGCUGCCGCAGGGAGCUGCCUGCUGGCAGGGCUGGGCCGUCCCCGGCCUGCUCUGCCUCCUCAUCCUCUGCCAGGCGCUCGGCAUUCUCCUUCUUUCGGUCCCGAUACUCUUCAAGGCAGCAGUCACCGACUAGCUCGGG*

*>hg19_ct_UserTrack_3545_(null) range=chr10:17086992-17087191 5'pad=0 3'pad=0 strand=+ repeatMasking=none*

*AACAUUAUACAUACAGCCAGGUAAUCUAAAGUGCAGUUUGGAUGAUGCUCCAAGUGAAAGUCUUUGAAUUCCAGUUCAAAUGCGCUGCCGUGGCUAGAUUUCAACCACCAGUAGCAUUCAGAGCUGUGGUAAUAGGGCAUCGGGUAGUUGGGAGAUAUGAACGUGCCGCUUGAAGUGGUGAGAUUACCCCCGCAACCUAC*

*>hg19_ct_UserTrack_3545_(null) range=chr16:72132741-72132940 5'pad=0 3'pad=0 strand=+ repeatMasking=none*

*GCCUCGGCAGGGAGAGCAGAUGGUGGCCAGGAGGACGUGGCUUAGCUCAGUGACAGUUUUGUGAUGCUGGUGCUGCUGUUCCCAGGUCUGUGAGGGGCAAGUACUCGGAUGACACGCCUCUGCCAACUCCCUCCUACAAAUAUAACGAGUGGGCCGAUGACAGAAGACACUUGGGGUCCACCCCGCGUCUGUCCAGGGGC*

*>hg19_ct_UserTrack_3545_(null) range=chr17:75202689-75202888 5'pad=0 3'pad=0 strand=+ repeatMasking=none*

*GAAGUAUAUAAACUUGAAUGUCUUCCCCCCACCCUCCCACUCAGAAUACCACAUUGUCAUUUUAAUCUUGGACACUCAGGCAGCAGAGAAAUAUGACUGCAUGGUCUUCUCCUCCUCACAGGUUAGUCCGUUCAUUGAUGACAACACCAGAAGGAAGUUCCUCAUUUAUGCAGGAAAUGACUACCAGGGUCCUGGAGGCC*

*>hg19_ct_UserTrack_3545_(null) range=chr20:44671687-44671886 5'pad=0 3'pad=0 strand=+ repeatMasking=none*

*UGGGGUUGAUCUUGACCCUGACUUUUUGCCCCCUCCCACCUCCUGGUCCCAGAGCUCUGGUCCCCUUAUGGCUGGCCUCCCCCUGAGCAUUCUGUCUCCCCACAGAGAACCUCUGGAGCUCCUACCUGACCAAGGGCGUGAUUGUGGAGAGGAGUGGGAUGACCUCGGUGGGCCUGGCCGAUGGCACUCCUAUCGACAUG*

*>hg19_ct_UserTrack_3545_(null) range=chr7:103014863-103015062 5'pad=0 3'pad=0 strand=+ repeatMasking=none*

*CAGGAGUGGCAUUGGGCUCCAAGUCCUCCUGGGAAGGGGGAGCCGAGGCUUCCUGUUCAGCAAGUGCCUCUCUAAGUUGGCUGCCUAAAACUGCAUCAUGAAUGCUGUGGAACAGCAGCUCCCAUAGGGCAGGAUUUUCAAAAAAUCUAUUCCGAGUGAGGUCAUUCACAACUUGUGCUAUUAAAAGAAGAAAAGAAAAU*

*>hg19_ct_UserTrack_3545_(null) range=chr9:101785506-101785705 5'pad=0 3'pad=0 strand=+ repeatMasking=none*

*AAAACGGGGAGCAGAAGUUAUCGGGCUAUAGGAUAUAGGAAACAUUUCCCCAUUUUGGAGAAAACUCAGUGACGUGGCUGUCCUUGGAGUCUGGCAGUUAAAUGCAAGUAGUGGAAAUUUCUUCUCUUUCCAGGCAGGAGCAGAAGCAGAGGGCUCUGGCCUAGGCUGGGGCUCGGACGUCGGCUCUGGCUCUGGUGACC*

*>hg19_ct_UserTrack_3545_(null) range=chr20:21492947-21493146 5'pad=0 3'pad=0 strand=+ repeatMasking=none*

*GCCGCUCCAGCUCGUAGGUCUGCGCCUUGGAGAAAAGCACUCGCCGCUUUCGCUUCUUGCCGGCGUCCCCCCCGCCGCCCGGGGUCUCCUUGUCAUUGUCCGGUGACUCGUCGGCCGAGGGCUCCGGGGACUUGGAGCUUGAGUCCUGAGGGGGCGCCCCGGCAGCCAGACCGUGCACUGGGGGGAGGGGGAGAGAGAAG*

*>hg19_ct_UserTrack_3545_(null) range=chr9:35704649-35704848 5'pad=0 3'pad=0 strand=+ repeatMasking=none*

*GGGAUGAGCACCGUCACCUUCUCAGAGACUCUCCGGGCACACUCUAUGAGCUCCUUCUUGGUGUAGGCAUCACUGGGGCUGCACUGCAGGGCGCCUGCCUUGGUGACCAGAGCGGCACAGCCAUGGCCCAGCUCCUGUACCCGGUGUUUGAUAUGGGAACCUAUCUGUGAGCCAAGGGAAAGACAGAUGGAUUUUACAAG*

*>hg19_ct_UserTrack_3545_(null) range=chr7:75189074-75189273 5'pad=0 3'pad=0 strand=+ repeatMasking=none*

*UCCUCCCGCUGCCUCCUGAGCUCGUCCAGUUCUGCCCGCAGGAAUUCACAGUCGUCGGCCGCCUGCUGCCGCAGGUGCUGCUGCUCGGCCAGAUCUGCUUCCAGCUCGCUGACGUGGCCCUUCAGCUGCAGCACAACCCGCUGGCUCUGUGGGGGGACUCCGGUCAUGAGGCCAACCGCCCACUGCCACGGGUCACGGGC*

*>hg19_ct_UserTrack_3545_(null) range=chr20:62128913-62129112 5'pad=0 3'pad=0 strand=+ repeatMasking=none*

*CCACCACGGGAGUUGGGGGUUCCUUCUCAGGGGGCCAAGACCAUAGCCUGGGGAGCUCACCUCAGCCGCCUCCUUCUCGAACUUCUCAAUGGUCCUUUUGUCAAUACCUCCGCAUUUGUAGAUGAGGUGGCCCGUGGUGGUGGACUUUCCGGAGUCCACGUGGCCGAUGACCACGAUGUUGAUGUGGGUCUUCUCCUUGC*

*>hg19_ct_UserTrack_3545_(null) range=chr11:102401268-102401467 5'pad=0 3'pad=0 strand=+ repeatMasking=none*

*UUUCUUAAUGCAGAUGGCAAAAGAAUGGAACCCUAAGUAAGUGGGCUGUGACAUACCUGAGCCUGUUCCCACUGUAGCUCACUCAUGCCUCCCGCCUCCUGAGGCAGCGGCAGGGCCAGGCUGCCAGGCAGCAGGCACACAGCACACAGCACGGUGAGUCGCAUAGCUGCCGUCCAGAGACAAUUGUUCUUGGACCUAUG*

*>hg19_ct_UserTrack_3545_(null) range=chr8:22974145-22974344 5'pad=0 3'pad=0 strand=+ repeatMasking=none*

*UGUACCCAGGUGCCCUAGUGGGGAAGUCCAAGUCAGUAAUUGUACGUCCUGGGAUGAUAUCCAGUGUGUUGAAGAAUUUGGUGCCAAUGCCACUGUGGAAACCCCAGCUGCUGAAGAGACAAUGAACACCAGCCCGGGGACUCCUGCCCCAGCUGCUGAAGAGACAAUGAACACCAGCCCGGGGACUCCUGCCCCAGCUG*

*>hg19_ct_UserTrack_3545_(null) range=chr2:20212216-20212415 5'pad=0 3'pad=0 strand=+ repeatMasking=none*

*GCCGUCGGGAGCCGCAGGAGAGGGGCGGCGUCCAGGGCUGCCCCCGGGACCUCGGGUCUCCAGCCUCCGGAAGCCCGGGCGGGCCACGGGGUCGGGGGCGGCGGAGGGCAGCAGCAGCAGCGGCCAGAGCAGCAGGAGGAGUCCCGGGAGGCGGCGCGCGGGGGCCGGGCGCGGCAUGGUGGGCUCCGUGGGCCUGGUGG*

*>hg19_ct_UserTrack_3545_(null) range=chr8:145662170-145662369 5'pad=0 3'pad=0 strand=+ repeatMasking=none*

*CCGUUCAAGCAGCAGCUCAGCCACCUCGAAGUGGCCACAGUUGAGGGCAUCGUGGAGGGGGGUGAUGCCUUCGCAGCCCUGGCCACCUGGGUCGUCCACUGCGGCCCCGUGGUCCAGCAGGAAGCGGACAAUUUCUGCAGACCAGGAGACGUAAGCCCAGCUCCCGAUGCCCCGCCAGGACUGACUCUUCGCCCCACGUG*

*>hg19_ct_UserTrack_3545_(null) range=chr15:64221683-64221882 5'pad=0 3'pad=0 strand=+ repeatMasking=none*

*CUCUCUCAUUCCUUCUGGUUCCCCCAGAUCUAAGCUGAGCCAGAGCCCCUGGAUCAGGGAUACUCACAGGAUGUAGGUGAUGACGCCUAUGCUCCUGCAAAAUAAAGAACAGUCAAGUCAGGGCCACCUGGGUCCCAUCCCCGACCAGCAAGGGACACCCUCAUGGAUCUAAGGGGAAGAGGUCACUUGCCUCCUCCACA*

*>hg19_ct_UserTrack_3545_(null) range=chr20:58490493-58490692 5'pad=0 3'pad=0 strand=+ repeatMasking=none*

*AAAAACCAUAUAAGUACAGAUUAUUACAUGAGAAUUAACAUUUCUUGGUUAGAGAGUAUUUUCCGGGCAUCUUGAGGCAUUUUGUCAAGCAUAGCAUUCAUUUUUUUUAUAAUCUAGAAAAGAAAAAAAGAUAAUGCAUUAACUUUACAUUCAGUGAAACACUAUUAAGAGAAAUAUUCUAGGCACAUAAACUUAGAAGC*

*>hg19_ct_UserTrack_3545_(null) range=chr3:8809588-8809787 5'pad=0 3'pad=0 strand=+ repeatMasking=none*

*GCGGCAGCACCUGAAACACUGCCACCACCAGGUCGGCGAUGCUUAGGUGCUUCAUGAAGAAGAAGAGGCGCGAGUGCUUCUGGCGUGUGGUGCGCAGCGCCAGCAGCACACACGCGUUCCCGCUCAGCGCCAGGAGCAGGAUGAGACACAGCACCGCCACCUCCACGCGCGCCAGGGCCUCGUUGCGCCGCGGGGGUCCG*

*>hg19_ct_UserTrack_3545_(null) range=chr16:83813537-83813736 5'pad=0 3'pad=0 strand=+ repeatMasking=none*

*CCAGUUGCAUACCAACAUCUUCCCUUUUUCCCACAGGCAACCCUCCCGCUACGGGCACUGGGACUUUGCUGAUAACCCUGGAGGACGUGAAUGACAAUGCCCCGUUCAUUUACCCCACAGUAGCUGAAGUCUGUGAUGAUGCCAAAAACCUCAGUGUAGUCAUUUUGGGAGCAUCAGAUAAGGAUCUUCACCCGAAUACA*

*>hg19_ct_UserTrack_3545_(null) range=chr22:19419926-19420125 5'pad=0 3'pad=0 strand=+ repeatMasking=none*

*AGGCGGGGCUGCGUCGGGGGCGGGGACAGGCGCGUCCCAGCCCACGCAGCCGGAACGCCUGAGCGCUGGGCGCCUGGUCUGCCGGACCUCAGCAGGGGGCACCGCGGGCCGGACUGUGGAGGGGCGCACGCCCGGAAGCGGCGAGGGUAGCCAUGACGGCCUCCGUGCUGCGAAGUAUCUCGCUAGCCCUGCGCCCGACU*

*>hg19_ct_UserTrack_3545_(null) range=chrX:114868225-114868424 5'pad=0 3'pad=0 strand=+ repeatMasking=none*

*AAAUAUUUGUAAAUAUUUACAGCCACUUUAUAAAAGGAAAUCAGCUCUUUUUUUUUACAUCAGCCUUUUUUUAAAUUUUUGUUUCAGUAAAAUGAUUAACCUUUCAGUUCCUGAUACCAUUGAUGAAAGAGCAAUCAACAAGAAGAAACUUACACCCUUCAUCAUUCAGGUAUGCAUUGUUCUCCCCUCCCUUUAACAUG*

*>hg19_ct_UserTrack_3545_(null) range=chr11:89531539-89531738 5'pad=0 3'pad=0 strand=+ repeatMasking=none*

*CCAUCUAUCUUCUCAUUCUGAUUCUUCUCUUUCCGAUACAUAUUACAGACACCAAAAGCCCAAUUCCAGGAGUCCCCUACAUGGACCUCCCAGUAAUAUUUGCCCGAGGUGAAAGUCUGAACACCCCAUGCAAGAAAACUUCUAGGUGUUGCAGUGAAAUAGGGUACAUCUUGAUGGUCACAUCCAAUACACAUGCUUCU*

*>hg19_ct_UserTrack_3545_(null) range=chr22:46930890-46931089 5'pad=0 3'pad=0 strand=+ repeatMasking=none*

*GCCUGUGAGCUGGUAGGUAAUCACACUGUUGGCGUCACGGUCGCGGGCCUGCAGGGUCAGCACGCUGCUCCCCACGGCCGCAUCCUCAUUCAGACGAAGCUCGUAGGUGGGCUGCGUGAACACCGGGUCGUUGUCAUUCACGUCCAGCACCGUGAUGGACACGCUGGUGGAGGAGCUCAUGGGGGGCGAGCCGUGGUCCA*

*>hg19_ct_UserTrack_3545_(null) range=chr9:19376361-19376560 5'pad=0 3'pad=0 strand=+ repeatMasking=none*

*UGUUCCUGGCGCUUCUCCUUAGCCUCCUAAACAAAACAAAACAGCAAACAGUUAAGGCCUUUCUGGGUUAAAGACGCAAAUCCAAAGCCAGGUCGAACUCCUCCCACCCCCUCAAAUCAUCUUAGACUAACCUUCAUUCUCUUGGCCAAAAGUUUAGCAUAUUCUGCAGCCUCUUCUUUAUUUUUCUUGGUACGCUGCUU*

*>hg19_ct_UserTrack_3545_(null) range=chr10:48387916-48388115 5'pad=0 3'pad=0 strand=+ repeatMasking=none*

*CAGCCCCCAGGAUCUCGGCUAGGGCCACUUCUGAGGUCACCCUGGAGUAGCGGCUCUGCAGACCGCUCAGUUUGGUGGCCAUCUUGGCCCCCAGCUCGGCAGAGGCAUAGUUAUCAGCCACCAGCUUCCCGGCCGUCUGCAGCACCGUGGGCACCUUGGCACGCAGAGCCACUAUGUCCUGGGCUAUGGAAAGGGCUUCG*

*>hg19_ct_UserTrack_3545_(null) range=chr3:13659490-13659689 5'pad=0 3'pad=0 strand=+ repeatMasking=none*

*ACCUGCACUAGUUUCCCAAAGCUGGUUGUCUCAGCCACCUACUCUGACCAAGCUGGCUCUGUCUUGAGCUCUAACCCCUGUCCUAGUUUCAGAGGCAGAGAUGGCGGGCCGAGAGGCCCUGUCACUGGGCACAGAGGCCGAGCUGCCGAACAGCCUGCCGGGCGAUGACCAGGAUGAGUGCCUUCUCCUCCCGGGAGAGC*

*>hg19_ct_UserTrack_3545_(null) range=chr3:46944027-46944226 5'pad=0 3'pad=0 strand=+ repeatMasking=none*

*AAUCCACGCUGGUGCUCAUGCCCCUCUUUGGCGUCCACUACAUUGUCUUCAUGGCCACACCAUACACCGAGGUCUCAGGGACGCUCUGGCAAGUCCAGAUGCACUAUGAGAUGCUCUUCAACUCCUUCCAGGUGCGCAGUGCUGGCCCGGGCCUGGCUGAGGGUGGGAGGGGUUCCGGGGGCAGGCCUGAUUCGAGACAC*

*>hg19_ct_UserTrack_3545_(null) range=chr1:108319778-108319977 5'pad=0 3'pad=0 strand=+ repeatMasking=none*

*AACAUCCACAGUUAAAACCCACAAUGGACACAUAAAUUGGCAGUGGCUAAAUUUUGCUUGAAAUUUUCAACAUACUCUAUUAAAUCAGGAAGGCCUUUGUAGAUGUCUUCAUCAUUAAUGCUUUCUUCUGUUGGGAAGGGCCUAGGAAGAGGAGAAAAAACAAAAACAAAAAAACAAACCCAUGAGUGAACGAGCAACAA*

*>hg19_ct_UserTrack_3545_(null) range=chr11:68527656-68527855 5'pad=0 3'pad=0 strand=+ repeatMasking=none*

*CGCCCCACUGCGCCUCGCCCAGCCCCGCCGCACUCACCGGUCCAAAGCCCCCUCCGCUGGACACGUACUCUGGGUUAUUCUCCAAGUCAAACAGCUCCACUUGCUGCUGAGGGGUCUGGCUUGUUGAUAAUCUCCAAGGCUCAGAUAAAACCUAUUGAGUGAAACAGGGAAAUGUUUCCUAAUCCCCUCCUCUACCGUCC*

*>hg19_ct_UserTrack_3545_(null) range=chr8:146156558-146156757 5'pad=0 3'pad=0 strand=+ repeatMasking=none*

*GGACUCGCUGGUGAAGGAUGAGGUUGGAGCUGCGACCAAAGGUCUUCCCACACUCGUGGCAGGCGUAGGGCUUGUCGCCUGUGUGCACGCCCUGGUGCUGAAUGAGGGCUGAGCUGUGGCUGAAGGCCUUCCCACAGACACUGCAUCUGUACGGCUUCUCUCCCGUGUGGAUGAUCUGGUGCUUUCGGAGCACUGAGCUA*

*>hg19_ct_UserTrack_3545_(null) range=chr4:24538648-24538847 5'pad=0 3'pad=0 strand=+ repeatMasking=none*

*GUGUCUCCAAUCAAAUUUACACUUACUUUGUUUAAAAGCAUGGUAGACGUUCAGCAGUGUCAGAUGAUCUCCAUCUAUGUGGGCAAAUCUCAUCUUGGCCUCAUCUGCGGCUUUCUUGGCCUCCGUGGGGCGAACAAAACACUGUGGGACUAAACAAGGUUGGUAUGGGCCCAACACAAACGCCCAUAUCGAUGAGUCAC*

*>hg19_ct_UserTrack_3545_(null) range=chr11:121427929-121428128 5'pad=0 3'pad=0 strand=+ repeatMasking=none*

*UGUCUGUAGCAGAAGCUGAGUAGCCAUCUUUGGCAAUGGGGGUCUUUAAGGAGCUCCGAUCCAUCUCAGCCUCUUUUCCCCCUGUUUUUGUCAGGUAGCUAAUCCAGAUGGCGACUUCCGACUCACAAUCGUCAAUUCCUCUGUGCUUGAUCGUCCCAGGGCUCUGGUCCUCGUGCCCCAAGAGGGGUAAGUGUUGCCCC*

*>hg19_ct_UserTrack_3545_(null) range=chr6:33333745-33333944 5'pad=0 3'pad=0 strand=+ repeatMasking=none*

*GAUGCCAGCCUCGUCCUCUGGGGCAUCUGGACUCAGCCCCAUCAGGUCAAACCAGGAGGGCAUCACCAUCUUCAUGUUGAGGGUCACAGGGAUCCUAGGCUCAUGGGAACAGAUGUACUUGACGUGAGGGAGCCGAAUGGUGGAGAGGGCGUCAGCCCAGCUGUGCCCUGUGUCUCCAAGUCCAUGUAAAAAAAUAACCA*

*>hg19_ct_UserTrack_3545_(null) range=chr19:15760669-15760868 5'pad=0 3'pad=0 strand=+ repeatMasking=none*

*CUGGGGCUUCAGGUGUAUUAAAUUGUUGCCUCCCUUUCUGCCCUUAUCCUGCAGGAAGCCCAGUGAAUAUAUUGCCGCCAUCUUGGAGCUCAGUGCCCUUGUGACAAAAAGACACCAGCAGAUCCUCCUGUACAUAGACUUCCUGUAUUAUCUCACCCCUGAUGGGCAGCGUUUCCGCAGGGCCUGCCGCCUGGUGCACG*

*>hg19_ct_UserTrack_3545_(null) range=chr1:204429644-204429843 5'pad=0 3'pad=0 strand=+ repeatMasking=none*

*GCCCUGGGCAACCCUCCACCACCCUCACACGAACCGUCCGGGCCAGGUCACUGCGCACAACCUUCUGCUCCAUCAGCUGUAGCCGAAUGUCAAUGUCAAACUUGCGGCAGUAUUGGAUGUACUCAUGACUGCCCAAGGCAUGCUUGCUGGUAGGGUAGAGGGACAAGACCAUUAGCAUCCUGGGGACUCAGUGGCAAGGG*

*>hg19_ct_UserTrack_3545_(null) range=chr12:52715001-52715200 5'pad=0 3'pad=0 strand=+ repeatMasking=none*

*AUGCCGCGGUAGGGGGCGGCGGUGAUGCAGCAGCGGCUUGGCCGGGGCCCGCAGGCAGAGACACAGCUGAAGUUUCCAGGGCGGAACCCACAGCCUAUGGAGUUGAAGCCACAGGUCAUGACGGAGGUUAGGAGGUGUCUGAACAGUGGAGUAGAUGGCAGAGGAUGGAACCAAGGGCCUGUGCUCCCUGGCUGAUGGCA*

*>hg19_ct_UserTrack_3545_(null) range=chr14:20849986-20850185 5'pad=0 3'pad=0 strand=+ repeatMasking=none*

*CAGUUGUUGAAGCUAUACAGAGGGCCCCGGCUCAAAGAAGGGAAGGCACAGAGAGGUCAGCGGGAGCUCUGCUGGGGCACCUGUACCUGCGCAGACUCUGGACGAGGCAGGCAAACGGGCCCAUGGGGUAGGGGUCUCCACUGUUACCAGCAGCCACUGCUUCUUCCCAGCUCUUAGUCCCCUUCGGUAGUGUCCGCCAC*

*>hg19_ct_UserTrack_3545_(null) range=chr16:50342452-50342651 5'pad=0 3'pad=0 strand=+ repeatMasking=none*

*CCUCGGGGACAAUUUCCUGAGUGCCCUGGAGGCUUCUCCCGAGGAUACCCCUCCCCAGGCUGCCAGCGACCAGCCCUCCUUGCCCAGGCUGUUGGCUCUGGGUGACUUGACCCUGUUACCCCACAGAGGUGCUGCCCAGCUCGGGGGACGCUCUGCACUAUCUCUGAGAGGGUGGAGACACAGCCCCUGCUGAGGCUGAC*

*>hg19_ct_UserTrack_3545_(null) range=chr19:36216068-36216267 5'pad=0 3'pad=0 strand=+ repeatMasking=none*

*GUCUGCCUUGUAUGCCUGGCGGCCCUCUGAUCCUGCAUCCUCUCUUCCCCCAGGAGGAUUGUGAUUUAGAGAACGUGUGGCUGAUGGGGGGCCUGAGUGUGCUCACCUCUGUGCCAGGGGGCCCCCCGAUGGUGUGCUUGCUGUGUGCCAGCAAAGGACUCCACGAGGUUAGAUCUCUGCCUUUCUUCACAGACCCCCAG*

*>hg19_ct_UserTrack_3545_(null) range=chr7:150439265-150439464 5'pad=0 3'pad=0 strand=+ repeatMasking=none*

*CUACAGGUAGAUCAGAAGAUAACUUGUCUGCAACACCACCGGCAUUGAGGAUUAUCCUAGUGGGCAAAACAGGCUGCGGGAAAAGUGCCACAGGGAACAGCAUCCUUGGCCAGCCCGUGUUUGAGUCCAAGCUGAGGGCCCAGUCAGUGACCAGGACGUGCCAGGUGAAAACAGGAACAUGGAACGGGAGGAAAGUCCUG*

*>hg19_ct_UserTrack_3545_(null) range=chr2:98262393-98262592 5'pad=0 3'pad=0 strand=+ repeatMasking=none*

*GCGCAAUUUCCGCCCCCGCCACAGUCUUGGGAGUGGGCGGGCUCGAGGGCUGGGAGGAAGCCGAAAGCUGACCGAGAGGAGAAAGAAGCCCGCCCCCGGAAGUCCCUCCUGUCUCUGCAGCUUGUUCCCGGAAGUUUUGCUGCUAGUCGCGGACGCAAUGGCUUCAAGGUUACUUCGCGGAGCUGGAACGCUGGCCGCGC*

*>hg19_ct_UserTrack_3545_(null) range=chr19:3979210-3979409 5'pad=0 3'pad=0 strand=+ repeatMasking=none*

*UGAGACCAAGACCGAGAUCAAGUCUAGGCGUCUGCAGAGCCUAGCUCAGCUCAGCUUUAAAGCAGAGGGCAGGUGUCCGGGGUGGGGCGUGGGGAAGGCUGGUCACUGGCGCCUCACCUUGAUGGGGAUGCAGGCGUGGUCCUCCUCCAGGUCCUUCAGGCAGAUCUCCAGGUGCAGCUCGCCGGCGCCCGCGAUGAUAU*

*>hg19_ct_UserTrack_3545_(null) range=chr16:71780466-71780665 5'pad=0 3'pad=0 strand=+ repeatMasking=none*

*GCAUUGCUUAGUUUCUUUCGCUCACCUCAUGUGGUCAUAUUUCUUGAAAAGUGCAUUAUAUUCUACUGCCCUCUGCUGGAGUUCCACAUCAAUGCUGCUUCCGUAGAUGGAAACCACUUUCUUAAUUCGGCUAUAGAUAAAAUGACAAACGAAAUAAAAUACCCAAAAGAAAAUUCACAAAUAAGCAGAGAUAAUGUUUA*

*>hg19_ct_UserTrack_3545_(null) range=chr10:96534752-96534951 5'pad=0 3'pad=0 strand=+ repeatMasking=none*

*UCAGGCUUAGUAAAUGGACAAAACAGUGACUUCAUUUGCUGUUAACUGUAUCUCCUUUUCUAGCUCUCAAAAAUCUAUGGCCCUGUGUUCACUCUGUAUUUUGGCCUGGAACGCAUGGUGGUGCUGCAUGGAUAUGAAGUGGUGAAGGAAGCCCUGAUUGAUCUUGGAGAGGAGUUUUCUGGAAGAGGCCAUUUCCCACU*

*>hg19_ct_UserTrack_3545_(null) range=chr1:64643315-64643514 5'pad=0 3'pad=0 strand=+ repeatMasking=none*

*CACCACCCCAAUAUUGUCUGCCUUCUAGGUGCCGUCACUCAGGAACAACCUGUGUGCAUGCUUUUUGAGUAUAUUAAUCAGGGGGAUCUCCAUGAGUUCCUCAUCAUGAGAUCCCCACACUCUGAUGUUGGCUGCAGCAGUGAUGAAGAUGGGACUGUGAAAUCCAGCCUGGACCACGGAGAUUUUCUGCACAUUGCAAU*

*>hg19_ct_UserTrack_3545_(null) range=chr19:48925012-48925211 5'pad=0 3'pad=0 strand=+ repeatMasking=none*

*GUGCCCUAAUCACUCCCCAUUCUGCCCCAGUUCCAGAGGCCCCAGGAGCAGUACCCGCCCCUGAAGUUUGGGACCGUGCCCAACGGCUCCACGGAGAAGAACAUCCGCAGCAACUAUCCCGACAUGCACAGCUACAUGGUGCGCUACAACCAGCCCCGCGUAGAGGAAGCGCUCACUCAGCUCAAGGCAGGGUCAGCGCA*

*>hg19_ct_UserTrack_3545_(null) range=chr1:109794386-109794585 5'pad=0 3'pad=0 strand=+ repeatMasking=none*

*UCUCUGUGGCUGCUGAACUGGACCGGGAGGAAGUUGAUUUCUACAGCUUUGGGGUAGAAGCUCGAGACCAUGGCACUCCAGCACUCACUGCCUCGGCCAGUGUCAGCGUGACUGUCCUGGAUGUCAACGACAACAAUCCAACCUUUACCCAACCAGAGUACACAGUGCGGCUCAAUGAGGAUGCAGCUGUGGGCACCAGC*

*>hg19_ct_UserTrack_3545_(null) range=chr12:6755910-6756109 5'pad=0 3'pad=0 strand=+ repeatMasking=none*

*UUGGGUGAACACUCUGGGUGCCUCGGUUUCCCCCCUAGUCUGGCCCUCACCAUCGGGCACUAAGCCGUGGUUUUCAUAUUGGUCCAGCUGGACGAGUGUGGGAUUCCGGCAGCCGUGGGUUGCACGGAGACGGCAGGUAGUCUCUGCCUUCCAGGUUGGAGUCAGCAGUGCGAAGAAGCGUUCGUAUUCGGUAGGAGAGA*

*>hg19_ct_UserTrack_3545_(null) range=chr19:40697963-40698162 5'pad=0 3'pad=0 strand=+ repeatMasking=none*

*GCCAAGGAGUGGGGCACGACCCCCGCGGGGCCCGUCUGGACCGCGGUGUUCGACUACGAGGCGGCGGGCGACGAGGAGCUGACCCUGCGGAGGGGCGAUCGCGUCCAGGUGCUUUCCCAAGACUGUGCGGUGUCCGGCGACGAGGGCUGGUGGACCGGGCAGCUCCCCAGCGGCCGCGUGGGCGUCUUCCCCAGCAACUA*

*>hg19_ct_UserTrack_3545_(null) range=chr10:71877480-71877679 5'pad=0 3'pad=0 strand=+ repeatMasking=none*

*ACCCUCCUGCUGGAAGCAGUCACCAAGCCUCCAAGCCACAUCUCACCAGGCCAGGGCAGGGCAGGGAGGGAGGUGAGGGUGCACGUACUCAGCAGCAGCUGCACGCCCUUCCGGAGGAGGAUCUCCUUCACUUCCUGCCGGACGGAGGGCAGGAGCUCCUUGUCAGCCAGGGCCACUUGGGAGUGAAUGAGAGUGACCUG*

*>hg19_ct_UserTrack_3545_(null) range=chr1:43826766-43826965 5'pad=0 3'pad=0 strand=+ repeatMasking=none*

*CUGACCCCACCUUUAUUCCAUCUAGGCCGUAGCAUGGUGUCCCUGGCAGUCCAAUGUCCUGGCAACAGGAGGGGGCACCAGUGAUCGACACAUUCGCAUCUGGAAUGUGUGCUCUGGGGCCUGUCUGAGUGCCGUGGAUGCCCAUUCCCAGGUAAUCUUUUGCCUGUUCCUGCCUCUCCCACAUGCAUUCUUACCGGGCA*

*>hg19_ct_UserTrack_3545_(null) range=chr1:214556776-214556975 5'pad=0 3'pad=0 strand=+ repeatMasking=none*

*UGGAUGGGCCGAGAGAGGCCCCGUUGACAGCCGGCGGGUCAGUCCGAGACAUGCUGAUGGCCUUGGCUGGCCCAUGCCUCAGCACCCUGGCUCUGGCCAUGGCGGGAGGAAGGCUGGCUUGGUCCUGCCUCAGAGCCCCAUUGCUCACACUCUUCCUUGGACCGGGGUACUCAGGCGGGGGCUUGUUGGGGAUGCGGGCC*

*>hg19_ct_UserTrack_3545_(null) range=chr7:16793465-16793664 5'pad=0 3'pad=0 strand=+ repeatMasking=none*

*GCCCCCCACCCACGUCUGCGUUGCUGCCCCGCCUGGGCCAGGCCCCAAAGGCAAGGACAAAGCAGCUGUCAGGGAACCUCCGCCGGAGUCGAAUUUACGUGCAGCUGCCGGCAACCACAGGUUCCAAGAUGGUUUGCGGGGGCUUCGCGUGUUCCAAGAACUGCCUGUGCGCCCUCAACCUGCUUUACACCGUGAGUAUC*

*>hg19_ct_UserTrack_3545_(null) range=chr22:37538405-37538604 5'pad=0 3'pad=0 strand=+ repeatMasking=none*

*CCUGAGAUCGCAGCCCGGAGCUGGGAGACCACCCUCUCCCUGCACUCUCUCCCUGGGUGGGCUACUCACAUCUGGGGCUCCGAGGAUCAGGUUGCAGGCCCAGGAUGCUUGACUCACGGGGAGCAGCUCACAGGUUUGGUUCCACCGCCUUUCAUGGCAAAAGACCCUCUUUAGAAGAACAGGAAGGACCCACACAGCUG*

*>hg19_ct_UserTrack_3545_(null) range=chr20:33759842-33760041 5'pad=0 3'pad=0 strand=+ repeatMasking=none*

*CUGCCCAGACUCCGCCCCUCCCAGACGGUCCUCACUUCUCUUUUCCCUAGACUGCAGCCAGCGGAGCCCGCAGCCGGCCCGAGCCAGGAACCCAGGUCCGGAGCCUCAACUUCAGGAUGUUGACAACAUUGCUGCCGAUACUGCUGCUGUCUGGCUGGGCCUUUUGUAGCCAAGACGCCUCAGAUGGUGAGUCGGGGGCA*

*>hg19_ct_UserTrack_3545_(null) range=chr2:227916983-227917182 5'pad=0 3'pad=0 strand=+ repeatMasking=none*

*AACUUAUUUGAUAUGGUUAAAAACUCUUAAGUGUUUACCUCUUUCUCCUGGGAAUCCAUCAUCUCCAGGAGGUCCAGGUUCCCCAGGUGUUCCCUUUUGUGAAAUGAUAGCCAUUUCUCCUUCAUCUCCGGGAGGUCCUAUGGCUCCUAUGGAUAUUAAUUAUGCAAGAACAAAAUGAACAGGAACAUCACACACAUAAU*

*>hg19_ct_UserTrack_3545_(null) range=chr14:23896733-23896932 5'pad=0 3'pad=0 strand=+ repeatMasking=none*

*UGGCUCAGAACCUUGGCAGAAUCCCUGCUCCUCUGUACCGGGAGCCUCAGUCCCUACUUACGCGCAUCAGCCCCAGCAUAGUUGGCAAACAGGGUGCUGAGCAGCUUGAGGGAAGACUUCUGAUACAAGCCCACGACAGUCUCAUUGAGAGGAUCCUUGUUCUUCUGCAGCCAGCCAAUGAUGUUGUAGUCCACGAUGCC*

*>hg19_ct_UserTrack_3545_(null) range=chr6:29797547-29797746 5'pad=0 3'pad=0 strand=+ repeatMasking=none*

*GUGAGGGCUGGGGGUCAGAGACCCUCACCUUCACCUCCUUUCCCAGAGCAGUCUUCCCUGCCCACCAUCCCCAUCAUGGGUAUCGUUGCUGGCCUGGUUGUCCUUGCAGCUGUAGUCACUGGAGCUGCGGUCGCUGCUGUGCUGUGGAGAAAGAAGAGCUCAGGUAAGGAAGGGGUGACAAGUGGGGUCUGAGUUUUCUU*

*>hg19_ct_UserTrack_3545_(null) range=chr20:23807047-23807246 5'pad=0 3'pad=0 strand=+ repeatMasking=none*

*CCUCAGGUGGAGGCAGCACCCACCUGCUCCCUGGCUCGUAGCACCCGCAGCAGGCGUCUGUAGUACUCAUCUUCAGUGGCCUUGUUAUACUCGCUGAUGACAAAGUGAAGGGCACGCUGUACCCGCUCAUCAUUGAGGUCUGCAUCAUAGAUGCCACCCUCGAUUAUCCUGUCCUCCUCCUGGGGGCUCCAGGCCAGGGC*

*>hg19_ct_UserTrack_3545_(null) range=chr11:47361118-47361317 5'pad=0 3'pad=0 strand=+ repeatMasking=none*

*UGGGGUGUGCAGCAGGUCCUAGCACUUGGCUGGUUCCACACACCCAUCUUAUAGAUGGGGAGACUGAGGAGGGACCCACAGUACCUGCGUGAUAGCCUUCUGCCAGAUCACAGUGGGAGCAGGGUCCCCAGAGAUAGGGACGUCCAGACGUAGCUUAUUUCCAGCUACAACCACAAUGGUGUCUGGUAUGCGGCCUGGGC*

*>hg19_ct_UserTrack_3545_(null) range=chr8:90949093-90949292 5'pad=0 3'pad=0 strand=+ repeatMasking=none*

*CCAAUUGAGAGGUGCUUAAGUAAUAAAAAUAAACACUUGUGUUAGGUGAAGGGACUAGGUGUCUAUGAGGACAGAAGAAACAAAUUCUUAAUUUCUAUGAACAGAACUAAAUUUUAUAUACAUCUCUCAAAGGUACAUGAGAAAGGUGAAUCAAACUUUACCUAAAAAGAUCAUCAGCAAGAGACUCUUCUUUUGCAUGU*

*>hg19_ct_UserTrack_3545_(null) range=chr9:118982239-118982438 5'pad=0 3'pad=0 strand=+ repeatMasking=none*

*ACGCCCAAUCAAGUCGCCAGAAUGCACUGUUACCUGGACCUGGUCUACCAGGGCUGGCAGCCCUCCAGGAAACCAGCGCCUGUUGCCCUCGCCCCCCAAGUUCUGGGCCACACAACGGACUCUGUGACACUGGAGUGGUUCCCACCUAUAGAUGGCCAUUUCUUUGAAAGGUGAGUGUGCCCUGUGUAGUGUUGAUCCCU*

*>hg19_ct_UserTrack_3545_(null) range=chr19:3960991-3961190 5'pad=0 3'pad=0 strand=+ repeatMasking=none*

*UGCCGGCCCCUCGUACUUGGGAUCUUUGACGAGCAGCCGGCGAAUGAAGUCCUUGGCCAGCUCGCUGGUGUUGCUGAAGUACUCCUCGUCGAAGUCGUAGUUCACGGCUGAGAUGUUGGUGAGCGUCUCCUGCUUGGUCUCGCCCAGGAACGGGGAUGCACCGCUCAGGCUGCGAGACAGGCGUGGGGGCUCAGUGGGGU*

*>hg19_ct_UserTrack_3545_(null) range=chr1:230372271-230372470 5'pad=0 3'pad=0 strand=+ repeatMasking=none*

*CCACAGAAAGAAGAGGUGCCUCUGUGAUGCCCCAAGGAGAUAAUCGGCCCUUAGAUGAUUGAGAAUACUAUGGUUGAAUGCAAAGCCCUUUAUGAACUCCAGGUUCAAAAUGCUCUGCUUCUUUUCUUUUCCUAGCUGAGGACGGGGCUCUCUUGGGGAAAAUUGAGAAAGUGCGAGUUCUUAGAAAUGAUCGACGAGAA*

*>hg19_ct_UserTrack_3545_(null) range=chr5:149452751-149452950 5'pad=0 3'pad=0 strand=+ repeatMasking=none*

*ACUUGCUCAAGGUCAUACACCAAGGUUGGGACAUGAGCCAAGCGUCCUGAUGCUUGCUGAAGCAUACCCCAUCUGGUUGUCGGGCUGUGUAGACGGGAGCUGAUAAGUGGUACCUGUAUGUGUCCUUGGUGGUAGCAUUAGCAAGCUUGGGCUCAGGCUGGUGGUCAGAAAAGGGUCCCAGGUAGGUCCAGUUAAAACCU*

*>hg19_ct_UserTrack_3545_(null) range=chr18:47113151-47113350 5'pad=0 3'pad=0 strand=+ repeatMasking=none*

*GCAUAUCCCCAGGCCGGGAGCUCUGGUUUCGCAAGUGUCGGGAUGGCUGGAGGAUGAAAAACGAAACCAGGUAACCAGGACUUUCUCACACGUUCCACCCAGGACACGUUGACAUGAUGAUCUCCUAGCAUGUGCUGGGGAUGGAUCUGGGUGCCAGGGACAUAGCAUGAACAAAACAGAUAAAAAUCUCUUCCUUUAAG*

*>hg19_ct_UserTrack_3545_(null) range=chr8:87544632-87544831 5'pad=0 3'pad=0 strand=+ repeatMasking=none*

*UAUAUUAGUGUAGGCUUUAUAUUAGAUAACAGAAUAAAUCAGUGCUUAAGUGUCUAUCAGAAUUCCUAAAACAUAUUUUCUUUCACUUACCUGAUCUACUCAUAGAUUUCAGCUGAAGAAAUAAAAGAUAAUAGAGUGGUCUUGUUUGAAAUGGAAGCCAGAAAACUGGAUAAUAAGGUGGGUAGACUAUGCAGAUUUCA*

*>hg19_ct_UserTrack_3545_(null) range=chr6:129633860-129634059 5'pad=0 3'pad=0 strand=+ repeatMasking=none*

*AAGUGACUAAACUAGAAUAGUACAAAGCCUAUAACAGAUACAUGAUUAAUAUGUGUUUAAUGGAAGCAUAGAAACAUUAGAAUUAAAAUUUUAAGAUAUUUCCCAUAAAGUUGUGUUAAUGGUUGCUGUUUUUAUCUCCUCUAUAGGCUUGUAACUGCAGCACAGUGGGAUCCUUGGAUUUCCAAUGCAAUGUAAAUACA*

*>hg19_ct_UserTrack_3545_(null) range=chrX:54475227-54475426 5'pad=0 3'pad=0 strand=+ repeatMasking=none*

*GCUGGCUCUUACCUCCAGGAUGGACCUCCGGCGCUGGGGUGUAUGCUGGCUGCAGGCUGGACUGCUCCCAGGCACCCCGUGCAAGGCCACAUAGCAAUCAGUGCACACACGGUUGGAGCGGUUGUUGUCAUAGACGAGGCGGGCCCGGAACUCGGAGCACUUCCCACAAACCACCUGGGGUGGCAAGUGGGCAAGAGUAG*

*>hg19_ct_UserTrack_3545_(null) range=chr5:68805349-68805548 5'pad=0 3'pad=0 strand=+ repeatMasking=none*

*GGCUGCCUUUUGUUUCAUUGCCGCGUUGGUGAUCUUUGUUACCAGUGUUAUAAGAUCUGAAAUGUCCAGAACAAGAAGAUACUACUUAAGUGUGAUAAUAGUGAGUGCUAUCCUGGGCAUCAUGGUGUUUAUUGCCACAAUUGUCUAUAUAAUGGGAGUGAACCCAACUGCUCAGUCUUCUGGAUCUCUAUAUGGUUCAC*

*>hg19_ct_UserTrack_3545_(null) range=chr6:32008237-32008436 5'pad=0 3'pad=0 strand=+ repeatMasking=none*

*AGCUCCCGGGUCCCCUACAAGGACCGUGCACGGCUGCCCUUGCUCAAUGCCACCAUCGCCGAGGUGCUGCGCCUGCGGCCCGUUGUGCCCUUAGCCUUGCCCCACCGCACCACACGGCCCAGCAGGUGACUCCCGAGGGUUGGGGAUGAGUGAGGAAAGCCCGAGCCCAGGGAGGUCCUGGCCAGCCUCUAACUCCAGCC*

*>hg19_ct_UserTrack_3545_(null) range=chr17:28575961-28576160 5'pad=0 3'pad=0 strand=+ repeatMasking=none*

*GGCUUCAGUCCCUGGAUCUGUCCUUUGCAGCUACGUCAGGUUCCAUGGAAGGAGGAAAGAGCUGGAGGGCAGUAUCACUCAGCCAAAGCUCCCAUGGGGUCCCAUGCUGGCAGGAUAAUGGGUUCCUGCUCUAACACAGCUAGCACCUCUUCAGGGACAUGCUUCCUGUCCACCACCACUUCGUAGACAUACUCAGAGAA*

*>hg19_ct_UserTrack_3545_(null) range=chr16:49559183-49559382 5'pad=0 3'pad=0 strand=+ repeatMasking=none*

*GAGGAAGAGCACACACUGGGAUCCCGCAAUAACAGGGCAGGGCUCCUGUGUUCAUCUCUCUCCCCUGAGGGGCACAAGCUGGGUACCUUACCUGUGAAACACACGGGGCAUUUGAAGGUGCCGCCCAUGCCCUCGAAGCUGUGCUCAAUGAGGUGACAGAGGAGCUUGGCCGGGGAGUCGAACAUCUGGUUGCACAGCUU*

*>hg19_ct_UserTrack_3545_(null) range=chr4:1991462-1991661 5'pad=0 3'pad=0 strand=+ repeatMasking=none*

*UGGCGCUCUUGGGUUUCCGCUUUAACUGAAAAUGCUUCACCGGGGGAGUGAGGGGUCCCGCGAGGGUCGUCAGGGCGUUUUUGUUCAAGUACUGGCACUCCAGUGGCAGCAUGGCAGACGCUUCACACUCACCCACUGCCGGUAAGAACCACAUGAAGUUAGGGGCGCCCAGGCCGCAGACCUCCCGGCUGAGAGGAGCU*

*>hg19_ct_UserTrack_3545_(null) range=chr11:61506877-61507076 5'pad=0 3'pad=0 strand=+ repeatMasking=none*

*CUGCCCCCCAGCCAGUGCUACUUAGCUCUGUCCCCAUCUCUCCCACUCCACCCCGCGCAGUGGCGGAUCAUCGUGGGGGCCACCAAAUGCAUCCCCAAGUCGGAGCUGCCUGAGGAGGUAGAGGUGACCACCCUGGCCAGCACGCGGCUCUGGACCCACCCCAGCGACCUAACUAUAGCCCUCUCAGCCAGCACUCCACU*

*>hg19_ct_UserTrack_3545_(null) range=chr9:35700108-35700307 5'pad=0 3'pad=0 strand=+ repeatMasking=none*

*GCCCCGCAGAUCCCUACAGUUUCUCUCCCACUAUGUUCUGGCCCAAAGCUGCCUCACGGAAAUGCCUCAAGGAUUUCUACCUUGCAAGCCCGAAGCAUAUCUGCAAUAGCACGGCGGCUCAGAUUGGCUGUGGCAAUGACAUCUUCCUGGCGACAGGAAUUGCCAGCAGCAACGGCCUUGGCGGUUGCCAUGGUGAUACC*

*>hg19_ct_UserTrack_3545_(null) range=chr1:216372910-216373109 5'pad=0 3'pad=0 strand=+ repeatMasking=none*

*ACAAUAGAUUCUCAUUCAUGUCUUGACCAAAAAGGGGAAUCUCAGCCUUGGAUUCUUACCAUUUAGUUCCGCUGGUGGAGACCAUUCUACAUGAAGUUCUGUAGAACUGAUUUUCUGCAUCUUAGGUGGACUUAGUCUUUGGGGAGGGGCCUGGGCUGUGGUCACUGUAAUGGGCAAGCUGUGUAAACAGCCCCCGCUAG*

*>hg19_ct_UserTrack_3545_(null) range=chr5:353821-354020 5'pad=0 3'pad=0 strand=+ repeatMasking=none*

*CUGACCCAGACCAUCUCCCCACAGGAGGCCCGCCGUGGGGGCAGAGAAGUCCAACCCCUCCAAGCGACACCGGGACCGCCUCAACGCCGAGUUGGACCACCUGGCCAGCCUGCUGCCGUUCCCGCCUGACAUCAUCUCCAAGCUGGACAAGCUUUCUGUCCUGCGCCUCAGUGUCAGUUACCUCCGGGUGAAGAGCUUCU*

*>hg19_ct_UserTrack_3545_(null) range=chr22:30891809-30892008 5'pad=0 3'pad=0 strand=+ repeatMasking=none*

*AGAAGAAACAGGAGAAUGGGACAGGGCCAGAGUCUCUACCCUUUUGCAGCAAAUCCCUGGGUCACAGUGCAUGGAAGGCAGGGCAGUAGGGAUCACAGAUCCUUACCCUUCGGAGCAUGUCUUCGGAUUUCUGCAGGUCAAAGUUUCGAGCUGCAAGAAGAGGAGGUAAAUAGAAGCUCAAGGCUAGAUCCAGAAAGAGC*

*>hg19_ct_UserTrack_3545_(null) range=chrX:118124388-118124587 5'pad=0 3'pad=0 strand=+ repeatMasking=none*

*AACCGGGGUGGCUCCAGCGCUGACUUCUUGCUUUCUAAAUCGCAGAUCCUCCCACUGAUCAGGGGGACAAACCUGCUCUCAGUUUACCACUUGCAUCUUUCGACGCAUCUGACCUUGAAUGCGCUCUAUGUAUGAGGUACGUCCUGUGUACUAUCAUUUACUUCCUGAUGUUUCUUCAUCUCUGGUGUCUGGACCUUUCU*

*>hg19_ct_UserTrack_3545_(null) range=chr9:121930016-121930215 5'pad=0 3'pad=0 strand=+ repeatMasking=none*

*CAUGCCGAUCACCAUGUGGAUGAAGUCCAUGCGGUUCUUGUUGCUCUUGAGAGUGAGGGACAUGCGCUUGCGCCACCGAGGGUCAAAGAAGGUGUCGAGGCGGAUCUCGUUGCUGAUGAAGGUGGUGUGGACGUAGAGGCGUGAGUCCAUCUUCUGCAGCAGGUACUUCAGCUCCAGGUCCUGGAAGUCCAGGUCAGUCU*

*>hg19_ct_UserTrack_3545_(null) range=chr1:40776292-40776491 5'pad=0 3'pad=0 strand=+ repeatMasking=none*

*CUUUUACAAGCUAGAGGCCUGAGCAGGGGAAUGACUCCAUGAAGGGCUCCUGGGGUGAGGGAGAAGAGGGCCACUGAGGCAAGGUGUUCCUUACCGGUGGUCCAGGGAUGCCUUGCUCUCCAGAGGCACCCACAUCUCCCUUGGGACCCUAAAGGGCAGGGAUGAGCUGUCAGACAGGCAGGCAGAUGGAAAGACACAUA*

*>hg19_ct_UserTrack_3545_(null) range=chr11:120996066-120996265 5'pad=0 3'pad=0 strand=+ repeatMasking=none*

*UCUACCAGAGUGGCAUAUCUACUGCCGUGGAAACAGAUUUUGGGCUCUUAGUGACUUUUGAUGGCCAGCACUACGCCUCCAUUUCCGUCCCAGGCUCCUAUAUAAACUCCACCUGUGGACUCUGUGGAAACUAUAAUAAAAACCCACUGGAUGACUUCCUCCGCCCGGAUGGCAGGCCGGCCAUGUCUGUCCUGGAUCUG*

*>hg19_ct_UserTrack_3545_(null) range=chr20:43577343-43577542 5'pad=0 3'pad=0 strand=+ repeatMasking=none*

*CCCAGGCCAGCAGCUGUAUAGGAGAUACCUGUUGCUGUACGUGGCAGAUUCCAGGUUACUACACAAGAGGCUUUCACUGUACUUCUCAAUAGCUUUCUUAUGGUUUCCCUUCUUUACAAGCUCAUUGCCUUCUUCCUUCAGAACUCUGGCUUUCUCCACAUCCCCAGCAGAAGGCACUAGAUACAAAUUCAGAAGAGGAA*

*>hg19_ct_UserTrack_3545_(null) range=chr6:131903693-131903892 5'pad=0 3'pad=0 strand=+ repeatMasking=none*

*UUUACCUUUGAAUGUAGGAUUUGUUCAAGAGAAUCAUACAUAACCAAGUGAAAACAUUGUAAUUUUAGAUUCCCGAUGUGCCAGGAUUCUCCUGGGUGACUCCCUGUAUAUCUGCCAAGGAUAUUGUGUAUAUUGGCUUGAGAGACGUGGACCCUGGGGAACAGUAAGCUUAUUCCUUGAUGUGAUUUGCCUCCAUUUUU*

*>hg19_ct_UserTrack_3545_(null) range=chr17:27975144-27975343 5'pad=0 3'pad=0 strand=+ repeatMasking=none*

*AGAAAGUCACUGACUCACCUUGCCAGCAAGAUCCCCUGAUACUCUUCCAGUUGUCUCAUGAAGCUUGGGUUGGGCUUGGUUACCGUUCGUCUUUCUUUCACAUAGUCAUAGGCUCGGUCCAGAUUCCAGCCAUAUUCCUUCAUUGCAUAGGCAAUCACGGUGGAGGCUGAGCGACUCACCCCCAUUUUGCAGUGCACAAG*

*>hg19_ct_UserTrack_3545_(null) range=chr9:94493202-94493401 5'pad=0 3'pad=0 strand=+ repeatMasking=none*

*UACGUCACACAGUUCCAUGCGUACGUUUUUAUUCUGCGUAAAGCACCAGGGGCCCUCCAUCUGGCCUCCGGGGUUCCGGCAGUAGGCGUGCCCCCCUCCAAGCUCAGGGAAGUCUGUGCUGGACAGGUGGUGGCUGUGGGGGUGCUGCAGGGCCCACGGCUGGCACUGGUGGCCUGACUUGGUGGUGCUUGCCGUUCCUC*

*>hg19_ct_UserTrack_3545_(null) range=chr17:39036780-39036979 5'pad=0 3'pad=0 strand=+ repeatMasking=none*

*UCUCCUGUUUCUCCCGCUCCUCCUAUUAUUAGUAUUAUAUAGAACCAUUCCAUUGUUUCCAAUACCACUUCUGAAUAUUUUCUCACCUCCUGAUGCUCCUUUUUGAGGAGAGCUAGGUCUUUAUUCAGUUCUUCAAUUUGAAUCUCCAAAUCUGUUUUAUGUAGGGUUAGGUCAUCAAAGACCUUAUUCAGGCCUUGGAG*

*>hg19_ct_UserTrack_3545_(null) range=chr3:184017042-184017241 5'pad=0 3'pad=0 strand=+ repeatMasking=none*

*AGUGGCGGCGGAGAUGGAGGAGGGAGGCCGGGACAAGGCGCCGGUGCAGCCCCAGCAGUCUCCAGCGGCGGCCCCCGGCGGCACGGACGAGAAGCCGAGCGGCAAGGAGCGGCGGGAUGCCGGGGACAAGGACAAAGAACAGGAGCUGGUGAGGCGCGACCCCGAGAGUCGGCGAAGGAGGCUGCGGGGCUGAGUCACGG*

*>hg19_ct_UserTrack_3545_(null) range=chr6:169631944-169632143 5'pad=0 3'pad=0 strand=+ repeatMasking=none*

*GCAAGAGGCCUGAGAGACCCUCCUUCCAAAUUCCCUGUGCUGCCCCCGCCCCCCGUCCGCGUAGCGUCCCCGGCGCCAGGAGGAGGGCUGACCGCCGUGGCCCCGUGUGCCCUGCGAGCCCCGCGCCUCACCUUGAUGCAGUGGUAGGUGGCGUUGGUGGCGCAGACCAGAUUGAGGUUGGGCCAGCCGUCCAGGUCCGA*

*>hg19_ct_UserTrack_3545_(null) range=chr6:83933492-83933691 5'pad=0 3'pad=0 strand=+ repeatMasking=none*

*AGGAAAAUAUUAUCUGUGAUCUGCCUCAAUCCACACGCCACAACACCAAGAGCAACUCCAGGGAACACAUAGGAAUUGUUGCCUUGGCCAGGAUAUAGGGUCUGUCCAUUUGGAAGAGUGACUGGAUCAAAAGGACUGCCACUGGCAAAAAUUGCACGUCCCUACAACAAAGACACAUACAACUCACUUUAAAAAGAAGC*

*>hg19_ct_UserTrack_3545_(null) range=chr1:112309048-112309247 5'pad=0 3'pad=0 strand=+ repeatMasking=none*

*AAAGGAAAUAAGUCAUACUUGGAAGGCUCUUCUGAUAAUCAGCUGAAAGACUCUGAAUCUACGCCUGUGGAUGAUCGUAUUUCUUUGGAACAACCACCAAAUGGAAGUGACACCCCCAAUCCAGAGAAAUAUCAAGAAUCACCUGGAAUCCAGAUGAAGACAAGACUUAAAGAGGGGGCUAGCCAGAGAGCUAAGCAGAG*

*>hg19_ct_UserTrack_3545_(null) range=chr8:118812019-118812218 5'pad=0 3'pad=0 strand=+ repeatMasking=none*

*CGAGCCUCAUCUGAGAGUGGAUCAGCGGCAUGUAGCCAAACCAGCUGGCAAACGUAUUCAUGCAGCUCUGUCGCUGGGCAAAGUGGUCAGGGUCAGCCCAACGGGAAGCCCGAGAAGUCUAGGGAGAAGGAGAGAAACAAGGAUAAUGAUGAGAGAAGUGCAAGGUGAGAUGGAAACAUUGCAGAAAAUGGAGUCAGGCA*

*>hg19_ct_UserTrack_3545_(null) range=chr9:136224531-136224730 5'pad=0 3'pad=0 strand=+ repeatMasking=none*

*CAGUCUGAAUCCUUGCUGUGAACUGUCCCUACAAAUUUGGUCUCUCUGCUCUGUAGGCACCAGUUGUUCUGCAAACUCACCCUGCGGCACAUCAACAAGUGCCCAGAACACGUGCUGAGGCACACCCAGGGCCGGCGGUACCAGCGAGCUCUGUGUAAAUGUAAGUCCCAGUGGACCCCCAUCAGUGCAUCGCCAUCUGA*

*>hg19_ct_UserTrack_3545_(null) range=chr11:102649792-102649991 5'pad=0 3'pad=0 strand=+ repeatMasking=none*

*AAAUCUGCUGGCACCUUAAUCUUGGACUUCCCAGCCUCCAGAAUUGUGAGCAAUAAAUUUCUGUUGUUUAUAAAUUACCCAGUCUAAGGUAACUUGCUAUAGCAGUCUGAACAGAUGAAUACACCAGAUAAAUGGAUGCUUUAUUUGAUUACCUUUAACUGCAAAAGAGAUCAUUAUAUCAGCCUCUCCUUCAUACAGCC*

*>hg19_ct_UserTrack_3545_(null) range=chr7:100200946-100201145 5'pad=0 3'pad=0 strand=+ repeatMasking=none*

*CAGCUUCCCUCCUUGGAGUUUCAUCCUCAUCACCUCAUCCCCAUCUUGCCAGUGCUCCCCCGUCCCACCCGUCCCUGCUCUUUCCUGACCCCUUUUCUGUUCUCCAGACCCGUGUUCCUGUGCGGAGGGGAUGUGAAGGGGGAAUCAGGUUACGUGGCAAGUGAGGGGUUCCCCAACCUCUACCCCCCUAAUAAGGAGUG*

*>hg19_ct_UserTrack_3545_(null) range=chr10:106737056-106737255 5'pad=0 3'pad=0 strand=+ repeatMasking=none*

*AACUCUGUCUGUAUAUGUCCCUCUAUCCUUUCUUUAGAUUAUGCUUCUCAGUGAUCCUGAGAUGGAGAGCAGCAUAUUGAUCAGCUCAGACGAAGGGGCGACCUAUCAGAAGUAUCGGCUCACCUUCUAUAUCCAGAGCCUGCUCUUUCAUCCCAAGCAAGAGGACUGGGUGCUGGCCUACAGUUUGGAUCAAAAGGUGA*

*>hg19_ct_UserTrack_3545_(null) range=chr6:31545130-31545329 5'pad=0 3'pad=0 strand=+ repeatMasking=none*

*UCAAGAGCCCCUGCCAGAGGGAGACCCCAGAGGGGGCUGAGGCCAAGCCCUGGUAUGAGCCCAUCUAUCUGGGAGGGGUCUUCCAGCUGGAGAAGGGUGACCGACUCAGCGCUGAGAUCAAUCGGCCCGACUAUCUCGACUUUGCCGAGUCUGGGCAGGUCUACUUUGGGAUCAUUGCCCUGUGAGGAGGACGAACAUCC*

*>hg19_ct_UserTrack_3545_(null) range=chr7:107591586-107591785 5'pad=0 3'pad=0 strand=+ repeatMasking=none*

*GUUUUAUAAUUUACAGAAGGCUCUUGUAGAACCCCAGUUCCCUCCACUCUCUGAUCAAGUCAAUGCGAGAACGCCCACAACUCACCUCGGCACUCCACGUCGGGGUCUCCCCAGAAGAGUUCCUGGCACUCGCUGCAGGUGCGGCCUCCAAACCCAGGCAUGCACUGGCACUGCCCCGUGAACUGCGGCCAGAACACAGA*

*>hg19_ct_UserTrack_3545_(null) range=chr16:230633-230832 5'pad=0 3'pad=0 strand=+ repeatMasking=none*

*CCGGACGCGCCUCACCCACGUUCCUCUCGCAGGACCUUCCUGGCUUUCCCCGCCACGAAGACCUACUUCUCCCACCUGGACCUGAGCCCCGGCUCCUCACAAGUCAGAGCCCACGGCCAGAAGGUGGCGGACGCGCUGAGCCUCGCCGUGGAGCGCCUGGACGACCUACCCCACGCGCUGUCCGCGCUGAGCCACCUGCA*

*>hg19_ct_UserTrack_3545_(null) range=chr12:52702150-52702349 5'pad=0 3'pad=0 strand=+ repeatMasking=none*

*CUGCGCCUGACGCGCGCCUCCGUCUCUUUCCCCUGCAGGCGUCAGCAGCUCCCGCGGUGGCGUUGUCUGUGGCGAUCUCUGCGCCUCCACUACUGCCCCUGUUGUCUCCACCAGAGUCAGUAGCGUCCCCAGCAACAGCAACGUGGUGGUGGGCACUACUAACGCCUGCGCCCCCUCCGCCCGGGUUGGCGUCUGCGGCG*

*>hg19_ct_UserTrack_3545_(null) range=chr2:136603675-136603874 5'pad=0 3'pad=0 strand=+ repeatMasking=none*

*UAAAGUGAGUUUAUCUAACAACUUAUCCUCUCUUCCGACAGAACAACACGAAGUUUGGCAAUCACAAGUGGCCUAUGUGAACAGAGCUUACAGAAGUGACCGAGCAUGUAAGCAGUACCAGGUAAGCCUCACCUUCUUCCACCUUUCUGAGGUGAAGCACAAUAAGGUUAGAGAUUCGGCAGUACUCAGAGAAGCCCAGC*

*>hg19_ct_UserTrack_3545_(null) range=chr22:37163741-37163940 5'pad=0 3'pad=0 strand=+ repeatMasking=none*

*UCCCUCAAGGAACUUCGAAGUCACUCAUCAUGGUGAACCGAGCACUUCUCUAUAAGCUGUGGCCAGGUCUUGACACCCCAGCUCACCAGGGUGUAGCUUUUCUGGAAAUGGGCUCCAUCACUGCGGAAGAUCUGUGCCAGGGCGGUCUUGCCCACUGCUGGGUCUCCUGUGAGAUCAGAAAAGAAGAAAAAGAACGCCUU*

*>hg19_ct_UserTrack_3545_(null) range=chr11:46748059-46748258 5'pad=0 3'pad=0 strand=+ repeatMasking=none*

*GAGGAGGAGACAGGAGAUGGGCUGGAUGAGGACUCAGACAGGGCCAUCGAAGGGCGUACCGCCACCAGUGAGUACCAGACUUUCUUCAAUCCGAGGACCUUUGGCUCGGGAGAGGCAGGUGAGGUAGUGGGCAUCCGAGGGGAUGCGGGGCUGCGGGGCUGGUGGCCAGGACUUGCCCCUCACUGCUUGGCUUGCUCUGC*

*>hg19_ct_UserTrack_3545_(null) range=chr2:33482330-33482529 5'pad=0 3'pad=0 strand=+ repeatMasking=none*

*AUUUUCCCAUCCCAAAUCUAGCUGCUUUUAAGGAAAUCUGUCCUGGUGGAAUGGGUUAUACGGUUUCUGGCGUUCAUAGACGCAGGCCAAUCCAUCACCAUGUAGGUAAAGGACCUGUAUUUGUCAAGCCAAAGAACACUCAACCUGUUGCUAAAAGUACUCAUCCUCCACCUCUCCCAGCCAAGGAAGAGCCAGUGGAG*

*>hg19_ct_UserTrack_3545_(null) range=chr12:9243741-9243940 5'pad=0 3'pad=0 strand=+ repeatMasking=none*

*AACUUUUAAUUUACUUGAUGGCUACCUUGUAUUUAAUUUAGGAAAGAGAUCCUUACCCGGAUGCAUUUGGGAAGGUAGUUUAGGACCGUGGCCUUGAGUGUGAAGGCCUCUCCACGAAUCACAGAGUAAGGCAUUGUGAGCUCCACAAAGAAGGGCUGGAAGGCUCGGAGAGAGGCAGUGGAAGAGAUACCAAGUCCAGC*

*>hg19_ct_UserTrack_3545_(null) range=chr15:90630568-90630767 5'pad=0 3'pad=0 strand=+ repeatMasking=none*

*GAGCUGAGCCAAAUGCACUCUAAUAGCGACCUUCCCAGGGUAGGAUGGGAACAGCAUGGGGGGAAGGGAAGAAAGGCCACAGAGUACAUGGAUGAGGCUUUACUUGUCAAAGAUCUCCUGGAAGAUGUCCUUGAAACGCCCAUCGUAGGCUUUCAGUAUGGUGUUCUUGGUGCUCAUGUACAGCGGCCAUUUCUUCUGGA*

*>hg19_ct_UserTrack_3545_(null) range=chr15:75014639-75014838 5'pad=0 3'pad=0 strand=+ repeatMasking=none*

*UUGACCAUCUUCUGCAUGAAGCUGUAGAACUUCUCAUUCAGGUCCUUGAAGGCAUUCAGGGAAGGGUUGGGUAGGUAGCGAAGAAUAGGGAUGAAGUCAGCUGGGUUUCCAGAGCCAACCACCUCCCCGAAAUUAUUAUUCAGGUUGACUAGGCUAAGCAGUUCUUGGUGGUUGUGGUCAUAGCGCCGGCCAAAGCAAAU*

*>hg19_ct_UserTrack_3545_(null) range=chr5:176318041-176318240 5'pad=0 3'pad=0 strand=+ repeatMasking=none*

*CUGGCCAGCACCCAGCAUCACCUCUUGGGGGAUCACAAACUCCUGGCUUCUGGGCUCCACCCUAUGCCCCUCAAUGCCAGUUAGAGUCACCCACAAAACACGCAGUGAGGCCCCUGUGGCCCCCAGCUCCAGCACCACGAAGUCUCCUUGCUCUGGAGGGCAAGCAGGUCACAGAAAGCCAUGCAGCUGGCAAGACCAGA*

*>hg19_ct_UserTrack_3545_(null) range=chr8:86356133-86356332 5'pad=0 3'pad=0 strand=+ repeatMasking=none*

*AAUUUUCUCAGCUUUUAUGAGAGUGCUCAGCAACCUUAGCCUGUACAAUGAUUGGGUUUAUUGUGGAUAAUGUUGUACUUAUUUCCAUAUUCCAGGAAUUUACCUUCUAAUCUGUCUUCUAUGGUUUCAGCUUCAUUUGGUUCACUGGAACCCGAAGUAUAACACUUUUAAAGAAGCCCUGAAGCAGCGCGAUGGGAUCG*

*>hg19_ct_UserTrack_3545_(null) range=chr3:1444869-1445068 5'pad=0 3'pad=0 strand=+ repeatMasking=none*

*UGUGAAUGAGACAAGUAUAAUAAAAUAUGUUACUAGAAGAGAAAUGAUAGUAUGAAAUUGGGGUUUUGAAAAAUUAAUCUAACAGGCAAUACUUUAUCUCAAAAUAUUUUUGUCUUAUUUUUAUAUUUUGCAGGUUUGAGUUCCAGAGGAAUUCAAUUCUUAGAACCUAGCACCCAUUUUCUUUCCAUUGUCAUUGUGAU*

*>hg19_rmsk_AluJo_1*

*GCCAAGGCAGGAUGAUGGCUUGAGCCCAGGAGUUUGAGACCAGCCUGGGUAACACAGCAAGACACUGUCUCUACAAAAUAAUAAUAAUAAUACUACAAAAAUUUGCUGGGUAUGGUGGUGUGUGUCUGUAGCCUUAGCUGCUCAGGAGGCUGAAGUAGGAGAAUCACUUUAACUCAGGAGUAUAGGGCUGCGGUGAGCUA*

*>hg19_rmsk_AluSx_1*

*UGAGACCAGCCUGGCCAAUAUGGUGAAACCCCGUCUCUACUGAAAAUACAAAAAAAAUUAGCCGGGCGUAGCGGUGCACACCUGGAGUCCCAGCUACUCAGAAGGCUGGGGCAGAAGAAUCACUUGAACCCAGGAGGCAGGGGUUGCAGUGAGUGGAGAUCGCACCACUGCACUCCAGCCUGGGCAACAGAAUGAGACUC*

*>hg19_rmsk_AluSc_2*

*GGUAGGCAGAUCACAAGGUCAGGAGAUCGAGACCAUCCUGGCCAACAUGGUGAAACCCCUGUCUCUACUAAAAUACAAAAACUUAGCCGGGCAUGGUGGCGCACGCCUGUGGUCCCAGCUACUCGGGAGGCUGAGGCGGGGGAAUUGCUUGAACCCGGAAGGCAGAGUUGCAGUGAGCCAAGAUCGCGCCACUGCACUCC*

*>hg19_rmsk_AluSx_2*

*GAAACCAGCCUGGCCAAUAUGGUGAAACUCUGUCUCUACUAAAAAUACAAAAAUUAGCCGGGCGUGAUGGCAUGCGCUUGUAAUCCCAGCUACUCGGGAGACUGAGGCAGAAGAAUCGCUUGCACCCGGGAGGCGGUGGUUGCAGUGAGCCGAGAUCAUGCCACUGCACUCCAGCCUGGGCGAGACUCCGUCUCAAAAAA*

*>hg19_rmsk_AluY_2*

*CACCUGAGGUCAGGAGAUCAAGACCAUCCUGGCUAACACGGUGAAGCCCCGUCUCUACCAAAAAUACAAAAAAUCAGCCAGGUGUGGUGGCGGGCGCCUGUAGUCCCAGCUACUCGAGAGGCUGAGGCAGGAGAAUGGCGUGAACCCGGGAGGCAGAGCUUGCAGUGAGCCAAGAUUGCGCCACUGCGCUCCAGCCUGGA*

*Training sequences*

*>hsa-mir-3678*

*AUCUUCCAGAAUGAAGCACUCCAAAUAGAGGACCAGGUCUUAAAAUAUUUGAGAAUCCGGUCCGUACAAACUCUGCUGUGUUGAAUGAUUGGUGAGUUUGUUUGCUCAUUGAUUGAAUCACUGCAGAGUUUGUACGGACCGGAUUCUCCAAUAUAGGCCGAACACUGUGCUGGGGAUAGAACAGAAAUCUUGGCCCUUAA*

*>hsa-mir-4306*

*UUGCUGGGUGAGGCCUGCAUGAGGACUUUACUCAUGGGCUCAUAGAAGCCCUUUUAAGCUGCUUAGUGUCCUUAGAGUCUCCAGAGGCAUCCCUAACCCAGAAUCUUUUGACUGUCCUCUGGAGAGAAAGGCAGUAGGUCUGUACCAAGACCAAGGGGCUUGAAGGCUUGGACGCUCUGCUUCCACCUGCCUCUGGAGUG*

*>hsa-mir-3201*

*AGAUGUCCUUUAUUAGGAAAGAUAGACAGAAGUCCAUAAACAAGCAAAUGCACAGUGUGUCAGAGGUGUCCAGGGAUAUGAAGAAAAAUAAGAGGCUAGGAUUGCCUCUUAUUUUUACAUGCCCAUAAUCCCAACACUUUGGGAGGCUGAUACAGGUGGAUCUCUUGAGCCCAGGAGUUGGAGACCAGCCUGGGCAACAU*

*>hsa-mir-3618*

*GAAGAAGAAAGGUGCCACUCCGGCAUGAAGACAGACUCGCUUAGUCGCCAGUCACUUAAGCUGAGUGCAUUGUGAUUUCCAAUAAUUGAGGCAGUGGUUCUAAAAGCUGUCUACAUUAAUGAAAAGAGCAAUGUGGCCAGCUUGACUAAGCCGCCAGCGCACAGCGCGGCAGGACGCGCCCGGGUCUCAGCGGACUUGUG*

*>hsa-mir-4263*

*AAGCUAGUUUAUGGCUUUGGUAAGAGUCUGUUUACUUCUGGUUCACUUCUGUUCUGGGAUAGUGCUCUUCAGGGUUUUACUUGGGAGAUUGGAGUGGCCAGUGUUCCUAAACAAUUCUAAGUGCCUUGGCCCACAACAUACUUCAGAAUAGAAUUCUCUGUCGUUUGUCAAAUAUUUAAACACACAAGCAGUGUGGAUCU*

*>hsa-mir-555*

*CAUGUUCCCCAAACUGUAUGUUUUUCUACAGGUAUGGCAGCACUGUGAUUGUAUGGGAGUGAACUCAGAUGUGGAGCACUACCUUUGUGAGCAGUGUGACCCAAGGCCUGUGGACAGGGUAAGCUGAACCUCUGAUAAAACUCUGAUCUAUAAGUAGGAGAGGUAGCACAUGGUAUCUCACCUGUCCUGCUCCUGCUUUG*

*>hsa-mir-4302*

*GGAGAGCAGAGCUCUAAGCAUGGUGGAUACUGAUGAGGAAUUCUGAAACUGUGAUGCAGAUCCUGGGGUCACUUCAGGAGGGACCAGUGUGGCUCAGCGAGGUGGCUGAGUUUACUUAAGGUAUUGGAAUGAGGUAGAAUUACUAUUUCUAUUCCCAAAUCACCAAAGUCAUGAAGCUUAAAAAAAGCAUCUCUUCCAAA*

*>hsa-mir-3653*

*GUGGGAAGGUUGCGUCAUGGACCCAGCCAGUGAGGCUAGGACUCCCUGGGGACCCCUGGCAGCCCCUCCUGAUGAUUCUUCUUCCUGAGCACGCUCAUGAUGAGCAAACUGAGCCUCUAAGAAGUUGACUGAAGGGGCUGCUUCCCCAAGGAAGCCUGGGGGCCAGUAGGUCCUGACCCUUGGGCAGACCGGGGCUAGGG*

*>hsa-mir-4258*

*GAAAGUCAGUCCCCUACUUUUGCUAACUCCAGGACGCUCCCCCAUCCCACGCUUCGAGAACGCCCCCCGCCCCGCCACCGCCUUGGAGGCUGACCUCUUACUUUCGGUCGGUCUUCUUCCCUGGGCUUGGUUUGGGGGCGGGGGAGUGUCCUAAGGGCCAAUUCAGCGUGAUGUCUCUUUCACCACCAUUGAAAACGAAA*

*>hsa-mir-3607*

*AUUGGAAAGAGUGUCAUAGUUUAGAGGGGGAAUAACUUAUCCAAGCUACUAAUGCGGAAAGGUUGCGGUGCAUGUGAUGAAGCAAAUCAGUAUGAAUGAAUUCAUGAUACUGUAAACGCUUUCUGAUGUACUACUCAAACUGAUAGAAAAUAUUUUCAUGAAACUACAUGAUUUCUGUUAAAUGAAUGGGAUUUUUAAAA*

*>hsa-mir-1183*

*CAGACCAAGGGAGAUGGAGGUUUGGGGUGGGAGAAUGUGUUGGAUUCCAGCUAUAUUAUUCAAAUGCUCGGAGACACAGAACAUUAGAGAAGACAGGAGUUCACUGUAGGUGAUGGUGAGAGUGGGCAUGGAGCAGGAGUGCCAAGAGAUGAAGUUAUAAAACUAAGCAGGAGUUAGAUCAUGAAGAACUUUAUGUACUA*

*>hsa-mir-1208*

*UGCUAAUUGAAGAGCUCAGAAAGAGGACAUUAAGAAGGGGGCUUUCUGGUGGGCAACAAUGAAUCACCGGCAGAAUCACUGUUCAGACAGGCGGAGACGGGUCUUUCUCGCCCUCUGAUGAGUCACCACUGUGGUGGGGGGGAGUGGACAGUCAGCCAGACAGAUAAUGUGUCAGUCUCUCUCCUCAGCCUGACCCUGGA*

*>hsa-mir-3621*

*GGGUGCGCGCUGCGCCCGCUCUGCGCCUGGCUGCGCCGGCACCCGCUGCCCGCGUCAGGUGAGCUGCUGGGGACGCGGGUCGGGGUCUGCAGGGCGGUGCGGCAGCCGCCACCUGACGCCGCGCCUUUGUCUGUGUCCCACAGAGGCCGAGACGGUGCUCUGCGUGUGGCCGGGACGCCUGACGCUCAGCCCCCUGACUG*

*>hsa-mir-3656*

*GAAAACUUUCAGUUAUCCGCUGGAUCUGCUGCUCAAGCUACACGAUGAGCGUGUGUUGGUUGCUUUCGGCCAGCGGGACGGCAUCCGAGGUGGGCUAGGCUCGGGCCCGUGGCGGGUGCGGGGGUGGGAGGGCCCCAGUGCUGUAGAAGUGGGCUGGUUGUAGGUGCGGGGUUGGGGGAAAAGGGGUUGUGUCGGGUCCC*

*>hsa-mir-4256*

*CUUGCUUUCUGGUUCAUCUUAGAUACUUAAAAAACAUGUUGUUUGAGGGGAAUUAAUGAUGUUUUAUUUUUGUUCCAUUUAUCUGACCUGAUGAAGGUCUCCUGGCAUUGAUUAGGUCUGAUGAUCCAUUUCUGGGGUGGCUGUGGAGCAGCAUGGUGUCCCGCAUGCAUUUUCUAAACUUGCUCUAUCUGAAGAAUCAC*

*>hsa-mir-425*

*GUCCUCAGUUUGACUGAGGGUUCCCAACCCUGCUAAGCAGUUGUCUCCAGGUCAUGCACCUUCAGAAUGGAAAGCGCUUUGGAAUGACACGAUCACUCCCGUUGAGUGGGCACCCGAGAAGCCAUCGGGAAUGUCGUGUCCGCCCAGUGCUCUUUCGGCGCCUCCCAGCAGGGCCUCAGCCCUGGCCCAGAAAGGGGCCU*

*>hsa-mir-4292*

*UGGAAAACACUGGCUCAGACAGCGGCUUCAAGCACCACGCAGGCCCUUCACAGCCACGAGAGGCAGGAGACACCAGAAGGCCACCUGCUUAGGAGGCCAGAGGUGCCCCUGGGCCGGCCUUGGUGAGGGGCCCGUUUAAAUGACUGCGUGUAAAUGAAAGAAAAUGAAGUUAGAAACAGGAUGUUGCAUUUUUUACUCCC*

*>hsa-mir-4298*

*UAUUUCUAGAUUCAUCUGGAACGAAUUGGCUAGAUCCUUCUAUCCCAAGUCCCAUUGCUUUUCUGAGGGGAGGUACCUGGGACAGGAGGAGGAGGCAGCCUUGCCUCAGAAACCAAACUGUCAAAAGUGUAGGUUCCACUCAGGAAGGAGGAGCACAGGCACUUUUGAGAUGAGGGGGACGUGCUCUUCUCUGGAGCAGA*

*>hsa-mir-647*

*GGAAAAACACCUGAACGCAGCCAGACACAUUUUCCGUCGUCUGGUCAGCCAAGGAAGUGUUGGCCUGUGGCUGCACUCACUUCCUUCAGCCCCAGGAAGCCUUGGUCGGGGGCAGGAGGGAGGGUCAGGCAGGGCUGGGGGCCUGACCGCCCACACUCCUCCUCAGGCCUGCGGGGAACGCCCUGCUCCCCAACUAAGCA*

*>hsa-mir-675*

*CAUUGCGCAGCAAGGAGGCUGCAGGGGCUCGGCCUGCGGGCGCCGGUCCCACGAGGCACUGCGGCCCAGGGUCUGGUGCGGAGAGGGCCCACAGUGGACUUGGUGACGCUGUAUGCCCUCACCGCUCAGCCCCUGGGGCUGGCUUGGCAGACAGUACAGCAUCCAGGGGAGUCAAGGGCAUGGGGCGAGACCAGACUAGG*

*>hsa-mir-4487:11:47422457:47422658*

*GAGGGCGAUGCAAAUUCAGACCACAAUGAGAUGCUAUUUACACCAUCUGAAGGGCAGAAGGGAACUGUCCUUCAGCCAGAGCUGGCUGAAGGGCAGAAGGGAACUGUCCUUCAGCCAGAGCUGGCUGAAGGGCAGAAGGGAACUGUCCUUCAGCCAGAGCUGGCAAGGAGGGGAGCAGCAGGCCCUCCUGCAUGUGGCUA*

*>hsa-mir-4421:1:51525441:51525642*

*GGAGCUACCCACUUCAGGUCUCCUCUACUUGUCAGUAUGACCUGCCUGUGGAAAAGAGCUACUCACUCUGGGUCUCCUUUCUGCUGAGAGUUGAACACUUGUUGGGACAACCUGUCUGUGGAAAGGAGCUACCUACUCUGGGUUUCCUCUCCCCUGAGAGCUGGACACUCAUCUGGACAACCUGCCUGCAGCUAGGAGCU*

*>hsa-mir-4492:11:118781357:118781558*

*CGGAGAGGCAGAGCGAGCGAGCGACAGCCAGCGAGGCAGCGAGCGGGAGGCGGACAGGACUGCAGCGUGCUUCUCCAGGCCCCGCGCGCGGACAGACACACGGACAAGUCCCGCCAGGGGCUGGGCGCGCGCCAGCCGGCAAGAGCUUGGCGAGCCCCGGGUGCUGCCAGCAGAGGCUCAGAGACCCACAGACACCCGAA*

*>hsa-mir-4728:17:37882679:37882880*

*GCUACUUCUCUACCACCUGAGGGCUUUGGGCUGUCCCUUGGGACUGUCUAGACCAGACUGGAGGGGGAGUGGGAGGGGAGAGGCAGCAAGCACACAGGGCCUGGGACUAGCAUGCUGACCUCCCUCCUGCCCCAGGUUGGAUGAUUGACUCUGAAUGUCGGCCAAGAUUCCGGGAGUUGGUGUCUGAAUUCUCCCGCAUG*

*>hsa-mir-214*

*UUGUAUCUGUCUAUGAGCAAAGGAAACCUGAAGGAACCAAGGGCCUGGCUGGACAGAGUUGUCAUGUGUCUGCCUGUCUACACUUGCUGUGCAGAACAUCCGCUCACCUGUACAGCAGGCACAGACAGGCAGUCACAUGACAACCCAGCCUGAAUGACAACCAGCCAUUGAAAGAAAGCAGCCCUCACACCAUAGCAUCU*

*>hsa-mir-708*

*GGGUUUUCAGAAACCUAACCCCCAUGGUUGGCGAGGGACUGCUGUGUGUGAAAUGGUAACUGCCCUCAAGGAGCUUACAAUCUAGCUGGGGGUAAAUGACUUGCACAUGAACACAACUAGACUGUGAGCUUCUAGAGGGCAGGGACCUUACCCUAGUCAUCUCUCUUCUCACCCUGCACACCCUCCCUGAGGGAUCUCAU*

*>hsa-mir-501*

*AGUCAAAUAUAUAUGGAGAUAUACAUGCAGGGUGCAGGUGCCUGAGUCUUCUGCUCUGCUCUUCCUCUCUAAUCCUUUGUCCCUGGGUGAGAGUGCUUUCUGAAUGCAAUGCACCCGGGCAAGGAUUCUGAGAGGGUGAGCCCCCUCUGCGUGGAGAACAGAAGAUCUGUAUGUCACCUCUCAGCUGCUUGCUGCUUGCC*

*>hsa-mir-27a*

*UGCCACCGAGGAUGCUGCCCGGGGACGGGGUGGCAGAGAGGCCCCGAAGCCUGUGCCUGGCCUGAGGAGCAGGGCUUAGCUGCUUGUGAGCAGGGUCCACACCAAGUCGUGUUCACAGUGGCUAAGUUCCGCCCCCCAGGCCCUCACCUCCUCUGGCCUUGCCGCCUGUCCCCUGCUGCCGCCUGUCUGCCUGCCAUCCU*

*>hsa-mir-3911*

*AUCUGUUUUAUUCAGUUUCUUCAGUGUGUCCAGCAGUUUCUGUGGGGUGAGGAUGUGUGUGGAUCCUGGAGGAGGCAGAGAAGACAGUGAGCUUGCCAGUUCUGGUUUCCAACACUUCCUUUCCUGCGCUUCUCGAUUCCCAGAUCUGCACCCACCCCCCUCCCCAACCCUGGUCUGGGUAUAGUGGAAGCAGGGAGGGG*

*>hsa-mir-24-2*

*CCUUGCCGCCUGUCCCCUGCUGCCGCCUGUCUGCCUGCCAUCCUGCUGCCUGGCCUCCCUGGGCUCUGCCUCCCGUGCCUACUGAGCUGAAACACAGUUGGUUUGUGUACACUGGCUCAGUUCAGCAGGAACAGGGGUCAAGCCCCCUUGGAGCCUGCAGCCCCUGCCUUCCCUGGGUGGGCUGAUGCUUGGAGCAGAGA*

*>hsa-mir-4287*

*UUAAUGAACCGACACUGGGGUCUGGGUGGAGUGGUGCCUUGUGCAGCCUCGCCCAGGUGCUCUCUAGUUCUUUUUCUCCCUUGAGGGCACUUUUCAGUUCCUGAGAUCAAUGUGGUCCCUACUGGGGAGACCAUAGGAGCCCUGCAGGCCAUGACUUUGCUCAAUGCAUGGUAAGCAUGGGCCAUGGGCCAGAGACAUCC*

*>hsa-mir-3140*

*AUAUAGUUAUACUGAGUAUGAAUGUCUUGGUAGGCUUGGAAGCCUACCAUUUAUGUCCUCUUGAGGUACCUGAAUUACCAAAAGCUUUAUGUAUUCUGAAGUUAUUGAAAAUAAGAGCUUUUGGGAAUUCAGGUAGUUCAGGAGUGACUUUUCUAAAAAACAGAACUGAGCACCAUACACUACUUCUUAUAGCCUUUUAA*

*>hsa-mir-615*

*CCUGCGGAGCCGGCUCCGCCGGCGCUUGCGGCUCCGGAGGAUUCCAGCGACUCGGGAGGGGCGGGAGGGGGGUCCCCGGUGCUCGGAUCUCGAGGGUGCUUAUUGUUCGGUCCGAGCCUGGGUCUCCCUCUUCCCCCCAACCCCCCCUCAGCCCCUCCGGCUGCAGAGUGAAGGCUGCGGUGGAAAGUUUCCUGCCUGGG*

*>hsa-mir-744*

*CAGUGCUGCACUGAGCCAGGCGGGAGCUGGAAGAAGACGCAGCACACUGGGUUGGGCAAGGUGCGGGGCUAGGGCUAACAGCAGUCUUACUGAAGGUUUCCUGGAAACCACGCACAUGCUGUUGCCACUAACCUCAACCUUACUCGGUCCUGACCGGCUCGGCUUCUGUUUGUUUAUUUCAUCUCUACUCAGUACUGCCC*

*>hsa-mir-1184-3*

*GGUGAAUGGGAUGGAUCAGACUCCCUGUCCUGAGGGGGAGAUGGUUUCUUGCAGAACGAGGUGAAGGAGGUGGUUCUGCUCAGCAGUCAACAGUGGCCACAUCUCCACCUGCAGCGACUUGAUGGCUUCCGUGUCCUUUUCGUGGGUAGCCAUGACCAAAGACUGGAGCAGCAGAAAGAGCUCCUCGGGAAGCUGGCCGC*

*>hsa-mir-30c-2*

*GUACUGGGUUUCCUGAGGCUUGUGCUGCUGACUGCCAACCCCAUCCUAGAGAGCACUGAGCGACAGAUACUGUAAACAUCCUACACUCUCAGCUGUGGAAAGUAAGAAAGCUGGGAGAAGGCUGUUUACUCUUUCUGCCUUGGAAGUCAACUAAAGAGAAAUGGAUUUUGAAUAUUUCUAUGUCUUAGAAUUGAUCCUAU*

*>hsa-mir-4322*

*CCAGGGGUCCCGCCUCGAGAUUCUGGGAAGACUGGGGGUGGGGGACCAGAUCGCAGCAGCAGCUGCACCGCGAGUUCCGCGCCUGGCCGUGUCGCCCCACGAGGGGGACUGUGGGCUCAGCGCGUGGGGCCCGGAGCAUCUGACAAGGACAGAGACAGAGGAGGGGGUGGAAAUCCCCGGGUGAGUCAACCCGUGCCUGA*

*>hsa-mir-4284*

*CUUGGCUUCCCAAAGUGCUGGGAGUAUAGGUGUGAGCCGCCACCAUGCCCGGCCUGAGCAUGUUCUGUGAGGGGCUCACAUCACCCCAUCAAAGUGGGGACUCAUGGGGAGAGGGGGUAGUUAGGAGCUUUGAUAGAGGCGGCUCAGAGGGGCCUCCUCUCUUUAAGAUAUCGUACCACCUCCCCAGCAGCUAAAAUAAA*

*>hsa-mir-495*

*GACCCUCAGUGUCCCUUCACGCCCAGGUGUGCCUCUGGCAGGGAGGACGUGCUCAUCUCUGGUACCUGAAAAGAAGUUGCCCAUGUUAUUUUCGCUUUAUAUGUGACGAAACAAACAUGGUGCACUUCUUUUUCGGUAUCAAAUAUCUCCUUGAAGUGCCUCCAUCCCCGACGGGACCCUGAGCAUGGGACCUGGUGUCA*

*>hsa-mir-106b*

*CCUUAAAGGGGUAGCUCCUUACCGUGCUCUCAUUGCCGCCUCCCCACCUCCCGCUCCAGCCCUGCCGGGGCUAAAGUGCUGACAGUGCAGAUAGUGGUCCUCUCCGUGCUACCGCACUGUGGGUACUUGCUGCUCCAGCAGGGCACGCACAGCGUCCGUGGAGGGAAAGGCCUUUUCCCCACUUCUUAACCUUCACUGAG*

*>hsa-mir-376a-1*

*ACUUUCUUUUCUGAUGACUCAAGCACAGGUGCACGCUUUCCUGGAUGGAAUCCUUCUUUGGUAUUUAAAAGGUAGAUUCUCCUUCUAUGAGUACAUUAUUUAUGAUUAAUCAUAGAGGAAAAUCCACGUUUUCAGUAUCAAAUGCUGCUUUGAAAACCUCGGAGGACGUGAAGUCGCCAUGGAAGAGGAGAUGUUAUCUG*

*>hsa-mir-1305*

*UGGUUCUGUUCCUUUAGAUAGCUUAACUGAGUACAAAAUUUGGGGUAUAGCAUUUCAGAAGAUCCUGCUGUUUCUACCAUUAGUUUUGAAUGUUUAUUGUAAAGAUACUUUUCAACUCUAAUGGGAGAGACAGCAGGAUUCUCCCAAAAAUUCAAGUAAAAAUCUGCACGAGGAAAAGAAGUCUCCUGAGAUGGAUUGAA*

*>hsa-mir-643*

*CAAAAUUAACCCACAAUUCUAUAGAAGUGUUAGGUAUUAUGUUCGACACACCAAGUGAUAUUCAUUGUCUACCUGAGCUAGAAUACAAGUAGUUGGCGUCUUCAGAGACACUUGUAUGCUAGCUCAGGUAGAUAUUGAAUGAAAAAUCUACACUAGUUUAAAGAAUAUCAUAACUUUUUAUGGAAAAGUAUAAUAAAACU*

*>hsa-mir-302d*

*UAUUGUAUUGACCGCAGCUCAUAUAUUUAAGCUUUAUUUUGUAUUUUUACAUCUGUUAAGGGGCCCCCUCUACUUUAACAUGGAGGCACUUGCUGUGACAUGACAAAAAUAAGUGCUUCCAUGUUUGAGUGUGGUGGUUCCUACCUAAUCAGCAAUUGCGUUAACGCCCACACUGUGUGCAGUUCUUGGCUACAGGCCAU*

*>hsa-mir-520g*

*UAACCUGGUCAAGGAAAAUUCCAACAAGAAACCCAGAGUGCUGGAGCAAGAAGAUCCCAUGCUGUGACCCUCUAGAGGAAGCACUUUCUGUUUGUUGUCUGAGAAAAAACAAAGUGCUUCCCUUUAGAGUGUUACCGUUUGGGAAAAGCAGUGUUGAAGUUGAUGCUGAUGUUGGUAAUAUAUUUGCAUGCUUAUUAUCA*

*>hsa-mir-551b*

*UAUCACCUUCUAGCUAACUGUCCUGACUUCAGACCUCUGUUAGAUCUGCCAGAUGUGCUCUCCUGGCCCAUGAAAUCAAGCGUGGGUGAGACCUGGUGCAGAACGGGAAGGCGACCCAUACUUGGUUUCAGAGGCUGUGAGAAUAACUGCAAUUUAGAGGGAAUACCAUUUAUGUUUAUGCAGGACUUCCAGAGAAAAAA*

*>hsa-mir-1200*

*GAACCAGGUUUCAAAUCCAGAGUUCUGUGACCCCAAAGCCCACAUUCUUCCUAGUUUACUAUGCUACUUCUCCUGAGCCAUUCUGAGCCUCAAUCACUUGCCAGAGAGAUUGGUUCAGGAAUUUGUCAGGGAUAGCCUGAAGUCCCAAAGUCUUCAGCCAGCAUCUGAGCAGUGUUAGAAAAGGCUGAGACUCACACGAU*

*>hsa-mir-329-2*

*CAGGACUGAAUGUCAAGUUUGGGGAAGGAAUCAGUGGUGUUCUUGUCAGUGUUACUUGGUGGUACCUGAAGAGAGGUUUUCUGGGUUUCUGUUUCUUUAUUGAGGACGAAACACACCUGGUUAACCUCUUUUCCAGUAUCAAAUCCCAUCUUGGAGGCCUUCUGGUCCAGACCUCAGCUUCAGGGAAGGGGUGCUGUGGA*

*>hsa-mir-3161*

*CAUUGUCUCUCUAAAGUACCUGAAGCCAGUAGUUCUCAAACUUUGGAGAGGGUUUUGAGUCACCUCGAGAGCUGAUAAGAACAGAGGCCCAGAUUGAAGUUGAAUAGUGCUGGGCCUUUGUUUUUACCAAGUUCCCUGGAUGGUUCUGCUACCUCCUAGCCUGUGAGAACUACUCCUUUGUGAUUUCUUGCCAUUGAAAU*

*>hsa-mir-130a*

*AGCUGAGUGGGCCAGGGACUGGGAGAAGGAGUGAGGAGGCAGGGCCGGCAUGCCUCUGCUGCUGGCCAGAGCUCUUUUCACAUUGUGCUACUGUCUGCACCUGUCACUAGCAGUGCAAUGUUAAAAGGGCAUUGGCCGUGUAGUGCUACCCAGCGCUGGCUGCCUCCUCAGCAUUGCAAUUCCUCUCCCAUCUGGGCACC*

*>hsa-mir-337*

*CUGCCAUCCGAGCGCUUGCACUGCGCUUCCGGGAGGAGGGUUGCGGCCCGUAGUCAGUAGUUGGGGGGUGGGAACGGCUUCAUACAGGAGUUGAUGCACAGUUAUCCAGCUCCUAUAUGAUGCCUUUCUUCAUCCCCUUCAACCACGCGCAGCCCCCGGACCCUCCUCUGCACCCUUGGCUGCACGGGGACGCGUCCUCA*

*>hsa-mir-4325*

*UCGUGUGUGUUAAAAAAGGAGAGUGCAGUGUGGGGAGGGAGAGUGGAGGUAAGGAGAUGGGGAAGAUGUUGCACUUGUCUCAGUGAGAGAUGCUUCUAGAUCCAGGAGGCAGACCUCAAGGAUGGAGAGAAGGCAGAUCCUUUGAGAUUGUUCCUAUUCAAGUAAGCUAUGGUCAAACCUGUUUUGGAAAUGUUGUACAC*

*>hsa-mir-629*

*AGAUGAUGGCACAGUGACAGCUGGGAGGGAUGGGAUGUGCUUGCUUCAUGUCCCUUUCCCAGGGGAGGGGCUGGGUUUACGUUGGGAGAACUUUUACGGUGAACCAGGAGGUUCUCCCAACGUAAGCCCAGCCCCUCCCCUCUGCCUGCCUCAACCCUACACAGUCCUGUCUGGUGACGUGCCAAAGUCCUUCCUGCCUU*

*>hsa-mir-4293*

*AAGGGCCUGACAAGGGCAGAGCAAUUAGUGAAAGUCGUGAUAUUGGUUGCCGGAGGUAUGGCAGAGACACCUGUUCCUUGGGAAGCUGGUGACAUUGCUAAUUCAUUUCACACCAGCCUGACAGGAACAGCCUGACUGAAGCAAGCGGUCAGUGAUGAAGAAGAAAUAGGAUUUCCCCUCUCCCGAGAGAGCCAGUUCCC*

*>hsa-mir-3655*

*GGAAAGGCUGCGAACGCAAAGCAGUGUGGGUUGAUUCUGAGGUGCACUGUGGGAAAGAGCUUGUCGCUGCGGUGUUGCUGUUGGAGACUCGAUUGUUGGUGACAGCGAAAGAACGAUAACAAAAUGCCGGAGCGAGAUAGUAAGGCUCAGGCCAUCCGUUAUUUCUUCCCCAUGGCACUUGGGGCACUUGGCGCAUUAUU*

*>hsa-mir-449b*

*GUUUCAUGUUACAAAUUUAGCCUCAGUGACUGCCUGGGCCACGAGAAUCGGCAGUGACCUGAAUCAGGUAGGCAGUGUAUUGUUAGCUGGCUGCUUGGGUCAAGUCAGCAGCCACAACUACCCUGCCACUUGCUUCUGGAUAAAUUCUUCUUGUCAAUGAAGUGCUCUGGAUACCUGUGUGUGAUGAGCUGGCAGUGUAU*

*>hsa-mir-100*

*AAUGGUUGUAAUAUUUUAUUUUCAGACAUGUCACAGCCCCAAAAGAGAGAAGAUAUUGAGGCCUGUUGCCACAAACCCGUAGAUCCGAACUUGUGGUAUUAGUCCGCACAAGCUUGUAUCUAUAGGUAUGUGUCUGUUAGGCAAUCUCACGGACCUGGGGCUUUGCUUAUAUGCCAUUCAAUAUCCCUUCGAGUUAUUUC*

*>hsa-mir-3622a*

*UUAGUGGCAAGGUCUUGUUUUGUCACCCAGGCUGGAGUGCAGUGGUGUGAUCAUAGCCAAUAGAGGGUGCACAGGCACGGGAGCUCAGGUGAGGCAGGGAGCUGAGCUCACCUGACCUCCCAUGCCUGUGCACCCUCUAUUAUAUCACUCGUAUGUCUACCAUUGUUUGCAGAUCGUGAGCUGCUUGAUGACUGAUGCUA*

*>hsa-mir-200a*

*CCGGUUCUUCCCUGGGCUUCCACAGCAGCCCCUGCCUGCCUGGCGGGACCCCACGUCCCUCCCGGGCCCCUGUGAGCAUCUUACCGGACAGUGCUGGAUUUCCCAGCUUGACUCUAACACUGUCUGGUAACGAUGUUCAAAGGUGACCCGCCGCUCGCCGGGGACACCACCGAGGCACAUCCGGAGCUCCUACUCCAGGG*

*>hsa-mir-7-3*

*UGGGCAGGGGUCUCAGACAUGGGGCAGAGGGUGGUGAAGAAGAUUAGAGUGGCUGUGGUCUAGUGCUGUGUGGAAGACUAGUGAUUUUGUUGUUCUGAUGUACUACGACAACAAGUCACAGCCGGCCUCAUAGCGCAGACUCCCUUCGACCUUCGCCUUCAAUGGGCUGGCCAGUGGGGGAGAACCGGGGAGGUCGGGGA*

*>hsa-mir-892a*

*AUAAAAGAUGCAGGGGACAAUGGUGGGGGAUGGGGCGGCACCUUCACAAUCAGCCAUGCUGUGGGCAGUGCCUUACUCAGAAAGGUGCCAGUCACUUACACUACAUGUCACUGUGUCCUUUCUGCGUAGAGUAAGGCUCACUAAGUACAAGUUUGUGGUGGCCUUCGGACAGGUAAUAGGGUGGAGCUGCAGGAGUUGUC*

*>hsa-mir-4255*

*CAGGAAAGCCUGUGGGAAAGGUGGGCCAUGGAGCCAUCUAAUGUAAGAGGUCAUUGAACUUUAUAGAGCAUCCUUCAGUGUUCAGAGAUGGAGUCAGUAUUGGUCUGGCCAUUUUUAGGGCAAAGAGGCAGCAUCAUGCUGGAAGCAGUAGUCUGCAAUGCUAGGAUGUGACUCCCAAGCCCUUGGUCAUACCCUCUACA*

*>hsa-mir-3156-2*

*UUUUCAGUAAUCUGUGCUUAGAUGAGCUAAAUAUUUAAAGGUUGGAGACUGCCAUGAAGCUCUGCAGAAGAAAGAUCUGGAAGUGGGAGACACUUUCACUAUAUAUAGUGGCUCCCACUUCCAGAUCUUUCUCUCUGUAUAUAUAGUACUUAGAGAAAUCCAACUAUCAGGACUCAGUUUUUCUAGCAGUCUCUCUCCUU*

*>hsa-mir-941-1*

*CCCGGCUGUGUGGACAUGUGCCCAGGGCCCAGGACAGCGCCACGGAAGAGGACACACCCGGCUGUGUGGACAUGUGCCCAGGGCCCGGGACAGCGCCACGGAAGAGGACGCACCCGGCUGUGUGCACAUGUGCCCAGGGCCCGGGACAGCGCCACGGAAGAGGACGCACCCGGCUGUGUGCACAUGUGCCCAGGGCCCGG*

*>hsa-mir-185*

*GGAGACCUGCUGGCUAGAGCUGGGUUGGGGGCCGGUGGGCAGUGGGCCUGGCUCGAGCAGGGGGCGAGGGAUUGGAGAGAAAGGCAGUUCCUGAUGGUCCCCUCCCCAGGGGCUGGCUUUCCUCUGGUCCUUCCCUCCCAAUGACCGCGUCUUCGUCGAGGCCACAGCCCUUGGCUCUGCGCCCACACCUCCAGUGCCAG*

*>hsa-mir-362*

*CUACACAUGCACACAUACAAACACACAAAAAGGGCAGGUGUCAGAGCCUUCUUGUCUGCUCCCCCUCUUGAAUCCUUGGAACCUAGGUGUGAGUGCUAUUUCAGUGCAACACACCUAUUCAAGGAUUCAAAGAGGCUGAGCCUUGUCUACAUGUAGAAGGACCAACAACCUGUCCCCUUCUUUCACAUGUUUGUGUUUGC*

*>hsa-mir-642a*

*CUGGUGGGACCAGGAAGUUCUCAGUCCAUUUCCUAUCUCCUACACUCUCCACAGUUUAUCUGAGUUGGGAGGGUCCCUCUCCAAAUGUGUCUUGGGGUGGGGGAUCAAGACACAUUUGGAGAGGGAACCUCCCAACUCGGCCUCUGCCAUCAUUUAACUCUCCCAGCCUAUCACUCCCAUACUGGAAUUUUCCGUUCCUC*

*>hsa-mir-520b*

*UUUAACCUGGUUAAGGAAGAUUCCAACAAAAAAUCCACGGUGCCACAGCAAGAAGAUGUCAGGCUGUGUCCCUCUACAGGGAAGCGCUUUCUGUUGUCUGAAAGAAAAGAAAGUGCUUCCUUUUAGAGGGUUACCGUUUAAGAAAAGCAACGUUUAGGUGGAUGCUGAUCUUGGCAAUAAUACAUUUGCAGAGCAUGCUU*

*>hsa-mir-106a*

*AUCUGUGAGGACGGAAAAGAAGAGCUCCUGGAAGACUUAAAAUUUUGCUACAGGAAUAGGCCUUGGCCAUGUAAAAGUGCUUACAGUGCAGGUAGCUUUUUGAGAUCUACUGCAAUGUAAGCACUUCUUACAUUACCAUGGUGAUUUAGUCAAUGGCUACUGAGAACUGUAGUUUGUGCAUAAUUAAGUAGUUGAUGCUU*

*>hsa-mir-202*

*GGACCCUCCCCCAGGACAGCCACCUCCCAGUGCUGAAGAGCCGGCCCGCCUCAGAGCCGCCCGCCGUUCCUUUUUCCUAUGCAUAUACUUCUUUGAGGAUCUGGCCUAAAGAGGUAUAGGGCAUGGGAAAACGGGGCGGUCGGGUCCUCCCCAGCGGCACCUGCACCAGUGCACCUGCACCUGCUCCAGCAGCACCUGCC*

*>hsa-mir-4320*

*CCACGUAUGAAUUGAGAGCAGGAAUGUGUGCAAGCUCUUGACUUGUCAAGGCCUUUGCUCACCAGAGUGACAUGUGGGGUUUGCUGUAGACAUUUCAGAUAACUCGGGAUUCUGUAGCUUCCUGGCAACUUUGCCCUGACAGCCCCCACUUUGUGGUCCCCUGUGAUUUGCCCUCUUAGCUCCAUUCUUCACUUUGUGGU*

*>hsa-mir-640*

*AUGAUGGUGAGGAGGUUACAUUGGGAGAAUGAGCCCUGCGUGCUGCCUGUGUGCUCUGUGACCCUGGGCAAGUUCCUGAAGAUCAGACACAUCAGAUCCCUUAUCUGUAAAAUGGGCAUGAUCCAGGAACCUGCCUCUACGGUUGCCUUGGGGAUUCAGAGAUGCUGUGUGAGCAUAGUACCUGGAACAUACUCCUAAGU*

*>hsa-mir-296*

*GGGAAGAUCCUGAGUGAAAUUGUGAAAUCAGGCCCAGCCUCAUGUGGGAGGUGAGGAGAAAGGACCCUUCCAGAGGGCCCCCCCUCAAUCCUGUUGUGCCUAAUUCAGAGGGUUGGGUGGAGGCUCUCCUGAAGGGCUCUGAAGAGCGCUGCACCCGCAGCUGUGUGCCAGGAGUGGAGACAGGACAGUCGAUACAAGAG*

*>hsa-mir-548h-4*

*AGAAAACUUGAAGCCAAACUUGUGAUCCUUCGUUGUUCCAUUUCCAAAGCUAUUAGGUUGGUGCAAAAGUAAUCGCGGUUUUUGUCAUUACUUUAAUUACUUUACGUUUCAUUAAUGACAAAAACCGCAAUUACUUUUGCACCAACCUAAUACUUGCUAUCAUCACUUCUUGUCUAUUUUCCAUUCCUCUUAUAAAUGUU*

*>hsa-mir-3669*

*CGGAAUAUAUAUAUACGGAAUAUAUAUACGGAAUAUAUAUAUACGGAAUAUAUAUACGGAAUAUAUAUAUACGGAAUAUAUAUACGGAAUAUAUAUAUACGGAAUAUAUAUAUACGGAAUAUGUAUACGGAAUAUAUAUAUACGGAAUAUGUAUACGGAAUAUAUAUAUACGGAAUAUGUAUACGGAAUAUAUAUUACGG*

*>hsa-mir-548i-3*

*CCAAACUUUGUUGAUCUUCAUCAAUCCCUCUGAUGCAGAUGGCUCCGAAGUUUACAUCCUAUUAGGUUUGUGCAAAAGUAAUUGCGGAUUUUGCCAUUAAAAGUAAUGGCAAAAAUAGCAAUUAUUUUUGUACCAGCCUAGUAUCUUUUCUCCUUCUACCAAACUUUGUCCCUGAGCCAUCUCAUCACCUAUAACUACCU*

*>hsa-mir-4276*

*UGUGUAUUUUUUCUUAUGUGUUCCUUGAGUCAAUGAGUUAAAUAAUGGGAUAUGCUGUGGGCAUGUCACAGUCUGACUCAGUGACUCAUGUGCUGGCAGUGGCCACGUAAAUAGAGCUACUGUGUCUGAAAGCAAUGGGUAGCUAGAAACCUUUUUGACAGCUCAUAUGAAAACUUAAAGAAUUUCAAAACAGGCUGGGU*

*>hsa-mir-525*

*UUUAACCCAGUGAAGGAAGAUUUCAACCAAAAACCCACGGUGCUGGAGCAAGAAGAUCUCAAGCUGUGACUCUCCAGAGGGAUGCACUUUCUCUUAUGUGAAAAAAAAGAAGGCGCUUCCCUUUAGAGCGUUACGGUUUGGGUAAAGCAACGUUGAAGUUGAUGCUGAUCUUGGUAAUAUAUUUGCAGAGCAUGCUUAUA*

*>hsa-mir-496*

*AGUAUCCCGUCCGUCCUCAGGCCUGCUGCUGGGACGCGCUGGAAGCGAGCACCCAAGUCAGGUACUCGAAUGGAGGUUGUCCAUGGUGUGUUCAUUUUAUUUAUGAUGAGUAUUACAUGGCCAAUCUCCUUUCGGUACUCAAUUCUUCUUGGGAAACGUCACGAGGGGAAAGCCCAGCCGGCACCUGCGCAGGGUAAGGA*

*>hsa-mir-431*

*GUGGGUGGGUGGCUGCUGUGUGGCUGGGUGGGGCCUCCUGCACGUCGUUGUCCUGCUUGUCCUGCGAGGUGUCUUGCAGGCCGUCAUGCAGGCCACACUGACGGUAACGUUGCAGGUCGUCUUGCAGGGCUUCUCGCAAGACGACAUCCUCAUCACCAACGACGUGCAGCUCCAGGUAGCGGUUCUGACGCAGGGCAGGG*

*>hsa-mir-1281*

*GGCCGGUGCGGCGGCGGCGGCGCGGAGCGCGGCGGCAGGAGGAGGGUUCGGAGGGUGGGGGCGCAGGCCCGGGAGGGGGCACCGGGAGGAGGUGAGUGUCUCUUGUCGCCUCCUCCUCUCCCCCCUUUUCGCCCCCGCCUCCUUGUGGCGAUGAGAAGGAGGAGGACAGCGCCGAGGAGGAAGAGGUUGAUGGCGGCGGC*

*>hsa-mir-1972-1*

*CAGACUGGUAUUGAACUUCUGAGCUUAAGCCAACAUCUUGCUUGUCUCCUGAGUAGAGGGACUAUAGGCAUGUGCCACCACACCUGGCUUAAAUGUGUCAUUUAAAAAUUCAGGCCAGGCACAGUGGCUCAUGCCUGUAAUCCCAGCAAUUUGGGAGGCUGAGGCAGGCGGAUCACCUGAGGUCAGGAGUUCGAGACCAG*

*>hsa-mir-3179-1*

*CCUCCCACCUUGGGCUCCCUAAGUGUUGGGAUUACAGGCAAGAGUCACCACGCCCAGCCAGGAUCACAGACGUUUAAAUUACACUCCUUCUGCUGUGCCUUACAGCAGUAGAAGGGGUGAAAUUUAAACGUCUGUGAUCCUGGGGUUGUUGAAGAUGCCACCCAUCUACAUAUUCUUUCAGAUGCACAAUAUUUCACUGU*

*>hsa-mir-524*

*UUUAACCUGGGCAAGGAAAAUUCCAACAAAAAACCCAGAGUUCUGGAGCAAGAAGAUCUCAUGCUGUGACCCUACAAAGGGAAGCACUUUCUCUUGUCCAAAGGAAAAGAAGGCGCUUCCCUUUGGAGUGUUACGGUUUGAGAAAAGCAGCGUUGAAGUUGAUGCUUAUCUCGGUAAUACAUUUGUAGAGCAUGCUUAUC*

*>hsa-mir-764*

*UAUACUCUGCCAUAGUAUAAAGAAUCCUGGUGUGUUCGGGUGAUAUUUUCAAUCUAAAAUCUAGGAGGCAGGUGCUCACUUGUCCUCCUCCAUGCUUGGAAAAUGCAGGGAGGAGGCCAUAGUGGCAACUGUUACCAUGAUUAAUUUCGUUGGGAUAGAAGAUGGACCCGCUGGUUGUAAUCAAUGCAUUGAGGACUCUC*

*>hsa-mir-1913*

*GGCCUGCAGAAGCUGGGCACACCCUGCUGUUCCUUCUCGGCCGUGGGCAGCGCAGUUGCAAACCUCUACCUCCCGGCAGAGGAGGCUGCAGAGGCUGGCUUUCCAAAACUCUGCCCCCUCCGCUGCUGCCAAGUGGCUGGUGUCAAGUCCCAUCCCACGUGUCAUUUACAUUACUUGCUAGGGUGCUUUUCUGAAGAAAU*

*>hsa-mir-4297*

*CGGAUGGAGGAGCCACAGAAAACGCUUUUAUUCAGCCUCCUUGUAAAGGCCACAAACAGUGUUGUCAGCACGCACGUGCCUUCCUGUCUGUGCCUGCCUUCGAAGUGCACGGCAGGGCCAGGACGGGUCGCUGUGGGUGGGGACUGGUCUCCACACCCGCAGACGGAACUGGUACUGAAGGGCAGUGAUAACUGGCCGGU*

*>hsa-mir-520e*

*CAAGGUUUUAGAAUCCAAGGAAACCAAUAAACACCCAGAGUGCUGGAGCAAGACUGUCUCCUGCUGUGACCCUCAAGAUGGAAGCAGUUUCUGUUGUCUGAAAGGAAAGAAAGUGCUUCCUUUUUGAGGGUUACUGUUUGAGAAAAGCAACCUUGAGGUUGAUGCUGAUGUUUGUAACACACCUGCAGAGUAUACUUAUA*

*>hsa-mir-3195*

*GGGGCGCCGCCUGCACCACCCUCUUGGGCGACUCGGCCUUGACCCCCGGCGCCGGCGCCGCAGCCGCCGCGCCGGGCCCGGGUUGGCCGCUGACCCCCGCGGGGCCCCCGGCGGCCGGGGCGGGGGCGGGGGCUGCCCCGGCGCUGCCCCCGUUCUGGGCGGCGGCGGGGGGCGGCACGGCGGGCGCGGCGGUCGGGGGU*

*>hsa-mir-526b*

*UUUAACUUGGUCAAAGAAAAUUCCAGGAAAAAAUCCACGGCAUCAGAGCAAGAAGAUGUCAGGCUGUGACCCUCUUGAGGGAAGCACUUUCUGUUGUCUGAAAGAAGAGAAAGUGCUUCCUUUUAGAGGCUUACUGUCUGAGAAAAGCAACGUUGUAGUUGAUGCUGAUCUUUGUAAUAUCUUUGCAGAGCACGCUUAUA*

*>hsa-mir-3928*

*CAGUCACAUGACGCGGAGCCGCCCCUCUCGGAGGGACUUCCGGCCCCAACCGGAAGAGGUUAAUUUCCAUGGCUGAAGCUCUAAGGUUCCGCCUGCGGGCAGGAAGCGGAGGAACCUUGGAGCUUCGGCAGCUUUUCAAAGAGCUUUGGGUUCGGGGCUCCUAAAAGAAAAAAACUGAUUCCGGCCGGGCGCGGUGGCUC*

*>hsa-mir-3150b*

*GCCAGCUUGGCCGCCUCCCUGCAGAGGGCUGACGCUUCUCCAGAAGUUGUAAGAAAAGGAGGGAAAGCAGGCCAACCUCGAGGAUCUCCCCAGCCUUGGCGUUCAGGUGCUGAGGAGAUCGUCGAGGUUGGCCUGCUUCCCCUCACUCCUCGGGCCUUGCUCUUCCCCCAGGUGCCCACUGCCCUCCCUGCUGUGUACCA*

*>hsa-mir-518b*

*UGGUCAAGGAAAAUUCCAACAGCAACAUCAAAAAACCAGUGUUGGAGCAAGAAUAUGUCAUGCUGUGGCCCUCCAGAGGGAAGCGCUUUCUGUUGUCUGAAAGAAAACAAAGCGCUCCCCUUUAGAGGUUUACGGUUUGAGUAAAGCAGCGUUGAAGUUGAUGCUGAUCUUGGUAAUACAUUUGCAGAGCGUGCUUAUCA*

*>hsa-mir-330*

*CUUGGUGACUCCCUUCUUCCAGGAUCGCGUCCCUGCCACUUCGUGCUGUGUGAUCUUUGGCGAUCACUGCCUCUCUGGGCCUGUGUCUUAGGCUCUGCAAGAUCAACCGAGCAAAGCACACGGCCUGCAGAGAGGCAGCGCUCUGCCCCUUACUCGGCCCCGUUUUCAUCGGAGACCUCCGGGGAGCGGUGGGGGUGGAG*

*>hsa-mir-1227*

*AGCGAGGAGCAGUGUCAGGAGUGGAUGGAGGCUCUGCGUCGGGCCAGGUGGGGCCAGGCGGUGGUGGGCACUGCUGGGGUGGGCACAGCAGCCAUGCAGAGCGGGCAUUUGACCCCGUGCCACCCUUUUCCCCAGCUACGAGUUCAUGCGGAGAAGCCUCAUCUUCUACAGGAACGAAAUCCGGAAGGUGACGGGCAAGG*

*>hsa-mir-145*

*ACUCCAGCUGGUCCUUAGGGACACGGCGGCCUUGGCGCUGAAGGCCACUCGCUCCCACCUUGUCCUCACGGUCCAGUUUUCCCAGGAAUCCCUUAGAUGCUAAGAUGGGGAUUCCUGGAAAUACUGUUCUUGAGGUCAUGGUUUCACAGCUGGAUUUGCCUCCUUCCCACCCCACAGUUGCCCCCCAAUGGGGCCUCGGC*

*>hsa-mir-4300*

*AGUAAGAUGAUGUCAUAUAUGCUGGGGUACAAAAAAAAAACCCCAGUGUUUUCUAUGAGUUUAGAAGAGGGCCAGCUAAAUCAGCAGAGACAUGAGGUGAUCAAAAACCUUUUUUCAAAGCAGUGGGAGCUGGACUACUUCUGAACCAAUAUGGCCUAUGCUAAUGUAACUUAAAGAUAACCACCUGGCUCAAACAUAAA*

*>hsa-mir-320b-2*

*AUUGUUAGUGCCAUUUUUAUUGUAAAUAUCAGAAUUGUUAUUUUUUGUCUUCUACCUAAGAAUUCUGUCUCUUAGGCUUUCUCUUCCCAGAUUUCCCAAAGUUGGGAAAAGCUGGGUUGAGAGGGCAAAAGGAAAAAAAAAGAAUUCUGUCUCUGACAUAAUUAGAUAGGGAACCAGUUGGGAAGCUGUAAGAAUAAUGC*

*>hsa-mir-148b*

*CUAAGUCACCCAAUCUCCCACAAAACAAUCUGCCUAUACAUCAUUUCCAAGCACGAUUAGCAUUUGAGGUGAAGUUCUGUUAUACACUCAGGCUGUGGCUCUCUGAAAGUCAGUGCAUCACAGAACUUUGUCUCGAAAGCUUUCUAGCAGCUACCCAUUUUGGGAGUGGGAGGGAAGAAUAGACCUUUUAAAUUCUUUCA*

*>hsa-mir-4257*

*GAGUGAGAUCUCCAUCUCAAAAAAAAAAAAAAAGCCUUUUGUGUGUGCCUGAGCCGGCUUAGAAACAGUCCCUAGGUAGGAUUUGGGGAGGAGCUAAGAAGCCCCUACAGGGCCCAGAGGUGGGGACUGAGCCUUAGUUGGAGGGCUGAGGUCAGCCCCUGACCAUGUAGCCUCUACAGAUGGACAAGGCAGUGAUCCUC*

*>hsa-mir-372*

*CUAUGGCCGUUUCCUCGUGAUAUAAAUUUCUUGGCCGGGGCUCUUGCAGAUGGAGCUGCUCACCCUGUGGGCCUCAAAUGUGGAGCACUAUUCUGAUGUCCAAGUGGAAAGUGCUGCGACAUUUGAGCGUCACCGGUGACGCCCAUAUCAACGGAUGCCGUGGAGCUCGGUCUUCUGCAGGAACUAAAGAGCCUGUGGUU*

*>hsa-mir-2113*

*UCUUAAGAGUAAAACUGACGGAGAGAAGCAGUAUACCUUUCACAGAGGCAGAUAUUUUCAAAGCAAUGUGUGACAGGUACAGGGACAAAUCCCGUUAAUAAGUAAGAGGAUUUGUGCUUGGCUCUGUCACAUGCCACUUUGAAAACCUACCCUCAAACUGUCUAUAAAUUUUCAUGCCCAUCUUUUUCUCCUUACUCUUU*

*>hsa-mir-30d*

*CUGAAGAUGAUGACUGGCAACAUUUAUGUCUGUUCCUCCUCUUAAAUUUCUUGUUCAGAAAGUCUGUUGUUGUAAACAUCCCCGACUGGAAGCUGUAAGACACAGCUAAGCUUUCAGUCAGAUGUUUGCUGCUACCGGCUAUUCACAGACAUCCUCUUGAUAUAAUUCUGUCCCGGAGUGGAGUUGAGGAGGCUAUAAAA*

*>hsa-mir-1301*

*UGCAAACAGCCUUUCCACUGACGCAGUGCCUUGGGGGCUCUGCCAAGCGACCCCUAGAAUGGGGAUUGUGGGGGGUCGCUCUAGGCACCGCAGCACUGUGCUGGGGAUGUUGCAGCUGCCUGGGAGUGACUUCACACAGUCCUCUCUGCCUCCAGGGUCACCCGGAACGGGGUUGCGGGGGACAAGCUGUGGGCGGGGGG*

*>hsa-mir-3116-1*

*UACUCAUGUCUUCAGUUUUGCUUUUCUUUGUUCUUUCUUUCUUGUUCAUUCAUUCAUCAAGCACUUUAUUGAGUCCCUACUAUGUUCCAGGCACUGGGUAUCGUAGGUGCCUGGAACAUAGUAGGGACUCAAUAAAGGAGCUAUGCUUCCUUUAUGUGAAUUAAAUGUCACUGAUGUAGCUGGCAUUUAGUAGGCUUUCA*

*>hsa-mir-654*

*UGGGAUCAUGGCUGGACUGAAGCCACCAUCAGGGACGUGAGGUGGGGCUGAGCCCUCCAGGGUAAGUGGAAAGAUGGUGGGCCGCAGAACAUGUGCUGAGUUCGUGCCAUAUGUCUGCUGACCAUCACCUUUAGAAGCCCCCUUUCGCUGAGGAGCCAGCUUGGUGCACAGGCUGUGGUGUGUUGUCACAGCUAUGCUAC*

*>hsa-mir-720*

*UCAGGUGGCGCCCCGCCUCCGAGCCGGCCGUCCUUCCGGGGGAGCCUGGAGCGAGACCGGAUCUCACACGGUGGUGUUAAUAUCUCGCUGGGGCCUCCAAAAUGUUGUGCCCAGGGGUGUUAGAGAAAACACCACACUUUGAGAUGAAUUAAGAGUCCUUUAUUAGCCGGCGACCGAGAGACCGCUAACACUCAAAAUUC*

*>hsa-mir-217*

*GAGAAUAAAGUUGUUGCAUUUGGAAAUAAAUGAUAUUUAGUAUAAUUAUUACAUAGUUUUUGAUGUCGCAGAUACUGCAUCAGGAACUGAUUGGAUAAGAAUCAGUCACCAUCAGUUCCUAAUGCAUUGCCUUCAGCAUCUAAACAAGCACCAGUAAAUACCUCAAGUUAGAUUUGCUGUGAUGUGUUCAUGGUCAUCCA*

*>hsa-mir-3605*

*GAUGACAUGGGGGAAGGUUUGAGGAGGGCUGGCCUCUGUGCCUGGAUACUUUAUACGUGUAAUUGUGAUGAGGAUGGAUAGCAAGGAAGCCGCUCCCACCUGACCCUCACGGCCUCCGUGUUACCUGUCCUCUAGGUGGGACGCUCGUCCCUGCUGGUGGGGAAUCUCAAGAAGAAGUAUGCACAGGGGUUCCUGCCUGA*

*>hsa-mir-3118-4*

*UUGCCUCUCCCAGGACCCCAGCCUGGCCACAUCUGCUUACAGGGCACUCUCAGGUGCCCACACAUACUACAAUAAUUUUCAUAAUGCAAUCACACACAAUCACCGUGUGACUGCAUUAUGAAAAUUCUUCUAGUGUGAUUUACAGCUCUGUCAGGUCAGUUAUUUUCUUCUUUAUACUUGCUAUUUUGUCUGUUAGUUCC*

*>hsa-mir-99b*

*GCUGAGGGCCUGGACUCCUGGGUUCCUUGGGGAGGAGGGGCCGGGGGCCCGGACUCCUGGGUCCUGGCACCCACCCGUAGAACCGACCUUGCGGGGCCUUCGCCGCACACAAGCUCGUGUCUGUGGGUCCGUGUCGGGGGCUCACCAUCGCGGCUGGGGCCUCCCCGGCCCUCCCCCUCAUCCCUGGUCCUCCUGGUCCC*

*>hsa-mir-624*

*UGUUCUUGUAAUGAAAAAGAAAAAAAGGUUAAAAAAGGAUGUGCUUUUGACCAAUAUAUAAUGCUGUUUCAAGGUAGUACCAGUACCUUGUGUUCAGUGGAACCAAGGUAAACACAAGGUAUUGGUAUUACCUUGAGAUAGCAUUACACCUAAGUGGUUUCAGUGAACAAGAUUUAAAAGACUAGAAUUAAAGCAAUUAU*

*>hsa-mir-633*

*GUCCUGGCUUAUUGCUUACCUGAUUAAUGGUUUUGGUUAUGCUACUCAACCUCUCUUAGCCUCUGUUUCUUUAUUGCGGUAGAUACUAUUAACCUAAAAUGAGAAGGCUAAUAGUAUCUACCACAAUAAAAUUGUUGUGAGGAUAAAAAAAAAUAUUAAUUAGGAUGCAGUAUGAAAAAAAUAAAAGUUAGCUAUCUUUA*

*>hsa-mir-3935*

*ACAGAACAGGCAUUGCCCUAAGUAUUUGGGGGUAUGAAAGAAGCUGAUGUGGAUGUGUUCCUGUCCCAGAAGGAGCUGAUGGUUGUAUCUAUGAAGGUAAGCAUUUUUGUAGAUACGAGCACCAGCCACCCUAAGCAAAGGCAGAGAAUGCUUAGGCCAGACAGAGGGCUAGGGCACUUGAAUAGAAAGAGAGCAAUUCU*

*>hsa-mir-506*

*CAUCUGUAGUUUCCCCGUGUUACUAUUAAGAUGGUCAGUGCCACCACCAUCAGCCAUACUAUGUGUAGUGCCUUAUUCAGGAAGGUGUUACUUAAUAGAUUAAUAUUUGUAAGGCACCCUUCUGAGUAGAGUAAUGUGCAACAUGGACAACAUUUGUGGUGGCAUUGGUAAUGACGAGUGGAAUUUCAGUGACAAUACUG*

*>hsa-mir-4294*

*UUCCUCUGCAGCCCCAUCCUGGCUUGACUGCUCCCCUGCCCUCGCCUCCAGCAGCGGACUCUGCUUCCGAUGCCUCGGGAGUCUACAGCAGGGCCAUGUCUGUGAGGGCCCAAGGGUGCAUGUGUCUCCCAGGUUUCGGUGCAGAGCGGCUUCCCUCCUCCCAGCAAUGCGAGCUAAUCCUGGAGGACCCCACUGGAGCA*

*>hsa-mir-378c*

*GGAGCCUCCCCUCCUGCUGUAGCUCCCAGGGCCACCCACGGAAUCCUCCACCCUCCACUCGGAGGCCAUCACUGGACUUGGAGUCAGAAGAGUGGAGUCGGGUCAGACUUCAACUCUGACUUUGAAGGUGGUGAGUGCCUCAAACUCAAACGUUCAUGCAUGGUGAGGAUAAAAAGUAACUGCCCUGGGAGGAUGAAACC*

*>hsa-mir-4282*

*AAGUUUGAGACCUGCCUAGACAACAUAGUGAGAACCUACUGAGACAUCUUUAACUUCUUAGGUGUGAAUGGUGAAGUUCCAGGGGAAGAUUUUAGUAUGCCACAUUUCUAAAAUUUGCAUCCAGGAACAUCAUCCUAUUCACCUUCUGACUGAUAUCUUAAGUGACCUUAAUGGUUUUUAGAACCUUGUAUGGAAAACUC*

*>hsa-mir-601*

*AGUCUCUAUCCAGACAACUCAGCGGCUACACGCUGACUUCCUGUGAUGCCUUGAAAAAGUGCAUGAGUUCGUCUUGGUCUAGGAUUGUUGGAGGAGUCAGAAAAACUACCCCAGGGAUCCUGAAGUCCUUUGGGUGGAAAGGGGGAGGCCCCCAUGUCUGUCUGGCUCUCAGAAAAUGGGAAAGAAGGUAAGGGACAUGG*

*>hsa-mir-378b*

*AUUUUCUGUCUCAGUUGUGAACCACACGUCCUGGGGCCUCAGUUUCCUCCGCUGAAGAAUGGGAGGAGGCUGGUCAUUGAGUCUUCAAGGCUAGUGGAAAGAGCACUGGACUUGGAGGCAGAAAGACCCAACUUGCAUCCUGGCUCUGCCACAUCUGGUGCCUUGACUGUGGACAAGCCACACUAGCUCUCUGGUUGUCC*

*>hsa-mir-3651*

*CCCUGCCCUGUGGUUGCUGGAUGCUGUUGUGCAUGGACAGCUCUCCAGUGGAUUCGAUGGGCCAUAGCAAUCCUGUGAUUUAUGCAUGGAGGCUGCUUCUCCUCAGCAGCUGCCAUAGCCCGGUCGCUGGUACAUGAUUCUUCCUGAAUAGUCAAUGCUGUUAUACUGAGUGUUUAUGCUUGACGUAAUAAUGCAGUGAU*

*>hsa-mir-3125*

*UUCCAUAGCUAUUCUGCUAAAACAGUGAAAUUGGAGAUGAGGAGUCUGGCUUGCGCUGGGUGAGAAUGGGUAGAGGAAGCUGUGGAGAGAACUCACGGUGCCUGUGGUUCGAGAUCCCCGCCUUCCUCCUCCUUUCCUCUGCCCCUUGGGUUUCACCUUUUCACCUCCGUCACAUCUCGACAGCUAGUAAGUCUCGUGGG*

*>hsa-mir-3915*

*UAUGAGGAAGGGAUGAAUGUCCAUUUUUUCCCAUAUAGAUCCAACUGAAACAACAAGUUGGCACUGUAGAAUAUUGAGGAAAAGAUGGUCUUAUUGCAAAGAUUUUCAAUAAGACCAUCCUUUCCUCAAUAUUCUGUGGUGUCAUCUUUGACGAAAAUAUUAGAAAUAAAAUGCCUAUACGUGCAUGGAUCAGUUUAUGG*

*>hsa-mir-3193*

*CCAGACUCGGCCUCCUCACUCUGGCCUGGCUCUGUCGGCAGUUCAAACAAAUCUCCCCCACCAACCCCGGGGCUCCUGCGUAGGAUCUGAGGAGUGGACGAGUCUCAUUACCCAGCUCCUGAGCAGGAAAAAUAUGUCAUUGGCAAGGGUCUAAGAAUCUUCCUGGACAUGUAUGUGGAUUUCUACUCAGGGGAGCAUGU*

*>hsa-mir-3939*

*CUUAGGAGACGCCUGCCGGCCGCUUUGGGAUAGGGCCUGUCUGCCCAUGCUGGCUUCCAAAGGCCUCUGUGUGUUCCUGUAUGUGGGCGUGCACGUACCUGUCACAUGUGUACGCGCAGACCACAGGAUGUCCACACUGGCUUCCAAACACAUCUCUGUGUUUCUGUCUGUGAGUGUGUACGCAGGUGUCAAAUGUGUAC*

*>hsa-mir-635*

*CCGGAAGUAGUGUUUGGGCCAAGACUUGGAGCAUCAGCUCCUAGAGCCAGAGAGGAGCUGCCACUUGGGCACUGAAACAAUGUCCAUUAGGCUUUGUUAUGGAAACUUCUCCUGAUCAUUGUUUUGUGUCCAUUGAGCUUCCAAUUGUAAAAGGAUGGAGUGCAGGUCUGGCUUCUUUCUCCCGCAGAUCCGUGGAACCC*

*>hsa-mir-2116*

*UUGGCUUCCUCUCGGCAGAAGGGCUCCUCUGCCGGCCGUCAGCAUCUCCUCCGUCAGUGGGACCUAGGCUAGGGGUUCUUAGCAUAGGAGGUCUUCCCAUGCUAAGAAGUCCUCCCAUGCCAAGAACUCCCAGACUAGGAGUCCUCCCAUGCUAAGUCCUCCCGAAGUCCUCCCAUGGUAAGAACUCCUAGACUAAGUUC*

*>hsa-mir-454*

*UAGAUUAUCAUAAUUGCUUAGUGAAUUUCUGCCAUCUAACUAAAAAAAAUUCUGUUUAUCACCAGAUCCUAGAACCCUAUCAAUAUUGUCUCUGCUGUGUAAAUAGUUCUGAGUAGUGCAAUAUUGCUUAUAGGGUUUUGGUGUUUGGAAAGAACAAUGGGCAGGCUGCAGAAAGUAUGUAGCAGAAACAAGCAAAAGUC*

*>hsa-mir-3196*

*GAGUGACGGGCACGUGACAGGGUGUGACGCUGCUGCCUGGGUUGGCUUCAGGGGGCUGUGCCAUGUGAGGGUGGGGGCGGGGCGGCAGGGGCCUCCCCCAGUGCCAGGCCCCAUUCUGCUUCUCUCCCAGCUUACCCCUUGGGCACAGCCCAUUGCCUGUCGCCAUACCACCCCCAACAGUGCCCAGCACACAGCAUCUG*

*>hsa-mir-186*

*CCAUUAUAAUUAAGGUUUACAGAACACCCAUCAUAUUCUUCCCAAACAUUUUUUCAUUGCUUGUAACUUUCCAAAGAAUUCUCCUUUUGGGCUUUCUGGUUUUAUUUUAAGCCCAAAGGUGAAUUUUUUGGGAAGUUUGAGCUAAAUUCCUUCAACCAAAAUAUACAAGUGAAGAAAAAAAAUUUGUAUUUAAACAUUUG*

*>hsa-mir-548w*

*GGACAAAUUUUUCCAAGAGGAAAGGACUGUAACAUCACCUGAUGUUAAGAUAGGGCCUAGUUAGGUUGGUGCAAAAGUAACUGCGGUUUUUGCCUUUCAACAUAAUGGCAAAACCCACAAUUACUUUUGCACCAAUCUAAUAGGUUGAUUGCCAAGUCUCCUUGCACUUUCCAUCAUCUAUUACCGUUUCAUUCGUACGC*

*>hsa-mir-650*

*GCAUGGAGGUCCCGCCCUUCUCUGAGGCAGAGGGAUAAGACAGGGCUGGGGGCAGGCCCAGUGCUGGGGUCUCAGGAGGCAGCGCUCUCAGGACGUCACCACCAUGGCCUGGGCUCUGCUCCUCCUCACCCUCCUCACUCAGGGCACAGGUGAUGCCUCCAGGGAAGGGGCCACAGGGACCUCUGGGCUGAUCCUUGGUC*

*>hsa-mir-517c*

*UAAAUAAAUAAAUACAUAAAUACAUAAAAAAUAACCCAGAGUACUGGAGCAAGAAGAUCUCAGGCAGUGACCCUCUAGAUGGAAGCACUGUCUGUUGUCUAAGAAAAGAUCGUGCAUCCUUUUAGAGUGUUACUGUUUGAGAAAAUCAACGUUGAAGAUGCUGCUGAUCUUGGUAACACAUUUGCAGAGCGUGCUUAUCA*

*>hsa-let-7i*

*GAGCGCCGGAGCCGCUUUGCUGCCCUCCGCGUGGUCCCGUGCCUCCCCGACACCAUGGCCCUGGCUGAGGUAGUAGUUUGUGCUGUUGGUCGGGUUGUGACAUUGCCCGCUGUGGAGAUAACUGCGCAAGCUACUGCCUUGCUAGUGCUGGUGAUGCUCAGCGCCGCGGAGGACAAUGGCUGGGAAUCCCCUUUGUUUUC*

*>hsa-mir-885*

*UGCCACCGUCUCCAGGGAGCCUAGGACGACACCGUGACUGCAGAAGGCCCAGUCUGCUACUCGGCCCGCACUCUCUCCAUUACACUACCCUGCCUCUUCUCCAUGAGAGGCAGCGGGGUGUAGUGGAUAGAGCACGGGUUCAAGUCCCGGCUCCACCACUCACAGCUGUGUGACCCUGGGCCAUCACUCAAGCUCUCUGA*

*>hsa-mir-1205*

*CUUCUGGAUGGAUGCACCACGAUAGGGAAAUCAGCAAGCUGAAGAUAGGGAAAGUGAACACUUAUCACUGAAGGCCUCUGCAGGGUUUGCUUUGAGGUACUUCCUUCCUGUCAACCCUGUUCUGGAGUCUGUCUCCAUUUUUCAGACACAAAAACUGAGGCAUAGAUAACUGUUUUCUGUUUUUUGUGGGGAGAGAGUAU*

*>hsa-mir-4278*

*CUCUGUGAUACACAGGAGUGAAACGUUACUUAGCAGAUGCAGCCUCACGUUUCCGUGAUACUGUAAUCAUCUAACACCAGGAGAAUCCCAUAGAACAUUGACAUCAACACUAGGGGGUUUGCCCUUGUGGGGAAGAAUCUAAGGUAACAUCAUGAGGACUCUAAUAAAUGCAAUGGUGUUUAAUUAUGGUUUAGGGCUCU*

*>hsa-mir-3117*

*UCAGUUUCCACACCUGUAAACAACAACAACAAAGAAAUUUACACUAGAUGGAAUCGAAUUUCCCUAAAGGGCCAGACACUAUACGAGUCAUAUAAGGGAAGGCAUUAUAGGACUCAUAUAGUGCCAGGUGUUUUGUGGGACACUAUUCGAUGGAUCUCAGGAGAACAUCGUUAAUGGAAACCAAAUCCAUUUCUGAAUGA*

*>hsa-mir-4309*

*GACAGAAAGGCCCUACGUGGCCCCUGAGCAGCUCUGGGGAACAUUCUUGGUCCGGGUUCCUGUCUGGGGGUUCUGGAGUCUAGGAUUCCAGGAUCUGGGUUUUGAGGUCUUGGGUUGUAGGGUCUGCGGUUUGAAGCCCCUCUUGUAACCCAGGACAGAGGUGAAAGCAGUGGGGUGGACUGGGCAGCCCCCAAAGUUCA*

*>hsa-let-7a-2*

*ACUAACUUGUAAUUUCCCUGCUUAAGAAAUGGUAGUUUUCCAGCCAUUGUGACUGCAUGCUCCCAGGUUGAGGUAGUAGGUUGUAUAGUUUAGAAUUACAUCAAGGGAGAUAACUGUACAGCCUCCUAGCUUUCCUUGGGUCUUGCACUAAACAACAUGGUGAGAACGAUCAUGAUUCCUCCAGGCCUUUUCUCCCUAUG*

*>hsa-mir-891a*

*AUUAUAAUUACGUAGCUUCUUUGUUUUUUUCUAGGUUCCCAAAGAGUCUACAAAUGUUGUCUCCUUAAUCCUUGCAACGAACCUGAGCCACUGAUUCAGUAAAAUACUCAGUGGCACAUGUUUGUUGUGAGGGUCAAAAGAGAUAAUCUCUACAGGCCUCAUGGACACUGCAAACCAUGUAAGUCAUCAGUUAUGUAGCU*

*>hsa-mir-29b-1*

*AGGUUGUCUUGGGUUUAUUGUAAGAGAGCAUUAUGAAGAAAAAAAUAGAUCAUAAAGCUUCUUCAGGAAGCUGGUUUCAUAUGGUGGUUUAGAUUUAAAUAGUGAUUGUCUAGCACCAUUUGAAAUCAGUGUUCUUGGGGGAGACCAGCUGCGCUGCACUACCAACAGCAAAAGAAGUGAAUGGGACAGCUCUGAAGUAU*

*>hsa-mir-1202*

*CAAAGUCAGCUGGUGGUAGUGAUGGCAGCAGCAGCCCAUCUGGAGCACCUGCUGCAGAGGUGCCAGCUGCAGUGGGGGAGGCACUGCCAGGGCUGCCCACUCUGCUUAGCCAGCAGGUGCCAAGAACAGGUGGGAGCCCCACCCCCUACUGAGUUGGCAGGAUGGGAGCCCCUUGCUCCCUGGCACACAGCUACAGCUGC*

*>hsa-mir-507*

*GACUGAGAGUGUUGGGAUUCUGACUGAAAUGGCGGAGUGAGGGACAUUUUCCGCUGUGCUGUGUGUAGUGCUUCACUUCAAGAAGUGCCAUGCAUGUGUCUAGAAAUAUGUUUUGCACCUUUUGGAGUGAAAUAAUGCACAACAGAUACAAGUCAGAACUACCCACUCAGUGAUGGGUCAACUCAUACAACCCUUUAAAG*

*>hsa-mir-935*

*UCGAGCGCAGCCGCGAGGCGCACUGCCAGUCUCGCUCGGACCUGCUCAAGGCCGGCGGGGGCGCGGGCGGCAGUGGCGGGAGCGGCCCCUCGGCCAUCCUCCGUCUGCCCAGUUACCGCUUCCGCUACCGCCGCCGCUCCCGCUCUAGCUCCCGCUCCAGCGAGCCGUCGCCGUCGCGGGACGCGUCUCCCGGCGGCCCC*

*>hsa-mir-511-2*

*AAGUAUAUUUGCUUUAUCAACAUUCUAAGUAUUGGAAUGAUCCAACGAUAAGAAAACAAUAGACACCCAUCGUGUCUUUUGCUCUGCAGUCAGUAAAUAUUUUUUUGUGAAUGUGUAGCAAAAGACAGAAUGGUGGUCCAUUGAAUCCCCAGGAUGAAGACAUGAGGAGACGUGGGAGGGAUGCUAUCUGCUGGGAAUAG*

*>hsa-mir-3200*

*AAGUGGAGGUACGGCGUGACCUGGAUGAGGCAUCAUCGAGAGAGACAAAGAAGGAGGUGGUCGAGGGAAUCUGAGAAGGCGCACAAGGUUUGUGUCCAAUACAGUCCACACCUUGCGCUACUCAGGUCUGCUCGUGCCCUCCCAUGAUAUCAGCUCUCCUACUUCAAUGUGGGAAUCUCCGUUUUCCUUGGAGAACAAAA*

*>hsa-mir-377*

*CAGGCAAUGCCGCCUUUGGUGAAGAGGCAUCUCGGUGUGUUCUUGCCCGUGCUGAUGUUUGACCCUUGAGCAGAGGUUGCCCUUGGUGAAUUCGCUUUAUUUAUGUUGAAUCACACAAAGGCAACUUUUGUUUGAGUAUCAAAUCCUGCUUGGGAUGGCUUCCGGGACCCAGUGGCAAGCUCAGGGGCAUCUACACCCCU*

*>hsa-mir-582*

*ACAGAGCCCUGCUGAACACCACUAGCAAAUCUCUCUCUAGUAGACCACAACAAGUCAAUCUGUGCUCUUUGAUUACAGUUGUUCAACCAGUUACUAAUCUAACUAAUUGUAACUGGUUGAACAACUGAACCCAAAGGGUGCAAAGUAGAAACAUUUCAUUGUGAAGUCGGCUGGGGACAAAGAAAACACCACCAGAACCC*

*>hsa-mir-4268*

*UUGUAUCUGUACUGGACAUGAUUAACUUUCAAGUUCAUAGAAAUCGUGCAACUCUGGUUUCCCUGGCAAAAUGCACAUCAGGUUCUAGAGGUUUUGCCCUAGCGGCUCCUCCUCUCAGGAUGUGAUGUCACCUGGGGGCAUCUUCACUGUGUGACAUCAUUUGUUAUGCAAUGGUGAGUUUGAGGUGCUGGGAAUAUUCU*

*>hsa-mir-937*

*AGACACUCUGGGGGCUCUGCCCAAUCUUCGGGAGCUGUGGCUUGACCGGAACCAGCUGUCAGCACUGCCCCCGGUGAGUCAGGGUGGGGCUGGCCCCCUGCUUCGUGCCCAUCCGCGCUCUGACUCUCUGCCCACCUGCAGGAGCUCGGGAACCUGCGGCGCCUGGUGUGCCUGGACGUGUCGGAAAACCGGCUGGAGGA*

*>hsa-mir-448*

*UUUCAGCAGAAACUGGAACUUCAUGGUGCAGCAGUUCAUCUUCGCAUGGGCCGGGAGGUUGAACAUCCUGCAUAGUGCUGCCAGGAAAUCCCUAUUUCAUAUAAGAGGGGGCUGGCUGGUUGCAUAUGUAGGAUGUCCCAUCUCCCAGCCCACUUCGUCAUGACAGACACCAGAAACACCACUCUGCUUCACUGAAGAAC*

*>hsa-mir-3142*

*AGUUUCUUAACUUGCCAGCUGUGUGGCCUUUCUGAACCUUCAGAAAGGCUGCUGAAUCUUCAGAAAGGCCUUUCUGAACCUUCAGAAAGGCUGCUGAAUCUUCAGAAAGGCCUUUCUGAACCUUCAGAAAGGCUGCUGAAUCUUCAGAAAGGCCUUUCUGAACCUUCAGAAAGGCUGCUGAACCUUUCUGAGCCUAAUUU*

*>hsa-mir-1185-2*

*GAGAUGUCCACUCCGGCAAAGACCCGGCAUGGUCCUCACCUGCUCUUUGAGCCACCCUUUGGUACUUAAAGAGAGGAUACCCUUUGUAUGUUCACUUGAUUAAUGGCGAAUAUACAGGGGGAGACUCUCAUUUGCGUAUCAAACUGUCGGAGAAGACACUCAUACCCUGCUUGUCUCCAGGACAAAGCCUCGGAUGUCAG*

*>hsa-mir-4324*

*CAACUUCUGUGUCCUGGGCUUCUGGGCGACAGUCAUCACACAUCGCUGCUGUGAGAGGUAAGGCCGGCCCCUUUGUUAAGGGUCUCAGCUCCAGGGAACUUUAAAACCCUGAGACCCUAACCUUAAAGGUGCUGCAAAGACCCAUGUUCACACAUUUCAACCUUGGGUGCACUUGCAAGCCCUCUACCUUCCCCAUAGCU*

*>hsa-mir-921*

*CGGGAAAUCAUCUAGCUCAUUUGCUUCUUUUACAGAUGGGGACGCUGAAGCCCAGAAAAGGGCAGUGCCACAACUAGUGAGGGACAGAACCAGGAUUCAGACUCAGGUCCAUGGGCCUGGAUCACUGGCUAAUUCUGUGGGUCAGGGCUGGAGGCACCCAGCACACCUAGUCUUCAAAGCAGCAGUCAGUGGUCACCUCC*

*>hsa-mir-1825*

*UGCUGAGGAGGUUGGAGAGAGAAAGGGUGAAAGCAGAGAGACCAGUGCAGGGCUGUUAACAGGGUUGCAGGCGAGAGACUGGGGUGCUGGGCUCCCCUAGACUAGGACUCCAGUGCCCUCCUCUCCCAAGAGACAAAGGCCAUUGCAUUGAAGGAGGUGGGAAAUGAUUAGAUUCUGAACAUAUGUAAUUAUUUUUCAGU*

*>hsa-mir-3912*

*AUUAUUUCUUACAGGAAAAUUAUAGAGACGAUUUCAGUGUCUAUCAAGAGAGGAAUGAACAGUUAAAUUAUAACAUGUCCAUAUUAUGGGUUAGUUGUGGACACAUACUAACGCAUAAUAUGGACAUGUUAUAAUUUAACUGUUCCUUUCUGAGAGCUCCUGUGUCACAUAAAGCUUUGAGUCACUUAAAUUUGUUAUGC*

*>hsa-mir-194-1*

*UACUUAGAGAUAUCGUACAGCUCCCAUGAUGAGCAAAAGGAAUCUCCUUAUAUGUUUAAUGGUGUUAUCAAGUGUAACAGCAACUCCAUGUGGACUGUGUACCAAUUUCCAGUGGAGAUGCUGUUACUUUUGAUGGUUACCAACUUGCUACAAUAUAAAGGUAUAAUAAAGAAAGACAGAAAAUUGUGGUCACUUGGACU*

*>hsa-mir-576*

*UGUAUUUAACUUUUGAGAAACUGCCAAACUGUUUUCCAAAGUAACUGCACCAUUUUACAAUCCAACGAGGAUUCUAAUUUCUCCACGUCUUUGGUAAUAAGGUUUGGCAAAGAUGUGGAAAAAUUGGAAUCCUCAUUCGAUUGGUUAUAACCAUCCUAGUGGAUGUGAAAUGUUAUCUCGUUGUUUUGAUUUUCAUUUUC*

*>hsa-mir-376b*

*GCUUCCAUUUUCCAUGAACUGUGUUCAGAUUUGUCCUUUCCAGAGCCCAGUCCUUCUUUGGUAUUUAAAACGUGGAUAUUCCUUCUAUGUUUACGUGAUUCCUGGUUAAUCAUAGAGGAAAAUCCAUGUUUUCAGUAUCAAAUGCUGCUUUGGAAAUGUUUCUGGAAGAGACCGUAGGGCCUGAGUAGGUGCAAAGAUGG*

*>hsa-mir-3613*

*CCCGGGUGGGGCCGCCCGGGAAGAAAGUCUCCCAGUUAAGCUGCCGUGCAAUUAAGAUGGUUGGGUUUGGAUUGUUGUACUUUUUUUUUUGUUCGUUGCAUUUUUAGGAACAAAAAAAAAAGCCCAACCCUUCACACCACUUCAUCCGCAUCUCAAGACCAGAAGCCGACCCCAGCGGCUUGGGGAAAUGGAGCCCCGCA*

*>hsa-mir-4272*

*CAGGAAUCCUCCUCCCAUAGAAAGGGCACCAUUAACCAACGGAGUUGUGUUAUCCAAAGGGCUGGAUCUUUUCUGCACAAAUUAAUCAGUUAAUGCAUAGAAAGCAUUCAACUAGUGAUUGUGUUAUAAGAGAUCUGCUCAAUAUUACUGUGUGUCUACCUUAUGGGGACACAUUGGUAAACAAAAACUGUCAUGGACCG*

*>hsa-mir-4304*

*UCCCUCUCAGCCUCAGAUCUAUACCUGGGUCCACUUGGCUCUCAGGGAACUGUCCGGUCUCCUAGCUUGGAGAGAAGUGGCCGGCAUGUCCAGGGCAUCCCCAUUGCUCUGUGACUGCUGCCAUCCUUCUCCUCACGCAGACACCCUGGUGGUUCCUUCCCCAGAACCCCCCACCUCUUGUUCAAGGGCCGGUGCUGCUG*

*>hsa-let-7f-1*

*UGUUGAAAUUUUCUUUCGAAAGAGAUUGUACUUUCCAUUCCAGAAGAAAACAUUGCUCUAUCAGAGUGAGGUAGUAGAUUGUAUAGUUGUGGGGUAGUGAUUUUACCCUGUUCAGGAGAUAACUAUACAAUCUAUUGCCUUCCCUGAGGAGUAGACUUGCUGCAUUAUUUUCUUUUUAUUUAGAUGAUAUUAAAACUCAG*

*>hsa-mir-3132*

*AUCUUCCUGCUGCCGAAUCCGCAUCAGUUGCACGCGUCGGCGCUGCCGGCUGAUGACCACUGUGGUGGGAUGGGUAGAGAAGGAGCUCAGAGGACGGUGCGCCUUGUUUCCCUUGAGCCCUCCCUCUCUCAUCCCACCUGUGGCUCUAGGCUUCCAGAUUCCUCUUGUCCAUAACCCCUUACUCCCACCCUGUCACUUCC*

*>hsa-mir-3120*

*AGAUGCUAUGGUGUGAGGGCUGCUUUCUUUCAAUGGCUGGUUGUCAUUCAGGCUGGGUUGUCAUGUGACUGCCUGUCUGUGCCUGCUGUACAGGUGAGCGGAUGUUCUGCACAGCAAGUGUAGACAGGCAGACACAUGACAACUCUGUCCAGCCAGGCCCUUGGUUCCUUCAGGUUUCCUUUGCUCAUAGACAGAUACAA*

*>hsa-mir-3648*

*GGGGGACCGGCCGCGACUGCGGCGGCGGUGGUGGGGGGAGCCGCGGGGAUCGCCGAGGGCCGGUCGGCCGCCCCGGGUGCCGCGCGGUGCCGCCGGCGGCGGUGAGGCCCCGCGCGUGUGUCCCGGCUGCGGUCGGCCGCGCUCGAGGGGUCCCCGUGGCGUCCCCUUCCCCGCCGGCCGCCUUUCUCGCGCCUUCCCCG*

*>hsa-mir-4265*

*CCUGGGAUCUGCACCCUAAAUUCACAUCCGAUUUCACGAUAGAGAAAAGUGCAGUGGGUUGGAGCUUCAGCCUACACCUGUAAAGAAUUGGUCAGCCUGGGGACUGGUGAUCUCUGCAGCUGUGGGCUCAGCUCUGGGCUGGGCCUGGUCACUGGGAAAGGCAGGCAUGUGCCUAGGUGCUGCAGAGGUCUCGAGCCCCU*

*>hsa-mir-592*

*CUCUUGCCCUUCCUGGGGCCAGGCUGUGGGGAGUGCUGAUGAAUUUAAUUUGAUGAUAUUAUGCCAUGACAUUGUGUCAAUAUGCGAUGAUGUGUUGUGAUGGCACAGCGUCAUCACGUGGUGACGCAACAUCAUGACGUAAGACGUCACAACGCCAGUUAAAGACGUGAUUCCAGAAGGGUUUUUAAACUGAGCAACAA*

*>hsa-mir-494*

*AAGAUUCGGCAGUUCUGUUUUGAUUUUUUUUGUUUGUUUUUUGAUCAGUGCUAAUCUUCGAUACUCGAAGGAGAGGUUGUCCGUGUUGUCUUCUCUUUAUUUAUGAUGAAACAUACACGGGAAACCUCUUUUUUAGUAUCAAAUCCCACCCUGGAGGCACUUCCUGUUCCUGAUGCAGCCUUCAGGGAGGGACGUUGCCG*

*>hsa-mir-1207*

*CCUGAGGAAUCAGAGAUAGGAGCCCUUUCUUUUUCCUGGCCGCCAGGCCUCCAUGUGCAGGGCUGGCAGGGAGGCUGGGAGGGGCUGGCUGGGUCUGGUAGUGGGCAUCAGCUGGCCCUCAUUUCUUAAGACAGCACUUCUGUUAGCUGACCUACACAGAAGCAGGUCAUUUCGAGAUGUUUGCGUCUUGGAGCCACUGA*

*>hsa-mir-1182*

*CAGGAGCAGAACUCCCUGCACGACAGGAGGGACCGAGGCCCUCCUCGGGACUUGUCACUGCCUGUCUCCUCCCUCUCCAGCAGCGACUGGAUUCUGGAGUCCAUCUAGAGGGUCUUGGGAGGGAUGUGACUGUUGGGAAGCCCUUCCUACUGGACACGCUGUCAUCAUUUGCUGCUUCUCUUGCAAGAAAGCACCUCCGU*

*>hsa-mir-1827*

*AAGUCAAGUGGGCCUGCAACAGAGGAAAUGACAGGAGUCAGACCCCAUGGGACCCUGAAGGCCAUGUUCAGCAGCACAGCCUUCAGCCUAAAGCAAUGAGAAGCCUCUGAAAGGCUGAGGCAGUAGAUUGAAUUGAUUCAGGUCCCUUAGAAUUGGGUCCCUGCAACAGGUGGACCCAGGACCGGCAGAGAAUGCAGAUA*

*>hsa-mir-320d-2*

*UUUAAGCAAGUGGGAACUUUUAGCCUUUCUAAAGUUGUAAUUCUACUUCUGAAAUGUCUUCAAAUGUAUCAAUAAGCCUUCUCUUCCCAGUUCUUCUUGGAGUCAGGAAAAGCUGGGUUGAGAGGAGCAGAAAAGAAAAAAGAAAAAAAAAUCGUUAUUUUUAAAUUCAAGAAAAAAAGCUGGAUUUCCCACUAUUAUAU*

*>hsa-mir-135a-1*

*CCCUCAGGGAGGAGGGGAGGGUUGGGGUGGAAGAAGUGCCUGCAAGAGCAGCCCCAGGCCUCGCUGUUCUCUAUGGCUUUUUAUUCCUAUGUGAUUCUACUGCUCACUCAUAUAGGGAUUGGAGCCGUGGCGCACGGCGGGGACAGCCAGCGGAGGGUUCUGACACUGAGCAAGGGGGCUCAAAAGGAGGCAGGACAGUG*

*>hsa-mir-548p*

*GAAAUUGUCACAUUUGAUUUUUGGAAGAAGAAUAACAUUUUAAGAGAAAAAACUAUCAUUAUUAGGUUGGUAUAAAAUUAAUUGCAGUUUUUGUCAUUACUUUCAAUAGCAAAAACUGCAGUUACUUUUGCACCAAUGUAAUACUUCUAAACUUUUAGACUUCUUUAUUUAAAUAUAAACACAAACCAAUAUUCCUUGUU*

*>hsa-mir-3154*

*GGUAGGAGCGGCCUCCUCCCCCCACCCCUCCCUUCCUGCCGGCCGGCCCCCUCCUCAGGCCCCUCCUUCUCAGCCCCAGCUCCCGCUCACCCCUGCCACGUCAAAGGAGGCAGAAGGGGAGUUGGGAGCAGAGAGGGGACCACGGGCUGGCUGGUCUGGGGCUAGGGGGACACAGGCUGGGGUUGGGGGGUUCUCGGAUC*

*>hsa-mir-1248*

*GUUUUAUAUUAAUGUUGAGAGAGAGGUAACUGUCUGAUUGUUAGACAUUAUUUUACCUUCUUGUAUAAGCACUGUGCUAAAAUUGCAGACACUAGGACCAUGUCUUGGUUUUUGCAAUAAUGCUAGCAGAGUACACACAAGAAGAAAAGUAACAGCACUAGAUUGUAAAGACUGGGGUGGACCUCUUUCUUAAUGUCCAA*

*>hsa-mir-3124*

*CGACGCCGAAAGUGAGCUGGUAAACUUCCGGCAGUGGCUCCGGGGGCAACUCGAGUCCGGACGCUGGCGGGCUUCGCGGGCGAAGGCAAAGUCGAUUUCCAAAAGUGACUUUCCUCACUCCCGUGAAGUCGGCGGAACCCUCCACUAACGGCGUCUGAGCGGACGGCUCCGAUGUGUAAAUCCCACUCCUCGAGGCCUCU*

*>hsa-mir-3175*

*GUAACAGCAGGGUCAACGGCGGCGCCUUCGGCAGCCUCCGCCCCGUGACGUCAGACGGCUCCCCUGGGGGGCGGGGAGAGAACGCAGUGACGUCUGGCCGCGUGCGCAUGUCGGGCGCUUUCUCCUCCCCCUACCCAGGGAGCCGCACGCCGAGGGGAAAAGGAAGGGAACUCUAGUUGGGACUUUCCGGUGGGCGGCUU*

*>hsa-mir-3187*

*CGCGAGGCGUCCAGCGGCACGUGGAUGGUCAUGUGGCGGCGCGCGGGGUCGUCCAGCGAGGGCGCGCUGGCCCUGGGCAGCGUGUGGCUGAAGGUCACCAUGUUCUCCUUGGCCAUGGGGCUGCGCGGGGCCAGCAGGUCCACGUCCACGCUGGCGCGCUUCAGGCUGCCCAGCGAGGUCUUCUCGCGGGCCACGGGCCG*

*>hsa-mir-568*

*AAGUUAAGCAUGUGCUAGUGUCUGAACCCAGUUCAGUUUAUCUCCAGUUGAAACGAUAUACACUAUAUUAUGUAUAAAUGUAUACACACUUCCUAUAUGUAUCCACAUAUAUAUAGUGUAUAUAUUAUACAUGUAUAGGUGUGUAUAUGUGCAUAUAUACACACAUGCACAUAACAAAAUCAGAUGCUCAUUACAAAUCC*

*>hsa-mir-548y*

*AAACAACUGUUUGUUGUUUAAGCCACCAGCCUAUAGUAGUUUGUGACAGCAGCCUAAACUAUUAGGUUGGUGCAAAAGUAAUCACUGUUUUUGCCAUUACUCUCAGUGGCAAAAACCGUGAUUACUUUUGCACCAACCUAGUAACACCUUCACUGUGGGGGUGCAGACAUGGCAGAUGUGGAGCAUCCAAGCUGGACUCU*

*>hsa-mir-101-2*

*UGGCCCAUCUGAGGUUGGUGAGCUGGGUGGCAGGGAAUAUUCAGGUAGAUAUGAGACUGAACUGUCCUUUUUCGGUUAUCAUGGUACCGAUGCUGUAUAUCUGAAAGGUACAGUACUGUGAUAACUGAAGAAUGGUGGUGCCAUCACAUUGAGAAAGGGUUGAGGACUGCGGUGGGGCUAUGCACCCUUGGGCCAUGGCG*

*>hsa-mir-637*

*GCUGGGAGGCAGACGCUGUUGGGGCCUCUCACUGCCGGGCUGUUGACCUUGAUCCUUUGGCUAAGGUGUUGGCUCGGGCUCCCCACUGCAGUUACCCUCCCCUCGGCGUUACUGAGCACUGGGGGCUUUCGGGCUCUGCGUCUGCACAGAUACUUCGAGUCUCCAAGGAGACUUCUGCCUGUUCAUUUCUGCUGCUGUCA*

*>hsa-mir-3131*

*UCUGCAGGGUGGGGGUAGGUUGAGACUUGGGCUGCCCACACUUGUUGGCGCCUCGAAUCUCAGAGUCUCAGAGUCGAGGACUGGUGGAAGGGCCUUUCCCCUCAGACCAAGGCCCUGGCCCCAGCUUCUUCUCAGAGUCAGCUGCACAGUCUCUUGGGCUCUCAGGACCCAGGCACACCAGGAGGCAGCAACCAAGGAUG*

*>hsa-mir-1249*

*AGGAGGCUGACGGGGCUCCCGAAGUCUCAGUCCCAGCUCAUCCUGCUCCUCAGAUUAUUGUUGGCAUGGGGAGGAGGGAGGAGAUGGGCCAAGUUCCCUCUGGCUGGAACGCCCUUCCCCCCCUUCUUCACCUGGCGAACUCCUACUCGUCCUUCAAGACCCAGCUCCAGCGGCAUCUCCUCCAGGAAGCCUUCCCUGAU*

*>hsa-mir-1290*

*AGUUGAGCAUCCCUACUCCCAAGUUCCAAAGUCCAAAAUGCUCCAAUGAGCAUUUCCUUUGAGCGUCACGUUGACACUCAAAAAGUUUCAGAUUUUGGAACAUUUCGGAUUUUGGAUUUUUGGAUCAGGGAUGCUCAACUUGUCAUGCGUUCCUCAUACGCUUAUGUGGUGGAUUCAGUGAGAUGAUCCUGUCUCACUUA*

*>hsa-mir-3122*

*CAGAAUGAAGGUUAUCCAACUCAAAAUGAAGGAGUAUAACUUUGGGUUUUGUAAAAGUUAGUGACCAGCUCUGUUGGGACAAGAGGACGGUCUUCUUUUGGAAGGAAGACCAUCAUCUUGUCCGAAGAGAGCUGGUAGCAUGUUUCAGAAAUCUUCAGGUUUCACAGUUAAUUUAGUGUUCCUAGAAGUAGCUGACUAGG*

*>hsa-mir-483*

*UUGGCACCCCAAGGUGGAGCCCCCAGCGACCUUCCCCUUCCAGCUGAGCAUUGCUGUGGGGGAGAGGGGGAAGACGGGAGGAAAGAAGGGAGUGGUUCCAUCACGCCUCCUCACUCCUCUCCUCCCGUCUUCUCCUCUCCUGCCCUUGUCUCCCUGUCUCAGCAGCUCCAGGGGUGGUGUGGGCCCCUCCAGCCUCCUAG*

*>hsa-mir-4253*

*GACUGGCUGUAACCGGACGGCACCCAAUCCUCACUCUGAGCUUUUCUGCAGGUAGACCUGGGACUCGGCCAGCCAUCGCCCUUGAGGGGCCCUAGGACUUACUUGUGCAGGGCAUGUCCAGGGGGUCCAGGUCUGCUCUGUAGUAGCCAUUGCGGCAGACACAGUUGGUGGCCCCUUCAGAAGUGGUCCGGCUGUUGAUG*

*>hsa-mir-609*

*UGUGGUUGGUGUGGUGGCUCCUCGGGGUCAGCGUCCAGUCUCCCGCUGUCAUGCUGCUCGGCUGUUCCUAGGGUGUUUCUCUCAUCUCUGGUCUAUAAUGGGUUAAAUAGUAGAGAUGAGGGCAACACCCUAGGAACAGCAGAGGAACCCUAAACCACCGGUGUAUUUUGGGAGCCAAUGGGGAGGGAAAAGGAUGAGGG*

*>hsa-mir-4271*

*AUCAUAUCAAGCAGAUCCUUUGUGACCUCUUGGCUAGAAUUGUUGAGUGGCAUGUUUGAGUCAGCCUUCAAAUCUCUCUCCAUAUCUUUCCUGCAGCCCCCAGGUGGGGGGGAAGAAAAGGUGGGGAAUUAGAUUCUCUUUCAAGAAAAUAUUGAAGGGAAACUCAGUUUUAGAUAAUCUCAAAGACAAAAACUUACUGG*

*>hsa-mir-3147*

*UGGCUACUGAUUUCGGACACCUCCUUGCCAUGGGAUGUGAGUCCGGAGAGGGACAGGAGGGGCAGGUCCGGGUUGGGCAGUGAGGAGGGUGUGACGCCGCGAAGUGCACCUCGCCCUUGUCCAACUCGGACGGGUGUGGUCCUCACCUCACUUCCCGCUGCUAGGCUGGGUUCCCUCUCCCACCUGCACCCGGGGUCUUU*

*>hsa-mir-1302-7*

*AAAAGAUAUUGAGCACCAGCUAAACGUAGGUACAUUUCAAUUCAUCCAAACUUGAAUUCCAUAUAAACAACAUGUUUUUAGGACAUGUAUGUCUGGUGCAAUAAUUGGGACAUACUUAUGCUAAAAAAAUUAGUGUUCACUAUUUAGCUGAGAUUAAAAUUUAGCUGGGCCUCCUGUUUUCAUGUGGAAACCCUACCCCG*

*>hsa-mir-556*

*UGGACUACAGCAGUUGUUCCAUGCACAGGGUUCCAAGGUCCCUAGGAGUUCACAAAGAUAGUAAUAAGAAAGAUGAGCUCAUUGUAAUAUGAGCUUCAUUUAUACAUUUCAUAUUACCAUUAGCUCAUCUUUUUUAUUACUACCUUCAACAAGGCCUUCUAGACUUAUCAAAACACUGGAUAUCAGUUAGGUAAUGUAAU*

*>hsa-mir-3659*

*UGGUAUGCAUAUGUGUCCUUGUGUGUGGAUGCACACGUGCUGCUUGUGUCUCUACAAGCAGAUACAAGGAUGCCCUUGUACACAACACACGUGCUGCUUGUAUAGACAUGAGUGUUGUCUACGAGGGCAUCCUUGUGUCUGUGUGUGUGCCUGUGUUGGUCUGGGCAUGAGUGCAUAUGAGUCUAUGGUUUUAUAUGUGU*

*>hsa-mir-1275*

*CCAAGUUUUUACCCAGGACCCACAAGUCUUUGGUCUUCACCAAGAUCACCAUCUAAGGACUCCUCUGUGAGAAAGGGUGUGGGGGAGAGGCUGUCUUGUGUCUGUAAGUAUGCCAAACUUAUUUUCCCCAAGGCAGAGGGAACUCAGCUUCUGUGAGUAGCCCCCAUGCGGUGGCUGCGGAGUUGCUGACCCUGGUGUGA*

*>hsa-mir-4289*

*CAUGAGUGACAGCAGCUAGCAUGGUAUCUCUGCCCUAGAUGCACUGAUGGCAGCAGAGAGCUCUGUCCCUUGGGAGGGCAUUGUGCAGGGCUAUCAGGCAGUUUCCUGGGCCCUGUCUGCAGAGCCUAAACAGAUCACAUCUGGCCAGUCUCAACAGGGCCUCCUUACCCUAUAAACCAUGCAUGUAUUUAUGUAACACU*

*>hsa-mir-218-1*

*CUCCAUUUUCCUCUUGGUCUUACCUUUGGCCUAGUGGUUGGUGUAGUGAUAAUGUAGCGAGAUUUUCUGUUGUGCUUGAUCUAACCAUGUGGUUGCGAGGUAUGAGUAAAACAUGGUUCCGUCAAGCACCAUGGAACGUCACGCAGCUUUCUACAGCAUGACAAGCUGCUGAGGCUUAAAUCAGGAUUUUCCUGUCUCUU*

*>hsa-mir-200b*

*ACUGAGCUUCCCAGCGAGUCCCAUGCAACCCUCAGCCGGGCGGCCCCCGGACCCAGCUCGGGCAGCCGUGGCCAUCUUACUGGGCAGCAUUGGAUGGAGUCAGGUCUCUAAUACUGCCUGGUAAUGAUGACGGCGGAGCCCUGCACGCAGCGACCGGCCGACCCCGUCCCGGCCCCCAGGGCCUCCCGCCGAGCCCCACA*

*>hsa-mir-3919*

*GUAUGGCAUAAUGAGAAAUAAUAGUCUUAUUUUAUUCCAAGUAGUUAACCCUUUACCUGAGCACCAUUUACUGAGUCCUUUGUUCUCUACUAGUUUGUAGUAGUUCGUAGCAGAGAACAAAGGACUCAGUAAAUGGUGCUCAGGAAUCUUUACCAUAUGUUAAAAAUAAAUCCAUUAGAAUCUGUUUCUGAACUACCUUC*

*>hsa-mir-3119-1*

*AUAGCUGUAUGGUGAGUUUUAUUCUGGAAGUUCCAAGUGCUUUUUAGGAAAUGUUGUUAUUAACUCUGGCUUUUAACUUUGAUGGCAAAGGGGUAGCUAAACAAUCUAUGUCUUUGCCAUCAAAGUUAAAAGCCAUAGUUAAUUUCCUCCUCAGAGACUGAUUCUUCUGAAAAGGGAUCAACGCAAGAGAGGAUACCUCC*

*>hsa-mir-3183*

*GUGUCCCCAACCCAGCUGCUGAGCUGGAAGCCAGGGGUAGGUCCUGGCAGCCCCUCCCUCUCUGCCCUGCCUCUCUCGGAGUCGCUCGGAGCAGUCACGUUGACGGAAUCCUCCGGCGCCUCCUCGAGGGAGGAGAGGCAGGGUCUUGGCAACUCCCGUCUCCCCCGUUGCCAGCUUGCCAGCCCCGCCGUGCCUCUCAC*

*>hsa-mir-552*

*UGUUCACAGCAGCUUUAUUUGUAAUAGUCGAAUAUACUACAGUUUGUUUAACCAUUCAAAUAUACCACAGUUUGUUUAACCUUUUGCCUGUUGGUUGAAGAUGCCUUUCAACAGGUGACUGGUUAGACAAACUGUGGUAUAUACACAGGAUGGAAUACUACUCAGCAAUAAACAAGCAACAAGCUAUUGAUUACAAACAA*

*>hsa-mir-548b*

*CAUUGUGCUUGGUACUAUUAAUUAUUUAAUACUAUUUAUUAUUCCAUUUCAGACUAUAUAUUUAGGUUGGCGCAAAAGUAAUUGUGGUUUUGGCCUUUAUUUUCAAUGGCAAGAACCUCAGUUGCUUUUGUGCCAACCUAAUACUUCACAUAUAUUUUCCAACAGACUUGAGAAAAUGUAACUGGAAUAUAUUAAUCAUA*

*>hsa-mir-641*

*AGGUCACUGAGCCCCUAGUGCCAUGCAUUCCUUGCUCACCAGGUUCCAGGCUGGGUGAAAGGAAGGAAAGACAUAGGAUAGAGUCACCUCUGUCCUCUGUCCUCUACCUAUAGAGGUGACUGUCCUAUGUCUUUCCUUCCUCUUACCCCUGAGCCUCAGGCUGCCCUGAGCAUCUCUGCCUACAGCCAGUCCAGCAUUUC*

*>hsa-mir-486*

*ACCAUCUCCAGCAGGUGUGUGUGGUGCUGGGGGCUUCAGCGGCCGGCUCUGAUCUCCAUCCUCCCUGGGGCAUCCUGUACUGAGCUGCCCCGAGGCCCUUCAUGCUGCCCAGCUCGGGGCAGCUCAGUACAGGAUACUCGGGGUGGGAGUCAGCAGGAGGUGAGGGGGCAUGGUGGCCCCAGUGCAGCACUAACCAGGCC*

*>hsa-mir-2355*

*UGUGAAUUAUGCAUUGGCCCUUUCAGCUACAUUCACGUGCAUAGAAAAUAACAUUGUCAGACGUGUCAUCCCCAGAUACAAUGGACAAUAUGCUAUUAUAAUCGUAUGGCAUUGUCCUUGCUGUUUGGAGAUAAUACUGCUGACUUUAUUCCUCUCAUACAUGUGAAUGUGGCUGUAGGGAGCACGUGACAUCUGUGGCU*

*>hsa-mir-3934*

*CAACCCACAGACCCAGAUGCUUCAUCGCAAAAUUUGGCCUGCUCCACACAGCCCUUCCUGUCCCCAGUUUUCAGGUGUGGAAACUGAGGCAGGAGGCAGUGAAGUAACUUGCUCAGGUUGCACAGCUGGGAAGUGGAGCAGGGAUUUGAAUCCUGGUGUGUGGACUCAGCACAUGCUGUGGAGUUGAGCAGGCACUGGGA*

*>hsa-mir-544b*

*UUGUACAAAUUAGAGGUUUCUAAUACAGUGCUUCUCAACUUUGAGUAUAUAUGAAUUACCUGGGAAUUUUGUUAAAAUGCAGAAUCCAUUUCUGUAGCUCUGAGACUAGACCUGAGGUUGUGCAUUUCUAACAAAGUGCCAGCUGAAACUGCAGACCACACUGAGUAGAGGGCCGUAAAUAUGCUUUCCAAUACAGUUUG*

*>hsa-mir-3650*

*UAAAAAACACGUUGGCCUUGGUGCUGGUGCAUCACAUAAAUCACAGAUGAAACAAACAAGCCUGUUUCCUACUCAAGGUGUGUCUGUAGAGUCCUGACUGCGUGCCAGGGGCUCUGUCUGGCACAUUUCUGAACUUUGCAAAAUUAAUUGUGCCGAAGAGAACACCAUCAAACGCGUCCGGCAUACAGCGUAAACAUCUU*

*>hsa-mir-3189*

*CCCCCAAGCAGCCCCCAUAUCUAAUCAGGGAUUCCUCAUCUUGAAAAGCCCAGACCUACCUUUGAGCCUCAGUUGCCCCAUCUGUGCCCUGGGUAGGAAUAUCCUGGAUCCCCUUGGGUCUGAUGGGGUAGCCGAUGCCUGAUUUGCACCCACAACGUGGGAGGUUAUAACCUGUCCCCAAGUUGCAAAUGGGGAAACUG*

*>hsa-mir-4290*

*AGGGAGGGAUUUGAGCAGAGAGGAACACAGCCCUUGGCCCCAUGGUGGGCUGCCACCAAGAAGGUGAAGGGAGGGUCAGUCCCAAUCUGAAUCCCACCAAAAUAGGUGGUAGAGGGUUGCCCUCCUUUCUUCCCUCACCUCUGACCCCGCUCUUCGAAGCCCUGGUCCUGUCAAGGCACCUAUCCCAUGGCCCAUGUGGC*

*>hsa-mir-320a*

*GGCGCGGGGCGGAAGUGACGUUAGGGGGGCGGGACUGGGCCACAGUAUUUAUCAGGCGGCGCUUCGCUCCCCUCCGCCUUCUCUUCCCGGUUCUUCCCGGAGUCGGGAAAAGCUGGGUUGAGAGGGCGAAAAAGGAUGAGGUGACUGGUCUGGGCUACGCUAUGCUGCGGCGCUCGGGGGUCUUGGCCUCCGGGCGGUGG*

*>hsa-mir-676*

*CAUGAAAAUGACCCUUUGCUCCUUCCAAGUUCAACACGAGACUUCAUAUCAGAUUCUCACCUGAACGCAUGACUCUUCAACCUCAGGACUUGCAGAAUUAAUGGAAUGCUGUCCUAAGGUUGUUGAGUUGUGCAUUUCUGGGCAUUUCAUCUCUAUGGGGAAGUACCCAUUCCCUCACCCCAAAGACUGAAGUAGAGAGA*

*>hsa-mir-638*

*CGGCCAGAGCGCCGGCAAGAGCUCGGUGCUGGAGAACUUCGUGGGCCGGUGAGCGGGCGCGGCAGGGAUCGCGGGCGGGUGGCGGCCUAGGGCGCGGAGGGCGGACCGGGAAUGGCGCGCCGUGCGCCGCCGGCGUAACUGCGGCGCUUGCGUGCCCGCGGCGGGGAACCGGACGGGGUCGGGGAGGCGGGCCCUGUGGG*

*>hsa-mir-4267*

*CCCAGAGUAGGCCCAGGGGUGGUAUCUCCGAAGGAGCCGAGGAUUCUCCGUGAUGACUUGCAAACUCAGCAGGCUCCAGCUCGGUGGCACUGGGGGAAGGCUCCAGACCCCAGCCUCUGUCAUCCCUGCAUGGAGCCCACAUCUCCAAGGAGGCUGGGAGAUCCUGUUUGGGGGUCAAUUUAAAUGUGAAGGAGCAGAAU*

*>hsa-mir-367*

*UGUGGUGGUUCCUACCUAAUCAGCAAUUGCGUUAACGCCCACACUGUGUGCAGUUCUUGGCUACAGGCCAUUACUGUUGCUAAUAUGCAACUCUGUUGAAUAUAAAUUGGAAUUGCACUUUAGCAAUGGUGAUGGAUUGUUAAGCCAAUGACAGAAUUUAAACCACAGACUUACUUUGAUAGCACUCUUAAUGGUAUAAC*

*>hsa-mir-152*

*AUAGCGCAGGUCCAGCCCGGCCAGGGAUCAGCUGGAAGAAGGAGGCUCGGCCCGCUGUCCCCCCCGGCCCAGGUUCUGUGAUACACUCCGACUCGGGCUCUGGAGCAGUCAGUGCAUGACAGAACUUGGGCCCGGAAGGACCUUCUGCACCCAACGGGCACAGCGCCCACUCGGGGCCUGCAGUGGAACAUCUGCCUGGG*

*>hsa-mir-1910*

*UCAGACUCGGGAAAUGACCUGCUUGAGGUGGCACAGCUCCUCUAGACUCCCAAUUCUUUCUUGUCCCUUCAGCCAGUCCUGUGCCUGCCGCCUUUGUGCUGUCCUUGGAGGGAGGCAGAAGCAGGAUGACAAUGAGGGCAAGUUCAGGGACACUGUGGGAGAUGAGAAAACUUGCUGUGUGAAAACUCUCCACGUGGGAG*

*>hsa-mir-3199-2*

*AUACAUCUGUGGAGUUAGGGCUGCCUCCAAAAAAAUAACGGAAAUGGACUGACUCAGUGACUCCCAGGGACUGCCUUAGGAGAAAGUUUCUGGAAUGUCAGAACUUCCAGAAACUUUCUCCUAAGGCAGUCCCUGGAGUCACCGAGUUUCUCUUAUGUGUGACUAGUGCUAGUUAUUGUGAGGGCAGAGAUUUAAGGAAA*

*>hsa-mir-3136*

*UUUGAGUGUUGCCCUUUGUCUAGCUCAUAACUAUAUUACUGAUAUUACAAGUAAAAAUUAAUAUGAAACUGACUGAAUAGGUAGGGUCAUUUUUCUGUGACUGCACAUGGCCCAACCUAUUCAGUUAGUUCCAUAUUAGUUUAUUUCAUAUUGAUAAGUAAAGCGUGUUAUUAUGAGAUGAAUCAUAGGUAUCUUGGACU*

*>hsa-mir-3918*

*CCAGAGGCUUAGGUAACAUAAUUGUGUUUAACGUAAAUAUACACAGAUACCAAUAGGCGGUUAAGCCAUGGGACAGGGCCGCAGAUGGAGACUGCUCAAGGUCAAAGGGGUCUCCAGCUGGGACCCUGCACCUGGUUCGUAGCCCCUCUGCAGACGCACAGUGCCUCACGCCUGCUGCAACCUGGAACCUUGAGGCCUUC*

*>hsa-mir-571*

*CUGGACUGUAGCUGAGAUUGAAUUUGAGAGAGGUUACAGAACUGCUUCAGAAUCCUCAGUAAGACCAAGCUCAGUGUGCCAUUUCCUUGUCUGUAGCCAUGUCUAUGGGCUCUUGAGUUGGCCAUCUGAGUGAGGGCCUGCUUAUUCUAAAUAACCCUCGAUCUCUGGCUCCACUGAGGUUUCACAACCCUAACCAUAGG*

*>hsa-mir-3622b*

*UAGCAUCAGUCAUCAAGCAGCUCACGAUCUGCAAACAAUGGUAGACAUACGAGUGAUAUAAUAGAGGGUGCACAGGCAUGGGAGGUCAGGUGAGCUCAGCUCCCUGCCUCACCUGAGCUCCCGUGCCUGUGCACCCUCUAUUGGCUAUGAUCACACCACUGCACUCCAGCCUGGGUGACAAAACAAGACCUUGCCACUAA*

*>hsa-mir-541*

*CUGGCCUCUUGCUCCACUUGUAGCAAGGCGCAGAGGGCUCUCUGAGCUCAUGCCAACGUCAGGGAAAGGAUUCUGCUGUCGGUCCCACUCCAAAGUUCACAGAAUGGGUGGUGGGCACAGAAUCUGGACUCUGCUUGUGGCAUCCAUCGUCUGGCCCGAUGCUUGCUGGCUGCCCGCUCCAGGAGGCACCUUCUGGGUGU*

*>hsa-mir-3184*

*CUGUCUCUCUUCACACUGCCCCUGAGAGCAUCCAAGCGGGGAGAAACUCAAGCGCGGGUUAGGAAGCAAGACUGAGGGGCCUCAGACCGAGCUUUUGGAAAAUAGAAAAGUCUCGCUCUCUGCCCCUCAGCCUAACUUCCUUUAUUUCCUCACAAGUUUCUCCCUCAAACUUCGGGCUUCCAUCCGGGAAAAUGUACGGG*

*>hsa-mir-384*

*AUGCCAGUGUAUGAUCAAGUUCCUUGCCUUUUAACUAGUAUUUAUGUUAUAGGCAGAAUGUUAAAUCAGGAAUUUUAAACAAUUCCUAGACAAUAUGUAUAAUGUUCAUAAGUCAUUCCUAGAAAUUGUUCAUAAUGCCUGUAACAUAUAUGCAAAUAACAAGAUGCCUGAAAUACAUAGGAAACCUUCAAGAUGCUAAC*

*>hsa-mir-3176*

*ACUCUCGGCCCCUGCUUUUUGAGCACCAGGCCGAGUUUCAGACCUCGGGAUGGCCUCUCCAGUCUGCAGCUCCCGGCAGCCUCGGGCCACACUCCCGGGAUCCCCAGGGACUGGCCUGGGACUACCGGGGGUGGCGGCCGUGGCUCUGGCUAUGGGGAGGGAGGCAGAGCCGCGGGGCAGGCGUGGGGUCUGCUGUGCCG*

*>hsa-mir-3913-2*

*UUUAGAGAACAAAUUUUUAGACAUCCGUCAACUUAGAGAAAGUAGAAAACUGUCUAUAAUAAACUGAAAUAUUUGGGACUGAUCUUGAUGUCUGCCAAGGUUUUGGCAGACAUCAAGAUCAGUCCCAAAUAUUUCAGUUUAUUAUAAACAAAGACUAAUAAGUCGUCUCUACUAUUGGAGGAAAAAAAAAAAAAAGACCG*

*>hsa-mir-4318*

*UACCUUCUUACCUACCUGUACCAUCUGUUAUUACCUCUUAAUUCCUUCAUGAGUUUGUAGGAGCUUCUUAAUUAUGUCAUAAACCCACUGUGGACAAGGGCCUUGUCUUAGACAGUCACUGUGGGUACAUGCUAGGUGCUCAAAAACUUGUUUCAAUGAUUGAUUUCUAUAUUCAUGAGGAUCAAAAUAAUCCCAAAGGA*

*>hsa-mir-3148*

*UGCUUCAGCAACAUCCUCUCCCAUGCACCUUAAACGUCUCUUUCUUUCUCAGCUCCUUUCUGGAGUUAAGAUGGAAAAAACUGGUGUGUGCUUAUUGAUGUAGCCAACAAGCAUACAUCAGUUUUUUCCAACUUAACUCCAGUAUUUUCCCCAAUUCAUCCUGAAAUUGCUGCCCUAUCCAUUCUCCCUCCUACACAGCC*

*>hsa-mir-1307*

*ACUCCCUGACAGAUAUCUCCCUCUUCCAUUUCAUCAAGACCCAGCUGAGUCACUGUCACUGCCUACCAAUCUCGACCGGACCUCGACCGGCUCGUCUGUGUUGCCAAUCGACUCGGCGUGGCGUCGGUCGUGGUAGAUAGGCGGUCAUGCAUACGAAUUUUCAGCUCUUGUUCUGGUGACCUUUUGAAUACGUCUUGUCU*

*>hsa-mir-1296*

*UUAUCGGCACCAGGGAAAGAAAGUGUUGGCAUUCAUAAUUGAAAUCAACGAAUCACCUACCUAACUGGGUUAGGGCCCUGGCUCCAUCUCCUUUAGGAAAACCUUCUGUGGGGAGUGGGGCUUCGACCCUAACCCAGGUGGGCUGUAACACUGCUGUGUUUUCUAAGGGGCAGAGUUUUCUACUACUUUUCCGCUGGCCC*

*>hsa-mir-630*

*AAGGCUCACUGAGGUUAAAUAACUCCCUCAAUUUUUCAUUUCCUUCUACCUGAAACUUAACAUCAUGCUACCUCUUUGUAUCAUAUUUUGUUAUUCUGGUCACAGAAUGACCUAGUAUUCUGUACCAGGGAAGGUAGUUCUUAACUAUAUUGUACUUUUAAACCACAUUAAAGACUAUUAUAAAUGCCUAUCACUGCUAC*

*>hsa-mir-4274*

*CCAGGGCCACCCCUGCAGAGCCCAGUGGCCCUGAGCAUCCAAUCUGGUAGGCGAGGGGCAUUUAGGGUAACUGAGCUGCUGCCGGGGCCUGGCGCUCCUCUACCUUGUCAGGUGACCCAGCAGUCCCUCCCCCUGCAUGGUGCCCAGUGCUAUUUGAGGGUCUGAGCCAGCCAAUGGGUUGAGAGUACCCCCGCUCCCCA*

*>hsa-mir-3158-1*

*CCCUGUGUGCUUGUGUAUGAGCGGGGUGUAGGGUGGGGUUUGAUCUGGCGUUAGGAUCAUUAUUCAGGCCGGUCCUGCAGAGAGGAAGCCCUUCUGCUUACAGGUAUUGGAAGGGCUUCCUCUCUGCAGGACCGGCCUGAAUAAUGUAAUCAGCUCCAUCGGGGAAGAGAAACUUGAAUGAGUAUCUAGUAUAGUACCUG*

*>hsa-mir-1273c*

*UUGAAGAUCAGUUGAGCCCAGGAGGUCAAGGCUUCAGUGAGCUAUGUUCACGCCUCUGCACUGCAGCCUGGGCGACAAAACGAGACCCUGUCUUUUUUUUUUUCUGAGACAGAGUCUCGUUCUGUUGCCCAAGCUGGAGUGCAUGAUCUCACCUCUCUGCAACCUCCGCCUCAGUACCAAGUAGCUGGGACCACAGGUGC*

*>hsa-mir-421*

*AUUCCUUAAGGGCAAGGACAGUGCCUUACUCAUCUUUGUAUUCACAGUGCCUAAUCCGGUGCACAUUGUAGGCCUCAUUAAAUGUUUGUUGAAUGAAAAAAUGAAUCAUCAACAGACAUUAAUUGGGCGCCUGCUCUGUGAUCUCCAUGGGCUCAGCUUGUCCCCGCCAGUUGCCAACAACGUCCAAGCUCUCUUCAGAA*

*>hsa-mir-2861*

*GGGGCCGCCGCUAGUAGUGGCGGCGGCGGAGGCGGGGGCAGCGGCGGCGGCGGCGGAGGCGCCUCUGCAGCUCCGGCUCCCCCUGGCCUCUCGGGAACUACAAGUCCCAGGGGGCCUGGCGGUGGGCGGCGGGCGGAAGAGGCGGGGUCGGCGCCGCGAGGCCGGAAGUGGCCGUGGAGGCGGAAGUGGCGCGGCCGCGG*

*>hsa-mir-4260*

*ACCGGCAGCCCCAAGCUUGUGGCCCUGAGGCUGGAGAUGUCUUCGUUGCCUGACCUGACACCCACCUUCAACAAGGUGACUUGGGGCAUGGAGUCCCACUUCCUGGAGCCCACACCCCAGCUUGUCACACACCAACUGCUGCCACCUGCUUCCUAUUAGGGCAGAUCUGCCCCUGUGAGAACCCCACUCCCCCAGCCAUC*

*>hsa-mir-3666*

*GCCUAUUUUUUUUUUUUCCUUCUGCUGUGUCUGACUGAUGGAAAAGUAAGGUCCGUCAGUUGUAAUGAGACCCAGUGCAAGUGUAGAUGCCGACUCCGUGGCAGAGUUCAGCGUUUCACACUGCCUGGUCUCUGUCACUCUAUUGAAUUAGAUUGAUGAUGGCAGAUUGCAGGGGGCACUAACUGGACCUCAGUGGGAAU*

*>hsa-mir-4311*

*CCAAUGGCAGACAGUAAGUGGCUAGACACCAAAGUGAGUGGUUUAACAAUUCAGAGAGGGGAAAGAGAGCUGAGUGUGACCUGGAGCAGCUCAGGAGGGCUUCCUGGGUGAGGUGGCAGGUUACAGGUUCGAUCUUUGGCCCUCAGAUUCAGCACCUGUGGUGGUGGUGGCUGCAGAAAGUGAGGCACUUCGGACUGGCU*

*>hsa-mir-3941*

*AAAAUGAUGGUGAGGGCAGGGGUUUCUUACUGUACAAUGUCUGCAGGUAGAGUCAGAAUUCUCAUCAGGCUGUGAUGCUCAGUUGUGUGUAGAUUGAAAGCCCUAAUUUUACACACAACUGAGGAUCAUAGCCUGAUGGUUCCUUUUUGUUUUACUCUUAAGUCCAAAAUACCAGUCAGAGAUAUUUAAGUGCUUCUGCA*

*>hsa-mir-890*

*AUGAAAGCUGCAGUGCACGAUGGUGGAUGAUGGGGCAGCACCUCUACAAUCAGACAUGCUGUGGGAAGUGCCCUACUUGGAAAGGCAUCAGUUGCUUAGAUUACAUGUAACUAUUCCCUUUCUGAGUAGAGUAAGUCUUACCAAGUACAAGUUCAUGGUGACCUUCGGACAGGUGAUGCAGUGGAGUUGCUGGAGGCUUU*

*>hsa-mir-548f-4*

*AAACUUAGAAACAUUAAAUAUAAAUUCUACAAUAUACAGUAUAAAAAUGAGUUCUAACGUAUUAGGUUGGUGCAAAAGUAAUAGUGGUUUUUGCCAUUAAAAGUAAUGACAAAAACUGUAAUUACUUUUGGAACAAUAUUAAUAGAAUUUCAGAGGAGAGAAAAGGGAGAAUGAUGGAGAAUCAAUUAAAAUACGUAACA*

*>hsa-mir-548aa-1*

*CAUGAGAAGAAAAUGCCACAAAUAAGAGAUAAGAAACAAAUAUAACCUUGUAUUUCUUUAUUAGUCUGGUGCAAAAGAAACUGUGGUUUUUGCCAUUACUUUUACAGGCAAAAACCACAAUUACUUUUGCACCAACCUAAUAUAACUUGUUUCAUAUUUGAAAUAAAUGUAGUAUUUAUUUUAACUGCUUAAAACAAUGU*

*>hsa-mir-3166*

*AUAUAUGACAAAUUGUACUUAUUUUGUCUUGUCUGUCUCCACCUACAAGAAGGUAAAUUUUUUUGAGGCCAGUAGGCAUUGUCUGCGUUAGGAUUUCUGUAUCAUCCUCCUAACGCAGACAAUGCCUACUGGCCUAAGAAAAAUUUACCUUCUUGUGGGUGGAGACAGGACAAGACAAAAUAAAUACAAUUUGUCAUAUA*

*>hsa-mir-520c*

*UUUAACCUGGUCAAGGAAGAUUCCCACAAAAAAUCCACAGUGCCAGAGCAAGAAGAUCUCAGGCUGUCGUCCUCUAGAGGGAAGCACUUUCUGUUGUCUGAAAGAAAAGAAAGUGCUUCCUUUUAGAGGGUUACCGUUUGAGAAAAGCAACAUUGAAGUUGAUGCUGAUCUUGGUAAUACAUUUUCAGAGCAUGCUUAUC*

*>hsa-mir-328*

*CUCGUGGAAGCUCUGGUCUUGGCCGAAGAACAUGGGUGAGAAGUCCUGGGCUGUCUCAGAGCCUGGAGUGGGGGGGCAGGAGGGGCUCAGGGAGAAAGUGCAUACAGCCCCUGGCCCUCUCUGCCCUUCCGUCCCCUGCUCUUGGCCAUGCUGUGCCCAGUCCAGGCCACUCACAGGGCUCCCCAACACGGAGCAGCUCA*

*>hsa-mir-335*

*GGUGCCAUUAACCUCAUUUUAUAGGUGAAGAAACUGAGAAACAGAUUGGAAAUGAUUUGUUUUGAGCGGGGGUCAAGAGCAAUAACGAAAAAUGUUUGUCAUAAACCGUUUUUCAUUAUUGCUCCUGACCUCCUCUCAUUUGCUAUAUUCAAUUAAGUAAGUAUACAAUUUUUAUUAUGUUGGCUUGGGUUACUUCCAGU*

*>hsa-mir-1469*

*UUCGCUCUGCGCGAGUUGGGUCUUUGUGAUAUAAAAUUCGCCGAGCGCCGCGAGCCGUGCUUUGCCAAUGGCGCGCUCGGCGCGGGGCGCGGGCUCCGGGUUGGGGCGAGCCAACGCCGGGGUUUCUUUGUGUUUCUGCGAGAGCGACUCUCCCGGUCCGGAGUCAGAUAACAGCCUGGGCCCGAGCCUCGCCGGCUUUC*

*>hsa-mir-450a-1*

*GUAUACCAAAAGUCUAGAGGUGGUUAUCGCUGACUUGUGUCACUAUAGGCAAUCAAAAACGAUACUAAACUGUUUUUGCGAUGUGUUCCUAAUAUGCACUAUAAAUAUAUUGGGAACAUUUUGCAUGUAUAGUUUUGUAUCAAUAUACAGAUGAAAAGAUGGAGGGAAUAAGCAAAAAAUCUAACAGCGGCUAUCUGUGG*

*>hsa-mir-943*

*UGCCCGCCAGGGGUUUGGCCCUUCCCCUCUGCGGCGCUAGCCUAGAAGAGGGUGGGACGUUCUGAGCUCGGGGUGGGGGACGUUUGCCGGUCACUGCUGCUGGCGCCCUGACUGUUGCCGUCCUCCAGCCCCACUCAAAGGCAUCCCGAAGCAGGCGCCCUUCAGAAGCCCCACGGCGCCCAGCGUCUUCAGCCCCACAG*

*>hsa-mir-1282*

*GCGAAGGGCAAGAGGGUUGGACACGAAGCCACAAAGCUACUUGGGUUCCUCCUUCUUCUCGUUUGCCUUUUUCUGCUUCUGCUGCAUGAUCUCCGAGUCCCUGGGGGUAGAGAUGAUGGGGCACUGGGAGGUACCAGAGGGCAAAAAGGACAAGAUGCAGGGGGAUGAAGGGCACAAAGGAUAAGAUGGAAGUGGAUGGG*

*>hsa-mir-3121*

*UCAAUGAAUGGCGCCAAUAGAAGCUAUGAUUGUCUCAUGAACAACCUUGUCUUAUUGCUUCUUAAAUGGUUAUGUCCUUUGCCUAUUCUAUUUAAGACACCCUGUACCUUAAAUAGAGUAGGCAAAGGACAGAAACAUUUUAAGUUCUCUAACACAGCAGUCCCCACCCUUUUUGGCACCAGGGACUGGUUUAGUGGAAG*

*>hsa-mir-29c*

*AAAUCAGACUUGCCACCUGGACUGUCGAGGUGCAGACCCUGGGAGCACCACUGGCCCAUCUCUUACACAGGCUGACCGAUUUCUCCUGGUGUUCAGAGUCUGUUUUUGUCUAGCACCAUUUGAAAUCGGUUAUGAUGUAGGGGGAAAAGCAGCAGCCUCGAAGCCUCAUGCCAACUCUGGGCAGCAGCAGCCUGUGGUUU*

*>hsa-mir-3668*

*UCUCUUCUAUUUAUAACUAUUUUACAACUAUUUAAAAAUGUCUUAGAGUUUAACUGGCCUAAAAUAUAUGAAAUGUAGAGAUUGAUCAAAAUAGUUUCUAUCAAAAUAGUUUUGAUCAAUCUCUGCAAUUUUAUAUAUGAGGAAACUGAAGUCUGAAAGUAUUAUGCCCUUUGUGUCUCUAUUUUGAAAGGCAGUGACAA*

*>hsa-mir-107*

*GUCUGAGCAUCCUUGCACAUAACUGCAGGUAAAAUGCUCGACUUGCAAUAUUCGAUAUUCUCUCUGCUUUCAGCUUCUUUACAGUGUUGCCUUGUGGCAUGGAGUUCAAGCAGCAUUGUACAGGGCUAUCAAAGCACAGAGAGCUUGCUACAGCCAAGGCCAGCCAGAGUCCCAGGCACAAAAAGGGGUGGAGCUGGUGA*

*>hsa-mir-580*

*UUUUAUUAUUUUGCACAUAUCUGAGUAAAAAUACAGAUAUGUGAAGUUAGAUAAAAUUUCCAAUUGGAACCUAAUGAUUCAUCAGACUCAGAUAUUUAAGUUAACAGUAUUUGAGAAUGAUGAAUCAUUAGGUUCCGGUCAGAAAUUUUUCAUUCAGUUUCAGAAUCUUUGGUUUUGACAAAAUUGUUGUCAGUGUUUUG*

*>hsa-mir-1246*

*AAUGUGAACCAUCAAUGAAGUAGGACUGGGCAGAGAUAAGAGACAUUGCAUUUGGAGGCGGUCAGAUUUGUAUCCUUGAAUGGAUUUUUGGAGCAGGAGUGGACACCUGACCCAAAGGAAAUCAAUCCAUAGGCUAGCAAUCAACCUAUUUUUUGUUUUUGUAGCAUUUGAUCGUUAUCGAGUUUUGCUGAAUCCUACUU*

*>hsa-mir-671*

*GCUUCUACAACGCUGACUACCUGGCGGCCCGAGCCCGGCUGGCAGGUGAACUGGCAGGCCAGGAAGAGGAGGAAGCCCUGGAGGGGCUGGAGGUGAUGGAUGUUUUCCUCCGGUUCUCAGGGCUCCACCUCUUUCGGGCCGUAGAGCCAGGGCUGGUGCAGAAGUUCUCCCUGCGAGACUGCAGCCCACGGCUCAGUGAA*

*>hsa-mir-29a*

*AAUGUAUGCUGGAUUUAGUAAGAUUUGGGCCCUCCCAACCCUCACGACCUUCUGUGACCCCUUAGAGGAUGACUGAUUUCUUUUGGUGUUCAGAGUCAAUAUAAUUUUCUAGCACCAUCUGAAAUCGGUUAUAAUGAUUGGGGAAGAGCACCAUGAUGCUGACUGCUGAGAGGAAAUGUAUUGGUGACCGUUGGGGCCAU*

*>hsa-mir-105-1*

*CUUCGUCCUCAGUAUCUACUCCUAUAUAUUGGAUGUCAGCUUCUGUUGCAGAACCUGAGUGUGCAUCGUGGUCAAAUGCUCAGACUCCUGUGGUGGCUGCUCAUGCACCACGGAUGUUUGAGCAUGUGCUACGGUGUCUACUUUUGCUACAUUGCCGUCUGCUUCUGGACCAAAGCCAUUUGUUCCUGUGCUCUGUGUUU*

*>hsa-let-7b*

*GGCAGGGGCUGGUGCUGGGCGGGGGGCGGCGGGCCCUCCCGCAGUGCAAGGCCGGGCCUGGCGGGGUGAGGUAGUAGGUUGUGUGGUUUCAGGGCAGUGAUGUUGCCCCUCGGAAGAUAACUAUACAACCUACUGCCUUCCCUGAGGAGCCCAGUGACACGACCCCAUGGGAGGGCCGCCCCCUACCUCAGUGACACGAC*

*>hsa-let-7c*

*UGGAGGAGCUGACUGAAGAUAUGAUAAGGAGUUUGAAGCAACAUUGGAAGCUGUGUGCAUCCGGGUUGAGGUAGUAGGUUGUAUGGUUUAGAGUUACACCCUGGGAGUUAACUGUACAACCUUCUAGCUUUCCUUGGAGCACACUUGAGCCGUCGAGGAAUUCUUCAUCACUUUAACCUGAUUGAGCCAAUUUGUGUGCA*

*>hsa-let-7e*

*AUCCCUGGUCCUCCUGGUCCCUGUCUGUCUGUCUGUCGGGUCUGUCCACCUGCCGCGCCCCCCGGGCUGAGGUAGGAGGUUGUAUAGUUGAGGAGGACACCCAAGGAGAUCACUAUACGGCCUCCUAGCUUUCCCCAGGCUGCGCCCUGCACGGGACGGGGCCCGGCGGGGACCCCCAGCCCCACUCAGGGACCCUUAGC*

*>hsa-let-7g*

*UCUCAAGUGCAUCCUGAAGAGUUCCUCCAGCGCUCCGUUUCCUUUUGCCUGAUUCCAGGCUGAGGUAGUAGUUUGUACAGUUUGAGGGUCUAUGAUACCACCCGGUACAGGAGAUAACUGUACAGGCCACUGCCUUGCCAGGAACAGCGCGCCAGCUGCCAAGUGGGGCUGAGAGGAUGGCGUCACCCUGCUCAUCUCUG*

*>hsa-mir-10a*

*CGAAGAAGGCGCGGAAAGUAGGAGAACUGGAAAAUUUCUGGGCCAAGAAGAUCUGUCUGUCUUCUGUAUAUACCCUGUAGAUCCGAAUUUGUGUAAGGAAUUUUGUGGUCACAAAUUCGUAUCUAGGGGAAUAUGUAGUUGACAUAAACACUCCGCUCUUAUUUUUCCAGAAGAAAAAAAUAUAUAUAUAUGUAUAUGUA*

*>hsa-mir-1197*

*GGCUUUCUGGAGGUGAUAGCAGGUGAAAGGAAGAGAGCCAGGGGUCAGCGUCACUUCCUGGUAUUUGAAGAUGCGGUUGACCAUGGUGUGUACGCUUUAUUUGUGACGUAGGACACAUGGUCUACUUCUUCUCAAUAUCACAUCUCGCCUUGGAAGACUUCCAGGAGGUGAUAUCAGCUUUGCGGAAGAGCCACUGUCCU*

*>hsa-mir-1204*

*UUAUUAGGAGAAAAACCUUCCCGGAAGCUGCAGAAGGACAAAUACAGAAUCCGUGUCUGGGAGAAACCUCGUGGCCUGGUCUCCAUUAUUUGAGAUGAGUUACAUCUUGGAGGUGAGGACGUGCCUCGUGGUCUAAAGCUUCGGCACAAGGGCCCAACUGGAAUUCCACUUACGGGUAUGACUGUGGGGUAUAUGCUGUA*

*>hsa-mir-122*

*AUCAGUGACAAUGGUGGAAUGUGGAGGUGAAGUUAACACCUUCGUGGCUACAGAGUUUCCUUAGCAGAGCUGUGGAGUGUGACAAUGGUGUUUGUGUCUAAACUAUCAAACGCCAUUAUCACACUAAAUAGCUACUGCUAGGCAAUCCUUCCCUCGAUAAAUGUCUUGGCAUCGUUUGCUUUGAGCAAGAAGGUUCAUCU*

*>hsa-mir-124-3*

*ACGCAAGCCCGAGCCGGUCCCGACCCUGGCCCCGACGCUCGCCGCCCGCCCCAGCCCUGAGGGCCCCUCUGCGUGUUCACAGCGGACCUUGAUUUAAUGUCUAUACAAUUAAGGCACGCGGUGAAUGCCAAGAGAGGCGCCUCCGCCGCUCCUUUCUCAUGGAAAUGGCCCGCGAGCCCGUCCGGCCCAGCGCCCCUCCC*

*>hsa-mir-1243*

*UUGGUUGACUCUGUACUCUUUUAUUUUAUUCCUGUAACUGGGCCAGUUUCUGCCCUUAACUAAAACUGGAUCAAUUAUAGGAGUGAAAUAAAGGUCCAUCUCCUGCCUAUUUAUUACUUUGCUUUGGUAAUAAAUCUAUUUUUAAAAGAACCUAUUACAGAGACUUCCAUUUGGAAUUAUGCAUACUUGGUGUCAAAUGG*

*>hsa-mir-1244-3*

*AUCUCAAAGGAGAAAAAAAAAACUUGUAAAAAAUGCAAAAAUGACAACAGAAAAACCAUCUUAUUCCGAGCAUUCCAGUAACUUUUUUGUGUAUGUACUUAGCUGUACUAUAAGUAGUUGGUUUGUAUGAGAUGGUUAAAAAGGCCAAAGAUAAAAGGUUUCUUUUUUUUCCUUUUUUGUCUAUGAAGUUGCUGUUUAUU*

*>hsa-mir-1251*

*UCAGUAAUUUCAUUGCACUGUGUAAUUAGGUGAAGAAACUCCACCCGAGCGUCCAUGGGUCAGCUAUGUGGACUCUAGCUGCCAAAGGCGCUUCUCCUUCUGAACAGAGCGCUUUGCUCAGCCAGUGUAGACAUGGCCUGAUAAACAAUGGAACUUCUCCCUUGAGAACUGGAGUUUGGAUUGAUCUAAUUCCUUAAUUC*

*>hsa-mir-1258*

*CCCCCUCCCGGUAGAGUUUGCACUUUGUCUCAAGGUCCCACUUGUAGAGGAGGGCGCCCAGGCGCCUGUGGCUUCCACGACCUAAUCCUAACUCCUGCGAGUCCCUGGAGUUAGGAUUAGGUCGUGGAAGCCACAGGAGCAUACUCCUCUGUUCCCCAGGGUAGGCUCCAAUUCCCAAGGACGGAUGGAGAGGGGAAAGG*

*>hsa-mir-125b-1*

*AGAAAUUGCCUGUCAUUCUUGUUUUGCUUUGCUUUGUCUCAAGAAAGAAAACAUUGUUGCGCUCCUCUCAGUCCCUGAGACCCUAACUUGUGAUGUUUACCGUUUAAAUCCACGGGUUAGGCUCUUGGGAGCUGCGAGUCGUGCUUUUGCAUCCUGGAAAUUUGGUGGAAUUUUAUUCUUUAAAGCAAAAACAAAAGAAA*

*>hsa-mir-1261*

*CUUUUCUGCUGCAGCUUCCUCACUUCUCUCAGCCUUCAUAGAACUGAAGAACUAGAGACUUGCUAUGGAUAAGGCUUUGGCUUAUGGGGAUAUUGUGGUUGAUCUGUUCUAUCCAGAUGACUGAAACUUUCUCCAUAGCAGCAGUGAGGCCAUUUUGCUUUCUUCUCAUUUGCGUGUUCACUGAAGUAACAUUUUUAAUU*

*>hsa-mir-1262*

*AUCUAUUGAUCAUAAACAAUUCUCUGAAUUUCAUCCUUCUACAAAUUCACCCCUCACAUUAUCUACAAUGGUGAUGGGUGAAUUUGUAGAAGGAUGAAAGUCAAAGAAUCCUUCUGGGAACUAAUUUUUGGCCUUCAACAAGAAUUGUGAUAUUUAAUUCUGUGUUUUCUGAUGCUUCUGAGGAAAGCUCCAUAAAGAGA*

*>hsa-mir-1266*

*UGUAACUGCCAUAACAGAACGGAGCCCAAAGCAGGCUCGUGUUGGCCUUGUUUGAUGCUAGACAGGUAGUGUCCCUCAGGGCUGUAGAACAGGGCUGGGAUUACUAAAGCCCUGUUCUAUGCCCUGAGGGACACUGAGCAUGUCAUUUCACUUCUCAGAGUCUUCAUCUUCUCUCUUUGAAAUGAAAAUAUUGGACCGGG*

*>hsa-mir-1284*

*UUGUAAAUUAUUUUAAUGCUGCAUUUCUGCUUAAACCUCAUGAUUUUGAUAUAUAAGCCAGUUUAAUGUUUUCUAUACAGACCCUGGCUUUUCUUAAAUUUUAUAUAUUGGAAAGCCCAUGUUUGUAUUGGAAACUGCUGGUUUCUUUCAUACUGAAAAUCUGUCUCGCGCGGCGUGAUGCCAUGUGGCAAAGGAGUAGU*

*>hsa-mir-1303*

*UUCCAACACUUUGGGAGGCCAAGUGGGAGAAACACUUGAGGCCGGGAGUUUGAGAUCAGGCUGGGCAACAUAGCGAGACCUCAACUCUACAAUUUUUUUUUUUUUAAAUUUUAGAGACGGGGUCUUGCUCUGUUGCCAGGCUUUGUGCAGCAUGUAAACUGUACAGCCCUAAGUGGGAGCCCUACUGCUAAUGCUUCCCC*

*>hsa-mir-130b*

*AGCCAGCCUGCAUUCCAGGUCUCAGAUCCCUGCAGACCACCCUGGGGGAGGCACUGGCAGGCCUGCCCGACACUCUUUCCCUGUUGCACUACUAUAGGCCGCUGGGAAGCAGUGCAAUGAUGAAAGGGCAUCGGUCAGGUCCAGCCUGCUACCCUGGGAGGGGGAAAGGGAGCUUGCUGCCUCACUCCACUUUCCAGUUG*

*>hsa-mir-1321*

*GGUCCACAAUAUGCUAGAUAUUUUAUAUUAUUUUUUGAUUACUUAAUGCAAAUAUAGCAACAUUAUGAAGCAAGUAUUAUUAUCCCUGUUUUACAAAUAAGGAAAUAAACUCAGGGAGGUGAAUGUGAUCAAAGAUAGAUAUAUAGUUAAGAAAGUUAAAGAGACAGGAUUCAAGCCUAAGUCUGUCUGACUUCAAAUCU*

*>hsa-mir-1324*

*GGACUCCAUGCACACAGAGGAUGCACACCUUGAGGCUGGACUAUGAGGAGAACAUUCCUGAAGAGGUGCAUGAAGCCUGGUCCUGCCCUCACUGGGAACCCCCUUCCCUCUGGGUACCAGACAGAAUUCUAUGCACUUUCCUGGAGGCUCCAUGCUGGUCUGUUCAUUUGGAAGUUUGAGGCUGUCCAUGAGGAAGUAAC*

*>hsa-mir-133b*

*CUUGAGACACACCAAGAUACCUGCACACUAGAGGCUGCAGUCACCUCAGAAGAAAGAUGCCCCCUGCUCUGGCUGGUCAAACGGAACCAAGUCCGUCUUCCUGAGAGGUUUGGUCCCCUUCAACCAGCUACAGCAGGGCUGGCAAUGCCCAGUCCUUGGAGAAACAGAAGAGAUUCAACUGCAACUGAAAUUACCUACUA*

*>hsa-mir-140*

*CCUCUCCAGGCUCUGCUUGGUGGGCUUCUGGUGUGGCCGCGCCCUGUGUGUGUCUCUCUCUGUGUCCUGCCAGUGGUUUUACCCUAUGGUAGGUUACGUCAUGCUGUUCUACCACAGGGUAGAACCACGGACAGGAUACCGGGGCACCCUCUGCGUCGACGGACUCCUCGUCUGCCCAGCCACAAACAGCCCAGCAGCUG*

*>hsa-mir-146b*

*GGGAGACGAUUCACAGAAGAAAGCAUGCAAGAGCAGCGUCCAGGCUGAAAGAACUUUGGCCACCUGGCACUGAGAACUGAAUUCCAUAGGCUGUGAGCUCUAGCAAUGCCCUGUGGACUCAGUUCUGGUGCCCGGCAGUGCUACAACAUCAAUGCCAAGGCCGUGGGGCAGCUGAUGGUUUGGGCUCCCAACUUCCCAGC*

*>hsa-mir-1470*

*CCCCCAGGGGUCGCAGAGGUGGGGGCUGCGCCGCGCGGGCCCACUCCUCGGGGGUCCAGGGUCCGGGGGAUGAGCCCUCCGCCCGUGCACCCCGGGGCAGGAGACCCCGCGGGACGCGCCGAGGUAGGGGGGACACCUGCCCCAAGACCCCCGCCCCUCCCCCCACCUGCACGGCCCCGCCCCCGGCCAGGCCCCGCCCC*

*>hsa-mir-1471*

*CGAGGGACUUGCAUUUCCUGUGGGUUCUGGGAUGGUUUUUAUCCCAUUCCCGGCUCAUCUCUCACGCGGACGAGCCCGCGUGUGGAGCCAGGUGUAGAGGCGGAGCACAGCUGGCUCUAAUUUGAGGGGCCUUCUGCCUUUAAAGAUGACCUCACUCCAUUCCCUUGACUUUUUAAUGUGCAAUUCAUGCCACUUUUCCA*

*>hsa-mir-150*

*GGGACUGGGCCCACGGGGAGGCAGCGUCCCCGAGGCAGCAGCGGCAGCGGCGGCUCCUCUCCCCAUGGCCCUGUCUCCCAACCCUUGUACCAGUGCUGGGCUCAGACCCUGGUACAGGCCUGGGGGACAGGGACCUGGGGACCCCGGCACCGGCAGGCCCCAAGGGGUGAGGUGAGCGGGCAUUGGGACCUCCCCUCCCU*

*>hsa-mir-15a*

*CUGAAAGAAAAUAUUUUUUAUAUUCUUUAGGCGCGAAUGUGUGUUUAAAAAAAAUAAAACCUUGGAGUAAAGUAGCAGCACAUAAUGGUUUGUGGAUUUUGAAAAGGUGCAGGCCAUAUUGUGCUGCCUCAAAAAUACAAGGAUCUGAUCUUCUGAAGAAAAUAUAUUUCUUUUUAUUCAUAGCUCUUAUGAUAGCAAUG*

*>hsa-mir-15b*

*UAUUCUUGUUACUUUUUUUUCUAUAAAGCUAGGUUGGAUGAAUCCUACAUUUUUGAGGCCUUAAAGUACUGUAGCAGCACAUCAUGGUUUACAUGCUACAGUCAAGAUGCGAAUCAUUAUUUGCUGCUCUAGAAAUUUAAGGAAAUUCAUUCAAAACUAUGUUUUCAUCAUCAGAUGUUCGUUUUAUGUUUGGAUGAACU*

*>hsa-mir-16-2*

*UCAAAACUAUGUUUUCAUCAUCAGAUGUUCGUUUUAUGUUUGGAUGAACUGACAUACUUGUUCCACUCUAGCAGCACGUAAAUAUUGGCGUAGUGAAAUAUAUAUUAAACACCAAUAUUACUGUGCUGCUUUAGUGUGACAGGGAUACAGCAACUAUUUUAUCAAUUGUUUGUAUUUCCCUUUAAGGUAACAUUUUAAAU*

*>hsa-mir-188*

*UGCCUCUUGCCUCCUAGAGCAUACCCAUAUGUCGUGCCAAGAGAGCAAGCCCUUCCCUGCUCCCUCUCUCACAUCCCUUGCAUGGUGGAGGGUGAGCUUUCUGAAAACCCCUCCCACAUGCAGGGUUUGCAGGAUGGCGAGCCUCAGCUUUCCUUGCUCUCUUGUGCAUGUGUAUAUACACAUACGCACUCCUUCACCAA*

*>hsa-mir-18a*

*ACAGCUGCCUCGGGAAGCCAAGUUGGGCUUUAAAGUGCAGGGCCUGCUGAUGUUGAGUGCUUUUUGUUCUAAGGUGCAUCUAGUGCAGAUAGUGAAGUAGAUUAGCAUCUACUGCCCUAAGUGCUCCUUCUGGCAUAAGAAGUUAUGUAUUCAUCCAAUAAUUCAAGCCAAGCAAGUAUAUAGGUGUUUUAAUAGUUUUU*

*>hsa-mir-191*

*AUGUGGCCCCAGGGCGAGUGACCUGGGGGCAGGAGCUCCCCCGCCCCCCGCCAACGGCUGGACAGCGGGCAACGGAAUCCCAAAAGCAGCUGUUGUCUCCAGAGCAUUCCAGCUGCGCUUGGAUUUCGUCCCCUGCUCUCCUGCCUGAGCAGCGCCCUGGCCCAGAUGGGGUGCCCCUGACCCCCAGACAUACUUUACUG*

*>hsa-mir-197*

*GCUCUGUCACACUUGCAGUCCCUGGCCCAACACCGAAAUCCUUCUGGAAUCUGUGCUCUGGGGGCUGUGCCGGGUAGAGAGGGCAGUGGGAGGUAAGAGCUCUUCACCCUUCACCACCUUCUCCACCCAGCAUGGCCGGCACACUUUGGUCUACGGCACAUCUCCAAGUAUAGAGUGGGUUUUGAAUGCUGUUAUGUCUC*

*>hsa-mir-20a*

*UAAUUUUGUGUACUUUUAUUGUGUCGAUGUAGAAUCUGCCUGGUCUAUCUGAUGUGACAGCUUCUGUAGCACUAAAGUGCUUAUAGUGCAGGUAGUGUUUAGUUAUCUACUGCAUUAUGAGCACUUAAAGUACUGCUAGCUGUAGAACUCCAGCUUCGGCCUGUCGCCCAAUCAAACUGUCCUGUUACUGAACACUGUUC*

*>hsa-mir-2115*

*AUAGGUGUGAGCCACCGUGCCUGGCCACCUCUCAUUUUCUUUCAGCAUUUUGACUGUCAUCCCACUGCUUCCAGCUUCCAUGACUCCUGAUGGAGGAAUCACAUGAAUUCAUCAGAAUUCAUGGAGGCUAGAAGCAGUAUGAGGAUCAUUUACAUGUGAUAUUCACUUCUCCCUUUCUGCUUUUUGGAUUCUCUCUUUGC*

*>hsa-mir-215*

*GUUUUGUAACACCAAAAAGAUCCAAUAAUGGAAGAGGAUUAAAGUCAUCAUUCAGAAAUGGUAUACAGGAAAAUGACCUAUGAAUUGACAGACAAUAUAGCUGAGUUUGUCUGUCAUUUCUUUAGGCCAAUAUUCUGUAUGACUGUGCUACUUCAAUAUCAGAAAUCGACUAACACCACGCAACCAACGCAAUGGCAGGU*

*>hsa-mir-216b*

*ACCAGAAAGAUCACUGUAGAAUGACGACAACUUUUUUCUAUCUUAAGUACAACUUCAAAGUGGCAGACUGGAAAAUCUCUGCAGGCAAAUGUGAUGUCACUGAGGAAAUCACACACUUACCCGUAGAGAUUCUACAGUCUGACAUCUUCAAGCAUCGAACCAUUUCAUCAACUAUGCUUUUGUUAUAAGCAGUUCUUUGG*

*>hsa-mir-2276*

*CUCAGUGCCUUCUGCUGCGGUUUUCCAUGCUCCAUCAGCAUCCCUGGGUUCCUUGUGUUCUUCCAGUCCGCCCUCUGUCACCUUGCAGACGGCUUUCUCUCCGAAUGUCUGCAAGUGUCAGAGGCGAGGAGUGGCAGCUGCAUCUCUGCAGGAGGACUCUGGAUUUCAGGACCCUCCCUUCUGCCACUGCUUCUGACUGA*

*>hsa-mir-2278*

*AUUGAGCCGCAGUCUUGGUAUCUGGAAGGCUAACAGGUCUUCCCAGCUGAUUGUGCUGCAGGUGUUGGAGAGCAGUGUGUGUUGCCUGGGGACUGUGUGGACUGGUAUCACCCAGACAGCUUGCACUGACUCCAGACCCUGCCGUCAUGUCACAGGUAAGAAGGUCAGGAAGAGGCUGGAGCGUGCCGGUGAAUGGGUGA*

*>hsa-mir-26a-2*

*CCUAAUCAUGACCUGGACAGACUGUCCUGUCGGAGCCAAGGACAGAAAGCUCCCAUAGAGGCUGUGGCUGGAUUCAAGUAAUCCAGGAUAGGCUGUUUCCAUCUGUGAGGCCUAUUCUUGAUUACUUGUUUCUGGAGGCAGCUGAUGGUCCGCCGCCGGAAACAGAGAUGGCUCCUGGGACAUGGUGUGUGCGCUUCUUC*

*>hsa-mir-302a*

*UGUGUUUUAAGGUUACUAAGCUUGUUACAGGUUAAAGGAUUCUAACUUUUUCCAAGACUGGGCUCCCCACCACUUAAACGUGGAUGUACUUGCUUUGAAACUAAAGAAGUAAGUGCUUCCAUGUUUUGGUGAUGGUAAGUCUUCCUUUUACAUUUUUAUUAUUUUUUUAGAAAAUAACUUUAUUGUAUUGACCGCAGCUC*

*>hsa-mir-302f*

*UAUGAAGUUUGUAUUCUUCAAUAAAACUUAAAUCACUAGUUAUUCUCUAAUACUAUAAUUGUCAAUGAUAAUGUCUUCUGUGUAAACCUGGCAAUUUUCACUUAAUUGCUUCCAUGUUUAUAAAAGACAUAAAAGAUGAUUAACAUCUGGUCUACUGUGGAACAGGCCUUGGAAAUUACUUAAAUAACUUUCCGUUGAUA*

*>hsa-mir-3074*

*UGGGAGCCCCAGCUCUCCUGAGCCUCGGGCACUUACAGACACGAAGGCUUUUUGCUCAAGGGCUCGACUCCUGUUCCUGCUGAACUGAGCCAGUGUGUAAAAUGAGAACUGAUAUCAGCUCAGUAGGCACCGGAGGGCGGGUCCAAUCGACAGCCCGGAGAAGCAGCGCCUCAGCUGGGAGCUCCGUGGGCACCGUCUGC*

*>hsa-mir-30e*

*UGUCACCUCCUUACUAGAGUAGGGUGUGCCUCACUGCGUCUCCGUUCUUUCUGGGCAGUCUUUGCUACUGUAAACAUCCUUGACUGGAAGCUGUAAGGUGUUCAGAGGAGCUUUCAGUCGGAUGUUUACAGCGGCAGGCUGCCACGGUCGUCCCCAGCUACGCACCGGCUUUGAAACAUUGCAGGUUUGUGCCCACAGGC*

*>hsa-mir-3139*

*AGGGCCUUUGGCUGCUGUGCUCCUGGGCUGGGUGCUGUGUCUUGAGCCCCUGUGCAAUGCCUGGCUCAGAGUAGGAGCUCAACAGAUGCCUGUUGACUGAAUAAUAAACAGGUAUCGCAGGAGCUUUUGUUAUGUGCCAUGUGUGGAAUGCAAGGAAGGAAAGGCCCACUCUCUGCCCUUAAGGAGUUUCUAAUCUGUUU*

*>hsa-mir-3145*

*UCUACCACUAACUCCAUGCAAAAACUAGUACAAUUUUGAAAGGAAAAAAAAUGUAUUUGUUUAUAUGAGUUCAACUCCAAACACUCAAAACUCAUUGUUGAAUGGAAUGAGAUAUUUUGAGUGUUUGGAAUUGAACUCGUAUACACUGAUACACCGAUACCAGUCAACUUCCCAUCUUCUAAAUUACUAAAUGGCAAAAU*

*>hsa-mir-3149*

*CUUGAGGUAUGUAGGAUAUCUUCAGGUAAAGAAAUUUAAAAAGGACAUCAAAAUGGGAUAUACAUACAUGUACACACACAUGUCAUCCACACACAUACAUAUAUAUAUGUUUGUAUGGAUAUGUGUGUGUAUGUGUGUGUAUACACAGAACCGACAGAUUUCCUCAUGUCUGAAAACAAUAACAUUUAAGAAUAUCUUAA*

*>hsa-mir-3153*

*CGUUUGAGUCCUGAAGCACCGUACUAGAACAUGGUGGCUGUAAUCGAUCAGUAAAAUUAGACAAAUUUUAAAUGUCCCUGUCCCCUUCCCCCCAAUUAAAGUAGAUUGGGGGAAAGCGAGUAGGGACAUUUAAAAUUUGUUGUCCUUACUGUAUGCGGGACACUUCAUUUAGGGCGUGGCUUCAGAAAACAGUCAACCCC*

*>hsa-mir-3160-1*

*CAUCCCUCCAGCCCAAAGCAACUGUCAAUCUGUUUGAAAAGUUGAUAAGUUUUUCAGUUGGACCUGCCCUGGGCUUUCUAGUCUCAGCUCUCCUCCAGCUCAGCUGGUCAGGAGAGCUGAGACUAGAAAGCCCAGGGCAGGUUCAUAUAACAGCCAAGCAGAAAGAUAGUAAUUGUGUCUCCAGGAUGAUGAGUGUUUAC*

*>hsa-mir-3162*

*UGUUUUAUAGUGAAUUUUUACAUAGUAAAAUCAUUUAAAAUUUCAAAGUUAAUUUUGAAGCUGACUUUUUUAGGGAGUAGAAGGGUGGGGAGCAUGAACAAUGUUUCUCACUCCCUACCCCUCCACUCCCCAAAAAAGUCAGCUUCUCUUGUUAACUUCUCUCUAUCCAGUGGGUAGCAUAGGGUCUGGCACACAUGGGU*

*>hsa-mir-3169*

*GUGACAGUGUUGAUAAGGUUGCUAAUAAUACUUUGUACUGUGACUUAUGCUACUAACAUAUGUGAAAACAUAGGACUGUGCUUGGCACAUAGCACAAAGUCUUAUGGUACUGUGUGCCAAGCAUAGUCCUGUGUUUUUACAUAUGUCAUCCCACAUGAAUCCUCACAAAACCUUGUAAUAUUUCAUACCAAUUUUACAAC*

*>hsa-mir-3170*

*ACAAGACUCCAUCUCAAAAAAAAAAAAAAAGCAGUUCUCAGAUCUUCACUCCAUAUGUCAUCUGGUAACACUGGGGUUCUGAGACAGACAGUGUUAGCUCCAGAAGCAUUGCCUGUCUUAGAACCCCUAUGUUACCAGACGACAUAAGGAGUGAAUAAAAUCUUCAAAAGGCUUUGAUGGACCGUAGGUGAGGAAGGUGA*

*>hsa-mir-3178*

*CAAGGCCCCGCGUGCCGCAACGCUCCCAGAGUUGCGCGUCAGCCUGGACUCAGCGCCUGCGCUACGAGGCUGGGCGGGGCGCGGCCGGAUCGGUCGAGAGCGUCCUGGCUGAUGACGGUCUCCCGUGCCCACGCCCCAAACGCAGUCUCUGGCCUGGAUGUGCUCGGAGCCCACGAGGUGCAGAGGCUCGGACGGCGAAA*

*>hsa-mir-3194*

*UUUAGAAGGCAGAUGUCACUGAGGUUUGAUGGGACGUAGUGGUGUAGAGAGGCAUUAAGAACGCAGGUGGCAGGGCCAGCCACCAGGAGGGCUGCGUGCCACCCGGGCAGCUCUGCUGCUCACUGGCAGUGUCACCUGCGGAAACUCUCCAUCAUCUAUCCCCUGAAAAGCAGGGACAGUGACAGUGCCUACCCACAAAG*

*>hsa-mir-320e*

*UACACUCCAGCCUGGGCAACAAGAGCAAAACUCUGUCUCAAAAAAAUGAAAAGAAAAGAAAAUACCUCCAUGGGGCCUUCUCUUCCCAGUUCUUCCUGGAGUCGGGGAAAAGCUGGGUUGAGAAGGUGAAAAGAAAAAACAAACCUUGACUGGGCACAGUGGUUCACACCUGUAACCCCAGCACUUUGGAGGCUGAGGCA*

*>hsa-mir-33a*

*CAAACAGAGCUGAAGACCACCCUGGGCACCUCCUUGGCUGGCCGCAUACCUCCUGGCGGGCAGCUGUGGUGCAUUGUAGUUGCAUUGCAUGUUCUGGUGGUACCCAUGCAAUGUUUCCACAGUGCAUCACAGAGGCCUGCCUGGCCCUCGAGAGACUGCCCUGACUGAAGGCCCUAUCAGGUGGGGGAGGGGAUCCUGAU*

*>hsa-mir-33b*

*AUCAGGAGGGCUGGACAGCUGCUCCCGGGCCGGUGGCGGGUGUGGGGGCCGAGAGAGGCGGGCGGCCCCGCGGUGCAUUGCUGUUGCAUUGCACGUGUGUGAGGCGGGUGCAGUGCCUCGGCAGUGCAGCCCGGAGCCGGCCCCUGGCACCACGGGCCCCCAUCCUGCCCCUCCCAGAGCUGGAGCCCUGGUGACCCCUG*

*>hsa-mir-34b*

*GGCCCCGACCCCGCGUCGGCGCUGCGGACCGUCCGGGAGCUGCAGCCGCGGGUGCCCGGUGCUCGGUUUGUAGGCAGUGUCAUUAGCUGAUUGUACUGUGGUGGUUACAAUCACUAACUCCACUGCCAUCAAAACAAGGCACAGCAUCACCGCCGCCCGGCCGGGAAGAAGACGCCGGCUCGGGCAGCCCGCAGCCUUCG*

*>hsa-mir-3606*

*UUUCAGCAACACACGAACCCUUUUUAAAAGUUCAAAUGACGUCCUCUCUUUGUAACCAAAAUAUUGUUGCUAUCUAGGUUAGUGAAGGCUAUUUUAAUUUUUUUAAAAUUUCUUUCACUACUUAGGGUCCUGCUGGAGAGCGUGGUGCUCCAGGCCCUGCAGGGCCCAGAGGAGCUGCUGGAGAACCUGGCAGAGAUGGC*

*>hsa-mir-3610*

*CACAGGUACCAUUUUGACCGUAAACAUCCUGCCGAUUUGAACCGAGGAUUUGGGCGGCAGGAAGAGCCGCGGCGUAACGGCAGCCAUCUUGUUUGUUUGAGUGAAUCGGAAAGGAGGCGCCGGCUGUGGCGGCGGCGGGAGCUGCUCGGAAGCUACACCUCGCAAGGGCUCCCCCCUUUCCCCACCCCCUCCCCCGACCC*

*>hsa-mir-3615*

*CCGCGGAGGUCCCCACCCUCAGCGCGGCCCCGCCCCCGGGGUAAGGAGCCGGGGCGGACUCUGGGACGCUCAGACGCCGCGCGGGGCGGGGAUUGGUCUGUGGUCCUCUCUCGGCUCCUCGCGGCUCGCGGCGGCCGACGGUUCCUGGGACACCUGCUUGCUUGGCCCGUCCGGCGGCUCAGGGCUUCUCUGCUGCGCUC*

*>hsa-mir-3646*

*CUUGCCAGGCUUCCUGCUACCAGCAGGUAUCAGUCACUGGGCUGUGCUUUUUCCACACUUCAGUAGGUUGGGUUCAUUUCAUUUUCAUGACAACCCUAUAUGGGAAAAUGUUGUGAAAAUGAAAUGAGCCCAGCCCAUUGAACCUAUUACAAUUCCUCCCUUUUUGUGGAUGAAGGAACUGAGGCCCACAAUGGUUAAGC*

*>hsa-mir-3657*

*UUGUCCUUUUUAGUGAGUUAAAUCAGGAGUCACUAAUGUUGUUGUGUCCCAUAAUUAAAUAAUGAAAUCUGAAAUCACCAAUAAUGGGACACUAAUGUGAUUAAUGUUGUUGUGUCCCAUUAUUGGUGAUUUCAGAUUUCAUAUAUGAUUAAGGACAUAUCUACCAGAUUUCUUUAUAAAGGUGCCUUUUUCCCUCUGUA*

*>hsa-mir-3661*

*UUUCCUCGGCGGGCUCGGCCCCGCGGCGGCUGUCCGCAGCCGUACUACAGCUCACCUUCUCGCAGAGGCUCUUGACCUGGGACUCGGACAGCUGCUUGCACUCGUUCAGCUGCUCGAUCCACUGGUCCAGCUCCUUGGUGAACACCUUCUCGUCCAUGAUGCCACCCGCCCCAGCCGGCUGCCGCUCCGCGCUGCUCCCG*

*>hsa-mir-3665*

*CGGCCGGGGGGCUCCGAGGACCCAAAGGCUGCAGGCCAGCGGGGGGCGGCGCGGCGGGCGGCGGCGGCGGCAGCAGCAGCAGGUGCGGGGCGGCGGCCGCGCUGGCCGCUCGACUCCGCAGCUGCUCGUUCUGCUUCUCCAGCUUGCGCACCAGCUCCUGCAGCUUCUUCACCUCCAGCUCCGCGUUCACCACCGGCCCG*

*>hsa-mir-3671*

*UUCUUUCAGGAUAUUUAGGAUCUGGCCUAAUUCAAAAGAAUAUUUACUGAAUGCCUAUGUUAUUGCUGCUGCUGUCACAUUUACAUGAAAAUAAAAUGUAAAUUAUUUUAUUUCUAUCAAAUAAGGACUAGUCUGCAGUGAUAUCUGGCACAUAUCGAUUUCAGGAAUACGAAGCUGUAAACACCAAGCCAGCAUCACGU*

*>hsa-mir-3682*

*UCAUAGCCCCCGUCCGUAUCUAUAUCUCUAUAUCUAUAGAGACAUAACUUCUUUCUUCGUAAGUUAUAUAUGUCUACUUCUACCUGUGUUAUCAUAAUAAAGGUGUCAUGAUGAUACAGGUGGAGGUAGAAAUAUAUAACUUAUCUCUUCAUAAGGGCUAAAAUGAGAACUAAAAGUAAUGAGAAUUCAGUUGAAUAUGA*

*>hsa-mir-3684*

*UAUAAUUUGGAAAAACUUGCACGUGGAGGAGUGUUGCUUGAUCUAAAAAUAGCAUUACCGAACAAUCUAAAGGACCUGUACUAGGUUUAACAUGUUGAGCAUUACUCAUGUUAGACCUAGUACACGUCCUUUAGAUUCUUUAAAAUUCCCAAAUUCUAUCUUGUUGUGGCCUGUGUGGGACCUGAAAUUAAGAAACAGUG*

*>hsa-mir-370*

*CGGUUGGACCCACGAGGGCGCUGCAUUCCUCAUUCUACAAACCGUACAAGUCGGGGCACAAGACAGAGAAGCCAGGUCACGUCUCUGCAGUUACACAGCUCACGAGUGCCUGCUGGGGUGGAACCUGGUCUGUCUGUCUGUCUAACACCAGAGCUCGGGCGCUGCUGCAGAGGGAGCCAAGAUUUGGGUGAGGGAGGGGG*

*>hsa-mir-381*

*CCCGGGAGAGGAGGCCGGCCCCGUGAAUGAUAGUGAGGAACCUGCCCAGUGCUAUUGUUUGGUACUUAAAGCGAGGUUGCCCUUUGUAUAUUCGGUUUAUUGACAUGGAAUAUACAAGGGCAAGCUCUCUGUGAGUAUCAAACCUUGUCUUGGACCCAGUCCACACUCAGCAAGGGAUGCGGUAUGUGUGUGCAUGACCG*

*>hsa-mir-382*

*GCCACUCCCCUUGGAGAAGUGGAAGGGGACUCCUUGUCUGUCUUGUCUCUGCUUUUCUGUGGUACUUGAAGAGAAGUUGUUCGUGGUGGAUUCGCUUUACUUAUGACGAAUCAUUCACGGACAACACUUUUUUCAGUACCAAAUGCUACCUCUAAGGACUUCCUGGACACAAUGGCAGCUUCAGGAAAGAUAGUCUUUGU*

*>hsa-mir-383*

*UGUGUAUUGUUUGAUUAUGUUUGCAUAAUUUAUCAAGAGACUUCAGAAUCUCCCCGUCACCUGCUCCUCAGAUCAGAAGGUGAUUGUGGCUUUGGGUGGAUAUUAAUCAGCCACAGCACUGCCUGGUCAGAAAGAGCAAGUGUCCUAGCCUUUACCUCAUGAGACCUUGGGUAUCCAUCCAGCGUAGGUCCCAAGGGAAC*

*>hsa-mir-3907*

*AGCUGUGGCACAGGUGUGGGGUUGGAAAGCUGUAGGUGUGGAGGGGCAUGGAUACGGGGGCCAUGAGGGUGGGGUCCAGGCUGGACCAGGCCUGCCCUGAGUCCCCCAGCAGGUGCUCCAGGCUGGCUCACACCCUCUGCCUCUCUCUCUUCCUUCCUGGCCCCAACCCUUGAGGCAGCAGACUGUGCCAGGCACCAGGC*

*>hsa-mir-3909*

*GGAGAACCCUGGCGCCUGCCCUGGUCACCAGCCUGGCUGAGGUAUGCUGUUGCGCUGUCCUUCCUCUGGGGAGCAGGCUCCGGGGGACAGGGAAAAGCACACAAGGAACUUGUCCUCUAGGGCCUGCAGUCUCAUGGGAGAGUGACAUGCACCAGGACCACCGCUGUGAAGGGCUAAGUGCUGUCCUGGAGGGGGCACCA*

*>hsa-mir-3921*

*UCACUAUAAUGUAAGUGGUUUGGCCUUGAUCCUGUUUAAGGACCCAUCCUAAACAUCACCUAGCCCAGUACAAGGCAUAUGGUACUCAAGAGACUUAGAAAUCCCUAAGUCUCUGAGUACCAUAUGCCUUGUACUGGGCUAGGGAGAAACUAGACACAGACACUGUCCCUAUUCUCAUGGAACCUGUAACCUAGCUAGAG*

*>hsa-mir-3922*

*UUCUCUUUAUCAAUGUUUAACAUAUUCCAUUCAUAAGCACCCUCAAAUUGAACUUAAAGGAAGAGUCAAGUCAAGGCCAGAGGUCCCACAGCAGGGCUGGAAAGCACACCUGUGGGACUUCUGGCCUUGACUUGACUCUUUCCAGAUAUCUGAGUCAGACGUUUUCAAAGCAUGCACCAUGACCCACGGGUGUGUUGUAA*

*>hsa-mir-3926-1*

*AUGAAAUUUUAAGUUCUGUUAAAAUUAUCACAGUAAAGUUCCUUGACUUUAAAAGCUUGCUUUUAAAAUGGAGCUGGCCAAAAAGCAGGCAGAGACUUUAAAAGCGUCUCUGCCUGCUUUUUGGCCAGCUCCGUUUUUCAUCACUGACGCUUAUAAUUGUUAUCAUUCCUUGUUUACCAGAGCCUGUCAUUUGUUUUCUU*

*>hsa-mir-3943*

*GGCCAGGGAGCGGGUGAUGAAGGACUUCAGCAUCUUUAUCAGCGCCCCCCCACACAGACGGCAGCUGCGGCCUAGCCCCCAGGCUUCACUUGGCGUGGACAACUUGCUAAGUAAAGUGGGGGGUGGGCCACGGCUGGCUCCUACCUGGACAGGCCUCCAGCUAACUCCGGGCUUGCACAGCAAAUACAAGAAAAAAAGAA*

*>hsa-mir-3945*

*AUGUACUCAAAUAUCACCUCAAAUAAGAUGAGGUCUUUCUGAGAGAAAAGAGAUGUUGAUGCACGUGACGGGGAGGGCAUAGGAGAGGGUUGAUAUAAAAUGCAAUUACAGCCUCUUAUGCUUUCCAAAGUGGGGGAUGAUUCAAUGAUUCCUGACUGGUCCACAGCCAUAUAUCAAUUGAGGUUAUAAUACUAAUUUGA*

*>hsa-mir-4251*

*CAGAAGCCUGGGUGCUGUGGCCAUGGAGGAGGGGCUCCCGCCUCUGGUCAGCCUUCUGGCAUCUGGGAACACGUCCUCCAGCUUUUUUCCUUAGUGGCCAAUUCCUGAGAAAAGGGCCAACGUGCUUCCAUCCACUUCAGAGGGCACUUGUAUGAAAAUCUCCUCCCCUCCCCCGUCAGCCCCGCUGUGGAGGGAUCAAC*

*>hsa-mir-4259*

*GCAGCAGAAGUCCGGAGUCAGGGCGUGUGGCUGAGGAGAUGGUGAGGGAGAACCUCAGAUGGGCCCCUUGUGUCCUGAAUUGGGUGGGGGCUCUGAGUGGGGAAAGUGGGGGCCUAGGGGAGGUCACAGUUGGGUCUAGGGGUCAGGAGGGCCCAGGAGUAAGGAGAACACCUCUAGAAGAAGCAAGGGAGAUCCUGAGG*

*>hsa-mir-4283-1*

*GAAGCAGGUGGUGUCCUGUCCCGGCAAAGGCAGACAUGGGGCUGACACAGAACGGCCCAGGGACUCUGAUCCUGGGGCUCAGCGAGUUUGCAAGGGGUGUUUCUGUCCAUGGUCAGGCUUGCCAGCCUUGGUCCUUGGGCCCACCAUAAGGUGGCCCAGUCCUGACCCUGUCUUGGAAUUGCUGAGAGCAGAAAUGCAGU*

*>hsa-mir-4288*

*ACUCAAGAGUGUGCUGGGUUUGCUCCAAUUACUUGUGCCUGCUAAGCUGGUUCCUGGAUCAGUAGAGGGAUGGAGGUGGAGAGUCAUCAGCAGCACUGAGCAGGCAGUGUUGUCUGCUGAGUUUCCACGUCAUUUGCUAUUAAAGAAACGUUACACUCUCAAACUGUGUUUGAUUAUUCUUUCUGUCAGUGGCAUCACUG*

*>hsa-mir-4303*

*GGAGUUCAGUUCUUCCACCUGGCUCUCAGGGGAAUGCUGAAAUGAGAUCUUGGUUGACAUGAAAUUUACAGAAAAUAGCUUCUGAGCUGAGGACAGCUUGCUCUGCUUUUCUUUAGCUUAGGAGCUAACCAUGGUCUUGGUUGAGGCUUGGGCUUCUGUAAUUCCUUCUCUGGUAUUUUGCAUAGUGAUUAAGUGUUUAG*

*>hsa-mir-4305*

*UGUUGGAAUACCACAAUAAAGAGUCAAGCUUCUGCUACUGCGGUUUCUUUCUAGAGCUGCCUUAGACCUAGACACCUCCAGUUCUGGGUUCUUAGAGGCCUAAUCCUCUACAAACUCAGUUUUCAGACUGUGAGGGAAAUUCUCUGUCUUAUUGCUUUCUAGGUAUGCUGUCAAUAACAGAGCCUGAUUGCCACCUCAAA*

*>hsa-mir-4307*

*GGAGAGAGAGAAAGGCACCUAGGGAAAACAGAUGACUUUUUGGAAAGAUAAAUGGGCUUUCAGAAGAAAAAACAGGAGAUAAAGUUUGUGAUAAUGUUUGUCUAUAUAGUUAUGAAUGUUUUUUCCUGUUUCCUUCAGGGCCAUAAUACUCCCCUGGAGAAGGGGUUUGAGGUAGGUUUUAUUUACAUUUUCCCUUCUGG*

*>hsa-mir-4317*

*UGUUAGGCACAAUCCCACAAAAGAAAUGUGGACUUUGCGGAUGAAGUAGACCUCAAUGUUGCCUUGCAUCUAAAAGGCGAGACAUUGCCAGGGAGUUUAUUUUGUAGCUCUCUUGAUAAAAUGUUUUAGCAAACACUAGCGUGCCAGGCUUGGUCCUAAGCCCUUUGCCGGUGUUAACCCAUGUAAUCCUCGUGACAGCU*

*>hsa-mir-4327*

*GGUGCCAGGGCCUGGAAAAGUCUCAAACAAUGAGGCCGGAUGACCUCCGAGAAAGCCACAGGCCUGGGUAGGCUUGCAUGGGGGACUGGGAAGAGACCAUGAACAGGUUAGUCCAGGGAGUUCUCAUCAAGCCUUUACUCAGUAGUGGGUUCUUCAUAGUUAGCAUUAAUGUUUAAUAGAUACAUUUCCCCUUGGGUGAG*

*>hsa-mir-4329*

*ACGUGUUCAAAGAGAGCCCUGAGAUAAGCACCCAUGCUUAAGGAAAAGGGAUGAUGGUCCAGGUUCUAGAGAGGAAGGUGUACCAGGGUUUUGGAGUUUUUUUUUCCUCCUGAGACCCUAGUUCCACAUUCUGGAGCUCCUCCACACUUUGAAGUCUCCCGGUGAGAAUACCGAUUGAGGCCAAGGCUUAGAGGACAAGA*

*>hsa-mir-449a*

*UACCCUGCCACUUGCUUCUGGAUAAAUUCUUCUUGUCAAUGAAGUGCUCUGGAUACCUGUGUGUGAUGAGCUGGCAGUGUAUUGUUAGCUGGUUGAAUAUGUGAAUGGCAUCGGCUAACAUGCAACUGCUGUCUUAUUGCAUAUACAAUGAACAUCAGAGUGUAACUGAAUCUGUAAUUAGUGUGUGUUUAUGUGUACUU*

*>hsa-mir-487a*

*GGACUAGCUGGAAGGAAGGCAGACUCUGGUGAAGACGUGGGCCGUCACCGUCAGCCUUCGGUACUUGAAGAGUGGUUAUCCCUGCUGUGUUCGCUUAAUUUAUGACGAAUCAUACAGGGACAUCCAGUUUUUCAGUAUCAAAUACUGCUUUGGAAGGCUUCCCGGACAGCGUCCCGCCUUCAGGCAAGGGGUCUUGGCUU*

*>hsa-mir-509-3*

*AUGGUUGUUUCAAAGGUGAAUGGGUGGGUAUUAAGGCAAGGCUGCCAUCCUCAGACAUGCUGUGUGUGGUACCCUACUGCAGACGUGGCAAUCAUGUAUAAUUAAAAAUGAUUGGUACGUCUGUGGGUAGAGUACUGCAUGACACGUGCAACAUACAUGAUGACACUGUGUGUGUGUUGGAGGCAUUUAGUUGCAUGCAG*

*>hsa-mir-510*

*UUUGAAAAGUGUGUGAUUAAGUAUUUAGGCUGUGCUCCCAUUUUCAGGAAUGCUGUGUAUGUGUGUGGUGUCCUACUCAGGAGAGUGGCAAUCACAUGUAAUUAGGUGUGAUUGAAACCUCUAAGAGUGGAGUAACACAUGACAUGUACUACAUAUGCAAUGAUAUUGCAGAUAUUUAGUUGCAUGCAGUGGUAUGCUUU*

*>hsa-mir-518d*

*UUUAACCUGGUCAAGGAAAAUUCCAACAAGAAACCCAGAGUGCUGGAGUGAGAAGAUCCCAUGCUGUGACCCUCUAGAGGGAAGCACUUUCUGUUGUCUGAAAGAAACCAAAGCGCUUCCCUUUGGAGCGUUACGGUUUGAGAAACUCAAUGUUGAAGUUGAUGCUGACUUCAGUAAUACAUUUGUAGAGGAUGCUUAUC*

*>hsa-mir-518e*

*CAUUUUAACCCGGUGAAGGAAAAUUCCAACAAAAAACCCACAAUGCUGGAGCAAGAUCUCAGGCUGUGACCCUCUAGAGGGAAGCGCUUUCUGUUGGCUAAAAGAAAAGAAAGCGCUUCCCUUCAGAGUGUUAACGCUUUGAGAAAAGCAACGUUGAUCUUGGUAAUACACUUGCAGAGAAUGCUUAUAAUCAACCAUGG*

*>hsa-mir-519c*

*UUUAACCUGGUCAAGGAAAAUUCCAACAAAAAAUCAAUGGUGCUGGAGCAAGAAGAUCUCAGCCUGUGACCCUCUAGAGGGAAGCGCUUUCUGUUGUCUGAAAGAAAAGAAAGUGCAUCUUUUUAGAGGAUUACAGUUUGAGAAAAGCAACGUUAACGUUCAUGCUGAUCUCGGCAAUACAUUUGCAGAGCGUGCUUAUC*

*>hsa-mir-519e*

*CAUUUUAACCUGGUAAGGAAAAUUCCAACAAAAACCCAGAGUUUUGGAGCGAGAAGAUCUCAUGCAGUCAUUCUCCAAAAGGGAGCACUUUCUGUUUGAAAGAAAACAAAGUGCCUCCUUUUAGAGUGUUACUGUUUGAGAAAAACCACGUUGAAGUUGAUGCUGAUCUUGGUAACGCAUUUGCAGAGCGUGCUUAUCAU*

*>hsa-mir-520h*

*UAACCUGGUCAAGGAAAAUUCCAACAAGAAACCCAGAGUGCUGGAGCAAGAAGAUCCCAUGCUGUGACCCUCUAGAGGAAGCACUUUCUGUUUGUUGUCUGAGAAAAAACAAAGUGCUUCCCUUUAGAGUUACUGUUUGGGAAAAGCAGUGUUGAAGUUGAUGCUGAUGUUGGUAAUAUAUUUGCAUGCUUAUCAUCAGA*

*>hsa-mir-526a-1*

*UUUAACCUGGUGAAGGAAAAUUCCAACAAAAAACCCACAGUGCUGGAGCAAGAAGAGCUCAGGCUGUGACCCUCUAGAGGGAAGCACUUUCUGUUGCUUGAAAGAAGAGAAAGCGCUUCCUUUUAGAGGAUUACUCUUUGAGAAAAACAACAUUGAAGUUAAUGCUGAUCUUGGAGAUCAUGCUUAUAAUCAGACUUGGA*

*>hsa-mir-542*

*UAAAAAGUCUUUAGGGGUCAUGGGUGAUACAGAAAUUUGGGAUCGGUCAAGGAUGCACAGAUCUCAGACAUCUCGGGGAUCAUCAUGUCACGAGAUACCAGUGUGCACUUGUGACAGAUUGAUAACUGAAAGGUCUGGGAGCCACUCAUCUUCAUAGCAAAACAUGUGGUAAGUAUUACAAGACGAAUCAAUGAAAAGAU*

*>hsa-mir-548j*

*ACCCCUGCACUCCACGAUGAUUGCAUUCCUGAGCCUGGCCCAGGUGGGCAGCCAGUGAAUAGUUAGCUGGUGCAAAAGUAAUUGCGGUCUUUGGUAUUACUUUCAGUGGCAAAAACUGCAUUACUUUUGCACCAGCCUACUAGAACGCUGAGUUCAGUGCCUUAAGCGGUGUUUCACAGAGAGGGAGCAAGGGGCAGCGC*

*>hsa-mir-548l*

*UCUGGCCCAACCACUAGUUGUAUGACCUUGAGCAACCGCAUCCCUUAUCCCUUGGUUUGUAUUAGGUUGGUGCAAAAGUAUUUGCGGGUUUUGUCGUAGAAAGUAAUGGCAAAAACUGCAGUUACUUGUGCACCAACCAAAUGCUUCGUAGAUUUAUGAGAUCUAAAUGGAUUAAAUAGUGUCUGUUGCACAGUAGGUAA*

*>hsa-mir-548m*

*UUACGAUUUUAUUUUCUUGUUGGUUCAUAUGAGUUUUUUUUGUUGGUGUUUAUUCUAGAUAUUAGGUUGGUGCAAAGGUAUUUGUGGUUUUUGUCAUUAAAGUAAUGCAAAAGCCACAAAUACCUUUGCACCAACCUAAUAUUAGCCCCUCGGAAUUAUAGACAUGGCAAGUAUCUACUAUCAGUAAGUUGACUGGAGUA*

*>hsa-mir-548t*

*UUGCUUGUAAGUGCUGGGUGAUUGAAUCUAUAAUUUGCGUCAAGAGAAGUGAAGAAAAAUGUUAGGGUGGUGCAAAAGUGAUCGUGGUUUUUGCAAUUUUUUAAUGACAAAAACCACAAUUACUUUUGCACCAACCUAACCUUGUUUUAGGCAUGUAUCUAUUUAAAGACACUUUAUUUUACCUAUAACGUUGAUGGGUU*

*>hsa-mir-553*

*CCUUAUAUGUUGAGCAAGAAGAUUAGCCUGGAGAAUCUCCAAAAUCCUUCUAACUAUAACAUUCUUCAAUUUUAUUUUAAAACGGUGAGAUUUUGUUUUGUCUGAGAAAAUCUCGCUGUUUUAGACUGAGGCAGUGUGUUAUGAUAGAGAGCACUAAACAGAGAGUGAGAAGACCUAAAUUUUAGUCCCAUGUCCUGUCA*

*>hsa-mir-574*

*CAGGGAGGACCCGGCUCUGGGGUGAGGGUCUGGGGCGGCGCGGCCGAGGGACCUGCGUGGGUGCGGGCGUGUGAGUGUGUGUGUGUGAGUGUGUGUCGCUCCGGGUCCACGCUCAUGCACACACCCACACGCCCACACUCAGGGUCUGCCCCCUCGGCCUGCGUGAACCUCCGCGGAGCCUGCCUGGAUCUCCCAAAGUA*

*>hsa-mir-577*

*UUGGUAAAUAACUGUGACCUGAGGGGGAAGUUUCAGGCACCCAAGGCAGGCUGUAUUUGGGGGAGUGAAGAGUAGAUAAAAUAUUGGUACCUGAUGAAUCUGAGGCCAGGUUUCAAUACUUUAUCUGCUCUUCAUUUCCCCAUAUCUACUUACUGUAGGUACGGCCUCAUUAAAAAAUAUAAACUCUCUGAAGACAGAAC*

*>hsa-mir-578*

*CAACAAUUAUUAUUACCUCAUUAUGGUAUUUCCCACUGUUGGUCUACAGAUAAAUCUAUAGACAAAAUACAAUCCCGGACAACAAGAAGCUCCUAUAGCUCCUGUAGCUUCUUGUGCUCUAGGAUUGUAUUUUGUUUAUAUAUAUUUUUUAAUCCUGAAUGUCUAGUAUUAUGCUUGGGACAUAGUGGCCACUCAGUGAA*

*>hsa-mir-579*

*CUGUCUCUAUAGAUUUGCCUAUUCUAGAGAUUUCAUAUAAAUGGAGUCAUAUUAGGUUAAUGCAAAAGUAAUCGCGGUUUGUGCCAGAUGACGAUUUGAAUUAAUAAAUUCAUUUGGUAUAAACCGCGAUUAUUUUUGCAUCAACUUACUACAGUCUAUGGUCUUUUGUGUCUGGCAUCGUUCACUUAGCGUGUUUUCAU*

*>hsa-mir-587*

*AAUACCCAGGCAAGAGAGAGUUGCUGUCCUUUAAAAGGUCUCUUUCUGCCCCUCCUAUGCACCCUCUUUCCAUAGGUGAUGAGUCACAGGGCUCAGGGAAUGUGUCUGCACCUGUGACUCAUCACCAGUGGAAAGCCCAUCCCAUAUCCCACCUAAACCACUCUUUGGUGCCCAGGAUCCCAAGGGCCCUGAACUCACUU*

*>hsa-mir-588*

*AACACCAAAUUGAACAACUAUUUAAAUAAAAAAGCACCUUCAUGAGAACCGAAAAUCAGCUUAGGUACCAAUUUGGCCACAAUGGGUUAGAACACUAUUCCAUUGUGUUCUUACCCACCAUGGCCAAAAUUGGGCCUAAGGGGUCCCAAUUCCCGGCCUCAGCUCUUGGAUGGAAUUUCUAGACCUGCCCUGGGCCAGCG*

*>hsa-mir-591*

*AUAGUGUAUGAAUCUUGAGAAUUGAUAGAUCUGUCAGAUUAUAGAGGAGGAGGGAGUCUUAUCAAUGAGGUAGACCAUGGGUUCUCAUUGUAAUAGUGUAGAAUGUUGGUUAACUGUGGACUCCCUGGCUCUGUCUCAAAUCUACUGAUUCAUAAUUUUUGGGUGUGGGGCUUGGGAAUGUGUUUUAAACAAGCUCUCCA*

*>hsa-mir-607*

*AACCCAAUCGUGUAUCCCAUUUUAUAACAGAGCACAGAGAGGUUAAGUAACUUGCCUAAAGUCACACAGGUUAUAGAUCUGGAUUGGAACCCAGGGAGCCAGACUGCCUGGGUUCAAAUCCAGAUCUAUAACUUGUGUGACUUUGGGCGAGUUACUUAAAGCCGGCUCUUUAACCAUCACACUAUUUUUUGCUUCCAGUG*

*>hsa-mir-611*

*GCUAGGCGCCUGCUCCUGCCGACGUGUUCUUCCGGUGGCGGAGCGGCGGAUUAGCCUUCGCGGGGCAAAAUGGUGAGAGCGUUGAGGGGAGUUCCAGACGGAGAUGCGAGGACCCCUCGGGGUCUGACCCACACCGCGCUUAUCUCCUCAGACGCCGGAGAUCCAGAGGGCUAGACGCUCCUGGAACCUCCGGCUUGGGC*

*>hsa-mir-612*

*CUGGCCUGACAUGUGUGUCCCUGAGCAGGUUACAGUCCUCUCUGAGCCUCUGCUUCCCAUCUGGACCCUGCUGGGCAGGGCUUCUGAGCUCCUUAGCACUAGCAGGAGGGGCUCCAGGGGCCCUCCCUCCAUGGCAGCCAGGACAGGACUCUCAAAUGAGGACAGCAGAGCUCGUGGGGGGCUCCCACGGACCCGCCGUG*

*>hsa-mir-613*

*UAUAAUAUAUUUUCUAUUUCUACUAGGUGUGGGCUUUAUUGGUUGAUUUCUCGGUGAGUGCGUUUCCAAGUGUGAAGGGACCCUUCCUGUAGUGUCUUAUAUACAAUACAGUAGGAAUGUUCCUUCUUUGCCACUCAUACACCUUUAUGUGCAUCUCACAUGGUGUUCUUCCCCAGGUGGAUGUGAUUCUCCAUGCCAGU*

*>hsa-mir-614*

*AAGUGCUGGGAUUACAGGCAUGAGCCACCGCGCCCGGCCCCGUUGUUUCCCUUCUAAGAAACGCAGUGGUCUCUGAAGCCUGCAGGGGCAGGCCAGCCCUGCACUGAACGCCUGUUCUUGCCAGGUGGCAGAAGGUUGCUGCUCAUUUGAGCAGUACCUGUCACCCCUCCUCCCACUGCUGCGGCUGUUAACUCAUCUUC*

*>hsa-mir-617*

*UAGCUAAAGAGUAGAGUUGAAGGGAUGGCCUGGAGCCAUGUCACGGAAGCUACCAUAAGCCAUCAUAAGGAGCCUAGACUUCCCAUUUGAAGGUGGCCAUUUCCUACCACCUUCAAAUGGUAAGUCCAGGCUCCUUCUGAUUCAAUAAAUGAGGAGCAUUCUGGAAAAGAGAUUUCAUCCAAGGCUUGAUCUCAAUUGAA*

*>hsa-mir-619*

*UGCCAUGUUGGCCAGGCUGGUCUCGAACCCCUGACCUCAAGUGAUCCGCCCACCUCAGCCUCCCAAAAUGCUGGGAUUACAGGCAUGAGCCACUGCGGUCGACCAUGACCUGGACAUGUUUGUGCCCAGUACUGUCAGUUUGCAGAGUGUCCCUCAGUGUGAGUGUGUCUGAUGUUCUUGUGUGAUUCCACCGGGCACGU*

*>hsa-mir-622*

*AAGAUCAUGGCAGACAUUCGGCCCAAUACGAUGAGCUGGCUGGGAAGAACCGAGAGAAGCUGGACAAGUACUGGUCUCAGCAGAUUGAGGAGAGCACCACAGUGGUCAUCACACAGUCUGCUGAGGUUGGAGCUGCUGAGAUGACACUCACGGAGCUGAGACAUACAGUCUAGUCCUUGGAGAUCGACUUGGACUCCAUG*

*>hsa-mir-636*

*GGAGGACCCCGCCUCGCGACUGGGGAAAUGGCGUCUGGCGGCGAGAUAAUGGCGGCCUGGGCGGGAGCGCGCGGGCGGGGCCGGCCCCGCUGCCUGGAAUUAACCCCGCUGUGCUUGCUCGUCCCGCCCGCAGCCCUAGGCGGCGUCGCCGCAGCCGAUCCCGGAGUCGGAGUCGUUCCAGGUCUCGCAGCCGAUCUCGC*

*>hsa-mir-642b*

*GAGGAACGGAAAAUUCCAGUAUGGGAGUGAUAGGCUGGGAGAGUUAAAUGAUGGCAGAGGCCGAGUUGGGAGGUUCCCUCUCCAAAUGUGUCUUGAUCCCCCACCCCAAGACACAUUUGGAGAGGGACCCUCCCAACUCAGAUAAACUGUGGAGAGUGUAGGAGAUAGGAAAUGGACUGAGAACUUCCUGGUCCCACCAG*

*>hsa-mir-645*

*GCUCAGGUGGUCAUGCUCAGUCGCUCGCCACUCACCUCCUGCCAUGCAGUCCAGUUCCUAACAGGCCUCAGACCAGUACCGGUCUGUGGCCUGGGGGUUGAGGACCCCUGCUCUAGGCUGGUACUGCUGAUGCUUAAAAAGAGAGGGUUUGCCAGAAAUCAGAUGGGACAAAAGGGCAAAGGCCGUGCCACAGAGUGCCC*

*>hsa-mir-646*

*ACCUGCCAGGACAGCACUGACUACCACAGCCAGGACAGUUCAGAAGCCUGGAGAUCAGGAGUCUGCCAGUGGAGUCAGCACACCUGCUUUUCACCUGUGAUCCCAGGAGAGGAAGCAGCUGCCUCUGAGGCCUCAGGCUCAGUGGCCUAUCUUUACCCCAAUGCAUGCAGGUCAGAAUGUAGCUGAACAGCAGCCCAGGG*

*>hsa-mir-651*

*GUACAAAUGUAUGAUACAGAUAACAAAAUGUAUUAACAAUUCCCUAGAUCUACAGAAAUCUAUCACUGCUUUUUAGGAUAAGCUUGACUUUUGUUCAAAUAAAAAUGCAAAAGGAAAGUGUAUCCUAAAAGGCAAUGACAGUUUAAUGUGUUUCUGACAUGCAACCUUCAAGAGAUCCAUAUAUGAACCAUUUUGUGUGG*

*>hsa-mir-655*

*UCUGGAAGCAAAGACAACGGUGGGGAAGAGGCCAUGGUUUUCGUUUCAGAACUAUGCAAGGAUAUUUGAGGAGAGGUUAUCCGUGUUAUGUUCGCUUCAUUCAUCAUGAAUAAUACAUGGUUAACCUCUUUUUGAAUAUCAGACUCUGCCUCGGAGGGCCGCCCCCACAGGAGACGUGAGAGUGUGUGCCUUGUGUGAGC*

*>hsa-mir-657*

*CACUCCAGGCCUCAGUUUCCCCAUCAAUUCCCACCUCCUGGGGAGCCGAGAGUGAUAGUGUAGUAGAGCUAGGAGGAGAGGGUCCUGGAGAAGCGUGGACCGGUCCGGGUGGGUUCCGGCAGGUUCUCACCCUCUCUAGGCCCCAUUCUCCUCUGCACUGUAACAUUUGAGGCCCACGCACACAGUCCCUCCCCAGGUCU*

*>hsa-mir-659*

*GUGCUGUCACCAGAGGUGACACAAAUUUGGAAAUAAUGGAGCUUAAGCUUUACCGACCCUCGAUUUGGUUCAGGACCUUCCCUGAACCAAGGAAGAGUCACAGUCUCUUCCUUGGUUCAGGGAGGGUCCCCAACAAUGUCCUCAUGGUCCUUUGCUUUUGUAAAAUUUGUGAAAUAUUUUUAUUGCUGAAACCACCUUUG*

*>hsa-mir-662*

*GCCAGUGGGGCUGGUGGGGCUGGCGUCCAGGCCGAGCUGGCCCAGGGUGGAGCUGUUGAGGCUGCGCAGCCAGGCCCUGACGGUGGGGUGGCUGCGGGCCUUCUGAAGGUCUCCCACGUUGUGGCCCAGCAGCGCAGUCACGUUGCCAACAUCCAGGCUCUGCGGAGAGGGGUGGGGCAGAGGGGCCGUGGGGCCAACUU*

*>hsa-mir-664*

*AAUUCCAAAGUGUUAAGUUCAGUUCAGGGUAGCUUCCCUGCUCUGUUAAUUAAACUUUGGAACAUUGAAACUGGCUAGGGAAAAUGAUUGGAUAGAAACUAUUAUUCUAUUCAUUUAUCCCCAGCCUACAAAAUGAAAAAAGGUACAAUAAACGUUAUUGUAUGUACAUCUUUAUGUAGAGGUCUGGUUAAUUUAUUCAA*

*>hsa-mir-765*

*GUCAUAACUCAGGAAGACAAGGCACAUACCUACCUGACCUAAGGGCUUUAGGCGCUGAUGAAAGUGGAGUUCAGUAGACAGCCCUUUUCAAGCCCUACGAGAAACUGGGGUUUCUGGAGGAGAAGGAAGGUGAUGAAGGAUCUGUUCUCGUGAGCCUGAACUUUCUAGACAAAACAUGUGGAAGCGGUCACCUCAGGCUG*

*>hsa-mir-767*

*UGGGCAUAUUCCUCAUUGUGUUAUUCACUCUUGUAUCACACCCACCUGCUUUUAUAUUGUAGGUUUUUGCUCAUGCACCAUGGUUGUCUGAGCAUGCAGCAUGCUUGUCUGCUCAUACCCCAUGGUUUCUGAGCAGGAACCUUCAUUGUCUACUGCUUUACAGGGAAAUAGUGUUUUAUGCAUCGUGUAUAUGAGUUUAG*

*>hsa-mir-891b*

*ACUAUAAUUACGUAGCUUCUUUGUUUCUUUGUAGGUCCCAAAAGAGUCUACAAAUGUUGUCUCCUUAAUCCUUGCAACUUACCUGAGUCAUUGAUUCAGUAAAACAUUCAAUGGCACAUGUUUGUUGUUAGGGUCAAAAGAGAUAAUCCAUCUAAAGGCCUCAUGGACAUUGCAAACCAUGUAAGUCAUCAGUUAUGUAG*

*>hsa-mir-922*

*UUCAGUUCUGGAGCUUCUGAGCCAGGCCUUUCUCAACCACCUCUCCUGCUGCUGAAACGGGGAUGGCGUUUUCCCUCUCCCUGUCCUGGACUGGGGUCAGACUGUGCCCCGAGGAGAAGCAGCAGAGAAUAGGACUACGUCAUGGGCAUUUCGUCCACUUAUUUGGGUAUUUUGGGGGCCACAGAACAAUCCUGACUAUC*

*>hsa-mir-924*

*GUUUCUGUUAUUUAUCCUAAGAUAGAUUGCUGGUGAAAAUUCCUCAACUGACCACCCACAGACCACCUAAGCCAGUCAAUAGAGUCUUGUGAUGUCUUGCUUAAGGGCCAUCCAACCUAGAGUCUACAACUGAUUCCCUUCCAUCCUAUGGUUUUUGCCUUUAUAAUAAUUUUCUACUCCUUGACUCCUCUUCGGUGUGU*

*>hsa-mir-933*

*GGCAGAGGAAAAAGGAAUAACGGCUCCCUCUUAGACCCCUCCCUCCGAAUUAUCUUCACCCUACUUGGGUCAGUUCAGAGGUCCUCGGGGCGCGCGUCGAGUCAGCCGUGUGCGCAGGGAGACCUCUCCCACCCACAGUCCUUGGGUUGGGCGGGGAGAGGGCCCACUCUGGUCUCCCCACCCAGUAUUCAAGCGAUUGA*

*>hsa-mir-934*

*AAGUAGGUAUCUGGGCCAGCCUUUGAUGGUGUGUGUCUGUAUCCUGAGAACGAACUUGAGAAAUAAGGCUUCUGUCUACUACUGGAGACACUGGUAGUAUAAAACCCAGAGUCUCCAGUAAUGGACGGGAGCCUUAUUUCUAUCACUCAGUGUUUUCAUAGAUAGAAUUUGUUUCAUUUUAGCCUCAAAAAAGAGUAUAU*

*>hsa-mir-936*

*CAGACAGGGCUGUCCCGUGGCUGAUGGCUGCGUGGGCUCCUGGGACACAAUGUGCUUCAAGGCCACUGGGACAGUAGAGGGAGGAAUCGCAGAAAUCACUCCAGGAGCAACUGAGAGACCUUGCUUCUACUUUACCAGGUCCUGCUGGCCCAGACGGACACCAAGGCCCAAGAGGUUGGUCACUCAUCUGCUGCCCCCAG*

*>hsa-mir-939*

*CCUGGAGGUGUACAGCGUGGACUUCAUGGUGGACAAUGCCCAGCUGGGUUUUCUGGGUAUGUGGGCAGGGCCCUGGGGAGCUGAGGCUCUGGGGGUGGCCGGGGCUGACCCUGGGCCUCUGCUCCCCAGUGUCUGACCGCGACCGCAACCUCAUGGUGUACAUGUACCUGCCCGAAGGUGAGUGCUUCCUCCACCCCCUC*

*>hsa-mir-93*

*AUGUGUCCUAGAUCCUGUGCUACAGACCUUCCUUUCUGUCCUCCCGUCUUGGACCUCAGUCCUGGGGGCUCCAAAGUGCUGUUCGUGCAGGUAGUGUGAUUACCCAACCUACUGCUGAGCUAGCACUUCCCGAGCCCCCGGGACACGUUCUCUCUGCCAAUUGUCUUCUUGGCUGAGCUCCCCAAGCUCCAUCUGUCAUG*

*>hsa-mir-96*

*UGCAGGGAUGCAAGGCCCCUCGUCCAGUGUGUCCCCAGAGAGCCCGCACCAGUGCCAUCUGCUUGGCCGAUUUUGGCACUAGCACAUUUUUGCUUGUGUCUCUCCGCUCUGAGCAAUCAUGUGCAGUGCCAAUAUGGGAAAAGCAGGACCCGCAGCUGCGUCCGCCUCCCCUGCAUCCUUGUGUCAGGGCCCCAGCCUGC*

*>hsa-mir-127*

*ACCCACAGGUUCCCAAGGCGCGGUGGAGGGACACUCGUAAAAGGUCUCGCUGUGAUCACUGUCUCCAGCCUGCUGAAGCUCAGAGGGCUCUGAUUCAGAAAGAUCAUCGGAUCCGUCUGAGCUUGGCUGGUCGGAAGUCUCAUCAUCUGCUUCCUUCGGGUUAAACACGUCGGCCAGGUCUGAGUAUGGGUGUGGCAGUC*

*>hsa-mir-155*

*AAAUCUGUGGUUUAAAUUCUUUAUGCCUCAUCCUCUGAGUGCUGAAGGCUUGCUGUAGGCUGUAUGCUGUUAAUGCUAAUCGUGAUAGGGGUUUUUGCCUCCAACUGACUCCUACAUAUUAGCAUUAACAGUGUAUGAUGCCUGUUACUAGCAUUCACAUGGAACAAAUUGCUGCCGUGGGAGGAUGACAAAGAAGCAUG*

*>hsa-mir-181c*

*UCUCUGGUUCCCUGCCACCUACCCCACCCCCGACUCCAGGUCCCGGAAAAUUUGCCAAGGGUUUGGGGGAACAUUCAACCUGUCGGUGAGUUUGGGCAGCUCAGGCAAACCAUCGACCGUUGAGUGGACCCUGAGGCCUGGAAUUGCCAUCCUCCUGCCGGUGACUCUGACCUUCCAGAUCUAGGGGGGCCUGGGGAGCC*

*>hsa-mir-184*

*AAUCAAACGUCCAUUUACAUCUUGUCCUGCAAAGCUUCAUCAAAACUUCUUUGCCGGCCAGUCACGUCCCCUUAUCACUUUUCCAGCCCAGCUUUGUGACUGUAAGUGUUGGACGGAGAACUGAUAAGGGUAGGUGAUUGACACUCACAGCCUCCGGAACCCCCGCGCCGCCUGCACUUGCGUGAUGGGGAAAACCUGGC*

*>hsa-mir-200c*

*GCAGCUUUUCCGCAGGGAUCCUGGGCCUGAAGCUGCCUGACCCAAGGUGGGCGGGCUGGGCGGGGGCCCUCGUCUUACCCAGCAGUGUUUGGGUGCGGUUGGGAGUCUCUAAUACUGCCGGGUAAUGAUGGAGGCCCCUGUCCCUGUGUCAGCAACAUCCAUCGCCUCAGGUCCCCAGCCCUUAGCUGGCUGCAGCCCCC*

*>hsa-mir-206*

*GAGGAAAGAUGCUACAAGUGGCCCACUUCUGAGAUGCGGGCUGCUUCUGGAUGACACUGCUUCCCGAGGCCACAUGCUUCUUUAUAUCCCCAUAUGGAUUACUUUGCUAUGGAAUGUAAGGAAGUGUGUGGUUUCGGCAAGUGCCUCCUCGCUGGCCCCAGGGUACCACCCGGAGCACAGGUUUGGUGACCUUCUUCCUC*

*>hsa-mir-433*

*UGUUCUGGCAGCUCCAUGACGUCAAAGUUGAAGUGGGAGAAGAAGAAGACCCAAUGCCCGGGGAGAAGUACGGUGAGCCUGUCAUUAUUCAGAGAGGCUAGAUCCUCUGUGUUGAGAAGGAUCAUGAUGGGCUCCUCGGUGUUCUCCAGGUAGCGGCACCACACCAUGAAGGCAGCCCGUAUUGGAAGAAUCUUCAUCUC*

*>hsa-mir-498*

*UCUGAUUUCAGAUGGACCCUGAGAAUGCUCAAGCCCCAAACCCUCCUUGGGAAGUGAAGCUCAGGCUGUGAUUUCAAGCCAGGGGGCGUUUUUCUAUAACUGGAUGAAAAGCACCUCCAGAGCUUGAAGCUCACAGUUUGAGAGCAAUCGUCUAAGGAAGUUGAUGGCAAUGUUAAUAGUUUUUUAAACAAGCAUGAAAU*

*>hsa-mir-554*

*CAACCAGUGGAGAGUCCCAGGGGUGAGAGGAAGGCCUACAGAUAGACCCAGGGUGAAGACCUGAGUAACCUUUGCUAGUCCUGACUCAGCCAGUACUGGUCUUAGACUGGUGAUGGGUCAGGGUUCAUAUUUUGGCAUCUCUCUCUGGGCAUCUUUCCUCUUCUUUUUUAGACCUCAUGACCAAGGCAGCUGGUGUCCCC*

*>hsa-mir-625*

*UGCAGGUUUUUAAGUUGUAAUCCUGUGUUGUACAAGAGCUGUACUGAUGUGGUGGUAAGGGUAGAGGGAUGAGGGGGAAAGUUCUAUAGUCCUGUAAUUAGAUCUCAGGACUAUAGAACUUUCCCCCUCAUCCCUCUGCCCUCUACCAACUGUGAGCCUGAGUUGAGUAACUGUCUCUUUUUCUCGGUCAAAGGAUAGAU*

*>hsa-mir-665*

*CCAGGGUGGAGGGGGUGGGGCUUUGGCGGACCAGGGCUGGCCCUUUCUGUGCCCCAAGAGGAGGGUCUCCUCGAGGGGUCUCUGCCUCUACCCAGGACUCUUUCAUGACCAGGAGGCUGAGGCCCCUCACAGGCGGCUUCUUACUCUCUCCUUAAACCUGUUCUGGAGACAUUUCCCCUCUCCCCAAGGAUUCCCAACUC*

*>hsa-mir-670*

*UCUUCCUCUCUGUCAAGUCCGAAGAUGCCAGCUGAGACGUUCUUCAUCCAGUUUAGGGGUGGACCUGAUGUCCCUGAGUGUAUGUGGUGAACCUGAAUUUGCCUUGGGUUUCCUCAUAUUCAUUCAGGAGUGUCAGUUGCCCCUUCACGCGACGUUGGAGAGCAUUACAGCAAGCAGGUGUCUACUCAUUUAUGGCAAGA*

*>hsa-mir-940*

*GCUAGUCCAUUUGGAGCUCCUUCAAGCCGAAGCAUCCAACACCAGUGUCUGUGAGGUGUGGGCCCGGCCCCAGGAGCGGGGCCUGGGCAGCCCCGUGUGUUGAGGAAGGAAGGCAGGGCCCCCGCUCCCCGGGCCUGACCCCACUGCUUCAGCCCCCUCCUGCCUGCCUACAGCCUGGCCUCGAGGGCCCAUCAGGUGAG*

*>hsa-mir-1224*

*CCUGACCCGGGCCAGGCCAACAACAGUGAAGGCAGCGACCAUGACUACCUGCCCUUGGUGAGGACUCGGGAGGUGGAGGGUGGUGCCGCCGGGGCCGGGCGCUGUUUCAGCUCGCUUCUCCCCCCACCUCCUCUCUCCUCAGGUGCGGCUGCAGGAGGCACCAGGCUCCUUCCGCCUGGACGCGCCCUUCUGCGCCGCUG*

*>hsa-mir-1238*

*CCCUCACUACUGCCAGCCCACUGUGGAUGUCAGCCAGGCCGACUUCCCCCUGGAGGUGAGUGGGAGCCCCAGUGUGUGGUUGGGGCCAUGGCGGGUGGGCAGCCCAGCCUCUGAGCCUUCCUCGUCUGUCUGCCCCAGUCCUUCCACUGCACCUCGCCCCGCAAGAUGGCCUUUGCCAAGAUGGACCCAAGCUGUACCGU*

*>hsa-mir-1273d*

*UAACCUGGUGGUGUGUUGGGCACCUGUAAUCCCAGCUACUCGGUAGGCUGAGGUAGGAGAAUCGCUUGAACCCAUGAGGUUGAGGCUGCAGUGAGCCAAGAUCGUGCCACUGCACUUCAGCCUGGGUGACAAGAGCGAAACUUCAUCUCAAAAAAAAAAGAGUUGGGAAAAGGUAACAUUCUAUAAUUCUUUCAUUUCUU*

*>hsa-mir-1277*

*AAAUAUAUGAUUGUCUUAUUUUACCCUAAUUUUUUCCAGCAGAAAUGCAGUAUCCAUUUCUAACCUCCCAAAUAUAUAUAUAUAUGUACGUAUGUGUAUAUAAAUGUAUACGUAGAUAUAUAUGUAUUUUUGGUGGGUUUAAUUUUUUUCAUCUUUUUUCAUAGUUUUUUUAGGGGCUUCAUUUAACUUCUGGUUAAGUC*

*>hsa-mir-1278*

*UUAUUGCUCUAUGCUAUGUGUUAUUCUAAGUACUUUAUAUGUAUUAACUCAUGUAAUCGUAUUUGCUCAUAGAUGAUAUGCAUAGUACUCCCAGAACUCAUUAAGUUGGUAGUACUGUGCAUAUCAUCUAUGAGCGAAUAGGCUCAGACAGAGUGAGUUCCUGAGGUUUGAAGAACCAGUUGGAACCUUGGCAGUCUGGU*

*>hsa-mir-1292*

*CCUGCGUUCGGGCCGCAGACAGGGCCUGGGCCUGGGCCUGGGCCUGCGCCUGCGCCUGCGCCUGCCCUGGGAACGGGUUCCGGCAGACGCUGAGGUUGCGUUGACGCUCGCGCCCCGGCUCCCGUUCCAGGUGCUGUUGCACGUGCUGUUUGAGCACGCGGUCGGCUACGCGCUGCUGGCGCUGAAGGAAGUGGAGGAGA*

*>hsa-mir-1306*

*GCCCGCAGGAGAAGCGGUGAUGGAGAGCCGAGCUCGCCCCUUCCAAGCGCUGCCCCGUGAGCAGUCUCCACCACCUCCCCUGCAAACGUCCAGUGGUGCAGAGGUAAUGGACGUUGGCUCUGGUGGUGAUGGACAGUCCGAACUCCCUGCUGAGGACCCCUUCAACUUCUACGGAGCUUCUCUUCUCUCCAAAGGAUCCU*

*>hsa-mir-3182*

*GGGGUUCUGGAGGAUAGCAGUGAGAAGAUGGGGUAAAAAAUUAGAGAGAAGUUUUGCGUUGCCCCAUGGCUGCUUCUGUAGUGUAGUCCGUGCAUCCGCCCUUCGAUGCUUGGGUUGGAUCAUAGAGCAGUGACUGUUGAUGGCUCACCCAGAUCCCCAGGAACCCACCACUCCCAUACAGGCUUUCAACCUGUUAAUCC*

*>hsa-mir-3652*

*GCCCGACCUGCUUGCGGUGUAGUGGGCGGACCGCGCGGCUGGAGGUGUGAGGAUCCGAACCCAGGGGUGGGGGGUGGAGGCGGCUCCUGCGAUCGAAGGGGACUUGAGACUCACCGGCCGCACGCCAUGAGGGCCCUGUGGGUGCUGGGCCUCUGCUGCGUCCUGCUGACCUUCGGUGAGUGAUUCUGGAGGAGCAGACG*

*>hsa-mir-3681*

*GUCCCCAGCCUGCCUGGCUCACAAGCCCUGCACACCAAGCCCAUCUUCAAGGAGACACUUGUCUACUUCCAGUAGUGGAUGAUGCACUCUGUGCAGGGCCAACUGUGCACACAGUGCUUCAUCCACUACUGGAAGUGUCAUGAGCUACUGUUCAGGAGUCCAGUGCAACCUGGAAUUCUGAGGGUGUUAACUGUCCAAGU*

*>hsa-mir-3923*

*AGAAUAUUACUGUGUUUGGGUAUCAGGUGUUGAAAGAGAUAAUUAGGCUAAUAUAAGUCUGGUAGAGUGAGCUCUAAUCCAAUAUUACUAGCUUCUUUAUAAGAAGAGGAAACUAGUAAUGUUGGAUUAGGGCUCACUCUACUAUUCUCAGCAAAAUAUCAGCAAAAUAUCACAAGGACAGGAAACCAAACAAUGCAUGU*

*>hsa-mir-4275*

*CUUCAGGAGGAAGCUGCUUUCACUACUUUUUUUUUUUUAAUUACCAUGAGGGUUAGAACAUUUUUGUCCAAUUACCACUUCUUUUUGCCACCUGAGCACAGUCAGCAGUCAGCAUAAAAAAGUGAUAAUGGGAAGUUAAUGUCUAACCAGGUCACAUUUCGCUUCAAAGGAACAAAACACAUACAAAAUGCAUGUUUUUU*

*>hsa-mir-4314*

*GCGGGGGAGGGUAGGGGGAGGGGCACUAAGUGGCAGCGCAGCUGGCCAGCCUCCAGGCCAUUCCUCUCUGGGAAAUGGGACAGGUAGUGGCCACAGUGAGAAAGCUGGCCUGUCCUUCUGCCCCAGGGCCCAGAGUCUGUGACUGGAAGGGUGGGAGGUGUUGAAGCUAGGGCUCGGGGACCACAUGGGUGCCCAAAGGC*

*>hsa-mir-4326*

*GAGCUGGGCUCCCCAGGCUAGGGUGGGAGGGAGGGUCUCACCCAUAUGCCCCCACGUAGGAGGAGCUGGCGCUGCUCUGCUGUUCCUCUGUCUCCCAGACUCUGGGUGGAUGGAGCAGGUCGGGGGCCAGGGGACAGGAAGGCUAGGGCCCCAGAGACCUGUCCUGGGCCCCAUGUCCAGCUCUGCCCUUAGUGCUUGGC*

*>hsa-mir-23a*

*UCUCUUUCUCCCCUCCAGGUGCCAGCCUCUGGCCCCGCCCGGUGCCCCCCUCACCCCUGUGCCACGGCCGGCUGGGGUUCCUGGGGAUGGGAUUUGCUUCCUGUCACAAAUCACAUUGCCAGGGAUUUCCAACCGACCCUGAGCUCUGCCACCGAGGAUGCUGCCCGGGGACGGGGUGGCAGAGAGGCCCCGAAGCCUGU*

*>hsa-mir-25*

*AGCCCAGUGGCGUUCAAAAGGGUCUGGUCUCCCUCACAGGACAGCUGAACUCCGGGACUGGCCAGUGUUGAGAGGCGGAGACUUGGGCAAUUGCUGGACGCUGCCCUGGGCAUUGCACUUGUCUCGGUCUGACAGUGCCGGCCCAACACUGCGGAUGCUGGGGGGAGGGGGGAUUCCACUCCUGUUUUGUGAGUAGGCGA*

*>hsa-mir-31*

*GAAAGGAAAAAUUUUGGAAAAGUAAAACACUGAAGAGUCAUAGUAUUCUCCUGUAACUUGGAACUGGAGAGGAGGCAAGAUGCUGGCAUAGCUGUUGAACUGGGAACCUGCUAUGCCAACAUAUUGCCAUCUUUCCUGUCUGACAGCAGCCAUGGCCACCUGCAUGCCAGUCCUUCGUGUAUUGCUGUGUAUGUGCGCCC*

*>hsa-mir-195*

*ACCCUGGGAGUAAGUUCUGCCUCAAGAGAACAAAGUGGAGUCUUUGUUGCCCACACCCAGCUUCCCUGGCUCUAGCAGCACAGAAAUAUUGGCACAGGGAAGCGAGUCUGCCAAUAUUGGCUGUGCUGCUCCAGGCAGGGUGGUGAAAACUACCGAGGAGGGGCUGAGCCCCCAUGGGCCGAGGAGAGAAGAGGGAACAG*

*>hsa-mir-339*

*GGCAGGAAGCGUCCCAUGCUCUGCAGGGGUGGCAGGAAGCGUCCUGUGCUCCGCAGGGGCGGGGCGGCCGCUCUCCCUGUCCUCCAGGAGCUCACGUGUGCCUGCCUGUGAGCGCCUCGACGACAGAGCCGGCGCCUGCCCCAGUGUCUGCGCCGGGACCAUGGAGUGGCCAGCUCGCCGCGGAGACGUCGCCGCGCUUU*

*>hsa-mir-600*

*GCCUGUCCCUGGCUGCAGUGCCAGGUGUGGCCUUUCCUGUGACAGCAAGUCACGUGCUGUGGCUCCAGCUUCAUAGGAAGGCUCUUGUCUGUCAGGCAGUGGAGUUACUUACAGACAAGAGCCUUGCUCAGGCCAGCCCUGCCCCUGCCUACAGGCACAGGUGCUUUGCCAUCUGUCCCGUUUUCCUGCUUAGGGAACCA*

*>hsa-mir-631*

*UUUGCCCCCUGUGGGACAGAGGAACAGGCAGAGAUCAGAGGGCAGGCUCAGGUUGGGAGGAGUGGGGAGCCUGGUUAGACCUGGCCCAGACCUCAGCUACACAAGCUGAUGGACUGAGUCAGGGGCCACACUCUCCCUCCUCUGGUGAUGUGACCUCAGCUGGUUUCUUCCCACUCGGCCAUGGGUUUCCCAUCCUGGAG*

*>hsa-mir-649*

*UCCUCUCACCUCAGCCUCCCAAAGUGAUGGGAUUACCGGUGUGAGCCACUGGGCCUAGCCAAAUACUGUAUUUUUGAUCGACAUUUGGUUGAAAAAUAUCUAUGUAUUAGUAAACCUGUGUUGUUCAAGAGUCCACUGUGUUUUGCUGGGGAUUUCUACAUCUAUAUUCAUAAGGGAUUUUGGUCUGUGGUUUUCUUUUC*

*>hsa-mir-653*

*CCAGACCAGGAACUGAUGCAGAUUAUUACUGCUCAUCUGACAACAGAGGUUCUAAUUUUUCAUUCCUUCAGUGUUGAAACAAUCUCUACUGAACCAGCUUCAAACAAGUUCACUGGAGUUUGUUUCAAUAUUGCAAGAAUGAUAAGAUGGAAGCUACUUUCAUCAGCAUGAAUUAUAACUACUCGUAUACAGAUUGUCGU*

*>hsa-mir-661*

*UCUCCUGCCGUGGCCCAGGAGAGGCGUCUCCAUCCCAGAUCCCUGGGACCCCAGGAGAGGCUGUGCUGUGGGGCAGGCGCAGGCCUGAGCCCUGGUUUCGGGCUGCCUGGGUCUCUGGCCUGCGCGUGACUUUGGGGUGGCUGUCCCGGCCACCGGUGGGGCCAGGUGCUGUGUUGAGUGACCUGCGCAGGGCCCUCUGG*

*>hsa-mir-1250*

*GCCCCUGGUAUGUCAUGACGCAGAAUGGGCCAAGGAGCUGGCUCUGCCCACCUGUCCCGCUGGCCUGGCAGGUGACGGUGCUGGAUGUGGCCUUUUUGCCUUUUCUAAAGGCCACAUUUUCCAGCCCAUUCAACCUUCCAGAGCCCUCUGAAGUGGCCACAGGCCCCAUCAACCAGUGAGGAGCUGCGUGCCUGGGGGGC*

*>hsa-mir-1253*

*AGUGAGGGGAUGGCCUGGCUACUGGGCUACUGGAAGAGUUUGUCAUCAGAGCAGCAAGAGAUAGAAUCCAAAAGAGAAGAAGAUCAGCCUGCAGAUGUGGACUGCUAAAUGCAGGCUGAUCUUCUCCCCUUUGGGAUUCUCUUAUGAGAAGCCAUAAACGCCAGGACGAGCACCCAGUGGGGUGCUUCUAGUUGCCAGGC*

*>hsa-mir-3202-2*

*UGACUGUCAACUGCUUUGGGGCCACUGGUGCUGUAAGUGAGCCCUAGUAGCUGAGAAAUACAUUAAUAUGGAAGGGAGAAGAGCUUUAAUGCUCUGAAAAUGACUCCAAUCAUUAAAGCUCUUCUCCCUUCCAUAUUAAUAAAUUUCGCACUCAAGAGGGUGGGCGGGUAGGCGGGCUCAGGGAGAGAGAAGGGUGGGCU*

*>hsa-mir-3667*

*CCUUAAAAGGCCCCUACUCCUGCUGUCUUAGAUGCUUCCAUAAAGAAGUCUUAUCUAGCUUCUCUGAGGAUGAAAGACCCAUUGAGGAGAAGGUUCUGCUGGCUGAGAACCUUCCUCUCCAUGGGUCUUUCAUCCUCAAAGAACAGUUCCCAGCCAACCUGCCAGCUAAACACAGCCACAUACCUGAGCCCAGGCAAGAA*

*>hsa-mir-3689a*

*GUGAUCCUGUUCUUCCUGAGAGGUGUGAUCCUGUGCUCCCUGGGGGGUCUGAUCCUGUGCUCCCUGGGAGGUGUGAUAUCAUGGUUCCUGGGAGGUGUGAUCCUGUGCUUCCUGGGAGGUGUGAUAUCGUGGUUCCUGGGAGGUGUGAUCCCGUGCUUCCUGGGAGGUGUGAUACUGUGCUUCCUGGGAGGUGUGAUCCC*

*>hsa-mir-3689b*

*GUGAUCCUGUUCUUCCUGAGCGGUGUGAUCCUGUGCUCCCUGGGGGGUCUGAUCCUGUGCUUCCUGGGAGGUGUGAUAUCAUGGUUCCUGGGAGGUGUGAUCCCGUGCUUCCUGGGAGGUGUGAUAUUGUGGUUCCUGGGAGGUGUGAUCCCGUGCUCCCUGGGAGGUGUGAUCCCGUGCUCCCUGGGAGGUGUGAUCCC*

*>hsa-mir-3927*

*GUUGUUCAUCUCAGGCUUCUUAGGGCAUGGUGGUUUCAGGUUUCCAAGAGGGCCAACCCCAAUAUGCCAAUGCCUAUCACAUAUCUGCCUGUCCUAUGACAAACAUGGCAGGUAGAUAUUUGAUAGGCAUUGGCACACUGGCAAAACGAAGUCCUAUGGCCAUGCUCAGCCUCAGCAUGAAAGGGGAAUUCACAGGAGUG*

*>hsa-mir-3940*

*UUCAAGUUGUUCAUCAUCCGGGGUUCACCCCAGCAGAUUGACCACGCCAAGCAGCUUAUCGAGGAAAAGAUCGAGGUGGGUUGGGGCGGGCUCUGGGGAUUUGGUCUCACAGCCCGGAUCCCAGCCCACUUACCUUGGUUACUCUCCUUCCUUCUAGGGUCCUCUCUGCCCAGUUGGACCAGGCCCAGGUGGCCCAGGCC*

*>hsa-mir-4252*

*AGUCACAUGCUGCUGUGGGGAACGGAGCCCAAGCCCUCUGUCCACCUCCCUGAGAUUCAUGGUGACUCCUGGGGGGCUGGCAGCUCAUCAGUCCAGGCCAUCUGGCCACUGAGUCAGCACCAGCGCCCAAUCACACACAGCACCUGGCAUGGCCUGGGAGGGGGUCAGGGUCCCCCAGCCCCGGAGCCCUGGAGGGCGUU*

*>hsa-mir-4279*

*AGUCCUGGGUUAUUAACUAUCAACAAAAAUACAAGCUGCGCCAUGCGUGCUCCAACUUCCUGCCAGCUCCCCAUGCUCUGUGGAGCUGAGGAGCAGAUUCUCUCUCUCUCCUCCCGGCUUCACCUCCUGAGCUCCAGAGCCCCACCCACCCACAGCACUCAGAUCAUUUUUCAUUUAUGAAUGCAUCCAGCCACGCUGUG*

*>hsa-mir-4296*

*CGGUGGGUGCUUUGCCUAGCUUAGUGCCUACCAUGCCCAGGCUUGCAAAUGGGGCUGUUUGGGCUUUGAUGUGGGCUCAGGCUCAGAGGGCUGAAGUGGUUGUGGGGAGGGGCUUCUGGGGACUGUGUCCAUGUCUCUGUCGUUUUUCCCCAUCACACCGUACACUCCCUUGAGGCCAGCCGUCCAUCACCAUUGAGUCA*

*>hsa-mir-4328*

*UGGCACCUCUUCCCAAAAGUUGAAGCAAUAAUUUGGCCUCUCUGGUAUUUAGAAUUUCCUGAAUUAACCCCAAAACAGUUGAGUCCUGAGAACCAUUGAGAACCAGUUUUCCCAGGAUUAACUGUUCCGAGAGAUAAAAACUAUUUUAGUAAGAACUUAAUUUCCAAAGGCAAAUUUCCCUGCUCCCUGUUCAUCCCUCA*

*>hsa-mir-4445:3:109321609:109321810*

*AGAUGAAUAGCUGGGGACUUAAUUAAACUAAAUUAAGCACAGCAAAAUUUUGUGAUUGUCUGUUUUUCCUGCAGAUUGUUUCUUUUGCCGUGCAAGUUUAAGUUUUUGCACGGCAAAAGAAACAAUCCAGAGGGUAAACAGACAACCCACAAAAUGGGAGAAAAUCUUUGCGAUCUAUACAUCCGACAAAGAGCUAACAU*

*>hsa-mir-4739*

*AGGCCUGGACCAGCUGGGAUGUUCCCAUCGGCGUGUCCCUCCCGCCCAGGCCACGUGGGCAGGGGAGGAAGAAGGGAGGAGGAGCGGAGGGGCCCUUGUCUUCCCAGAGCCUCUCCCUUCCUCCCCUCCCCCUCCCUCUGCUCAUCUCAUCAUGAGACAGCUGGCAAGGGCCUUCGGCUGCCCUGGAGUGCCCCUGCCCU*

*>hsa-mir-5584:1:45011092:45011293*

*ACUCAGCAAGGCAGUUCACGUUAUUCCCACCUUGAGAUUCAGAGGAGGAAGUGACUAAUCCAAGAUCCAUAACAGGGAAAUGGGAAGAACUAGAUUUGAAUCCAGACCUUUAGUUCUUCCCUUUGCCCAAUUAUGCUCCUUGUGGUUUGCUGGAGAAAGCCUGGCAGAGGGUGCAAGGGCAACAGUGGGGGUGGGGGUGC*

*>hsa-mir-5680:8:103137601:103137802*

*AAAACAAAAAUUAUCUAGGAGGCUGUUAAACCAUCAUCAAAUACUAAAACCUUUGCUUGCAUUGGGUUAGCAGGUUAGCCCAGCAUUUCCCUUCCUGGACACACAGGAGGAGAAAUGCUGGACUAAUCUGCUAAUCCAAUGCAAGCAAAAAUUUGCAUUGGAAAGUUGGAUUUUUUGGUUUUUGGUUUUUUCUUUUUUCU*

*>hsa-mir-378i*

*CUGACCUGGACUUCGCCCUGGGCAGCUUCCUUGGAACUUGUUGCAGCUUAGCAUCUUUGGAAGGGAGCACUGGACUAGGAGUCAGAAGGUGGAGUUCUGGGUGCUGUUUUCCCACUCUUGGGCCCUGGGCAUGUUCUGUUCUGUGCAUCAGAUGUUUUCUGCCUGCCUCCCCCUGAGCUGCCACCAGAGCCACCUGUAGA*

*>hsa-mir-4679-1:10:90823030:90823231*

*UUUCUCUCCAUUAUGACUUAUAUAAAAGAAACAAAAGCAGAAACAAAAACUUUUGCUUUACCGUCUUUUUUCUGUGAUAGAGAUUCUUUGCUUUGUUAGAAACAAAAAGCAAAGAAUCUCUAUCACAGAAAAAAGAUAGUAAUAAGCACAGAUGCCCAAAGAAGCCAGGCAGACUUUUUUUUUAAAGGUUUAAGUUUUAU*

*>hsa-mir-4472-1:8:143257639:143257840*

*AAGCUCCUGAAUACUAAUUGAAAAGUGAUUAAUUACAUUCACGGAGCCCCAUCUCCUCACUGGCAGACCCUUGCUCUCUCACUCUCCCUAAUGGGGCUGAAGACAGCUCAGGGGCAGGGUGGGGGGUGUUGUUUUUGUUUUUUUAAGGCUGAGUUGGUUUAAAUCUCAUUUAAUUUUCACAAGGGAGAGCCUUGAGCCUC*

*>hsa-mir-378a:5:149112321:149112522*

*GAAAGAGGCUGCGAGGAGUGAGCGGCUUGUAUGGGACCAUGCAGCCAGAGGGUGACAGAGCCACCCAGGGCUCCUGACUCCAGGUCCUGUGUGUUACCUAGAAAUAGCACUGGACUUGGAGUCAGAAGGCCUGAGUGGAGUCACCUUCCCCACUCUCUGGCUGGGUGACCCCGGAGCAAGCCAUUUGAACUCUCCGAGCC*

*>hsa-mir-4726:17:36875871:36876072*

*GCAGGAGGGACACCCGAGGGAGGAGGGACGGAGAGACCGGCGUGGGCUGGAACCCCUGGGGGAUACGGGAGAAGGGCCAGAGGAGCCUGGAGUGGUCGGGUCGACUGAACCCAGGUUCCCUCUGGCCGCAGGUCCCCCAUCAGCAGCCUCCCCGCACUCUUCGACCAGACAGCCUCUGCACCCUGUGGGGGCGGCCAGUU*

*>hsa-mir-4648:7:2566635:2566836*

*UCUCUAAGCGGCAUGACUCUACAUAGAGGUGUCCCCCGGAGUCCUGCUUGCUCGGGGUGGGGCCGCCAGUGUUGUGGGACUGCAAAUGGGAGCUCAGCACCUGCCUGCCACCCACGCAGACCAGCCCCUGCUCUGUUCCCACAGGUUCCGCUCCAUCCACUGCCACCUGUACCCGGACACACCCUGGUGUCCCCGCACUG*

*>hsa-mir-4438:2:214622737:214622938*

*GGGCAUUCAAAGUAACUGCACAUAUGGUGACAAUCAGAAAAUGACUAAGUUACUAAGUGUAAACUUAAGGACUGUCUUUUCUAAGCCUGUGCCUUGCCUUUCCUUUGGCACAGGCUUAGAAAAGACAGUCUUUAAGUUUACACUUCAGACUGAUUCUUGGCACAGAGAUAGCCUACAACAGAAUCCUCUUAUGACAAUAU*

*>hsa-mir-4524a*

*AGGGAAUUCUCCAGAAAGUGGAACCAGAGGAGUAGCAGAAAGAGACAGUGAUUACCCUUCCUCUUUGGAACGAUAGCAGCAUGAACCUGUCUCACUGCAGAAUUAUUUUGAGACAGGCUUAUGCUGCUAUCCUUCAAAAGAACACAUCCACCCAGCUACUGAGGUUUCCAUCUCUGGGUUUGCUCAGAAAGACAUGCUGC*

*>hsa-mir-4743:18:46196903:46197104*

*UGGGACAGCCCCUGCAGGGCUUGAGGGUGGGCAGCACCAGGUAUCUGCUGACUGUCCCUCCUCUCCUGCUGGCCGGAUGGGACAGGAGGCAUGAAUGAGCCAUCUUUCCAAUGCCUUUCUGUCUUUUCUGGUCCAGGUGUGGCAAAGGGAAGCUGGAAGAUGGGGAUGGCAUCAACCUGAAUGACAUCGAGAAGGUCCUU*

*>hsa-mir-4687:11:3877232:3877433*

*GAAGUCUCCGGAAGCGGCACGAGCUCAGGCCGCCGCAGCCCCGGCGGACCCACUGUUGGACCUGAGGAGCCAGCCCUCCUCCCGCACCCAAACUUGGAGCACUUGACCUUUGGCUGUUGGAGGGGGCAGGCUCGCGGGUGGCUGGACAGCUGCGGAGCCGCGAGGGCAUCUUGCCUGGAGACCGUCGGCUGCACUCCCGG*

*>hsa-mir-4776-2*

*CUCUUGCACUGCUGUGUUUUUGCAUCCCCUUCUACCUUCUGGCUUCCUGAGAUGGAGCAGACUAUAUGCAGUGGACCAGGAUGGCAAGGGCUCUACUGUCCUUUCAGGAGAGCCCUUGCCAUCCUGGUCCACUGCAUAUAGCCGAGCUCCCACUGACCUGGAACCCUCAUCCUGGACUGUUGUCCAAAAGAGAGAUAGAU*

*>hsa-mir-4436a:2:89111827:89112028*

*GACAUGAAUUUCUCCCUUGUUUCAGCCACCGGCAUCCCAGGCCAGGACUCCUCAUGGCCUCACUUUUCCACUUAUGCCUGCCCUGCCCCUCGAAUCUGCUCCACGAUUUGGGCAGGACAGGCAGAAGUGGAUAAGUGAGGACACUUCUCACUCAUGGGGAGAACACGACUGUGUUGCCCCAUGUUGAAGCUGUGUGAUGG*

*>hsa-mir-4660:8:8905892:8906093*

*UCCUGGCACAUUACUCAAUUGAUAGUUUAAAAAACGUCCUUAAGAAGAAGAAAGACAGCCAAACUCCUUCUGCAGCUCUGGUGGAAAAUGGAGAAGACUUUUCCUUUCCUCCAUCUCCCCCAGGGCCUGGUGGAGUGAGGCGUUGCCCAGCUAUAAACUGUGGUCACUCUUCUGGUGGGGCCAGCAGAUAACAGCUGAUG*

*>hsa-mir-4420*

*CAUGGAAGGGCCCUGGCCCGGGAGUCUGGAGACCUUGGUUCUAGUCUUGGGUCUGCCACCAGCUCUUGGUAUGAACAUCUGUGUGUUCAUGUCUCUCUGUGCACAGGGGACGAGAGUCACUGAUGUCUGUAGCUGAGACGGUCUCUCAAGUGAGGGCCUACUAUGUGCCGCUCAGUAACUGGGACUUUAAUUGCUGCUCC*

*>hsa-mir-5682:3:120768424:120768625*

*GCACAGAGCUGCUCCUGAUCCGAGAAUUCUGGUAGGGCUGUGGUGGUCUCACUAGUGUCCCAGGCCCAUGGGUCUUAUCCUGCAAGGUGCUGCAGAGACGAGGCCUGUAGCACCUUGCAGGAUAAGGUCUACUGGGCCUAGUUACUCUUCAGCCACAUGGAUUCAGCCCCUUUCCUGGAGACGUGAGGGAGCCUGGCCUC*

*>hsa-mir-3135a:3:20178995:20179196*

*AUCUUUGCAGUUCCUUCCUUUCUCCGUUCCUCCCUUCCUUUUUUUUUUUUGAGACAGGGUCUCACUUUGGUGCCUAGGCUGAGACUGCAGUGGUGCAAUCUCAGUUCACUGCAGCCUUGACCUCCUGGGCUCAGGUGAUUCUCCCACCUCAGCCUCCCAAGUAGCUGUGACUACACCCAGCUAAUUUUUUGUAUUUUUAG*

*>hsa-mir-548al:11:74110226:74110427*

*CAAACUAGGGUUCCAAUCUCAUUUCAGAUCCCCUCAGCUGGCAGUUAUUUUAUUAGGUCGGUGCAAAAGUAAUUGCUGUUUUUGCCAUUAAAAAUAAUGGCAUUAAAAGUAAUGGCAAAAACGGCAAUGACUUUUGUACCAAUCUAAUAUCUCUGCUGCUAUAGUAAACUAGGAGGUAGGGAGAACAAGAUUACUGGAAA*

*>hsa-mir-4646*

*AGGCAUCUUCUUCCUCUUAGGGUAUGGCUGUUCCAAACAGGGAGUGUGGGGUGGAGGUUGGGGCUGAACUGGGAAGAGGAGCUGAGGGACAUUGCGGAGAGGGUCUCACAUUGUCCCUCUCCCUUCCCAGGUUACUUGAGUUUGUCCAAAGUGGUGCCGUUUUCUCACUAUGCUGGGACAUUGCUGCUACUUCUGGCAGG*

*>hsa-mir-4450:4:77494653:77494854*

*GAAAUAAAAUCCUCCCUUGCUCAGAUGUCAUCUUUUUUCUCCACUGCCCAGCUGUCUUGCUAAACAGUGUCUGGGGAUUUGGAGAAGUGGUGAGCGCAGGUCUUUGGCACCAUCUCCCCUGGUCCCUUGGCUCUGUCCAUAGCCUUCCCAGCCACUGCUGGCCUCCAACGCUGAAUCCUGCAGCUGGUAAAUCCAUGGUC*

*>hsa-mir-4784*

*CGCGGAACUCCCAGGGUGCAGAGGCCUGCGGGGUCUGAAGGGCCUCCUCCCUCUCCAGUGUGGUGACUGGGCUGAGGAGAUGCUGGGACUGAGAGUGUCAUGGUGGAGCCUCCGUCCCUGCUCAUCCUCUCCGCAUGUUGCUUCUGCUCCCGAUGGCUCUCUCUGAAAUGCAGCACAACCUCCCAGGCCAAUGGAAGGAG*

*>hsa-mir-518a-2:19:54242530:54242731*

*UUUGAACUGGUUAAGGAAAAUUUCAACAAGAAACCCAGAGUGCCGGAGCAAGAAGAUCUCAAGCUGUGGGUCUGCAAAGGGAAGCCCUUUCUGUUGUCUAAAAGAAGAGAAAGCGCUUCCCUUUGCUGGAUUACGGUUUGAGAAAGCGACGUUGAAGUUGAUGCAUUUGCAGAGCAUGCUUAUCAUCAGGCUUGGACAAU*

*>hsa-mir-4683*

*GUGCCGCUGUGUAAGGGCAUCGGCUACAACUACACCUACAUGCCCAAUCAGUUCAACCACGACACGCAAGACGAGGCGGGCCUGGAGGUGCACCAGUUCUGGCCGCUGGUGGAGAUCCAGUGCUCGCCCGAUCUCAAGUUCUUCCUGUGCAGCAUGUACACGCCCAUCUGCCUAGAGGACUACAAGAAGCCGCUGCCGCC*

*>hsa-mir-4526:18:13611055:13611256*

*CUGCCCCCAAAAAUGAGAACAUGAGAAAGUGAUGUCUUCUAGCCACCUGGAGGACCCUGCGGUGACAUCAGGGCCCAGUCCCUGCUGUCAUGCCCCAGGUGACGUGCUGGGCUGACAGCAGGGCUGGCCGCUAACGUCACUGUCCUGGCUUCUAACGCUGGUAGUAACCAACAGAGUUUAGUUUUGCUUUGACAACUCCU*

*>hsa-mir-4506*

*CCCAGGCCCUCUUACAUGGAGUGAGGGUUAAGAGCAUGGCCUCUACCAUCAGAAUAUCCACCAUGGCCUCUGCCAUCAGACCAUCUGGGUUCAAGUUUGGCUCCAUCUUUAUGAAAUGGGUGGUCUGAGGCAAGUGGUCUAGGUGUUCUGUGCUUCACUGUCCCCAUCUGUAAUCUGGAGAUAAGAGCAGUACUGACCUC*

*>hsa-mir-151a*

*UGUUGAGCACCUACAGUAGCUGAGCCUGGUGCUAGUCACUGGGGCAAAGAUGACUAAAACACUUUUCCUGCCCUCGAGGAGCUCACAGUCUAGUAUGUCUCAUCCCCUACUAGACUGAAGCUCCUUGAGGACAGGGAUGGUCAUACUCACCUCGGUGUUGCCCAAACCUCCAGGCCAGUAGCCUAGCUAGCUAGAUACAG*

*>hsa-mir-3591*

*CAGAUGAACCUUCUUGCUCAAAGCAAACGAUGCCAAGACAUUUAUCGAGGGAAGGAUUGCCUAGCAGUAGCUAUUUAGUGUGAUAAUGGCGUUUGAUAGUUUAGACACAAACACCAUUGUCACACUCCACAGCUCUGCUAAGGAAACUCUGUAGCCACGAAGGUGUUAACUUCACCUCCACAUUCCACCAUUGUCACUGA*

*>hsa-mir-4455*

*GUAAUCAUAUAAAUGCUCAACAAAGAGCUAACAAAAAUUAUGAUAUAACCAGAUUAGAAAGAUAAGCAGAUGAGAAGGGUGUGUGUGUUUUUCCUGAGAAUAAGAGAAGGAAGGACAGCCAAAUUCUUCAUUUGCAAAGGGAUAAGCCAAUAGAUGAAUGCAUAAAGCUGAAAAAUCAAGGAAAGCAAUAUAAGCAUUUU*

*>hsa-mir-4705*

*AGAAUAGAUCUUGACAUUGACAAAAGUCCUUAAGAACAUGCCAAGGAUUUACCACUUCUCUAACCCUCACAAGAUCAAUCACUUGGUAAUUGCUGUGAUAACAACUCAGCAAUUACCAAGUGAUUGGUUUUGUGAGAAUGCUUGUUCAGCUCUUCUAGCCAUUCCUCACAGGCUUGUCAGACCCCGGGCCCUUCUGCAUG*

*>hsa-mir-4718:16:12814102:12814303*

*CCUUUUUCAGCCUUGGAGAAAGAAGCCUUUGCCCUAAAAAUACAAAAGUGUCAGCUUCUCUUCUGUGUUCUUUGUAGCUGUACCUGAAACCAAGCACCUGUUUGUGACUUGGCUUCAGUUACUAGCAAGCUUUUCCUAAAGAGGUGUUCAAAUCCCGCACACCAAAGGAUUUGUGCUUCAGUGAAGGAAACUUCUUGUUU*

*>hsa-mir-4442*

*GAGAGGCUGUGGCGACAAGGCCCGGAUUGGACAGCAUGGCGCUGACUGACAGCGGGGGCGGCCGCCGCGCCCUCCCUCUCUCCCCGGUGUGCAAAUGUGUGUGUGCGGUGUUAUGCCGGACAAGAGGGAGGUGACCGUGGCGGCGGCGGCGGCGGCUCUGUUUAUUGUCCCUCUCGGUGUGUGUGUGUGAGGAAAUCGGG*

*>hsa-mir-4466*

*CGCCCCAAACGGCGACCGCGCGGGUGGCGCCUCGGGAUCUCGCUUCGCGUUCCCGGGCCUGGGGGAGGGGGGGGACGCGGGUGCGGGCCGGCGGGGUAGAAGCCACCCGGCCCGGCCCGGCCCGGCGAAGCCCUCCCAGGCCUGGACAGCGCGCCGGCCCCCGGGGCGCCCCGACCGCGGCGGGAGGCGGCCGCCCGUCA*

*>hsa-mir-5590:2:135615316:135615517*

*UGUUCCUGAUCUUAGAGAAAAAGUUUUCAACUUUUCAUCAUUUUCACCAUCAAGUAUGAUGUUAGCUGUGGGAUUGCCAUACAUAGACUUUAUUGUGUUGAUCAACAAUAAAGUUCAUGUAUGGCAAUCCCACAGCUUUAUUCCUUCUAUACCUAAUUUAUUGAGGUUUUUUAAAGUCAUAAAAGGAUGUAGAAAUUUGU*

*>hsa-mir-5188:12:125400044:125400245*

*UGUACAUUCUGAGGGCCAGGUAUGGAAAGGUCCUACUUGAUGCCAACAGGGAGGCAUGGAAAUUUCUCUGGUUUCAAUGGGUACGAUUAUUGUAAGCAGGAUCCAUUCAAUAAUCGGACCCAUUUAAACCGGAGAUUUUAAAAGACAGGAAUAGAAUCCCAACCUUAUUUCAGUGCAGGGUAGGGUGUCAAAUAUGAAAU*

*>hsa-mir-4666a:1:228649714:228649915*

*AAACUAAGCCAAAUUUUUAAACUAAGCUUCAUCUCACCUAUCUGAGAUGGGUGAGUGACAAUCACUUAAAUACAUGUCAGAUUGUAUGCCUACAAAAUCCCUCCAGACUGGCAUACAAUCUGACAUGUAUUUAAGAGAUUGCCCCUCACCCAUCUCAGAUGGAAAUUUCAAGUCAGAUUUGCUAAUUAUUAUGCAGUAAA*

*>hsa-mir-3972:1:17604326:17604527*

*ACCCAGGCUUGCUCAGGUACCCAGCACAUACCCAGAGGCCACAAUACUCUGGAGGUCGCCCAUUUGCCUUGGCUUGGGGUGGCAGUCCUGUGGGAAUGAGAGAUGCCAAACUGGACCUGCCAGCCCCGUUCCAGGGCACAGCAUGUGGGUCUCAUAGCAGAGGCAUGCCCAAGCACAGCCCCCUGGGAGCUAGGCCUCCG*

*>hsa-mir-644b:X:153996801:153997002*

*UUUCCAAAGUGUUGAGUUCAGUCCAGGGCAGCUUCCCUGUUCUGUUAAUUAAACUUUGGGACAUUAAAAUGGGCUAAGGGAGAUGAUUGGGUAGAAAGUAUUAUUCUAUUCAUUUGCCUCCCAGCCUACAAAAAUGCCUGCUUGGGGUCUAAUACUUCAACGGUUAAAGAUGCCUGGAAGAGGGCGCGGUGGCUCACGCC*

*>hsa-mir-5010:17:40666165:40666366*

*AGCUCUGCAUGGUGGUCCCACUAAGGCAGUGGGAAGCCAGGAUCCAGGGAACCCUAGAGCAGGGGGAUGGCAGAGCAAAAUUCAUGGCCUACAGCUGCCUCUUGCCAAACUGCACUGGAUUUUGUGUCUCCCAUUCCCCAGAGCUGUCUGAGGUGCUUUGGACCAUGGUGAUCCACAUCGGCCUGAGCGUGAAGAGCUUG*

*>hsa-mir-4520b:17:6558694:6558895*

*CAGGCUUGCAUACACGUGCAUAACUCCUACCCCUACCUCGCCUGCCUGCUGUACCAGGGGUUGAUUCCUUCUUCCUGCGUGUUUUCUGUCCAAAUCCUUUUCUGAUUUGGACAGAAAACACGCAGGUGGCACACAGUAAGCGCUUAAUAAAUUUUCCGGCUACCUGAUCUUCUUGGAAUUGAGGGCAUGCUCCUGUGCGC*

*>hsa-mir-3974:12:17826178:17826379*

*AUCUUGCCUUGAAGUUACUUACAAAAGAUGCAGCAUCUUUUUUUCUAGGAAUAAGUUCAGGGAAAAGGUCAUUGUAAGGUUAAUGCACCCAUAUUUUAAUAUCAAACUAUGACAAAUUUGACUACAGCCUUUCCGUACCCCUGCCAAAACAUUAAGAAGAAAGCUUAAACAACAUGAAACGAGAGGAGGUAUUUCUAAAA*

*>hsa-mir-4763:22:46509395:46509596*

*CUUCCCAAGACCAGCCAGCAGCAGCUGCCCCUUCCUGGGGUGCCAUCUCCCCUGUCCCUCCUGCCCUGCGCCUGCCCAGCCCUCCUGCUCUGGUGACUGAGGACCGCCAGGCAGGGGCUGGUGCUGGGCGGGGGGCGGCGGGCCCUCCCGCAGUGCAAGGCCGGGCCUGGCGGGGUGAGGUAGUAGGUUGUGUGGUUUCA*

*>hsa-mir-5094*

*CGCCUCUGCUCUGCAGCUUGAGUGAUAGUGACAGAGCAAGACCCUGUCUCAAAAAAAAAAAAGAAAAAAAUCAGUGAAUGCCUUGAACCUAACACACUGCCUUUUAUGUGGUAGGUACAGUGGGCUCACUGAAACAUUCAACUUGUUCAACUCUAUCCAUUUGUCCAAAAACAAAAAGGAAAUGAAAAAUUCUAAAUACA*

*>hsa-mir-4796*

*CUAAGGAUGAUAUGGCUUAAGUACAUAUAGAAAAGGAAAUCAAGGCUCAAGUCAUGGCAGUAAAUUUGUGUCUAUACUCUGUCACUUUACUUUUGGCCUCAAGUCAUUGCAGUAAAGUGGCAGAGUAUAGACACAAAUUUAGGCCUUCUUCCUACUACAGACAUUUUCUUUUCACUUCUACUAGAGAUAUUUUCACUUUA*

*>hsa-mir-5008*

*CGAGUGGGGGUUCCCCAGACCCCAUGCCUUCUGGCUGGAAGUUGACCUCUGAUGGGGCUGACCCCUAGGGUCAGGUGAGGCCCUUGGGGCACAGUGGUGCCAUCUCCCCUGUGCUCCCAGGGCCUCGCCUGUCCCUUGAGGUCGGCCCCAGCUGCUGCUUCUUGCAGGAAGCCCAGUGACUGCCCAGAUGCCUCCUUCUC*

*>hsa-mir-4704:13:66792319:66792520*

*GUCUUUUCUAUGGUUGUAGUCAGUUGGUGGCUGGGACUGGAGUCAUCUUAAGGAUGAAUCUUCUUAUCCUAGACACUAGGCAUGUGAGUGAUUGUCUUCCUCACUCAAUCAGUCACAUAUCUAGUGUCUAGAAUGAGAAGACUCAAACAUCUGGGGGCUAGAACACCUCUUGUCUCUUGAUCAUCUCUCUGUGUGAUCUC*

*>hsa-mir-4737*

*AGUUGUAUUUCUAGCAAGCUGCAAGCAGAGCUGCAGUUCAGCCAGUUUGUCCUGGCCCAGCUGCACAGGAUGCGAGGAUGCUGACAGUGCCUCACAGCCGCACAGGACCGAGGAUGCUGACGGUGCCUCACAGCCACACAGGACGCGAGGAUGCUGACGGUGCCUCACAGCCACACAGGACGCGAGGAUGCUGACAGUGC*

*>hsa-mir-3978:X:109325282:109325483*

*UACCUCAGUUGACAUGUGACUUGCUCAGUCCUGCUGCUAUAGCCUGCUGCUGGAAGGGCUGCCUCAGUGGAAAGCAUGCAUCCAGGGUGUGGAGCCAAAAUUAGAAGGGCCAAAAUUCUACCUGGCCCACUACCACAGCAACCUUGGGCAUCGUUUUCUUUUGAUCUCUAGUAUCUCCAACUCCUAGAAUUCCAGCCUGG*

*>hsa-mir-516a-2:19:54264333:54264534*

*CUUGAUCUAGGAAAAUUCCAAAAAAAAAAACCCACGGUGCUGGAGCAAGAAGAUCUCAGGUUGUGACCUUCUCGAGGAAAGAAGCACUUUCUGUUGUCUGAAAGAAAAGAAAGUGCUUCCUUUCAGAGGGUUACGGUUUGAGAAAAGCAACGUCGAAGUUGACGCUGAUCUUGGUAAUACAUUUGCAGAGCGUGCUGAUC*

*>hsa-mir-4697*

*AGGUCUUGACCUCCCAUUCAGAUUUAAUUUCCUAACUGCCAGGUGUGGGGCUGGGGAUAGAGGGCCCAGAAGGGGGCGCAGUCACUGACGUGAAGGGACCACAUCCCGCUUCAUGUCAGUGACUCCUGCCCCUUGGUCUUCAGUGUUUUUCUCUUCCCCAGGAGGGACUUUGAUCAUGCAGGAUAGAAUUCUCCCAUCGC*

*>hsa-mir-4782*

*ACAAAACGGGGAAAUGUAUGCACAUGCAAUUAAUUAUAAAAACAUAACCAAACUUUAACUGAUUGCCCAGUUCUGGAUAUGAAGACAAUCAAGAAAAGAUUUGGUGUUCUUGAUUGUCUUCAUAUCUAGAACUGGGCAGUCAGUUAAAGGAGCAGGCUGUGUGUUGGUGUCAGGAUUGAGGUUAGGGAGAUUCCAUCUGG*

*>hsa-mir-103b-2*

*ACAAGCCAUAAGCUGCACCAACUGGAUCCUAGAUCCUCUUUUCCCAAGGGCAGCCCAUUCUUGGUUCUUUCAUAGCCCUGUACAAUGCUGCUUGACCUGAAUGCUACAAGGCAGCACUGUAAAGAAGCUGAAAGCACAAAGACGCAGCUCUGCUGAGGUGGGGGAGCCAAGGAGGGCACCCCUCGGCCUGCUCAGUCUUC*

*>hsa-mir-219-2*

*CUGGCGCUUUUUAGGAGCGAAGGGGAACCCCGCAGGAGACCAGGGCCCUGAACUCAGGGGCUUCGCCACUGAUUGUCCAAACGCAAUUCUUGUACGAGUCUGCGGCCAACCGAGAAUUGUGGCUGGACAUCUGUGGCUGAGCUCCGGGCGCAACAGGGGCGGGGGCCCCAGGGACAGGGCUCAGCGCGGGCGAGACCUCU*

*>hsa-mir-5692b*

*UACAAAUGAUAUCACAGUAUGUACACUCACUGUGAUAUUAGGAGUCCCAUUUUCCUAGGAUAUUAUGAAUAAUAUCACAGUAGGUGUUCACACAUAAUGUGUACACCAUGUGUGUACACCCAUGUGAUAUUUGAAGUAGUAUGUCCCUAGGAUCUUAUGAAUAAUCUCAAAAGGUGUACACCCCAUGUGACAUUAAAAGU*

*>hsa-mir-4692:11:72494506:72494707*

*AUUCUCUGCUAUCUACCUCCUCUGCCUCUAACUCUAGGCAGGUCACUGCCCCUCCCCAGGCCUCAGCAGUUUUACUUGAUACCCACACUGCCUGGGUGGGACACUCAGGCAGUGUGGGUAUCAGAUAAAACCCAAAGGGGACCCCAUUAGAGCCAAAGCCUGCUUUGCUUUGUGAACUGUAUCCUCUUGGAUCUGCCACG*

*>hsa-mir-199a-1*

*GCACGGCCUGGCCUGGUGGCCCCAGCGUCUGCCUGGGGGGUUCUGCAGGAUGGAUAGCCGGCCCCGCCAACCCAGUGUUCAGACUACCUGUUCAGGAGGCUCUCAAUGUGUACAGUAGUCUGCACAUUGGUUAGGCUGGGCUUGGGUGAGCGGCUCGUCGAGACAGGCCCCCCAAACUCGCCGGCAGGUGAGUGUCAUUU*

*>hsa-mir-4514*

*AUCUGAUGAAAAGUAAGUGAACAAGUUGCAAAGAGAGAACUGAGGAUUUGGGCAGGACAAGAGGAACUGACAGGUUGAGACAGGCAGGAUUGGGGAAACAUCUUUUACCUCGUCUCUUGCCUGUUUUAGAAGGUGCAUCCACCACUUGGUAUAAGAGAACAUUAUGGGCAUUCUGUUUUGUUUUUAUGUUUUAAAUCGUG*

*>hsa-mir-5100:10:43492970:43493171*

*AACCUGGGCUGGUGAGUCCCUCCAUCACCUUCAGAAACAGCCAUGAGGAGCUGGCAGUGGGAUGGCCUGGGGGUAGGAGCGUGGCUUCUGGAGCUAGACCACAUGGGUUCAGAUCCCAGCGGUGCCUCUAACUGGCCACAGGACCUUGGGCAGUCAGCUGACCCUGCCCAGUCCCAGCAUCCUCAUCUGGUCUUAGCUAA*

*>hsa-mir-190a:15:63116098:63116299*

*CCGUAACCUUCAAUACUGUGCCUAGUCUCGAGCUCAGUCAAACCUGGAUGCCUUUUCUGCAGGCCUCUGUGUGAUAUGUUUGAUAUAUUAGGUUGUUAUUUAAUCCAACUAUAUAUCAAACAUAUUCCUACAGUGUCUUGCCCUGUCUCCGGGGGUUCCUAAUAAAGUUUAUAAGGCAACAGGAAAGAACACGUUAAAAU*

*>hsa-mir-4757:2:19548127:19548328*

*CAUCUGUGACGUCACGAAGUUACGUAAUGGAAUGCCUCUGUGGACCUCAUUCAAGCCUUGUGUUCCAGCCCGAGGCCUCUGUGACGUCACGGUGUCUGCGGGAGGAGACCAUGACGUCACAGAGGCUUCGCGCUCUGAGAGGUGGGACGGGACUCUUUUGAGCCGGCUAUGGGGACACCAAGAUUCUAGGGGAUUUAGGA*

*>hsa-mir-4502:13:115039242:115039443*

*UCUCAAAAAAAAAAAAGAGAACACUUUAUUGUUAAAAAAAAAAAUGCUUAUGAUCCUCUAAGCCUUUAGCAAGUUGUAAUCUUUUUGCUGAUGGAGGGUCUUGCCUCCAUGGGGAUGGCUGAUGAUGAUGGUGCUGAAGGCUGGGGUGACCGGUAACUUCUUAAAAUAAGAGUGAAGUUUGCUGCAUUGAUUGACUCUUU*

*>hsa-mir-3529*

*ACACGUGGAAGGAUAGCCAAAAAGGGGGGCUGCCCCCAUUUCCUGCACCCCGCUGCGAUGGCUGGCACCAUUAGGUAGACUGGGAUUUGUUGUUGAGCGCAGUAAGACAACAACAAAAUCACUAGUCUUCCAGAUGGGGCCAGCCGGUCCACUCUGUAUCCAGGCCAGUUCUGCAAGGCGUUCGAGGACCACCCCCCUCC*

*>hsa-mir-4512*

*GAGGAGGAGUCGGGAUUUAGGCCCACGUGGGCCACUUCCCAAACCUGUGCUUAAUGCAGCCCCUCAGCCCGGGCAAUAUAGUGAGACCUCGUCUCUACAAAAAAUUGAGACAGGGCCUCACUGUAUCGCCCAGGCUGGAGUGCAGUGACAUGAUCUCGGCUCACUGCAACCUCCGCCUUCUGGGUUCAAGCGAUUCUCGU*

*>hsa-mir-548au:9:96357046:96357247*

*CACCAGCCUUAGUCCACACCGGCUGAGGACACAGACCAGCCUAAGUUCCAGUCUGAAGAUGUUAUUAGGUUGGUACAAAAGUAAUUGCGGUUUUUGCUAUUGGUUUUAAUGGCAGUUACUUUUGCACCAGCCUAAUAUCAUUCUGAAGGAAUUAUGUAUCUUCAUUUGAAGAAUGGAGAUUCUUCCUGCACAGGCUUUAA*

*>hsa-mir-4540*

*AUACCUGGACCGUCUCUGUCCAGGUCCACUCCUGGAACUCCUCAGGCUCUAGUGGAGAAUUAGGGAGGCUGAGAAGCUGCAUGGACCAGGACUUGGCACCUUUGGCCUUAGUCCUGCCUGUAGGUUUACCAGCCUUAGAGGGAAGUCACCUUUCUGUCUCUGUACCUCAGCCUGCACUUUUAUGGUCUUUUUCCCAGGGC*

*>hsa-mir-1587:X:39696741:39696942*

*UAAAAAAACACCCCAAAACUUAGUGGCUUAAAAGAGCAAUCAUUUAUCAGCCCAUGAUUCUGCAGAUCUGCAAUUUGGGCUGGGCUGGGUUGGGCAGUUCUUCUGCUGGACUCACCUGUGACCAGCUGGCAGGUCACCUGGGAACCGAAUGGUCUAGGAUGGCCUCAUGCAGUGUGUCUGGUGGUUGGUGCUGGUUGUCA*

*>hsa-mir-5192:2:62432908:62433109*

*GGCGGGGCCUGCCAGCCCUUGGCUGUCAUUUGCCACCUGAGGGACAGGCUGCUUAGUUCCAGCCUCCUGGCUCACCUGGAACCAUUUCUCCUGGGAAGCAUGGUAGCCAGGAGAGUGGAUUCCAGGUGGUGAGGGCUUGGUACUCCUUUUGGGGGAUCCUGAGUGCCAGGAGAAGAGGAUGAAAUCGAUGGACUGGGGGA*

*>hsa-mir-5699*

*GCCCAGGCUGGUCUCGAAUUCCAGCAUUUCUUCUGCCUCAGCCCCCCAAAAUGCUUCUGUACCCCUGCCCCAACAAGGAAGGACAAGAGGUGUGAGCCACACACACGCCUGGCCUCCUGUCUUUCCUUGUUGGAGCAGGGAUGUAGAAGCACUUGCCGCAGCCGACUUGAGUACAUCUGACUUUACUUAGCUUUCCUCAU*

*>hsa-mir-4437*

*CUCUCACCUGCUGAUUCCUGUGUAAUCCCUCCCAGCCAUUCAUAAGAGGACUCGGAGGUGCAGGCAACUGUACUUUGUGCAUUGGGUCCACAAGGAGGGGAUGACCCUUGUGGGCUCAGGGUACAAAGGUUCACAUUUCACACUUGGGCUUUAAUCUGCAUUAUAAUUGUUGAAUAAGGAAGUAUUUAAGCACACUAGUA*

*>hsa-mir-4730:17:78393155:78393356*

*GACAAACGAUGGCGGAGCCCGUUCUGUGCCAGAUGCUGAGCCAUGGCUGGUGUGUGCUGCUCCGCAGGCCUCUGGCGGAGCCCAUUCCAUGCCAGAUGCUGAGCGAUGGCUGGUGUGUGCUGCUCCACAGGCCUGGUGGAGCCUGUUGUGUGCUGGACGCUGAGCAAUGGCUAGUGUGUUCUGCUCCGCAGGGAGCUGAC*

*>hsa-mir-5092*

*CGGAAGCAGGGCUGCCCCUUCAGCAUUUCUCUUGAGACCUACCGCACCCCCUCACAAUCCCAGAUCAGAUGCCAAAGCCAGUGGGGACUGGACAACAUGAUGAGCCCAAACCAAUCCACGCUGAGCUUGGCAUCUGAUUUGGGAUUCUCCACCUUGCAGAGAAUCGGAAAGUAUCUCCACAAUUUCUUUUGGACCGAGCU*

*>hsa-mir-4461:5:134263667:134263868*

*UUUUUGGAGAGUCAUGUCAGUGGUAGUAAUAUAAUUGUUGGGACAAUUAGUUUUAGCAUUGGAGUAGGCUUAGGUUAUGUACGUAGUCUAGGCCAUACGUGUUGGAGAUUGAGACUAGUAGGGCUAGGCCUACUGCUGCCUUGCAGGCAGCAAAGACUAGUAUGGCGAUAGGUACAAUAUUGGCUAAGAGGGAGUGGGUG*

*>hsa-mir-5002:3:123851726:123851927*

*GGUUCCUCCUGCUGGGGCAUUUACACUGUUUUCUCUGAAGUCAAAUCCAUCUUCCUCUCUGUCCUCUGGAAUUUGGUUUCUGAGGCACUUAGUAGGUGAUAGCAUGACUGACUGCCUCACUGACCACUUCCAGAUGAGGGUUACUCUCCUCACAGCACGGCCUUGAGGCAGCUCAUGCCAGGGAUGCUGCCUGUGUUCCA*

*>hsa-mir-4523:17:27717613:27717814*

*CCUCCUUACCAGGGGUGGUGUUGGUGGCGGAGGGCUGCGCGUGGGCCCGCCCGCCGAGGGGCCGCGGCGGGGGACCGAGAGGGCCUCGGCUGUGUGAGGACUAGAGGCGGCCGAGGCCCGGGCCGGUUCCCCCGAGGCGGCGACGGAGACGGCUCCCGGCACUUCCCCGCGCCAUCUUAACUGAGCCCAAGCGCUGAGGG*

*>hsa-mir-5001*

*CGGAACGUCGGCGCGCAGGCCCGGUUUGGAAGCGGCUAUCUCGGGACGCCAGCUCAGGGCGGCUGCGCAGAGGGCUGGACUCAGCGGCGGAGCUGGCUGCUGGCCUCAGUUCUGCCUCUGUCCAGGUCCUUGUGACCCGCCCGCUCUCCUAAGGGAGCGUGUCCUGGCCCCCUCCCGCAGCGGCUCGGAAAAAAGGCAGC*

*>hsa-mir-4689*

*CCCUUGGCAGUUUUCUUGAGUGUCUGCUGGCGCAGUGUCAGAGGCUUCCUGUCUACAGCACAAGCCCGGUUUCUCCUUGAGGAGACAUGGUGGGGGCCGGUCAGGCAGCCCAUGCCAUGUGUCCUCAUGGAGAGGCCGCUCAAGCCUGCCCUGGGUGGGGGCUGAGACCCCGGCUCUGAGACGGCAGCAGUCGGGUAGAA*

*>hsa-mir-103a-2:20:3898077:3898278*

*GAGAAGACUGAGCAGGCCGAGGGGUGCCCUCCUUGGCUCCCCCACCUCAGCAGAGCUGCGUCUUUGUGCUUUCAGCUUCUUUACAGUGCUGCCUUGUAGCAUUCAGGUCAAGCAGCAUUGUACAGGGCUAUGAAAGAACCAAGAAUGGGCUGCCCUUGGGAAAAGAGGAUCUAGGAUCCAGUUGGUGCAGCUUAUGGCUU*

*>hsa-mir-4457*

*UGGGACUACAGGCGUGAGCCACCACGCCCAGCCCAAACGUCAAUUAUCUUGGCAUGAUGACUCUUUGGAGUACUCCAGUCAAUACCGUGUGAGUUAGAAAAGCUCAAUUCACAAGGUAUUGACUGGCGUAUUCAGAAGAGUCUGGCCUCAGUAGCGGAGAAAAAACCUUCCUCGUGGAUCAACCCCUGCCCUGGUCCCAC*

*>hsa-mir-323a:14:101492010:101492211*

*AGGAGGUGAUAUCAGCUUUGCGGAAGAGCCACUGUCCUGGUGUCAGUACGGCUGCUGCUUGGUACUUGGAGAGAGGUGGUCCGUGGCGCGUUCGCUUUAUUUAUGGCGCACAUUACACGGUCGACCUCUUUGCAGUAUCUAAUCCCGCCUUGCAAGCUUUCCUGGAGCUAACAUCAACUGCGGGGGUGGGGGCCACUAGG*

*>hsa-mir-512-2:19:54172358:54172559*

*UUCUGCAUAGAGGAUGUGCCUGCAGUUUCCAGGUACUGGACAAUACAGGGAGGGUACUUCUCAGUCUGUGGCACUCAGCCUUGAGGGCACUUUCUGGUGCCAGAAUGAAAGUGCUGUCAUAGCUGAGGUCCAAUGACUGAGGCGAGCACCGAAAAAACACCAUGGGGGGGAGGGGGGGCGGGGGGCUCCAGGAGCCACUG*

*>hsa-mir-4731*

*UGGAUGUUGAUCUUUGGUGCCCGAGAGAAGGCUGUCUGGGAUGAAGCCUUCUGACUGUGGUCAGGUCCCUGCCAGUGCUGGGGGCCACAUGAGUGUGCAGUCAUCCACACACAAGUGGCCCCCAACACUGGCAGGGACCCGGAGUCAUCUGGCCCAUCCCUCCUUCCUAUCCCAUCGAGCCACAGACCCCUCUGCAAUAU*

*>hsa-mir-499b*

*CCCUCCCAGAGCCCCAGGGCUCCCACCCCUUCCCCACAAACCCUGCCCAGGCAGCGUAGGGACGGGAAGCAGCACAGACUUGCUGUGAUGUUCACGUGGAGAGGAGUUAAACAUCACUGCAAGUCUUAACAGCCGCCCGCCCAAGGCACAGGGGACAGGGCAGGGGGUGAAGGACGGGGCUGGAGGCUGCCAAGAGGGAG*

*>hsa-mir-4721*

*GGCCGGAGAUAACCUCGGGGCCCUGGUCCGAGGCUUGAAGCGGGAGGACUUGCGGCGGGGCCUGGUCAUGGUCAAGCCAGGUUCCAUCAAGCCCCACCAGAAGGUGGAGGCCCAGGUGAGGGCUCCAGGUGACGGUGGGCAGGGUUGAGCCAAGCUCUCCCCAGCCUCCAGCCAAGCCCAGCUCACCGUCAUUGCUUGCU*

*>hsa-mir-4794:1:65045468:65045669*

*CAGGCAGUGUGCUAGAGCCUGAAGAUACAGUGGUGAACAAAAACAUAUAUCGUAUGUUAUCUUUUAACAUCUGGCUAUCUCACGAGACUGUAUGUCCUAACAGUGCUUGUAGUCUCAUGAGAUAGCCAGAUGUUAAAAAAAAAAAACAGCAUCAAAUUUAAUGUGUGUUAUGAGAUGGCUUAUGCAGGGCAUUAUGAGAA*

*>hsa-mir-3689f*

*GUGAUAUCGUGGUUCCUGGGAGGUGUGAUCCUGUGCUUCCUGGAAGGUGUGAUCCUGUGCUUCCUGGGAGGUGUGAUAUCGUGCUUCCUGGGACGUGUGAUGCUGUGCUUCCUGGGAGGUGUGAUCCCACACUCGCUGGGAGGUGUGAUCCCGUGCUCCCUGGGAGGUCUGAUCCCGUGCUUUCCUGGGAGGUGUGAUCC*

*>hsa-mir-5088:19:50185269:50185470*

*CCGGCAUCCUCUGCAUGUUUGCUGCCAAGGCCGGGGCCCGCAAGGUCAUCGGGGUGAGUCUCCAGGGUGGCCAGGCGGGGCCGGGCCUGAGGGAUGGAGGGGAGCCCAUCAGGGCUCAGGGAUUGGAUGGAGGUGAUGGGGGCAGGGGAUGGGUCUCACCCUCCCUUCUUCCUGGGCCCUCAGAUCGAGUGUUCCAGUAU*

*>hsa-mir-4440*

*UCCCUCUCACCAAGCAAGUGCAGUGGGGCUUGCUGGCUUGCACCGUGACUCCCUCUCACCAAGCAAGUGCAGUGGGGCUUGCUGGCUUGCACCGUGACUCCCUCUCACCAAGCAAGUGUCGUGGGGCUUGCUGGCUUGCACUGUGAAGAUGUGAAAUGAGCCUGGAUCACACGUGGGUUGAUUCCCUUCUAAUUAAGCGU*

*>hsa-mir-548ad:2:35696411:35696612*

*UUACAAGAAUUCUAUAAGGAUCCAGGAUCAAAACACAACAAUUUUCACAUACGUGUCAACUGUUAGGUUGGUGCAAAAGUAAUUGUGGUUUUUGAAAGUAACUUGGCGAAAACGACAAUGACUUUUGCACCAAUCUAAUACAAUCAACAGUUGUGGGAAGAAUUUUAACUUUUCACUCUAUGUUUGUACUGUUUUAGUGU*

*>hsa-mir-4760*

*GGGAAAGGAUGUACUGUCCAAGCGACAACUAGACUGCAGAGGUAAGGCUGAGACCAGGGCUGCCAUGGUGUUUAGAUUGAACAUGAAGUUAGAAUUCUUAAGUAUCAAAACUAAAUUCAUGUUCAAUCUAAACCCCAUGGCUAUGCCUCUCAAGGUGAGGCCUCUGUGUUGUUCUCUAUGCCUUGAGCACCCUUCCUUCU*

*>hsa-mir-5694*

*GCCUAUAUUAGUCUGGAAUUUCCAGAGAGACAGAACCAAUAGGAAGGAGAUAGAUAUUUAUAGCCAACUGCAGAUCAUGGGACUGUCUCAGCCCCAUAUGUAUCUGAAGGCUGAGAAGUCCCAUGAUCCGCACUUGGCAAGCUGGAGACUCAGGAGAGCCAGUGCUGUGAGUUCCAGUCCGAGAACGAAGGCAGGAGAAG*

*>hsa-mir-3198-1*

*CAUGAAAAAGCACAAGGUCCUGGCUGCUUGCAGUCACAUUCUGGUUCUCUGUGUUUUGUGGACUGUGCUCUCACUGUUCACCCAGCACUAGCAGUACCAGACGGUUCUGUGGAGUCCUGGGGAAUGGAGAGAGCACAGUCUGACGCCCUGCCAAGUAGCCAGGAGUUGACUUGCCCAUGGUCCGCUGGCUUUCCCACCAC*

*>hsa-mir-4786*

*AGAUAACUGUGAGUUGAAACUCUCCGUAAGAAGUGGAGGCUCCAGGGAUGCGUUGGCCUGUGGGCAUGGCCUGAGACCAGGACUGGAUGCACCACUCUCCCUGUGAUGAGGUGAAGCCAGCUCUGGUCUGGGCCAUUUCACAGGAUUCCAGAAGCAGGAGCAGAGCCUCAGCAUUUGAACGAACCUUCUUCCACUUCGGC*

*>hsa-mir-4752:19:54785899:54786100*

*GGGUGUUCAUAGAGCAUUUAUUAGGGGGACCUCUGCACAGUGGGGCAUCCUUGUCUUCUUGCCCAGUGUCUCCUUGUGGAUCUCAAGGAUGUGCUUCCACAUAGCAGCAUGUUCUUCAGAUGGACAAGGAGACACUGGGUAUUCUAUCCAAAGCUUUAACCUAAAAUAAAAACAAAACCAAAAAUAAACCCCUAGAAAAU*

*>hsa-mir-4452*

*UUUUUUUUAGAGAGAGUCUCACGUUGCCCAGGCUAGAGUGCAGUGGCUAUUCACAGGUGCGAUCAUGGAUCACUUGAGGCCAAGAGUGCAAGGCUGUAGUGUGCACAGCCUUGAAUUCUUGGCCUUAAGUGAUCCCUGUCCCAGCCUCCUGAAUAGCUGGGACUACAGGUACACACCACUGUGUCCAGCUAGGGAGUUGA*

*>hsa-mir-4724:17:29861844:29862045*

*GUAGUAGACUCAGAUUUUACAUGGCCCUGAGCUGCCCUUGCGUUCAGUUGGAAGCCACGCAAAAUGAACUGAACCAGGAGUGAGCUUCGUGUACAUUAUCUAUUAGAAAAUGAAGUACCUUCUGGUUCAGCUAGUCCCUGUGCGUUGAGAGGCUUUAAGAAACUUCUAGUAAAAAUGUUUUUUUCUUUUAGCUUUGAAUU*

*>hsa-mir-3155a:10:6194100:6194301*
[truncated: 1,373,049 more chars]
