# Supplementary material for: miR-BAG: Bagging Based Identification of MicroRNA Precursors
Source: PLoS One. 2012 Sep 25;7(9):e45782. doi: 10.1371/journal.pone.0045782 (PMC3458082; doi:10.1371/journal.pone.0045782)
Supplement: Supporting Material S2 — Implementation details for parallelism in miR-BAG. (DOC) [file pone.0045782.s002.doc]

**Supporting Information S2: Introduction of concurrency**

In miR-BAG divide and conquer approach has been implemented to achieve parallelism. Parallelism has been implemented by applying Java Concurrent Library (JCL). Parallelism has been implemented at three major levels:

**Sequence level parallelism**

miR-BAG works on the fragment size of 200 bp. However, if the sequence length exceeds 200bp then miR-BAG splits the sequence in sliding windows of 200bp region. It depends upon the user to specify the number of sequences of length 200 bp to be taken at once. If user selects two processors then two fragments of 200 bp are executed at the same time and so on (**figure S1**). If the length of all submitted sequences is exactly 200bp and user has “*n”* number of sequences in input file then number of sequences executed simultaneously depends upon the number of processors opted by the user.

**W****indows level parallelism**

For each 200bp fragment, data generation and classification have been done on sliding windows of length 21. The 200bp fragment is further divided into three regions. The left region which consists of nucleotides from position 1-100 and consist of 80 sliding windows of length 21 nucleotides each. The Right region which consists of nucleotides from position 101-200 also consists of 80 sliding windows of length 21 nucleotides each. The Fixed region consists of middle most 21 nucleotides. All the analysis of different Class I features (mononucleotide, dinucleotide, structural triplet etc features) is done simultaneously on these three regions for every sequence. Every windows is calculated simultaneously through threads instead of following iteratively one after another.

**Matrix probability level parallelism**

This is the Class II feature which assigns scores to the sequence on the basis of pre-calculated structural profile matrices. The profiles created for different miRNAs are used to score the input sequence. To detect the best matrix score for a sequence given by the user, the secondary structure representation of the sequence is mapped through all profiles through overlapping sliding manner, scoring for every position. For every position, all profiles are scanned. This entire process requires a lot of time as there are large number of matrices for different miRNAs in each species. Depending upon the processor load, the program distributes the process of matrices scanning in parallel and batch mode, cutting time by number of batches created.


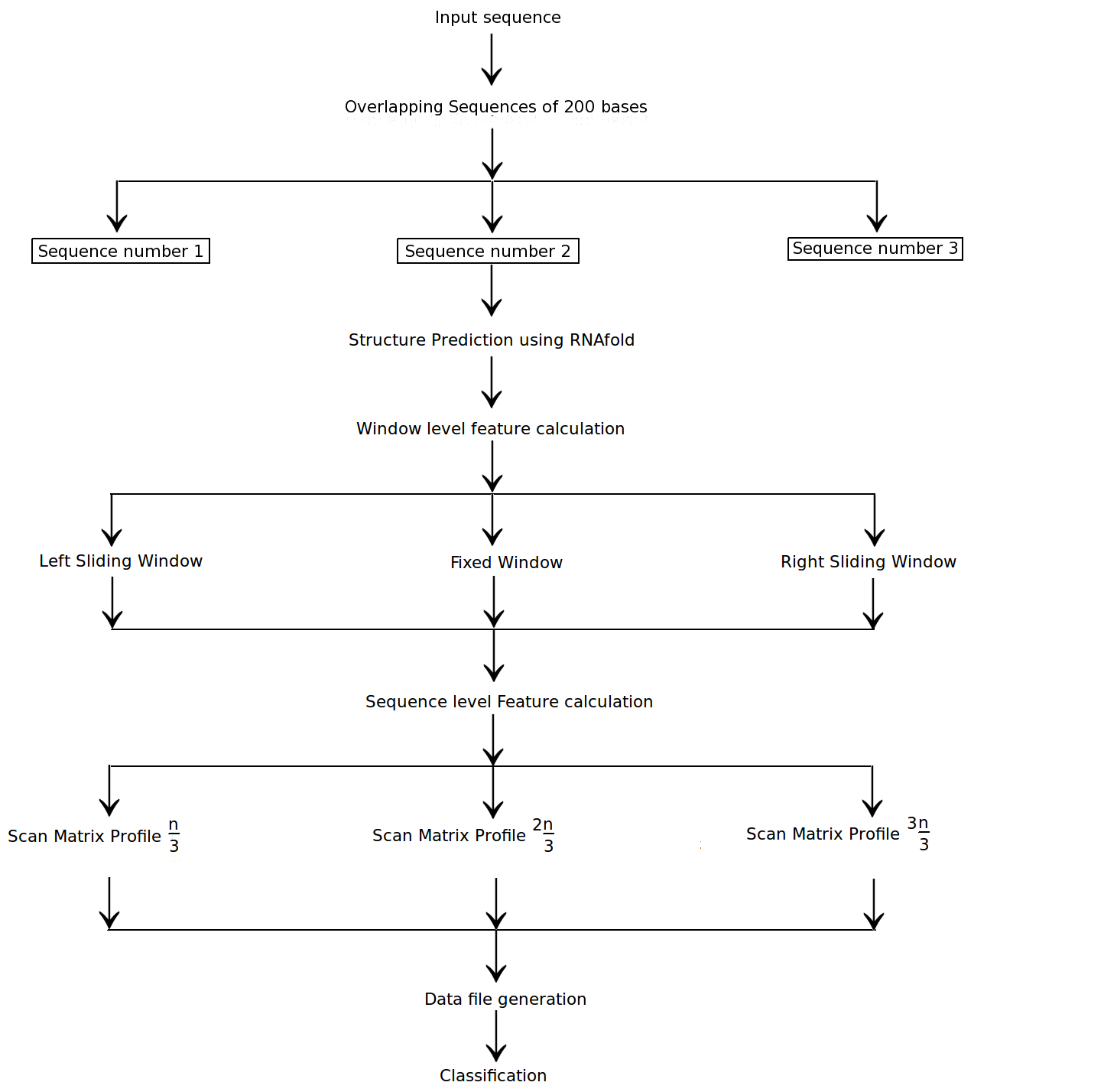


**Figure S1. Implementation of parallelism.** Flow chart describing the different level of Parallel architecture developed in miR-BAG when three processors are involved in execution.
