# Supplementary material for: miR-BAG: Bagging Based Identification of MicroRNA Precursors
Source: PLoS One. 2012 Sep 25;7(9):e45782. doi: 10.1371/journal.pone.0045782 (PMC3458082; doi:10.1371/journal.pone.0045782)
Supplement: Supporting Material S3 — Energy and Structural profile matrix score plots drawn between the positive and negative instances for all target species, depicting the distribution patterns for positive and negative instances. (DOC) [file pone.0045782.s003.doc]

**Supporting Information 3: Energy and Matrix score plots**

a- Matrix score distribution of various species in the given class intervals.

Drosophila melanogaster

| Class interval | Number positive instances falling in this interval | Number negative instances falling in this interval | % of positive instances(miRNA) | % of negative instances(Non-miRNA) |
| --- | --- | --- | --- | --- |
| 0.004080102 | 4 | 51 | 1.8518518519 | 23.0769230769 |
| 0.0081602041 | 10 | 52 | 4.6296296296 | 23.5294117647 |
| 0.0122403061 | 16 | 25 | 7.4074074074 | 11.3122171946 |
| 0.0163204081 | 32 | 25 | 14.8148148148 | 11.3122171946 |
| 0.0204005102 | 43 | 32 | 19.9074074074 | 14.479638009 |
| 0.0244806122 | 44 | 21 | 20.3703703704 | 9.5022624434 |
| 0.0285607142 | 40 | 9 | 18.5185185185 | 4.07239819 |
| 0.0326408162 | 14 | 3 | 6.4814814815 | 1.3574660633 |
| 0.0367209183 | 13 | 3 | 6.0185185185 | 1.3574660633 |

Canis familiaris

| Class interval | Number positive instances falling in this interval | Number negative instances falling in this interval | % of positive instances(miRNA) | % of negative instances(Non-miRNA) |
| --- | --- | --- | --- | --- |
| 0.0058837759 | 1 | 55 | 0.3676470588 | 17.6282051282 |
| 0.0117675519 | 6 | 107 | 2.2058823529 | 34.2948717949 |
| 0.0176513278 | 9 | 55 | 3.3088235294 | 17.6282051282 |
| 0.0235351038 | 26 | 46 | 9.5588235294 | 14.7435897436 |
| 0.0294188797 | 42 | 34 | 15.4411764706 | 10.8974358974 |
| 0.0353026556 | 67 | 11 | 24.6323529412 | 3.5256410256 |
| 0.0411864316 | 86 | 4 | 31.6176470588 | 1.2820512821 |
| 0.0470702075 | 18 | 0 | 6.6176470588 | 0 |
| 0.0529539835 | 17 | 0 | 6.25 | 0 |

Caenorhabditis elegans

| Class interval | Number positive instances falling in this interval | Number negative instances falling in this interval | % of positive instances(miRNA) | % of negative instances(Non-miRNA) |
| --- | --- | --- | --- | --- |
| 0.0066552632 | 4 | 61 | 2.0100502513 | 29.0476190476 |
| 0.0133105263 | 10 | 33 | 5.0251256281 | 15.7142857143 |
| 0.0199657895 | 5 | 45 | 2.5125628141 | 21.4285714286 |
| 0.0266210526 | 28 | 37 | 14.0703517588 | 17.619047619 |
| 0.0332763158 | 33 | 23 | 16.5829145729 | 10.9523809524 |
| 0.0399315789 | 52 | 7 | 26.1306532663 | 3.3333333333 |
| 0.0465868421 | 48 | 4 | 24.1206030151 | 1.9047619048 |
| 0.0532421053 | 10 | 0 | 5.0251256281 | 0 |
| 0.0598973684 | 9 | 0 | 4.5226130653 | 0 |

Homo sapiens

| Class interval | Number positive instances falling in this interval | Number negative instances falling in this interval | % of positive instances(miRNA) | % of negative instances(Non-miRNA) |
| --- | --- | --- | --- | --- |
| 0.0056042943 | 5 | 45 | 0.4840271055 | 3.6555645816 |
| 0.0112085886 | 30 | 275 | 2.9041626331 | 22.3395613323 |
| 0.0168128829 | 77 | 267 | 7.454017425 | 21.6896831844 |
| 0.0224171772 | 117 | 272 | 11.3262342691 | 22.0958570268 |
| 0.0280214715 | 201 | 182 | 19.4578896418 | 14.7847278635 |
| 0.0336257658 | 220 | 126 | 21.2971926428 | 10.2355808286 |
| 0.0392300601 | 214 | 48 | 20.7163601162 | 3.8992688871 |
| 0.0448343544 | 85 | 8 | 8.2284607938 | 0.6498781478 |
| 0.0504386486 | 84 | 8 | 8.1316553727 | 0.6498781478 |

Mus musculus

| Class interval | Number positive instances falling in this interval | Number negative instances falling in this interval | % of positive instances(miRNA) | % of negative instances(Non-miRNA) |
| --- | --- | --- | --- | --- |
| 0.0054573247 | 3 | 64 | 0.4601226994 | 9.1954022989 |
| 0.0109146495 | 17 | 218 | 2.6073619632 | 31.3218390805 |
| 0.0163719742 | 28 | 161 | 4.2944785276 | 23.132183908 |
| 0.021829299 | 71 | 134 | 10.8895705521 | 19.2528735632 |
| 0.0272866237 | 143 | 83 | 21.9325153374 | 11.9252873563 |
| 0.0327439485 | 190 | 28 | 29.1411042945 | 4.0229885057 |
| 0.0382012732 | 174 | 8 | 26.6871165644 | 1.1494252874 |
| 0.0436585979 | 13 | 0 | 1.9938650307 | 0 |
| 0.0491159227 | 12 | 0 | 1.8404907975 | 0 |
| 0.0545732474 | 1 | 0 | 0.1533742331 | 0 |

Rattus norvegicus

| Class interval | Number positive instances falling in this interval | Number negative instances falling in this interval | % of positive instances(miRNA) | % of negative instances(Non-miRNA) |
| --- | --- | --- | --- | --- |
| 0.0075891966 | 1 | 91 | 0.2747252747 | 24.8633879781 |
| 0.0151783932 | 19 | 115 | 5.2197802198 | 31.4207650273 |
| 0.0227675898 | 18 | 65 | 4.9450549451 | 17.7595628415 |
| 0.0303567864 | 38 | 60 | 10.4395604396 | 16.393442623 |
| 0.037945983 | 82 | 28 | 22.5274725275 | 7.650273224 |
| 0.0455351796 | 104 | 6 | 28.5714285714 | 1.6393442623 |
| 0.0531243762 | 79 | 1 | 21.7032967033 | 0.2732240437 |
| 0.0607135728 | 12 | 0 | 3.2967032967 | 0 |
| 0.0683027694 | 11 | 0 | 3.021978022 | 0 |

b- MFE value of various species in the given class intervals.

Canis familiaris

| Class interval | Number positive instances falling in this interval | Number negative instances falling in this interval | % of positive instances(miRNA) | % of negative instances(Non-miRNA) |
| --- | --- | --- | --- | --- |
| -14.06 | 24 | 148 | 7.570977918 | 47.1337579618 |
| -28.12 | 122 | 124 | 38.4858044164 | 39.4904458599 |
| -42.18 | 62 | 22 | 19.5583596215 | 7.0063694268 |
| -56.24 | 56 | 10 | 17.665615142 | 3.1847133758 |
| -70.3 | 24 | 9 | 7.570977918 | 2.8662420382 |
| -84.36 | 16 | 1 | 5.047318612 | 0.3184713376 |
| -98.42 | 8 | 0 | 2.523659306 | 0 |
| -112.48 | 3 | 0 | 0.9463722397 | 0 |
| -126.54 | 2 | 0 | 0.6309148265 | 0 |

Drosophila melanogaster

| Class interval | Number positive instances falling in this interval | Number negative instances falling in this interval | % of positive instances(miRNA) | % of negative instances(Non-miRNA) |
| --- | --- | --- | --- | --- |
| -8.67 | 8 | 54 | 6.9565217391 | 24 |
| -17.34 | 32 | 79 | 27.8260869565 | 35.1111111111 |
| -26.01 | 25 | 26 | 21.7391304348 | 11.5555555556 |
| -34.68 | 16 | 31 | 13.9130434783 | 13.7777777778 |
| -43.35 | 16 | 18 | 13.9130434783 | 8 |
| -52.02 | 5 | 6 | 4.347826087 | 2.6666666667 |
| -60.69 | 6 | 7 | 5.2173913043 | 3.1111111111 |
| -69.36 | 4 | 2 | 3.4782608696 | 0.8888888889 |
| -78.03 | 3 | 2 | 2.6086956522 | 0.8888888889 |

Caenorhabditis elegans

| Class interval | Number positive instances falling in this interval | Number negative instances falling in this interval | % of positive instances(miRNA) | % of negative instances(Non-miRNA) |
| --- | --- | --- | --- | --- |
| -14.39 | 8 | 21 | 3.7037037037 | 10 |
| -28.78 | 141 | 165 | 65.2777777778 | 78.5714285714 |
| -43.17 | 44 | 23 | 20.3703703704 | 10.9523809524 |
| -57.56 | 12 | 1 | 5.5555555556 | 0.4761904762 |
| -71.95 | 8 | 0 | 3.7037037037 | 0 |
| -86.34 | 2 | 0 | 0.9259259259 | 0 |
| -100.73 | 0 | 0 | 0 | 0 |
| -115.12 | 1 | 0 | 0.462962963 | 0 |

Homo sapiens

| Class interval | Number positive instances falling in this interval | Number negative instances falling in this interval | % of positive instances(miRNA) | % of negative instances(Non-miRNA) |
| --- | --- | --- | --- | --- |
| -16.96 | 19 | 292 | 1.7625231911 | 23.5673930589 |
| -33.92 | 443 | 721 | 41.094619666 | 58.1920903955 |
| -50.88 | 334 | 159 | 30.9833024119 | 12.8329297821 |
| -67.84 | 177 | 50 | 16.4192949907 | 4.0355125101 |
| -84.8 | 78 | 16 | 7.2356215213 | 1.2913640032 |
| -101.76 | 22 | 1 | 2.0408163265 | 0.0807102502 |
| -118.72 | 2 | 0 | 0.185528757 | 0 |
| -135.68 | 2 | 0 | 0.185528757 | 0 |
| -152.64 | 1 | 0 | 0.0927643785 | 0 |

Mus musculus

| Class interval | Number positive instances falling in this interval | Number negative instances falling in this interval | % of positive instances(miRNA) | % of negative instances(Non-miRNA) |
| --- | --- | --- | --- | --- |
| -12.95 | 4 | 69 | 0.5797101449 | 9.9137931034 |
| -25.9 | 117 | 440 | 16.9565217391 | 63.2183908046 |
| -38.85 | 198 | 113 | 28.6956521739 | 16.2356321839 |
| -51.8 | 194 | 46 | 28.115942029 | 6.6091954023 |
| -64.75 | 101 | 21 | 14.6376811594 | 3.0172413793 |
| -77.7 | 48 | 7 | 6.9565217391 | 1.0057471264 |
| -90.65 | 21 | 0 | 3.0434782609 | 0 |
| -103.6 | 4 | 0 | 0.5797101449 | 0 |
| -116.55 | 3 | 0 | 0.4347826087 | 0 |

Rattus norvegicus

| Class interval | Number positive instances falling in this interval | Number negative instances falling in this interval | % of positive instances(miRNA) | % of negative instances(Non-miRNA) |
| --- | --- | --- | --- | --- |
| -11.34 | 6 | 108 | 1.5831134565 | 29.5081967213 |
| -22.68 | 79 | 178 | 20.8443271768 | 48.6338797814 |
| -34.02 | 77 | 43 | 20.3166226913 | 11.7486338798 |
| -45.36 | 93 | 24 | 24.5382585752 | 6.5573770492 |
| -56.7 | 59 | 8 | 15.5672823219 | 2.1857923497 |
| -68.04 | 41 | 4 | 10.8179419525 | 1.0928961749 |
| -79.38 | 17 | 1 | 4.4854881266 | 0.2732240437 |
| -90.72 | 4 | 0 | 1.055408971 | 0 |
| -102.06 | 3 | 0 | 0.7915567282 | 0 |
