# Supplementary material for: miR-BAG: Bagging Based Identification of MicroRNA Precursors
Source: PLoS One. 2012 Sep 25;7(9):e45782. doi: 10.1371/journal.pone.0045782 (PMC3458082; doi:10.1371/journal.pone.0045782)
Supplement: Supporting Material S5 — The top 15 feature scores along with the feature names and type of the features obtained after feature selection for every species. (DOC) [file pone.0045782.s005.doc]

**Supporting Information S5 : Top 15 feature with feature score of all species considered in this study.**

Homo sapiens

| Position | Feature-score | Feature name | Feature Description |
| --- | --- | --- | --- |
| 1 | 0.63 | ))) | Structural Triplet feature in 8^th^ sliding Right window |
| 2 | 0.62 | ))) | Structural Triplet feature in 9^th^ sliding Right window |
| 3 | 0.62 | ))) | Structural Triplet feature in 7^th^ sliding Right window |
| 4 | 0.61 | ))) | Structural Triplet feature in 10^th^ sliding Right window |
| 5 | 0.6 | ))) | Structural Triplet feature in 6^th^ sliding Right window |
| 6 | 0.58 | ((( | Structural Triplet feature in 72^nd^ sliding Left window |
| 7 | 0.58 | ((( | Structural Triplet feature in 71^st^ sliding Left window |
| 8 | 0.58 | ))) | Structural Triplet feature in 11^th^ sliding Right window |
| 9 | 0.57 | ((( | Structural Triplet feature in 73^rd^ sliding Left window |
| 10 | 0.56 | ((( | Structural Triplet feature in 70^th^ sliding Left window |
| 11 | 0.56 | ))) | Structural Triplet feature in 12^th^ sliding Right window |
| 12 | 0.55 | ((( | Structural Triplet feature in 69^th^ sliding Left window |
| 13 | 0.55 | ((( | Structural Triplet feature in 68^th^ sliding Left window |
| 14 | 0.54 | ))) | Structural Triplet feature in 5^th^ sliding Right window |
| 15 | 0.54 | ))) | Structural Triplet feature in 13^th^ sliding Right window |
| 17 | 0.53 | *Matrix* | Structural profile matrix based scoring features |

Caenorhabditis elegans

| Position | Feature-score | Feature name. | Feature Description |
| --- | --- | --- | --- |
| 1 | 0.89 | Matrix | Structural profile matrix based scoring features |
| 2 | 0.48 | ((( | Structural Triplet feature in 72 sliding Left window |
| 3 | 0.48 | ((( | Structural Triplet feature in 73 sliding Left window |
| 4 | 0.48 | ((( | Structural Triplet feature in 74 sliding Left window |
| 5 | 0.46 | ((( | Structural Triplet feature in 71 sliding Left window |
| 6 | 0.46 | ))) | Structural Triplet feature in 18 sliding Right window |
| 7 | 0.45 | ))) | Structural Triplet feature in 19 sliding Right window |
| 8 | 0.45 | ))) | Structural Triplet feature in 17 sliding Right window |
| 9 | 0.44 | ))) | Structural Triplet feature in 24 sliding Right window |
| 10 | 0.44 | ((( | Structural Triplet feature in 75 sliding Left window |
| 11 | 0.44 | ))) | Structural Triplet feature in 20 sliding Right window |
| 12 | 0.44 | ))) | Structural Triplet feature in 23 sliding Right window |
| 13 | 0.43 | ))) | Structural Triplet feature in 21 sliding Right window |
| 14 | 0.43 | ((( | Structural Triplet feature in 70 sliding Left window |
| 15 | 0.43 | ((( | Structural Triplet feature in 76 sliding Left window |

Drosophila melanogester

| Position | Feature-score | Feature name | Feature Description |
| --- | --- | --- | --- |
| 1 | 0.64 | ))) | Structural Triplet feature in 10 sliding Right window |
| 2 | 0.64 | ))) | Structural Triplet feature in 11 sliding Right window |
| 3 | 0.63 | ((( | Structural Triplet feature in 71 sliding Left window |
| 4 | 0.63 | ))) | Structural Triplet feature in 9 sliding Right window |
| 5 | 0.61 | ((( | Structural Triplet feature in 72 sliding Left window |
| 6 | 0.6 | ))) | Structural Triplet feature in 12 sliding Right window |
| 7 | 0.58 | ((( | Structural Triplet feature in 70 sliding Left window |
| 8 | 0.58 | ))) | Structural Triplet feature in 13 sliding Right window |
| 9 | 0.56 | ((( | Structural Triplet feature in 73 sliding Left window |
| 10 | 0.55 | ))) | Structural Triplet feature in 14 sliding Right window |
| 11 | 0.55 | ))) | Structural Triplet feature in 8 sliding Right window |
| 12 | 0.51 | ))) | Structural Triplet feature in 15 sliding Right window |
| 13 | 0.5 | ((( | Structural Triplet feature in 69 sliding Left window |
| 14 | 0.48 | ((( | Structural Triplet feature in 74 sliding Left window |
| 15 | 0.47 | ))) | Structural Triplet feature in 7 sliding Right window |
| 49 | 0.34 | Matrix | Structural profile matrix based scoring features |

Rattus norvegicus

| Position | Feature-score | Feature name | Feature Description |
| --- | --- | --- | --- |
| 1 | 1.03 | Matrix | Structural profile matrix based scoring features |
| 2 | 0.99 | ))) | Structural Triplet feature in 9 sliding Right window |
| 3 | 0.97 | ))) | Structural Triplet feature in 8 sliding Right window |
| 4 | 0.95 | ))) | Structural Triplet feature in 10 sliding Right window |
| 5 | 0.94 | ))) | Structural Triplet feature in 7 sliding Right window |
| 6 | 0.9 | ))) | Structural Triplet feature in 11 sliding Right window |
| 7 | 0.87 | ))) | Structural Triplet feature in 12 sliding Right window |
| 8 | 0.83 | ))) | Structural Triplet feature in 6 sliding Right window |
| 9 | 0.81 | ((( | Structural Triplet feature in 72 sliding Left window |
| 10 | 0.8 | ))) | Structural Triplet feature in 13 sliding Right window |
| 11 | 0.8 | ((( | Structural Triplet feature in 73 sliding Left window |
| 12 | 0.79 | ((( | Structural Triplet feature in 71 sliding Left window |
| 13 | 0.78 | ((( | Structural Triplet feature in 69 sliding Left window |
| 14 | 0.77 | ((( | Structural Triplet feature in 68 sliding Left window |
| 15 | 0.77 | ((( | Structural Triplet feature in 70 sliding Left window |

Canis familiaris

| Position | Feature-score | Feature name | Feature Description |
| --- | --- | --- | --- |
| 1 | 1.16 | ))) | Structural Triplet feature in 9 sliding Right window |
| 2 | 1.16 | Matrix | Structural profile matrix based scoring features |
| 3 | 1.16 | ))) | Structural Triplet feature in 8 sliding Right window |
| 4 | 1.12 | ((( | Structural Triplet feature in 72 sliding Left window |
| 5 | 1.11 | ))) | Structural Triplet feature in 10 sliding Right window |
| 6 | 1.11 | ))) | Structural Triplet feature in 11 sliding Right window |
| 7 | 1.1 | ))) | Structural Triplet feature in 7 sliding Right window |
| 8 | 1.09 | ((( | Structural Triplet feature in 73 sliding Left window |
| 9 | 1.09 | ((( | Structural Triplet feature in 71 sliding Left window |
| 10 | 1.08 | ((( | Structural Triplet feature in 70 sliding Left window |
| 11 | 1.04 | ))) | Structural Triplet feature in 12 sliding Right window |
| 12 | 1.03 | ))) | Structural Triplet feature in 15 sliding Right window |
| 13 | 1.01 | ))) | Structural Triplet feature in 13 sliding Right window |
| 14 | 1.01 | ))) | Structural Triplet feature in 14 sliding Right window |
| 15 | 1 | ((( | Structural Triplet feature in 69 sliding Left window |

Mus musculus

| Position | Feature-score | Feature name | Feature Description |
| --- | --- | --- | --- |
| 1 | 0.81 | Matrix | Structural profile matrix based scoring features |
| 2 | 0.35 | ... | Structural Triplet feature in 79 sliding Left window |
| 3 | 0.34 | ... | Structural Triplet feature in 78 sliding Left window |
| 4 | 0.33 | ... | Structural Triplet feature in 77 sliding Left window |
| 5 | 0.31 | ... | Structural Triplet feature in 76 sliding Left window |
| 6 | 0.3 | ... | Structural Triplet feature in 75 sliding Left window |
| 7 | 0.3 | ((( | Structural Triplet feature in 79 sliding Left window |
| 8 | 0.28 | ... | Structural Triplet feature in 74 sliding Left window |
| 9 | 0.27 | ((( | Structural Triplet feature in 78 sliding Left window |
| 10 | 0.27 | ... | Structural Triplet feature in 73 sliding Left window |
| 11 | 0.26 | ))) | Structural Triplet feature in 13 sliding Right window |
| 12 | 0.26 | ))) | Structural Triplet feature in 14 sliding Right window |
| 13 | 0.26 | ... | Structural Triplet feature in 13 sliding Right window |
| 14 | 0.26 | ... | Structural Triplet feature in 14 sliding Right window |
| 15 | 0.26 | ))) | Structural Triplet feature in 15 sliding Right window |
