# Supplementary figures and images for: miR-BAG: Bagging Based Identification of MicroRNA Precursors
Source: PLoS One. 2012 Sep 25;7(9):e45782. doi: 10.1371/journal.pone.0045782 (PMC3458082; doi:10.1371/journal.pone.0045782)

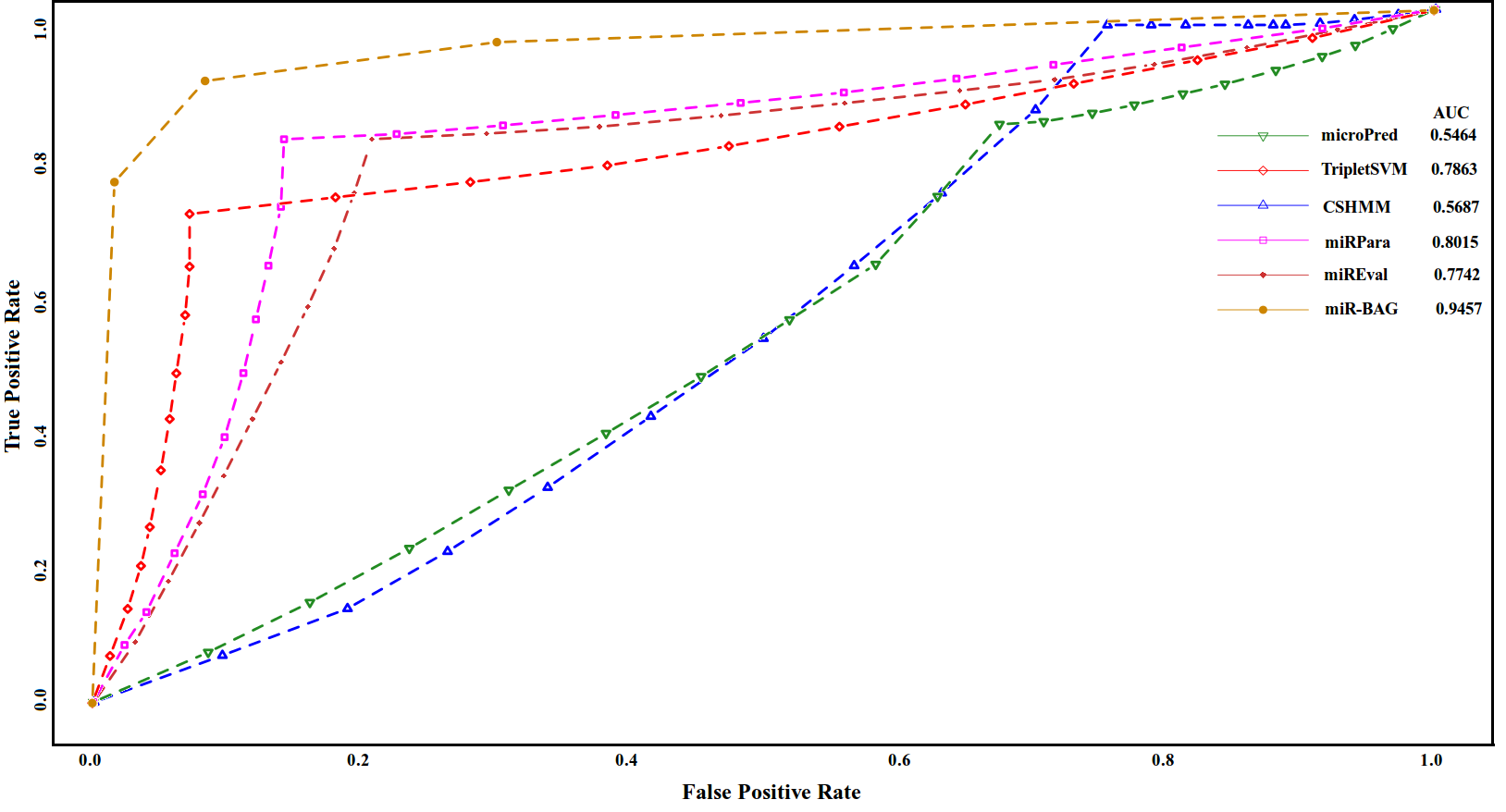

Supplement: Supporting Material S6 — Comparative ROC plots for different precursor identification tools. (TIFF) [file pone.0045782.s006.tif]
