# Supplementary material for: miR-BAG: Bagging Based Identification of MicroRNA Precursors
Source: PLoS One. 2012 Sep 25;7(9):e45782. doi: 10.1371/journal.pone.0045782 (PMC3458082; doi:10.1371/journal.pone.0045782)
Supplement: Supporting Material S7 — Sequence data for novel miRNAs identified in Bos taurus genome, using the NGS module of miR-BAG. (DOC) [file pone.0045782.s007.doc]

**Supporting Information S6: Sequences of *Bos Taurus* genome identified as potential miRNA precursor candidates.**

>sequence_582

UCUAUAGAAUUCUUCAGGCAAGAAUACUGGAGUGGGUUGCCAUUCCCUUCUCCAAGGGAUCCUAAAAUAGCAUACGGAUUUAUAAAUGAAAAAUCAGUUGUAGAUGGAGUGAUAAAAUUUUGCAGCUUUUGUAAGUUGUUGUUUUAGUAUCUUAUUUAACUAUAUAGUUUUAAACUAAGCUUUUAUUAUAAACAUAUGUG

>sequence_1333

CUGGUGGGGGUGGUCUGUGAUGAGGUUUAUCAUCUUUUGGGGGUGGUCUGUGAUGAGGUUUGCCAUCCUUUGGUGGUGGUCUGUGGUGAUGUUUACCAUGUUCUGGGGGUGUUCCUUGCUCAUUGUCAUCACCUUGUGUGGGUGGUUCUUGCUCAGCUUCAUCACCUUGUGCGGGUGGUCUGUGAUGAGGUUUACCAUCC

>sequence_1676

CUGUAUACAAACAAUCUGCAGGGCUGCGUCCACCUGGAGGGGGACCCUUUUCCUAAUUGCACGAAGUUGCCACAUUGUCUAGCAGCAGCCACCUUGUGUGGGUGCUCCGUGUGGGCAGUGAGGGGAGCAAGGCUGGAGAGGGUAUGGGGAUGUGACCAAGCCACUGCCCUGUAGCCAUCCUAGGACGGUCACCUCCCCGC

>sequence_1750

UGCCCUCAGUGGUGACUGUCUGUCUUUUGGCAAGCUUCUUAGGGCCAUCCGGUUGGAGUGGUUCUGCCUUUGAGGUUUCUGGGGACUCUGGGUUGAGGAGCGGGGCCUGAACUGUGGACCUUGUGUGGGUGCUGAGUGGUCUGGAACCUGGAGCUGCCCUGUGGGGAGAAGGUUAUCAGAGGGGUGUUAUCUCCAUUUCA

>sequence_3415

UUCGUGUCUGAGGAACAAAGGCCAUGGGAGAAGAACAGGAGAGCUGGGGGUUUGAGAAAAGGUGUGGUGGCCUCUGACAGCAGGCUCCUGAAGGAAGGCUUCCUGGAGGAGGUGGCCUGGGAGCCCUGCAGGACAGAUGGAGGAGGACUUUCCUGUGGGAAUCUAGUCCCCAUGGCUGGAGAACCAGGAGGGUCCGAGGA

>sequence_3880

UCUGAUAGGCACGUUCUCAUAGCUACCAAGGUGAUAUGUACUUUUUCCAUGCUUUAUUGAGAGUUACCUUGUUUGAACUUUACCAAUUUUUAUUGUAAGUAUGUUUUUACUUAUUUGUGGUGGUGGUUUACUCGGUAAGUCAUGUCCUACUGUUGUGACCCUAUGGACUUGUAGCCUGCCAGGCUCCUCUGACCAUGGGA

>sequence_6321

AGAAGUAAACUUGUCAAAAAGACACAUUUCUGAUAUGGAUGGGAAGAAACUGAAAUAUCCAGUAACAUUUAUAAACAAAGUUGAGUGAAAAGAGUUAUAAGCCAACUUAAACUUAAGUUUGUUUCAUAAACUGACUUUUGGAGUCAUUGUUUUGUAUGUCAAAAGCAUUGUGUUUAGAAUACAGCUUUUUGUUAAUGGCU

>sequence_7279

CCUCAGGUAAACCUAAUGUUCAAUUUAAAUCUGGGGGCAGAGCUAGAGAAGGCGUAAUCUUGGUGCCAGUCUUGAGUAGGAUGAAUUGUGAAGUAGACAUAACUGACCAUACAGUGGUGUCUAGCCUCAGAGCAUUGUGCUCAAACUCACUUUUAUACAAAAGUCAACAGACCUACCACUGGUGUAUAGACUUCGCUUUG

>sequence_7423

AAUUCUAUUUUUUUUCCCUAGACUAUCCAGGGAACUGCUCAUAGUAGUUCCCCUGUGGAAACAGGAACAUUCUUAGUCUGAAGGUUUUUUAUUAAUGUAACUAUUUGGUAGACUAUUAAAAGUUGUCUUGUACAAGGAUUCUCAAACUAUCUUUCAAUCUGUCAUAGACUAUGUGAUAACUUGUGGUAACUUGGCUGGGC

>sequence_8969

CCUACAAGGCGCUAUGAUAAAGUGAUAUGUCCUUCAAGGUGUAGACUGUAGAUCACUCGUGUCAGAAUCCCCUAAGUGUUAAACCCCCUGCCUCCAGACUCAGAACCUGGGGGAGAGGGGUGAUGCGGGGCAAUCAGCAUUGUGACUCCAAGACCAGCCAGGCUCUCCAGGUGGUCUUGGAGCCCAUAUCUGCUGGAGAA

>sequence_9091

CUAGGUUGAGAGCCCUUUAUGAGACUCCACUGCCUGUUGAUCUCAGGUGGAGCUGAGGCAGUGACGCUAGUGCCAGGGAGUGGCUGCAGUUACAGAGUAUCACGAGCAGAGAGGUAUGACUGCAGAGACCUGAAUAAAUGAGCUGCAGACUCGUGUCAGAACCCUGCCAGUGAGUGGCAAGUGACAGCUAAGCUGCACCU

>sequence_9129

GAUCUCAGGUGGAGCUGAGGCAGUGACGCUAGUGCCAGGGAGUGGCUGCAGUUACAGAGUAUCACGAGCAGAGAGGUAUGACUGCAGAGACCUGAAUAAAUGAGCUGCAGACUCGUGUCAGAACCCUGCCAGUGAGUGGCAAGUGACAGCUAAGCUGCACCUGGCGAAGUGUGUCCUUAUUUUACUGUAUAUCACAGUGU

>sequence_9241

CUGUCCCAGACUGGUCUUGCUGGACACGCGCCUCCCCAGGCCAUCGUAACGAUAGAUCACUGUCCAGCCGCUGCCUUUACUGUAGACUCGGGUCAGAAGCCCCUUGGAGCUGUACUCGAAGAUCUCGGUGCCUCUCUGGCGCAGGAAACCAUCUUCAUCCAGCCGGUACUGAACGUCACCCAGCCGGGUGAUCCUGUCUC

>sequence_12295

UUUUAUCAAAGCUUAAGACCUAUGAAAAGACAUCUAUUUAUUGAUUUGUCAGAUUCUUUUGAGAAUAAAGGUAUUUUGGUAUGCUUCAUAAAAAGAUAGUAACUGAAGUUAUCAUAAAGGUGUUUCUUAUGUAAGACAAACUGAGUACUUCUAUUAAUAAGCAGAAUUUCUUUUAUUUAAAGAAUGAAAACUUAUGAGAA

>sequence_12300

UCAAAGCUUAAGACCUAUGAAAAGACAUCUAUUUAUUGAUUUGUCAGAUUCUUUUGAGAAUAAAGGUAUUUUGGUAUGCUUCAUAAAAAGAUAGUAACUGAAGUUAUCAUAAAGGUGUUUCUUAUGUAAGACAAACUGAGUACUUCUAUUAAUAAGCAGAAUUUCUUUUAUUUAAAGAAUGAAAACUUAUGAGAAUCACU

>sequence_14389

UCCUUUUGCACCUUUGUUUACAAAGCUAAGAUUUCUUCUUCUUCAUUGAGGGAAACCAGUAGAUCACCAAGAAGUGUCAUCCGUCUUGAGACUGUUGCUUUUGCAAACCUAAGAAUGAUAUGCUCUUACCUGGUAGACUUUUCCAAACUUAUCUCAGGGAAAGUCAGCUAUCUGUGCCAAUCAAUCUGACCUCCUUGAAG

>sequence_14456

GGGAAUGGGGAAGGGUAAGAAUUGUUCUGAGAAUCAGGACAUUCUGUCGCAAUUUAGCCUCCAGACAGACGUUGGGCAAGUCAGUCUUACCUCUUGGGACUUUACUCAGAAGGGGCUGAAUUAGACUGUUGCUUUUGCUGUUUUGAGCUCUGAAGUUUUGUGAAUUUUUGUUUCUAUGAAGGCAUGUUACGUAUCUAUCA

>sequence_14560

UGCCUGGAGAAUCCCAGGGACGGGGGAGCCGUCUGGUGGGCUGCCGUCUAUGGGGUUGCUCAGAGUCGGACACGACUGAAGCGACUUAGCAGCAGAGUCAGAAUUCAAAUCCAGCUUGCUCUGAUUCUGUAUCAGACUGUUGCUUUUUCUAUUGCUACAUAAGAAUACUACUUUCUGUGUGAUAUUGGUCCACAGAAUCU

>sequence_14691

AUGCAUUUAACGUUUCCAUCGUAAGACUUCUCUCUGCUGAUGGGAUUUGGAAAGCUGGCCUCUAGGAAAAGCCCAGCGUAGGAAUCUCCUGGGAGCAGCGAGCAGACUGUUGCUUUUUCCUGACUUCCAGCAAGAUCUUAAUUUUUCCUGACUUCCAGCAAGAUCUUAAUUUUUCCUGACUGUUUAUGCACCUUCCACCA

>sequence_14709

UCGUAAGACUUCUCUCUGCUGAUGGGAUUUGGAAAGCUGGCCUCUAGGAAAAGCCCAGCGUAGGAAUCUCCUGGGAGCAGCGAGCAGACUGUUGCUUUUUCCUGACUUCCAGCAAGAUCUUAAUUUUUCCUGACUUCCAGCAAGAUCUUAAUUUUUCCUGACUGUUUAUGCACCUUCCACCACACCAGAAAGCUUCUGGG

>sequence_15142

AUGGAAACCUGCAGGGGUCAGGGUCUACCACCUGGUGAAGACCCACUGGAGAGUGAAGAUUUCAGUAGGCAAAAUGGAGCUGGAAAGGGAAAAAGAGGCGUCUAUAGAUUCUUUUCUUAAAUAUUUAUUUAAUUUAUUAGGCUGUGACAAGUCUUAGUUGCAGCAUGCAACUCUUUAGUUGGGGCAUGCCAACUCUUAGU

>sequence_15200

GAGGUGCCAGGGUAAGUCAGGAGGCAGCGGGAGCAAGGGGAAAGCGUGGGUGAGACAGGAUAGGGGAGCCGAACAAGAUUAGGGUUAGUCAGCUCACAUAGAUUUCAGUAGGCAGUGGGUAAUGGGUGGUCUCUAAGUGUCGGGUACCUGGCCGUGGGGGAUUUCAGGCAGAGGAAUAGUGUCCUGGUGAGGGCUAUGAG
