# Supplementary material for: miR-BAG: Bagging Based Identification of MicroRNA Precursors
Source: PLoS One. 2012 Sep 25;7(9):e45782. doi: 10.1371/journal.pone.0045782 (PMC3458082; doi:10.1371/journal.pone.0045782)
Supplement: Supporting Material S8 — Web-server and standalone implementation details for miR-BAG. (PDF) [file pone.0045782.s008.pdf]

Server and Standalone implementation

Input to web server for sequence analysis (Figure 1)

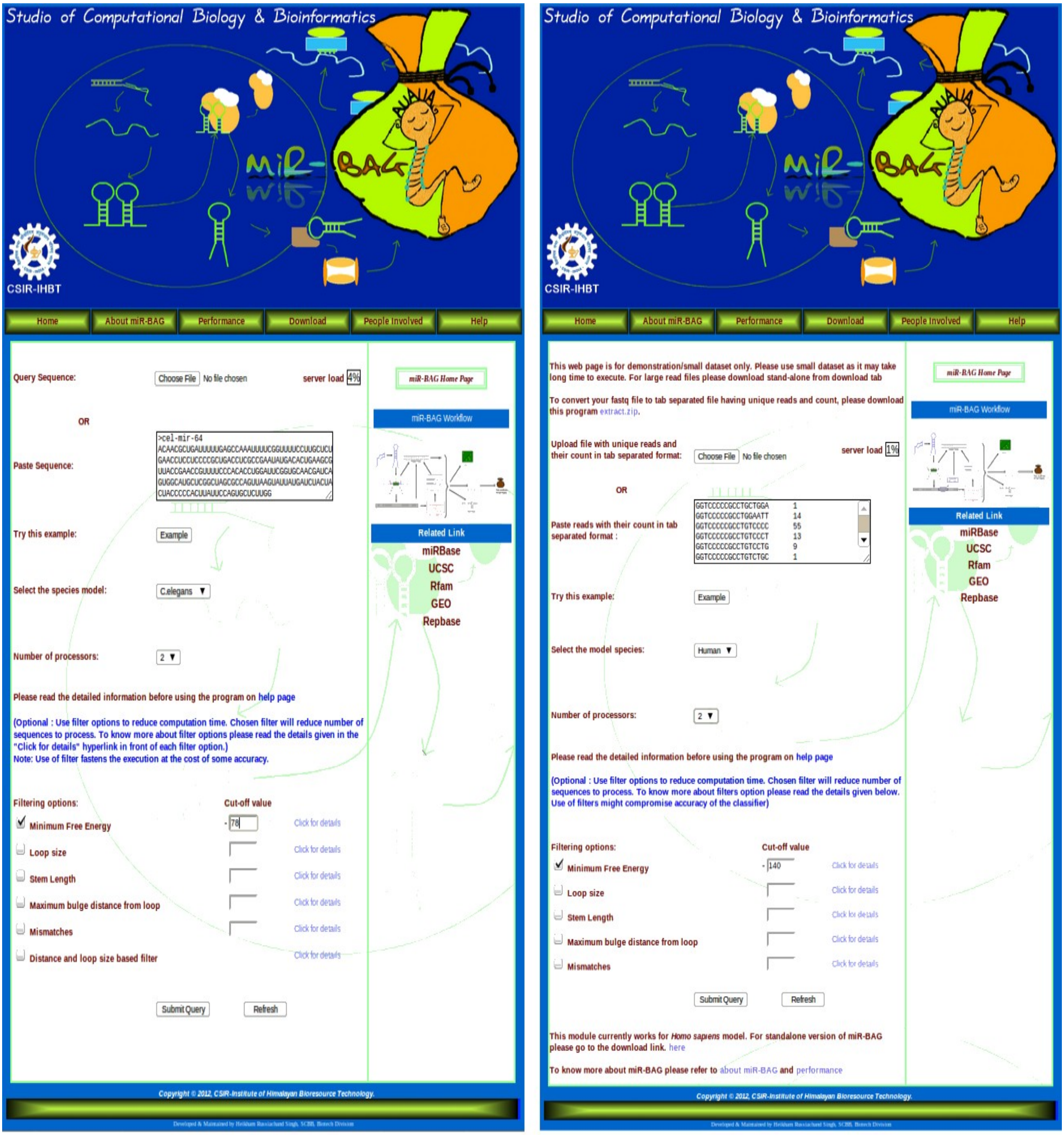

a) Simple sequencescan web-page

b) NGS data scan web-page

The web-server for miR-BAG has been developed in HTML and PHP, on Linux platform (Ubuntu 10.04 LTS version), while successfully tested on other flavors of Linux like CentOS and Fedora (See the figure 1).

All the back-end programs are developed in JAVA and PERL programming languages along with Linux shell scripting. The input file format for sequence analysis requires a FASTA header with sequence. The minimum length of sequences needs to be 200 bases with maximum length of 300 bases. User can upload the sequence file or paste the sequences in the paste sequence box. After submitting the sequences, choice is made for the species model to be considered for the classifier. The web server also provides an option to the user to run miR-BAG on multiple processors. The output consists of sequence ID, start position of the putative precursor sequence found, end position of the putative precursor sequence and classification score. The classification score could be useful for the user to pick the right candidate. The average distribution of various instances into these four scoring categories, as tested for human datasets, is shown in figure 2 below:

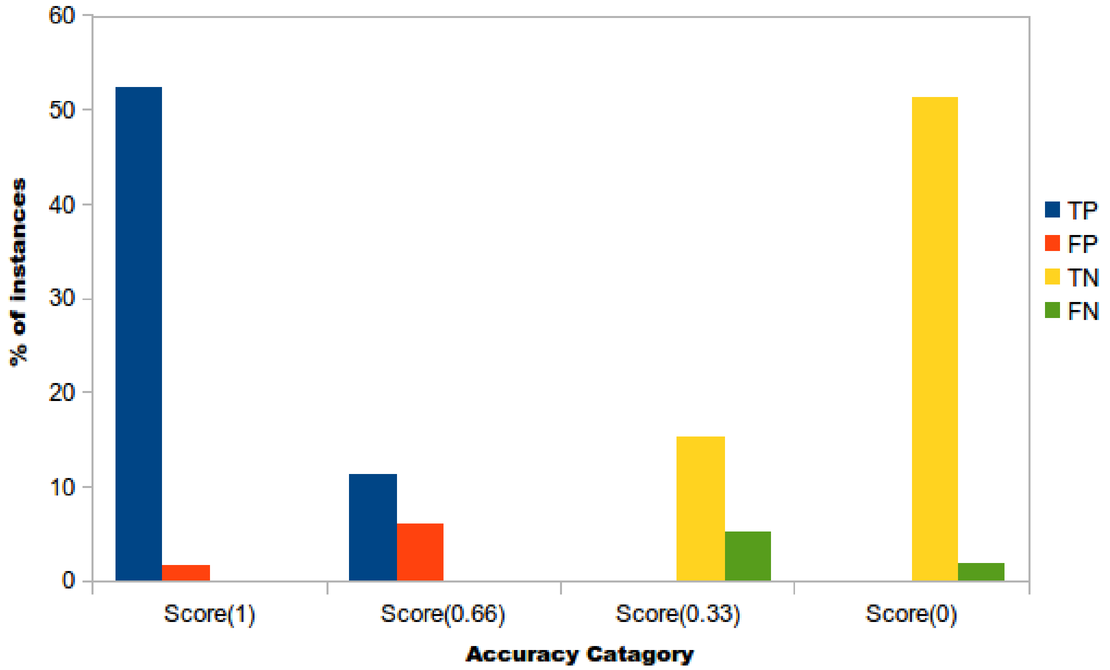

(Figure 2)

This suggests that sequence having a score 0.6 or above is considered as positive miRNA candidate, while score of 1 suggests the highest confidence. Besides this, the server also provides an initial filtering option to fasten the execution at the cost of some accuracy. This filter is based on six different conventional properties, applied for long time in miRNA candidate identification. A user is given choice to choose any combinations of these filters to put cut-offs based on the MFE, structure, loop size, bulges and their distance from loop, etc. The cut-off values can be adjusted in an equalizer mode. This is suggested that in case of longer and larger amount of sequences, the user should opt for the stand-alone version, which is also independent of query sequence length and amount.

Input to web server for NGS data analysis

The web server also provides NGS **module for miRNA discovery**. The Input file to submit read sequences to web server needs to have a tab separated unique read file with read count. A Java code has been provided to convert the FASTQ data file into unique reads with their corresponding count values in tab separated format. This code can be downloaded from the server page. Once the unique reads with count file is submitted, the read input is searched against pre-miRNA, non coding RNAs and mRNAs of user specified species. All those reads which do not map to any of the previously annotated sequences are taken forward to identify potential novel miRNA regions using miR-BAG methodology. RPKM (Reads Per Kilobase of exon model per Million) derived abundance measurement of known as well as novel candidates is also displayed in the output.

Stand-alone version

Large scale and genome level analyses essentially requires user friendly standalone version. For analysis over large number of sequences, high throughput data and genomic sequences, the stand-alone version of miR-BAG has been developed with a user friendly GUI. It has sequence as well as NGS based modules (See the figure below).

Like the web server version, both of its modules have been implemented with parallel architecture. The GUI of stand-alone has been developed in QT4 C++, which can be installed on Linux O.S. with QT4 library. RNAfold [31] has been used for RNA secondary structure determination and Bowtie for read mapping. These tools need to be installed on the users machine. To run miR-BAG sequence module, the user has to upload the sequence file, choose the species model and provide the number of processors to execute miR-BAG. The sequence file may have multiple query sequences in fasta format, without any size and amount restriction. Once the analysis is complete, a message box appears asking the user to refer to the result file in the output destination. The result file is named as "miR-BAG_result_file", which appears in the output destination specified by the user. It consists of sequence ID, start position of best precursor/candidate sequence found, its end position, putative precursor/candidate sequence of length 200bp and associated classification score. The above mentioned server's scoring scheme is followed here too.

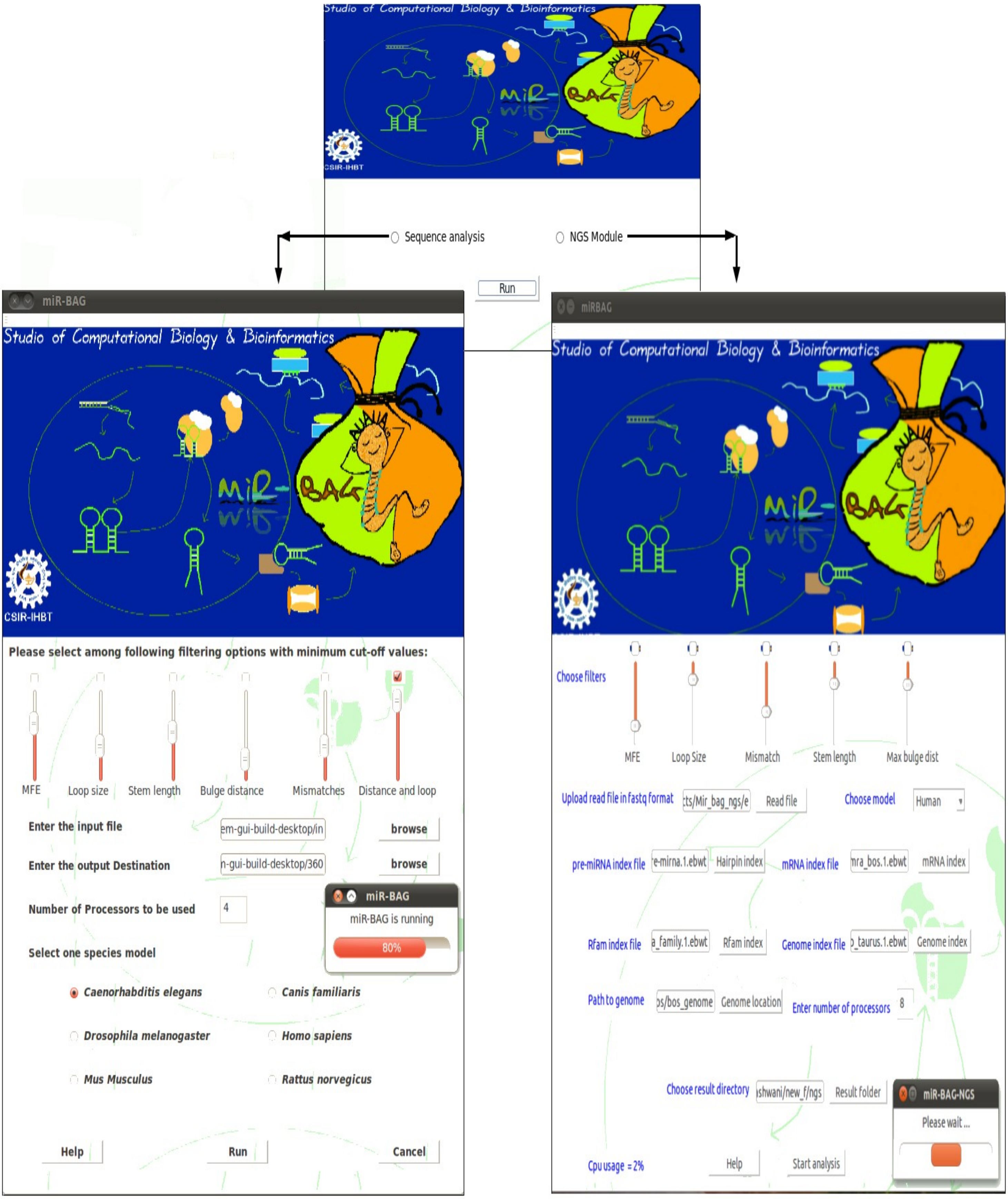
Studio of Computational Biology & Bioinformatics
CSIR-IHBT
Sequence analysis
NGS Module
Run
miR-BAG
Please select among following filtering options with minimum cut-off values:
MFE
Loop size
Stem length
Bulge distance
Mismatches
Distance and loop
Enter the input file
em-gui-build-desktop/in
browse
Enter the output Destination
n-gui-build-desktop/360
browse
Number of Processors to be used
4
miR-BAG is running
80%
Select one species model
Caenorhabditis elegans
Canis familiaris
Drosophila melanogaster
Homo sapiens
Mus Musculus
Rattus norvegicus
Help
Run
Cancel
miRBAG
Choose filters
MFE
Loop Size
Mismatch
Stem length
Max bulge dist
Upload read file in fastq format
Read File
Choose model
Human
pre-miRNA index file
Hairpin index
mRNA index file
mRNA index
Rfam index file
Rfam index
Genome index file
Genome index
Path to genome
Genome location
Enter number of processors
8
Choose result directory
Result folder
miR-BAG-NGS
Please wait ...
Cpu usage = 2%
Help
Start analysis

(Figure 3)

For miR-BAG NGS module, a user can submit the FASTQ file directly to the program along with full path for hairpin indexes, Rfam indexes and genome indexes formed by bowtie-build. Also the user needs to choose the number of processors to be alloted to run miR-BAG in parallel mode. The NGS module of miR-BAG first counts the number of unique reads from the FASTQ file and a unique read file is generated, containing reads with corresponding count number. The unique reads are mapped on hairpin sequences, non coding RNA sequences and mRNA sequences to remove already annotated RNA using Bowtie. The reads which do not map to any of the annotated sequences are mapped on genome to find the genomic coordinates. Using these coordinates, the sequences with 250 bp are extracted (125 upstream and 125 downstream). Considering 250 bases length has reasoning that this length covers whole pre-miRNA region as well as also meets the minimum length requirement of miR-BAG. The final output consists of reads, associated sequences identified as potential miRNA candidate regions, with score and the abundance values. With this all, the NGS module of miR-BAG becomes highly useful tool for NGS based data analysis and miRNA discovery.
